# Sensitivity Analysis of Flux Determination in Heart by H_2_^18^O -provided Labeling Using a Dynamic Isotopologue Model of Energy Transfer Pathways

**DOI:** 10.1371/journal.pcbi.1002795

**Published:** 2012-12-06

**Authors:** David W. Schryer, Pearu Peterson, Ardo Illaste, Marko Vendelin

**Affiliations:** Laboratory of Systems Biology, Institute of Cybernetics, Tallinn University of Technology, Tallinn, Estonia; University of Auckland, New Zealand

## Abstract

To characterize intracellular energy transfer in the heart, two organ-level methods have frequently been employed: 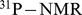 inversion and saturation transfer, and dynamic 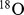 labeling. Creatine kinase (CK) fluxes obtained by following oxygen labeling have been considerably smaller than the fluxes determined by 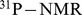 saturation transfer. It has been proposed that dynamic 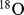 labeling determines net flux through CK shuttle, whereas 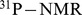 saturation transfer measures total unidirectional flux. However, to our knowledge, no sensitivity analysis of flux determination by oxygen labeling has been performed, limiting our ability to compare flux distributions predicted by different methods. Here we analyze oxygen labeling in a physiological heart phosphotransfer network with active CK and adenylate kinase (AdK) shuttles and establish which fluxes determine the labeling state. A mathematical model consisting of a system of ordinary differential equations was composed describing 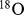 enrichment in each phosphoryl group and inorganic phosphate. By varying flux distributions in the model and calculating the labeling, we analyzed labeling sensitivity to different fluxes in the heart. We observed that the labeling state is predominantly sensitive to total unidirectional CK and AdK fluxes and not to net fluxes. We conclude that measuring dynamic incorporation of 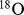 into the high-energy phosphotransfer network in heart does not permit unambiguous determination of energetic fluxes with a higher magnitude than the ATP synthase rate when the bidirectionality of fluxes is taken into account. Our analysis suggests that the flux distributions obtained using dynamic 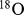 labeling, after removing the net flux assumption, are comparable with those from 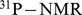 inversion and saturation transfer.

## Author Summary

In heart, the movement of energy metabolites between force-producing myosin, other ATPases, and mitochondria is vital for its function and closely related to heart pathologies. In addition to diffusion, transport of ATP, ADP, Pi, and phosphocreatine occurs along parallel pathways such as the adenylate kinase and creatine kinase shuttles. Two organ-level methods have been developed to study the relative flux through these pathways. However, their results differ. It was recently demonstrated that  studies often suffer from the exclusion of compartmentation from their metabolic models. One study overcame this limitation by using compartmental models and statistical methods on multiple experiments. Here, we analyzed the sensitivity of the other method - dynamic  labeling of phosphoryl groups and inorganic phosphate. For that, we composed a mathematical model tracking enrichment of the metabolites and evaluated sensitivity of labeling to different flux distribution scenarios. Our study shows that the dynamic  method provides a measure of total flux, and not net flux as presumed previously, making the fluxes predicted from both methods consistent. Importantly, conclusions derived on the basis of  labeling analysis, particularly those regarding the net flux through the shuttles in control and pathological cases, need to be reevaluated.

## Introduction

In heart, the mechanisms that ensure energy production meets demand over a wide range of workloads, remains unclear. Fundamental to this search is an accurate understanding of the recycling fluxes of ATP, ADP, Pi, and phosphocreatine (PCr) between the mitochondrial inner membrane space and the ATPases on both the myofibrils and sarcoplasmic reticulum. In highly compartmentalized environments, such as heart muscle [1]–[8], determination of energy transfer fluxes is far from trivial. The organ level methods used to determine the fluxes include 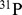-NMR inversion and saturation transfer, and the transient labeling of 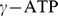, 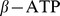, 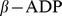, and Pi using 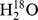, a method we refer to as dynamic 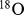 labeling. To estimate the fluxes using 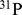-NMR inversion and saturation transfer, magnetization transfer has been simulated in the compartmentalized system and fitted against experimental data [9]–[12]. A recent study [12] employed rigorous statistical testing of model solutions against experimental data using stochastic models that described measurement uncertainty. Such analysis was applied to discriminate between energy transfer pathways over a range of cardiac performance levels. These results suggest that at least 40% of the energy is exported via direct ATP transfer at high cardiac performance. This method is unable to determine the split between direct ATP transfer and the creatine kinase (CK) shuttle at lower cardiac performance. While the split has not been determined for all conditions, total CK unidirectional flux was found to be stable if energy demand was changed either by variation of extracellular calcium or by left ventricle balloon volume in isovolumetric contractions.

There are several complications that have to be considered when interpreting magnetization transfer experiments. As summarized recently [13], [14], magnetization transfer interpretation should be based on a complete model of the biochemical reactions that could contribute to the measured transfer. For example, oversimplified interpretation of saturation transfer experiments to determine the ATP synthesis flux by analyzing the rate Pi and ATP can lead to overestimated values for ATP synthesis [13]. A recent study overcame this limitation by analyzing 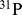-NMR inversion and saturation transfer results using compartmental models and statistical methods on multiple experiments [12].

**Figure 1:**
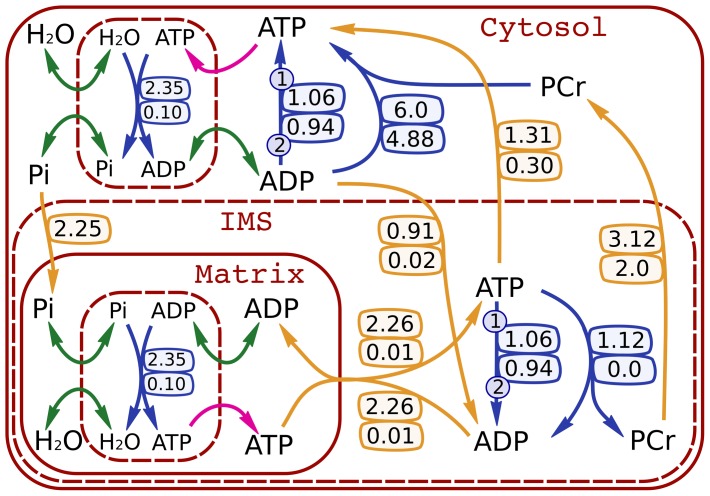
Heart phosphotransfer network used as the basis of the model in this work. CK fluxes are taken from Vendelin et al. [12]. The AdK fluxes are taken from flux distribution 1 found in Table S1 where it was assumed that the ratio of total CK fluxes to the total AdK fluxes is the same as the ratio of the activity measurements made by Aksentijević et al. [47]. Enzymatic fluxes are given in blue and transport fluxes are given in orange with arrows indicating the direction of positive net flux. Bidirectional fluxes have two bubbles attached to their arrows, with the upper bubble showing the forward flux and the lower bubble the reverse flux (net flux is forward minus reverse). Unidirectional fluxes have arrows with one bubble that indicates the forward flux. The numbers on the AdK reaction arrows indicate stoichiometry. To compare the influence of bidirectional and net fluxes we introduce two *total* fluxes, one each for AdK and CK. The total flux through the CK shuttle is the sum of unidirectional reactions in the mitochondrial intermembrane space (IMS) and cytosol that proceed towards PCr (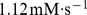
+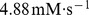), and the total AdK flux is the sum of unidirectional AdK reactions in the IMS and cytosol that proceed towards ADP (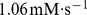
+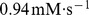). The magenta arrows indicate unidirectional substrate fluxes, while the green arrows show bidirectional substrate exchange fluxes. The intermediate enzyme bound state of both ATPase and ATP synthase is reversible with respect to 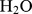 and Pi (see Methods).

Dynamic 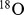 labeling has also been employed to measure the fluxes of high-energy metabolites [15]–[28], although no study to date has measured the cardiac performance dependence of these fluxes [29]. The dynamic incorporation of 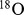 into 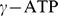, 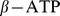, 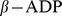, and Pi was quantified using mass spectrometry in earlier papers, and more recently using 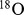-assisted 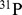-NMR [20]. From the 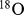 labeling data in [28], a linear relationship was found between rate pressure product after ischemia-reperfusion recovery of individual hearts and energy transfer through CK. This relationship was identified due to variation of heart recovery after being exposed to ischemia-reperfusion with and without preconditioning. In control conditions, before exposure to ischemia, the flux through CK was estimated to be 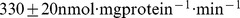 which converts to 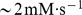 (see Methods for conversion factors). This is considerably smaller than the total CK unidirectional flux 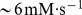 in [12] and other 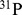-NMR saturation transfer studies [30]–[38]. However, in contrast to 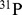-NMR saturation transfer studies, it is proposed that flux estimation on the basis of 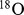 labeling leads to the estimation of *net* flux through CK shuttle system [20], [24]. Definitions for net and total flux are provided in Figure 1. Usually, labeling by isotopic tracers depends on the bidirectionality of reactions which allows transfer of the label in the opposite direction to net flux in the system [39]. Considering that the adenylate kinase (AdK) and CK reactions are reversible, it is expected that 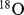 labeling could be influenced by the bidirectionality of the reactions, as well as the net flux through the shuttles.

The incorporation of 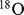 into the phosphoryl groups of ATP and Pi has been used in many studies including analysis of the ATP synthase mechanism [40] and cyclic nucleotide metabolism [41]. To study cyclic nucleotide metabolism, the rate of 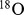 incorporation into the 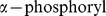 groups of guanine nucleotides was measured to calculate the change in cyclic GMP and cyclic AMP flux [41]. Another study by the same group used the rate of 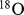 incorporation into the 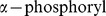 groups of ADP and ATP to calculate cyclic AMP fluxes in human platelets [42]. A number of years later the same group developed a technique that is able to determine the rate of ATP hydrolysis (and synthesis) by exchanging 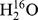 with 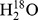 and tracking the time course of 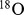 incorporation into 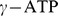 [43]. In contrast to 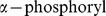 groups, 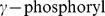 groups are exchanged much more often, and this technique was deemed to be unreliable if Pi and ATP also participate in reactions that have a higher magnitude of flux than the synthesis rate [43], such as the CK reaction in isolated rat heart [31].

The 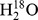 labeling technique was then applied in a long series of papers that explore the fluxes in the phosphotransfer networks of both heart and skeletal muscle [15]–[28], [44], [45]. While it was stated in [20], [22], [24], [25], [46] that labeling was analyzed using models, the details of these models were not presented. Despite this, it was further stated that the method determines net flux through the individual phosphotransfer pathways. For determination of net flux through the CK shuttle, a relationship between PCr labeling and CK flux was established on the basis of gradual inhibition of CK [16], [27]. However, to our knowledge, no sensitivity analysis of this method of flux determination has been performed which limits our ability to compare flux distributions predicted by 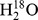 labeling, and 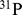-NMR inversion and saturation transfer. In addition, we are unaware of any comprehensive kinetic analysis of 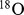 labeling on a phosphotransfer network in its entirety since the work by Dawis et al. [43].

The aim of this work is to analyze the properties of 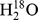-provided labeling in a physiologic heart phosphotransfer network with active CK and AdK shuttles and establish which fluxes determine the labeling state. For this, we use an integrated kinetic model able to predict the dynamic 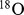 labeling state to assess if the labeling state is sensitive to net fluxes when enzymatic and transport fluxes are not assumed to be unidirectional.

## Results

An integrated kinetic model was constructed to account for all isotope transformations that occur in the high-energy phosphotransfer network reactions in heart (Figure 1). The dynamic transformation of isotopologue species occurs while the phosphotransfer network reactions proceed at a steady rate. This kinetic model is able to simulate the dynamic change in 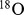 enrichment in each phosphoryl group and phosphate ion given a steady flux distribution and a time dependent function of the ratio of 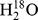: 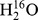. Note that the labeling state of 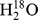 is simulated in enzyme bound compartments. Details of the model construction are provided in Methods.

### Model validation

**Figure 2:**
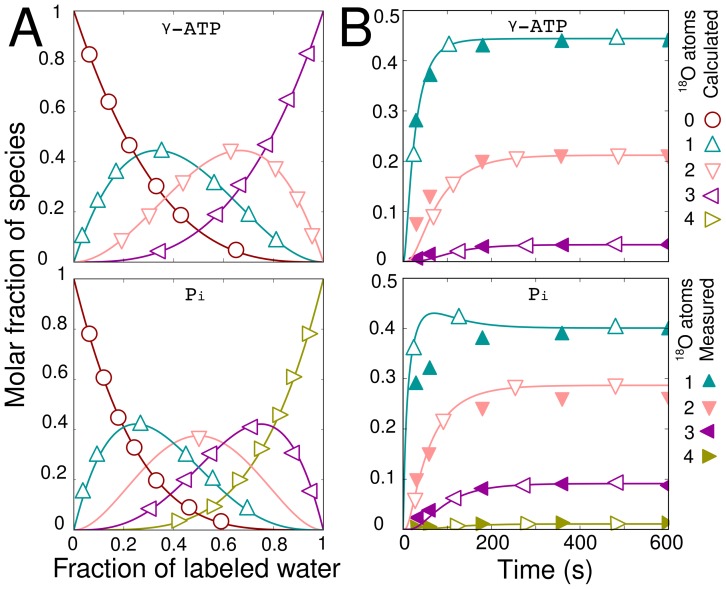
Model validation using the steady state labeling distribution and dynamic human platelet results in [43]. All labeled species are plotted separately with the number of attached 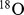 atoms indicated by symbols. Measured data points from [43] are filled; Calculated points from [43] are unfilled; Lines represent simulations performed in this work. Subplot A shows the labeling distribution found at isotopic steady state as the 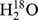 is ranged from zero to 100% 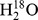. The steady state points are identical to those predicted in [43]. Subplot B is a fit to the dynamic human platelet data in [43] (37 C, 32.3% 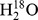.). The model in [43] is reproduced by setting the CK flux equal to zero and assuming all reactions are unidirectional. Note that the results of our simulation match with the simulation results in [43]. The parameters 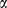 and 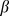 from [43] were used to find the ATP pool (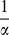), and Pi pool (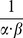).

Prior to conducting the sensitivity analysis a number of model validation steps were performed. First, we tested if the steady state labeling distribution predicted by the model matches the theoretical distribution provided by Dawis et al. [43]. Following this we compared the dynamic predictions of our model to the dynamic predictions in [43]. Figure 2 shows that both the steady state and dynamic labeling state predictions provided by our model are indistinguishable from those provided by the model developed by Dawis et al.

**Figure 3:**
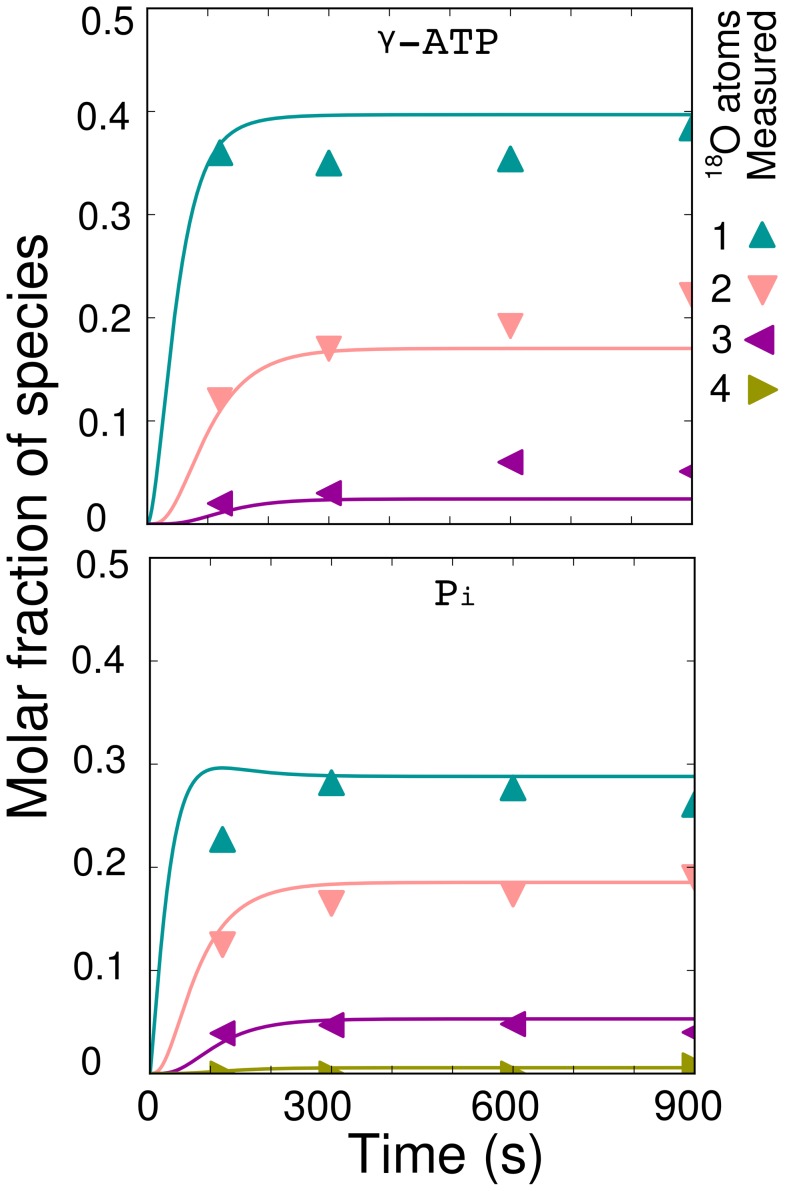
Model simulation together with dynamic 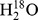 rat atrial tissue results from [28]. The fluxes used to simulate the labeling state plotted here correspond to flux distribution 1 from Table 1, however, a wide range of flux distributions are able to fit this data. This distribution uses the same direct ATP and CK fluxes as those predicted by 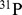-NMR inversion and saturation transfer in [12], and uses the activity measurements in [47] to estimate the AdK flux. The exchange of 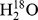 occurs over 30 s and the isotopic labeling state is seen to reach its equilibrium value at 120 s as stated in [20]. The labeling state curves take into account the active metabolic pool sizes of 90% ATP and 70% Pi reported in [28].

Additionally, it is important to ensure that the model we constructed is able to provide predictions that adequately match published dynamic 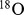 labeling data measured in heart. In most studies, the fluxes derived from organ-level labeling are published without the corresponding labeling dynamics of individual species. However, a recent paper does provide dynamic measurements of the four labeled species of 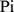 (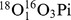,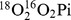, 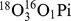, 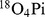) and the three labeled species of 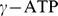 (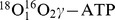, 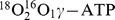, 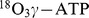) that result after surgically removing hearts from anesthetized male rats, and immediatly immersing harvested atrial tissue in Krebs-Henseleit solution enriched with 30% 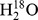 [28]. As a test we used the first flux distribution from Table 1 and simulated the labeling state. This flux distribution is based on a flux distribution found using organ-level rat heart 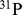-NMR inversion and saturation transfer results from [12] together with an estimation of the AdK flux from activity measurements [47]. We found that without any fitting, the model prediction could explain the 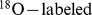 atrial tissue data in [28] (see Figure 3). Note that such close similarity between the measurements and the model solution was obtained using a flux distribution that corresponds to significantly different conditions (isovolumetrically beating heart vs isolated atrial tissue). It is clear from Figure 3 that the model does not exactly match the 120 second data point for 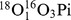. This species of Pi is the entry point of 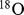 into the model and the overshoot in predicted labeling is analogous to that seen in Figure 2B and discussed in [43]. The dynamic overshoot in 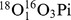 is less dramatic than in Figure 2B due to the differences in the flux distributions used and inclusion of CK reactions. The absence of the overshoot in the measurements can be due to the gradual labeling dynamics of water (the simulation uses a perfect step change), compartmentation of Pi, and the influence of reactions not considered in the model. The available data does not contain enough information to warrant the changes required for our model to fit this one datapoint. This is especially true because no estimation of measurement error was provided in [28], so it is not possible for us to quantify the goodness of fit. However, while comparing the model solution to this measured data we found that many possible flux distributions provide labeling predictions that also adequately explain the measured data.

**Table 1:** List of flux distributions considered in this paper.

Flux distribution	Percentage of energy export			Bidirectional fluxes	
	AdK	ATP	CK	CK	AdK
	shuttle	diffusion	shuttle	shuttle	shuttle
1†	5	45	50	**√**	**√**
2	5	45	50	**√**	**√**
3	5	45	50		**√**
4✠	5	45	50	**√**	
5✠	5	45	50		
6	0	50	50	**√**	**√**
7	0	0	100	**√**	**√**
8	5	0	95	**√**	**√**
9	5	95	0	**√**	**√**

These flux distributions were selected to range between physiologically feasible states while considering both unidirectional and bidirectional flux cases. Supporting Table S1 contains the flux values.

† Relatively high total AdK flux, taken from [47].

✠ Unidirectionality leads to low total AdK flux.

### Model sensitivity

**Figure 4:**
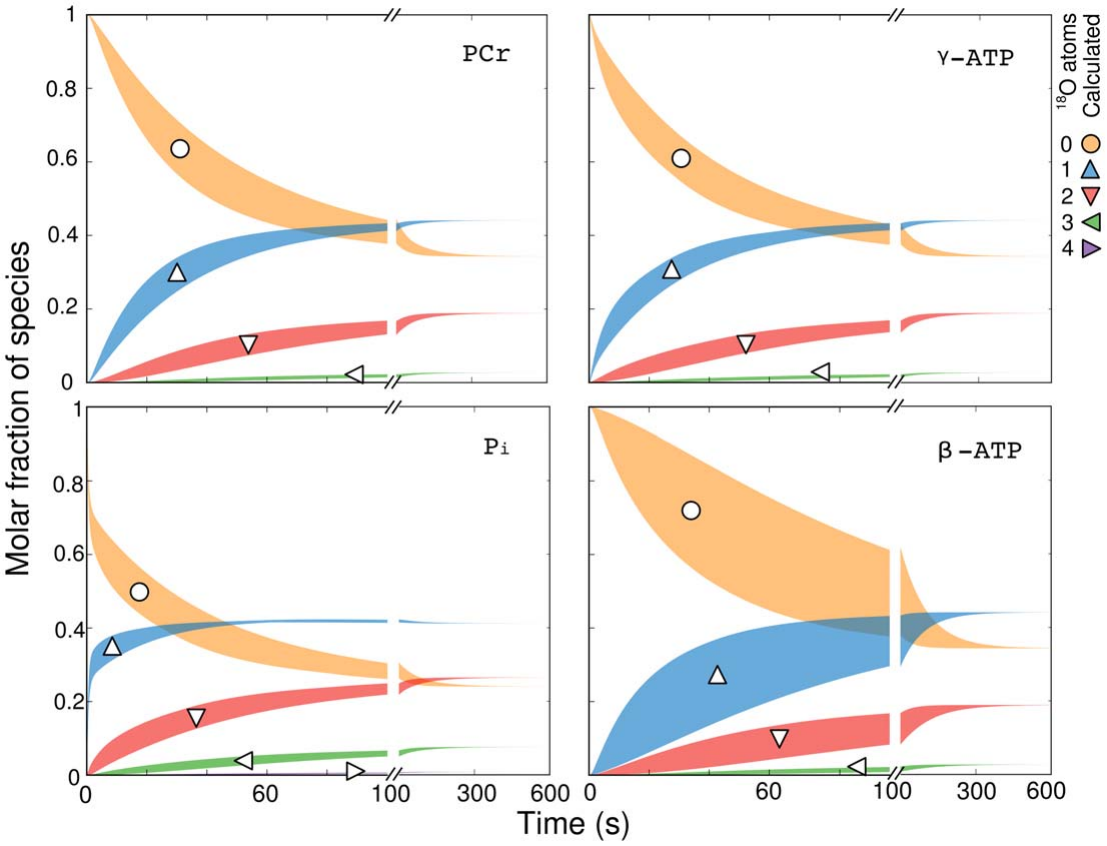
Influence of bidirectional fluxes and metabolic pool sizes on the predicted labeling state. Keeping the cardiac performance constant and using net fluxes from flux distribution 1 within Table 1, all other parameters (18 bidirectional fluxes and 18 metabolic pool sizes) in the model were varied over wide physiological ranges (Table S2 provides the ranges simulated). The upper and lower bounds of the predicted labeling state over all of these simulations are shown with filled regions. Regions with maximum spread are most sensitive to these parameters. Note that the labeling state of 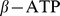 is the most sensitive to changes in bidirectional fluxes and metabolic pool sizes. Symbols indicate labeled species.

**Figure 5:**
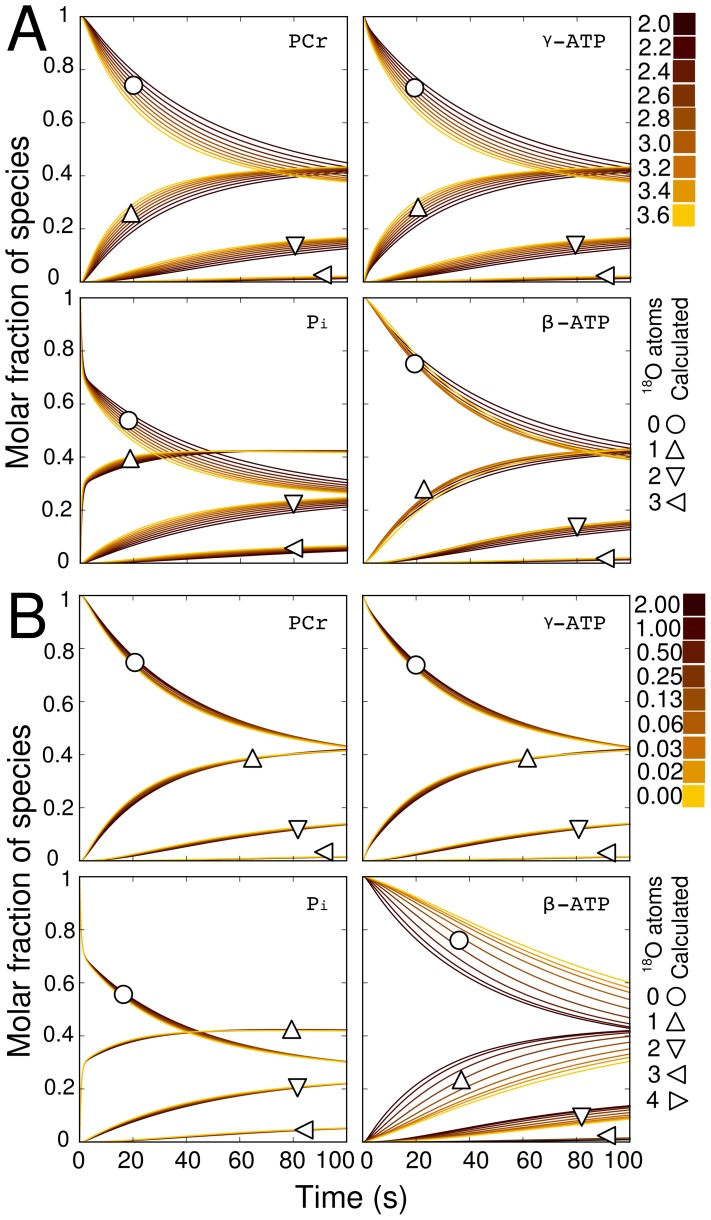
Influence of heart cardiac performance and AdK flux on the predicted labeling state. Subplot A shows the labeling state predicted using flux distribution 1 within Table 1 while varying the ATP synthase flux, shown with a color gradient. Note that the total CK and total AdK fluxes were kept constant while changing the ATP synthase flux by changing the CK and AdK exchange fluxes. The AdK exchange fluxes in the cytosol and IMS were changed by the same amount, while the CK exchange flux in the IMS was maintained at zero, i.e. only the cytosolic exchange flux was adjusted to maintain a constant total CK flux. Subplot B shows the labeling state predicted using flux distribution 1 while varying the total AdK flux, shown with the same color gradient. The units in both legends are 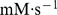. Note that the labeling state is more sensitive to changes in lower magnitude total AdK fluxes. As the AdK flux approaches the ATP synthase rate (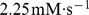), the predicted labeling state becomes insensitive to changes in the AdK flux. Symbols indicate labeled species.

Following model validation, we performed a sensitivity scan of the model parameters. The most sensitive parameters were found to be the ATP synthase rate as well as the net and exchange fluxes through the AdK shuttle. Figure 4 provides upper and lower bounds for the dynamic labeling state predictions when the cardiac performance and net fluxes in the system are kept constant. Note that the upper and lower bounds are formed from multiple simulation results. We varied six enzymatic exchange fluxes (AdK (2 fluxes, one in cytoplasm and one in mitochondria), CK (2), ATP synthase, ATPase), 12 transport exchange fluxes (Pi (2), water (3), ADP (4), ATP (2), PCr (1)), and 18 pool parameters (ADP (5), ATP (5), Pi (4), PCr (2), water (2)). The six enzymatic exchange fluxes were varied over five evenly spaced points each for a total of 15625 combinations. Small and large pool size sets were constructed based on their mean values and standard deviations reported in [12]. Two transport exchange flux sets with high and low values were used. This gives a total of 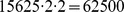 combinations. Additional combinations of pool parameters could increase the upper and lower ranges of sections of these curves, however, the change is not expected to be pronounced because the total pool size of the metabolites in this model are well characterized [12]. Table S2 provides upper and lower ranges for all 36 parameters that were varied. The labeling state of 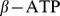 is most sensitive to changes in metabolic pool sizes and exchange fluxes. It was found that AdK exchange fluxes influence the 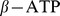 labeling state, and not the labeling state of other species. Figure 5 illustrates how the transient labeling predictions change when the ATP synthesis rate or the total AdK flux is changed.

Referring to Figure 5, the ATP synthesis rate is seen to influence the labeling state of all species, however, the total AdK flux mainly influences the labeling state of 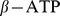. Note that the combined sensitivity of all exchange fluxes and pool sizes is roughly the same as the influence of the ATP synthesis rate.

### Physiological sensitivity

The main application of dynamic 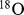 labeling analysis is for the determination of intracellular flux distributions. In general, to be able to determine the flux distributions, the method must be sensitive to variations in the fluxes. In the case of dynamic 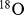 labeling analysis, the 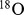 labeling of species has to be sensitive to the changes in the fluxes of reactions considered. Having determined which model parameters influence labeling state predictions most, we tested if the model is able to distinguish between various flux distributions that are physiologically possible within the context of the model we present. We restrict the physiological sensitivity analysis to a heart performance where predictions of the energetic fluxes in rat heart are available. To simplify the initial phase of the physiological sensitivity analysis, the bidirectionality of transport reactions as well as pool size parameters were held constant. The combined influence of these parameters, together with the physiological parameters, provide similar predictions of the labeling state (see Figure 4), and thus can be treated sequentially in a more general sensitivity analysis.

In total, nine flux distributions were studied. These flux distributions were selected to test the labeling sensitivity to the changes of specific fluxes, as explained below. General descriptions of these are provided in Table 1; Table S1 provides a detailed summary of the fluxes. Flux distributions 1 through 6 have roughly equal contributions to the net export of energy via direct ATP transfer and the CK shuttle. Flux distributions 6 and 7 have zero net flux through the AdK shuttle. Flux distributions 7 and 8 have 100% and 95%, respectively, of the energy exported via the CK shuttle, while flux distribution 9 exports 95% via direct ATP transfer.

There are several specific questions that are of interest when studying the flux distributions. First, what is the magnitude of the fluxes? In terms of dynamic 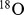 labeling analysis: How does the labeling state change when the activities of CK and AdK are increased? Comparing the simulated labeling states of flux distributions 1, 2, and 4 could provide insight into the effect of increasing the activity of AdK, while comparing flux distributions 2 and 3 could provide insight into the effect of increasing CK activity. Comparison of flux distribution 5 to the above tests reducing the total flux of both enzyme systems (CK and AdK) simultaneously.

Another question of interest is: Do net flux and bidirectional enzyme activity have different influences on the labeling state? Comparing flux distributions 2 and 6, as well as 7 and 8, allows one to test if the labeling state is determined by net AdK flux, or bidirectional AdK enzyme activity. Note that in those pairs of the flux distributions, the net flux of AdK shuttle changes.

Finally, how does the labeling state change when the net transport of energetic phosphoryl groups occurs via the CK shuttle, or direct ATP transport? Insight into this important question could be provided by comparing flux distributions 2, 8, and 9.

Additional combinations of these flux distributions allow for additional comparisons. Transient solutions for these nine flux distributions after a step to 30% 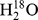 and 100% 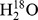 are provided in Figure S1.

To compare the different influence of bidirectional and net fluxes we introduce two total fluxes, one each for AdK and CK. The total flux through CK is the sum of unidirectional reactions in the mitochondrial intermembrane space (IMS) and cytosol that proceed towards PCr, and the total AdK flux is the sum of unidirectional AdK reactions in the IMS and cytosol that proceed towards ADP. To perform the comparison between fluxes, in our analysis, the total flux is increased by simultaneously increasing the forward and reverse flux in one or both compartmental locations (Method 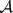), or by increasing the net flux through the shuttle (Method 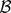). Table S1 provides the expressions used to increase total flux using both methods. It should be noted that Method 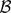 requires changing the net flux through one of the other parallel pathways (AdK shuttle, or direct ATP transport, or the CK shuttle.

**Figure 6:**
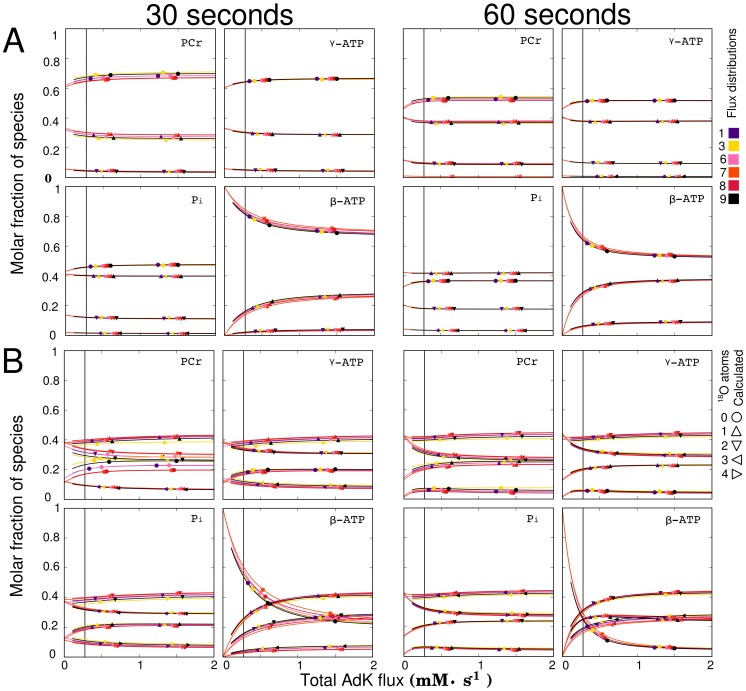
Influence of total AdK flux on the labeling state at 30 s and 60 s after a step change to 30% 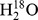 (A), and at 30 s and 60 s after a step change to 100% 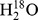 (B). Different flux distributions (see Table 1) were used to analyze the sensitivity of the labeling state to total AdK flux variation. All subplots indicate that the labeling state of 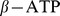 is the most sensitive indicator of total AdK flux. The vertical black line indicates the total AdK flux found in [20] for normoxic rat hearts. Comparing the slopes of the curves in (A) and (B), we see that labeling with 100% 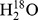 enhances the sensitivity of the dynamic 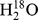 labeling method. Line colors indicate the flux distribution, while the symbols indicate the number of 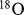 atoms.

Excluding Figure 6, where the sensitivity to total CK flux was analyzed, all flux distributions chosen use a total CK flux of 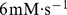 taken from [12] (except flux distributions 3 and 5 which have unidirectional CK fluxes). We take this as the maximum CK flux. This flux lies at the lower range determined in other 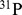-NMR saturation transfer studies [30]–[38]. Aksentijevi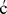 et al. found that the activity of CK is three times higher than AdK [47], so we took the maximum AdK flux to be 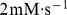, represented in flux distribution 1. Pucar et al. reported an AdK flux equivalent to 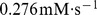 [20], and a lower value of 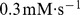 was chosen for all other flux distributions (except 4 and 5 which have unidirectional AdK fluxes).

Figure 6 shows the influence of changing the total AdK flux using Method 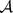 while keeping all other fluxes in the flux distribution constant (see Table 1). In this and following figures, some flux distributions overlap others over the range of the plot (flux ranges provided in Table S1), i.e. in Figure 6, flux distribution 2 is not plotted since it is a subset of flux distribution 1. All solutions for 30% 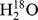 are seen to give similar labeling distributions as the AdK flux is changed. Note that regardless of the differences in the net flux between these flux distributions, the labeling state depends mainly on total AdK flux. After a step change to 30% 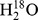 the labeling states of PCr, 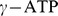, and Pi are only sensitive to total AdK fluxes below 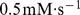. 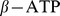 is seen to be sensitive up to 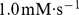 at 30s, reducing to 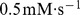 at longer sampling times, as evidenced by the variation of 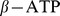 labeling induced by changes in total AdK flux (see Figure S2). Labeling with 100% 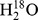 is seen to extend the range of sensitivity to total AdK flux for all species. The use of 100% 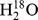 increases the rate of label incorporation into the phosphotransfer network without changing the ATP synthase or ATPase rates. This increases the ratio of the rate of label uptake to the rate of phosphotransfer reactions.

Because the metabolic system given in Figure 1 is compartmentalized, it is helpful to explore if the compartmental location of the AdK flux has an influence on the labeling state. Figure S3 shows the influence of changing the compartmental location of the AdK flux while keeping both the total AdK flux and all other fluxes in the flux distribution constant. Using 30% 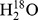, the compartmental location of the AdK flux is not seen to influence the labeling state, however, labeling with 100% 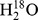 shows that the labeling state is somewhat sensitive to the compartmental location of the AdK flux, with the largest sensitivity observed in the labeling state of 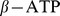.

**Figure 7:**
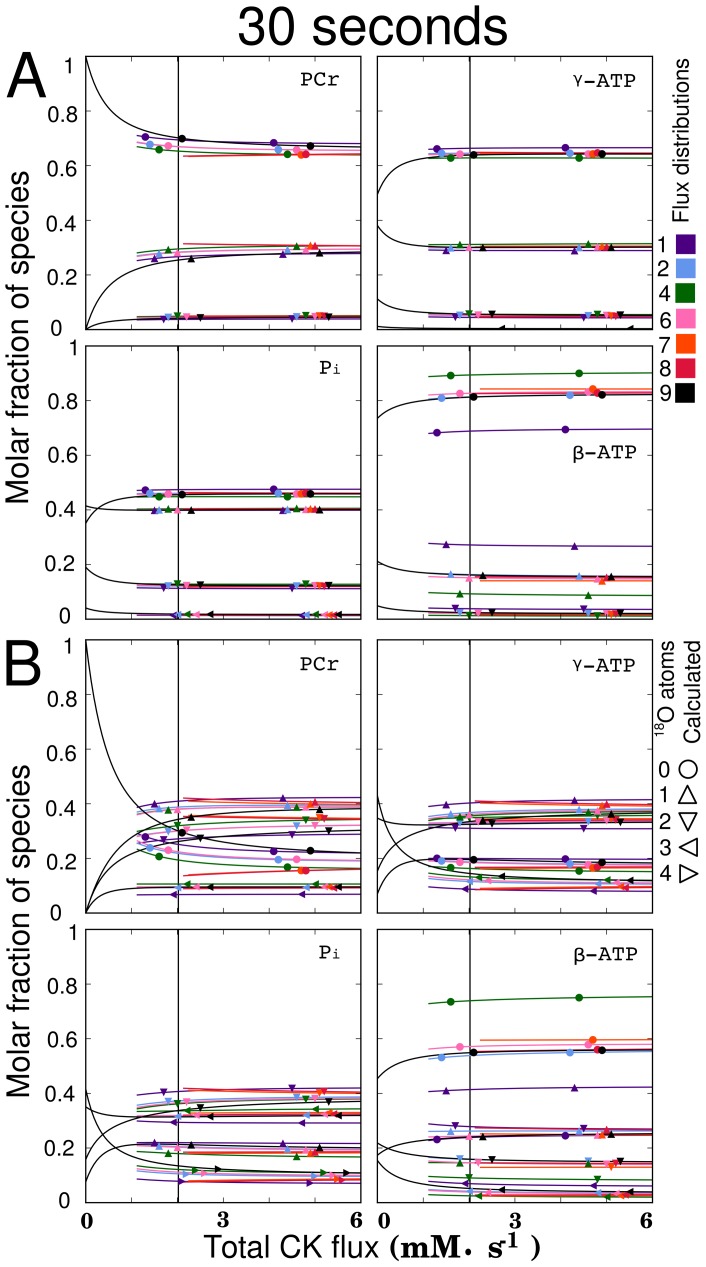
Influence of total CK flux on the labeling state at 30 s after a step change to 30% 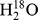 (A), and 100% 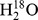 (B). Different flux distributions (see Table 1) were used to analyze the sensitivity of the labeling state to total CK flux variation. Both subplots indicate that the labeling state of all species is insensitive to the total CK flux above 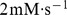 (vertical black line). This insensitive range includes values found in [20] and in [12] where total CK flux was found to be around 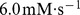. Analogous plots at 10s provided in Figure S4 show that it may be possible to gain information about the total CK flux in experiments shorter than 10s using 100% 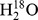, although it would be technically challenging to perform such an experiment. Line colors indicate the flux distribution, while the symbols indicate the number of 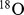 atoms.

To explore how the two coupled CK fluxes influence the labeling state, a plot (Figure 7) of total CK flux versus species labeling was produced using Method 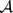 while keeping the other fluxes in the system constant (see Table S1). The vertical black line shows the CK flux that was calculated by Pucar et. al based on observations of the labeling state [20] (see Methods). While the flux found in [20] was expected to be *net* flux through CK shuttle, *total* flux through the CK reaction should be at least as large as the one estimated in [20]. This plot clearly shows an insensitivity of the labeling state to total CK flux above 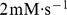. This means, for example, that the 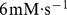 total CK flux determined in [12] provides almost the same labeling state of 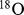 species as that provided by 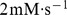. This demonstrates that the dynamic 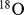 method is not sensitive enough to distinguish between any CK flux above 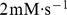. The use of 100% 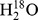 is seen to slightly increase the range of sensitivity (see Figure S4). Note that the differences between solutions are due to differences in total AdK flux.

**Figure 8:**
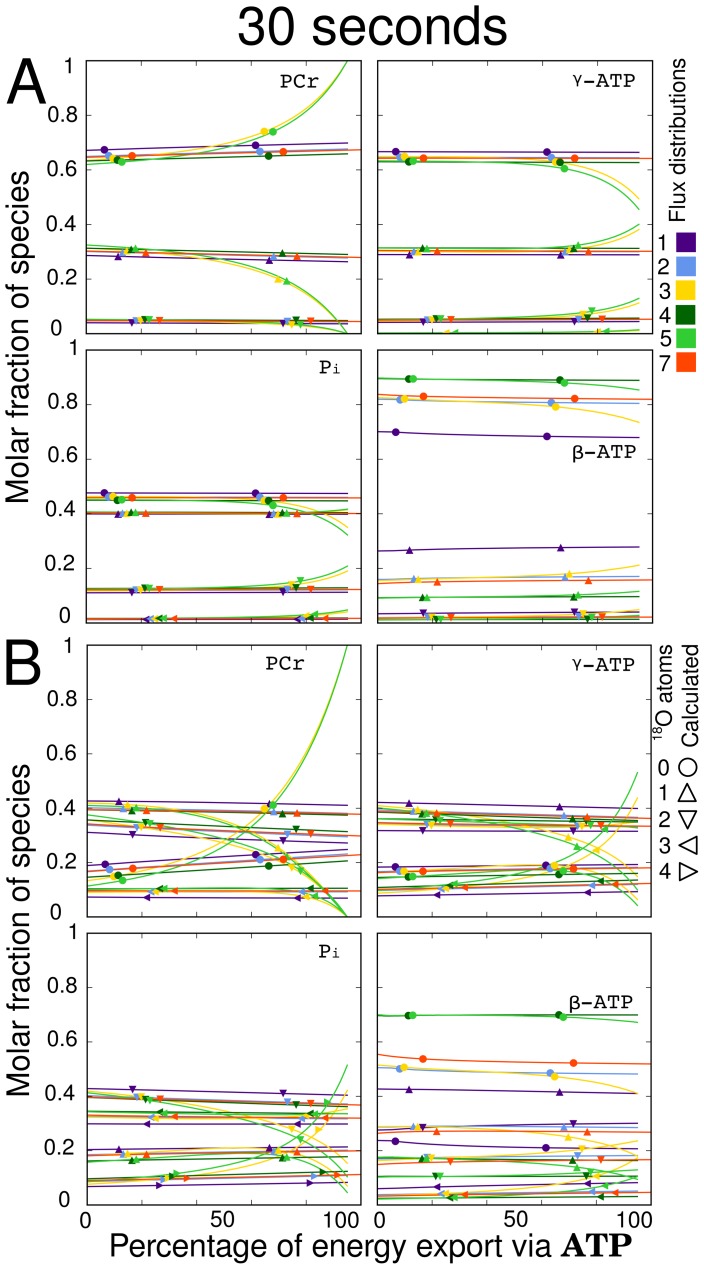
Change in metabolic labeling state at 30s with a transition from the maximum possible CK shuttle export ratio to the maximum possible ATP export ratio after a step change to 30% 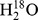 (A) and 100% 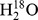 (B). Flux distributions 1, 2, 4, and 7 have constant total and net AdK flux as well as total CK flux over the range of the plot. Flux distributions 3 and 5 have unidirectional CK flux, and thus the total CK flux varies over the range of the plot. The sensitivity displayed by these two flux distributions results from the change in total CK flux and not CK export ratio. Looking at the other four flux distributions, subplot (A) shows that the export of energy via direct ATP export or the CK shuttle has a minor influence on the labeling state. Subplot (B) shows that the change in export ratio provides a small change in labeling state but cannot be considered a sensitive parameter. Line colors indicate the flux distribution, while the symbols indicate the number of 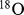 atoms.

Keeping total and net AdK flux constant as well as total CK flux constant, the percentage of energy exported via direct ATP transfer was varied from zero (maximum net flux through the CK shuttle) to its maximum possible value (zero net flux through the CK shuttle). The labeling states of flux distributions 1, 2, 4, and 7 were plotted during this change in Figure 8. For illustration, two unidirectional CK flux distributions (3 and 5) were included in the plot and are discussed below separately. Figure 8A (30% 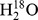) shows that the labeling state in the four flux distributions with reversible CK does not change appreciably as the flux of energy export is shifted from predominantly CK mediated to direct ATP transport, while Figure 8B (100% 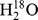) shows a moderate shift in labeling state. Figure S5 provides additional time points for Figure 8. Looking at these we see that in practice, this moderate shift cannot be used to determine which parallel pathway carries the most flux. This is also true because a number of less characterized parameters of this model such as the reversibility of ATP synthase to oxygen exchange and the sizes of metabolic pools have comparable effects on the labeling state (see Figure 4). Considering bidirectional flux distributions 1, 2, 4, and 7, in Figures 7 and 8, we see that the labeling states for each flux distribution are almost identical. This demonstrates that the labeling state is predominantly determined by total CK flux and not net CK flux. This property prevents the labeling state from explicitly defining the net flux through the CK shuttle. Similar conclusions can be reached from the analysis of energy export via AdK shuttle, as demonstrated in Figure S6.

For illustration, two additional flux distributions (3 and 5) with unidirectional CK fluxes are plotted on Figure 8. In this case, it is not possible to keep the total CK flux constant over the range of the plot, so the range of Figure 8 can be interpreted as a change in total CK flux produced using Method 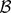. For these two flux distributions, the labeling state approaches that of the other four flux distributions while moving from lower to higher percentages of energy export via CK (lower to higher total CK flux). This shows that total CK fluxes above 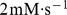 produce the same labeling state regardless of the percentage of energy export.

**Figure 9:**
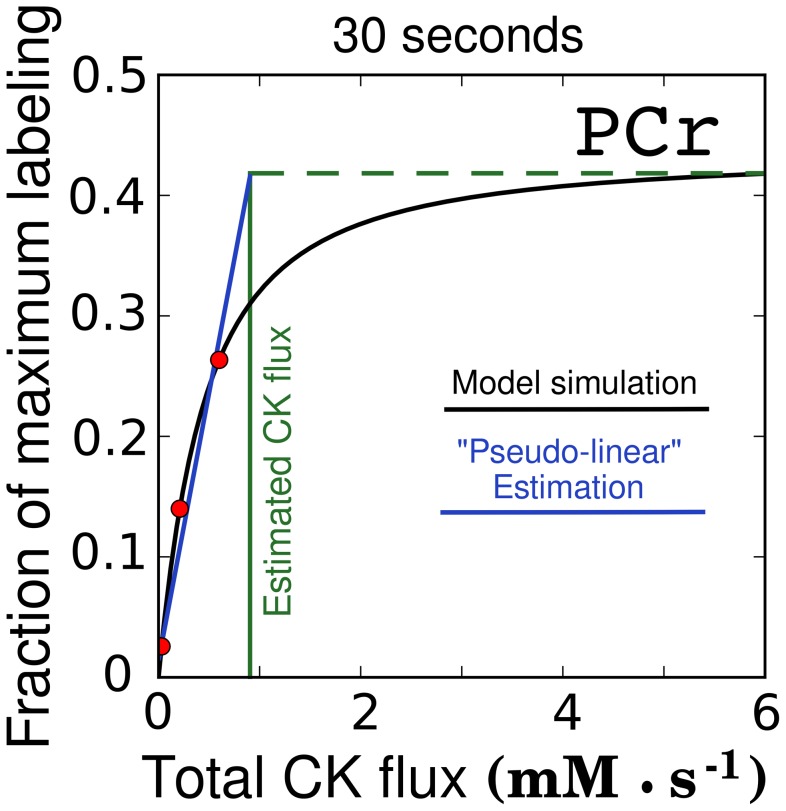
Pseudo-linear estimation of CK flux in rat heart on the basis PCr labeling at 30s. The total PCr oxygen labeling at 30s after a step change to 30% 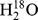 is found by combining the labeling of the three labeled PCr species in Figure 7 (flux distribution 9), using the equation for total labeling used by Dzeja et al. [20]. In [16], to estimate CK flux, CK activity was inhibited and corresponding total PCr labeling was found. Assuming that the total CK flux equals 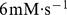, the red dots show the fraction of inhibition used in [16] to calculate the CK flux. As demonstrated in the plot, a linear approximation of PCr labeling-CK flux relationship (blue line, least squares fit), leads to the underestimation of total CK flux. In this example, instead of 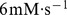 (PCr labeling taken for that CK flux), the CK flux found by the pseudo-linear estimation is 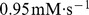, close to the flux reported in [20]. A horizontal dashed line corresponding to the labeling state at 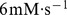 flux and a vertical green line at CK flux found by pseudo-linear approximation are provided as visual aids for clarity. The above geometry shows that the pseudo-linear method underestimates total CK flux.

To illustrate the properties of the pseudo-linear approximation method, used to determine the CK flux in [16] and more recently in [27], we used the same pseudo-linear approximation on predictions provided by our model. This approach is based on establishing the relationship between PCr labeling and CK flux through inhibition of CK activity by DNFB. For illustration, we used flux distribution 9, plotted in Figure 7, that provides a model prediction of the labeling state of all labeled species of CK as the total CK flux is gradually inhibited. Figure 9 applies the pseudo-linear approximation method to the total labeling curve found by combining the single, double, and triply labeled species of this model prediction. As Figure 9 demonstrates, the pseudo-linear approximation method underestimates total CK flux.

## Discussion

The main result of this work is that measuring the dynamic incorporation of 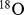 into the high-energy phosphotransfer network in heart does not allow for the unambiguous determination of net energetic fluxes larger than the ATP synthase rate when the bidirectionality of fluxes is taken into account. A number of observations from our sensitivity analysis lead to this statement: (I) the same 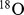 labeling states can be produced using many different flux distributions, (II) the labeling state is observed to be considerably more sensitive to total flux than to net flux, (III) in both the CK and AdK shuttles, the labeling state is sensitive only within the lower ranges of total flux, and (IV) the shift from 0 to 95% flux through direct ATP transfer results in indistinguishable labeling states when total CK flux is kept constant.

### Use of dynamic 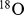 labeling to study flux distributions

Looking at the sensitivity plots in Figures 4 and 5 we see that a wide range of model parameters provide very similar predictions of the 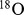 labeling state (both in magnitude and structure). Changing different parameters simultaneously is likely to result in a very similar prediction of the labeling state. This identifies that in the range of physiologically relevant cardiac performance and shuttle activity, many parameters in this model are structurally unidentifiable — including net flux (observation (I).

Observation (II) does not support the suggestion that the 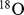 labeling method leads to estimation of *net* flux through phosphotransfer systems, as proposed in [20], [24]. According to our simulations, total flux through CK and AdK reactions have a major role in determining the labeling state of metabolites (Figures 3 and 6). Thus, our simulation results suggest that the fluxes estimated using the 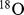 labeling method in [20] are *total* fluxes and can be directly compared to the fluxes estimated using 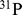-NMR saturation studies.

Observation (III) limits the use of the 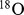 labeling method to study fluxes that are smaller than the ATP synthase rate, echoing a statement made by Dawis et al. [43]. The 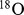 labeling method may be used to measure the flux of reactions that proceed at a rate slower than the ATP synthase rate such as reactions involving the 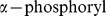 groups of ATP or ADP [41], [42].

With regards to observation (IV), we note that the use of 100% 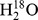 increases the sensitivity of the method, although this is not enough to find the proportion of energy exported via direct ATP transfer and the CK shuttle. Looking at Figure S5B, we see that a ten second labeling experiment using 100% 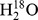 would result in a mass isotopologue distribution that could allow one to determine this split. This short duration experiment would present technical challenges, and the mixing rate of 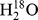 would become a critical component of the model, perhaps requiring the use of an organ level model taking into account heterogeneity within the heart.

Taken together, these observations lead to the conclusion that labeling with 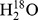 does not provide sufficient sensitivity to study the large fluxes, such as expected for the CK shuttle, under the conditions simulated herein. However, there are a number of ways that the sensitivity of the 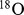 method can be improved, for example, by: (I) increasing the rate input of 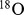 into the phosphotransfer network through the use of 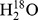 with a larger enrichment, (II) performing experiments at higher cardiac performance, and (III) using shorter sampling times. Figure S6 shows that the labeling state is more sensitive to changes in AdK flux using 100% 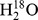 and labeling states predicted for the different flux distributions show a larger variation at shorter time points.

### Model implementation and analysis

The integrated kinetic model presented in this work was constructed to account for the most rapid isotope transformations that occur in the high-energy phosphotransfer reaction network in heart. As a first step in our analysis, a number of tests were conducted to determine if this model is suitable for the analysis of published dynamic 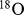 labeling data. Figure 2 shows that both steady state and dynamic labeling state predictions provided by our model match the predictions from the model developed by Dawis et al. [43]. Thus, we are able to reproduce earlier published studies with our implementation of the model. The model presented here is considerably more complex because it considers the bidirectionality of reactions as well as compartmentalized metabolic pools. It should be noted, however, that this added complexity is a minimum requirement to separate out the effects of net versus total flux. Following this, we demonstrate that the model solution is consistent with published dynamic traces of 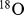 labeling (see Figure 3). Intriguingly, in Figure 3, we used a flux distribution determined by 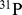-NMR inversion and saturation transfer [12] to calculate the labeling of metabolites in rat heart atrial tissue, and found that a wide range of flux distributions are able to reproduce these measured labeling states.

A number of simplifying assumptions were made to construct the model we present in this work. It is explicitly assumed that all metabolic fluxes proceed at a steady metabolic rate. In addition, we do not employ enzyme kinetics in the flux simulations, although this is not seen as a trade off because the resulting model has fewer parameters and many of the enzymatic kinetic parameters are not well characterized. A number of phosphotransfer fluxes were excluded from the model. However, the dynamic 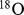 labeling method may be useful to determine total fluxes lower than the ATP synthase rate, so adding reactions with fluxes of lower magnitude may allow one to determine total flux in the additional phosphotransfer pathways. This extension of the model could be used to study how these total fluxes, which include the AdK shuttle flux, are altered under diseased conditions. It is expected that adding additional phosphotransfer reactions into the model will result in a dampening of the model dynamics, including the 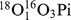 dynamics observed in Figure 3.

Unfortunately, the original 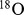 labeling dynamics used for flux estimations in [20] have not been published. While early dynamic 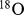 labeling studies include original labeling dynamics of individual species [41]–[43], only sums of species are reported in later studies [15]–[17], [25], [44], [45], [48]. For the heart, most of the reports include only derived data in the form of flux estimates [19]–[21], [23], [24], [26], [49]. Two heart studies include labeling dynamics reported as a sum of species in mouse and rabbit [18], [27]. A recent study reports labeling dynamics in atrial tissues taken from surgically excised male rat hearts [28]. However, no fluxes are reported in [28], thus we cannot compare the flux distributions derived from 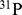-NMR inversion and saturation transfer [12] with 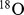 labeling dynamics.

We produced these modeling results using a step change from 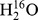 to a percentage of 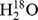 in the surrounding water. This change provides the most sensitive change in labeling state. In practice, however, the switch between 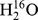 and 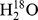 will be slower and will reduce the sensitivity of the method relative to our predictions.

As an alternative approach to the analysis presented in this work, one could compose hypothetical data sets and find the confidence intervals of model parameters. This would give an estimate of the sensitivity of the labeling method. Regardless of the approach used, we expect the conclusions to be the same. The approach used in this work was tailored towards comparison of different flux distributions to see whether the different energy transfer mechanisms proposed in the literature can be distinguished on the basis of 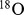 labeling data.

### Physiological implications

Without going through the published data presented in all dynamic 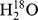 studies [15]–[27], [44], [45], it is sufficient to state that interpretation of dynamic 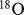 labeling data requires one to consider the size of metabolic pools and the bidirectionality of metabolic reactions. When comparing the total flux determined using 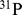-NMR inversion and saturation transfer analysis (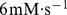) [12] with the net flux determined by 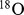 labeling analysis in control conditions for hearts before exposure to ischemia-reperfusion (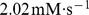) [20], we see from Figure 6 that the same labeling state is predicted with both fluxes. Keeping in mind that the pseudo-linear approximation method may underestimate the total CK flux (see Figure 9), the underlying flux distribution in the corresponding experiments could have been the same, regardless of the fluxes predicted in the control cases in [12] and [20]. However, we should stress that the flux distributions in [12] and [20] could be different due to differences in substrates used in those studies.

Importantly, our modeling results resolve all known discrepancies between the results of the dynamic 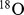 labeling method, and 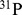-NMR inversion and saturation transfer. No fundamental difference was found in the nature of the fluxes being measured (net versus total), and indistinguishable labeling states were predicted using fluxes with different magnitudes. Because our model is non-discriminatory with respect to CK fluxes, we suggest that interpretation of dynamic 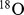 labeling data would result in flux predictions that are compatible with 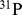-NMR inversion and saturation transfer results.

### Future directions

It has been shown that information regarding the compartmentation of metabolites and the bidirectional nature of metabolic fluxes is contained in the dynamic component of labeling data [39]. Combining a range of sampling points from short and long labeling experiments may increase the sensitivity of isotopologue modeling because many fewer flux distributions will have same labeling dynamics that match all measured data compared with only one sampling point. Finding plausible flux distributions from dynamic data sets requires the use of an integrative kinetic model. The model composed in this work included a system of 132 ordinary differential equations that were generated using a specialized program (see Text S1). While composition of such a model is not trivial, we find it an obligatory step for the analysis of labeling dynamics.

For the phosphotransfer network in the heart, sampling at an earlier time, in addition to 30 seconds, would enhance the sensitivity of the method. Adding an additional sampling point at a longer time during the approach to isotopic equilibrium would provide a better means to extract pool size information from the isotopic transient. However, the dynamic 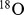 labeling method requires the sacrifice of multiple animals per time point, and adding additional sampling times will greatly increase both the ethical and monetary costs.

In [20], the performance of Langendorff perfused rat hearts was relatively low, as evidenced by the rate pressure product (RPP) equal to 
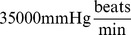
. For comparison, the performance range used to study energy transfer in [12] was, in terms of RPP, from 1700 to 
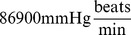
. While the highest RPP value in [12] corresponded to the case which should be considered as an extreme condition representing pathology and at the limit of the isovolumic perfused heart [12], [29]. However, no signs of pathological conditions at 63300 and 
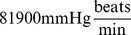
 were observed. These levels of cardiac performance are considerably larger than the level of cardiac performance used in [20] suggesting that higher cardiac performance levels are attainable using the same isovolumic preparation. This would increase the flux through ATPase reactions and improve the sensitivity of the dynamic 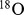 labeling method. In this work (Figure 5A), the upper range of simulated cardiac performance is 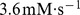, which roughly corresponds to 
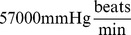
.

While our results show that dynamic 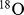 labeling data is unable to determine CK fluxes with a higher magnitude than the ATP synthase rate, or the split between the CK shuttle and direct ATP transfer in normoxic hearts, this method is sensitive to total AdK flux because total AdK flux is expected to be lower than the ATP synthase flux [47]. Our sensitivity analysis shows that changes in total AdK flux produce significant changes in the labeling state of 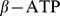. This opens up the possibility of combining the dynamic 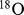 labeling approach with 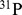-NMR inversion and saturation transfer. By using the same rigorous statistical testing of the model solutions against experimental data as in [12], with both types of data, it would be possible to combine the strengths of both methods with the promise of determining intracellular energy transfer flux distribution in the beating heart. It is for this reason that we view these two organ-level methods as complementary.

Our results widen the discussion that attempts to reveal the mechanisms that ensure the homeostasis of metabolites during cardiac function (or not) [50]. Understanding these mechanisms will require the use of integrative kinetic models that consider all possible operating modes of this metabolic network and all possible functional purposes of all enzymes, metabolites, and dynamic effects involved in the transfer of ATP from the IMS to the myofibrils and sarcoplasmic reticulum Ca^2+^ ATPase pumps.

## Methods

### Composition of metabolic system

The metabolic network in Figure 1 is the simplest possible model that is able to separate out the effects of net versus total flux in the CK and AdK shuttles. The reactions included in the network are a subset of the reactions known to transfer high-energy phosphoryl groups. Reactions catalyzed by the enzymes glyceraldehyde-3-phosphate dehydrogenase, 3-phosphoglycerate kinase, pyruvate kinase, hexokinase, succinyl-CoA synthase, and guanylate kinase have been excluded to simplify the system. The activities of the four glycolytic enzymes mentioned are equal to or less than the activity of AdK [47], [49]. With the exception of guanylate kinase, the net flux through all of these reactions is constrained by the stoichiometry of a larger metabolic network and is not expected to contribute appreciably to ATP regeneration. An estimate of glycolytic net flux is provided by 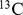 isotopologue studies which have calculated that the anaplerotic flux into the citric acid cycle derived from glycolysis is between 3 and 12% [51] of net citric acid cycle flux which varies between 4 and 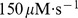 in normoxic heart [52]. Because the citric acid cycle flux is much smaller than the ATP synthase flux considered here (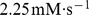), and is constrained by the stoichiometry of the entire metabolic network, we have excluded these reactions from the analysis. However, the reversibility of these fluxes coupled to large pools could facilitate temporary regeneration of the ATP pool in the failing heart, a phenomena recently observed by Aksentijević et al. [47]. Their reversibility could slow down the dynamics of 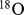 incorporation somewhat by increasing the size of the pool of metabolites that become labeled. This phenomenon is analogous to adding a compartmentalized side pool which slows down the labeling transient as described in [39]. Because this study is concerned with only the net fluxes in normoxic heart we need not consider these reactions here.

### Uptake of 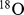 from 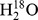


Because the model developed in this work tracks the transient exchange of each oxygen atom in the phosphotransfer network with the surrounding water environment, it is necessary to know how each oxygen atom transfers during the course of each reaction. It is known that exchange of phosphate oxygens with those of water does not occur in glycolysis [53, and references therein] and requires ATPases. Only the ATPase and ATP synthase reactions are able to transfer 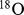 between 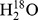 and any of the four oxygen atoms of inorganic phosphate. Pi is symmetric and enrichment in each oxygen position occurs at the same rate. The enrichment observed in the three oxygen atoms of 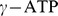 are also identical. Enzyme bound states for both ATPase and ATP synthase were included in the model because it is known that even under physiological conditions of oxidative phosphorylation multiple reversals of ATP formation occur before ATP is released to the media [54], and multiple reversals are known to occur during actomyosin catalysis [55]. No appreciable amount of positional oxygen exchange is observed between the 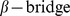 and 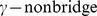 oxygens in ATP or the 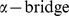 and 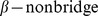 oxygens in ADP [56]. Taken together, these properties ensure that all three oxygen atoms in every phosphoryl group of all species have an equal probability of being isotopically labeled. The derivation of the model equations assumes this behavior.

### Derivation of model equations

The model was constructed by: (I) generating the full set of individual isotopic transformations, (II) combining these transformations into a mass balance around each isotopologue in the system, (III) composing mass isotopologue pool relations while taking into account oxygen atom mappings, and (IV) composing mass isotopologue balances by collecting the isotopologue balances according to the pool relations. The result is a system of 132 ordinary differential equations (ODEs). The intermediate equations for all of these steps are provided in supporting Text S1. The mass isotopologue equations contain variables for (I) the pool size of all 18 metabolic species and (II) the forward flux for all 20 reactions as well as the reverse flux for the 17 bidirectional reactions, giving a total of 37 unidirectional flux variables. A program was written in Python to generate these equations. This program implements symbolic manipulation tools specifically designed to carry out steps (I) through (IV), and is available as a Python module for generation of mass isotopologue equations in http://code.google.com/p/iocbio/wiki/IOCBioOxygenIsotopeEquationGenerator.

### Selection of model parameters

The 18 metabolic pools are assumed to be constant during the labeling processes. Pool size measurements for total ATP, PCr, and Pi were taken from Vendelin et al. for activation by Ca 1.8 mM [12]. The sizes of these pools are 7.55
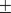
1.13 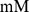, 16.4
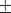
2.44 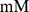, and 1.41
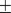
0.78 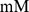, respectively. In [12], the pool of ADP was too small to measure, and was taken as 1% the ATP pool. The pools of these four species are split between the various compartments in the model. In the IMS, the fraction of the total pool of ATP, PCr, and ADP was taken to be 1% of the total amount of each metabolite. The fraction of the total pool of ATP, ADP, and Pi in the enzyme bound ATPase and ATP synthase states was taken to be 0.05% of the total amount of each metabolite. The fraction of ATP, ADP, and Pi in the mitochondrial matrix was taken to be 12.5% from measurements of the ATP pool [12]. The pool size of enzyme bound water was taken to be the same size as the enzyme bound ATP pool.

The 20 net flux variables in the model are constrained to metabolic steady state by three independent net flux variables. We chose these to be the net rate of ATP synthase, the net flux of ATP between the IMS and the cytosol, and the net flux of PCr between the IMS and the cytosol. A set of 17 relations between these and all other net flux variables was found using a method we recently developed [57]. The forward and reverse fluxes for the 17 bidirectional reactions were constructed by combining the net flux with an exchange flux, as described by Wiechert and Graaf [58].

### Conversion of fluxes

Figure 1B in the paper by Pucar et al. reports a CK flux of 330 
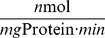
 [20]. All metabolite data are expressed in 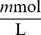 of intracellular water, assuming 2.72 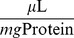 intracellular water to total protein content, as in Vendelin et al. [12], so the CK flux in [20] converts to 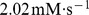. Likewise, Figure 2B in [20] reports an AdK flux of 45.6 
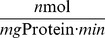
 which converts to 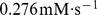.

The citric acid cycle flux is reported by Des Rosier et al. to be between 0.1 and 4 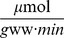 in [52]. As above, assuming 2.72 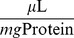, and 160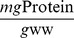 as in Vendelin et al. [12], these values convert to between 4 and 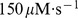.

### Numerical methods

To solve the system of ODEs we used a variable-coefficient ODE solver with the Backward Differentiation Formula method [59] provided by SciPy (http://www.scipy.org). Our system of ODEs was implemented in C for computational efficiency and exposed to Python using f2py [60] for efficient prototyping.

## Supporting Information

Figure S1Dynamic simulation of the 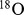 labeling state of the phosphotransfer network given in Figure 1 for the nine different sets of steady fluxes found in Table S1. Subplot (A) shows the simulations with a step change to 30% 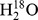 while subplot (B) shows the simulations with a step change to 100% 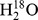. Flux distributions 4 and 5 (in green) deviate with respect to 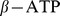 labeling state and are the only solutions with unidirectional AdK flux. Solution 1 has a larger AdK flux and also deviates with respect to 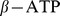 labeling. Greater differences between solutions and a more complex dynamic component is observed in the lower plot with 100% 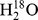 labeling. The vertical black line indicates the 30 s sampling point used in [20]. Colors represent flux distributions in Table S1, and symbols indicate the number of 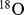 atoms attached to either Pi or the phosphoryl group of the species being plotted (indicated in the top right corner of each subplot).(PDF)

Figure S2Additional time slices for Figure 6. Influence of total AdK flux on the labeling state at 30 s and 60 s after a step change to 30% 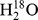 (A), and at 30 s and 60 s after a step change to 100% 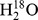 (B). The first two grey bars are the regions plotted in Figure S6. The third grey bar shows the high value of AdK flux estimated from the ratio of AdK and CK activity measurements made by Aksentijević et al. [47].(PDF)

Figure S3Influence of compartmental location of AdK flux on the labeling state at 30 s after a step change to 30% 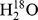 (A), and 100% 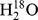 (B). All flux parameters are given in Table S1. When 100% 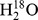 is used as the labeling agent, the labeling state is weakly sensitive to the compartmental location of AdK flux. Line color and symbol notation are identical to Figure S1.(PDF)

Figure S4Additional time slices for Figure 7. Influence of total CK flux on the labeling state at 30 s after a step change to 30% 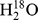 (A), and 100% 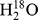 (B). The plots at 10 s show that it may be possible to gain information about the total CK flux in experiments shorter than 10 s when using 100% 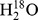, although, it would be technically challenging to perform such an experiment.(PDF)

Figure S5Additional time slices for Figure 8. Change in metabolic labeling state at 30 s with a transition from the maximum possible CK shuttle export ratio to the maximum possible ATP export ratio after a step change to 30% 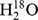 (A) and 100% 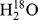 (B).(PDF)

Figure S6Change in labeling state with increasing energy export via AdK at 30 s after a step change to 30% 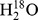 (A), and 100% 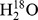 (B). All flux parameters are given in Table S1. The two ranges (0–0.112 and 0.188–0.3) correspond to the first two grey bars in Figure S2. The vertical grey line indicates the total AdK flux found in [20]. This line lies in a region where only the labeling state of 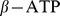 can be used to find total AdK flux. In contrast, using 100% 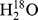, total AdK flux is weakly sensitive to the labeling state of the other species. Line color and symbol notation are identical to Figure S1.(PDF)

Table S1 Flux distributions used in Figures 6–8 and Figures S1, S2, S3, S4, S5, S6. The rows in this table summarize all flux distributions considered in this paper. These flux distributions were selected to range between physiologically feasible states. Figures S2, S3, S4, S5, S6 plot predictions of the labeling state as one flux parameter (shown in bold) is varied over the range of the plot. Relationships between all other flux parameters are provided. Flux distribution and figure(s), are denoted by FD and Fig., respectively. Subscripts x, f, and r refer to the fraction of net flux, forward, and reverse fluxes, respectively. Superscripts o, i, and t refer to cytosolic, IMS, and total fluxes, respectively.
(PDF)

Table S2 Parameter ranges used to construct Figure 4. In total, 36 fluxes are listed, however, these are derived from 18 exchange flux parameters. In addition, 18 pool size parameters are included.
(PDF)

Text S1 Derivation of the model equations with all intermediate steps.
(PDF)
