# Supplementary figures and images for: Sensitivity Analysis of Flux Determination in Heart by H2 18O -provided Labeling Using a Dynamic Isotopologue Model of Energy Transfer Pathways
Source: PLoS Comput Biol. 2012 Dec 6;8(12):e1002795. doi: 10.1371/journal.pcbi.1002795 (PMC3516558; doi:10.1371/journal.pcbi.1002795)

Figure S1 — Step to 30% (A) and 100% (B)  $\text{H}_2^{18}\text{O}$ .

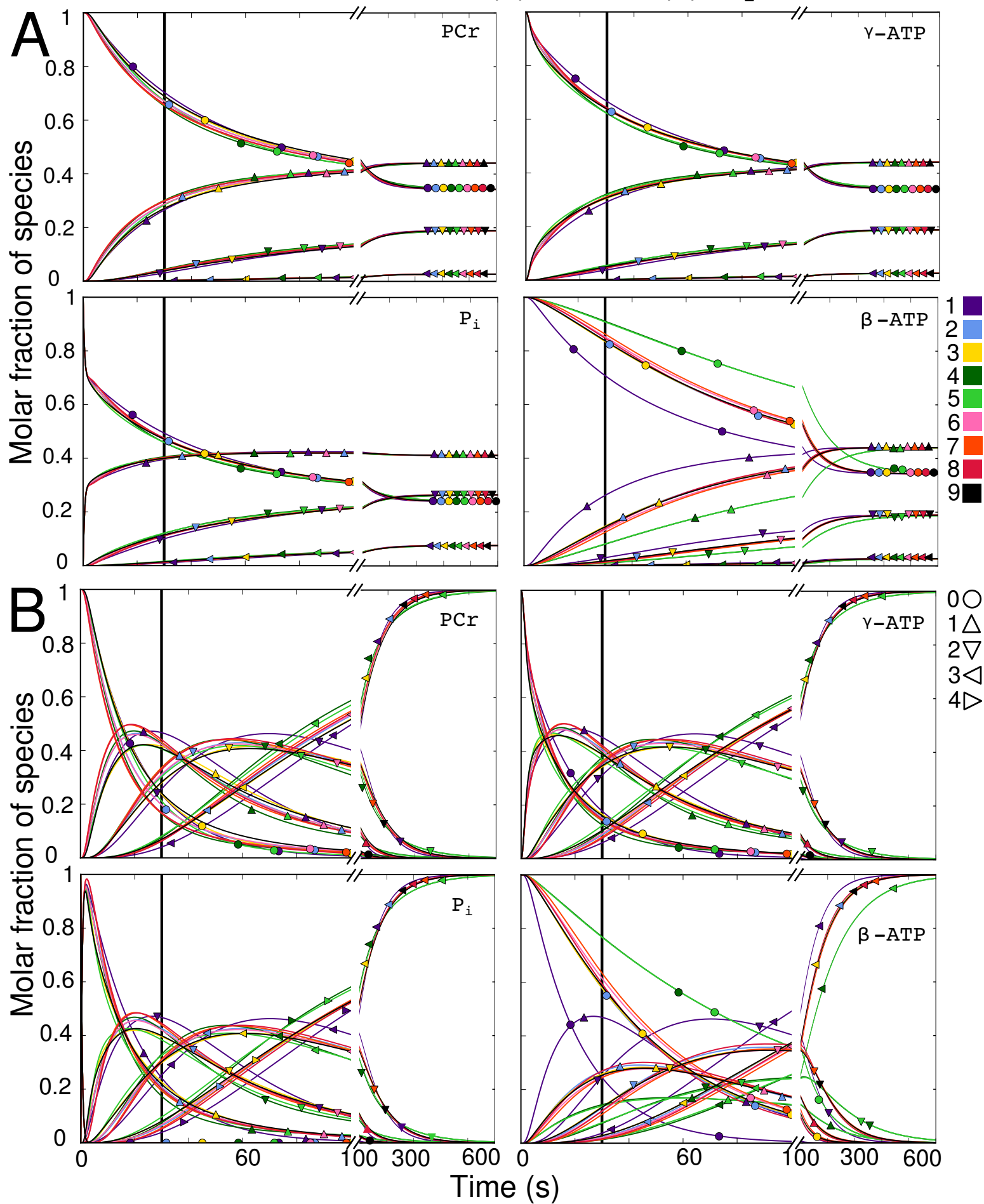

Supplement: Figure S1 — Dynamic simulation of the labeling state of the phosphotransfer network given in Figure 1 for the nine different sets of steady fluxes found in Table S1. Subplot (A) shows the simulations with a step change to 30% while subplot (B) shows the simulations with a step change to 100% . Flux distributions 4 and 5 (in green) deviate with respect to labeling state and are the only solutions with unidirectional AdK flux. Solution 1 has a larger AdK flux and also deviates with respect to labeling. Greater differences between solutions and a more complex dynamic component is observed in the lower plot with 100% labeling. The vertical black line indicates the 30 s sampling point used in [20]. Colors represent flux distributions in Table S1, and symbols indicate the number of atoms attached to either Pi or the phosphoryl group of the species being plotted (indicated in the top right corner of each subplot). (PDF) [file pcbi.1002795.s001.pdf]

A

Figure S2A — Step to 30%  $\text{H}_2^{18}\text{O}$ .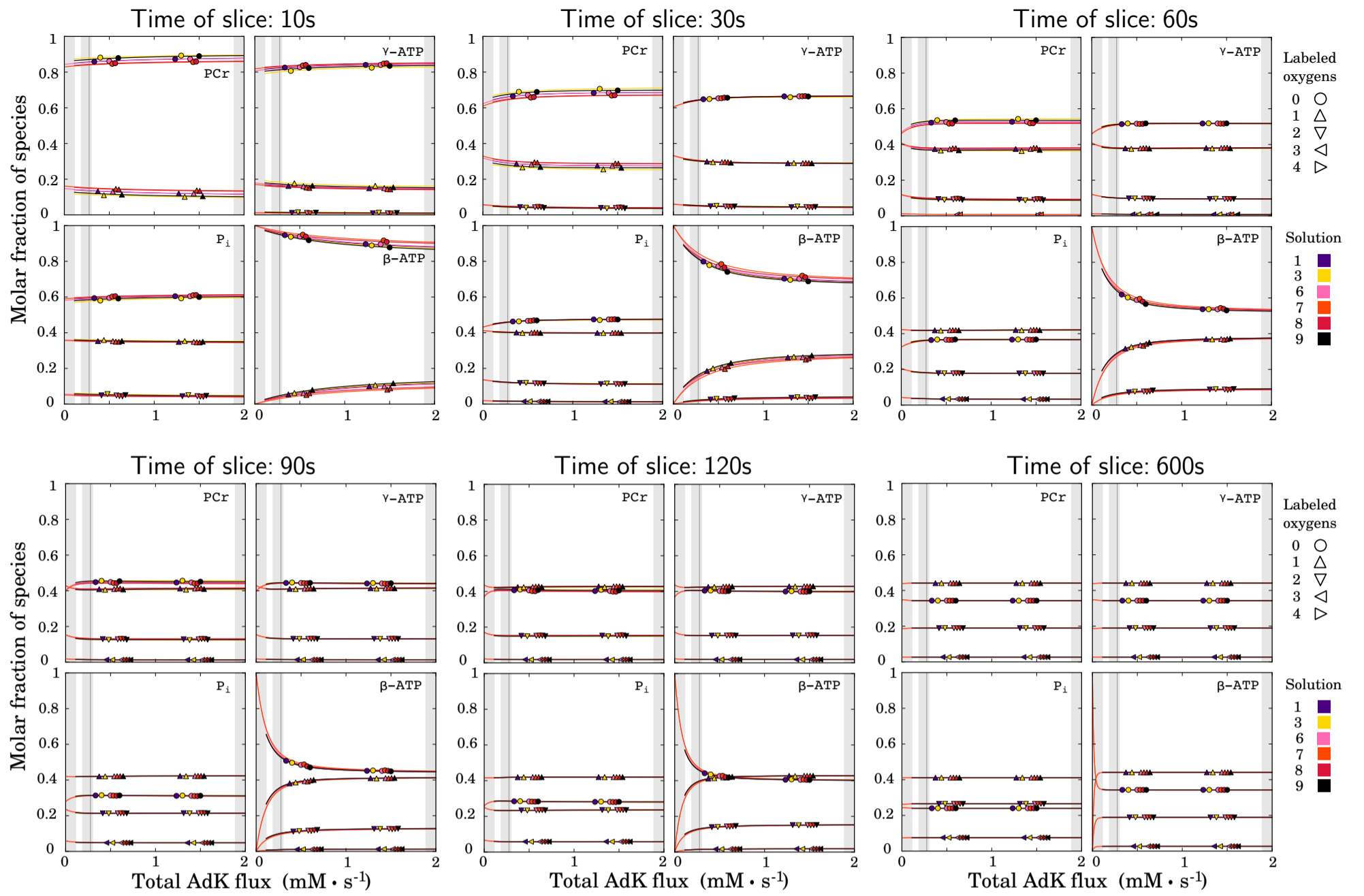

B

Figure S2B — Step to 100%  $\text{H}_2^{18}\text{O}$ .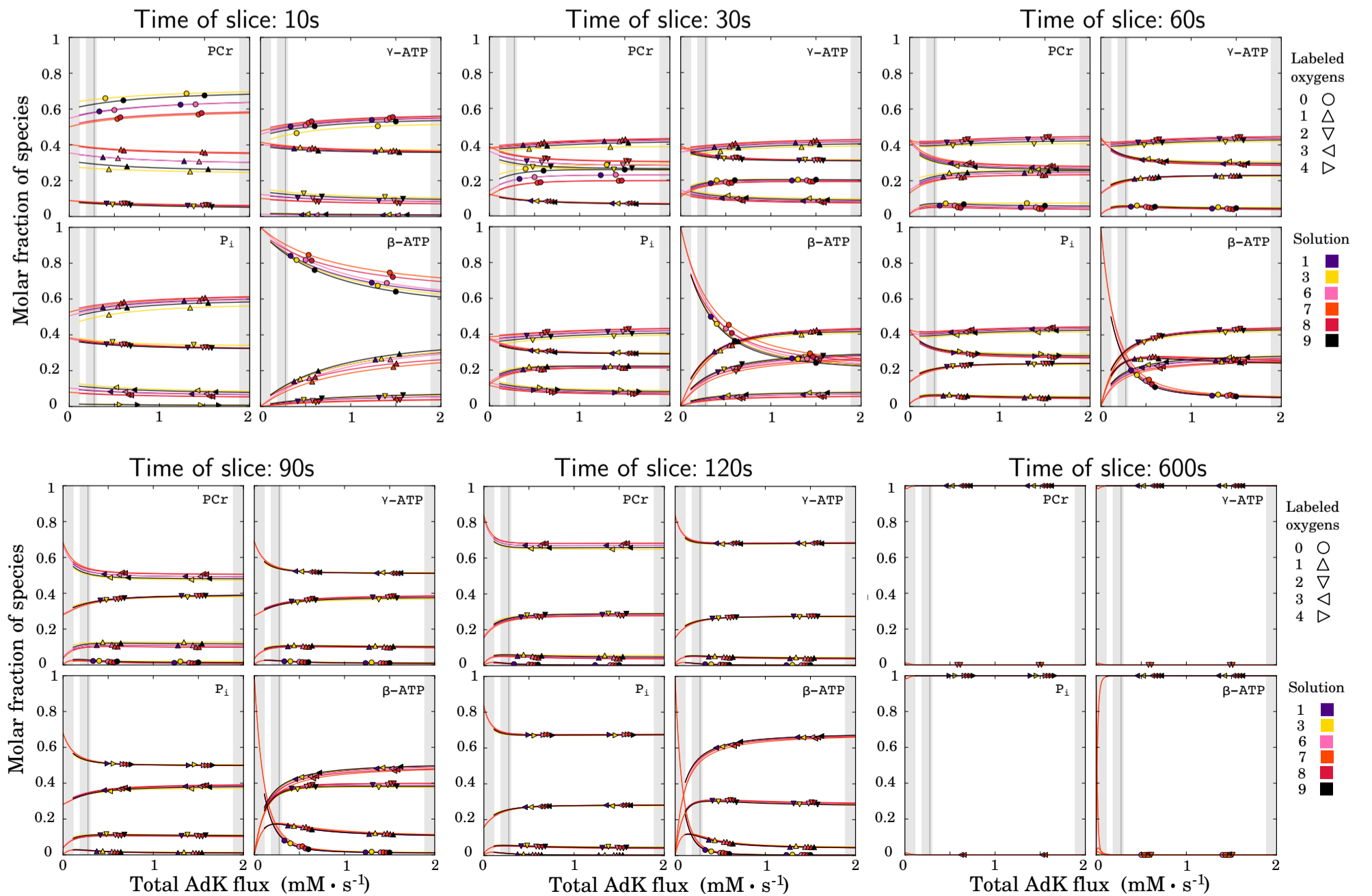

Supplement: Figure S2 — Additional time slices for Figure 6. Influence of total AdK flux on the labeling state at 30 s and 60 s after a step change to 30% (A), and at 30 s and 60 s after a step change to 100% (B). The first two grey bars are the regions plotted in Figure S6. The third grey bar shows the high value of AdK flux estimated from the ratio of AdK and CK activity measurements made by Aksentijević et al. [47]. (PDF) [file pcbi.1002795.s002.pdf]

A

Figure S3A — Step to 30%  $\text{H}_2^{18}\text{O}$ .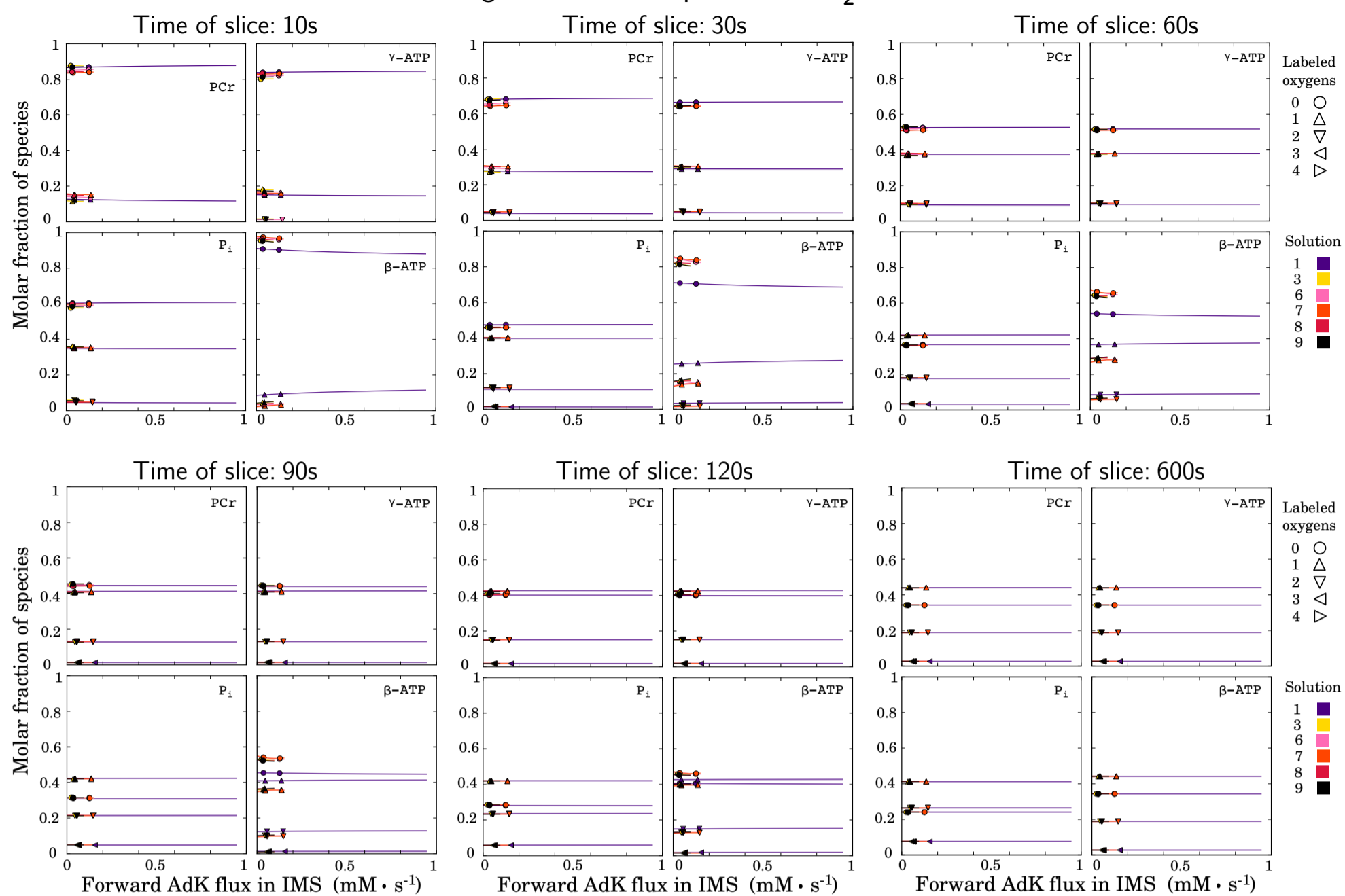

B

Figure S3B — Step to 100%  $\text{H}_2^{18}\text{O}$ .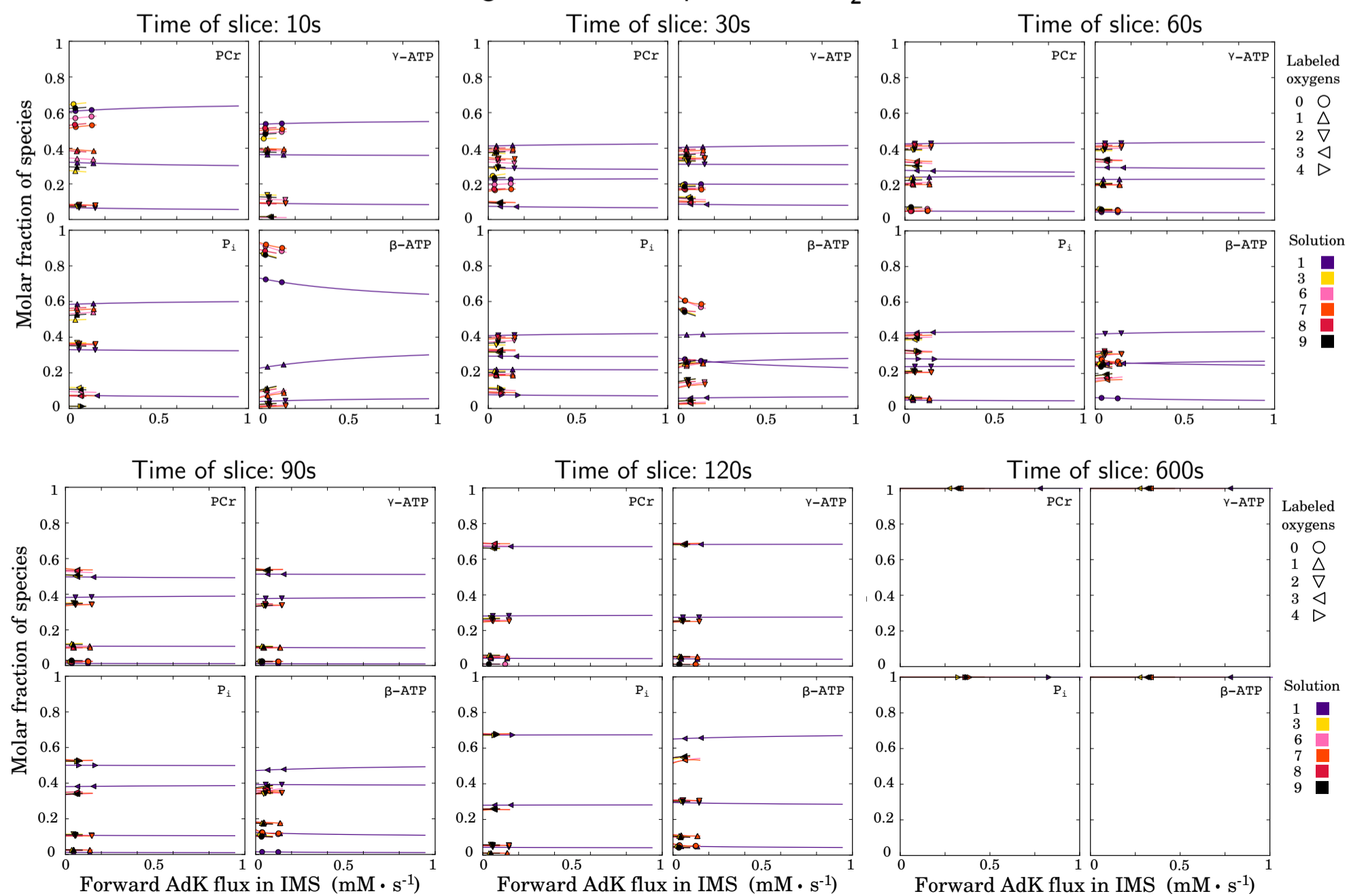

Supplement: Figure S3 — Influence of compartmental location of AdK flux on the labeling state at 30 s after a step change to 30% (A), and 100% (B). All flux parameters are given in Table S1. When 100% is used as the labeling agent, the labeling state is weakly sensitive to the compartmental location of AdK flux. Line color and symbol notation are identical to Figure S1. (PDF) [file pcbi.1002795.s003.pdf]

A

Figure S4A — Step to 30%  $\text{H}_2^{18}\text{O}$ .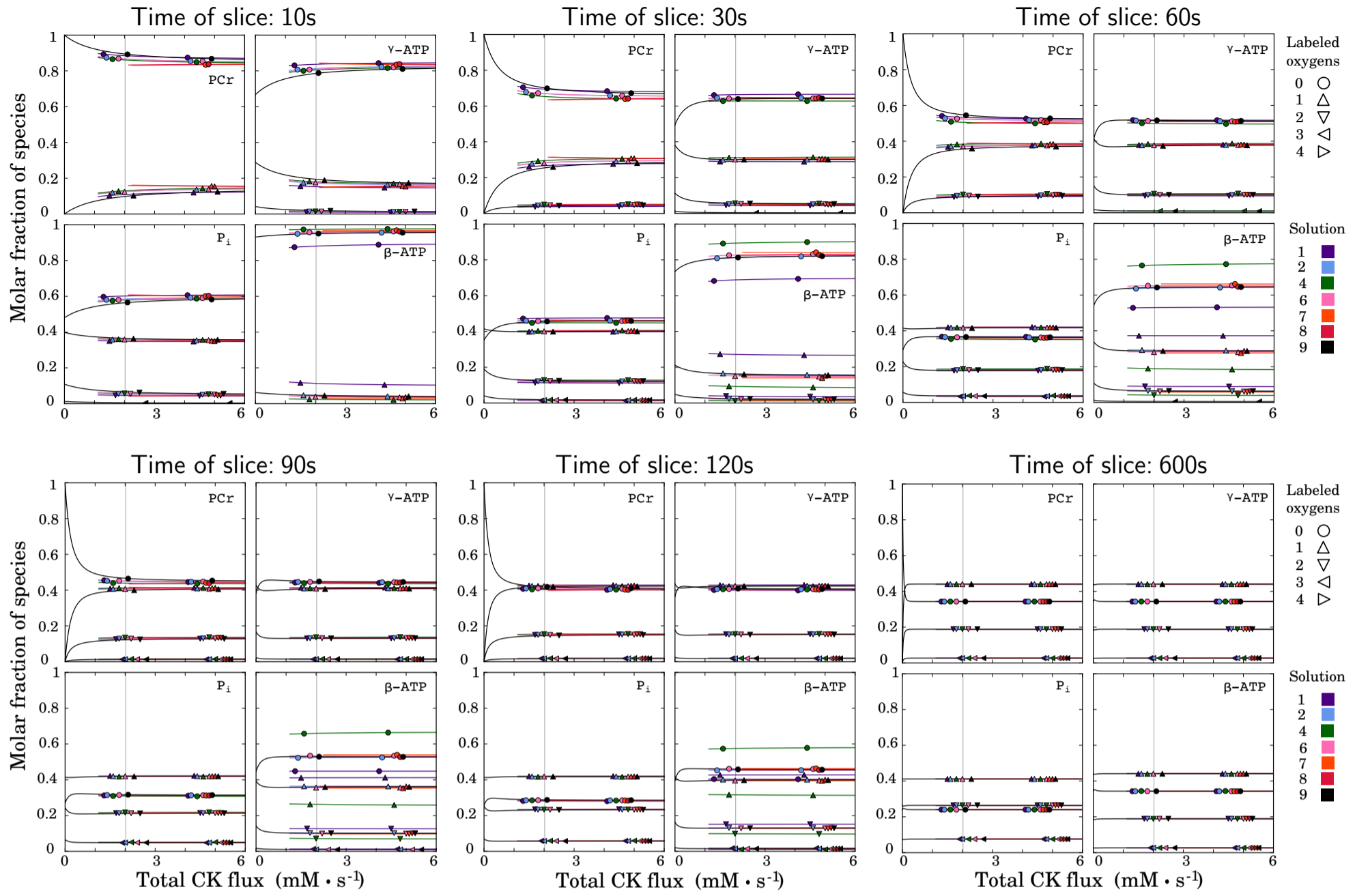

B

Figure S4B — Step to 100%  $\text{H}_2^{18}\text{O}$ .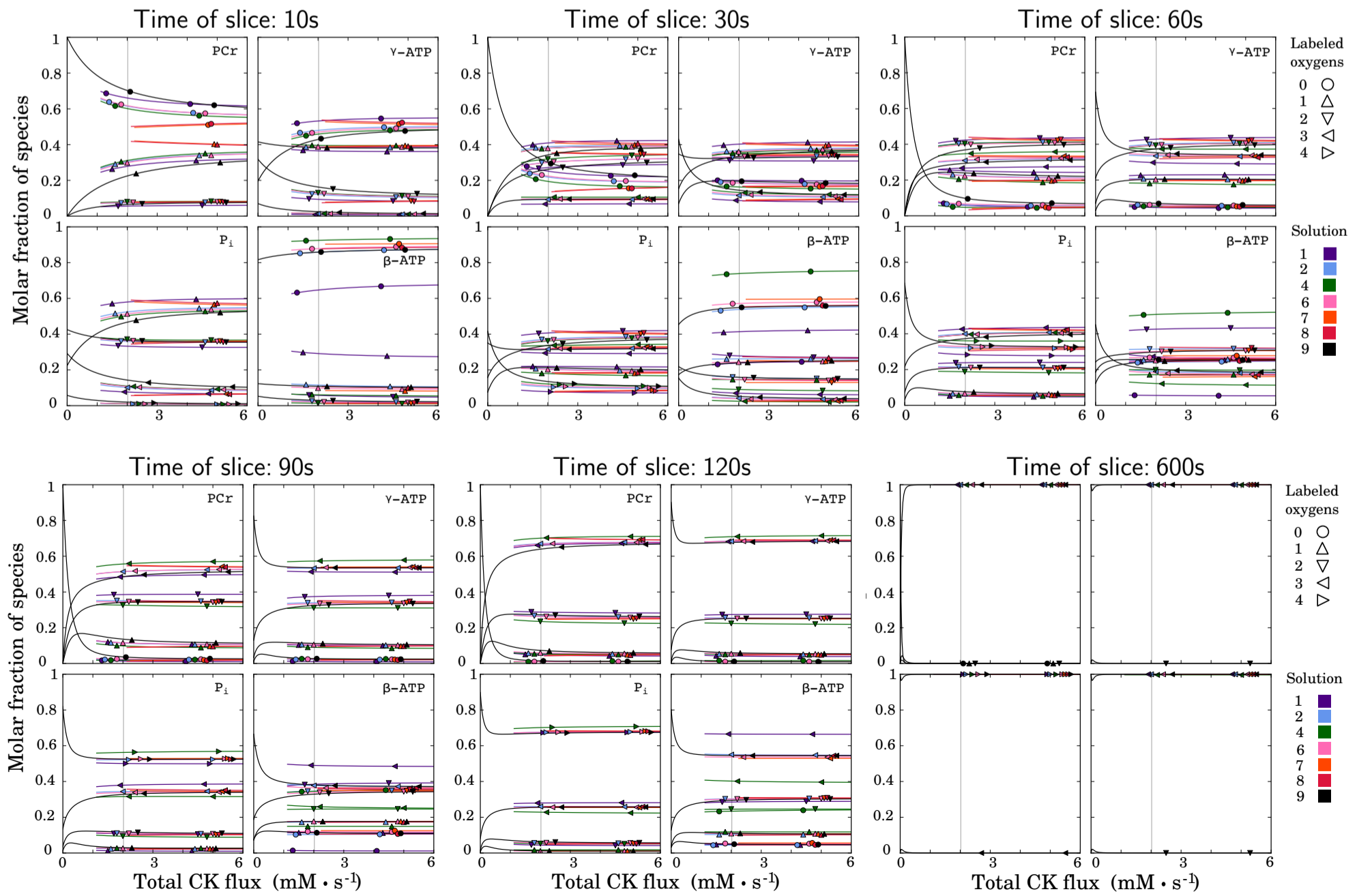

Supplement: Figure S4 — Additional time slices for Figure 7. Influence of total CK flux on the labeling state at 30 s after a step change to 30% (A), and 100% (B). The plots at 10 s show that it may be possible to gain information about the total CK flux in experiments shorter than 10 s when using 100% , although, it would be technically challenging to perform such an experiment. (PDF) [file pcbi.1002795.s004.pdf]

A

Figure S5A — Step to 30%  $\text{H}_2^{18}\text{O}$ .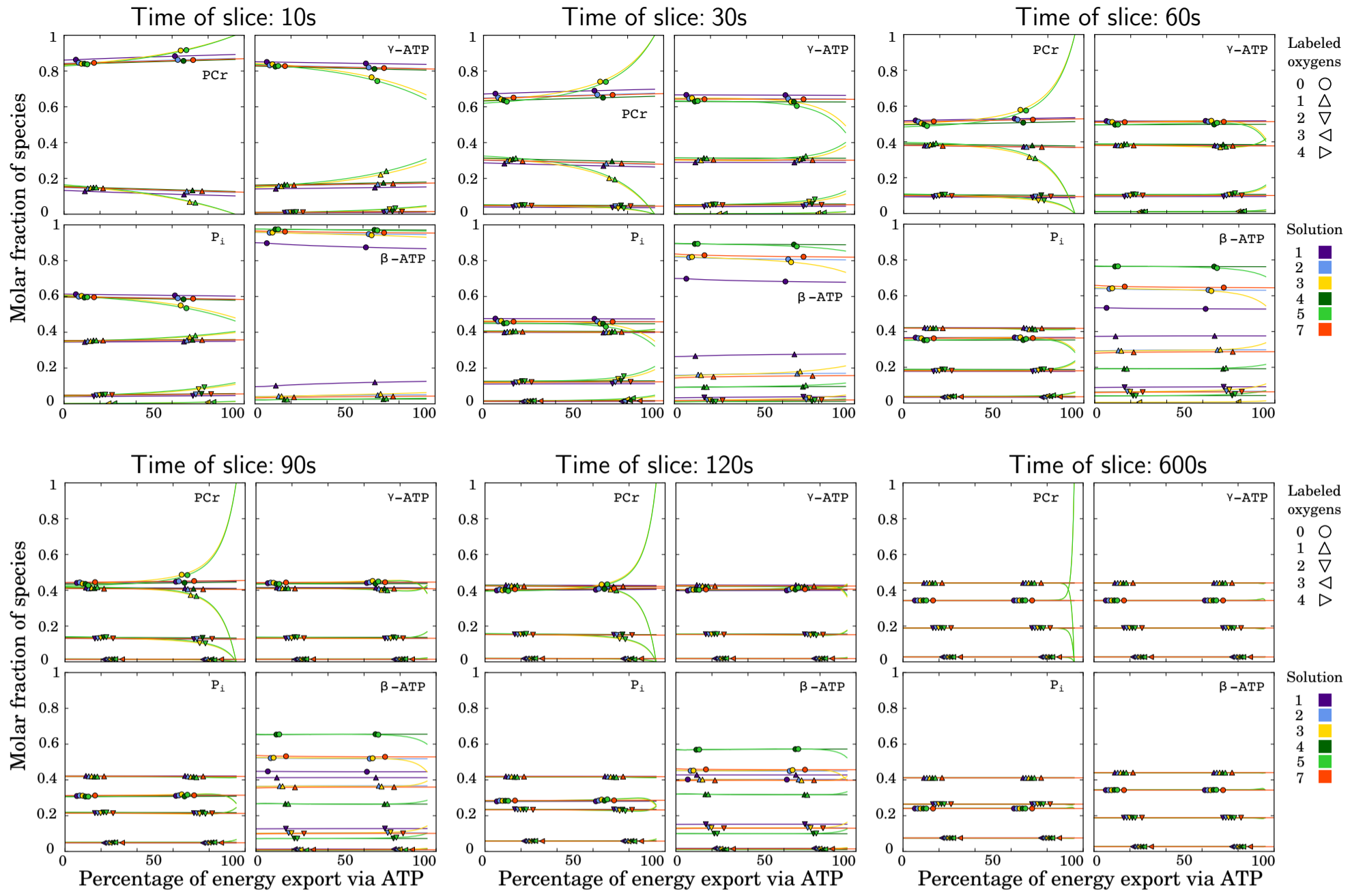

B

Figure S5B — Step to 100%  $\text{H}_2^{18}\text{O}$ .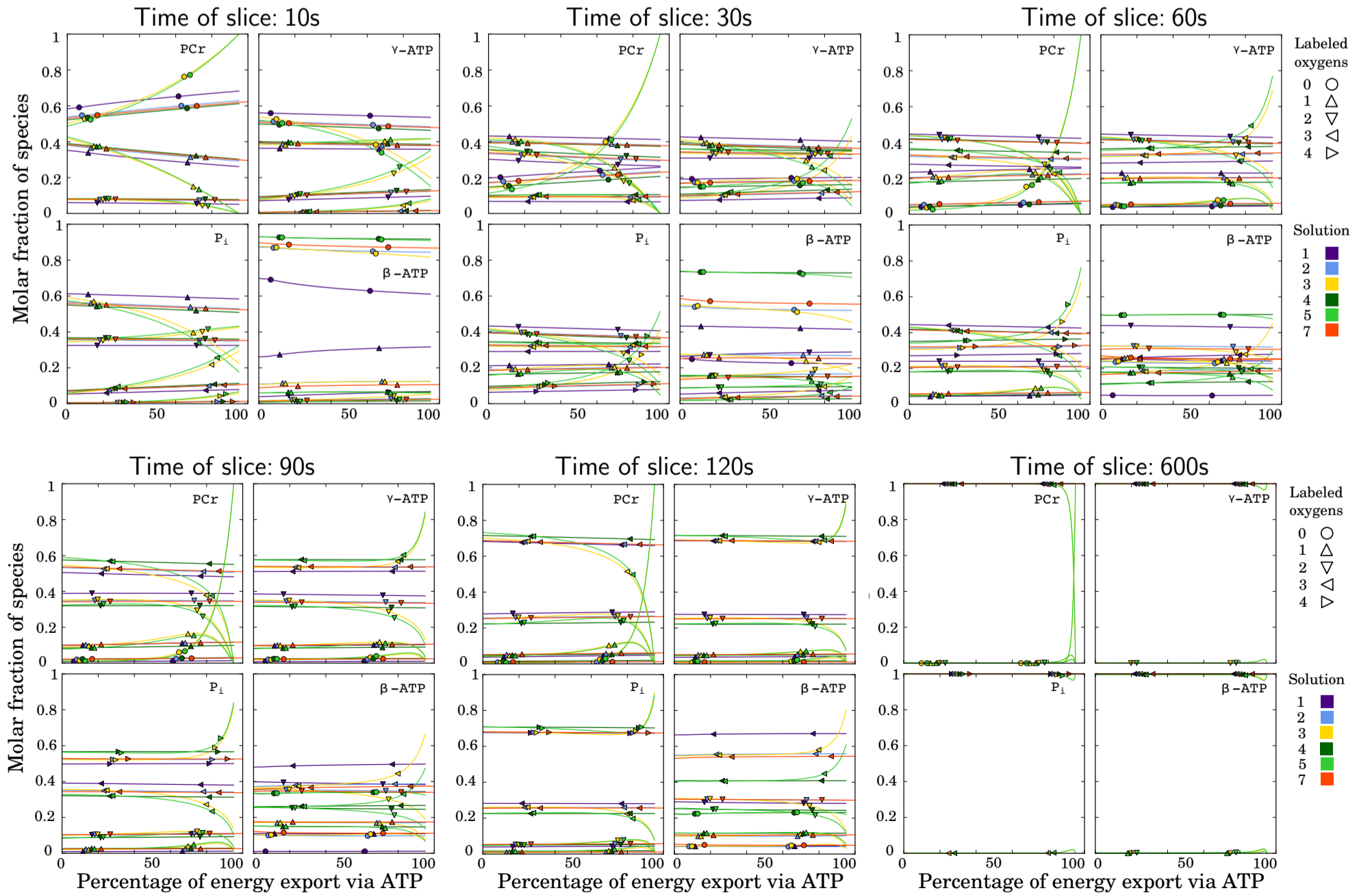

Supplement: Figure S5 — Additional time slices for Figure 8. Change in metabolic labeling state at 30 s with a transition from the maximum possible CK shuttle export ratio to the maximum possible ATP export ratio after a step change to 30% (A) and 100% (B). (PDF) [file pcbi.1002795.s005.pdf]

A

Figure S6A — Step to 30%  $\text{H}_2^{18}\text{O}$ .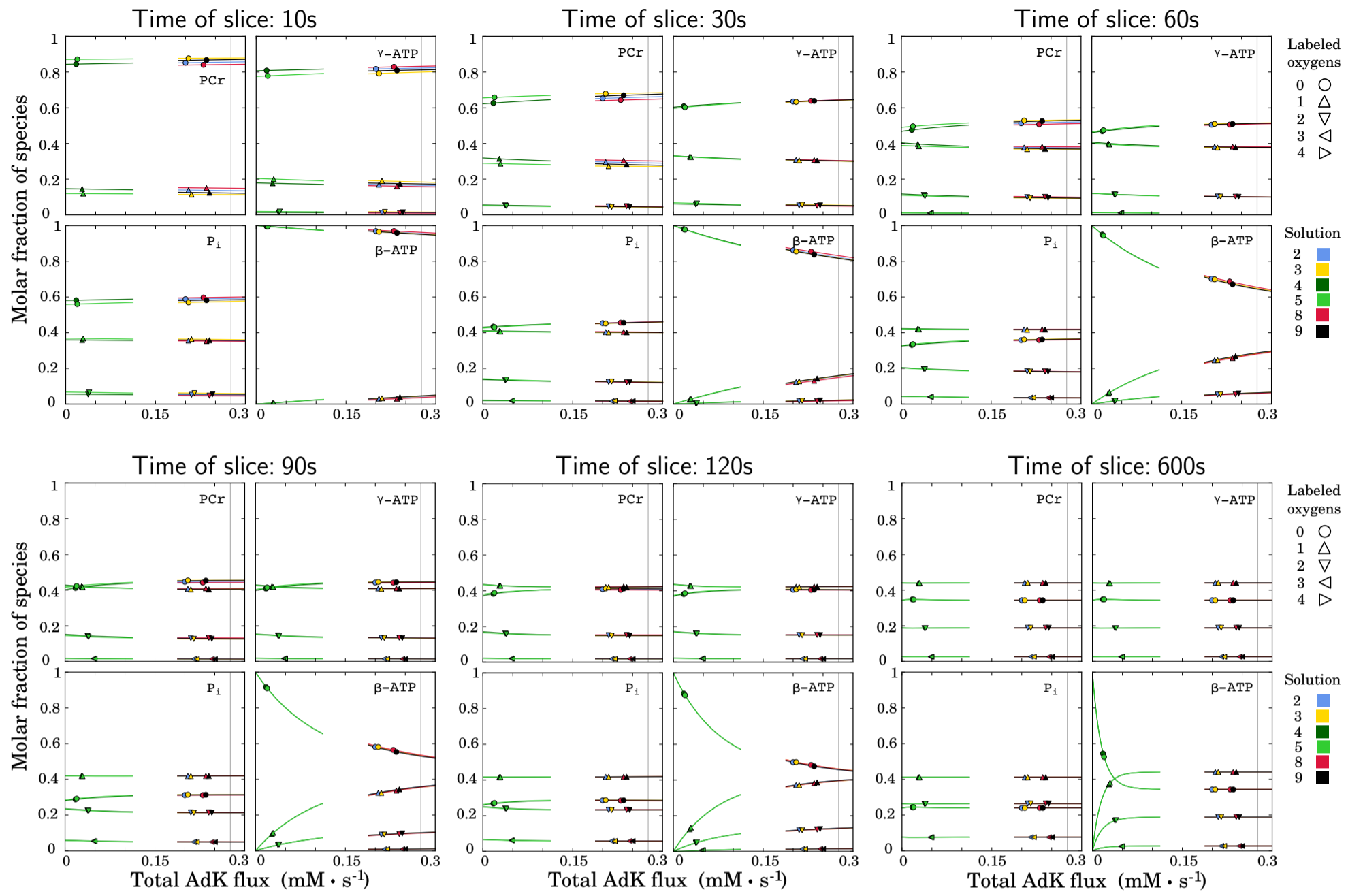

B

Figure S6B — Step to 100%  $\text{H}_2^{18}\text{O}$ .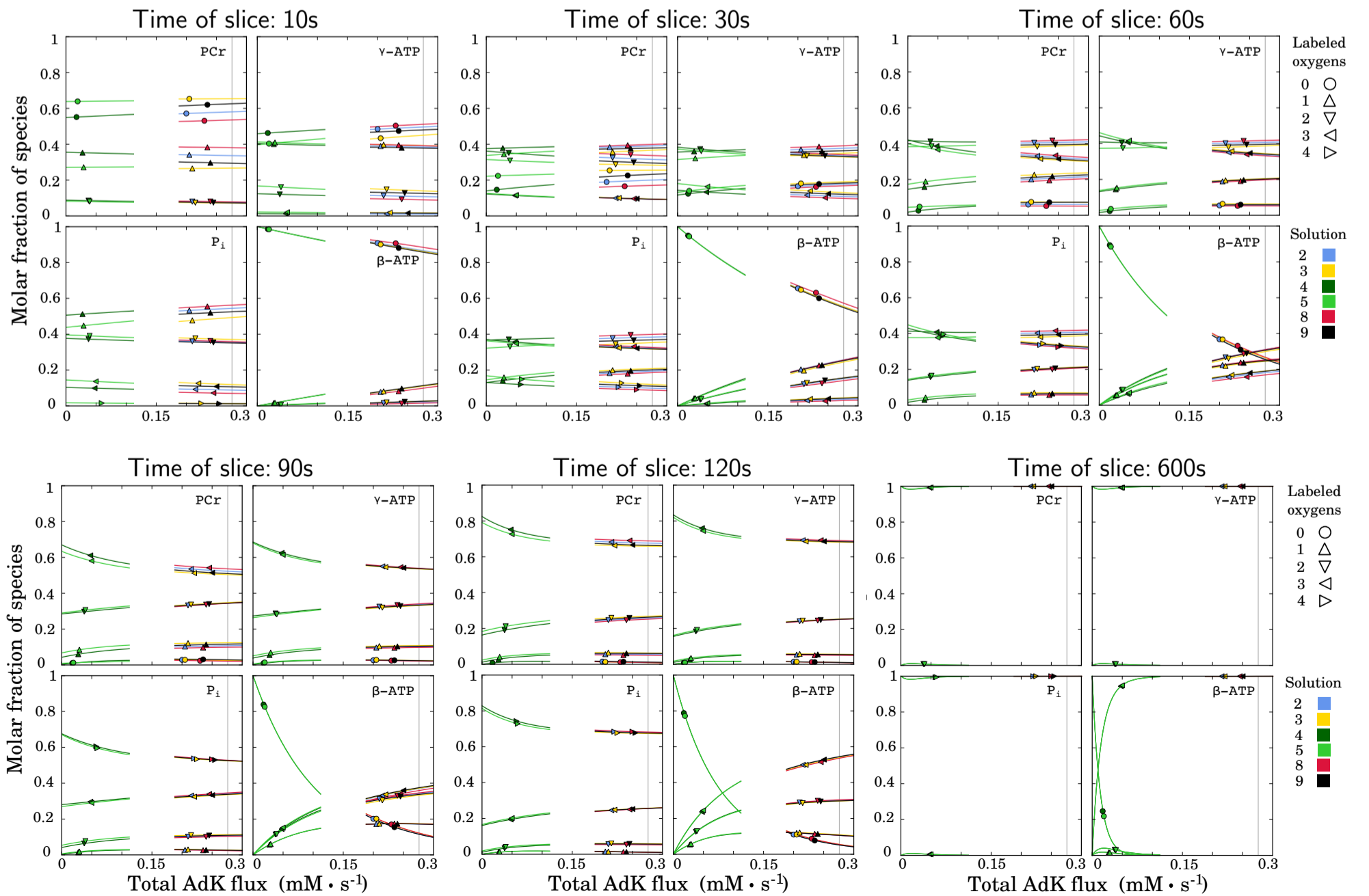

Supplement: Figure S6 — Change in labeling state with increasing energy export via AdK at 30 s after a step change to 30% (A), and 100% (B). All flux parameters are given in Table S1. The two ranges (0–0.112 and 0.188–0.3) correspond to the first two grey bars in Figure S2. The vertical grey line indicates the total AdK flux found in [20]. This line lies in a region where only the labeling state of can be used to find total AdK flux. In contrast, using 100% , total AdK flux is weakly sensitive to the labeling state of the other species. Line color and symbol notation are identical to Figure S1. (PDF) [file pcbi.1002795.s006.pdf]
