# Supplementary material for: Sensitivity Analysis of Flux Determination in Heart by H2 18O -provided Labeling Using a Dynamic Isotopologue Model of Energy Transfer Pathways
Source: PLoS Comput Biol. 2012 Dec 6;8(12):e1002795. doi: 10.1371/journal.pcbi.1002795 (PMC3516558; doi:10.1371/journal.pcbi.1002795)
Supplement: Table S1 — Flux distributions used in Figures 6–8 and Figures S1, S2, S3, S4, S5, S6. The rows in this table summarize all flux distributions considered in this paper. These flux distributions were selected to range between physiologically feasible states. Figures S2, S3, S4, S5, S6 plot predictions of the labeling state as one flux parameter (shown in bold) is varied over the range of the plot. Relationships between all other flux parameters are provided. Flux distribution and figure(s), are denoted by FD and Fig., respectively. Subscripts x, f, and r refer to the fraction of net flux, forward, and reverse fluxes, respectively. Superscripts o, i, and t refer to cytosolic, IMS, and total fluxes, respectively. (PDF) [file pcbi.1002795.s007.pdf]

Table S1

FD	Fig.	Fraction of energy export			CK fluxes, $CK_{net} = CK_x \cdot 2.25$ mM $\cdot s^{-1}$, $ATP + Cr \underset{CK_r}{\overset{CK_f}{\rightleftharpoons}} PCr + ADP$					AdK fluxes, $AdK_{net} = AdK_x \cdot 2.25$ mM $\cdot s^{-1}$, $ATP + AMP \underset{AdK_r}{\overset{AdK_f}{\rightleftharpoons}} 2\,ADP$				
		AdK_x	ATP_x	CK_x	CK_f^i mM $\cdot s^{-1}$	CK_r^i mM $\cdot s^{-1}$	CK_f^o mM $\cdot s^{-1}$	CK_r^o mM $\cdot s^{-1}$	CK^t mM $\cdot s^{-1}$	AdK_f^i mM $\cdot s^{-1}$	AdK_r^i mM $\cdot s^{-1}$	AdK_f^o mM $\cdot s^{-1}$	AdK_r^o mM $\cdot s^{-1}$	AdK^t mM $\cdot s^{-1}$
1	S1	0.05	0.45	0.5	1.12	0.0	4.88	6.0	6.0	0.944	1.06	AdK_r^i	AdK_f^i	2.0
	6,S2	0.05	0.45	0.5	1.12	0.0	4.88	6.0	6.0	0.0…0.944<br>$\frac{AdK^t-AdK_{net}}{2.0}$	0.112…1.06<br>$AdK_{net}+\frac{AdK^t-AdK_{net}}{2.0}$	AdK_r^i	AdK_f^i	**0.112…2.0**
	S3	0.05	0.45	0.5	1.12	0.0	4.88	6.0	6.0	**0.0…0.944**	0.112…1.06<br>$AdK_{net}+AdK_f^i$	2.0…1.06<br>$AdK^t-AdK_f^i$	1.89…0.944<br>$AdK^t-AdK_{net}-AdK_f^i$	2.0
	7,S5	0.05	0.45	0.5	1.12…3.56<br>$CK_{net}+\frac{CK^t-CK_{net}}{2.0}$	0.0…2.44<br>$\frac{CK^t-CK_{net}}{2.0}$	CK_r^i	CK_f^i	**1.12…6.0**	0.944	1.06	AdK_r^i	AdK_f^i	2.0
	8,S6	0.05	**0.0…0.95**	0.95…0.0<br>$1.0-ATP_x-AdK_x$	2.14…0.0<br>CK_{net}	0.0	3.86…6.0<br>CK^t-CK_{net}	6.0	6.0	0.944	1.06	AdK_r^i	AdK_f^i	2.0
2	S1	0.05	0.45	0.5	1.12	0.0	4.88	6.0	6.0	0.094	0.206	AdK_r^i	AdK_f^i	0.3
	S4	**0.0…0.05**	0.5…0.45<br>$1.0-CK_x-AdK_x$	0.5	1.12	0.0	4.88	6.0	6.0	0.094	0.094…0.206<br>$AdK_{net}+AdK_f^i$	AdK_r^i	AdK_f^i	0.188…0.3<br>$AdK_f^i+AdK_f^o$
	7,S5	0.05	0.45	0.5	1.12…3.56<br>$CK_{net}+\frac{CK^t-CK_{net}}{2.0}$	0.0…2.44<br>$\frac{CK^t-CK_{net}}{2.0}$	CK_r^i	CK_f^i	**1.12…6.0**	0.094	0.206	AdK_r^i	AdK_f^i	0.3
	8,S6	0.05	**0.0…0.95**	0.95…0.0<br>$1.0-ATP_x-AdK_x$	2.14…0.0<br>CK_{net}	0.0	3.86…6.0<br>CK^t-CK_{net}	6.0	6.0	0.094	0.206	AdK_r^i	AdK_f^i	0.3
3	S1	0.05	0.45	0.5	1.12	0.0	CK_r^i	CK_f^i	1.12	0.094	0.206	AdK_r^i	AdK_f^i	0.3
	6,S2	0.05	0.45	0.5	1.12	0.0	CK_r^i	CK_f^i	1.12	0.0…0.944<br>$\frac{AdK^t-AdK_{net}}{2.0}$	0.112…1.06<br>$AdK_{net}+\frac{AdK^t-AdK_{net}}{2.0}$	AdK_r^i	AdK_f^i	**0.112…2.0**
	S3	0.05	0.45	0.5	1.12	0.0	CK_r^i	CK_f^i	1.12	**0.0…0.094**	0.112…0.206<br>$AdK_{net}+AdK_f^i$	0.3…0.206<br>$AdK^t-AdK_f^i$	0.188…0.094<br>$AdK^t-AdK_{net}-AdK_f^i$	0.3
	S4	**0.0…0.05**	0.5…0.45<br>$1.0-CK_x-AdK_x$	0.5	1.12	0.0	CK_r^i	CK_f^i	1.12	0.094	0.094…0.206<br>$AdK_{net}+AdK_f^i$	AdK_r^i	AdK_f^i	0.188…0.3<br>$AdK_f^i+AdK_f^o$
	8,S6	0.05	**0.0…0.95**	0.95…0.0<br>$1.0-ATP_x-AdK_x$	2.14…0.0<br>CK_{net}	0.0	CK_r^i	CK_f^i	2.14…0.0<br>$CK_f^i+CK_f^o$	0.094	0.206	AdK_r^i	AdK_f^i	0.3
4	S1	0.05	0.45	0.5	1.12	0.0	4.88	6.0	6.0	0.0	0.112	AdK_r^i	AdK_f^i	0.112
	S4	**0.0…0.05**	0.5…0.45<br>$1.0-CK_x-AdK_x$	0.5	1.12	0.0	4.88	6.0	6.0	0.0	0…0.112<br>$AdK_{net}+AdK_f^i$	AdK_r^i	AdK_f^i	0.0…0.112<br>$AdK_f^i+AdK_f^o$
	7,S5	0.05	0.45	0.5	1.12…3.56<br>$CK_{net}+\frac{CK^t-CK_{net}}{2.0}$	0.0…2.44<br>$\frac{CK^t-CK_{net}}{2.0}$	CK_r^i	CK_f^i	**1.12…6.0**	0.0	0.112	AdK_r^i	AdK_f^i	0.112
	8,S6	0.05	**0.0…0.95**	0.95…0.0<br>$1.0-ATP_x-AdK_x$	2.14…0.0<br>CK_{net}	0.0	3.86…6.0<br>CK^t-CK_{net}	6.0	6.0	0.0	0.112	AdK_r^i	AdK_f^i	0.112
5	S1	0.05	0.45	0.5	1.12	0.0	CK_r^i	CK_f^i	1.12	0.0	0.112	AdK_r^i	AdK_f^i	0.112
	S4	**0.0…0.05**	0.5…0.45<br>$1.0-CK_x-AdK_x$	0.5	1.12	0.0	CK_r^i	CK_f^i	1.12	0.0	0…0.112<br>$AdK_{net}+AdK_f^i$	AdK_r^i	AdK_f^i	0.0…0.112<br>$AdK_f^i+AdK_f^o$
	8,S6	0.05	**0.0…0.95**	0.95…0.0<br>$1.0-ATP_x-AdK_x$	2.14…0.0<br>CK_{net}	0.0	CK_r^i	CK_f^i	2.14…0.0<br>$CK_f^i+CK_f^o$	0.0	0.112	AdK_r^i	AdK_f^i	0.112
6	S1	0.0	0.5	0.5	1.12	0.0	4.88	6.0	6.0	0.15	0.15	AdK_r^i	AdK_f^i	0.3
	6,S2	0.0	0.5	0.5	1.12	0.0	4.88	6.0	6.0	0.0…1.0<br>$\frac{AdK^t}{2.0}$	0.0…1.0<br>$\frac{AdK^t}{2.0}$	AdK_r^i	AdK_f^i	**0.0…2.0**
	S3	0.0	0.5	0.5	1.12	0.0	4.88	6.0	6.0	**0.0…0.15**	AdK_f^i	0.3…0.15<br>$AdK^t-AdK_f^i$	AdK_f^o	0.3
	7,S5	0.0	0.5	0.5	1.12…3.56<br>$CK_{net}+\frac{CK^t-CK_{net}}{2.0}$	0.0…2.44<br>$\frac{CK^t-CK_{net}}{2.0}$	CK_r^i	CK_f^i	**1.12…6.0**	0.15	0.15	AdK_r^i	AdK_f^i	0.3
7	S1	0.0	0.0	1.0	2.25	0.0	3.75	6.0	6.0	0.15	0.15	AdK_r^i	AdK_f^i	0.3
	6,S2	0.0	0.0	1.0	2.25	0.0	3.75	6.0	6.0	0.0…1.0<br>$\frac{AdK^t}{2.0}$	0.0…1.0<br>$\frac{AdK^t}{2.0}$	AdK_r^i	AdK_f^i	**0.0…2.0**
	S3	0.0	0.0	1.0	2.25	0.0	3.75	6.0	6.0	**0.0…0.15**	AdK_f^i	0.3…0.15<br>$AdK^t-AdK_f^i$	AdK_f^o	0.3
	7,S5	0.0	0.0	1.0	2.25…4.12<br>$CK_{net}+\frac{CK^t-CK_{net}}{2.0}$	0.0…1.89<br>$\frac{CK^t-CK_{net}}{2.0}$	CK_r^i	CK_f^i	**2.25…6.0**	0.15	0.15	AdK_r^i	AdK_f^i	0.3
	8,S6	0.0	**0.0…1.0**	1.0…0.0<br>$1.0-ATP_x$	2.25…0.0<br>CK_{net}	0.0	3.75…6.0<br>CK^t-CK_{net}	6.0	6.0	0.15	0.15	AdK_r^i	AdK_f^i	0.3
8	S1	0.05	0.0	0.95	2.14	0.0	3.86	6.0	6.0	0.094	0.206	AdK_r^i	AdK_f^i	0.3
	6,S2	0.05	0.0	0.95	2.14	0.0	3.86	6.0	6.0	0.0…0.944<br>$\frac{AdK^t-AdK_{net}}{2.0}$	0.112…1.06<br>$AdK_{net}+\frac{AdK^t-AdK_{net}}{2.0}$	AdK_r^i	AdK_f^i	**0.112…2.0**
	S3	0.05	0.0	0.95	2.14	0.0	3.86	6.0	6.0	**0.0…0.094**	0.112…0.206<br>$AdK_{net}+AdK_f^i$	0.3…0.206<br>$AdK^t-AdK_f^i$	0.188…0.094<br>$AdK^t-AdK_{net}-AdK_f^i$	0.3
	S4	**0.0…0.05**	0.05…0.0<br>$1.0-CK_x-AdK_x$	0.95	2.14	0.0	3.86	6.0	6.0	0.094	0.094…0.206<br>$AdK_{net}+AdK_f^i$	AdK_r^i	AdK_f^i	0.188…0.3<br>$AdK_f^i+AdK_f^o$
	7,S5	0.05	0.0	0.95	2.14…4.07<br>$CK_{net}+\frac{CK^t-CK_{net}}{2.0}$	0.0…1.93<br>$\frac{CK^t-CK_{net}}{2.0}$	CK_r^i	CK_f^i	**2.14…6.0**	0.094	0.206	AdK_r^i	AdK_f^i	0.3
9	S1	0.05	0.95	0.0	0.0	0.0	6.0	6.0	6.0	0.094	0.206	AdK_r^i	AdK_f^i	0.3
	6,S2	0.05	0.95	0.0	0.0	0.0	6.0	6.0	6.0	0.0…0.944<br>$\frac{AdK^t-AdK_{net}}{2.0}$	0.112…1.06<br>$AdK_{net}+\frac{AdK^t-AdK_{net}}{2.0}$	AdK_r^i	AdK_f^i	**0.112…2.0**
	S3	0.05	0.95	0.0	0.0	0.0	6.0	6.0	6.0	**0.0…0.094**	0.112…0.206<br>$AdK_{net}+AdK_f^i$	0.3…0.206<br>$AdK^t-AdK_f^i$	0.188…0.094<br>$AdK^t-AdK_{net}-AdK_f^i$	0.3
	S4	**0.0…0.05**	1.0…0.95<br>$1.0-CK_x-AdK_x$	0.0	0.0	0.0	6.0	6.0	6.0	0.094	0.094…0.206<br>$AdK_{net}+AdK_f^i$	AdK_r^i	AdK_f^i	0.188…0.3<br>$AdK_f^i+AdK_f^o$
	7,S5	0.05	0.95	0.0	0.0…3.0<br>$CK_{net}+\frac{CK^t-CK_{net}}{2.0}$	0.0…3.0<br>$\frac{CK^t-CK_{net}}{2.0}$	CK_r^i	CK_f^i	**0.0…6.0**	0.094	0.206	AdK_r^i	AdK_f^i	0.3
