# Supplementary material for: Sensitivity Analysis of Flux Determination in Heart by H2 18O -provided Labeling Using a Dynamic Isotopologue Model of Energy Transfer Pathways
Source: PLoS Comput Biol. 2012 Dec 6;8(12):e1002795. doi: 10.1371/journal.pcbi.1002795 (PMC3516558; doi:10.1371/journal.pcbi.1002795)
Supplement: Table S2 — Parameter ranges used to construct Figure 4. In total, 36 fluxes are listed, however, these are derived from 18 exchange flux parameters. In addition, 18 pool size parameters are included. (PDF) [file pcbi.1002795.s008.pdf]

Table S2

Flux	Lower value $mM \cdot s^{-1}$	Upper value $mM \cdot s^{-1}$	Pool size	Lower value mM	Upper value mM
ν^{f}_{AdKi}	0	10.944	D_M	0.008021875	0.010853125
ν^{r}_{AdKi}	1.06	11.06	D_I	0.00064175	0.00086825
ν^{f}_{AdKo}	1.06	11.06	D_O	0.054227875	0.073367125
ν^{r}_{AdKo}	0	10.944	D_E	0.00064175	0.00086825
ν^{f}_{ASe}	2.35	12.35	D_S	0.00064175	0.00086825
ν^{r}_{ASe}	0.10	10.10	T_M	0.8021875	1.0853125
ν^{f}_{ASs}	2.35	12.35	T_I	0.064175	0.086825
ν^{r}_{ASs}	0.10	10.10	T_O	5.54472	7.50168
ν^{f}_{CKi}	1.12	11.12	T_E	0.00320875	0.00434125
ν^{r}_{CKi}	0	10.0	T_S	0.00320875	0.00434125
ν^{f}_{CKo}	4.88	14.88	P_M	0.088125	0.3525
ν^{r}_{CKo}	6.0	16.0	P_O	0.61617	2.46468
ν^{f}_{ATPoe}	2.25	2.25	P_E	0.0003525	0.00141
ν^{f}_{ATPsm}	2.25	2.25	P_S	0.0003525	0.00141
ν^{f}_{Peo}	2.25	52.25	C_I	0.1394	0.1886
ν^{r}_{Peo}	0	50.0	C_O	13.8006	18.6714
ν^{f}_{Pms}	2.25	52.25	W_E	0.00025	0.001
ν^{r}_{Pms}	0	50.0	W_S	0.00025	0.001
ν^{f}_{Weo}	2.25	52.25			
ν^{r}_{Weo}	0	50.0			
ν^{f}_{Wos}	2.25	52.25			
ν^{r}_{Wos}	0	50.0			
ν^{f}_{ADPeo}	2.25	52.25			
ν^{r}_{ADPeo}	0	50.0			
ν^{f}_{ADPms}	2.25	52.25			
ν^{r}_{ADPms}	0	50.0			
ν^{f}_{ADPim}	2.25	2.55			
ν^{r}_{ADPim}	0	0.30			
ν^{f}_{ADPoi}	2.25	2.55			
ν^{r}_{ADPoi}	0	0.30			
ν^{f}_{ATPmi}	2.25	2.55			
ν^{r}_{ATPmi}	0	0.30			
ν^{f}_{ATPio}	2.25	2.55			
ν^{r}_{ATPio}	0	0.30			
ν^{f}_{Cio}	2.25	4.25			
ν^{r}_{Cio}	0	2.0			
ν^{f}_{Pom}	2.25	2.25			
