# Supplementary material for: Sensitivity Analysis of Flux Determination in Heart by H2 18O -provided Labeling Using a Dynamic Isotopologue Model of Energy Transfer Pathways
Source: PLoS Comput Biol. 2012 Dec 6;8(12):e1002795. doi: 10.1371/journal.pcbi.1002795 (PMC3516558; doi:10.1371/journal.pcbi.1002795)
Supplement: Text S1 — Derivation of the model equations with all intermediate steps. (PDF) [file pcbi.1002795.s009.pdf]

Text S1

Text S1 for the paper entitled:
Sensitivity analysis of flux determination in heart by $H_2^{18}O$-provided labeling using a dynamic isotopologue model of energy transfer pathways

David W. Schryer, Pearu Peterson, Ardo Illaste, and Marko Vendelin
Laboratory of Systems Biology, Institute of Cybernetics, Tallinn University of Technology, Estonia

This document presents the definition of the phosphotransfer network studied in the main text and the derivation of the model equations. All intermediate derivation steps are included as well as the full set of model equations.

Contents

1 Definition of phosphotransfer network **1**
1.1 Compartments included in the model . . . 1
1.2 Species included in the model . . . 1
1.3 Phosphotransfer network reactions . . . 2
1.4 Oxygen atom mappings . . . 3

2 Model derivation **3**
2.1 Individual isotopic transformations . . . 4
2.2 Kinetic equations for isotopomers . . . 4
2.3 Pool definitions . . . 30
2.4 Kinetic equations for mass isotopomers . . . 34

1 DEFINITION OF PHOSPHOTRANSFER NETWORK

The phosphotransfer network under consideration is compartmentalized such that each of the three types of enzymatic reactions occurs in several compartments (see Figure S7 below). The species that participate in these reactions move between compartments via transport reactions.

1.1 Compartments included in the model

The reactions and species that form this network are located in three compartments: cytosol (O), intermitochondrial membrane space (I), and the mitochondrial matrix (M). In addition, enzyme bound compartments are included for both ATP synthase (S) and the ATPase reactions (E). The names of all species, reactions and fluxes include one or two of these compartmental tags.

1.2 Species included in the model

All species that become labeled with ^{18}O are considered. We introduce a compact notation for these species:

- five species of ADP (D): D_M, D_I, D_O, D_E, D_S;
- five species of ATP (T): T_M, T_I, T_O, T_E, T_S;
- four species of inorganic phosphate (P): P_M, P_O, P_E, P_S;
- two species of phosphocreatine (C): C_I, C_O;

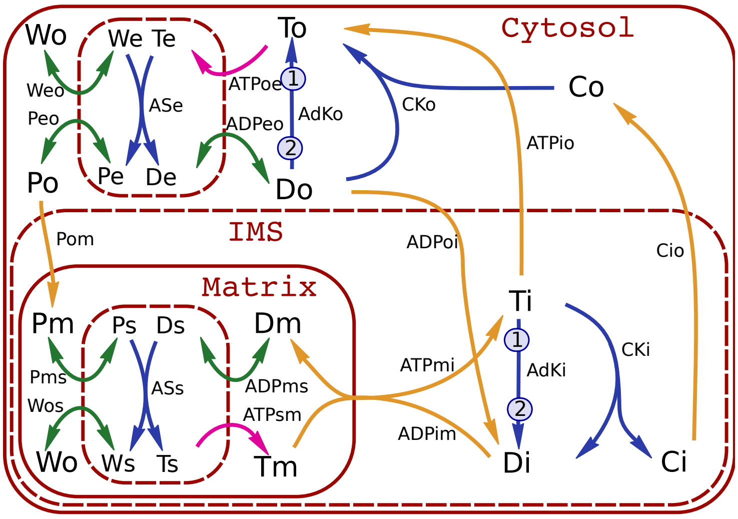

Figure S7: Network diagram with flux names. Note that this is the same as Figure 1 in the main text with flux values replaced with the names of each reaction used in this document, and the metabolite species names that are used in the equations in this document. The species names include subscripts that indicate compartmental location.

- two species of water (W): W_E, W_S;

Adenosine monophosphate is not included since it does not become isotopically labeled with ^{18}O. The isotopic labeling state of intercellular water (W_O) is specified as a function for model simulation.

1.3 Phosphotransfer network reactions

The compartmentalized model of the phosphotransfer network is defined by six bidirectional enzymatic reactions (AdKi, AdKo, ASe, ASs, CKi, CKo), two unidirectional substrate exchange reactions (ATPoe, ATPsm), six bidirectional substrate exchange reactions (Peo, Pms, Weo, Wos, ADPeo, ADPms), five bidirectional transport reactions (ADPim, ADPoi, ATPio, ATPmi, Cio), and one unidirectional transport reaction (Pom). The network of these fluxes is presented in Figure S7, and their reaction definitions are given below.

AdK reaction in the IMS: $T_I \xrightleftharpoons[\nu_r^{\text{AdKi}}]{\nu_f^{\text{AdKi}}} D_I + D_I$

AdK reaction in the cytosol: $T_O \xrightleftharpoons[\nu_r^{\text{AdKo}}]{\nu_f^{\text{AdKo}}} D_O + D_O$

ATPase reactions: $T_E + W_E \xrightleftharpoons[\nu_r^{\text{ASe}}]{\nu_f^{\text{ASe}}} D_E + P_E$

ATP synthase: $D_S + P_S \xrightleftharpoons[\nu_r^{\text{ASs}}]{\nu_f^{\text{ASs}}} T_S + W_S$

CK reaction in the IMS: $T_I \xrightleftharpoons[\nu_r^{\text{CKi}}]{\nu_f^{\text{CKi}}} D_I + C_I$

CK reaction in the cytosol: $T_O \xrightleftharpoons[\nu_r^{\text{CKo}}]{\nu_f^{\text{CKo}}} C_O + D_O$

Transport of ATP (cytosol, ATPase): $T_O \xrightarrow{\nu_f^{\text{ATPoe}}} T_E$

Transport of ATP (matrix, ATP syn.): $T_S \xrightarrow{\nu_f^{\text{ATPsm}}} T_M$

Transport of P (cytosol, ATPase): $P_E \xrightleftharpoons[\nu_r^{\text{Peo}}]{\nu_f^{\text{Peo}}} P_O$

Transport of P (matrix, ATP syn.): $P_M \xrightleftharpoons[\nu_r^{\text{Pms}}]{\nu_f^{\text{Pms}}} P_S$

Transport of water (cytosol, ATPase): $W_E \xrightleftharpoons[\nu_r^{\text{Weo}}]{\nu_f^{\text{Weo}}} W_O$

Transport of water (cytosol, ATP syn.): $W_O \xrightleftharpoons[\nu_r^{\text{Wos}}]{\nu_f^{\text{Wos}}} W_S$

Transport of ADP (cytosol, ATPase): $D_E \xrightleftharpoons[\nu_r^{\text{ADPeo}}]{\nu_f^{\text{ADPeo}}} D_O$

Transport of ADP (matrix, ATP syn.): $D_M \xrightleftharpoons[\nu_r^{\text{ADPms}}]{\nu_f^{\text{ADPms}}} D_S$

Transport of ADP (IMS, matrix): $\mathrm{D_I} \underset{\nu^r_{\mathrm{ADPIM}}}{\overset{\nu^f_{\mathrm{ADPIM}}}{\rightleftharpoons}} \mathrm{D_M}$

Transport of ADP (cytosol, IMS): $\mathrm{D_O} \underset{\nu^r_{\mathrm{ADPOI}}}{\overset{\nu^f_{\mathrm{ADPOI}}}{\rightleftharpoons}} \mathrm{D_I}$

Transport of ATP (cytosol, IMS): $\mathrm{T_I} \underset{\nu^r_{\mathrm{ATPIO}}}{\overset{\nu^f_{\mathrm{ATPIO}}}{\rightleftharpoons}} \mathrm{T_O}$

Transport of ATP (IMS, matrix): $\mathrm{T_M} \underset{\nu^r_{\mathrm{ATPMI}}}{\overset{\nu^f_{\mathrm{ATPMI}}}{\rightleftharpoons}} \mathrm{T_I}$

Transport of CK (cytosol, IMS): $\mathrm{C_I} \underset{\nu^r_{\mathrm{CIO}}}{\overset{\nu^f_{\mathrm{CIO}}}{\rightleftharpoons}} \mathrm{C_O}$

Transport of P (cytosol, matrix): $\mathrm{P_O} \xrightarrow{\nu^f_{\mathrm{POM}}} \mathrm{P_M}$

1.4 Oxygen atom mappings

All three oxygen atoms in every phosphoryl group of all species have an equal probability of being isotopically labeled (see main text).

2 MODEL DERIVATION

The derivation of the mass balance equations used to calculate the dynamic change in labeling state for all oxygen atoms in this system is discussed in the main text. In short, (I) the full set of individual isotopic transformations is generated (Section 2.1), (II) these transformations are combined into mass balances around each isotopologue in the system (Section 2.2), (III) mass isotopologue pool relations are composed taking into account oxygen atom mappings (Section 2.3), and (IV) mass isotopologue balances are composed by collecting the isotopologue balances according to the pool relations (Section 2.4). A program was written in Python to generate these equations. This program implements symbolic manipulation tools specifically designed to carry out steps (I) through (IV), and is available upon request.

Note that a number of subexpressions, such as the sum of all inorganic phosphate isotopologues in the first four mass isotopologue balances (and others), always equal one. These subexpressions were not simplified since these terms act as stabilizing attractors during integration.

A succinct notation was devised to make the isotopologue equations more readable. The labeling state of the oxygen atoms attached to the mobile phosphorus atom are written in a column of filled or unfilled circles corresponding to ^{18}O and ^{16}O, respectively. If a species has more than one phosphoryl group, for example, the two phosphoryl groups in T each is written in a separate columns of three oxygen atoms with the outermost row corresponding to the outermost phosphoryl group (i.e. γ-ATPin the case of T). T is written as $\mathrm{T_E}\substack{\bullet\bullet\\ \bullet\bullet\\ \bullet\bullet}$, and the four oxygen atoms of P are written in one column (i.e. $\mathrm{P_O}\substack{\bullet\\ \bullet\\ \bullet\\ \bullet}$).

2.1 Individual isotopic transformations

The isotopic transformations for one reaction are provided as an example. The kinetic equations in Section 2.2 are constructed from the set of all transformations from all reactions.

$$\mathrm{T_I}\begin{smallmatrix}\bullet&\bullet\\\bullet&\bullet\\\bullet&\bullet\end{smallmatrix}\overset{\nu_f,\nu_r}{\overset{\mathrm{AK_I}}{\rightleftharpoons}}\mathrm{D_I}\begin{smallmatrix}\bullet\\\bullet\\\bullet\end{smallmatrix}+\mathrm{D_I}\begin{smallmatrix}\bullet\\\bullet\\\bullet\end{smallmatrix}\qquad \mathrm{T_I}\begin{smallmatrix}\bullet&\bullet\\\bullet&\bullet\\\bullet&\circ\end{smallmatrix}\overset{\nu_f,\nu_r}{\overset{\mathrm{AK_I}}{\rightleftharpoons}}\mathrm{D_I}\begin{smallmatrix}\bullet\\\bullet\\\bullet\end{smallmatrix}+\mathrm{D_I}\begin{smallmatrix}\bullet\\\bullet\\\circ\end{smallmatrix}\qquad \mathrm{T_I}\begin{smallmatrix}\bullet&\bullet\\\bullet&\circ\\\bullet&\bullet\end{smallmatrix}\overset{\nu_f,\nu_r}{\overset{\mathrm{AK_I}}{\rightleftharpoons}}\mathrm{D_I}\begin{smallmatrix}\bullet\\\bullet\\\bullet\end{smallmatrix}+\mathrm{D_I}\begin{smallmatrix}\bullet\\\circ\\\bullet\end{smallmatrix}\qquad \mathrm{T_I}\begin{smallmatrix}\bullet&\bullet\\\bullet&\circ\\\bullet&\circ\end{smallmatrix}\overset{\nu_f,\nu_r}{\overset{\mathrm{AK_I}}{\rightleftharpoons}}\mathrm{D_I}\begin{smallmatrix}\bullet\\\bullet\\\bullet\end{smallmatrix}+\mathrm{D_I}\begin{smallmatrix}\bullet\\\circ\\\circ\end{smallmatrix}$$

$$\mathrm{T_I}\begin{smallmatrix}\bullet&\circ\\\bullet&\bullet\\\bullet&\bullet\end{smallmatrix}\overset{\nu_f,\nu_r}{\overset{\mathrm{AK_I}}{\rightleftharpoons}}\mathrm{D_I}\begin{smallmatrix}\bullet\\\bullet\\\bullet\end{smallmatrix}+\mathrm{D_I}\begin{smallmatrix}\circ\\\bullet\\\bullet\end{smallmatrix}\qquad \mathrm{T_I}\begin{smallmatrix}\bullet&\circ\\\bullet&\bullet\\\bullet&\circ\end{smallmatrix}\overset{\nu_f,\nu_r}{\overset{\mathrm{AK_I}}{\rightleftharpoons}}\mathrm{D_I}\begin{smallmatrix}\bullet\\\bullet\\\bullet\end{smallmatrix}+\mathrm{D_I}\begin{smallmatrix}\circ\\\bullet\\\circ\end{smallmatrix}\qquad \mathrm{T_I}\begin{smallmatrix}\bullet&\circ\\\bullet&\circ\\\bullet&\bullet\end{smallmatrix}\overset{\nu_f,\nu_r}{\overset{\mathrm{AK_I}}{\rightleftharpoons}}\mathrm{D_I}\begin{smallmatrix}\bullet\\\bullet\\\bullet\end{smallmatrix}+\mathrm{D_I}\begin{smallmatrix}\circ\\\circ\\\bullet\end{smallmatrix}\qquad \mathrm{T_I}\begin{smallmatrix}\bullet&\circ\\\bullet&\circ\\\bullet&\circ\end{smallmatrix}\overset{\nu_f,\nu_r}{\overset{\mathrm{AK_I}}{\rightleftharpoons}}\mathrm{D_I}\begin{smallmatrix}\bullet\\\bullet\\\bullet\end{smallmatrix}+\mathrm{D_I}\begin{smallmatrix}\circ\\\circ\\\circ\end{smallmatrix}$$

$$\mathrm{T_I}\begin{smallmatrix}\bullet&\bullet\\\bullet&\bullet\\\circ&\bullet\end{smallmatrix}\overset{\nu_f,\nu_r}{\overset{\mathrm{AK_I}}{\rightleftharpoons}}\mathrm{D_I}\begin{smallmatrix}\bullet\\\bullet\\\circ\end{smallmatrix}+\mathrm{D_I}\begin{smallmatrix}\bullet\\\bullet\\\bullet\end{smallmatrix}\qquad \mathrm{T_I}\begin{smallmatrix}\bullet&\bullet\\\bullet&\bullet\\\circ&\circ\end{smallmatrix}\overset{\nu_f,\nu_r}{\overset{\mathrm{AK_I}}{\rightleftharpoons}}\mathrm{D_I}\begin{smallmatrix}\bullet\\\bullet\\\circ\end{smallmatrix}+\mathrm{D_I}\begin{smallmatrix}\bullet\\\bullet\\\circ\end{smallmatrix}\qquad \mathrm{T_I}\begin{smallmatrix}\bullet&\bullet\\\bullet&\circ\\\circ&\bullet\end{smallmatrix}\overset{\nu_f,\nu_r}{\overset{\mathrm{AK_I}}{\rightleftharpoons}}\mathrm{D_I}\begin{smallmatrix}\bullet\\\bullet\\\circ\end{smallmatrix}+\mathrm{D_I}\begin{smallmatrix}\bullet\\\circ\\\bullet\end{smallmatrix}\qquad \mathrm{T_I}\begin{smallmatrix}\bullet&\bullet\\\bullet&\circ\\\circ&\circ\end{smallmatrix}\overset{\nu_f,\nu_r}{\overset{\mathrm{AK_I}}{\rightleftharpoons}}\mathrm{D_I}\begin{smallmatrix}\bullet\\\bullet\\\circ\end{smallmatrix}+\mathrm{D_I}\begin{smallmatrix}\bullet\\\circ\\\circ\end{smallmatrix}$$

$$\mathrm{T_I}\begin{smallmatrix}\bullet&\circ\\\bullet&\bullet\\\circ&\bullet\end{smallmatrix}\overset{\nu_f,\nu_r}{\overset{\mathrm{AK_I}}{\rightleftharpoons}}\mathrm{D_I}\begin{smallmatrix}\bullet\\\bullet\\\circ\end{smallmatrix}+\mathrm{D_I}\begin{smallmatrix}\circ\\\bullet\\\bullet\end{smallmatrix}\qquad \mathrm{T_I}\begin{smallmatrix}\bullet&\circ\\\bullet&\bullet\\\circ&\circ\end{smallmatrix}\overset{\nu_f,\nu_r}{\overset{\mathrm{AK_I}}{\rightleftharpoons}}\mathrm{D_I}\begin{smallmatrix}\bullet\\\bullet\\\circ\end{smallmatrix}+\mathrm{D_I}\begin{smallmatrix}\circ\\\bullet\\\circ\end{smallmatrix}\qquad \mathrm{T_I}\begin{smallmatrix}\bullet&\circ\\\bullet&\circ\\\circ&\bullet\end{smallmatrix}\overset{\nu_f,\nu_r}{\overset{\mathrm{AK_I}}{\rightleftharpoons}}\mathrm{D_I}\begin{smallmatrix}\bullet\\\bullet\\\circ\end{smallmatrix}+\mathrm{D_I}\begin{smallmatrix}\circ\\\circ\\\bullet\end{smallmatrix}\qquad \mathrm{T_I}\begin{smallmatrix}\bullet&\circ\\\bullet&\circ\\\circ&\circ\end{smallmatrix}\overset{\nu_f,\nu_r}{\overset{\mathrm{AK_I}}{\rightleftharpoons}}\mathrm{D_I}\begin{smallmatrix}\bullet\\\bullet\\\circ\end{smallmatrix}+\mathrm{D_I}\begin{smallmatrix}\circ\\\circ\\\circ\end{smallmatrix}$$

$$\mathrm{T_I}\begin{smallmatrix}\bullet&\bullet\\\circ&\bullet\\\bullet&\bullet\end{smallmatrix}\overset{\nu_f,\nu_r}{\overset{\mathrm{AK_I}}{\rightleftharpoons}}\mathrm{D_I}\begin{smallmatrix}\bullet\\\circ\\\bullet\end{smallmatrix}+\mathrm{D_I}\begin{smallmatrix}\bullet\\\bullet\\\bullet\end{smallmatrix}\qquad \mathrm{T_I}\begin{smallmatrix}\bullet&\bullet\\\circ&\bullet\\\bullet&\circ\end{smallmatrix}\overset{\nu_f,\nu_r}{\overset{\mathrm{AK_I}}{\rightleftharpoons}}\mathrm{D_I}\begin{smallmatrix}\bullet\\\circ\\\bullet\end{smallmatrix}+\mathrm{D_I}\begin{smallmatrix}\bullet\\\bullet\\\circ\end{smallmatrix}\qquad \mathrm{T_I}\begin{smallmatrix}\bullet&\bullet\\\circ&\circ\\\bullet&\bullet\end{smallmatrix}\overset{\nu_f,\nu_r}{\overset{\mathrm{AK_I}}{\rightleftharpoons}}\mathrm{D_I}\begin{smallmatrix}\bullet\\\circ\\\bullet\end{smallmatrix}+\mathrm{D_I}\begin{smallmatrix}\bullet\\\circ\\\bullet\end{smallmatrix}\qquad \mathrm{T_I}\begin{smallmatrix}\bullet&\bullet\\\circ&\circ\\\bullet&\circ\end{smallmatrix}\overset{\nu_f,\nu_r}{\overset{\mathrm{AK_I}}{\rightleftharpoons}}\mathrm{D_I}\begin{smallmatrix}\bullet\\\circ\\\bullet\end{smallmatrix}+\mathrm{D_I}\begin{smallmatrix}\bullet\\\circ\\\circ\end{smallmatrix}$$

$$\mathrm{T_I}\begin{smallmatrix}\bullet&\circ\\\circ&\bullet\\\bullet&\bullet\end{smallmatrix}\overset{\nu_f,\nu_r}{\overset{\mathrm{AK_I}}{\rightleftharpoons}}\mathrm{D_I}\begin{smallmatrix}\bullet\\\circ\\\bullet\end{smallmatrix}+\mathrm{D_I}\begin{smallmatrix}\circ\\\bullet\\\bullet\end{smallmatrix}\qquad \mathrm{T_I}\begin{smallmatrix}\bullet&\circ\\\circ&\bullet\\\bullet&\circ\end{smallmatrix}\overset{\nu_f,\nu_r}{\overset{\mathrm{AK_I}}{\rightleftharpoons}}\mathrm{D_I}\begin{smallmatrix}\bullet\\\circ\\\bullet\end{smallmatrix}+\mathrm{D_I}\begin{smallmatrix}\circ\\\bullet\\\circ\end{smallmatrix}\qquad \mathrm{T_I}\begin{smallmatrix}\bullet&\circ\\\circ&\circ\\\bullet&\bullet\end{smallmatrix}\overset{\nu_f,\nu_r}{\overset{\mathrm{AK_I}}{\rightleftharpoons}}\mathrm{D_I}\begin{smallmatrix}\bullet\\\circ\\\bullet\end{smallmatrix}+\mathrm{D_I}\begin{smallmatrix}\circ\\\circ\\\bullet\end{smallmatrix}\qquad \mathrm{T_I}\begin{smallmatrix}\bullet&\circ\\\circ&\circ\\\bullet&\circ\end{smallmatrix}\overset{\nu_f,\nu_r}{\overset{\mathrm{AK_I}}{\rightleftharpoons}}\mathrm{D_I}\begin{smallmatrix}\bullet\\\circ\\\bullet\end{smallmatrix}+\mathrm{D_I}\begin{smallmatrix}\circ\\\circ\\\circ\end{smallmatrix}$$

$$\mathrm{T_I}\begin{smallmatrix}\bullet&\bullet\\\circ&\bullet\\\circ&\bullet\end{smallmatrix}\overset{\nu_f,\nu_r}{\overset{\mathrm{AK_I}}{\rightleftharpoons}}\mathrm{D_I}\begin{smallmatrix}\bullet\\\circ\\\circ\end{smallmatrix}+\mathrm{D_I}\begin{smallmatrix}\bullet\\\bullet\\\bullet\end{smallmatrix}\qquad \mathrm{T_I}\begin{smallmatrix}\bullet&\bullet\\\circ&\bullet\\\circ&\circ\end{smallmatrix}\overset{\nu_f,\nu_r}{\overset{\mathrm{AK_I}}{\rightleftharpoons}}\mathrm{D_I}\begin{smallmatrix}\bullet\\\circ\\\circ\end{smallmatrix}+\mathrm{D_I}\begin{smallmatrix}\bullet\\\bullet\\\circ\end{smallmatrix}\qquad \mathrm{T_I}\begin{smallmatrix}\bullet&\bullet\\\circ&\circ\\\circ&\bullet\end{smallmatrix}\overset{\nu_f,\nu_r}{\overset{\mathrm{AK_I}}{\rightleftharpoons}}\mathrm{D_I}\begin{smallmatrix}\bullet\\\circ\\\circ\end{smallmatrix}+\mathrm{D_I}\begin{smallmatrix}\bullet\\\circ\\\bullet\end{smallmatrix}\qquad \mathrm{T_I}\begin{smallmatrix}\bullet&\bullet\\\circ&\circ\\\circ&\circ\end{smallmatrix}\overset{\nu_f,\nu_r}{\overset{\mathrm{AK_I}}{\rightleftharpoons}}\mathrm{D_I}\begin{smallmatrix}\bullet\\\circ\\\circ\end{smallmatrix}+\mathrm{D_I}\begin{smallmatrix}\bullet\\\circ\\\circ\end{smallmatrix}$$

$$\mathrm{T_I}\begin{smallmatrix}\bullet&\circ\\\circ&\bullet\\\circ&\bullet\end{smallmatrix}\overset{\nu_f,\nu_r}{\overset{\mathrm{AK_I}}{\rightleftharpoons}}\mathrm{D_I}\begin{smallmatrix}\bullet\\\circ\\\circ\end{smallmatrix}+\mathrm{D_I}\begin{smallmatrix}\circ\\\bullet\\\bullet\end{smallmatrix}\qquad \mathrm{T_I}\begin{smallmatrix}\bullet&\circ\\\circ&\bullet\\\circ&\circ\end{smallmatrix}\overset{\nu_f,\nu_r}{\overset{\mathrm{AK_I}}{\rightleftharpoons}}\mathrm{D_I}\begin{smallmatrix}\bullet\\\circ\\\circ\end{smallmatrix}+\mathrm{D_I}\begin{smallmatrix}\circ\\\bullet\\\circ\end{smallmatrix}\qquad \mathrm{T_I}\begin{smallmatrix}\bullet&\circ\\\circ&\circ\\\circ&\bullet\end{smallmatrix}\overset{\nu_f,\nu_r}{\overset{\mathrm{AK_I}}{\rightleftharpoons}}\mathrm{D_I}\begin{smallmatrix}\bullet\\\circ\\\circ\end{smallmatrix}+\mathrm{D_I}\begin{smallmatrix}\circ\\\circ\\\bullet\end{smallmatrix}\qquad \mathrm{T_I}\begin{smallmatrix}\bullet&\circ\\\circ&\circ\\\circ&\circ\end{smallmatrix}\overset{\nu_f,\nu_r}{\overset{\mathrm{AK_I}}{\rightleftharpoons}}\mathrm{D_I}\begin{smallmatrix}\bullet\\\circ\\\circ\end{smallmatrix}+\mathrm{D_I}\begin{smallmatrix}\circ\\\circ\\\circ\end{smallmatrix}$$

$$\mathrm{T_I}\begin{smallmatrix}\circ&\bullet\\\bullet&\bullet\\\bullet&\bullet\end{smallmatrix}\overset{\nu_f,\nu_r}{\overset{\mathrm{AK_I}}{\rightleftharpoons}}\mathrm{D_I}\begin{smallmatrix}\circ\\\bullet\\\bullet\end{smallmatrix}+\mathrm{D_I}\begin{smallmatrix}\bullet\\\bullet\\\bullet\end{smallmatrix}\qquad \mathrm{T_I}\begin{smallmatrix}\circ&\bullet\\\bullet&\bullet\\\bullet&\circ\end{smallmatrix}\overset{\nu_f,\nu_r}{\overset{\mathrm{AK_I}}{\rightleftharpoons}}\mathrm{D_I}\begin{smallmatrix}\circ\\\bullet\\\bullet\end{smallmatrix}+\mathrm{D_I}\begin{smallmatrix}\bullet\\\bullet\\\circ\end{smallmatrix}\qquad \mathrm{T_I}\begin{smallmatrix}\circ&\bullet\\\bullet&\circ\\\bullet&\bullet\end{smallmatrix}\overset{\nu_f,\nu_r}{\overset{\mathrm{AK_I}}{\rightleftharpoons}}\mathrm{D_I}\begin{smallmatrix}\circ\\\bullet\\\bullet\end{smallmatrix}+\mathrm{D_I}\begin{smallmatrix}\bullet\\\circ\\\bullet\end{smallmatrix}\qquad \mathrm{T_I}\begin{smallmatrix}\circ&\bullet\\\bullet&\circ\\\bullet&\circ\end{smallmatrix}\overset{\nu_f,\nu_r}{\overset{\mathrm{AK_I}}{\rightleftharpoons}}\mathrm{D_I}\begin{smallmatrix}\circ\\\bullet\\\bullet\end{smallmatrix}+\mathrm{D_I}\begin{smallmatrix}\bullet\\\circ\\\circ\end{smallmatrix}$$

$$\mathrm{T_I}\begin{smallmatrix}\circ&\circ\\\bullet&\bullet\\\bullet&\bullet\end{smallmatrix}\overset{\nu_f,\nu_r}{\overset{\mathrm{AK_I}}{\rightleftharpoons}}\mathrm{D_I}\begin{smallmatrix}\circ\\\bullet\\\bullet\end{smallmatrix}+\mathrm{D_I}\begin{smallmatrix}\circ\\\bullet\\\bullet\end{smallmatrix}\qquad \mathrm{T_I}\begin{smallmatrix}\circ&\circ\\\bullet&\bullet\\\bullet&\circ\end{smallmatrix}\overset{\nu_f,\nu_r}{\overset{\mathrm{AK_I}}{\rightleftharpoons}}\mathrm{D_I}\begin{smallmatrix}\circ\\\bullet\\\bullet\end{smallmatrix}+\mathrm{D_I}\begin{smallmatrix}\circ\\\bullet\\\circ\end{smallmatrix}\qquad \mathrm{T_I}\begin{smallmatrix}\circ&\circ\\\bullet&\circ\\\bullet&\bullet\end{smallmatrix}\overset{\nu_f,\nu_r}{\overset{\mathrm{AK_I}}{\rightleftharpoons}}\mathrm{D_I}\begin{smallmatrix}\circ\\\bullet\\\bullet\end{smallmatrix}+\mathrm{D_I}\begin{smallmatrix}\circ\\\circ\\\bullet\end{smallmatrix}\qquad \mathrm{T_I}\begin{smallmatrix}\circ&\circ\\\bullet&\circ\\\bullet&\circ\end{smallmatrix}\overset{\nu_f,\nu_r}{\overset{\mathrm{AK_I}}{\rightleftharpoons}}\mathrm{D_I}\begin{smallmatrix}\circ\\\bullet\\\bullet\end{smallmatrix}+\mathrm{D_I}\begin{smallmatrix}\circ\\\circ\\\circ\end{smallmatrix}$$

$$\mathrm{T_I}\begin{smallmatrix}\circ&\bullet\\\bullet&\bullet\\\circ&\bullet\end{smallmatrix}\overset{\nu_f,\nu_r}{\overset{\mathrm{AK_I}}{\rightleftharpoons}}\mathrm{D_I}\begin{smallmatrix}\circ\\\bullet\\\circ\end{smallmatrix}+\mathrm{D_I}\begin{smallmatrix}\bullet\\\bullet\\\bullet\end{smallmatrix}\qquad \mathrm{T_I}\begin{smallmatrix}\circ&\bullet\\\bullet&\bullet\\\circ&\circ\end{smallmatrix}\overset{\nu_f,\nu_r}{\overset{\mathrm{AK_I}}{\rightleftharpoons}}\mathrm{D_I}\begin{smallmatrix}\circ\\\bullet\\\circ\end{smallmatrix}+\mathrm{D_I}\begin{smallmatrix}\bullet\\\bullet\\\circ\end{smallmatrix}\qquad \mathrm{T_I}\begin{smallmatrix}\circ&\bullet\\\bullet&\circ\\\circ&\bullet\end{smallmatrix}\overset{\nu_f,\nu_r}{\overset{\mathrm{AK_I}}{\rightleftharpoons}}\mathrm{D_I}\begin{smallmatrix}\circ\\\bullet\\\circ\end{smallmatrix}+\mathrm{D_I}\begin{smallmatrix}\bullet\\\circ\\\bullet\end{smallmatrix}\qquad \mathrm{T_I}\begin{smallmatrix}\circ&\bullet\\\bullet&\circ\\\circ&\circ\end{smallmatrix}\overset{\nu_f,\nu_r}{\overset{\mathrm{AK_I}}{\rightleftharpoons}}\mathrm{D_I}\begin{smallmatrix}\circ\\\bullet\\\circ\end{smallmatrix}+\mathrm{D_I}\begin{smallmatrix}\bullet\\\circ\\\circ\end{smallmatrix}$$

$$\mathrm{T_I}\begin{smallmatrix}\circ&\circ\\\bullet&\bullet\\\circ&\bullet\end{smallmatrix}\overset{\nu_f,\nu_r}{\overset{\mathrm{AK_I}}{\rightleftharpoons}}\mathrm{D_I}\begin{smallmatrix}\circ\\\bullet\\\circ\end{smallmatrix}+\mathrm{D_I}\begin{smallmatrix}\circ\\\bullet\\\bullet\end{smallmatrix}\qquad \mathrm{T_I}\begin{smallmatrix}\circ&\circ\\\bullet&\bullet\\\circ&\circ\end{smallmatrix}\overset{\nu_f,\nu_r}{\overset{\mathrm{AK_I}}{\rightleftharpoons}}\mathrm{D_I}\begin{smallmatrix}\circ\\\bullet\\\circ\end{smallmatrix}+\mathrm{D_I}\begin{smallmatrix}\circ\\\bullet\\\circ\end{smallmatrix}\qquad \mathrm{T_I}\begin{smallmatrix}\circ&\circ\\\bullet&\circ\\\circ&\bullet\end{smallmatrix}\overset{\nu_f,\nu_r}{\overset{\mathrm{AK_I}}{\rightleftharpoons}}\mathrm{D_I}\begin{smallmatrix}\circ\\\bullet\\\circ\end{smallmatrix}+\mathrm{D_I}\begin{smallmatrix}\circ\\\circ\\\bullet\end{smallmatrix}\qquad \mathrm{T_I}\begin{smallmatrix}\circ&\circ\\\bullet&\circ\\\circ&\circ\end{smallmatrix}\overset{\nu_f,\nu_r}{\overset{\mathrm{AK_I}}{\rightleftharpoons}}\mathrm{D_I}\begin{smallmatrix}\circ\\\bullet\\\circ\end{smallmatrix}+\mathrm{D_I}\begin{smallmatrix}\circ\\\circ\\\circ\end{smallmatrix}$$

$$\mathrm{T_I}\begin{smallmatrix}\circ&\bullet\\\circ&\bullet\\\bullet&\bullet\end{smallmatrix}\overset{\nu_f,\nu_r}{\overset{\mathrm{AK_I}}{\rightleftharpoons}}\mathrm{D_I}\begin{smallmatrix}\circ\\\circ\\\bullet\end{smallmatrix}+\mathrm{D_I}\begin{smallmatrix}\bullet\\\bullet\\\bullet\end{smallmatrix}\qquad \mathrm{T_I}\begin{smallmatrix}\circ&\bullet\\\circ&\bullet\\\bullet&\circ\end{smallmatrix}\overset{\nu_f,\nu_r}{\overset{\mathrm{AK_I}}{\rightleftharpoons}}\mathrm{D_I}\begin{smallmatrix}\circ\\\circ\\\bullet\end{smallmatrix}+\mathrm{D_I}\begin{smallmatrix}\bullet\\\bullet\\\circ\end{smallmatrix}\qquad \mathrm{T_I}\begin{smallmatrix}\circ&\bullet\\\circ&\circ\\\bullet&\bullet\end{smallmatrix}\overset{\nu_f,\nu_r}{\overset{\mathrm{AK_I}}{\rightleftharpoons}}\mathrm{D_I}\begin{smallmatrix}\circ\\\circ\\\bullet\end{smallmatrix}+\mathrm{D_I}\begin{smallmatrix}\bullet\\\circ\\\bullet\end{smallmatrix}\qquad \mathrm{T_I}\begin{smallmatrix}\circ&\bullet\\\circ&\circ\\\bullet&\circ\end{smallmatrix}\overset{\nu_f,\nu_r}{\overset{\mathrm{AK_I}}{\rightleftharpoons}}\mathrm{D_I}\begin{smallmatrix}\circ\\\circ\\\bullet\end{smallmatrix}+\mathrm{D_I}\begin{smallmatrix}\bullet\\\circ\\\circ\end{smallmatrix}$$

$$\mathrm{T_I}\begin{smallmatrix}\circ&\circ\\\circ&\bullet\\\bullet&\bullet\end{smallmatrix}\overset{\nu_f,\nu_r}{\overset{\mathrm{AK_I}}{\rightleftharpoons}}\mathrm{D_I}\begin{smallmatrix}\circ\\\circ\\\bullet\end{smallmatrix}+\mathrm{D_I}\begin{smallmatrix}\circ\\\bullet\\\bullet\end{smallmatrix}\qquad \mathrm{T_I}\begin{smallmatrix}\circ&\circ\\\circ&\bullet\\\bullet&\circ\end{smallmatrix}\overset{\nu_f,\nu_r}{\overset{\mathrm{AK_I}}{\rightleftharpoons}}\mathrm{D_I}\begin{smallmatrix}\circ\\\circ\\\bullet\end{smallmatrix}+\mathrm{D_I}\begin{smallmatrix}\circ\\\bullet\\\circ\end{smallmatrix}\qquad \mathrm{T_I}\begin{smallmatrix}\circ&\circ\\\circ&\circ\\\bullet&\bullet\end{smallmatrix}\overset{\nu_f,\nu_r}{\overset{\mathrm{AK_I}}{\rightleftharpoons}}\mathrm{D_I}\begin{smallmatrix}\circ\\\circ\\\bullet\end{smallmatrix}+\mathrm{D_I}\begin{smallmatrix}\circ\\\circ\\\bullet\end{smallmatrix}\qquad \mathrm{T_I}\begin{smallmatrix}\circ&\circ\\\circ&\circ\\\bullet&\circ\end{smallmatrix}\overset{\nu_f,\nu_r}{\overset{\mathrm{AK_I}}{\rightleftharpoons}}\mathrm{D_I}\begin{smallmatrix}\circ\\\circ\\\bullet\end{smallmatrix}+\mathrm{D_I}\begin{smallmatrix}\circ\\\circ\\\circ\end{smallmatrix}$$

$$\mathrm{T_I}\begin{smallmatrix}\circ&\bullet\\\circ&\bullet\\\circ&\bullet\end{smallmatrix}\overset{\nu_f,\nu_r}{\overset{\mathrm{AK_I}}{\rightleftharpoons}}\mathrm{D_I}\begin{smallmatrix}\circ\\\circ\\\circ\end{smallmatrix}+\mathrm{D_I}\begin{smallmatrix}\bullet\\\bullet\\\bullet\end{smallmatrix}\qquad \mathrm{T_I}\begin{smallmatrix}\circ&\bullet\\\circ&\bullet\\\circ&\circ\end{smallmatrix}\overset{\nu_f,\nu_r}{\overset{\mathrm{AK_I}}{\rightleftharpoons}}\mathrm{D_I}\begin{smallmatrix}\circ\\\circ\\\circ\end{smallmatrix}+\mathrm{D_I}\begin{smallmatrix}\bullet\\\bullet\\\circ\end{smallmatrix}\qquad \mathrm{T_I}\begin{smallmatrix}\circ&\bullet\\\circ&\circ\\\circ&\bullet\end{smallmatrix}\overset{\nu_f,\nu_r}{\overset{\mathrm{AK_I}}{\rightleftharpoons}}\mathrm{D_I}\begin{smallmatrix}\circ\\\circ\\\circ\end{smallmatrix}+\mathrm{D_I}\begin{smallmatrix}\bullet\\\circ\\\bullet\end{smallmatrix}\qquad \mathrm{T_I}\begin{smallmatrix}\circ&\bullet\\\circ&\circ\\\circ&\circ\end{smallmatrix}\overset{\nu_f,\nu_r}{\overset{\mathrm{AK_I}}{\rightleftharpoons}}\mathrm{D_I}\begin{smallmatrix}\circ\\\circ\\\circ\end{smallmatrix}+\mathrm{D_I}\begin{smallmatrix}\bullet\\\circ\\\circ\end{smallmatrix}$$

$$\mathrm{T_I}\begin{smallmatrix}\circ&\circ\\\circ&\bullet\\\circ&\bullet\end{smallmatrix}\overset{\nu_f,\nu_r}{\overset{\mathrm{AK_I}}{\rightleftharpoons}}\mathrm{D_I}\begin{smallmatrix}\circ\\\circ\\\circ\end{smallmatrix}+\mathrm{D_I}\begin{smallmatrix}\circ\\\bullet\\\bullet\end{smallmatrix}\qquad \mathrm{T_I}\begin{smallmatrix}\circ&\circ\\\circ&\bullet\\\circ&\circ\end{smallmatrix}\overset{\nu_f,\nu_r}{\overset{\mathrm{AK_I}}{\rightleftharpoons}}\mathrm{D_I}\begin{smallmatrix}\circ\\\circ\\\circ\end{smallmatrix}+\mathrm{D_I}\begin{smallmatrix}\circ\\\bullet\\\circ\end{smallmatrix}\qquad \mathrm{T_I}\begin{smallmatrix}\circ&\circ\\\circ&\circ\\\circ&\bullet\end{smallmatrix}\overset{\nu_f,\nu_r}{\overset{\mathrm{AK_I}}{\rightleftharpoons}}\mathrm{D_I}\begin{smallmatrix}\circ\\\circ\\\circ\end{smallmatrix}+\mathrm{D_I}\begin{smallmatrix}\circ\\\circ\\\bullet\end{smallmatrix}\qquad \mathrm{T_I}\begin{smallmatrix}\circ&\circ\\\circ&\circ\\\circ&\circ\end{smallmatrix}\overset{\nu_f,\nu_r}{\overset{\mathrm{AK_I}}{\rightleftharpoons}}\mathrm{D_I}\begin{smallmatrix}\circ\\\circ\\\circ\end{smallmatrix}+\mathrm{D_I}\begin{smallmatrix}\circ\\\circ\\\circ\end{smallmatrix}$$

2.2 Kinetic equations for isotopomers

$$\frac{d\mathrm{D_E}\begin{smallmatrix}\circ\\\circ\\\circ\end{smallmatrix}}{dt}=\underset{\mathrm{ASE}}{\nu_f}\Big(\big(\mathrm{T_E}\begin{smallmatrix}\circ&\bullet\\\circ&\bullet\\\circ&\bullet\end{smallmatrix}+\mathrm{T_E}\begin{smallmatrix}\circ&\bullet\\\circ&\bullet\\\circ&\circ\end{smallmatrix}+\mathrm{T_E}\begin{smallmatrix}\circ&\bullet\\\circ&\circ\\\circ&\bullet\end{smallmatrix}+\mathrm{T_E}\begin{smallmatrix}\circ&\bullet\\\circ&\circ\\\circ&\circ\end{smallmatrix}+\mathrm{T_E}\begin{smallmatrix}\circ&\circ\\\circ&\bullet\\\circ&\bullet\end{smallmatrix}+\mathrm{T_E}\begin{smallmatrix}\circ&\circ\\\circ&\bullet\\\circ&\circ\end{smallmatrix}+\mathrm{T_E}\begin{smallmatrix}\circ&\circ\\\circ&\circ\\\circ&\bullet\end{smallmatrix}+\mathrm{T_E}\begin{smallmatrix}\circ&\circ\\\circ&\circ\\\circ&\circ\end{smallmatrix}\big)\big(\mathrm{W_E}^{\bullet}+\mathrm{W_E}^{\circ}\big)\Big)+\underset{\mathrm{ADPEO}}{\nu_r}\big(\mathrm{D_O}\begin{smallmatrix}\circ\\\circ\\\circ\end{smallmatrix}\big)-\underset{\mathrm{ADPEO}}{\nu_f}\big(\mathrm{D_E}\begin{smallmatrix}\circ\\\circ\\\circ\end{smallmatrix}\big)-\underset{\mathrm{ASE}}{\nu_r}\Big(\big(\mathrm{P_E}\begin{smallmatrix}\bullet\\\bullet\\\bullet\\\bullet\end{smallmatrix}+\mathrm{P_E}\begin{smallmatrix}\bullet\\\bullet\\\bullet\\\circ\end{smallmatrix}+\mathrm{P_E}\begin{smallmatrix}\bullet\\\bullet\\\circ\\\bullet\end{smallmatrix}+\mathrm{P_E}\begin{smallmatrix}\bullet\\\bullet\\\circ\\\circ\end{smallmatrix}+\mathrm{P_E}\begin{smallmatrix}\bullet\\\circ\\\bullet\\\bullet\end{smallmatrix}+\mathrm{P_E}\begin{smallmatrix}\bullet\\\circ\\\bullet\\\circ\end{smallmatrix}+\mathrm{P_E}\begin{smallmatrix}\bullet\\\circ\\\circ\\\bullet\end{smallmatrix}+\mathrm{P_E}\begin{smallmatrix}\bullet\\\circ\\\circ\\\circ\end{smallmatrix}+\mathrm{P_E}\begin{smallmatrix}\circ\\\bullet\\\bullet\\\bullet\end{smallmatrix}+\mathrm{P_E}\begin{smallmatrix}\circ\\\bullet\\\bullet\\\circ\end{smallmatrix}+\mathrm{P_E}\begin{smallmatrix}\circ\\\bullet\\\circ\\\bullet\end{smallmatrix}+\mathrm{P_E}\begin{smallmatrix}\circ\\\bullet\\\circ\\\circ\end{smallmatrix}+\mathrm{P_E}\begin{smallmatrix}\circ\\\circ\\\bullet\\\bullet\end{smallmatrix}+\mathrm{P_E}\begin{smallmatrix}\circ\\\circ\\\bullet\\\circ\end{smallmatrix}+\mathrm{P_E}\begin{smallmatrix}\circ\\\circ\\\circ\\\bullet\end{smallmatrix}+\mathrm{P_E}\begin{smallmatrix}\circ\\\circ\\\circ\\\circ\end{smallmatrix}\big)\mathrm{D_E}\begin{smallmatrix}\circ\\\circ\\\circ\end{smallmatrix}\Big)$$

$$\frac{d\mathrm{D_E}\begin{smallmatrix}\circ\\\circ\\\bullet\end{smallmatrix}}{dt}=\underset{\mathrm{ASE}}{\nu_f}\Big(\big(\mathrm{T_E}\begin{smallmatrix}\circ&\bullet\\\circ&\bullet\\\bullet&\bullet\end{smallmatrix}+\mathrm{T_E}\begin{smallmatrix}\circ&\bullet\\\circ&\bullet\\\bullet&\circ\end{smallmatrix}+\mathrm{T_E}\begin{smallmatrix}\circ&\bullet\\\circ&\circ\\\bullet&\bullet\end{smallmatrix}+\mathrm{T_E}\begin{smallmatrix}\circ&\bullet\\\circ&\circ\\\bullet&\circ\end{smallmatrix}+\mathrm{T_E}\begin{smallmatrix}\circ&\circ\\\circ&\bullet\\\bullet&\bullet\end{smallmatrix}+\mathrm{T_E}\begin{smallmatrix}\circ&\circ\\\circ&\bullet\\\bullet&\circ\end{smallmatrix}+\mathrm{T_E}\begin{smallmatrix}\circ&\circ\\\circ&\circ\\\bullet&\bullet\end{smallmatrix}+\mathrm{T_E}\begin{smallmatrix}\circ&\circ\\\circ&\circ\\\bullet&\circ\end{smallmatrix}\big)\big(\mathrm{W_E}^{\bullet}+\mathrm{W_E}^{\circ}\big)\Big)+\underset{\mathrm{ADPEO}}{\nu_r}\big(\mathrm{D_O}\begin{smallmatrix}\circ\\\circ\\\bullet\end{smallmatrix}\big)-\underset{\mathrm{ADPEO}}{\nu_f}\big(\mathrm{D_E}\begin{smallmatrix}\circ\\\circ\\\bullet\end{smallmatrix}\big)-\underset{\mathrm{ASE}}{\nu_r}\Big(\big(\mathrm{P_E}\begin{smallmatrix}\bullet\\\bullet\\\bullet\\\bullet\end{smallmatrix}+\mathrm{P_E}\begin{smallmatrix}\bullet\\\bullet\\\bullet\\\circ\end{smallmatrix}+\mathrm{P_E}\begin{smallmatrix}\bullet\\\bullet\\\circ\\\bullet\end{smallmatrix}+\mathrm{P_E}\begin{smallmatrix}\bullet\\\bullet\\\circ\\\circ\end{smallmatrix}+\mathrm{P_E}\begin{smallmatrix}\bullet\\\circ\\\bullet\\\bullet\end{smallmatrix}+\mathrm{P_E}\begin{smallmatrix}\bullet\\\circ\\\bullet\\\circ\end{smallmatrix}+\mathrm{P_E}\begin{smallmatrix}\bullet\\\circ\\\circ\\\bullet\end{smallmatrix}+\mathrm{P_E}\begin{smallmatrix}\bullet\\\circ\\\circ\\\circ\end{smallmatrix}+\mathrm{P_E}\begin{smallmatrix}\circ\\\bullet\\\bullet\\\bullet\end{smallmatrix}+\mathrm{P_E}\begin{smallmatrix}\circ\\\bullet\\\bullet\\\circ\end{smallmatrix}+\mathrm{P_E}\begin{smallmatrix}\circ\\\bullet\\\circ\\\bullet\end{smallmatrix}+\mathrm{P_E}\begin{smallmatrix}\circ\\\bullet\\\circ\\\circ\end{smallmatrix}+\mathrm{P_E}\begin{smallmatrix}\circ\\\circ\\\bullet\\\bullet\end{smallmatrix}+\mathrm{P_E}\begin{smallmatrix}\circ\\\circ\\\bullet\\\circ\end{smallmatrix}+\mathrm{P_E}\begin{smallmatrix}\circ\\\circ\\\circ\\\bullet\end{smallmatrix}+\mathrm{P_E}\begin{smallmatrix}\circ\\\circ\\\circ\\\circ\end{smallmatrix}\big)\mathrm{D_E}\begin{smallmatrix}\circ\\\circ\\\bullet\end{smallmatrix}\Big)$$

$$\frac{d\mathrm{D_E}^{[illegible]}}{dt} = \nu^f_{\mathrm{ASE}}\big((\mathrm{T_E}^{[illegible]} + \cdots + \mathrm{T_E}^{[illegible]})(\mathrm{W_E}^{\bullet} + \mathrm{W_E}^{\circ})\big) + \nu^r_{\mathrm{ADPEO}}(\mathrm{D_O}^{[illegible]}) - \nu^f_{\mathrm{ADPEO}}(\mathrm{D_E}^{[illegible]}) - \nu^r_{\mathrm{ASE}}\big((\mathrm{P_E}^{[illegible]} + \cdots + \mathrm{P_E}^{[illegible]})\mathrm{D_E}^{[illegible]}\big)$$

$$\frac{d\mathrm{D_E}^{[illegible]}}{dt} = \nu^f_{\mathrm{ASE}}\big((\mathrm{T_E}^{[illegible]} + \cdots + \mathrm{T_E}^{[illegible]})(\mathrm{W_E}^{\bullet} + \mathrm{W_E}^{\circ})\big) + \nu^r_{\mathrm{ADPEO}}(\mathrm{D_O}^{[illegible]}) - \nu^f_{\mathrm{ADPEO}}(\mathrm{D_E}^{[illegible]}) - \nu^r_{\mathrm{ASE}}\big((\mathrm{P_E}^{[illegible]} + \cdots + \mathrm{P_E}^{[illegible]})\mathrm{D_E}^{[illegible]}\big)$$

$$\frac{d\mathrm{D_E}^{[illegible]}}{dt} = \nu^f_{\mathrm{ASE}}\big((\mathrm{T_E}^{[illegible]} + \cdots + \mathrm{T_E}^{[illegible]})(\mathrm{W_E}^{\bullet} + \mathrm{W_E}^{\circ})\big) + \nu^r_{\mathrm{ADPEO}}(\mathrm{D_O}^{[illegible]}) - \nu^f_{\mathrm{ADPEO}}(\mathrm{D_E}^{[illegible]}) - \nu^r_{\mathrm{ASE}}\big((\mathrm{P_E}^{[illegible]} + \cdots + \mathrm{P_E}^{[illegible]})\mathrm{D_E}^{[illegible]}\big)$$

$$\frac{d\mathrm{D_E}^{[illegible]}}{dt} = \nu^f_{\mathrm{ASE}}\big((\mathrm{T_E}^{[illegible]} + \cdots + \mathrm{T_E}^{[illegible]})(\mathrm{W_E}^{\bullet} + \mathrm{W_E}^{\circ})\big) + \nu^r_{\mathrm{ADPEO}}(\mathrm{D_O}^{[illegible]}) - \nu^f_{\mathrm{ADPEO}}(\mathrm{D_E}^{[illegible]}) - \nu^r_{\mathrm{ASE}}\big((\mathrm{P_E}^{[illegible]} + \cdots + \mathrm{P_E}^{[illegible]})\mathrm{D_E}^{[illegible]}\big)$$

$$\frac{d\mathrm{D_E}^{[illegible]}}{dt} = \nu^f_{\mathrm{ASE}}\big((\mathrm{T_E}^{[illegible]} + \cdots + \mathrm{T_E}^{[illegible]})(\mathrm{W_E}^{\bullet} + \mathrm{W_E}^{\circ})\big) + \nu^r_{\mathrm{ADPEO}}(\mathrm{D_O}^{[illegible]}) - \nu^f_{\mathrm{ADPEO}}(\mathrm{D_E}^{[illegible]}) - \nu^r_{\mathrm{ASE}}\big((\mathrm{P_E}^{[illegible]} + \cdots + \mathrm{P_E}^{[illegible]})\mathrm{D_E}^{[illegible]}\big)$$

$$\frac{d\mathrm{D_E}^{[illegible]}}{dt} = \nu^f_{\mathrm{ASE}}\big((\mathrm{T_E}^{[illegible]} + \cdots + \mathrm{T_E}^{[illegible]})(\mathrm{W_E}^{\bullet} + \mathrm{W_E}^{\circ})\big) + \nu^r_{\mathrm{ADPEO}}(\mathrm{D_O}^{[illegible]}) - \nu^f_{\mathrm{ADPEO}}(\mathrm{D_E}^{[illegible]}) - \nu^r_{\mathrm{ASE}}\big((\mathrm{P_E}^{[illegible]} + \cdots + \mathrm{P_E}^{[illegible]})\mathrm{D_E}^{[illegible]}\big)$$

$$\frac{d\mathrm{D_I}^{[illegible]}}{dt} = \nu^f_{\mathrm{ADPOI}}(\mathrm{D_O}^{[illegible]}) + \nu^f_{\mathrm{AKI}}(2\mathrm{T_I}^{[illegible]} + \cdots + \mathrm{T_I}^{[illegible]}) + \nu^f_{\mathrm{CKI}}(\mathrm{T_I}^{[illegible]} + \cdots + \mathrm{T_I}^{[illegible]}) + \nu^r_{\mathrm{ADPIM}}(\mathrm{D_M}^{[illegible]}) - \nu^f_{\mathrm{ADPIM}}(\mathrm{D_I}^{[illegible]}) - \nu^r_{\mathrm{ADPOI}}(\mathrm{D_I}^{[illegible]}) - \nu^r_{\mathrm{AKI}}\big(2(\mathrm{D_I}^{[illegible]} + \cdots + \mathrm{D_I}^{[illegible]})\mathrm{D_I}^{[illegible]}\big) - \nu^r_{\mathrm{CKI}}\big((\mathrm{C_I}^{[illegible]} + \cdots + \mathrm{C_I}^{[illegible]})\mathrm{D_I}^{[illegible]}\big)$$

$$\frac{d\mathrm{D_I}^{[illegible]}}{dt} = \nu^f_{\mathrm{ADPOI}}(\mathrm{D_O}^{[illegible]}) + \nu^f_{\mathrm{AKI}}(2\mathrm{T_I}^{[illegible]} + \cdots + \mathrm{T_I}^{[illegible]}) + \nu^f_{\mathrm{CKI}}(\mathrm{T_I}^{[illegible]} + \cdots + \mathrm{T_I}^{[illegible]}) + \nu^r_{\mathrm{ADPIM}}(\mathrm{D_M}^{[illegible]}) - \nu^f_{\mathrm{ADPIM}}(\mathrm{D_I}^{[illegible]}) - \nu^r_{\mathrm{ADPOI}}(\mathrm{D_I}^{[illegible]}) - \nu^r_{\mathrm{AKI}}\big(2(\mathrm{D_I}^{[illegible]} + \cdots + \mathrm{D_I}^{[illegible]})\mathrm{D_I}^{[illegible]}\big) - \nu^r_{\mathrm{CKI}}\big((\mathrm{C_I}^{[illegible]} + \cdots + \mathrm{C_I}^{[illegible]})\mathrm{D_I}^{[illegible]}\big)$$

$$\frac{d\mathrm{D_I}^{[illegible]}}{dt} = \nu^f_{\mathrm{ADPOI}}(\mathrm{D_O}^{[illegible]}) + \nu^f_{\mathrm{AKI}}(2\mathrm{T_I}^{[illegible]} + \cdots + \mathrm{T_I}^{[illegible]}) + \nu^f_{\mathrm{CKI}}(\mathrm{T_I}^{[illegible]} + \cdots + \mathrm{T_I}^{[illegible]}) + \nu^r_{\mathrm{ADPIM}}(\mathrm{D_M}^{[illegible]}) - \nu^f_{\mathrm{ADPIM}}(\mathrm{D_I}^{[illegible]}) - \nu^r_{\mathrm{ADPOI}}(\mathrm{D_I}^{[illegible]}) - \nu^r_{\mathrm{AKI}}\big(2(\mathrm{D_I}^{[illegible]} + \cdots + \mathrm{D_I}^{[illegible]})\mathrm{D_I}^{[illegible]}\big) - \nu^r_{\mathrm{CKI}}\big((\mathrm{C_I}^{[illegible]} + \cdots + \mathrm{C_I}^{[illegible]})\mathrm{D_I}^{[illegible]}\big)$$

$$\frac{d\mathrm{D_I}^{[illegible]}}{dt} = \nu^f_{\mathrm{ADPOI}}(\mathrm{D_O}^{[illegible]}) + \nu^f_{\mathrm{AKI}}(2\mathrm{T_I}^{[illegible]} + \cdots + \mathrm{T_I}^{[illegible]}) + \nu^f_{\mathrm{CKI}}(\mathrm{T_I}^{[illegible]} + \cdots + \mathrm{T_I}^{[illegible]}) + \nu^r_{\mathrm{ADPIM}}(\mathrm{D_M}^{[illegible]}) - \nu^f_{\mathrm{ADPIM}}(\mathrm{D_I}^{[illegible]}) - \nu^r_{\mathrm{ADPOI}}(\mathrm{D_I}^{[illegible]}) - \nu^r_{\mathrm{AKI}}\big(2(\mathrm{D_I}^{[illegible]} + \cdots + \mathrm{D_I}^{[illegible]})\mathrm{D_I}^{[illegible]}\big) - \nu^r_{\mathrm{CKI}}\big((\mathrm{C_I}^{[illegible]} + \cdots + \mathrm{C_I}^{[illegible]})\mathrm{D_I}^{[illegible]}\big)$$

$$\frac{d\mathrm{D_I}^{[illegible]}}{dt} = \nu^f_{\mathrm{ADPOI}}(\mathrm{D_O}^{[illegible]}) + \nu^f_{\mathrm{AKI}}(2\mathrm{T_I}^{[illegible]} + \cdots + \mathrm{T_I}^{[illegible]}) + \nu^f_{\mathrm{CKI}}(\mathrm{T_I}^{[illegible]} + \cdots + \mathrm{T_I}^{[illegible]}) + \nu^r_{\mathrm{ADPIM}}(\mathrm{D_M}^{[illegible]}) - \nu^f_{\mathrm{ADPIM}}(\mathrm{D_I}^{[illegible]}) - \nu^r_{\mathrm{ADPOI}}(\mathrm{D_I}^{[illegible]}) - \nu^r_{\mathrm{AKI}}\big(2(\mathrm{D_I}^{[illegible]} + \cdots + \mathrm{D_I}^{[illegible]})\mathrm{D_I}^{[illegible]}\big) - \nu^r_{\mathrm{CKI}}\big((\mathrm{C_I}^{[illegible]} + \cdots + \mathrm{C_I}^{[illegible]})\mathrm{D_I}^{[illegible]}\big)$$

$$\frac{d\mathrm{D_I}^{[illegible]}}{dt} = \nu^f_{\mathrm{ADPOI}}(\mathrm{D_O}^{[illegible]}) + \nu^f_{\mathrm{AKI}}(2\mathrm{T_I}^{[illegible]} + \cdots + \mathrm{T_I}^{[illegible]}) + \nu^f_{\mathrm{CKI}}(\mathrm{T_I}^{[illegible]} + \cdots + \mathrm{T_I}^{[illegible]}) + \nu^r_{\mathrm{ADPIM}}(\mathrm{D_M}^{[illegible]}) - \nu^f_{\mathrm{ADPIM}}(\mathrm{D_I}^{[illegible]}) - \nu^r_{\mathrm{ADPOI}}(\mathrm{D_I}^{[illegible]}) - \nu^r_{\mathrm{AKI}}\big(2(\mathrm{D_I}^{[illegible]} + \mathrm{D_I}^{[illegible]} + \mathrm{D_I}^{[illegible]} + \mathrm{D_I}^{[illegible]} +$$

$$\mathrm{D}_{\mathrm{I}}^{\substack{\circ\\\bullet\\\bullet}} + \mathrm{D}_{\mathrm{I}}^{\substack{\circ\\\bullet\\\circ}} + \mathrm{D}_{\mathrm{I}}^{\substack{\circ\\\circ\\\bullet}} + \mathrm{D}_{\mathrm{I}}^{\substack{\circ\\\circ\\\circ}})\mathrm{D}_{\mathrm{I}}^{\substack{\bullet\\\circ\\\bullet}}) - \nu^{r}_{\mathrm{CKi}}((\mathrm{C}_{\mathrm{I}}^{\substack{\bullet\\\bullet\\\bullet}} + \mathrm{C}_{\mathrm{I}}^{\substack{\bullet\\\bullet\\\circ}} + \mathrm{C}_{\mathrm{I}}^{\substack{\bullet\\\circ\\\bullet}} + \mathrm{C}_{\mathrm{I}}^{\substack{\bullet\\\circ\\\circ}} + \mathrm{C}_{\mathrm{I}}^{\substack{\circ\\\bullet\\\bullet}} + \mathrm{C}_{\mathrm{I}}^{\substack{\circ\\\bullet\\\circ}} + \mathrm{C}_{\mathrm{I}}^{\substack{\circ\\\circ\\\bullet}} + \mathrm{C}_{\mathrm{I}}^{\substack{\circ\\\circ\\\circ}})\mathrm{D}_{\mathrm{I}}^{\substack{\bullet\\\circ\\\bullet}})$$

$$\frac{d\mathrm{D}_{\mathrm{I}}^{\substack{\bullet\\\bullet\\\circ}}}{dt} = \nu^{f}_{\mathrm{ADPoi}}(\mathrm{D}_{\mathrm{O}}^{\substack{\bullet\\\bullet\\\circ}}) + \nu^{f}_{\mathrm{AKi}}(2\mathrm{T}_{\mathrm{I}}^{\substack{\bullet\bullet\\\bullet\bullet\\\circ\circ}} + \mathrm{T}_{\mathrm{I}}^{\substack{\bullet\bullet\\\bullet\bullet\\\bullet\circ}} + \mathrm{T}_{\mathrm{I}}^{\substack{\bullet\bullet\\\bullet\bullet\\\circ\bullet}} + \mathrm{T}_{\mathrm{I}}^{\substack{\bullet\bullet\\\bullet\circ\\\circ\bullet}} + \mathrm{T}_{\mathrm{I}}^{\substack{\bullet\bullet\\\bullet\circ\\\circ\circ}} + \mathrm{T}_{\mathrm{I}}^{\substack{\bullet\circ\\\bullet\bullet\\\circ\bullet}} + \mathrm{T}_{\mathrm{I}}^{\substack{\bullet\circ\\\bullet\bullet\\\circ\circ}} + \mathrm{T}_{\mathrm{I}}^{\substack{\bullet\circ\\\bullet\circ\\\circ\bullet}} + \mathrm{T}_{\mathrm{I}}^{\substack{\bullet\circ\\\bullet\circ\\\circ\circ}} + \mathrm{T}_{\mathrm{I}}^{\substack{\bullet\bullet\\\circ\bullet\\\bullet\circ}} + \mathrm{T}_{\mathrm{I}}^{\substack{\bullet\bullet\\\circ\bullet\\\circ\circ}} + \mathrm{T}_{\mathrm{I}}^{\substack{\circ\bullet\\\bullet\bullet\\\bullet\circ}} + \mathrm{T}_{\mathrm{I}}^{\substack{\circ\bullet\\\bullet\bullet\\\circ\circ}} + \mathrm{T}_{\mathrm{I}}^{\substack{\circ\bullet\\\circ\bullet\\\bullet\circ}} + \mathrm{T}_{\mathrm{I}}^{\substack{\circ\bullet\\\circ\bullet\\\circ\circ}}) + \nu^{f}_{\mathrm{CKi}}(\mathrm{T}_{\mathrm{I}}^{\substack{\bullet\bullet\\\bullet\bullet\\\circ\bullet}} + \mathrm{T}_{\mathrm{I}}^{\substack{\bullet\bullet\\\bullet\bullet\\\circ\circ}} + \mathrm{T}_{\mathrm{I}}^{\substack{\bullet\bullet\\\bullet\circ\\\circ\bullet}} + \mathrm{T}_{\mathrm{I}}^{\substack{\bullet\bullet\\\bullet\circ\\\circ\circ}} + \mathrm{T}_{\mathrm{I}}^{\substack{\bullet\circ\\\bullet\bullet\\\circ\bullet}} + \mathrm{T}_{\mathrm{I}}^{\substack{\bullet\circ\\\bullet\bullet\\\circ\circ}} + \mathrm{T}_{\mathrm{I}}^{\substack{\bullet\circ\\\bullet\circ\\\circ\bullet}} + \mathrm{T}_{\mathrm{I}}^{\substack{\bullet\circ\\\bullet\circ\\\circ\circ}}) + \nu^{r}_{\mathrm{ADPim}}(\mathrm{D}_{\mathrm{M}}^{\substack{\bullet\\\bullet\\\circ}}) - \nu^{f}_{\mathrm{ADPim}}(\mathrm{D}_{\mathrm{I}}^{\substack{\bullet\\\bullet\\\circ}}) - \nu^{r}_{\mathrm{ADPoi}}(\mathrm{D}_{\mathrm{I}}^{\substack{\bullet\\\bullet\\\circ}}) - \nu^{r}_{\mathrm{AKi}}(2(\mathrm{D}_{\mathrm{I}}^{\substack{\bullet\\\bullet\\\bullet}} + \mathrm{D}_{\mathrm{I}}^{\substack{\bullet\\\bullet\\\circ}} + \mathrm{D}_{\mathrm{I}}^{\substack{\bullet\\\circ\\\bullet}} + \mathrm{D}_{\mathrm{I}}^{\substack{\bullet\\\circ\\\circ}} + \mathrm{D}_{\mathrm{I}}^{\substack{\circ\\\bullet\\\bullet}} + \mathrm{D}_{\mathrm{I}}^{\substack{\circ\\\bullet\\\circ}} + \mathrm{D}_{\mathrm{I}}^{\substack{\circ\\\circ\\\bullet}} + \mathrm{D}_{\mathrm{I}}^{\substack{\circ\\\circ\\\circ}})\mathrm{D}_{\mathrm{I}}^{\substack{\bullet\\\bullet\\\circ}}) - \nu^{r}_{\mathrm{CKi}}((\mathrm{C}_{\mathrm{I}}^{\substack{\bullet\\\bullet\\\bullet}} + \mathrm{C}_{\mathrm{I}}^{\substack{\bullet\\\bullet\\\circ}} + \mathrm{C}_{\mathrm{I}}^{\substack{\bullet\\\circ\\\bullet}} + \mathrm{C}_{\mathrm{I}}^{\substack{\bullet\\\circ\\\circ}} + \mathrm{C}_{\mathrm{I}}^{\substack{\circ\\\bullet\\\bullet}} + \mathrm{C}_{\mathrm{I}}^{\substack{\circ\\\bullet\\\circ}} + \mathrm{C}_{\mathrm{I}}^{\substack{\circ\\\circ\\\bullet}} + \mathrm{C}_{\mathrm{I}}^{\substack{\circ\\\circ\\\circ}})\mathrm{D}_{\mathrm{I}}^{\substack{\bullet\\\bullet\\\circ}})$$

$$\frac{d\mathrm{D}_{\mathrm{I}}^{\substack{\bullet\\\bullet\\\bullet}}}{dt} = \nu^{f}_{\mathrm{ADPoi}}(\mathrm{D}_{\mathrm{O}}^{\substack{\bullet\\\bullet\\\bullet}}) + \nu^{f}_{\mathrm{AKi}}(2\mathrm{T}_{\mathrm{I}}^{\substack{\bullet\bullet\\\bullet\bullet\\\bullet\bullet}} + \mathrm{T}_{\mathrm{I}}^{\substack{\bullet\bullet\\\bullet\bullet\\\bullet\circ}} + \mathrm{T}_{\mathrm{I}}^{\substack{\bullet\bullet\\\bullet\circ\\\bullet\bullet}} + \mathrm{T}_{\mathrm{I}}^{\substack{\bullet\bullet\\\bullet\circ\\\bullet\circ}} + \mathrm{T}_{\mathrm{I}}^{\substack{\bullet\circ\\\bullet\bullet\\\bullet\bullet}} + \mathrm{T}_{\mathrm{I}}^{\substack{\bullet\circ\\\bullet\bullet\\\bullet\circ}} + \mathrm{T}_{\mathrm{I}}^{\substack{\bullet\circ\\\bullet\circ\\\bullet\bullet}} + \mathrm{T}_{\mathrm{I}}^{\substack{\bullet\circ\\\bullet\circ\\\bullet\circ}} + \mathrm{T}_{\mathrm{I}}^{\substack{\bullet\bullet\\\bullet\bullet\\\circ\bullet}} + \mathrm{T}_{\mathrm{I}}^{\substack{\bullet\bullet\\\circ\bullet\\\bullet\bullet}} + \mathrm{T}_{\mathrm{I}}^{\substack{\bullet\bullet\\\circ\bullet\\\circ\bullet}} + \mathrm{T}_{\mathrm{I}}^{\substack{\circ\bullet\\\bullet\bullet\\\bullet\bullet}} + \mathrm{T}_{\mathrm{I}}^{\substack{\circ\bullet\\\bullet\bullet\\\circ\bullet}} + \mathrm{T}_{\mathrm{I}}^{\substack{\circ\bullet\\\circ\bullet\\\bullet\bullet}} + \mathrm{T}_{\mathrm{I}}^{\substack{\circ\bullet\\\circ\bullet\\\circ\bullet}}) + \nu^{f}_{\mathrm{CKi}}(\mathrm{T}_{\mathrm{I}}^{\substack{\bullet\bullet\\\bullet\bullet\\\bullet\bullet}} + \mathrm{T}_{\mathrm{I}}^{\substack{\bullet\bullet\\\bullet\bullet\\\bullet\circ}} + \mathrm{T}_{\mathrm{I}}^{\substack{\bullet\bullet\\\bullet\circ\\\bullet\bullet}} + \mathrm{T}_{\mathrm{I}}^{\substack{\bullet\bullet\\\bullet\circ\\\bullet\circ}} + \mathrm{T}_{\mathrm{I}}^{\substack{\bullet\circ\\\bullet\bullet\\\bullet\bullet}} + \mathrm{T}_{\mathrm{I}}^{\substack{\bullet\circ\\\bullet\bullet\\\bullet\circ}} + \mathrm{T}_{\mathrm{I}}^{\substack{\bullet\circ\\\bullet\circ\\\bullet\bullet}} + \mathrm{T}_{\mathrm{I}}^{\substack{\bullet\circ\\\bullet\circ\\\bullet\circ}}) + \nu^{r}_{\mathrm{ADPim}}(\mathrm{D}_{\mathrm{M}}^{\substack{\bullet\\\bullet\\\bullet}}) - \nu^{f}_{\mathrm{ADPim}}(\mathrm{D}_{\mathrm{I}}^{\substack{\bullet\\\bullet\\\bullet}}) - \nu^{r}_{\mathrm{ADPoi}}(\mathrm{D}_{\mathrm{I}}^{\substack{\bullet\\\bullet\\\bullet}}) - \nu^{r}_{\mathrm{AKi}}(2(\mathrm{D}_{\mathrm{I}}^{\substack{\bullet\\\bullet\\\bullet}} + \mathrm{D}_{\mathrm{I}}^{\substack{\bullet\\\bullet\\\circ}} + \mathrm{D}_{\mathrm{I}}^{\substack{\bullet\\\circ\\\bullet}} + \mathrm{D}_{\mathrm{I}}^{\substack{\bullet\\\circ\\\circ}} + \mathrm{D}_{\mathrm{I}}^{\substack{\circ\\\bullet\\\bullet}} + \mathrm{D}_{\mathrm{I}}^{\substack{\circ\\\bullet\\\circ}} + \mathrm{D}_{\mathrm{I}}^{\substack{\circ\\\circ\\\bullet}} + \mathrm{D}_{\mathrm{I}}^{\substack{\circ\\\circ\\\circ}})\mathrm{D}_{\mathrm{I}}^{\substack{\bullet\\\bullet\\\bullet}}) - \nu^{r}_{\mathrm{CKi}}((\mathrm{C}_{\mathrm{I}}^{\substack{\bullet\\\bullet\\\bullet}} + \mathrm{C}_{\mathrm{I}}^{\substack{\bullet\\\bullet\\\circ}} + \mathrm{C}_{\mathrm{I}}^{\substack{\bullet\\\circ\\\bullet}} + \mathrm{C}_{\mathrm{I}}^{\substack{\bullet\\\circ\\\circ}} + \mathrm{C}_{\mathrm{I}}^{\substack{\circ\\\bullet\\\bullet}} + \mathrm{C}_{\mathrm{I}}^{\substack{\circ\\\bullet\\\circ}} + \mathrm{C}_{\mathrm{I}}^{\substack{\circ\\\circ\\\bullet}} + \mathrm{C}_{\mathrm{I}}^{\substack{\circ\\\circ\\\circ}})\mathrm{D}_{\mathrm{I}}^{\substack{\bullet\\\bullet\\\bullet}})$$

$$\frac{d\mathrm{D}_{\mathrm{M}}^{\substack{\circ\\\circ\\\circ}}}{dt} = \nu^{f}_{\mathrm{ADPim}}(\mathrm{D}_{\mathrm{I}}^{\substack{\circ\\\circ\\\circ}}) + \nu^{r}_{\mathrm{ADPms}}(\mathrm{D}_{\mathrm{S}}^{\substack{\circ\\\circ\\\circ}}) - \nu^{f}_{\mathrm{ADPms}}(\mathrm{D}_{\mathrm{M}}^{\substack{\circ\\\circ\\\circ}}) - \nu^{r}_{\mathrm{ADPim}}(\mathrm{D}_{\mathrm{M}}^{\substack{\circ\\\circ\\\circ}})$$

$$\frac{d\mathrm{D}_{\mathrm{M}}^{\substack{\circ\\\circ\\\bullet}}}{dt} = \nu^{f}_{\mathrm{ADPim}}(\mathrm{D}_{\mathrm{I}}^{\substack{\circ\\\circ\\\bullet}}) + \nu^{r}_{\mathrm{ADPms}}(\mathrm{D}_{\mathrm{S}}^{\substack{\circ\\\circ\\\bullet}}) - \nu^{f}_{\mathrm{ADPms}}(\mathrm{D}_{\mathrm{M}}^{\substack{\circ\\\circ\\\bullet}}) - \nu^{r}_{\mathrm{ADPim}}(\mathrm{D}_{\mathrm{M}}^{\substack{\circ\\\circ\\\bullet}})$$

$$\frac{d\mathrm{D}_{\mathrm{M}}^{\substack{\circ\\\bullet\\\circ}}}{dt} = \nu^{f}_{\mathrm{ADPim}}(\mathrm{D}_{\mathrm{I}}^{\substack{\circ\\\bullet\\\circ}}) + \nu^{r}_{\mathrm{ADPms}}(\mathrm{D}_{\mathrm{S}}^{\substack{\circ\\\bullet\\\circ}}) - \nu^{f}_{\mathrm{ADPms}}(\mathrm{D}_{\mathrm{M}}^{\substack{\circ\\\bullet\\\circ}}) - \nu^{r}_{\mathrm{ADPim}}(\mathrm{D}_{\mathrm{M}}^{\substack{\circ\\\bullet\\\circ}})$$

$$\frac{d\mathrm{D}_{\mathrm{M}}^{\substack{\circ\\\bullet\\\bullet}}}{dt} = \nu^{f}_{\mathrm{ADPim}}(\mathrm{D}_{\mathrm{I}}^{\substack{\circ\\\bullet\\\bullet}}) + \nu^{r}_{\mathrm{ADPms}}(\mathrm{D}_{\mathrm{S}}^{\substack{\circ\\\bullet\\\bullet}}) - \nu^{f}_{\mathrm{ADPms}}(\mathrm{D}_{\mathrm{M}}^{\substack{\circ\\\bullet\\\bullet}}) - \nu^{r}_{\mathrm{ADPim}}(\mathrm{D}_{\mathrm{M}}^{\substack{\circ\\\bullet\\\bullet}})$$

$$\frac{d\mathrm{D}_{\mathrm{M}}^{\substack{\bullet\\\circ\\\circ}}}{dt} = \nu^{f}_{\mathrm{ADPim}}(\mathrm{D}_{\mathrm{I}}^{\substack{\bullet\\\circ\\\circ}}) + \nu^{r}_{\mathrm{ADPms}}(\mathrm{D}_{\mathrm{S}}^{\substack{\bullet\\\circ\\\circ}}) - \nu^{f}_{\mathrm{ADPms}}(\mathrm{D}_{\mathrm{M}}^{\substack{\bullet\\\circ\\\circ}}) - \nu^{r}_{\mathrm{ADPim}}(\mathrm{D}_{\mathrm{M}}^{\substack{\bullet\\\circ\\\circ}})$$

$$\frac{d\mathrm{D}_{\mathrm{M}}^{\substack{\bullet\\\circ\\\bullet}}}{dt} = \nu^{f}_{\mathrm{ADPim}}(\mathrm{D}_{\mathrm{I}}^{\substack{\bullet\\\circ\\\bullet}}) + \nu^{r}_{\mathrm{ADPms}}(\mathrm{D}_{\mathrm{S}}^{\substack{\bullet\\\circ\\\bullet}}) - \nu^{f}_{\mathrm{ADPms}}(\mathrm{D}_{\mathrm{M}}^{\substack{\bullet\\\circ\\\bullet}}) - \nu^{r}_{\mathrm{ADPim}}(\mathrm{D}_{\mathrm{M}}^{\substack{\bullet\\\circ\\\bullet}})$$

$$\frac{d\mathrm{D}_{\mathrm{M}}^{\substack{\bullet\\\bullet\\\circ}}}{dt} = \nu^{f}_{\mathrm{ADPim}}(\mathrm{D}_{\mathrm{I}}^{\substack{\bullet\\\bullet\\\circ}}) + \nu^{r}_{\mathrm{ADPms}}(\mathrm{D}_{\mathrm{S}}^{\substack{\bullet\\\bullet\\\circ}}) - \nu^{f}_{\mathrm{ADPms}}(\mathrm{D}_{\mathrm{M}}^{\substack{\bullet\\\bullet\\\circ}}) - \nu^{r}_{\mathrm{ADPim}}(\mathrm{D}_{\mathrm{M}}^{\substack{\bullet\\\bullet\\\circ}})$$

$$\frac{d\mathrm{D}_{\mathrm{M}}^{\substack{\bullet\\\bullet\\\bullet}}}{dt} = \nu^{f}_{\mathrm{ADPim}}(\mathrm{D}_{\mathrm{I}}^{\substack{\bullet\\\bullet\\\bullet}}) + \nu^{r}_{\mathrm{ADPms}}(\mathrm{D}_{\mathrm{S}}^{\substack{\bullet\\\bullet\\\bullet}}) - \nu^{f}_{\mathrm{ADPms}}(\mathrm{D}_{\mathrm{M}}^{\substack{\bullet\\\bullet\\\bullet}}) - \nu^{r}_{\mathrm{ADPim}}(\mathrm{D}_{\mathrm{M}}^{\substack{\bullet\\\bullet\\\bullet}})$$

$$\frac{d\mathrm{D}_{\mathrm{O}}^{\substack{\circ\\\circ\\\circ}}}{dt} = \nu^{f}_{\mathrm{ADPeo}}(\mathrm{D}_{\mathrm{E}}^{\substack{\circ\\\circ\\\circ}}) + \nu^{f}_{\mathrm{AKo}}(2\mathrm{T}_{\mathrm{O}}^{\substack{\circ\circ\\\circ\circ\\\circ\circ}} + \mathrm{T}_{\mathrm{O}}^{\substack{\bullet\circ\\\bullet\circ\\\bullet\circ}} + \mathrm{T}_{\mathrm{O}}^{\substack{\bullet\circ\\\bullet\circ\\\circ\circ}} + \mathrm{T}_{\mathrm{O}}^{\substack{\bullet\circ\\\circ\circ\\\bullet\circ}} + \mathrm{T}_{\mathrm{O}}^{\substack{\bullet\circ\\\circ\circ\\\circ\circ}} + \mathrm{T}_{\mathrm{O}}^{\substack{\circ\circ\\\bullet\circ\\\bullet\circ}} + \mathrm{T}_{\mathrm{O}}^{\substack{\circ\circ\\\bullet\circ\\\circ\circ}} + \mathrm{T}_{\mathrm{O}}^{\substack{\circ\circ\\\circ\circ\\\bullet\circ}} + \mathrm{T}_{\mathrm{O}}^{\substack{\circ\bullet\\\circ\bullet\\\circ\bullet}} + \mathrm{T}_{\mathrm{O}}^{\substack{\circ\bullet\\\circ\bullet\\\circ\circ}} + \mathrm{T}_{\mathrm{O}}^{\substack{\circ\bullet\\\circ\circ\\\circ\bullet}} + \mathrm{T}_{\mathrm{O}}^{\substack{\circ\bullet\\\circ\circ\\\circ\circ}} + \mathrm{T}_{\mathrm{O}}^{\substack{\circ\circ\\\circ\bullet\\\circ\bullet}} + \mathrm{T}_{\mathrm{O}}^{\substack{\circ\circ\\\circ\bullet\\\circ\circ}} + \mathrm{T}_{\mathrm{O}}^{\substack{\circ\circ\\\circ\circ\\\circ\bullet}}) + \nu^{f}_{\mathrm{CKo}}(\mathrm{T}_{\mathrm{O}}^{\substack{\circ\bullet\\\circ\bullet\\\circ\bullet}} + \mathrm{T}_{\mathrm{O}}^{\substack{\circ\bullet\\\circ\bullet\\\circ\circ}} + \mathrm{T}_{\mathrm{O}}^{\substack{\circ\bullet\\\circ\circ\\\circ\bullet}} + \mathrm{T}_{\mathrm{O}}^{\substack{\circ\bullet\\\circ\circ\\\circ\circ}} + \mathrm{T}_{\mathrm{O}}^{\substack{\circ\circ\\\circ\bullet\\\circ\bullet}} + \mathrm{T}_{\mathrm{O}}^{\substack{\circ\circ\\\circ\bullet\\\circ\circ}} + \mathrm{T}_{\mathrm{O}}^{\substack{\circ\circ\\\circ\circ\\\circ\bullet}} + \mathrm{T}_{\mathrm{O}}^{\substack{\circ\circ\\\circ\circ\\\circ\circ}}) + \nu^{r}_{\mathrm{ADPoi}}(\mathrm{D}_{\mathrm{I}}^{\substack{\circ\\\circ\\\circ}}) - \nu^{f}_{\mathrm{ADPoi}}(\mathrm{D}_{\mathrm{O}}^{\substack{\circ\\\circ\\\circ}}) - \nu^{r}_{\mathrm{ADPeo}}(\mathrm{D}_{\mathrm{O}}^{\substack{\circ\\\circ\\\circ}}) - \nu^{r}_{\mathrm{AKo}}(2(\mathrm{D}_{\mathrm{O}}^{\substack{\bullet\\\bullet\\\bullet}} + \mathrm{D}_{\mathrm{O}}^{\substack{\bullet\\\bullet\\\circ}} + \mathrm{D}_{\mathrm{O}}^{\substack{\bullet\\\circ\\\bullet}} + \mathrm{D}_{\mathrm{O}}^{\substack{\bullet\\\circ\\\circ}} + \mathrm{D}_{\mathrm{O}}^{\substack{\circ\\\bullet\\\bullet}} + \mathrm{D}_{\mathrm{O}}^{\substack{\circ\\\bullet\\\circ}} + \mathrm{D}_{\mathrm{O}}^{\substack{\circ\\\circ\\\bullet}} + \mathrm{D}_{\mathrm{O}}^{\substack{\circ\\\circ\\\circ}})\mathrm{D}_{\mathrm{O}}^{\substack{\circ\\\circ\\\circ}}) - \nu^{r}_{\mathrm{CKo}}((\mathrm{C}_{\mathrm{O}}^{\substack{\bullet\\\bullet\\\bullet}} + \mathrm{C}_{\mathrm{O}}^{\substack{\bullet\\\bullet\\\circ}} + \mathrm{C}_{\mathrm{O}}^{\substack{\bullet\\\circ\\\bullet}} + \mathrm{C}_{\mathrm{O}}^{\substack{\bullet\\\circ\\\circ}} + \mathrm{C}_{\mathrm{O}}^{\substack{\circ\\\bullet\\\bullet}} + \mathrm{C}_{\mathrm{O}}^{\substack{\circ\\\bullet\\\circ}} + \mathrm{C}_{\mathrm{O}}^{\substack{\circ\\\circ\\\bullet}} + \mathrm{C}_{\mathrm{O}}^{\substack{\circ\\\circ\\\circ}})\mathrm{D}_{\mathrm{O}}^{\substack{\circ\\\circ\\\circ}})$$

$$\frac{d\mathrm{D}_{\mathrm{O}}^{\substack{\circ\\\circ\\\bullet}}}{dt} = \nu^{f}_{\mathrm{ADPeo}}(\mathrm{D}_{\mathrm{E}}^{\substack{\circ\\\circ\\\bullet}}) + \nu^{f}_{\mathrm{AKo}}(2\mathrm{T}_{\mathrm{O}}^{\substack{\circ\circ\\\circ\circ\\\bullet\bullet}} + \mathrm{T}_{\mathrm{O}}^{\substack{\bullet\circ\\\bullet\circ\\\bullet\bullet}} + \mathrm{T}_{\mathrm{O}}^{\substack{\bullet\circ\\\bullet\circ\\\circ\bullet}} + \mathrm{T}_{\mathrm{O}}^{\substack{\bullet\circ\\\circ\circ\\\bullet\bullet}} + \mathrm{T}_{\mathrm{O}}^{\substack{\bullet\circ\\\circ\circ\\\circ\bullet}} + \mathrm{T}_{\mathrm{O}}^{\substack{\circ\circ\\\bullet\circ\\\bullet\bullet}} + \mathrm{T}_{\mathrm{O}}^{\substack{\circ\circ\\\bullet\circ\\\circ\bullet}} + \mathrm{T}_{\mathrm{O}}^{\substack{\circ\bullet\\\circ\bullet\\\bullet\bullet}} + \mathrm{T}_{\mathrm{O}}^{\substack{\circ\bullet\\\circ\bullet\\\bullet\circ}} + \mathrm{T}_{\mathrm{O}}^{\substack{\circ\bullet\\\circ\circ\\\bullet\bullet}} + \mathrm{T}_{\mathrm{O}}^{\substack{\circ\bullet\\\circ\circ\\\bullet\circ}} + \mathrm{T}_{\mathrm{O}}^{\substack{\circ\circ\\\circ\bullet\\\bullet\bullet}} + \mathrm{T}_{\mathrm{O}}^{\substack{\circ\circ\\\circ\bullet\\\bullet\circ}} + \mathrm{T}_{\mathrm{O}}^{\substack{\circ\circ\\\circ\circ\\\bullet\circ}} + \mathrm{T}_{\mathrm{O}}^{\substack{\circ\circ\\\circ\circ\\\circ\bullet}}) + \nu^{f}_{\mathrm{CKo}}(\mathrm{T}_{\mathrm{O}}^{\substack{\circ\bullet\\\circ\bullet\\\bullet\bullet}} + \mathrm{T}_{\mathrm{O}}^{\substack{\circ\bullet\\\circ\bullet\\\bullet\circ}} + \mathrm{T}_{\mathrm{O}}^{\substack{\circ\bullet\\\circ\circ\\\bullet\bullet}} + \mathrm{T}_{\mathrm{O}}^{\substack{\circ\bullet\\\circ\circ\\\bullet\circ}} + \mathrm{T}_{\mathrm{O}}^{\substack{\circ\circ\\\circ\bullet\\\bullet\bullet}} + \mathrm{T}_{\mathrm{O}}^{\substack{\circ\circ\\\circ\bullet\\\bullet\circ}} + \mathrm{T}_{\mathrm{O}}^{\substack{\circ\circ\\\circ\circ\\\bullet\bullet}} + \mathrm{T}_{\mathrm{O}}^{\substack{\circ\circ\\\circ\circ\\\bullet\circ}}) + \nu^{r}_{\mathrm{ADPoi}}(\mathrm{D}_{\mathrm{I}}^{\substack{\circ\\\circ\\\bullet}}) - \nu^{f}_{\mathrm{ADPoi}}(\mathrm{D}_{\mathrm{O}}^{\substack{\circ\\\circ\\\bullet}}) - \nu^{r}_{\mathrm{ADPeo}}(\mathrm{D}_{\mathrm{O}}^{\substack{\circ\\\circ\\\bullet}}) - \nu^{r}_{\mathrm{AKo}}(2(\mathrm{D}_{\mathrm{O}}^{\substack{\bullet\\\bullet\\\bullet}} + \mathrm{D}_{\mathrm{O}}^{\substack{\bullet\\\bullet\\\circ}} + \mathrm{D}_{\mathrm{O}}^{\substack{\bullet\\\circ\\\bullet}} + \mathrm{D}_{\mathrm{O}}^{\substack{\bullet\\\circ\\\circ}} + \mathrm{D}_{\mathrm{O}}^{\substack{\circ\\\bullet\\\bullet}} + \mathrm{D}_{\mathrm{O}}^{\substack{\circ\\\bullet\\\circ}} + \mathrm{D}_{\mathrm{O}}^{\substack{\circ\\\circ\\\bullet}} + \mathrm{D}_{\mathrm{O}}^{\substack{\circ\\\circ\\\circ}})\mathrm{D}_{\mathrm{O}}^{\substack{\circ\\\circ\\\bullet}}) - \nu^{r}_{\mathrm{CKo}}((\mathrm{C}_{\mathrm{O}}^{\substack{\bullet\\\bullet\\\bullet}} + \mathrm{C}_{\mathrm{O}}^{\substack{\bullet\\\bullet\\\circ}} + \mathrm{C}_{\mathrm{O}}^{\substack{\bullet\\\circ\\\bullet}} + \mathrm{C}_{\mathrm{O}}^{\substack{\bullet\\\circ\\\circ}} + \mathrm{C}_{\mathrm{O}}^{\substack{\circ\\\bullet\\\bullet}} + \mathrm{C}_{\mathrm{O}}^{\substack{\circ\\\bullet\\\circ}} + \mathrm{C}_{\mathrm{O}}^{\substack{\circ\\\circ\\\bullet}} + \mathrm{C}_{\mathrm{O}}^{\substack{\circ\\\circ\\\circ}})\mathrm{D}_{\mathrm{O}}^{\substack{\circ\\\circ\\\bullet}})$$

$$\frac{d\mathrm{D}_{\mathrm{O}}^{\substack{\circ\\\bullet\\\circ}}}{dt} = \nu^{f}_{\mathrm{ADPeo}}(\mathrm{D}_{\mathrm{E}}^{\substack{\circ\\\bullet\\\circ}}) + \nu^{f}_{\mathrm{AKo}}(2\mathrm{T}_{\mathrm{O}}^{\substack{\circ\circ\\\bullet\bullet\\\circ\circ}} + \mathrm{T}_{\mathrm{O}}^{\substack{\bullet\circ\\\bullet\bullet\\\bullet\circ}} + \mathrm{T}_{\mathrm{O}}^{\substack{\bullet\circ\\\bullet\bullet\\\circ\circ}} + \mathrm{T}_{\mathrm{O}}^{\substack{\bullet\circ\\\circ\bullet\\\bullet\circ}} + \mathrm{T}_{\mathrm{O}}^{\substack{\bullet\circ\\\circ\bullet\\\circ\circ}} + \mathrm{T}_{\mathrm{O}}^{\substack{\circ\circ\\\bullet\bullet\\\bullet\circ}} + \mathrm{T}_{\mathrm{O}}^{\substack{\circ\bullet\\\bullet\bullet\\\circ\bullet}} + \mathrm{T}_{\mathrm{O}}^{\substack{\circ\bullet\\\bullet\bullet\\\circ\circ}} + \mathrm{T}_{\mathrm{O}}^{\substack{\circ\bullet\\\bullet\circ\\\circ\bullet}} + \mathrm{T}_{\mathrm{O}}^{\substack{\circ\bullet\\\bullet\circ\\\circ\circ}} + \mathrm{T}_{\mathrm{O}}^{\substack{\circ\circ\\\bullet\bullet\\\circ\bullet}} + \mathrm{T}_{\mathrm{O}}^{\substack{\circ\circ\\\bullet\circ\\\circ\bullet}} + \mathrm{T}_{\mathrm{O}}^{\substack{\circ\circ\\\bullet\circ\\\circ\circ}} + \mathrm{T}_{\mathrm{O}}^{\substack{\circ\circ\\\circ\bullet\\\bullet\circ}} + \mathrm{T}_{\mathrm{O}}^{\substack{\circ\circ\\\circ\bullet\\\circ\circ}}) + \nu^{f}_{\mathrm{CKo}}(\mathrm{T}_{\mathrm{O}}^{\substack{\circ\bullet\\\bullet\bullet\\\circ\bullet}} + \mathrm{T}_{\mathrm{O}}^{\substack{\circ\bullet\\\bullet\bullet\\\circ\circ}} + \mathrm{T}_{\mathrm{O}}^{\substack{\circ\bullet\\\bullet\circ\\\circ\bullet}} + \mathrm{T}_{\mathrm{O}}^{\substack{\circ\bullet\\\bullet\circ\\\circ\circ}} + \mathrm{T}_{\mathrm{O}}^{\substack{\circ\circ\\\bullet\bullet\\\circ\bullet}} + \mathrm{T}_{\mathrm{O}}^{\substack{\circ\circ\\\bullet\bullet\\\circ\circ}} + \mathrm{T}_{\mathrm{O}}^{\substack{\circ\circ\\\bullet\circ\\\circ\bullet}} + \mathrm{T}_{\mathrm{O}}^{\substack{\circ\circ\\\bullet\circ\\\circ\circ}}) + \nu^{r}_{\mathrm{ADPoi}}(\mathrm{D}_{\mathrm{I}}^{\substack{\circ\\\bullet\\\circ}}) - \nu^{f}_{\mathrm{ADPoi}}(\mathrm{D}_{\mathrm{O}}^{\substack{\circ\\\bullet\\\circ}}) - \nu^{r}_{\mathrm{ADPeo}}(\mathrm{D}_{\mathrm{O}}^{\substack{\circ\\\bullet\\\circ}}) - \nu^{r}_{\mathrm{AKo}}(2(\mathrm{D}_{\mathrm{O}}^{\substack{\bullet\\\bullet\\\bullet}} + \mathrm{D}_{\mathrm{O}}^{\substack{\bullet\\\bullet\\\circ}} + \mathrm{D}_{\mathrm{O}}^{\substack{\bullet\\\circ\\\bullet}} + \mathrm{D}_{\mathrm{O}}^{\substack{\bullet\\\circ\\\circ}} + \mathrm{D}_{\mathrm{O}}^{\substack{\circ\\\bullet\\\bullet}} + \mathrm{D}_{\mathrm{O}}^{\substack{\circ\\\bullet\\\circ}} + \mathrm{D}_{\mathrm{O}}^{\substack{\circ\\\circ\\\bullet}} + \mathrm{D}_{\mathrm{O}}^{\substack{\circ\\\circ\\\circ}})\mathrm{D}_{\mathrm{O}}^{\substack{\circ\\\bullet\\\circ}}) - \nu^{r}_{\mathrm{CKo}}((\mathrm{C}_{\mathrm{O}}^{\substack{\bullet\\\bullet\\\bullet}} + \mathrm{C}_{\mathrm{O}}^{\substack{\bullet\\\bullet\\\circ}} + \mathrm{C}_{\mathrm{O}}^{\substack{\bullet\\\circ\\\bullet}} + \mathrm{C}_{\mathrm{O}}^{\substack{\bullet\\\circ\\\circ}} + \mathrm{C}_{\mathrm{O}}^{\substack{\circ\\\bullet\\\bullet}} + \mathrm{C}_{\mathrm{O}}^{\substack{\circ\\\bullet\\\circ}} + \mathrm{C}_{\mathrm{O}}^{\substack{\circ\\\circ\\\bullet}} + \mathrm{C}_{\mathrm{O}}^{\substack{\circ\\\circ\\\circ}})\mathrm{D}_{\mathrm{O}}^{\substack{\circ\\\bullet\\\circ}})$$

$$\frac{d\mathrm{D}_{\mathrm{O}}^{\substack{\circ\\\bullet\\\bullet}}}{dt} = \nu^{f}_{\mathrm{ADPeo}}(\mathrm{D}_{\mathrm{E}}^{\substack{\circ\\\bullet\\\bullet}}) + \nu^{f}_{\mathrm{AKo}}(2\mathrm{T}_{\mathrm{O}}^{\substack{\circ\circ\\\bullet\bullet\\\bullet\bullet}} + \mathrm{T}_{\mathrm{O}}^{\substack{\bullet\circ\\\bullet\bullet\\\bullet\bullet}} + \mathrm{T}_{\mathrm{O}}^{\substack{\bullet\circ\\\bullet\bullet\\\circ\bullet}} + \mathrm{T}_{\mathrm{O}}^{\substack{\bullet\circ\\\circ\bullet\\\bullet\bullet}} + \mathrm{T}_{\mathrm{O}}^{\substack{\bullet\circ\\\circ\bullet\\\circ\bullet}} + \mathrm{T}_{\mathrm{O}}^{\substack{\circ\bullet\\\bullet\bullet\\\bullet\bullet}} + \mathrm{T}_{\mathrm{O}}^{\substack{\circ\bullet\\\bullet\bullet\\\bullet\circ}} + \mathrm{T}_{\mathrm{O}}^{\substack{\circ\bullet\\\bullet\circ\\\bullet\bullet}} + \mathrm{T}_{\mathrm{O}}^{\substack{\circ\bullet\\\bullet\circ\\\bullet\circ}} + \mathrm{T}_{\mathrm{O}}^{\substack{\circ\circ\\\bullet\bullet\\\bullet\circ}} + \mathrm{T}_{\mathrm{O}}^{\substack{\circ\circ\\\bullet\circ\\\bullet\bullet}} + \mathrm{T}_{\mathrm{O}}^{\substack{\circ\circ\\\bullet\circ\\\bullet\circ}} + \mathrm{T}_{\mathrm{O}}^{\substack{\circ\circ\\\bullet\bullet\\\circ\bullet}} + \mathrm{T}_{\mathrm{O}}^{\substack{\circ\circ\\\circ\bullet\\\bullet\bullet}} + \mathrm{T}_{\mathrm{O}}^{\substack{\circ\circ\\\circ\bullet\\\circ\bullet}}) + \nu^{f}_{\mathrm{CKo}}(\mathrm{T}_{\mathrm{O}}^{\substack{\circ\bullet\\\bullet\bullet\\\bullet\bullet}} + \mathrm{T}_{\mathrm{O}}^{\substack{\circ\bullet\\\bullet\bullet\\\bullet\circ}} + \mathrm{T}_{\mathrm{O}}^{\substack{\circ\bullet\\\bullet\circ\\\bullet\bullet}} + \mathrm{T}_{\mathrm{O}}^{\substack{\circ\bullet\\\bullet\circ\\\bullet\circ}} + \mathrm{T}_{\mathrm{O}}^{\substack{\circ\circ\\\bullet\bullet\\\bullet\bullet}} + \mathrm{T}_{\mathrm{O}}^{\substack{\circ\circ\\\bullet\bullet\\\bullet\circ}} + \mathrm{T}_{\mathrm{O}}^{\substack{\circ\circ\\\bullet\circ\\\bullet\bullet}} + \mathrm{T}_{\mathrm{O}}^{\substack{\circ\circ\\\bullet\circ\\\bullet\circ}}) + \nu^{r}_{\mathrm{ADPoi}}(\mathrm{D}_{\mathrm{I}}^{\substack{\circ\\\bullet\\\bullet}}) - \nu^{f}_{\mathrm{ADPoi}}(\mathrm{D}_{\mathrm{O}}^{\substack{\circ\\\bullet\\\bullet}}) - \nu^{r}_{\mathrm{ADPeo}}(\mathrm{D}_{\mathrm{O}}^{\substack{\circ\\\bullet\\\bullet}}) - \nu^{r}_{\mathrm{AKo}}(2(\mathrm{D}_{\mathrm{O}}^{\substack{\bullet\\\bullet\\\bullet}} + \mathrm{D}_{\mathrm{O}}^{\substack{\bullet\\\bullet\\\circ}} + \mathrm{D}_{\mathrm{O}}^{\substack{\bullet\\\circ\\\bullet}} + \mathrm{D}_{\mathrm{O}}^{\substack{\bullet\\\circ\\\circ}} + \mathrm{D}_{\mathrm{O}}^{\substack{\circ\\\bullet\\\bullet}} + \mathrm{D}_{\mathrm{O}}^{\substack{\circ\\\bullet\\\circ}} + \mathrm{D}_{\mathrm{O}}^{\substack{\circ\\\circ\\\bullet}} + \mathrm{D}_{\mathrm{O}}^{\substack{\circ\\\circ\\\circ}})\mathrm{D}_{\mathrm{O}}^{\substack{\circ\\\bullet\\\bullet}}) - \nu^{r}_{\mathrm{CKo}}((\mathrm{C}_{\mathrm{O}}^{\substack{\bullet\\\bullet\\\bullet}} + \mathrm{C}_{\mathrm{O}}^{\substack{\bullet\\\bullet\\\circ}} + \mathrm{C}_{\mathrm{O}}^{\substack{\bullet\\\circ\\\bullet}} + \mathrm{C}_{\mathrm{O}}^{\substack{\bullet\\\circ\\\circ}} + \mathrm{C}_{\mathrm{O}}^{\substack{\circ\\\bullet\\\bullet}} + \mathrm{C}_{\mathrm{O}}^{\substack{\circ\\\bullet\\\circ}} + \mathrm{C}_{\mathrm{O}}^{\substack{\circ\\\circ\\\bullet}} + \mathrm{C}_{\mathrm{O}}^{\substack{\circ\\\circ\\\circ}})\mathrm{D}_{\mathrm{O}}^{\substack{\circ\\\bullet\\\bullet}})$$

$$\frac{d\mathrm{D}_{\mathrm{O}}^{\substack{\bullet\\\circ\\\circ}}}{dt} = \nu^{f}_{\mathrm{ADPeo}}(\mathrm{D}_{\mathrm{E}}^{\substack{\bullet\\\circ\\\circ}}) + \nu^{f}_{\mathrm{AKo}}(2\mathrm{T}_{\mathrm{O}}^{\substack{\bullet\bullet\\\circ\circ\\\circ\circ}} + \mathrm{T}_{\mathrm{O}}^{\substack{\bullet\bullet\\\bullet\circ\\\bullet\circ}} + \mathrm{T}_{\mathrm{O}}^{\substack{\bullet\bullet\\\bullet\circ\\\circ\circ}} + \mathrm{T}_{\mathrm{O}}^{\substack{\bullet\bullet\\\circ\circ\\\bullet\circ}} + \mathrm{T}_{\mathrm{O}}^{\substack{\bullet\bullet\\\circ\bullet\\\circ\bullet}} + \mathrm{T}_{\mathrm{O}}^{\substack{\bullet\bullet\\\circ\bullet\\\circ\circ}} + \mathrm{T}_{\mathrm{O}}^{\substack{\bullet\bullet\\\circ\circ\\\circ\bullet}} + \mathrm{T}_{\mathrm{O}}^{\substack{\bullet\circ\\\circ\bullet\\\circ\bullet}} + \mathrm{T}_{\mathrm{O}}^{\substack{\bullet\circ\\\circ\bullet\\\circ\circ}} + \mathrm{T}_{\mathrm{O}}^{\substack{\bullet\circ\\\circ\circ\\\circ\bullet}} + \mathrm{T}_{\mathrm{O}}^{\substack{\bullet\circ\\\circ\circ\\\circ\circ}} + \mathrm{T}_{\mathrm{O}}^{\substack{\circ\bullet\\\bullet\circ\\\bullet\circ}} + \mathrm{T}_{\mathrm{O}}^{\substack{\circ\bullet\\\bullet\circ\\\circ\circ}} + \mathrm{T}_{\mathrm{O}}^{\substack{\circ\bullet\\\circ\circ\\\bullet\circ}} +$$

$$\mathrm{T_O}^{\substack{\circ\bullet\\\circ\circ\\\circ\circ}}\Big) + \underset{\mathrm{CKo}}{\nu_f}\Big(\mathrm{T_O}^{\substack{\bullet\bullet\\\circ\bullet\\\circ\bullet}} + \mathrm{T_O}^{\substack{\bullet\bullet\\\circ\bullet\\\circ\circ}} + \mathrm{T_O}^{\substack{\bullet\bullet\\\circ\circ\\\circ\bullet}} + \mathrm{T_O}^{\substack{\bullet\bullet\\\circ\circ\\\circ\circ}} + \mathrm{T_O}^{\substack{\bullet\circ\\\circ\bullet\\\circ\bullet}} + \mathrm{T_O}^{\substack{\bullet\circ\\\circ\bullet\\\circ\circ}} + \mathrm{T_O}^{\substack{\bullet\circ\\\circ\circ\\\circ\bullet}} + \mathrm{T_O}^{\substack{\bullet\circ\\\circ\circ\\\circ\circ}}\Big) + \underset{\mathrm{ADPoI}}{\nu_r}\Big(\mathrm{D_I}^{\substack{\bullet\\\circ\\\circ}}\Big) - \underset{\mathrm{ADPoI}}{\nu_f}\Big(\mathrm{D_O}^{\substack{\bullet\\\circ\\\circ}}\Big) - \underset{\mathrm{ADPeO}}{\nu_r}\Big(\mathrm{D_O}^{\substack{\bullet\\\circ\\\circ}}\Big) - \underset{\mathrm{AKo}}{\nu_r}\Big(2\Big(\mathrm{D_O}^{\substack{\bullet\\\bullet\\\bullet}} + \mathrm{D_O}^{\substack{\bullet\\\bullet\\\circ}} + \mathrm{D_O}^{\substack{\bullet\\\circ\\\bullet}} + \mathrm{D_O}^{\substack{\bullet\\\circ\\\circ}} + \mathrm{D_O}^{\substack{\circ\\\bullet\\\bullet}} + \mathrm{D_O}^{\substack{\circ\\\bullet\\\circ}} + \mathrm{D_O}^{\substack{\circ\\\circ\\\bullet}} + \mathrm{D_O}^{\substack{\circ\\\circ\\\circ}}\Big)\mathrm{D_O}^{\substack{\bullet\\\circ\\\circ}}\Big) - \underset{\mathrm{CKo}}{\nu_r}\Big(\Big(\mathrm{C_O}^{\substack{\bullet\\\bullet\\\bullet}} + \mathrm{C_O}^{\substack{\bullet\\\bullet\\\circ}} + \mathrm{C_O}^{\substack{\bullet\\\circ\\\bullet}} + \mathrm{C_O}^{\substack{\bullet\\\circ\\\circ}} + \mathrm{C_O}^{\substack{\circ\\\bullet\\\bullet}} + \mathrm{C_O}^{\substack{\circ\\\bullet\\\circ}} + \mathrm{C_O}^{\substack{\circ\\\circ\\\bullet}} + \mathrm{C_O}^{\substack{\circ\\\circ\\\circ}}\Big)\mathrm{D_O}^{\substack{\bullet\\\circ\\\circ}}\Big)$$

$$\frac{d\mathrm{D_O}^{\substack{\bullet\\\circ\\\bullet}}}{dt} = \underset{\mathrm{ADPeO}}{\nu_f}\Big(\mathrm{D_E}^{\substack{\bullet\\\circ\\\bullet}}\Big) + \underset{\mathrm{AKo}}{\nu_f}\Big(2\mathrm{T_O}^{\substack{\bullet\bullet\\\circ\circ\\\bullet\bullet}} + \mathrm{T_O}^{\substack{\bullet\bullet\\\bullet\circ\\\bullet\bullet}} + \mathrm{T_O}^{\substack{\bullet\bullet\\\bullet\circ\\\circ\bullet}} + \mathrm{T_O}^{\substack{\bullet\bullet\\\circ\bullet\\\bullet\bullet}} + \mathrm{T_O}^{\substack{\bullet\bullet\\\circ\bullet\\\bullet\circ}} + \mathrm{T_O}^{\substack{\bullet\bullet\\\circ\circ\\\bullet\circ}} + \mathrm{T_O}^{\substack{\bullet\circ\\\circ\bullet\\\bullet\bullet}} + \mathrm{T_O}^{\substack{\bullet\circ\\\circ\bullet\\\bullet\circ}} + \mathrm{T_O}^{\substack{\bullet\circ\\\circ\circ\\\bullet\bullet}} + \mathrm{T_O}^{\substack{\bullet\circ\\\circ\circ\\\bullet\circ}} + \mathrm{T_O}^{\substack{\bullet\bullet\\\circ\circ\\\circ\bullet}} + \mathrm{T_O}^{\substack{\circ\bullet\\\bullet\circ\\\bullet\bullet}} + \mathrm{T_O}^{\substack{\circ\bullet\\\bullet\circ\\\circ\bullet}} + \mathrm{T_O}^{\substack{\circ\bullet\\\circ\circ\\\bullet\bullet}} + \mathrm{T_O}^{\substack{\circ\bullet\\\circ\circ\\\circ\bullet}}\Big) + \underset{\mathrm{CKo}}{\nu_f}\Big(\mathrm{T_O}^{\substack{\bullet\bullet\\\circ\bullet\\\bullet\bullet}} + \mathrm{T_O}^{\substack{\bullet\bullet\\\circ\bullet\\\bullet\circ}} + \mathrm{T_O}^{\substack{\bullet\bullet\\\circ\circ\\\bullet\bullet}} + \mathrm{T_O}^{\substack{\bullet\bullet\\\circ\circ\\\bullet\circ}} + \mathrm{T_O}^{\substack{\bullet\circ\\\circ\bullet\\\bullet\bullet}} + \mathrm{T_O}^{\substack{\bullet\circ\\\circ\bullet\\\bullet\circ}} + \mathrm{T_O}^{\substack{\bullet\circ\\\circ\circ\\\bullet\bullet}} + \mathrm{T_O}^{\substack{\bullet\circ\\\circ\circ\\\bullet\circ}}\Big) + \underset{\mathrm{ADPoI}}{\nu_r}\Big(\mathrm{D_I}^{\substack{\bullet\\\circ\\\bullet}}\Big) - \underset{\mathrm{ADPoI}}{\nu_f}\Big(\mathrm{D_O}^{\substack{\bullet\\\circ\\\bullet}}\Big) - \underset{\mathrm{ADPeO}}{\nu_r}\Big(\mathrm{D_O}^{\substack{\bullet\\\circ\\\bullet}}\Big) - \underset{\mathrm{AKo}}{\nu_r}\Big(2\Big(\mathrm{D_O}^{\substack{\bullet\\\bullet\\\bullet}} + \mathrm{D_O}^{\substack{\bullet\\\bullet\\\circ}} + \mathrm{D_O}^{\substack{\bullet\\\circ\\\bullet}} + \mathrm{D_O}^{\substack{\bullet\\\circ\\\circ}} + \mathrm{D_O}^{\substack{\circ\\\bullet\\\bullet}} + \mathrm{D_O}^{\substack{\circ\\\bullet\\\circ}} + \mathrm{D_O}^{\substack{\circ\\\circ\\\bullet}} + \mathrm{D_O}^{\substack{\circ\\\circ\\\circ}}\Big)\mathrm{D_O}^{\substack{\bullet\\\circ\\\bullet}}\Big) - \underset{\mathrm{CKo}}{\nu_r}\Big(\Big(\mathrm{C_O}^{\substack{\bullet\\\bullet\\\bullet}} + \mathrm{C_O}^{\substack{\bullet\\\bullet\\\circ}} + \mathrm{C_O}^{\substack{\bullet\\\circ\\\bullet}} + \mathrm{C_O}^{\substack{\bullet\\\circ\\\circ}} + \mathrm{C_O}^{\substack{\circ\\\bullet\\\bullet}} + \mathrm{C_O}^{\substack{\circ\\\bullet\\\circ}} + \mathrm{C_O}^{\substack{\circ\\\circ\\\bullet}} + \mathrm{C_O}^{\substack{\circ\\\circ\\\circ}}\Big)\mathrm{D_O}^{\substack{\bullet\\\circ\\\bullet}}\Big)$$

$$\frac{d\mathrm{D_O}^{\substack{\bullet\\\bullet\\\circ}}}{dt} = \underset{\mathrm{ADPeO}}{\nu_f}\Big(\mathrm{D_E}^{\substack{\bullet\\\bullet\\\circ}}\Big) + \underset{\mathrm{AKo}}{\nu_f}\Big(2\mathrm{T_O}^{\substack{\bullet\bullet\\\bullet\bullet\\\circ\circ}} + \mathrm{T_O}^{\substack{\bullet\bullet\\\bullet\bullet\\\bullet\circ}} + \mathrm{T_O}^{\substack{\bullet\bullet\\\bullet\bullet\\\circ\bullet}} + \mathrm{T_O}^{\substack{\bullet\bullet\\\bullet\circ\\\circ\bullet}} + \mathrm{T_O}^{\substack{\bullet\bullet\\\bullet\circ\\\circ\circ}} + \mathrm{T_O}^{\substack{\bullet\circ\\\bullet\bullet\\\circ\bullet}} + \mathrm{T_O}^{\substack{\bullet\circ\\\bullet\bullet\\\circ\circ}} + \mathrm{T_O}^{\substack{\bullet\circ\\\bullet\circ\\\circ\bullet}} + \mathrm{T_O}^{\substack{\bullet\circ\\\bullet\circ\\\circ\circ}} + \mathrm{T_O}^{\substack{\bullet\bullet\\\circ\bullet\\\bullet\circ}} + \mathrm{T_O}^{\substack{\bullet\bullet\\\circ\bullet\\\circ\circ}} + \mathrm{T_O}^{\substack{\circ\bullet\\\bullet\bullet\\\bullet\circ}} + \mathrm{T_O}^{\substack{\circ\bullet\\\bullet\bullet\\\circ\circ}} + \mathrm{T_O}^{\substack{\circ\bullet\\\circ\bullet\\\bullet\circ}} + \mathrm{T_O}^{\substack{\circ\bullet\\\circ\bullet\\\circ\circ}}\Big) + \underset{\mathrm{CKo}}{\nu_f}\Big(\mathrm{T_O}^{\substack{\bullet\bullet\\\bullet\bullet\\\circ\bullet}} + \mathrm{T_O}^{\substack{\bullet\bullet\\\bullet\bullet\\\circ\circ}} + \mathrm{T_O}^{\substack{\bullet\bullet\\\bullet\circ\\\circ\bullet}} + \mathrm{T_O}^{\substack{\bullet\bullet\\\bullet\circ\\\circ\circ}} + \mathrm{T_O}^{\substack{\bullet\circ\\\bullet\bullet\\\circ\bullet}} + \mathrm{T_O}^{\substack{\bullet\circ\\\bullet\bullet\\\circ\circ}} + \mathrm{T_O}^{\substack{\bullet\circ\\\bullet\circ\\\circ\bullet}} + \mathrm{T_O}^{\substack{\bullet\circ\\\bullet\circ\\\circ\circ}}\Big) + \underset{\mathrm{ADPoI}}{\nu_r}\Big(\mathrm{D_I}^{\substack{\bullet\\\bullet\\\circ}}\Big) - \underset{\mathrm{ADPoI}}{\nu_f}\Big(\mathrm{D_O}^{\substack{\bullet\\\bullet\\\circ}}\Big) - \underset{\mathrm{ADPeO}}{\nu_r}\Big(\mathrm{D_O}^{\substack{\bullet\\\bullet\\\circ}}\Big) - \underset{\mathrm{AKo}}{\nu_r}\Big(2\Big(\mathrm{D_O}^{\substack{\bullet\\\bullet\\\bullet}} + \mathrm{D_O}^{\substack{\bullet\\\bullet\\\circ}} + \mathrm{D_O}^{\substack{\bullet\\\circ\\\bullet}} + \mathrm{D_O}^{\substack{\bullet\\\circ\\\circ}} + \mathrm{D_O}^{\substack{\circ\\\bullet\\\bullet}} + \mathrm{D_O}^{\substack{\circ\\\bullet\\\circ}} + \mathrm{D_O}^{\substack{\circ\\\circ\\\bullet}} + \mathrm{D_O}^{\substack{\circ\\\circ\\\circ}}\Big)\mathrm{D_O}^{\substack{\bullet\\\bullet\\\circ}}\Big) - \underset{\mathrm{CKo}}{\nu_r}\Big(\Big(\mathrm{C_O}^{\substack{\bullet\\\bullet\\\bullet}} + \mathrm{C_O}^{\substack{\bullet\\\bullet\\\circ}} + \mathrm{C_O}^{\substack{\bullet\\\circ\\\bullet}} + \mathrm{C_O}^{\substack{\bullet\\\circ\\\circ}} + \mathrm{C_O}^{\substack{\circ\\\bullet\\\bullet}} + \mathrm{C_O}^{\substack{\circ\\\bullet\\\circ}} + \mathrm{C_O}^{\substack{\circ\\\circ\\\bullet}} + \mathrm{C_O}^{\substack{\circ\\\circ\\\circ}}\Big)\mathrm{D_O}^{\substack{\bullet\\\bullet\\\circ}}\Big)$$

$$\frac{d\mathrm{D_O}^{\substack{\bullet\\\bullet\\\bullet}}}{dt} = \underset{\mathrm{ADPeO}}{\nu_f}\Big(\mathrm{D_E}^{\substack{\bullet\\\bullet\\\bullet}}\Big) + \underset{\mathrm{AKo}}{\nu_f}\Big(2\mathrm{T_O}^{\substack{\bullet\bullet\\\bullet\bullet\\\bullet\bullet}} + \mathrm{T_O}^{\substack{\bullet\bullet\\\bullet\bullet\\\bullet\circ}} + \mathrm{T_O}^{\substack{\bullet\bullet\\\bullet\circ\\\bullet\bullet}} + \mathrm{T_O}^{\substack{\bullet\bullet\\\bullet\circ\\\bullet\circ}} + \mathrm{T_O}^{\substack{\bullet\circ\\\bullet\bullet\\\bullet\bullet}} + \mathrm{T_O}^{\substack{\bullet\circ\\\bullet\bullet\\\bullet\circ}} + \mathrm{T_O}^{\substack{\bullet\circ\\\bullet\circ\\\bullet\bullet}} + \mathrm{T_O}^{\substack{\bullet\circ\\\bullet\circ\\\bullet\circ}} + \mathrm{T_O}^{\substack{\bullet\bullet\\\bullet\bullet\\\circ\bullet}} + \mathrm{T_O}^{\substack{\bullet\bullet\\\circ\bullet\\\bullet\bullet}} + \mathrm{T_O}^{\substack{\bullet\bullet\\\circ\bullet\\\circ\bullet}} + \mathrm{T_O}^{\substack{\circ\bullet\\\bullet\bullet\\\bullet\bullet}} + \mathrm{T_O}^{\substack{\circ\bullet\\\bullet\bullet\\\circ\bullet}} + \mathrm{T_O}^{\substack{\circ\bullet\\\circ\bullet\\\bullet\bullet}} + \mathrm{T_O}^{\substack{\circ\bullet\\\circ\bullet\\\circ\bullet}}\Big) + \underset{\mathrm{CKo}}{\nu_f}\Big(\mathrm{T_O}^{\substack{\bullet\bullet\\\bullet\bullet\\\bullet\bullet}} + \mathrm{T_O}^{\substack{\bullet\bullet\\\bullet\bullet\\\bullet\circ}} + \mathrm{T_O}^{\substack{\bullet\bullet\\\bullet\circ\\\bullet\bullet}} + \mathrm{T_O}^{\substack{\bullet\bullet\\\bullet\circ\\\bullet\circ}} + \mathrm{T_O}^{\substack{\bullet\circ\\\bullet\bullet\\\bullet\bullet}} + \mathrm{T_O}^{\substack{\bullet\circ\\\bullet\bullet\\\bullet\circ}} + \mathrm{T_O}^{\substack{\bullet\circ\\\bullet\circ\\\bullet\bullet}} + \mathrm{T_O}^{\substack{\bullet\circ\\\bullet\circ\\\bullet\circ}}\Big) + \underset{\mathrm{ADPoI}}{\nu_r}\Big(\mathrm{D_I}^{\substack{\bullet\\\bullet\\\bullet}}\Big) - \underset{\mathrm{ADPoI}}{\nu_f}\Big(\mathrm{D_O}^{\substack{\bullet\\\bullet\\\bullet}}\Big) - \underset{\mathrm{ADPeO}}{\nu_r}\Big(\mathrm{D_O}^{\substack{\bullet\\\bullet\\\bullet}}\Big) - \underset{\mathrm{AKo}}{\nu_r}\Big(2\Big(\mathrm{D_O}^{\substack{\bullet\\\bullet\\\bullet}} + \mathrm{D_O}^{\substack{\bullet\\\bullet\\\circ}} + \mathrm{D_O}^{\substack{\bullet\\\circ\\\bullet}} + \mathrm{D_O}^{\substack{\bullet\\\circ\\\circ}} + \mathrm{D_O}^{\substack{\circ\\\bullet\\\bullet}} + \mathrm{D_O}^{\substack{\circ\\\bullet\\\circ}} + \mathrm{D_O}^{\substack{\circ\\\circ\\\bullet}} + \mathrm{D_O}^{\substack{\circ\\\circ\\\circ}}\Big)\mathrm{D_O}^{\substack{\bullet\\\bullet\\\bullet}}\Big) - \underset{\mathrm{CKo}}{\nu_r}\Big(\Big(\mathrm{C_O}^{\substack{\bullet\\\bullet\\\bullet}} + \mathrm{C_O}^{\substack{\bullet\\\bullet\\\circ}} + \mathrm{C_O}^{\substack{\bullet\\\circ\\\bullet}} + \mathrm{C_O}^{\substack{\bullet\\\circ\\\circ}} + \mathrm{C_O}^{\substack{\circ\\\bullet\\\bullet}} + \mathrm{C_O}^{\substack{\circ\\\bullet\\\circ}} + \mathrm{C_O}^{\substack{\circ\\\circ\\\bullet}} + \mathrm{C_O}^{\substack{\circ\\\circ\\\circ}}\Big)\mathrm{D_O}^{\substack{\bullet\\\bullet\\\bullet}}\Big)$$

$$\frac{d\mathrm{D_S}^{\substack{\circ\\\circ\\\circ}}}{dt} = \underset{\mathrm{ADPMS}}{\nu_f}\Big(\mathrm{D_M}^{\substack{\circ\\\circ\\\circ}}\Big) + \underset{\mathrm{ASs}}{\nu_r}\Big(\Big(\mathrm{T_S}^{\substack{\circ\bullet\\\circ\bullet\\\circ\bullet}} + \mathrm{T_S}^{\substack{\circ\bullet\\\circ\bullet\\\circ\circ}} + \mathrm{T_S}^{\substack{\circ\bullet\\\circ\circ\\\circ\bullet}} + \mathrm{T_S}^{\substack{\circ\bullet\\\circ\circ\\\circ\circ}} + \mathrm{T_S}^{\substack{\circ\circ\\\circ\bullet\\\circ\bullet}} + \mathrm{T_S}^{\substack{\circ\circ\\\circ\bullet\\\circ\circ}} + \mathrm{T_S}^{\substack{\circ\circ\\\circ\circ\\\circ\bullet}} + \mathrm{T_S}^{\substack{\circ\circ\\\circ\circ\\\circ\circ}}\Big)\Big(\mathrm{W_S}^{\bullet} + \mathrm{W_S}^{\circ}\Big)\Big) - \underset{\mathrm{ASs}}{\nu_f}\Big(\Big(\mathrm{P_S}^{\substack{\bullet\\\bullet\\\bullet\\\bullet}} + \mathrm{P_S}^{\substack{\bullet\\\bullet\\\bullet\\\circ}} + \mathrm{P_S}^{\substack{\bullet\\\bullet\\\circ\\\bullet}} + \mathrm{P_S}^{\substack{\bullet\\\bullet\\\circ\\\circ}} + \mathrm{P_S}^{\substack{\bullet\\\circ\\\bullet\\\bullet}} + \mathrm{P_S}^{\substack{\bullet\\\circ\\\bullet\\\circ}} + \mathrm{P_S}^{\substack{\bullet\\\circ\\\circ\\\bullet}} + \mathrm{P_S}^{\substack{\bullet\\\circ\\\circ\\\circ}} + \mathrm{P_S}^{\substack{\circ\\\bullet\\\bullet\\\bullet}} + \mathrm{P_S}^{\substack{\circ\\\bullet\\\bullet\\\circ}} + \mathrm{P_S}^{\substack{\circ\\\bullet\\\circ\\\bullet}} + \mathrm{P_S}^{\substack{\circ\\\bullet\\\circ\\\circ}} + \mathrm{P_S}^{\substack{\circ\\\circ\\\bullet\\\bullet}} + \mathrm{P_S}^{\substack{\circ\\\circ\\\bullet\\\circ}} + \mathrm{P_S}^{\substack{\circ\\\circ\\\circ\\\bullet}} + \mathrm{P_S}^{\substack{\circ\\\circ\\\circ\\\circ}}\Big)\mathrm{D_S}^{\substack{\circ\\\circ\\\circ}}\Big) - \underset{\mathrm{ADPMS}}{\nu_r}\Big(\mathrm{D_S}^{\substack{\circ\\\circ\\\circ}}\Big)$$

$$\frac{d\mathrm{D_S}^{\substack{\circ\\\circ\\\bullet}}}{dt} = \underset{\mathrm{ADPMS}}{\nu_f}\Big(\mathrm{D_M}^{\substack{\circ\\\circ\\\bullet}}\Big) + \underset{\mathrm{ASs}}{\nu_r}\Big(\Big(\mathrm{T_S}^{\substack{\circ\bullet\\\circ\bullet\\\bullet\bullet}} + \mathrm{T_S}^{\substack{\circ\bullet\\\circ\bullet\\\bullet\circ}} + \mathrm{T_S}^{\substack{\circ\bullet\\\circ\circ\\\bullet\bullet}} + \mathrm{T_S}^{\substack{\circ\bullet\\\circ\circ\\\bullet\circ}} + \mathrm{T_S}^{\substack{\circ\circ\\\circ\bullet\\\bullet\bullet}} + \mathrm{T_S}^{\substack{\circ\circ\\\circ\bullet\\\bullet\circ}} + \mathrm{T_S}^{\substack{\circ\circ\\\circ\circ\\\bullet\bullet}} + \mathrm{T_S}^{\substack{\circ\circ\\\circ\circ\\\bullet\circ}}\Big)\Big(\mathrm{W_S}^{\bullet} + \mathrm{W_S}^{\circ}\Big)\Big) - \underset{\mathrm{ASs}}{\nu_f}\Big(\Big(\mathrm{P_S}^{\substack{\bullet\\\bullet\\\bullet\\\bullet}} + \mathrm{P_S}^{\substack{\bullet\\\bullet\\\bullet\\\circ}} + \mathrm{P_S}^{\substack{\bullet\\\bullet\\\circ\\\bullet}} + \mathrm{P_S}^{\substack{\bullet\\\bullet\\\circ\\\circ}} + \mathrm{P_S}^{\substack{\bullet\\\circ\\\bullet\\\bullet}} + \mathrm{P_S}^{\substack{\bullet\\\circ\\\bullet\\\circ}} + \mathrm{P_S}^{\substack{\bullet\\\circ\\\circ\\\bullet}} + \mathrm{P_S}^{\substack{\bullet\\\circ\\\circ\\\circ}} + \mathrm{P_S}^{\substack{\circ\\\bullet\\\bullet\\\bullet}} + \mathrm{P_S}^{\substack{\circ\\\bullet\\\bullet\\\circ}} + \mathrm{P_S}^{\substack{\circ\\\bullet\\\circ\\\bullet}} + \mathrm{P_S}^{\substack{\circ\\\bullet\\\circ\\\circ}} + \mathrm{P_S}^{\substack{\circ\\\circ\\\bullet\\\bullet}} + \mathrm{P_S}^{\substack{\circ\\\circ\\\bullet\\\circ}} + \mathrm{P_S}^{\substack{\circ\\\circ\\\circ\\\bullet}} + \mathrm{P_S}^{\substack{\circ\\\circ\\\circ\\\circ}}\Big)\mathrm{D_S}^{\substack{\circ\\\circ\\\bullet}}\Big) - \underset{\mathrm{ADPMS}}{\nu_r}\Big(\mathrm{D_S}^{\substack{\circ\\\circ\\\bullet}}\Big)$$

$$\frac{d\mathrm{D_S}^{\substack{\circ\\\bullet\\\circ}}}{dt} = \underset{\mathrm{ADPMS}}{\nu_f}\Big(\mathrm{D_M}^{\substack{\circ\\\bullet\\\circ}}\Big) + \underset{\mathrm{ASs}}{\nu_r}\Big(\Big(\mathrm{T_S}^{\substack{\circ\bullet\\\bullet\bullet\\\circ\bullet}} + \mathrm{T_S}^{\substack{\circ\bullet\\\bullet\bullet\\\circ\circ}} + \mathrm{T_S}^{\substack{\circ\bullet\\\bullet\circ\\\circ\bullet}} + \mathrm{T_S}^{\substack{\circ\bullet\\\bullet\circ\\\circ\circ}} + \mathrm{T_S}^{\substack{\circ\circ\\\bullet\bullet\\\circ\bullet}} + \mathrm{T_S}^{\substack{\circ\circ\\\bullet\bullet\\\circ\circ}} + \mathrm{T_S}^{\substack{\circ\circ\\\bullet\circ\\\circ\bullet}} + \mathrm{T_S}^{\substack{\circ\circ\\\bullet\circ\\\circ\circ}}\Big)\Big(\mathrm{W_S}^{\bullet} + \mathrm{W_S}^{\circ}\Big)\Big) - \underset{\mathrm{ASs}}{\nu_f}\Big(\Big(\mathrm{P_S}^{\substack{\bullet\\\bullet\\\bullet\\\bullet}} + \mathrm{P_S}^{\substack{\bullet\\\bullet\\\bullet\\\circ}} + \mathrm{P_S}^{\substack{\bullet\\\bullet\\\circ\\\bullet}} + \mathrm{P_S}^{\substack{\bullet\\\bullet\\\circ\\\circ}} + \mathrm{P_S}^{\substack{\bullet\\\circ\\\bullet\\\bullet}} + \mathrm{P_S}^{\substack{\bullet\\\circ\\\bullet\\\circ}} + \mathrm{P_S}^{\substack{\bullet\\\circ\\\circ\\\bullet}} + \mathrm{P_S}^{\substack{\bullet\\\circ\\\circ\\\circ}} + \mathrm{P_S}^{\substack{\circ\\\bullet\\\bullet\\\bullet}} + \mathrm{P_S}^{\substack{\circ\\\bullet\\\bullet\\\circ}} + \mathrm{P_S}^{\substack{\circ\\\bullet\\\circ\\\bullet}} + \mathrm{P_S}^{\substack{\circ\\\bullet\\\circ\\\circ}} + \mathrm{P_S}^{\substack{\circ\\\circ\\\bullet\\\bullet}} + \mathrm{P_S}^{\substack{\circ\\\circ\\\bullet\\\circ}} + \mathrm{P_S}^{\substack{\circ\\\circ\\\circ\\\bullet}} + \mathrm{P_S}^{\substack{\circ\\\circ\\\circ\\\circ}}\Big)\mathrm{D_S}^{\substack{\circ\\\bullet\\\circ}}\Big) - \underset{\mathrm{ADPMS}}{\nu_r}\Big(\mathrm{D_S}^{\substack{\circ\\\bullet\\\circ}}\Big)$$

$$\frac{d\mathrm{D_S}^{\substack{\circ\\\bullet\\\bullet}}}{dt} = \underset{\mathrm{ADPMS}}{\nu_f}\Big(\mathrm{D_M}^{\substack{\circ\\\bullet\\\bullet}}\Big) + \underset{\mathrm{ASs}}{\nu_r}\Big(\Big(\mathrm{T_S}^{\substack{\circ\bullet\\\bullet\bullet\\\bullet\bullet}} + \mathrm{T_S}^{\substack{\circ\bullet\\\bullet\bullet\\\bullet\circ}} + \mathrm{T_S}^{\substack{\circ\bullet\\\bullet\circ\\\bullet\bullet}} + \mathrm{T_S}^{\substack{\circ\bullet\\\bullet\circ\\\bullet\circ}} + \mathrm{T_S}^{\substack{\circ\circ\\\bullet\bullet\\\bullet\bullet}} + \mathrm{T_S}^{\substack{\circ\circ\\\bullet\bullet\\\bullet\circ}} + \mathrm{T_S}^{\substack{\circ\circ\\\bullet\circ\\\bullet\bullet}} + \mathrm{T_S}^{\substack{\circ\circ\\\bullet\circ\\\bullet\circ}}\Big)\Big(\mathrm{W_S}^{\bullet} + \mathrm{W_S}^{\circ}\Big)\Big) - \underset{\mathrm{ASs}}{\nu_f}\Big(\Big(\mathrm{P_S}^{\substack{\bullet\\\bullet\\\bullet\\\bullet}} + \mathrm{P_S}^{\substack{\bullet\\\bullet\\\bullet\\\circ}} + \mathrm{P_S}^{\substack{\bullet\\\bullet\\\circ\\\bullet}} + \mathrm{P_S}^{\substack{\bullet\\\bullet\\\circ\\\circ}} + \mathrm{P_S}^{\substack{\bullet\\\circ\\\bullet\\\bullet}} + \mathrm{P_S}^{\substack{\bullet\\\circ\\\bullet\\\circ}} + \mathrm{P_S}^{\substack{\bullet\\\circ\\\circ\\\bullet}} + \mathrm{P_S}^{\substack{\bullet\\\circ\\\circ\\\circ}} + \mathrm{P_S}^{\substack{\circ\\\bullet\\\bullet\\\bullet}} + \mathrm{P_S}^{\substack{\circ\\\bullet\\\bullet\\\circ}} + \mathrm{P_S}^{\substack{\circ\\\bullet\\\circ\\\bullet}} + \mathrm{P_S}^{\substack{\circ\\\bullet\\\circ\\\circ}} + \mathrm{P_S}^{\substack{\circ\\\circ\\\bullet\\\bullet}} + \mathrm{P_S}^{\substack{\circ\\\circ\\\bullet\\\circ}} + \mathrm{P_S}^{\substack{\circ\\\circ\\\circ\\\bullet}} + \mathrm{P_S}^{\substack{\circ\\\circ\\\circ\\\circ}}\Big)\mathrm{D_S}^{\substack{\circ\\\bullet\\\bullet}}\Big) - \underset{\mathrm{ADPMS}}{\nu_r}\Big(\mathrm{D_S}^{\substack{\circ\\\bullet\\\bullet}}\Big)$$

$$\frac{d\mathrm{D_S}^{\substack{\bullet\\\circ\\\circ}}}{dt} = \underset{\mathrm{ADPMS}}{\nu_f}\Big(\mathrm{D_M}^{\substack{\bullet\\\circ\\\circ}}\Big) + \underset{\mathrm{ASs}}{\nu_r}\Big(\Big(\mathrm{T_S}^{\substack{\bullet\bullet\\\circ\bullet\\\circ\bullet}} + \mathrm{T_S}^{\substack{\bullet\bullet\\\circ\bullet\\\circ\circ}} + \mathrm{T_S}^{\substack{\bullet\bullet\\\circ\circ\\\circ\bullet}} + \mathrm{T_S}^{\substack{\bullet\bullet\\\circ\circ\\\circ\circ}} + \mathrm{T_S}^{\substack{\bullet\circ\\\circ\bullet\\\circ\bullet}} + \mathrm{T_S}^{\substack{\bullet\circ\\\circ\bullet\\\circ\circ}} + \mathrm{T_S}^{\substack{\bullet\circ\\\circ\circ\\\circ\bullet}} + \mathrm{T_S}^{\substack{\bullet\circ\\\circ\circ\\\circ\circ}}\Big)\Big(\mathrm{W_S}^{\bullet} + \mathrm{W_S}^{\circ}\Big)\Big) - \underset{\mathrm{ASs}}{\nu_f}\Big(\Big(\mathrm{P_S}^{\substack{\bullet\\\bullet\\\bullet\\\bullet}} + \mathrm{P_S}^{\substack{\bullet\\\bullet\\\bullet\\\circ}} + \mathrm{P_S}^{\substack{\bullet\\\bullet\\\circ\\\bullet}} + \mathrm{P_S}^{\substack{\bullet\\\bullet\\\circ\\\circ}} + \mathrm{P_S}^{\substack{\bullet\\\circ\\\bullet\\\bullet}} + \mathrm{P_S}^{\substack{\bullet\\\circ\\\bullet\\\circ}} + \mathrm{P_S}^{\substack{\bullet\\\circ\\\circ\\\bullet}} + \mathrm{P_S}^{\substack{\bullet\\\circ\\\circ\\\circ}} + \mathrm{P_S}^{\substack{\circ\\\bullet\\\bullet\\\bullet}} + \mathrm{P_S}^{\substack{\circ\\\bullet\\\bullet\\\circ}} + \mathrm{P_S}^{\substack{\circ\\\bullet\\\circ\\\bullet}} + \mathrm{P_S}^{\substack{\circ\\\bullet\\\circ\\\circ}} + \mathrm{P_S}^{\substack{\circ\\\circ\\\bullet\\\bullet}} + \mathrm{P_S}^{\substack{\circ\\\circ\\\bullet\\\circ}} + \mathrm{P_S}^{\substack{\circ\\\circ\\\circ\\\bullet}} + \mathrm{P_S}^{\substack{\circ\\\circ\\\circ\\\circ}}\Big)\mathrm{D_S}^{\substack{\bullet\\\circ\\\circ}}\Big) - \underset{\mathrm{ADPMS}}{\nu_r}\Big(\mathrm{D_S}^{\substack{\bullet\\\circ\\\circ}}\Big)$$

$$\frac{d\mathrm{D_S}^{\substack{\bullet\\\circ\\\bullet}}}{dt} = \underset{\mathrm{ADPMS}}{\nu_f}\Big(\mathrm{D_M}^{\substack{\bullet\\\circ\\\bullet}}\Big) + \underset{\mathrm{ASs}}{\nu_r}\Big(\Big(\mathrm{T_S}^{\substack{\bullet\bullet\\\circ\bullet\\\bullet\bullet}} + \mathrm{T_S}^{\substack{\bullet\bullet\\\circ\bullet\\\bullet\circ}} + \mathrm{T_S}^{\substack{\bullet\bullet\\\circ\circ\\\bullet\bullet}} + \mathrm{T_S}^{\substack{\bullet\bullet\\\circ\circ\\\bullet\circ}} + \mathrm{T_S}^{\substack{\bullet\circ\\\circ\bullet\\\bullet\bullet}} + \mathrm{T_S}^{\substack{\bullet\circ\\\circ\bullet\\\bullet\circ}} + \mathrm{T_S}^{\substack{\bullet\circ\\\circ\circ\\\bullet\bullet}} + \mathrm{T_S}^{\substack{\bullet\circ\\\circ\circ\\\bullet\circ}}\Big)\Big(\mathrm{W_S}^{\bullet} + \mathrm{W_S}^{\circ}\Big)\Big) - \underset{\mathrm{ASs}}{\nu_f}\Big(\Big(\mathrm{P_S}^{\substack{\bullet\\\bullet\\\bullet\\\bullet}} + \mathrm{P_S}^{\substack{\bullet\\\bullet\\\bullet\\\circ}} + \mathrm{P_S}^{\substack{\bullet\\\bullet\\\circ\\\bullet}} + \mathrm{P_S}^{\substack{\bullet\\\bullet\\\circ\\\circ}} + \mathrm{P_S}^{\substack{\bullet\\\circ\\\bullet\\\bullet}} + \mathrm{P_S}^{\substack{\bullet\\\circ\\\bullet\\\circ}} + \mathrm{P_S}^{\substack{\bullet\\\circ\\\circ\\\bullet}} + \mathrm{P_S}^{\substack{\bullet\\\circ\\\circ\\\circ}} + \mathrm{P_S}^{\substack{\circ\\\bullet\\\bullet\\\bullet}} + \mathrm{P_S}^{\substack{\circ\\\bullet\\\bullet\\\circ}} + \mathrm{P_S}^{\substack{\circ\\\bullet\\\circ\\\bullet}} + \mathrm{P_S}^{\substack{\circ\\\bullet\\\circ\\\circ}} + \mathrm{P_S}^{\substack{\circ\\\circ\\\bullet\\\bullet}} + \mathrm{P_S}^{\substack{\circ\\\circ\\\bullet\\\circ}} + \mathrm{P_S}^{\substack{\circ\\\circ\\\circ\\\bullet}} + \mathrm{P_S}^{\substack{\circ\\\circ\\\circ\\\circ}}\Big)\mathrm{D_S}^{\substack{\bullet\\\circ\\\bullet}}\Big) - \underset{\mathrm{ADPMS}}{\nu_r}\Big(\mathrm{D_S}^{\substack{\bullet\\\circ\\\bullet}}\Big)$$

$$\frac{d\mathrm{D_S}^{\substack{\bullet\\\bullet\\\circ}}}{dt} = \underset{\mathrm{ADPMS}}{\nu_f}\Big(\mathrm{D_M}^{\substack{\bullet\\\bullet\\\circ}}\Big) + \underset{\mathrm{ASs}}{\nu_r}\Big(\Big(\mathrm{T_S}^{\substack{\bullet\bullet\\\bullet\bullet\\\circ\bullet}} + \mathrm{T_S}^{\substack{\bullet\bullet\\\bullet\bullet\\\circ\circ}} + \mathrm{T_S}^{\substack{\bullet\bullet\\\bullet\circ\\\circ\bullet}} + \mathrm{T_S}^{\substack{\bullet\bullet\\\bullet\circ\\\circ\circ}} + \mathrm{T_S}^{\substack{\bullet\circ\\\bullet\bullet\\\circ\bullet}} + \mathrm{T_S}^{\substack{\bullet\circ\\\bullet\bullet\\\circ\circ}} + \mathrm{T_S}^{\substack{\bullet\circ\\\bullet\circ\\\circ\bullet}} + \mathrm{T_S}^{\substack{\bullet\circ\\\bullet\circ\\\circ\circ}}\Big)\Big(\mathrm{W_S}^{\bullet} + \mathrm{W_S}^{\circ}\Big)\Big) - \underset{\mathrm{ASs}}{\nu_f}\Big(\Big(\mathrm{P_S}^{\substack{\bullet\\\bullet\\\bullet\\\bullet}} + \mathrm{P_S}^{\substack{\bullet\\\bullet\\\bullet\\\circ}} + \mathrm{P_S}^{\substack{\bullet\\\bullet\\\circ\\\bullet}} + \mathrm{P_S}^{\substack{\bullet\\\bullet\\\circ\\\circ}} + \mathrm{P_S}^{\substack{\bullet\\\circ\\\bullet\\\bullet}} + \mathrm{P_S}^{\substack{\bullet\\\circ\\\bullet\\\circ}} + \mathrm{P_S}^{\substack{\bullet\\\circ\\\circ\\\bullet}} + \mathrm{P_S}^{\substack{\bullet\\\circ\\\circ\\\circ}} + \mathrm{P_S}^{\substack{\circ\\\bullet\\\bullet\\\bullet}} + \mathrm{P_S}^{\substack{\circ\\\bullet\\\bullet\\\circ}} + \mathrm{P_S}^{\substack{\circ\\\bullet\\\circ\\\bullet}} + \mathrm{P_S}^{\substack{\circ\\\bullet\\\circ\\\circ}} + \mathrm{P_S}^{\substack{\circ\\\circ\\\bullet\\\bullet}} + \mathrm{P_S}^{\substack{\circ\\\circ\\\bullet\\\circ}} + \mathrm{P_S}^{\substack{\circ\\\circ\\\circ\\\bullet}} + \mathrm{P_S}^{\substack{\circ\\\circ\\\circ\\\circ}}\Big)\mathrm{D_S}^{\substack{\bullet\\\bullet\\\circ}}\Big) - \underset{\mathrm{ADPMS}}{\nu_r}\Big(\mathrm{D_S}^{\substack{\bullet\\\bullet\\\circ}}\Big)$$

$$\frac{d\mathrm{D_S}^{\substack{\bullet\\\bullet\\\bullet}}}{dt} = \underset{\mathrm{ADPMS}}{\nu_f}\Big(\mathrm{D_M}^{\substack{\bullet\\\bullet\\\bullet}}\Big) + \underset{\mathrm{ASs}}{\nu_r}\Big(\Big(\mathrm{T_S}^{\substack{\bullet\bullet\\\bullet\bullet\\\bullet\bullet}} + \mathrm{T_S}^{\substack{\bullet\bullet\\\bullet\bullet\\\bullet\circ}} + \mathrm{T_S}^{\substack{\bullet\bullet\\\bullet\circ\\\bullet\bullet}} + \mathrm{T_S}^{\substack{\bullet\bullet\\\bullet\circ\\\bullet\circ}} + \mathrm{T_S}^{\substack{\bullet\circ\\\bullet\bullet\\\bullet\bullet}} + \mathrm{T_S}^{\substack{\bullet\circ\\\bullet\bullet\\\bullet\circ}} + \mathrm{T_S}^{\substack{\bullet\circ\\\bullet\circ\\\bullet\bullet}} + \mathrm{T_S}^{\substack{\bullet\circ\\\bullet\circ\\\bullet\circ}}\Big)\Big(\mathrm{W_S}^{\bullet} + \mathrm{W_S}^{\circ}\Big)\Big) - \underset{\mathrm{ASs}}{\nu_f}\Big(\Big(\mathrm{P_S}^{\substack{\bullet\\\bullet\\\bullet\\\bullet}} + \mathrm{P_S}^{\substack{\bullet\\\bullet\\\bullet\\\circ}} + \mathrm{P_S}^{\substack{\bullet\\\bullet\\\circ\\\bullet}} + \mathrm{P_S}^{\substack{\bullet\\\bullet\\\circ\\\circ}} + \mathrm{P_S}^{\substack{\bullet\\\circ\\\bullet\\\bullet}} + \mathrm{P_S}^{\substack{\bullet\\\circ\\\bullet\\\circ}} + \mathrm{P_S}^{\substack{\bullet\\\circ\\\circ\\\bullet}} + \mathrm{P_S}^{\substack{\bullet\\\circ\\\circ\\\circ}} + \mathrm{P_S}^{\substack{\circ\\\bullet\\\bullet\\\bullet}} + \mathrm{P_S}^{\substack{\circ\\\bullet\\\bullet\\\circ}} + \mathrm{P_S}^{\substack{\circ\\\bullet\\\circ\\\bullet}} + \mathrm{P_S}^{\substack{\circ\\\bullet\\\circ\\\circ}} + \mathrm{P_S}^{\substack{\circ\\\circ\\\bullet\\\bullet}} + \mathrm{P_S}^{\substack{\circ\\\circ\\\bullet\\\circ}} + \mathrm{P_S}^{\substack{\circ\\\circ\\\circ\\\bullet}} + \mathrm{P_S}^{\substack{\circ\\\circ\\\circ\\\circ}}\Big)\mathrm{D_S}^{\substack{\bullet\\\bullet\\\bullet}}\Big) - \underset{\mathrm{ADPMS}}{\nu_r}\Big(\mathrm{D_S}^{\substack{\bullet\\\bullet\\\bullet}}\Big)$$

$$\frac{d\mathrm{T_E}^{\substack{\circ\circ\\\circ\circ\\\circ\circ}}}{dt} = \underset{\mathrm{ATPoE}}{\nu_f}\Big(\mathrm{T_O}^{\substack{\circ\circ\\\circ\circ\\\circ\circ}}\Big) + \underset{\mathrm{ASE}}{\nu_r}\Big(\Big(\mathrm{P_E}^{\substack{\circ\\\circ\\\circ\\\circ}} + \frac{1}{4}\Big(\mathrm{P_E}^{\substack{\bullet\\\circ\\\circ\\\circ}} + \mathrm{P_E}^{\substack{\circ\\\bullet\\\circ\\\circ}} + \mathrm{P_E}^{\substack{\circ\\\circ\\\bullet\\\circ}} + \mathrm{P_E}^{\substack{\circ\\\circ\\\circ\\\bullet}}\Big)\Big)\mathrm{D_E}^{\substack{\circ\\\circ\\\circ}}\Big) - \underset{\mathrm{ASE}}{\nu_f}\Big(\Big(\mathrm{W_E}^{\bullet} + \mathrm{W_E}^{\circ}\Big)\mathrm{T_E}^{\substack{\circ\circ\\\circ\circ\\\circ\circ}}\Big)$$

$$\frac{d\mathrm{T_E}^{\substack{\circ\circ\\\circ\circ\\\circ\bullet}}}{dt} = \underset{\mathrm{ATPoE}}{\nu_f}\left(\mathrm{T_O}^{\substack{\circ\circ\\\circ\circ\\\circ\bullet}}\right) + \underset{\mathrm{ASE}}{\nu_r}\left(\left(\frac{1}{4}\left(\mathrm{P_E}^{\substack{\bullet\\\circ\\\circ\\\circ}}+\mathrm{P_E}^{\substack{\circ\\\bullet\\\circ\\\circ}}+\mathrm{P_E}^{\substack{\circ\\\circ\\\bullet\\\circ}}+\mathrm{P_E}^{\substack{\circ\\\circ\\\circ\\\bullet}}\right)+\frac{1}{6}\left(\mathrm{P_E}^{\substack{\bullet\\\bullet\\\circ\\\circ}}+\mathrm{P_E}^{\substack{\bullet\\\circ\\\bullet\\\circ}}+\mathrm{P_E}^{\substack{\bullet\\\circ\\\circ\\\bullet}}+\mathrm{P_E}^{\substack{\circ\\\bullet\\\bullet\\\circ}}+\mathrm{P_E}^{\substack{\circ\\\bullet\\\circ\\\bullet}}+\mathrm{P_E}^{\substack{\circ\\\circ\\\bullet\\\bullet}}\right)\right)\mathrm{D_E}^{\substack{\circ\\\circ\\\circ}}\right) - \underset{\mathrm{ASE}}{\nu_f}\left(\left(\mathrm{W_E}^{\bullet}+\mathrm{W_E}^{\circ}\right)\mathrm{T_E}^{\substack{\circ\circ\\\circ\circ\\\circ\bullet}}\right)$$

$$\frac{d\mathrm{T_E}^{\substack{\circ\circ\\\circ\bullet\\\circ\circ}}}{dt} = \underset{\mathrm{ATPoE}}{\nu_f}\left(\mathrm{T_O}^{\substack{\circ\circ\\\circ\bullet\\\circ\circ}}\right) + \underset{\mathrm{ASE}}{\nu_r}\left(\left(\frac{1}{4}\left(\mathrm{P_E}^{\substack{\bullet\\\circ\\\circ\\\circ}}+\mathrm{P_E}^{\substack{\circ\\\bullet\\\circ\\\circ}}+\mathrm{P_E}^{\substack{\circ\\\circ\\\bullet\\\circ}}+\mathrm{P_E}^{\substack{\circ\\\circ\\\circ\\\bullet}}\right)+\frac{1}{6}\left(\mathrm{P_E}^{\substack{\bullet\\\bullet\\\circ\\\circ}}+\mathrm{P_E}^{\substack{\bullet\\\circ\\\bullet\\\circ}}+\mathrm{P_E}^{\substack{\bullet\\\circ\\\circ\\\bullet}}+\mathrm{P_E}^{\substack{\circ\\\bullet\\\bullet\\\circ}}+\mathrm{P_E}^{\substack{\circ\\\bullet\\\circ\\\bullet}}+\mathrm{P_E}^{\substack{\circ\\\circ\\\bullet\\\bullet}}\right)\right)\mathrm{D_E}^{\substack{\circ\\\circ\\\circ}}\right) - \underset{\mathrm{ASE}}{\nu_f}\left(\left(\mathrm{W_E}^{\bullet}+\mathrm{W_E}^{\circ}\right)\mathrm{T_E}^{\substack{\circ\circ\\\circ\bullet\\\circ\circ}}\right)$$

$$\frac{d\mathrm{T_E}^{\substack{\circ\circ\\\circ\bullet\\\circ\bullet}}}{dt} = \underset{\mathrm{ATPoE}}{\nu_f}\left(\mathrm{T_O}^{\substack{\circ\circ\\\circ\bullet\\\circ\bullet}}\right) + \underset{\mathrm{ASE}}{\nu_r}\left(\left(\frac{1}{4}\left(\mathrm{P_E}^{\substack{\bullet\\\bullet\\\bullet\\\circ}}+\mathrm{P_E}^{\substack{\bullet\\\bullet\\\circ\\\bullet}}+\mathrm{P_E}^{\substack{\bullet\\\circ\\\bullet\\\bullet}}+\mathrm{P_E}^{\substack{\circ\\\bullet\\\bullet\\\bullet}}\right)+\frac{1}{6}\left(\mathrm{P_E}^{\substack{\bullet\\\bullet\\\circ\\\circ}}+\mathrm{P_E}^{\substack{\bullet\\\circ\\\bullet\\\circ}}+\mathrm{P_E}^{\substack{\bullet\\\circ\\\circ\\\bullet}}+\mathrm{P_E}^{\substack{\circ\\\bullet\\\bullet\\\circ}}+\mathrm{P_E}^{\substack{\circ\\\bullet\\\circ\\\bullet}}+\mathrm{P_E}^{\substack{\circ\\\circ\\\bullet\\\bullet}}\right)\right)\mathrm{D_E}^{\substack{\circ\\\circ\\\circ}}\right) - \underset{\mathrm{ASE}}{\nu_f}\left(\left(\mathrm{W_E}^{\bullet}+\mathrm{W_E}^{\circ}\right)\mathrm{T_E}^{\substack{\circ\circ\\\circ\bullet\\\circ\bullet}}\right)$$

$$\frac{d\mathrm{T_E}^{\substack{\circ\bullet\\\circ\circ\\\circ\circ}}}{dt} = \underset{\mathrm{ATPoE}}{\nu_f}\left(\mathrm{T_O}^{\substack{\circ\bullet\\\circ\circ\\\circ\circ}}\right) + \underset{\mathrm{ASE}}{\nu_r}\left(\left(\frac{1}{4}\left(\mathrm{P_E}^{\substack{\bullet\\\circ\\\circ\\\circ}}+\mathrm{P_E}^{\substack{\circ\\\bullet\\\circ\\\circ}}+\mathrm{P_E}^{\substack{\circ\\\circ\\\bullet\\\circ}}+\mathrm{P_E}^{\substack{\circ\\\circ\\\circ\\\bullet}}\right)+\frac{1}{6}\left(\mathrm{P_E}^{\substack{\bullet\\\bullet\\\circ\\\circ}}+\mathrm{P_E}^{\substack{\bullet\\\circ\\\bullet\\\circ}}+\mathrm{P_E}^{\substack{\bullet\\\circ\\\circ\\\bullet}}+\mathrm{P_E}^{\substack{\circ\\\bullet\\\bullet\\\circ}}+\mathrm{P_E}^{\substack{\circ\\\bullet\\\circ\\\bullet}}+\mathrm{P_E}^{\substack{\circ\\\circ\\\bullet\\\bullet}}\right)\right)\mathrm{D_E}^{\substack{\circ\\\circ\\\circ}}\right) - \underset{\mathrm{ASE}}{\nu_f}\left(\left(\mathrm{W_E}^{\bullet}+\mathrm{W_E}^{\circ}\right)\mathrm{T_E}^{\substack{\circ\bullet\\\circ\circ\\\circ\circ}}\right)$$

$$\frac{d\mathrm{T_E}^{\substack{\circ\bullet\\\circ\circ\\\circ\bullet}}}{dt} = \underset{\mathrm{ATPoE}}{\nu_f}\left(\mathrm{T_O}^{\substack{\circ\bullet\\\circ\circ\\\circ\bullet}}\right) + \underset{\mathrm{ASE}}{\nu_r}\left(\left(\frac{1}{4}\left(\mathrm{P_E}^{\substack{\bullet\\\bullet\\\bullet\\\circ}}+\mathrm{P_E}^{\substack{\bullet\\\bullet\\\circ\\\bullet}}+\mathrm{P_E}^{\substack{\bullet\\\circ\\\bullet\\\bullet}}+\mathrm{P_E}^{\substack{\circ\\\bullet\\\bullet\\\bullet}}\right)+\frac{1}{6}\left(\mathrm{P_E}^{\substack{\bullet\\\bullet\\\circ\\\circ}}+\mathrm{P_E}^{\substack{\bullet\\\circ\\\bullet\\\circ}}+\mathrm{P_E}^{\substack{\bullet\\\circ\\\circ\\\bullet}}+\mathrm{P_E}^{\substack{\circ\\\bullet\\\bullet\\\circ}}+\mathrm{P_E}^{\substack{\circ\\\bullet\\\circ\\\bullet}}+\mathrm{P_E}^{\substack{\circ\\\circ\\\bullet\\\bullet}}\right)\right)\mathrm{D_E}^{\substack{\circ\\\circ\\\circ}}\right) - \underset{\mathrm{ASE}}{\nu_f}\left(\left(\mathrm{W_E}^{\bullet}+\mathrm{W_E}^{\circ}\right)\mathrm{T_E}^{\substack{\circ\bullet\\\circ\circ\\\circ\bullet}}\right)$$

$$\frac{d\mathrm{T_E}^{\substack{\circ\bullet\\\circ\bullet\\\circ\circ}}}{dt} = \underset{\mathrm{ATPoE}}{\nu_f}\left(\mathrm{T_O}^{\substack{\circ\bullet\\\circ\bullet\\\circ\circ}}\right) + \underset{\mathrm{ASE}}{\nu_r}\left(\left(\frac{1}{4}\left(\mathrm{P_E}^{\substack{\bullet\\\bullet\\\bullet\\\circ}}+\mathrm{P_E}^{\substack{\bullet\\\bullet\\\circ\\\bullet}}+\mathrm{P_E}^{\substack{\bullet\\\circ\\\bullet\\\bullet}}+\mathrm{P_E}^{\substack{\circ\\\bullet\\\bullet\\\bullet}}\right)+\frac{1}{6}\left(\mathrm{P_E}^{\substack{\bullet\\\bullet\\\circ\\\circ}}+\mathrm{P_E}^{\substack{\bullet\\\circ\\\bullet\\\circ}}+\mathrm{P_E}^{\substack{\bullet\\\circ\\\circ\\\bullet}}+\mathrm{P_E}^{\substack{\circ\\\bullet\\\bullet\\\circ}}+\mathrm{P_E}^{\substack{\circ\\\bullet\\\circ\\\bullet}}+\mathrm{P_E}^{\substack{\circ\\\circ\\\bullet\\\bullet}}\right)\right)\mathrm{D_E}^{\substack{\circ\\\circ\\\circ}}\right) - \underset{\mathrm{ASE}}{\nu_f}\left(\left(\mathrm{W_E}^{\bullet}+\mathrm{W_E}^{\circ}\right)\mathrm{T_E}^{\substack{\circ\bullet\\\circ\bullet\\\circ\circ}}\right)$$

$$\frac{d\mathrm{T_E}^{\substack{\circ\bullet\\\circ\bullet\\\circ\bullet}}}{dt} = \underset{\mathrm{ATPoE}}{\nu_f}\left(\mathrm{T_O}^{\substack{\circ\bullet\\\circ\bullet\\\circ\bullet}}\right) + \underset{\mathrm{ASE}}{\nu_r}\left(\left(\mathrm{P_E}^{\substack{\bullet\\\bullet\\\bullet\\\bullet}} + \frac{1}{4}\left(\mathrm{P_E}^{\substack{\bullet\\\bullet\\\bullet\\\circ}} + \mathrm{P_E}^{\substack{\bullet\\\bullet\\\circ\\\bullet}} + \mathrm{P_E}^{\substack{\bullet\\\circ\\\bullet\\\bullet}} + \mathrm{P_E}^{\substack{\circ\\\bullet\\\bullet\\\bullet}}\right)\right)\mathrm{D_E}^{\substack{\circ\\\circ\\\circ}}\right) - \underset{\mathrm{ASE}}{\nu_f}\left(\left(\mathrm{W_E}^{\bullet} + \mathrm{W_E}^{\circ}\right)\mathrm{T_E}^{\substack{\circ\bullet\\\circ\bullet\\\circ\bullet}}\right)$$

$$\frac{d\mathrm{T_E}^{\substack{\circ\circ\\\circ\circ\\\bullet\circ}}}{dt} = \underset{\mathrm{ATPoE}}{\nu_f}\left(\mathrm{T_O}^{\substack{\circ\circ\\\circ\circ\\\bullet\circ}}\right) + \underset{\mathrm{ASE}}{\nu_r}\left(\left(\mathrm{P_E}^{\substack{\circ\\\circ\\\circ\\\circ}} + \frac{1}{4}\left(\mathrm{P_E}^{\substack{\bullet\\\circ\\\circ\\\circ}} + \mathrm{P_E}^{\substack{\circ\\\bullet\\\circ\\\circ}} + \mathrm{P_E}^{\substack{\circ\\\circ\\\bullet\\\circ}} + \mathrm{P_E}^{\substack{\circ\\\circ\\\circ\\\bullet}}\right)\right)\mathrm{D_E}^{\substack{\circ\\\circ\\\bullet}}\right) - \underset{\mathrm{ASE}}{\nu_f}\left(\left(\mathrm{W_E}^{\bullet} + \mathrm{W_E}^{\circ}\right)\mathrm{T_E}^{\substack{\circ\circ\\\circ\circ\\\bullet\circ}}\right)$$

$$\frac{d\mathrm{T_E}^{\substack{\circ\circ\\\circ\circ\\\bullet\bullet}}}{dt} = \underset{\mathrm{ATPoE}}{\nu_f}\left(\mathrm{T_O}^{\substack{\circ\circ\\\circ\circ\\\bullet\bullet}}\right) + \underset{\mathrm{ASE}}{\nu_r}\left(\left(\frac{1}{4}\left(\mathrm{P_E}^{\substack{\bullet\\\circ\\\circ\\\circ}}+\mathrm{P_E}^{\substack{\circ\\\bullet\\\circ\\\circ}}+\mathrm{P_E}^{\substack{\circ\\\circ\\\bullet\\\circ}}+\mathrm{P_E}^{\substack{\circ\\\circ\\\circ\\\bullet}}\right)+\frac{1}{6}\left(\mathrm{P_E}^{\substack{\bullet\\\bullet\\\circ\\\circ}}+\mathrm{P_E}^{\substack{\bullet\\\circ\\\bullet\\\circ}}+\mathrm{P_E}^{\substack{\bullet\\\circ\\\circ\\\bullet}}+\mathrm{P_E}^{\substack{\circ\\\bullet\\\bullet\\\circ}}+\mathrm{P_E}^{\substack{\circ\\\bullet\\\circ\\\bullet}}+\mathrm{P_E}^{\substack{\circ\\\circ\\\bullet\\\bullet}}\right)\right)\mathrm{D_E}^{\substack{\circ\\\circ\\\bullet}}\right) - \underset{\mathrm{ASE}}{\nu_f}\left(\left(\mathrm{W_E}^{\bullet}+\mathrm{W_E}^{\circ}\right)\mathrm{T_E}^{\substack{\circ\circ\\\circ\circ\\\bullet\bullet}}\right)$$

$$\frac{d\mathrm{T_E}^{\substack{\circ\circ\\\circ\bullet\\\bullet\circ}}}{dt} = \underset{\mathrm{ATPoE}}{\nu_f}\left(\mathrm{T_O}^{\substack{\circ\circ\\\circ\bullet\\\bullet\circ}}\right) + \underset{\mathrm{ASE}}{\nu_r}\left(\left(\frac{1}{4}\left(\mathrm{P_E}^{\substack{\bullet\\\circ\\\circ\\\circ}}+\mathrm{P_E}^{\substack{\circ\\\bullet\\\circ\\\circ}}+\mathrm{P_E}^{\substack{\circ\\\circ\\\bullet\\\circ}}+\mathrm{P_E}^{\substack{\circ\\\circ\\\circ\\\bullet}}\right)+\frac{1}{6}\left(\mathrm{P_E}^{\substack{\bullet\\\bullet\\\circ\\\circ}}+\mathrm{P_E}^{\substack{\bullet\\\circ\\\bullet\\\circ}}+\mathrm{P_E}^{\substack{\bullet\\\circ\\\circ\\\bullet}}+\mathrm{P_E}^{\substack{\circ\\\bullet\\\bullet\\\circ}}+\mathrm{P_E}^{\substack{\circ\\\bullet\\\circ\\\bullet}}+\mathrm{P_E}^{\substack{\circ\\\circ\\\bullet\\\bullet}}\right)\right)\mathrm{D_E}^{\substack{\circ\\\circ\\\bullet}}\right) - \underset{\mathrm{ASE}}{\nu_f}\left(\left(\mathrm{W_E}^{\bullet}+\mathrm{W_E}^{\circ}\right)\mathrm{T_E}^{\substack{\circ\circ\\\circ\bullet\\\bullet\circ}}\right)$$

$$\frac{d\mathrm{T_E}^{\substack{\circ\circ\\\circ\bullet\\\bullet\bullet}}}{dt} = \underset{\mathrm{ATPoE}}{\nu_f}\left(\mathrm{T_O}^{\substack{\circ\circ\\\circ\bullet\\\bullet\bullet}}\right) + \underset{\mathrm{ASE}}{\nu_r}\left(\left(\frac{1}{4}\left(\mathrm{P_E}^{\substack{\bullet\\\bullet\\\bullet\\\circ}}+\mathrm{P_E}^{\substack{\bullet\\\bullet\\\circ\\\bullet}}+\mathrm{P_E}^{\substack{\bullet\\\circ\\\bullet\\\bullet}}+\mathrm{P_E}^{\substack{\circ\\\bullet\\\bullet\\\bullet}}\right)+\frac{1}{6}\left(\mathrm{P_E}^{\substack{\bullet\\\bullet\\\circ\\\circ}}+\mathrm{P_E}^{\substack{\bullet\\\circ\\\bullet\\\circ}}+\mathrm{P_E}^{\substack{\bullet\\\circ\\\circ\\\bullet}}+\mathrm{P_E}^{\substack{\circ\\\bullet\\\bullet\\\circ}}+\mathrm{P_E}^{\substack{\circ\\\bullet\\\circ\\\bullet}}+\mathrm{P_E}^{\substack{\circ\\\circ\\\bullet\\\bullet}}\right)\right)\mathrm{D_E}^{\substack{\circ\\\circ\\\bullet}}\right) - \underset{\mathrm{ASE}}{\nu_f}\left(\left(\mathrm{W_E}^{\bullet}+\mathrm{W_E}^{\circ}\right)\mathrm{T_E}^{\substack{\circ\circ\\\circ\bullet\\\bullet\bullet}}\right)$$

$$\frac{d\mathrm{T_E}^{\substack{\circ\bullet\\\circ\circ\\\bullet\circ}}}{dt} = \underset{\mathrm{ATPoE}}{\nu_f}\left(\mathrm{T_O}^{\substack{\circ\bullet\\\circ\circ\\\bullet\circ}}\right) + \underset{\mathrm{ASE}}{\nu_r}\left(\left(\frac{1}{4}\left(\mathrm{P_E}^{\substack{\bullet\\\circ\\\circ\\\circ}}+\mathrm{P_E}^{\substack{\circ\\\bullet\\\circ\\\circ}}+\mathrm{P_E}^{\substack{\circ\\\circ\\\bullet\\\circ}}+\mathrm{P_E}^{\substack{\circ\\\circ\\\circ\\\bullet}}\right)+\frac{1}{6}\left(\mathrm{P_E}^{\substack{\bullet\\\bullet\\\circ\\\circ}}+\mathrm{P_E}^{\substack{\bullet\\\circ\\\bullet\\\circ}}+\mathrm{P_E}^{\substack{\bullet\\\circ\\\circ\\\bullet}}+\mathrm{P_E}^{\substack{\circ\\\bullet\\\bullet\\\circ}}+\mathrm{P_E}^{\substack{\circ\\\bullet\\\circ\\\bullet}}+\mathrm{P_E}^{\substack{\circ\\\circ\\\bullet\\\bullet}}\right)\right)\mathrm{D_E}^{\substack{\circ\\\circ\\\bullet}}\right) - \underset{\mathrm{ASE}}{\nu_f}\left(\left(\mathrm{W_E}^{\bullet}+\mathrm{W_E}^{\circ}\right)\mathrm{T_E}^{\substack{\circ\bullet\\\circ\circ\\\bullet\circ}}\right)$$

$$\frac{d\mathrm{T_E}^{\substack{\circ\bullet\\\circ\circ\\\bullet\bullet}}}{dt} = \underset{\mathrm{ATPoE}}{\nu_f}\left(\mathrm{T_O}^{\substack{\circ\bullet\\\circ\circ\\\bullet\bullet}}\right) + \underset{\mathrm{ASE}}{\nu_r}\left(\left(\frac{1}{4}\left(\mathrm{P_E}^{\substack{\bullet\\\bullet\\\bullet\\\circ}}+\mathrm{P_E}^{\substack{\bullet\\\bullet\\\circ\\\bullet}}+\mathrm{P_E}^{\substack{\bullet\\\circ\\\bullet\\\bullet}}+\mathrm{P_E}^{\substack{\circ\\\bullet\\\bullet\\\bullet}}\right)+\frac{1}{6}\left(\mathrm{P_E}^{\substack{\bullet\\\bullet\\\circ\\\circ}}+\mathrm{P_E}^{\substack{\bullet\\\circ\\\bullet\\\circ}}+\mathrm{P_E}^{\substack{\bullet\\\circ\\\circ\\\bullet}}+\mathrm{P_E}^{\substack{\circ\\\bullet\\\bullet\\\circ}}+\mathrm{P_E}^{\substack{\circ\\\bullet\\\circ\\\bullet}}+\mathrm{P_E}^{\substack{\circ\\\circ\\\bullet\\\bullet}}\right)\right)\mathrm{D_E}^{\substack{\circ\\\circ\\\bullet}}\right) - \underset{\mathrm{ASE}}{\nu_f}\left(\left(\mathrm{W_E}^{\bullet}+\mathrm{W_E}^{\circ}\right)\mathrm{T_E}^{\substack{\circ\bullet\\\circ\circ\\\bullet\bullet}}\right)$$

$$\frac{d\mathrm{T_E}^{\substack{\circ\bullet\\\circ\bullet\\\bullet\circ}}}{dt} = \underset{\mathrm{ATPoE}}{\nu_f}\left(\mathrm{T_O}^{\substack{\circ\bullet\\\circ\bullet\\\bullet\circ}}\right) + \underset{\mathrm{ASE}}{\nu_r}\left(\left(\frac{1}{4}\left(\mathrm{P_E}^{\substack{\bullet\\\bullet\\\bullet\\\circ}}+\mathrm{P_E}^{\substack{\bullet\\\bullet\\\circ\\\bullet}}+\mathrm{P_E}^{\substack{\bullet\\\circ\\\bullet\\\bullet}}+\mathrm{P_E}^{\substack{\circ\\\bullet\\\bullet\\\bullet}}\right)+\frac{1}{6}\left(\mathrm{P_E}^{\substack{\bullet\\\bullet\\\circ\\\circ}}+\mathrm{P_E}^{\substack{\bullet\\\circ\\\bullet\\\circ}}+\mathrm{P_E}^{\substack{\bullet\\\circ\\\circ\\\bullet}}+\mathrm{P_E}^{\substack{\circ\\\bullet\\\bullet\\\circ}}+\mathrm{P_E}^{\substack{\circ\\\bullet\\\circ\\\bullet}}+\mathrm{P_E}^{\substack{\circ\\\circ\\\bullet\\\bullet}}\right)\right)\mathrm{D_E}^{\substack{\circ\\\circ\\\bullet}}\right) - \underset{\mathrm{ASE}}{\nu_f}\left(\left(\mathrm{W_E}^{\bullet}+\mathrm{W_E}^{\circ}\right)\mathrm{T_E}^{\substack{\circ\bullet\\\circ\bullet\\\bullet\circ}}\right)$$

$$\frac{d\mathrm{T_E}^{\substack{\circ\bullet\\\circ\bullet\\\bullet\bullet}}}{dt} = \underset{\mathrm{ATPoE}}{\nu_f}\left(\mathrm{T_O}^{\substack{\circ\bullet\\\circ\bullet\\\bullet\bullet}}\right) + \underset{\mathrm{ASE}}{\nu_r}\left(\left(\mathrm{P_E}^{\substack{\bullet\\\bullet\\\bullet\\\bullet}} + \frac{1}{4}\left(\mathrm{P_E}^{\substack{\bullet\\\bullet\\\bullet\\\circ}} + \mathrm{P_E}^{\substack{\bullet\\\bullet\\\circ\\\bullet}} + \mathrm{P_E}^{\substack{\bullet\\\circ\\\bullet\\\bullet}} + \mathrm{P_E}^{\substack{\circ\\\bullet\\\bullet\\\bullet}}\right)\right)\mathrm{D_E}^{\substack{\circ\\\circ\\\bullet}}\right) - \underset{\mathrm{ASE}}{\nu_f}\left(\left(\mathrm{W_E}^{\bullet} + \mathrm{W_E}^{\circ}\right)\mathrm{T_E}^{\substack{\circ\bullet\\\circ\bullet\\\bullet\bullet}}\right)$$

$$\frac{d\mathrm{T_E}^{\substack{\circ\circ\\\bullet\circ\\\circ\circ}}}{dt} = \underset{\mathrm{ATPoE}}{\nu_f}\left(\mathrm{T_O}^{\substack{\circ\circ\\\bullet\circ\\\circ\circ}}\right) + \underset{\mathrm{ASE}}{\nu_r}\left(\left(\mathrm{P_E}^{\substack{\circ\\\circ\\\circ\\\circ}} + \frac{1}{4}\left(\mathrm{P_E}^{\substack{\bullet\\\circ\\\circ\\\circ}} + \mathrm{P_E}^{\substack{\circ\\\bullet\\\circ\\\circ}} + \mathrm{P_E}^{\substack{\circ\\\circ\\\bullet\\\circ}} + \mathrm{P_E}^{\substack{\circ\\\circ\\\circ\\\bullet}}\right)\right)\mathrm{D_E}^{\substack{\circ\\\bullet\\\circ}}\right) - \underset{\mathrm{ASE}}{\nu_f}\left(\left(\mathrm{W_E}^{\bullet} + \mathrm{W_E}^{\circ}\right)\mathrm{T_E}^{\substack{\circ\circ\\\bullet\circ\\\circ\circ}}\right)$$

$$\frac{d\mathrm{T_E}^{\substack{\circ\circ\\\bullet\circ\\\circ\bullet}}}{dt} = \underset{\mathrm{ATPoE}}{\nu_f}\left(\mathrm{T_O}^{\substack{\circ\circ\\\bullet\circ\\\circ\bullet}}\right) + \underset{\mathrm{ASE}}{\nu_r}\left(\left(\frac{1}{4}\left(\mathrm{P_E}^{\substack{\bullet\\\circ\\\circ\\\circ}}+\mathrm{P_E}^{\substack{\circ\\\bullet\\\circ\\\circ}}+\mathrm{P_E}^{\substack{\circ\\\circ\\\bullet\\\circ}}+\mathrm{P_E}^{\substack{\circ\\\circ\\\circ\\\bullet}}\right)+\frac{1}{6}\left(\mathrm{P_E}^{\substack{\bullet\\\bullet\\\circ\\\circ}}+\mathrm{P_E}^{\substack{\bullet\\\circ\\\bullet\\\circ}}+\mathrm{P_E}^{\substack{\bullet\\\circ\\\circ\\\bullet}}+\mathrm{P_E}^{\substack{\circ\\\bullet\\\bullet\\\circ}}+\mathrm{P_E}^{\substack{\circ\\\bullet\\\circ\\\bullet}}+\mathrm{P_E}^{\substack{\circ\\\circ\\\bullet\\\bullet}}\right)\right)\mathrm{D_E}^{\substack{\circ\\\bullet\\\circ}}\right) - \underset{\mathrm{ASE}}{\nu_f}\left(\left(\mathrm{W_E}^{\bullet}+\mathrm{W_E}^{\circ}\right)\mathrm{T_E}^{\substack{\circ\circ\\\bullet\circ\\\circ\bullet}}\right)$$

$$\frac{d\mathrm{T_E}^{\substack{\circ\circ\\\bullet\bullet\\\circ\circ}}}{dt} = \underset{\mathrm{ATPoE}}{\nu_f}\left(\mathrm{T_O}^{\substack{\circ\circ\\\bullet\bullet\\\circ\circ}}\right) + \underset{\mathrm{ASE}}{\nu_r}\left(\left(\frac{1}{4}\left(\mathrm{P_E}^{\substack{\bullet\\\circ\\\circ\\\circ}}+\mathrm{P_E}^{\substack{\circ\\\bullet\\\circ\\\circ}}+\mathrm{P_E}^{\substack{\circ\\\circ\\\bullet\\\circ}}+\mathrm{P_E}^{\substack{\circ\\\circ\\\circ\\\bullet}}\right)+\frac{1}{6}\left(\mathrm{P_E}^{\substack{\bullet\\\bullet\\\circ\\\circ}}+\mathrm{P_E}^{\substack{\bullet\\\circ\\\bullet\\\circ}}+\mathrm{P_E}^{\substack{\bullet\\\circ\\\circ\\\bullet}}+\mathrm{P_E}^{\substack{\circ\\\bullet\\\bullet\\\circ}}+\mathrm{P_E}^{\substack{\circ\\\bullet\\\circ\\\bullet}}+\mathrm{P_E}^{\substack{\circ\\\circ\\\bullet\\\bullet}}\right)\right)\mathrm{D_E}^{\substack{\circ\\\bullet\\\circ}}\right) - \underset{\mathrm{ASE}}{\nu_f}\left(\left(\mathrm{W_E}^{\bullet}+\mathrm{W_E}^{\circ}\right)\mathrm{T_E}^{\substack{\circ\circ\\\bullet\bullet\\\circ\circ}}\right)$$

$$\frac{d\mathrm{T_E}^{\substack{\circ\circ\\\bullet\bullet\\\circ\bullet}}}{dt} = \underset{\mathrm{ATPoE}}{\nu_f}\left(\mathrm{T_O}^{\substack{\circ\circ\\\bullet\bullet\\\circ\bullet}}\right) + \underset{\mathrm{ASE}}{\nu_r}\left(\left(\frac{1}{4}\left(\mathrm{P_E}^{\substack{\bullet\\\bullet\\\bullet\\\circ}}+\mathrm{P_E}^{\substack{\bullet\\\bullet\\\circ\\\bullet}}+\mathrm{P_E}^{\substack{\bullet\\\circ\\\bullet\\\bullet}}+\mathrm{P_E}^{\substack{\circ\\\bullet\\\bullet\\\bullet}}\right)+\frac{1}{6}\left(\mathrm{P_E}^{\substack{\bullet\\\bullet\\\circ\\\circ}}+\mathrm{P_E}^{\substack{\bullet\\\circ\\\bullet\\\circ}}+\mathrm{P_E}^{\substack{\bullet\\\circ\\\circ\\\bullet}}+\mathrm{P_E}^{\substack{\circ\\\bullet\\\bullet\\\circ}}+\mathrm{P_E}^{\substack{\circ\\\bullet\\\circ\\\bullet}}+\mathrm{P_E}^{\substack{\circ\\\circ\\\bullet\\\bullet}}\right)\right)\mathrm{D_E}^{\substack{\circ\\\bullet\\\circ}}\right) - \underset{\mathrm{ASE}}{\nu_f}\left(\left(\mathrm{W_E}^{\bullet}+\mathrm{W_E}^{\circ}\right)\mathrm{T_E}^{\substack{\circ\circ\\\bullet\bullet\\\circ\bullet}}\right)$$

$$\frac{d\mathrm{T_E}^{\substack{\circ\bullet\\\bullet\circ\\\circ\circ}}}{dt} = \underset{\mathrm{ATPoE}}{\nu_f}\left(\mathrm{T_O}^{\substack{\circ\bullet\\\bullet\circ\\\circ\circ}}\right) + \underset{\mathrm{ASE}}{\nu_r}\left(\left(\frac{1}{4}\left(\mathrm{P_E}^{\substack{\bullet\\\circ\\\circ\\\circ}}+\mathrm{P_E}^{\substack{\circ\\\bullet\\\circ\\\circ}}+\mathrm{P_E}^{\substack{\circ\\\circ\\\bullet\\\circ}}+\mathrm{P_E}^{\substack{\circ\\\circ\\\circ\\\bullet}}\right)+\frac{1}{6}\left(\mathrm{P_E}^{\substack{\bullet\\\bullet\\\circ\\\circ}}+\mathrm{P_E}^{\substack{\bullet\\\circ\\\bullet\\\circ}}+\mathrm{P_E}^{\substack{\bullet\\\circ\\\circ\\\bullet}}+\mathrm{P_E}^{\substack{\circ\\\bullet\\\bullet\\\circ}}+\mathrm{P_E}^{\substack{\circ\\\bullet\\\circ\\\bullet}}+\mathrm{P_E}^{\substack{\circ\\\circ\\\bullet\\\bullet}}\right)\right)\mathrm{D_E}^{\substack{\circ\\\bullet\\\circ}}\right) - \underset{\mathrm{ASE}}{\nu_f}\left(\left(\mathrm{W_E}^{\bullet}+\mathrm{W_E}^{\circ}\right)\mathrm{T_E}^{\substack{\circ\bullet\\\bullet\circ\\\circ\circ}}\right)$$

$$\frac{d\mathrm{T_E}^{\substack{\circ\bullet\\\bullet\circ\\\circ\bullet}}}{dt} = \underset{\mathrm{ATPoE}}{\nu_f}\left(\mathrm{T_O}^{\substack{\circ\bullet\\\bullet\circ\\\circ\bullet}}\right) + \underset{\mathrm{ASE}}{\nu_r}\left(\left(\frac{1}{4}\left(\mathrm{P_E}^{\substack{\bullet\\\bullet\\\bullet\\\circ}}+\mathrm{P_E}^{\substack{\bullet\\\bullet\\\circ\\\bullet}}+\mathrm{P_E}^{\substack{\bullet\\\circ\\\bullet\\\bullet}}+\mathrm{P_E}^{\substack{\circ\\\bullet\\\bullet\\\bullet}}\right)+\frac{1}{6}\left(\mathrm{P_E}^{\substack{\bullet\\\bullet\\\circ\\\circ}}+\mathrm{P_E}^{\substack{\bullet\\\circ\\\bullet\\\circ}}+\mathrm{P_E}^{\substack{\bullet\\\circ\\\circ\\\bullet}}+\mathrm{P_E}^{\substack{\circ\\\bullet\\\bullet\\\circ}}+\mathrm{P_E}^{\substack{\circ\\\bullet\\\circ\\\bullet}}+\mathrm{P_E}^{\substack{\circ\\\circ\\\bullet\\\bullet}}\right)\right)\mathrm{D_E}^{\substack{\circ\\\bullet\\\circ}}\right) - \underset{\mathrm{ASE}}{\nu_f}\left(\left(\mathrm{W_E}^{\bullet}+\mathrm{W_E}^{\circ}\right)\mathrm{T_E}^{\substack{\circ\bullet\\\bullet\circ\\\circ\bullet}}\right)$$

$$\frac{d\mathrm{T}_{\mathrm{E}}{\substack{\circ\bullet\\\bullet\bullet\\\circ\circ}}}{dt}=\overset{\nu_f}{\mathrm{ATPoE}}\big(\mathrm{T}_{\mathrm{O}}{\substack{\circ\bullet\\\bullet\bullet\\\circ\circ}}\big)+\overset{\nu_r}{\mathrm{ASE}}\Big(\Big(\frac{1}{4}\big(\mathrm{P}_{\mathrm{E}}{\substack{\bullet\\\bullet\\\bullet\\\circ}}+\mathrm{P}_{\mathrm{E}}{\substack{\bullet\\\bullet\\\circ\\\bullet}}+\mathrm{P}_{\mathrm{E}}{\substack{\bullet\\\circ\\\bullet\\\bullet}}+\mathrm{P}_{\mathrm{E}}{\substack{\circ\\\bullet\\\bullet\\\bullet}}\big)+\frac{1}{6}\big(\mathrm{P}_{\mathrm{E}}{\substack{\bullet\\\bullet\\\circ\\\circ}}+\mathrm{P}_{\mathrm{E}}{\substack{\bullet\\\circ\\\bullet\\\circ}}+\mathrm{P}_{\mathrm{E}}{\substack{\bullet\\\circ\\\circ\\\bullet}}+\mathrm{P}_{\mathrm{E}}{\substack{\circ\\\bullet\\\bullet\\\circ}}+\mathrm{P}_{\mathrm{E}}{\substack{\circ\\\bullet\\\circ\\\bullet}}+\mathrm{P}_{\mathrm{E}}{\substack{\circ\\\circ\\\bullet\\\bullet}}\big)\Big)\mathrm{D}_{\mathrm{E}}{\substack{\circ\\\bullet\\\circ}}\Big)-\overset{\nu_f}{\mathrm{ASE}}\Big(\big(\mathrm{W}_{\mathrm{E}}{}^{\bullet}+\mathrm{W}_{\mathrm{E}}{}^{\circ}\big)\mathrm{T}_{\mathrm{E}}{\substack{\circ\bullet\\\bullet\bullet\\\circ\circ}}\Big)$$

$$\frac{d\mathrm{T}_{\mathrm{E}}{\substack{\circ\bullet\\\bullet\bullet\\\circ\bullet}}}{dt}=\overset{\nu_f}{\mathrm{ATPoE}}\big(\mathrm{T}_{\mathrm{O}}{\substack{\circ\bullet\\\bullet\bullet\\\circ\bullet}}\big)+\overset{\nu_r}{\mathrm{ASE}}\Big(\Big(\mathrm{P}_{\mathrm{E}}{\substack{\bullet\\\bullet\\\bullet\\\bullet}}+\frac{1}{4}\big(\mathrm{P}_{\mathrm{E}}{\substack{\bullet\\\bullet\\\bullet\\\circ}}+\mathrm{P}_{\mathrm{E}}{\substack{\bullet\\\bullet\\\circ\\\bullet}}+\mathrm{P}_{\mathrm{E}}{\substack{\bullet\\\circ\\\bullet\\\bullet}}+\mathrm{P}_{\mathrm{E}}{\substack{\circ\\\bullet\\\bullet\\\bullet}}\big)\Big)\mathrm{D}_{\mathrm{E}}{\substack{\circ\\\bullet\\\circ}}\Big)-\overset{\nu_f}{\mathrm{ASE}}\Big(\big(\mathrm{W}_{\mathrm{E}}{}^{\bullet}+\mathrm{W}_{\mathrm{E}}{}^{\circ}\big)\mathrm{T}_{\mathrm{E}}{\substack{\circ\bullet\\\bullet\bullet\\\circ\bullet}}\Big)$$

$$\frac{d\mathrm{T}_{\mathrm{E}}{\substack{\circ\circ\\\bullet\circ\\\bullet\circ}}}{dt}=\overset{\nu_f}{\mathrm{ATPoE}}\big(\mathrm{T}_{\mathrm{O}}{\substack{\circ\circ\\\bullet\circ\\\bullet\circ}}\big)+\overset{\nu_r}{\mathrm{ASE}}\Big(\Big(\mathrm{P}_{\mathrm{E}}{\substack{\circ\\\circ\\\circ\\\circ}}+\frac{1}{4}\big(\mathrm{P}_{\mathrm{E}}{\substack{\bullet\\\circ\\\circ\\\circ}}+\mathrm{P}_{\mathrm{E}}{\substack{\circ\\\bullet\\\circ\\\circ}}+\mathrm{P}_{\mathrm{E}}{\substack{\circ\\\circ\\\bullet\\\circ}}+\mathrm{P}_{\mathrm{E}}{\substack{\circ\\\circ\\\circ\\\bullet}}\big)\Big)\mathrm{D}_{\mathrm{E}}{\substack{\circ\\\bullet\\\bullet}}\Big)-\overset{\nu_f}{\mathrm{ASE}}\Big(\big(\mathrm{W}_{\mathrm{E}}{}^{\bullet}+\mathrm{W}_{\mathrm{E}}{}^{\circ}\big)\mathrm{T}_{\mathrm{E}}{\substack{\circ\circ\\\bullet\circ\\\bullet\circ}}\Big)$$

$$\frac{d\mathrm{T}_{\mathrm{E}}{\substack{\circ\circ\\\bullet\circ\\\bullet\bullet}}}{dt}=\overset{\nu_f}{\mathrm{ATPoE}}\big(\mathrm{T}_{\mathrm{O}}{\substack{\circ\circ\\\bullet\circ\\\bullet\bullet}}\big)+\overset{\nu_r}{\mathrm{ASE}}\Big(\Big(\frac{1}{4}\big(\mathrm{P}_{\mathrm{E}}{\substack{\bullet\\\circ\\\circ\\\circ}}+\mathrm{P}_{\mathrm{E}}{\substack{\circ\\\bullet\\\circ\\\circ}}+\mathrm{P}_{\mathrm{E}}{\substack{\circ\\\circ\\\bullet\\\circ}}+\mathrm{P}_{\mathrm{E}}{\substack{\circ\\\circ\\\circ\\\bullet}}\big)+\frac{1}{6}\big(\mathrm{P}_{\mathrm{E}}{\substack{\bullet\\\bullet\\\circ\\\circ}}+\mathrm{P}_{\mathrm{E}}{\substack{\bullet\\\circ\\\bullet\\\circ}}+\mathrm{P}_{\mathrm{E}}{\substack{\bullet\\\circ\\\circ\\\bullet}}+\mathrm{P}_{\mathrm{E}}{\substack{\circ\\\bullet\\\bullet\\\circ}}+\mathrm{P}_{\mathrm{E}}{\substack{\circ\\\bullet\\\circ\\\bullet}}+\mathrm{P}_{\mathrm{E}}{\substack{\circ\\\circ\\\bullet\\\bullet}}\big)\Big)\mathrm{D}_{\mathrm{E}}{\substack{\circ\\\bullet\\\bullet}}\Big)-\overset{\nu_f}{\mathrm{ASE}}\Big(\big(\mathrm{W}_{\mathrm{E}}{}^{\bullet}+\mathrm{W}_{\mathrm{E}}{}^{\circ}\big)\mathrm{T}_{\mathrm{E}}{\substack{\circ\circ\\\bullet\circ\\\bullet\bullet}}\Big)$$

$$\frac{d\mathrm{T}_{\mathrm{E}}{\substack{\circ\circ\\\bullet\bullet\\\bullet\circ}}}{dt}=\overset{\nu_f}{\mathrm{ATPoE}}\big(\mathrm{T}_{\mathrm{O}}{\substack{\circ\circ\\\bullet\bullet\\\bullet\circ}}\big)+\overset{\nu_r}{\mathrm{ASE}}\Big(\Big(\frac{1}{4}\big(\mathrm{P}_{\mathrm{E}}{\substack{\bullet\\\circ\\\circ\\\circ}}+\mathrm{P}_{\mathrm{E}}{\substack{\circ\\\bullet\\\circ\\\circ}}+\mathrm{P}_{\mathrm{E}}{\substack{\circ\\\circ\\\bullet\\\circ}}+\mathrm{P}_{\mathrm{E}}{\substack{\circ\\\circ\\\circ\\\bullet}}\big)+\frac{1}{6}\big(\mathrm{P}_{\mathrm{E}}{\substack{\bullet\\\bullet\\\circ\\\circ}}+\mathrm{P}_{\mathrm{E}}{\substack{\bullet\\\circ\\\bullet\\\circ}}+\mathrm{P}_{\mathrm{E}}{\substack{\bullet\\\circ\\\circ\\\bullet}}+\mathrm{P}_{\mathrm{E}}{\substack{\circ\\\bullet\\\bullet\\\circ}}+\mathrm{P}_{\mathrm{E}}{\substack{\circ\\\bullet\\\circ\\\bullet}}+\mathrm{P}_{\mathrm{E}}{\substack{\circ\\\circ\\\bullet\\\bullet}}\big)\Big)\mathrm{D}_{\mathrm{E}}{\substack{\circ\\\bullet\\\bullet}}\Big)-\overset{\nu_f}{\mathrm{ASE}}\Big(\big(\mathrm{W}_{\mathrm{E}}{}^{\bullet}+\mathrm{W}_{\mathrm{E}}{}^{\circ}\big)\mathrm{T}_{\mathrm{E}}{\substack{\circ\circ\\\bullet\bullet\\\bullet\circ}}\Big)$$

$$\frac{d\mathrm{T}_{\mathrm{E}}{\substack{\circ\circ\\\bullet\bullet\\\bullet\bullet}}}{dt}=\overset{\nu_f}{\mathrm{ATPoE}}\big(\mathrm{T}_{\mathrm{O}}{\substack{\circ\circ\\\bullet\bullet\\\bullet\bullet}}\big)+\overset{\nu_r}{\mathrm{ASE}}\Big(\Big(\frac{1}{4}\big(\mathrm{P}_{\mathrm{E}}{\substack{\bullet\\\bullet\\\bullet\\\circ}}+\mathrm{P}_{\mathrm{E}}{\substack{\bullet\\\bullet\\\circ\\\bullet}}+\mathrm{P}_{\mathrm{E}}{\substack{\bullet\\\circ\\\bullet\\\bullet}}+\mathrm{P}_{\mathrm{E}}{\substack{\circ\\\bullet\\\bullet\\\bullet}}\big)+\frac{1}{6}\big(\mathrm{P}_{\mathrm{E}}{\substack{\bullet\\\bullet\\\circ\\\circ}}+\mathrm{P}_{\mathrm{E}}{\substack{\bullet\\\circ\\\bullet\\\circ}}+\mathrm{P}_{\mathrm{E}}{\substack{\bullet\\\circ\\\circ\\\bullet}}+\mathrm{P}_{\mathrm{E}}{\substack{\circ\\\bullet\\\bullet\\\circ}}+\mathrm{P}_{\mathrm{E}}{\substack{\circ\\\bullet\\\circ\\\bullet}}+\mathrm{P}_{\mathrm{E}}{\substack{\circ\\\circ\\\bullet\\\bullet}}\big)\Big)\mathrm{D}_{\mathrm{E}}{\substack{\circ\\\bullet\\\bullet}}\Big)-\overset{\nu_f}{\mathrm{ASE}}\Big(\big(\mathrm{W}_{\mathrm{E}}{}^{\bullet}+\mathrm{W}_{\mathrm{E}}{}^{\circ}\big)\mathrm{T}_{\mathrm{E}}{\substack{\circ\circ\\\bullet\bullet\\\bullet\bullet}}\Big)$$

$$\frac{d\mathrm{T}_{\mathrm{E}}{\substack{\circ\bullet\\\bullet\circ\\\bullet\circ}}}{dt}=\overset{\nu_f}{\mathrm{ATPoE}}\big(\mathrm{T}_{\mathrm{O}}{\substack{\circ\bullet\\\bullet\circ\\\bullet\circ}}\big)+\overset{\nu_r}{\mathrm{ASE}}\Big(\Big(\frac{1}{4}\big(\mathrm{P}_{\mathrm{E}}{\substack{\bullet\\\circ\\\circ\\\circ}}+\mathrm{P}_{\mathrm{E}}{\substack{\circ\\\bullet\\\circ\\\circ}}+\mathrm{P}_{\mathrm{E}}{\substack{\circ\\\circ\\\bullet\\\circ}}+\mathrm{P}_{\mathrm{E}}{\substack{\circ\\\circ\\\circ\\\bullet}}\big)+\frac{1}{6}\big(\mathrm{P}_{\mathrm{E}}{\substack{\bullet\\\bullet\\\circ\\\circ}}+\mathrm{P}_{\mathrm{E}}{\substack{\bullet\\\circ\\\bullet\\\circ}}+\mathrm{P}_{\mathrm{E}}{\substack{\bullet\\\circ\\\circ\\\bullet}}+\mathrm{P}_{\mathrm{E}}{\substack{\circ\\\bullet\\\bullet\\\circ}}+\mathrm{P}_{\mathrm{E}}{\substack{\circ\\\bullet\\\circ\\\bullet}}+\mathrm{P}_{\mathrm{E}}{\substack{\circ\\\circ\\\bullet\\\bullet}}\big)\Big)\mathrm{D}_{\mathrm{E}}{\substack{\circ\\\bullet\\\bullet}}\Big)-\overset{\nu_f}{\mathrm{ASE}}\Big(\big(\mathrm{W}_{\mathrm{E}}{}^{\bullet}+\mathrm{W}_{\mathrm{E}}{}^{\circ}\big)\mathrm{T}_{\mathrm{E}}{\substack{\circ\bullet\\\bullet\circ\\\bullet\circ}}\Big)$$

$$\frac{d\mathrm{T}_{\mathrm{E}}{\substack{\circ\bullet\\\bullet\circ\\\bullet\bullet}}}{dt}=\overset{\nu_f}{\mathrm{ATPoE}}\big(\mathrm{T}_{\mathrm{O}}{\substack{\circ\bullet\\\bullet\circ\\\bullet\bullet}}\big)+\overset{\nu_r}{\mathrm{ASE}}\Big(\Big(\frac{1}{4}\big(\mathrm{P}_{\mathrm{E}}{\substack{\bullet\\\bullet\\\bullet\\\circ}}+\mathrm{P}_{\mathrm{E}}{\substack{\bullet\\\bullet\\\circ\\\bullet}}+\mathrm{P}_{\mathrm{E}}{\substack{\bullet\\\circ\\\bullet\\\bullet}}+\mathrm{P}_{\mathrm{E}}{\substack{\circ\\\bullet\\\bullet\\\bullet}}\big)+\frac{1}{6}\big(\mathrm{P}_{\mathrm{E}}{\substack{\bullet\\\bullet\\\circ\\\circ}}+\mathrm{P}_{\mathrm{E}}{\substack{\bullet\\\circ\\\bullet\\\circ}}+\mathrm{P}_{\mathrm{E}}{\substack{\bullet\\\circ\\\circ\\\bullet}}+\mathrm{P}_{\mathrm{E}}{\substack{\circ\\\bullet\\\bullet\\\circ}}+\mathrm{P}_{\mathrm{E}}{\substack{\circ\\\bullet\\\circ\\\bullet}}+\mathrm{P}_{\mathrm{E}}{\substack{\circ\\\circ\\\bullet\\\bullet}}\big)\Big)\mathrm{D}_{\mathrm{E}}{\substack{\circ\\\bullet\\\bullet}}\Big)-\overset{\nu_f}{\mathrm{ASE}}\Big(\big(\mathrm{W}_{\mathrm{E}}{}^{\bullet}+\mathrm{W}_{\mathrm{E}}{}^{\circ}\big)\mathrm{T}_{\mathrm{E}}{\substack{\circ\bullet\\\bullet\circ\\\bullet\bullet}}\Big)$$

$$\frac{d\mathrm{T}_{\mathrm{E}}{\substack{\circ\bullet\\\bullet\bullet\\\bullet\circ}}}{dt}=\overset{\nu_f}{\mathrm{ATPoE}}\big(\mathrm{T}_{\mathrm{O}}{\substack{\circ\bullet\\\bullet\bullet\\\bullet\circ}}\big)+\overset{\nu_r}{\mathrm{ASE}}\Big(\Big(\frac{1}{4}\big(\mathrm{P}_{\mathrm{E}}{\substack{\bullet\\\bullet\\\bullet\\\circ}}+\mathrm{P}_{\mathrm{E}}{\substack{\bullet\\\bullet\\\circ\\\bullet}}+\mathrm{P}_{\mathrm{E}}{\substack{\bullet\\\circ\\\bullet\\\bullet}}+\mathrm{P}_{\mathrm{E}}{\substack{\circ\\\bullet\\\bullet\\\bullet}}\big)+\frac{1}{6}\big(\mathrm{P}_{\mathrm{E}}{\substack{\bullet\\\bullet\\\circ\\\circ}}+\mathrm{P}_{\mathrm{E}}{\substack{\bullet\\\circ\\\bullet\\\circ}}+\mathrm{P}_{\mathrm{E}}{\substack{\bullet\\\circ\\\circ\\\bullet}}+\mathrm{P}_{\mathrm{E}}{\substack{\circ\\\bullet\\\bullet\\\circ}}+\mathrm{P}_{\mathrm{E}}{\substack{\circ\\\bullet\\\circ\\\bullet}}+\mathrm{P}_{\mathrm{E}}{\substack{\circ\\\circ\\\bullet\\\bullet}}\big)\Big)\mathrm{D}_{\mathrm{E}}{\substack{\circ\\\bullet\\\bullet}}\Big)-\overset{\nu_f}{\mathrm{ASE}}\Big(\big(\mathrm{W}_{\mathrm{E}}{}^{\bullet}+\mathrm{W}_{\mathrm{E}}{}^{\circ}\big)\mathrm{T}_{\mathrm{E}}{\substack{\circ\bullet\\\bullet\bullet\\\bullet\circ}}\Big)$$

$$\frac{d\mathrm{T}_{\mathrm{E}}{\substack{\circ\bullet\\\bullet\bullet\\\bullet\bullet}}}{dt}=\overset{\nu_f}{\mathrm{ATPoE}}\big(\mathrm{T}_{\mathrm{O}}{\substack{\circ\bullet\\\bullet\bullet\\\bullet\bullet}}\big)+\overset{\nu_r}{\mathrm{ASE}}\Big(\Big(\mathrm{P}_{\mathrm{E}}{\substack{\bullet\\\bullet\\\bullet\\\bullet}}+\frac{1}{4}\big(\mathrm{P}_{\mathrm{E}}{\substack{\bullet\\\bullet\\\bullet\\\circ}}+\mathrm{P}_{\mathrm{E}}{\substack{\bullet\\\bullet\\\circ\\\bullet}}+\mathrm{P}_{\mathrm{E}}{\substack{\bullet\\\circ\\\bullet\\\bullet}}+\mathrm{P}_{\mathrm{E}}{\substack{\circ\\\bullet\\\bullet\\\bullet}}\big)\Big)\mathrm{D}_{\mathrm{E}}{\substack{\circ\\\bullet\\\bullet}}\Big)-\overset{\nu_f}{\mathrm{ASE}}\Big(\big(\mathrm{W}_{\mathrm{E}}{}^{\bullet}+\mathrm{W}_{\mathrm{E}}{}^{\circ}\big)\mathrm{T}_{\mathrm{E}}{\substack{\circ\bullet\\\bullet\bullet\\\bullet\bullet}}\Big)$$

$$\frac{d\mathrm{T}_{\mathrm{E}}{\substack{\bullet\circ\\\circ\circ\\\circ\circ}}}{dt}=\overset{\nu_f}{\mathrm{ATPoE}}\big(\mathrm{T}_{\mathrm{O}}{\substack{\bullet\circ\\\circ\circ\\\circ\circ}}\big)+\overset{\nu_r}{\mathrm{ASE}}\Big(\Big(\mathrm{P}_{\mathrm{E}}{\substack{\circ\\\circ\\\circ\\\circ}}+\frac{1}{4}\big(\mathrm{P}_{\mathrm{E}}{\substack{\bullet\\\circ\\\circ\\\circ}}+\mathrm{P}_{\mathrm{E}}{\substack{\circ\\\bullet\\\circ\\\circ}}+\mathrm{P}_{\mathrm{E}}{\substack{\circ\\\circ\\\bullet\\\circ}}+\mathrm{P}_{\mathrm{E}}{\substack{\circ\\\circ\\\circ\\\bullet}}\big)\Big)\mathrm{D}_{\mathrm{E}}{\substack{\bullet\\\circ\\\circ}}\Big)-\overset{\nu_f}{\mathrm{ASE}}\Big(\big(\mathrm{W}_{\mathrm{E}}{}^{\bullet}+\mathrm{W}_{\mathrm{E}}{}^{\circ}\big)\mathrm{T}_{\mathrm{E}}{\substack{\bullet\circ\\\circ\circ\\\circ\circ}}\Big)$$

$$\frac{d\mathrm{T}_{\mathrm{E}}{\substack{\bullet\circ\\\circ\circ\\\circ\bullet}}}{dt}=\overset{\nu_f}{\mathrm{ATPoE}}\big(\mathrm{T}_{\mathrm{O}}{\substack{\bullet\circ\\\circ\circ\\\circ\bullet}}\big)+\overset{\nu_r}{\mathrm{ASE}}\Big(\Big(\frac{1}{4}\big(\mathrm{P}_{\mathrm{E}}{\substack{\bullet\\\circ\\\circ\\\circ}}+\mathrm{P}_{\mathrm{E}}{\substack{\circ\\\bullet\\\circ\\\circ}}+\mathrm{P}_{\mathrm{E}}{\substack{\circ\\\circ\\\bullet\\\circ}}+\mathrm{P}_{\mathrm{E}}{\substack{\circ\\\circ\\\circ\\\bullet}}\big)+\frac{1}{6}\big(\mathrm{P}_{\mathrm{E}}{\substack{\bullet\\\bullet\\\circ\\\circ}}+\mathrm{P}_{\mathrm{E}}{\substack{\bullet\\\circ\\\bullet\\\circ}}+\mathrm{P}_{\mathrm{E}}{\substack{\bullet\\\circ\\\circ\\\bullet}}+\mathrm{P}_{\mathrm{E}}{\substack{\circ\\\bullet\\\bullet\\\circ}}+\mathrm{P}_{\mathrm{E}}{\substack{\circ\\\bullet\\\circ\\\bullet}}+\mathrm{P}_{\mathrm{E}}{\substack{\circ\\\circ\\\bullet\\\bullet}}\big)\Big)\mathrm{D}_{\mathrm{E}}{\substack{\bullet\\\circ\\\circ}}\Big)-\overset{\nu_f}{\mathrm{ASE}}\Big(\big(\mathrm{W}_{\mathrm{E}}{}^{\bullet}+\mathrm{W}_{\mathrm{E}}{}^{\circ}\big)\mathrm{T}_{\mathrm{E}}{\substack{\bullet\circ\\\circ\circ\\\circ\bullet}}\Big)$$

$$\frac{d\mathrm{T}_{\mathrm{E}}{\substack{\bullet\circ\\\circ\bullet\\\circ\circ}}}{dt}=\overset{\nu_f}{\mathrm{ATPoE}}\big(\mathrm{T}_{\mathrm{O}}{\substack{\bullet\circ\\\circ\bullet\\\circ\circ}}\big)+\overset{\nu_r}{\mathrm{ASE}}\Big(\Big(\frac{1}{4}\big(\mathrm{P}_{\mathrm{E}}{\substack{\bullet\\\circ\\\circ\\\circ}}+\mathrm{P}_{\mathrm{E}}{\substack{\circ\\\bullet\\\circ\\\circ}}+\mathrm{P}_{\mathrm{E}}{\substack{\circ\\\circ\\\bullet\\\circ}}+\mathrm{P}_{\mathrm{E}}{\substack{\circ\\\circ\\\circ\\\bullet}}\big)+\frac{1}{6}\big(\mathrm{P}_{\mathrm{E}}{\substack{\bullet\\\bullet\\\circ\\\circ}}+\mathrm{P}_{\mathrm{E}}{\substack{\bullet\\\circ\\\bullet\\\circ}}+\mathrm{P}_{\mathrm{E}}{\substack{\bullet\\\circ\\\circ\\\bullet}}+\mathrm{P}_{\mathrm{E}}{\substack{\circ\\\bullet\\\bullet\\\circ}}+\mathrm{P}_{\mathrm{E}}{\substack{\circ\\\bullet\\\circ\\\bullet}}+\mathrm{P}_{\mathrm{E}}{\substack{\circ\\\circ\\\bullet\\\bullet}}\big)\Big)\mathrm{D}_{\mathrm{E}}{\substack{\bullet\\\circ\\\circ}}\Big)-\overset{\nu_f}{\mathrm{ASE}}\Big(\big(\mathrm{W}_{\mathrm{E}}{}^{\bullet}+\mathrm{W}_{\mathrm{E}}{}^{\circ}\big)\mathrm{T}_{\mathrm{E}}{\substack{\bullet\circ\\\circ\bullet\\\circ\circ}}\Big)$$

$$\frac{d\mathrm{T}_{\mathrm{E}}{\substack{\bullet\circ\\\circ\bullet\\\circ\bullet}}}{dt}=\overset{\nu_f}{\mathrm{ATPoE}}\big(\mathrm{T}_{\mathrm{O}}{\substack{\bullet\circ\\\circ\bullet\\\circ\bullet}}\big)+\overset{\nu_r}{\mathrm{ASE}}\Big(\Big(\frac{1}{4}\big(\mathrm{P}_{\mathrm{E}}{\substack{\bullet\\\bullet\\\bullet\\\circ}}+\mathrm{P}_{\mathrm{E}}{\substack{\bullet\\\bullet\\\circ\\\bullet}}+\mathrm{P}_{\mathrm{E}}{\substack{\bullet\\\circ\\\bullet\\\bullet}}+\mathrm{P}_{\mathrm{E}}{\substack{\circ\\\bullet\\\bullet\\\bullet}}\big)+\frac{1}{6}\big(\mathrm{P}_{\mathrm{E}}{\substack{\bullet\\\bullet\\\circ\\\circ}}+\mathrm{P}_{\mathrm{E}}{\substack{\bullet\\\circ\\\bullet\\\circ}}+\mathrm{P}_{\mathrm{E}}{\substack{\bullet\\\circ\\\circ\\\bullet}}+\mathrm{P}_{\mathrm{E}}{\substack{\circ\\\bullet\\\bullet\\\circ}}+\mathrm{P}_{\mathrm{E}}{\substack{\circ\\\bullet\\\circ\\\bullet}}+\mathrm{P}_{\mathrm{E}}{\substack{\circ\\\circ\\\bullet\\\bullet}}\big)\Big)\mathrm{D}_{\mathrm{E}}{\substack{\bullet\\\circ\\\circ}}\Big)-\overset{\nu_f}{\mathrm{ASE}}\Big(\big(\mathrm{W}_{\mathrm{E}}{}^{\bullet}+\mathrm{W}_{\mathrm{E}}{}^{\circ}\big)\mathrm{T}_{\mathrm{E}}{\substack{\bullet\circ\\\circ\bullet\\\circ\bullet}}\Big)$$

$$\frac{d\mathrm{T}_{\mathrm{E}}{\substack{\bullet\bullet\\\circ\circ\\\circ\circ}}}{dt}=\overset{\nu_f}{\mathrm{ATPoE}}\big(\mathrm{T}_{\mathrm{O}}{\substack{\bullet\bullet\\\circ\circ\\\circ\circ}}\big)+\overset{\nu_r}{\mathrm{ASE}}\Big(\Big(\frac{1}{4}\big(\mathrm{P}_{\mathrm{E}}{\substack{\bullet\\\circ\\\circ\\\circ}}+\mathrm{P}_{\mathrm{E}}{\substack{\circ\\\bullet\\\circ\\\circ}}+\mathrm{P}_{\mathrm{E}}{\substack{\circ\\\circ\\\bullet\\\circ}}+\mathrm{P}_{\mathrm{E}}{\substack{\circ\\\circ\\\circ\\\bullet}}\big)+\frac{1}{6}\big(\mathrm{P}_{\mathrm{E}}{\substack{\bullet\\\bullet\\\circ\\\circ}}+\mathrm{P}_{\mathrm{E}}{\substack{\bullet\\\circ\\\bullet\\\circ}}+\mathrm{P}_{\mathrm{E}}{\substack{\bullet\\\circ\\\circ\\\bullet}}+\mathrm{P}_{\mathrm{E}}{\substack{\circ\\\bullet\\\bullet\\\circ}}+\mathrm{P}_{\mathrm{E}}{\substack{\circ\\\bullet\\\circ\\\bullet}}+\mathrm{P}_{\mathrm{E}}{\substack{\circ\\\circ\\\bullet\\\bullet}}\big)\Big)\mathrm{D}_{\mathrm{E}}{\substack{\bullet\\\circ\\\circ}}\Big)-\overset{\nu_f}{\mathrm{ASE}}\Big(\big(\mathrm{W}_{\mathrm{E}}{}^{\bullet}+\mathrm{W}_{\mathrm{E}}{}^{\circ}\big)\mathrm{T}_{\mathrm{E}}{\substack{\bullet\bullet\\\circ\circ\\\circ\circ}}\Big)$$

$$\frac{d\mathrm{T}_{\mathrm{E}}{\substack{\bullet\bullet\\\circ\circ\\\circ\bullet}}}{dt}=\overset{\nu_f}{\mathrm{ATPoE}}\big(\mathrm{T}_{\mathrm{O}}{\substack{\bullet\bullet\\\circ\circ\\\circ\bullet}}\big)+\overset{\nu_r}{\mathrm{ASE}}\Big(\Big(\frac{1}{4}\big(\mathrm{P}_{\mathrm{E}}{\substack{\bullet\\\bullet\\\bullet\\\circ}}+\mathrm{P}_{\mathrm{E}}{\substack{\bullet\\\bullet\\\circ\\\bullet}}+\mathrm{P}_{\mathrm{E}}{\substack{\bullet\\\circ\\\bullet\\\bullet}}+\mathrm{P}_{\mathrm{E}}{\substack{\circ\\\bullet\\\bullet\\\bullet}}\big)+\frac{1}{6}\big(\mathrm{P}_{\mathrm{E}}{\substack{\bullet\\\bullet\\\circ\\\circ}}+\mathrm{P}_{\mathrm{E}}{\substack{\bullet\\\circ\\\bullet\\\circ}}+\mathrm{P}_{\mathrm{E}}{\substack{\bullet\\\circ\\\circ\\\bullet}}+\mathrm{P}_{\mathrm{E}}{\substack{\circ\\\bullet\\\bullet\\\circ}}+\mathrm{P}_{\mathrm{E}}{\substack{\circ\\\bullet\\\circ\\\bullet}}+\mathrm{P}_{\mathrm{E}}{\substack{\circ\\\circ\\\bullet\\\bullet}}\big)\Big)\mathrm{D}_{\mathrm{E}}{\substack{\bullet\\\circ\\\circ}}\Big)-\overset{\nu_f}{\mathrm{ASE}}\Big(\big(\mathrm{W}_{\mathrm{E}}{}^{\bullet}+\mathrm{W}_{\mathrm{E}}{}^{\circ}\big)\mathrm{T}_{\mathrm{E}}{\substack{\bullet\bullet\\\circ\circ\\\circ\bullet}}\Big)$$

$$\frac{d\mathrm{T}_{\mathrm{E}}{\substack{\bullet\bullet\\\circ\bullet\\\circ\circ}}}{dt}=\overset{\nu_f}{\mathrm{ATPoE}}\big(\mathrm{T}_{\mathrm{O}}{\substack{\bullet\bullet\\\circ\bullet\\\circ\circ}}\big)+\overset{\nu_r}{\mathrm{ASE}}\Big(\Big(\frac{1}{4}\big(\mathrm{P}_{\mathrm{E}}{\substack{\bullet\\\bullet\\\bullet\\\circ}}+\mathrm{P}_{\mathrm{E}}{\substack{\bullet\\\bullet\\\circ\\\bullet}}+\mathrm{P}_{\mathrm{E}}{\substack{\bullet\\\circ\\\bullet\\\bullet}}+\mathrm{P}_{\mathrm{E}}{\substack{\circ\\\bullet\\\bullet\\\bullet}}\big)+\frac{1}{6}\big(\mathrm{P}_{\mathrm{E}}{\substack{\bullet\\\bullet\\\circ\\\circ}}+\mathrm{P}_{\mathrm{E}}{\substack{\bullet\\\circ\\\bullet\\\circ}}+\mathrm{P}_{\mathrm{E}}{\substack{\bullet\\\circ\\\circ\\\bullet}}+\mathrm{P}_{\mathrm{E}}{\substack{\circ\\\bullet\\\bullet\\\circ}}+\mathrm{P}_{\mathrm{E}}{\substack{\circ\\\bullet\\\circ\\\bullet}}+\mathrm{P}_{\mathrm{E}}{\substack{\circ\\\circ\\\bullet\\\bullet}}\big)\Big)\mathrm{D}_{\mathrm{E}}{\substack{\bullet\\\circ\\\circ}}\Big)-\overset{\nu_f}{\mathrm{ASE}}\Big(\big(\mathrm{W}_{\mathrm{E}}{}^{\bullet}+\mathrm{W}_{\mathrm{E}}{}^{\circ}\big)\mathrm{T}_{\mathrm{E}}{\substack{\bullet\bullet\\\circ\bullet\\\circ\circ}}\Big)$$

$$\frac{d\mathrm{T}_{\mathrm{E}}{\substack{\bullet\bullet\\\circ\bullet\\\circ\bullet}}}{dt}=\overset{\nu_f}{\mathrm{ATPoE}}\big(\mathrm{T}_{\mathrm{O}}{\substack{\bullet\bullet\\\circ\bullet\\\circ\bullet}}\big)+\overset{\nu_r}{\mathrm{ASE}}\Big(\Big(\mathrm{P}_{\mathrm{E}}{\substack{\bullet\\\bullet\\\bullet\\\bullet}}+\frac{1}{4}\big(\mathrm{P}_{\mathrm{E}}{\substack{\bullet\\\bullet\\\bullet\\\circ}}+\mathrm{P}_{\mathrm{E}}{\substack{\bullet\\\bullet\\\circ\\\bullet}}+\mathrm{P}_{\mathrm{E}}{\substack{\bullet\\\circ\\\bullet\\\bullet}}+\mathrm{P}_{\mathrm{E}}{\substack{\circ\\\bullet\\\bullet\\\bullet}}\big)\Big)\mathrm{D}_{\mathrm{E}}{\substack{\bullet\\\circ\\\circ}}\Big)-\overset{\nu_f}{\mathrm{ASE}}\Big(\big(\mathrm{W}_{\mathrm{E}}{}^{\bullet}+\mathrm{W}_{\mathrm{E}}{}^{\circ}\big)\mathrm{T}_{\mathrm{E}}{\substack{\bullet\bullet\\\circ\bullet\\\circ\bullet}}\Big)$$

$$\frac{d\mathrm{T}_{\mathrm{E}}{\substack{\bullet\circ\\\circ\circ\\\bullet\circ}}}{dt}=\overset{\nu_f}{\mathrm{ATPoE}}\big(\mathrm{T}_{\mathrm{O}}{\substack{\bullet\circ\\\circ\circ\\\bullet\circ}}\big)+\overset{\nu_r}{\mathrm{ASE}}\Big(\Big(\mathrm{P}_{\mathrm{E}}{\substack{\circ\\\circ\\\circ\\\circ}}+\frac{1}{4}\big(\mathrm{P}_{\mathrm{E}}{\substack{\bullet\\\circ\\\circ\\\circ}}+\mathrm{P}_{\mathrm{E}}{\substack{\circ\\\bullet\\\circ\\\circ}}+\mathrm{P}_{\mathrm{E}}{\substack{\circ\\\circ\\\bullet\\\circ}}+\mathrm{P}_{\mathrm{E}}{\substack{\circ\\\circ\\\circ\\\bullet}}\big)\Big)\mathrm{D}_{\mathrm{E}}{\substack{\bullet\\\circ\\\bullet}}\Big)-\overset{\nu_f}{\mathrm{ASE}}\Big(\big(\mathrm{W}_{\mathrm{E}}{}^{\bullet}+\mathrm{W}_{\mathrm{E}}{}^{\circ}\big)\mathrm{T}_{\mathrm{E}}{\substack{\bullet\circ\\\circ\circ\\\bullet\circ}}\Big)$$

$$\frac{d\mathrm{T}_{\mathrm{E}}{\substack{\bullet\circ\\\circ\circ\\\bullet\bullet}}}{dt}=\overset{\nu_f}{\mathrm{ATPoE}}\big(\mathrm{T}_{\mathrm{O}}{\substack{\bullet\circ\\\circ\circ\\\bullet\bullet}}\big)+\overset{\nu_r}{\mathrm{ASE}}\Big(\Big(\frac{1}{4}\big(\mathrm{P}_{\mathrm{E}}{\substack{\bullet\\\circ\\\circ\\\circ}}+\mathrm{P}_{\mathrm{E}}{\substack{\circ\\\bullet\\\circ\\\circ}}+\mathrm{P}_{\mathrm{E}}{\substack{\circ\\\circ\\\bullet\\\circ}}+\mathrm{P}_{\mathrm{E}}{\substack{\circ\\\circ\\\circ\\\bullet}}\big)+\frac{1}{6}\big(\mathrm{P}_{\mathrm{E}}{\substack{\bullet\\\bullet\\\circ\\\circ}}+\mathrm{P}_{\mathrm{E}}{\substack{\bullet\\\circ\\\bullet\\\circ}}+\mathrm{P}_{\mathrm{E}}{\substack{\bullet\\\circ\\\circ\\\bullet}}+\mathrm{P}_{\mathrm{E}}{\substack{\circ\\\bullet\\\bullet\\\circ}}+\mathrm{P}_{\mathrm{E}}{\substack{\circ\\\bullet\\\circ\\\bullet}}+\mathrm{P}_{\mathrm{E}}{\substack{\circ\\\circ\\\bullet\\\bullet}}\big)\Big)\mathrm{D}_{\mathrm{E}}{\substack{\bullet\\\circ\\\bullet}}\Big)-\overset{\nu_f}{\mathrm{ASE}}\Big(\big(\mathrm{W}_{\mathrm{E}}{}^{\bullet}+\mathrm{W}_{\mathrm{E}}{}^{\circ}\big)\mathrm{T}_{\mathrm{E}}{\substack{\bullet\circ\\\circ\circ\\\bullet\bullet}}\Big)$$

$$\frac{d\mathrm{T}_{\mathrm{E}}{\substack{\bullet\circ\\\circ\bullet\\\bullet\circ}}}{dt}=\overset{\nu_f}{\mathrm{ATPoE}}\big(\mathrm{T}_{\mathrm{O}}{\substack{\bullet\circ\\\circ\bullet\\\bullet\circ}}\big)+\overset{\nu_r}{\mathrm{ASE}}\Big(\Big(\frac{1}{4}\big(\mathrm{P}_{\mathrm{E}}{\substack{\bullet\\\circ\\\circ\\\circ}}+\mathrm{P}_{\mathrm{E}}{\substack{\circ\\\bullet\\\circ\\\circ}}+\mathrm{P}_{\mathrm{E}}{\substack{\circ\\\circ\\\bullet\\\circ}}+\mathrm{P}_{\mathrm{E}}{\substack{\circ\\\circ\\\circ\\\bullet}}\big)+\frac{1}{6}\big(\mathrm{P}_{\mathrm{E}}{\substack{\bullet\\\bullet\\\circ\\\circ}}+\mathrm{P}_{\mathrm{E}}{\substack{\bullet\\\circ\\\bullet\\\circ}}+\mathrm{P}_{\mathrm{E}}{\substack{\bullet\\\circ\\\circ\\\bullet}}+\mathrm{P}_{\mathrm{E}}{\substack{\circ\\\bullet\\\bullet\\\circ}}+\mathrm{P}_{\mathrm{E}}{\substack{\circ\\\bullet\\\circ\\\bullet}}+\mathrm{P}_{\mathrm{E}}{\substack{\circ\\\circ\\\bullet\\\bullet}}\big)\Big)\mathrm{D}_{\mathrm{E}}{\substack{\bullet\\\circ\\\bullet}}\Big)-\overset{\nu_f}{\mathrm{ASE}}\Big(\big(\mathrm{W}_{\mathrm{E}}{}^{\bullet}+\mathrm{W}_{\mathrm{E}}{}^{\circ}\big)\mathrm{T}_{\mathrm{E}}{\substack{\bullet\circ\\\circ\bullet\\\bullet\circ}}\Big)$$

$$\frac{d\mathrm{T_E}\begin{smallmatrix}\bullet&\circ\\\circ&\bullet\\\bullet&\bullet\end{smallmatrix}}{dt} = \underset{\mathrm{ATPoE}}{\nu_f}\big(\mathrm{T_O}\begin{smallmatrix}\bullet&\circ\\\circ&\bullet\\\bullet&\bullet\end{smallmatrix}\big) + \underset{\mathrm{ASE}}{\nu_r}\Big(\Big(\tfrac{1}{4}\big(\mathrm{P_E}\begin{smallmatrix}\bullet\\\bullet\\\bullet\\\circ\end{smallmatrix}+\mathrm{P_E}\begin{smallmatrix}\bullet\\\bullet\\\circ\\\bullet\end{smallmatrix}+\mathrm{P_E}\begin{smallmatrix}\bullet\\\circ\\\bullet\\\bullet\end{smallmatrix}+\mathrm{P_E}\begin{smallmatrix}\circ\\\bullet\\\bullet\\\bullet\end{smallmatrix}\big) + \tfrac{1}{6}\big(\mathrm{P_E}\begin{smallmatrix}\bullet\\\bullet\\\circ\\\circ\end{smallmatrix}+\mathrm{P_E}\begin{smallmatrix}\bullet\\\circ\\\bullet\\\circ\end{smallmatrix}+\mathrm{P_E}\begin{smallmatrix}\bullet\\\circ\\\circ\\\bullet\end{smallmatrix}+\mathrm{P_E}\begin{smallmatrix}\circ\\\bullet\\\bullet\\\circ\end{smallmatrix}+\mathrm{P_E}\begin{smallmatrix}\circ\\\bullet\\\circ\\\bullet\end{smallmatrix}+\mathrm{P_E}\begin{smallmatrix}\circ\\\circ\\\bullet\\\bullet\end{smallmatrix}\big)\Big)\mathrm{D_E}\begin{smallmatrix}\bullet\\\circ\\\bullet\end{smallmatrix}\Big) - \underset{\mathrm{ASE}}{\nu_f}\Big(\big(\mathrm{W_E}\bullet + \mathrm{W_E}\circ\big)\mathrm{T_E}\begin{smallmatrix}\bullet&\circ\\\circ&\bullet\\\bullet&\bullet\end{smallmatrix}\Big)$$

$$\frac{d\mathrm{T_E}\begin{smallmatrix}\bullet&\bullet\\\circ&\circ\\\bullet&\circ\end{smallmatrix}}{dt} = \underset{\mathrm{ATPoE}}{\nu_f}\big(\mathrm{T_O}\begin{smallmatrix}\bullet&\bullet\\\circ&\circ\\\bullet&\circ\end{smallmatrix}\big) + \underset{\mathrm{ASE}}{\nu_r}\Big(\Big(\tfrac{1}{4}\big(\mathrm{P_E}\begin{smallmatrix}\bullet\\\circ\\\circ\\\circ\end{smallmatrix}+\mathrm{P_E}\begin{smallmatrix}\circ\\\bullet\\\circ\\\circ\end{smallmatrix}+\mathrm{P_E}\begin{smallmatrix}\circ\\\circ\\\bullet\\\circ\end{smallmatrix}+\mathrm{P_E}\begin{smallmatrix}\circ\\\circ\\\circ\\\bullet\end{smallmatrix}\big) + \tfrac{1}{6}\big(\mathrm{P_E}\begin{smallmatrix}\bullet\\\bullet\\\circ\\\circ\end{smallmatrix}+\mathrm{P_E}\begin{smallmatrix}\bullet\\\circ\\\bullet\\\circ\end{smallmatrix}+\mathrm{P_E}\begin{smallmatrix}\bullet\\\circ\\\circ\\\bullet\end{smallmatrix}+\mathrm{P_E}\begin{smallmatrix}\circ\\\bullet\\\bullet\\\circ\end{smallmatrix}+\mathrm{P_E}\begin{smallmatrix}\circ\\\bullet\\\circ\\\bullet\end{smallmatrix}+\mathrm{P_E}\begin{smallmatrix}\circ\\\circ\\\bullet\\\bullet\end{smallmatrix}\big)\Big)\mathrm{D_E}\begin{smallmatrix}\bullet\\\circ\\\bullet\end{smallmatrix}\Big) - \underset{\mathrm{ASE}}{\nu_f}\Big(\big(\mathrm{W_E}\bullet + \mathrm{W_E}\circ\big)\mathrm{T_E}\begin{smallmatrix}\bullet&\bullet\\\circ&\circ\\\bullet&\circ\end{smallmatrix}\Big)$$

$$\frac{d\mathrm{T_E}\begin{smallmatrix}\bullet&\bullet\\\circ&\circ\\\bullet&\bullet\end{smallmatrix}}{dt} = \underset{\mathrm{ATPoE}}{\nu_f}\big(\mathrm{T_O}\begin{smallmatrix}\bullet&\bullet\\\circ&\circ\\\bullet&\bullet\end{smallmatrix}\big) + \underset{\mathrm{ASE}}{\nu_r}\Big(\Big(\tfrac{1}{4}\big(\mathrm{P_E}\begin{smallmatrix}\bullet\\\bullet\\\bullet\\\circ\end{smallmatrix}+\mathrm{P_E}\begin{smallmatrix}\bullet\\\bullet\\\circ\\\bullet\end{smallmatrix}+\mathrm{P_E}\begin{smallmatrix}\bullet\\\circ\\\bullet\\\bullet\end{smallmatrix}+\mathrm{P_E}\begin{smallmatrix}\circ\\\bullet\\\bullet\\\bullet\end{smallmatrix}\big) + \tfrac{1}{6}\big(\mathrm{P_E}\begin{smallmatrix}\bullet\\\bullet\\\circ\\\circ\end{smallmatrix}+\mathrm{P_E}\begin{smallmatrix}\bullet\\\circ\\\bullet\\\circ\end{smallmatrix}+\mathrm{P_E}\begin{smallmatrix}\bullet\\\circ\\\circ\\\bullet\end{smallmatrix}+\mathrm{P_E}\begin{smallmatrix}\circ\\\bullet\\\bullet\\\circ\end{smallmatrix}+\mathrm{P_E}\begin{smallmatrix}\circ\\\bullet\\\circ\\\bullet\end{smallmatrix}+\mathrm{P_E}\begin{smallmatrix}\circ\\\circ\\\bullet\\\bullet\end{smallmatrix}\big)\Big)\mathrm{D_E}\begin{smallmatrix}\bullet\\\circ\\\bullet\end{smallmatrix}\Big) - \underset{\mathrm{ASE}}{\nu_f}\Big(\big(\mathrm{W_E}\bullet + \mathrm{W_E}\circ\big)\mathrm{T_E}\begin{smallmatrix}\bullet&\bullet\\\circ&\circ\\\bullet&\bullet\end{smallmatrix}\Big)$$

$$\frac{d\mathrm{T_E}\begin{smallmatrix}\bullet&\bullet\\\circ&\bullet\\\bullet&\circ\end{smallmatrix}}{dt} = \underset{\mathrm{ATPoE}}{\nu_f}\big(\mathrm{T_O}\begin{smallmatrix}\bullet&\bullet\\\circ&\bullet\\\bullet&\circ\end{smallmatrix}\big) + \underset{\mathrm{ASE}}{\nu_r}\Big(\Big(\tfrac{1}{4}\big(\mathrm{P_E}\begin{smallmatrix}\bullet\\\bullet\\\bullet\\\circ\end{smallmatrix}+\mathrm{P_E}\begin{smallmatrix}\bullet\\\bullet\\\circ\\\bullet\end{smallmatrix}+\mathrm{P_E}\begin{smallmatrix}\bullet\\\circ\\\bullet\\\bullet\end{smallmatrix}+\mathrm{P_E}\begin{smallmatrix}\circ\\\bullet\\\bullet\\\bullet\end{smallmatrix}\big) + \tfrac{1}{6}\big(\mathrm{P_E}\begin{smallmatrix}\bullet\\\bullet\\\circ\\\circ\end{smallmatrix}+\mathrm{P_E}\begin{smallmatrix}\bullet\\\circ\\\bullet\\\circ\end{smallmatrix}+\mathrm{P_E}\begin{smallmatrix}\bullet\\\circ\\\circ\\\bullet\end{smallmatrix}+\mathrm{P_E}\begin{smallmatrix}\circ\\\bullet\\\bullet\\\circ\end{smallmatrix}+\mathrm{P_E}\begin{smallmatrix}\circ\\\bullet\\\circ\\\bullet\end{smallmatrix}+\mathrm{P_E}\begin{smallmatrix}\circ\\\circ\\\bullet\\\bullet\end{smallmatrix}\big)\Big)\mathrm{D_E}\begin{smallmatrix}\bullet\\\circ\\\bullet\end{smallmatrix}\Big) - \underset{\mathrm{ASE}}{\nu_f}\Big(\big(\mathrm{W_E}\bullet + \mathrm{W_E}\circ\big)\mathrm{T_E}\begin{smallmatrix}\bullet&\bullet\\\circ&\bullet\\\bullet&\circ\end{smallmatrix}\Big)$$

$$\frac{d\mathrm{T_E}\begin{smallmatrix}\bullet&\bullet\\\circ&\bullet\\\bullet&\bullet\end{smallmatrix}}{dt} = \underset{\mathrm{ATPoE}}{\nu_f}\big(\mathrm{T_O}\begin{smallmatrix}\bullet&\bullet\\\circ&\bullet\\\bullet&\bullet\end{smallmatrix}\big) + \underset{\mathrm{ASE}}{\nu_r}\Big(\Big(\mathrm{P_E}\begin{smallmatrix}\bullet\\\bullet\\\bullet\\\bullet\end{smallmatrix} + \tfrac{1}{4}\big(\mathrm{P_E}\begin{smallmatrix}\bullet\\\bullet\\\bullet\\\circ\end{smallmatrix}+\mathrm{P_E}\begin{smallmatrix}\bullet\\\bullet\\\circ\\\bullet\end{smallmatrix}+\mathrm{P_E}\begin{smallmatrix}\bullet\\\circ\\\bullet\\\bullet\end{smallmatrix}+\mathrm{P_E}\begin{smallmatrix}\circ\\\bullet\\\bullet\\\bullet\end{smallmatrix}\big)\Big)\mathrm{D_E}\begin{smallmatrix}\bullet\\\circ\\\bullet\end{smallmatrix}\Big) - \underset{\mathrm{ASE}}{\nu_f}\Big(\big(\mathrm{W_E}\bullet + \mathrm{W_E}\circ\big)\mathrm{T_E}\begin{smallmatrix}\bullet&\bullet\\\circ&\bullet\\\bullet&\bullet\end{smallmatrix}\Big)$$

$$\frac{d\mathrm{T_E}\begin{smallmatrix}\bullet&\circ\\\bullet&\circ\\\circ&\circ\end{smallmatrix}}{dt} = \underset{\mathrm{ATPoE}}{\nu_f}\big(\mathrm{T_O}\begin{smallmatrix}\bullet&\circ\\\bullet&\circ\\\circ&\circ\end{smallmatrix}\big) + \underset{\mathrm{ASE}}{\nu_r}\Big(\Big(\mathrm{P_E}\begin{smallmatrix}\circ\\\circ\\\circ\\\circ\end{smallmatrix} + \tfrac{1}{4}\big(\mathrm{P_E}\begin{smallmatrix}\bullet\\\circ\\\circ\\\circ\end{smallmatrix}+\mathrm{P_E}\begin{smallmatrix}\circ\\\bullet\\\circ\\\circ\end{smallmatrix}+\mathrm{P_E}\begin{smallmatrix}\circ\\\circ\\\bullet\\\circ\end{smallmatrix}+\mathrm{P_E}\begin{smallmatrix}\circ\\\circ\\\circ\\\bullet\end{smallmatrix}\big)\Big)\mathrm{D_E}\begin{smallmatrix}\bullet\\\bullet\\\circ\end{smallmatrix}\Big) - \underset{\mathrm{ASE}}{\nu_f}\Big(\big(\mathrm{W_E}\bullet + \mathrm{W_E}\circ\big)\mathrm{T_E}\begin{smallmatrix}\bullet&\circ\\\bullet&\circ\\\circ&\circ\end{smallmatrix}\Big)$$

$$\frac{d\mathrm{T_E}\begin{smallmatrix}\bullet&\circ\\\bullet&\circ\\\circ&\bullet\end{smallmatrix}}{dt} = \underset{\mathrm{ATPoE}}{\nu_f}\big(\mathrm{T_O}\begin{smallmatrix}\bullet&\circ\\\bullet&\circ\\\circ&\bullet\end{smallmatrix}\big) + \underset{\mathrm{ASE}}{\nu_r}\Big(\Big(\tfrac{1}{4}\big(\mathrm{P_E}\begin{smallmatrix}\bullet\\\circ\\\circ\\\circ\end{smallmatrix}+\mathrm{P_E}\begin{smallmatrix}\circ\\\bullet\\\circ\\\circ\end{smallmatrix}+\mathrm{P_E}\begin{smallmatrix}\circ\\\circ\\\bullet\\\circ\end{smallmatrix}+\mathrm{P_E}\begin{smallmatrix}\circ\\\circ\\\circ\\\bullet\end{smallmatrix}\big) + \tfrac{1}{6}\big(\mathrm{P_E}\begin{smallmatrix}\bullet\\\bullet\\\circ\\\circ\end{smallmatrix}+\mathrm{P_E}\begin{smallmatrix}\bullet\\\circ\\\bullet\\\circ\end{smallmatrix}+\mathrm{P_E}\begin{smallmatrix}\bullet\\\circ\\\circ\\\bullet\end{smallmatrix}+\mathrm{P_E}\begin{smallmatrix}\circ\\\bullet\\\bullet\\\circ\end{smallmatrix}+\mathrm{P_E}\begin{smallmatrix}\circ\\\bullet\\\circ\\\bullet\end{smallmatrix}+\mathrm{P_E}\begin{smallmatrix}\circ\\\circ\\\bullet\\\bullet\end{smallmatrix}\big)\Big)\mathrm{D_E}\begin{smallmatrix}\bullet\\\bullet\\\circ\end{smallmatrix}\Big) - \underset{\mathrm{ASE}}{\nu_f}\Big(\big(\mathrm{W_E}\bullet + \mathrm{W_E}\circ\big)\mathrm{T_E}\begin{smallmatrix}\bullet&\circ\\\bullet&\circ\\\circ&\bullet\end{smallmatrix}\Big)$$

$$\frac{d\mathrm{T_E}\begin{smallmatrix}\bullet&\circ\\\bullet&\bullet\\\circ&\circ\end{smallmatrix}}{dt} = \underset{\mathrm{ATPoE}}{\nu_f}\big(\mathrm{T_O}\begin{smallmatrix}\bullet&\circ\\\bullet&\bullet\\\circ&\circ\end{smallmatrix}\big) + \underset{\mathrm{ASE}}{\nu_r}\Big(\Big(\tfrac{1}{4}\big(\mathrm{P_E}\begin{smallmatrix}\bullet\\\circ\\\circ\\\circ\end{smallmatrix}+\mathrm{P_E}\begin{smallmatrix}\circ\\\bullet\\\circ\\\circ\end{smallmatrix}+\mathrm{P_E}\begin{smallmatrix}\circ\\\circ\\\bullet\\\circ\end{smallmatrix}+\mathrm{P_E}\begin{smallmatrix}\circ\\\circ\\\circ\\\bullet\end{smallmatrix}\big) + \tfrac{1}{6}\big(\mathrm{P_E}\begin{smallmatrix}\bullet\\\bullet\\\circ\\\circ\end{smallmatrix}+\mathrm{P_E}\begin{smallmatrix}\bullet\\\circ\\\bullet\\\circ\end{smallmatrix}+\mathrm{P_E}\begin{smallmatrix}\bullet\\\circ\\\circ\\\bullet\end{smallmatrix}+\mathrm{P_E}\begin{smallmatrix}\circ\\\bullet\\\bullet\\\circ\end{smallmatrix}+\mathrm{P_E}\begin{smallmatrix}\circ\\\bullet\\\circ\\\bullet\end{smallmatrix}+\mathrm{P_E}\begin{smallmatrix}\circ\\\circ\\\bullet\\\bullet\end{smallmatrix}\big)\Big)\mathrm{D_E}\begin{smallmatrix}\bullet\\\bullet\\\circ\end{smallmatrix}\Big) - \underset{\mathrm{ASE}}{\nu_f}\Big(\big(\mathrm{W_E}\bullet + \mathrm{W_E}\circ\big)\mathrm{T_E}\begin{smallmatrix}\bullet&\circ\\\bullet&\bullet\\\circ&\circ\end{smallmatrix}\Big)$$

$$\frac{d\mathrm{T_E}\begin{smallmatrix}\bullet&\circ\\\bullet&\bullet\\\circ&\bullet\end{smallmatrix}}{dt} = \underset{\mathrm{ATPoE}}{\nu_f}\big(\mathrm{T_O}\begin{smallmatrix}\bullet&\circ\\\bullet&\bullet\\\circ&\bullet\end{smallmatrix}\big) + \underset{\mathrm{ASE}}{\nu_r}\Big(\Big(\tfrac{1}{4}\big(\mathrm{P_E}\begin{smallmatrix}\bullet\\\bullet\\\bullet\\\circ\end{smallmatrix}+\mathrm{P_E}\begin{smallmatrix}\bullet\\\bullet\\\circ\\\bullet\end{smallmatrix}+\mathrm{P_E}\begin{smallmatrix}\bullet\\\circ\\\bullet\\\bullet\end{smallmatrix}+\mathrm{P_E}\begin{smallmatrix}\circ\\\bullet\\\bullet\\\bullet\end{smallmatrix}\big) + \tfrac{1}{6}\big(\mathrm{P_E}\begin{smallmatrix}\bullet\\\bullet\\\circ\\\circ\end{smallmatrix}+\mathrm{P_E}\begin{smallmatrix}\bullet\\\circ\\\bullet\\\circ\end{smallmatrix}+\mathrm{P_E}\begin{smallmatrix}\bullet\\\circ\\\circ\\\bullet\end{smallmatrix}+\mathrm{P_E}\begin{smallmatrix}\circ\\\bullet\\\bullet\\\circ\end{smallmatrix}+\mathrm{P_E}\begin{smallmatrix}\circ\\\bullet\\\circ\\\bullet\end{smallmatrix}+\mathrm{P_E}\begin{smallmatrix}\circ\\\circ\\\bullet\\\bullet\end{smallmatrix}\big)\Big)\mathrm{D_E}\begin{smallmatrix}\bullet\\\bullet\\\circ\end{smallmatrix}\Big) - \underset{\mathrm{ASE}}{\nu_f}\Big(\big(\mathrm{W_E}\bullet + \mathrm{W_E}\circ\big)\mathrm{T_E}\begin{smallmatrix}\bullet&\circ\\\bullet&\bullet\\\circ&\bullet\end{smallmatrix}\Big)$$

$$\frac{d\mathrm{T_E}\begin{smallmatrix}\bullet&\bullet\\\bullet&\circ\\\circ&\circ\end{smallmatrix}}{dt} = \underset{\mathrm{ATPoE}}{\nu_f}\big(\mathrm{T_O}\begin{smallmatrix}\bullet&\bullet\\\bullet&\circ\\\circ&\circ\end{smallmatrix}\big) + \underset{\mathrm{ASE}}{\nu_r}\Big(\Big(\tfrac{1}{4}\big(\mathrm{P_E}\begin{smallmatrix}\bullet\\\circ\\\circ\\\circ\end{smallmatrix}+\mathrm{P_E}\begin{smallmatrix}\circ\\\bullet\\\circ\\\circ\end{smallmatrix}+\mathrm{P_E}\begin{smallmatrix}\circ\\\circ\\\bullet\\\circ\end{smallmatrix}+\mathrm{P_E}\begin{smallmatrix}\circ\\\circ\\\circ\\\bullet\end{smallmatrix}\big) + \tfrac{1}{6}\big(\mathrm{P_E}\begin{smallmatrix}\bullet\\\bullet\\\circ\\\circ\end{smallmatrix}+\mathrm{P_E}\begin{smallmatrix}\bullet\\\circ\\\bullet\\\circ\end{smallmatrix}+\mathrm{P_E}\begin{smallmatrix}\bullet\\\circ\\\circ\\\bullet\end{smallmatrix}+\mathrm{P_E}\begin{smallmatrix}\circ\\\bullet\\\bullet\\\circ\end{smallmatrix}+\mathrm{P_E}\begin{smallmatrix}\circ\\\bullet\\\circ\\\bullet\end{smallmatrix}+\mathrm{P_E}\begin{smallmatrix}\circ\\\circ\\\bullet\\\bullet\end{smallmatrix}\big)\Big)\mathrm{D_E}\begin{smallmatrix}\bullet\\\bullet\\\circ\end{smallmatrix}\Big) - \underset{\mathrm{ASE}}{\nu_f}\Big(\big(\mathrm{W_E}\bullet + \mathrm{W_E}\circ\big)\mathrm{T_E}\begin{smallmatrix}\bullet&\bullet\\\bullet&\circ\\\circ&\circ\end{smallmatrix}\Big)$$

$$\frac{d\mathrm{T_E}\begin{smallmatrix}\bullet&\bullet\\\bullet&\circ\\\circ&\bullet\end{smallmatrix}}{dt} = \underset{\mathrm{ATPoE}}{\nu_f}\big(\mathrm{T_O}\begin{smallmatrix}\bullet&\bullet\\\bullet&\circ\\\circ&\bullet\end{smallmatrix}\big) + \underset{\mathrm{ASE}}{\nu_r}\Big(\Big(\tfrac{1}{4}\big(\mathrm{P_E}\begin{smallmatrix}\bullet\\\bullet\\\bullet\\\circ\end{smallmatrix}+\mathrm{P_E}\begin{smallmatrix}\bullet\\\bullet\\\circ\\\bullet\end{smallmatrix}+\mathrm{P_E}\begin{smallmatrix}\bullet\\\circ\\\bullet\\\bullet\end{smallmatrix}+\mathrm{P_E}\begin{smallmatrix}\circ\\\bullet\\\bullet\\\bullet\end{smallmatrix}\big) + \tfrac{1}{6}\big(\mathrm{P_E}\begin{smallmatrix}\bullet\\\bullet\\\circ\\\circ\end{smallmatrix}+\mathrm{P_E}\begin{smallmatrix}\bullet\\\circ\\\bullet\\\circ\end{smallmatrix}+\mathrm{P_E}\begin{smallmatrix}\bullet\\\circ\\\circ\\\bullet\end{smallmatrix}+\mathrm{P_E}\begin{smallmatrix}\circ\\\bullet\\\bullet\\\circ\end{smallmatrix}+\mathrm{P_E}\begin{smallmatrix}\circ\\\bullet\\\circ\\\bullet\end{smallmatrix}+\mathrm{P_E}\begin{smallmatrix}\circ\\\circ\\\bullet\\\bullet\end{smallmatrix}\big)\Big)\mathrm{D_E}\begin{smallmatrix}\bullet\\\bullet\\\circ\end{smallmatrix}\Big) - \underset{\mathrm{ASE}}{\nu_f}\Big(\big(\mathrm{W_E}\bullet + \mathrm{W_E}\circ\big)\mathrm{T_E}\begin{smallmatrix}\bullet&\bullet\\\bullet&\circ\\\circ&\bullet\end{smallmatrix}\Big)$$

$$\frac{d\mathrm{T_E}\begin{smallmatrix}\bullet&\bullet\\\bullet&\bullet\\\circ&\circ\end{smallmatrix}}{dt} = \underset{\mathrm{ATPoE}}{\nu_f}\big(\mathrm{T_O}\begin{smallmatrix}\bullet&\bullet\\\bullet&\bullet\\\circ&\circ\end{smallmatrix}\big) + \underset{\mathrm{ASE}}{\nu_r}\Big(\Big(\tfrac{1}{4}\big(\mathrm{P_E}\begin{smallmatrix}\bullet\\\bullet\\\bullet\\\circ\end{smallmatrix}+\mathrm{P_E}\begin{smallmatrix}\bullet\\\bullet\\\circ\\\bullet\end{smallmatrix}+\mathrm{P_E}\begin{smallmatrix}\bullet\\\circ\\\bullet\\\bullet\end{smallmatrix}+\mathrm{P_E}\begin{smallmatrix}\circ\\\bullet\\\bullet\\\bullet\end{smallmatrix}\big) + \tfrac{1}{6}\big(\mathrm{P_E}\begin{smallmatrix}\bullet\\\bullet\\\circ\\\circ\end{smallmatrix}+\mathrm{P_E}\begin{smallmatrix}\bullet\\\circ\\\bullet\\\circ\end{smallmatrix}+\mathrm{P_E}\begin{smallmatrix}\bullet\\\circ\\\circ\\\bullet\end{smallmatrix}+\mathrm{P_E}\begin{smallmatrix}\circ\\\bullet\\\bullet\\\circ\end{smallmatrix}+\mathrm{P_E}\begin{smallmatrix}\circ\\\bullet\\\circ\\\bullet\end{smallmatrix}+\mathrm{P_E}\begin{smallmatrix}\circ\\\circ\\\bullet\\\bullet\end{smallmatrix}\big)\Big)\mathrm{D_E}\begin{smallmatrix}\bullet\\\bullet\\\circ\end{smallmatrix}\Big) - \underset{\mathrm{ASE}}{\nu_f}\Big(\big(\mathrm{W_E}\bullet + \mathrm{W_E}\circ\big)\mathrm{T_E}\begin{smallmatrix}\bullet&\bullet\\\bullet&\bullet\\\circ&\circ\end{smallmatrix}\Big)$$

$$\frac{d\mathrm{T_E}\begin{smallmatrix}\bullet&\bullet\\\bullet&\bullet\\\circ&\bullet\end{smallmatrix}}{dt} = \underset{\mathrm{ATPoE}}{\nu_f}\big(\mathrm{T_O}\begin{smallmatrix}\bullet&\bullet\\\bullet&\bullet\\\circ&\bullet\end{smallmatrix}\big) + \underset{\mathrm{ASE}}{\nu_r}\Big(\Big(\mathrm{P_E}\begin{smallmatrix}\bullet\\\bullet\\\bullet\\\bullet\end{smallmatrix} + \tfrac{1}{4}\big(\mathrm{P_E}\begin{smallmatrix}\bullet\\\bullet\\\bullet\\\circ\end{smallmatrix}+\mathrm{P_E}\begin{smallmatrix}\bullet\\\bullet\\\circ\\\bullet\end{smallmatrix}+\mathrm{P_E}\begin{smallmatrix}\bullet\\\circ\\\bullet\\\bullet\end{smallmatrix}+\mathrm{P_E}\begin{smallmatrix}\circ\\\bullet\\\bullet\\\bullet\end{smallmatrix}\big)\Big)\mathrm{D_E}\begin{smallmatrix}\bullet\\\bullet\\\circ\end{smallmatrix}\Big) - \underset{\mathrm{ASE}}{\nu_f}\Big(\big(\mathrm{W_E}\bullet + \mathrm{W_E}\circ\big)\mathrm{T_E}\begin{smallmatrix}\bullet&\bullet\\\bullet&\bullet\\\circ&\bullet\end{smallmatrix}\Big)$$

$$\frac{d\mathrm{T_E}\begin{smallmatrix}\bullet&\circ\\\bullet&\circ\\\bullet&\circ\end{smallmatrix}}{dt} = \underset{\mathrm{ATPoE}}{\nu_f}\big(\mathrm{T_O}\begin{smallmatrix}\bullet&\circ\\\bullet&\circ\\\bullet&\circ\end{smallmatrix}\big) + \underset{\mathrm{ASE}}{\nu_r}\Big(\Big(\mathrm{P_E}\begin{smallmatrix}\circ\\\circ\\\circ\\\circ\end{smallmatrix} + \tfrac{1}{4}\big(\mathrm{P_E}\begin{smallmatrix}\bullet\\\circ\\\circ\\\circ\end{smallmatrix}+\mathrm{P_E}\begin{smallmatrix}\circ\\\bullet\\\circ\\\circ\end{smallmatrix}+\mathrm{P_E}\begin{smallmatrix}\circ\\\circ\\\bullet\\\circ\end{smallmatrix}+\mathrm{P_E}\begin{smallmatrix}\circ\\\circ\\\circ\\\bullet\end{smallmatrix}\big)\Big)\mathrm{D_E}\begin{smallmatrix}\bullet\\\bullet\\\bullet\end{smallmatrix}\Big) - \underset{\mathrm{ASE}}{\nu_f}\Big(\big(\mathrm{W_E}\bullet + \mathrm{W_E}\circ\big)\mathrm{T_E}\begin{smallmatrix}\bullet&\circ\\\bullet&\circ\\\bullet&\circ\end{smallmatrix}\Big)$$

$$\frac{d\mathrm{T_E}\begin{smallmatrix}\bullet&\circ\\\bullet&\circ\\\bullet&\bullet\end{smallmatrix}}{dt} = \underset{\mathrm{ATPoE}}{\nu_f}\big(\mathrm{T_O}\begin{smallmatrix}\bullet&\circ\\\bullet&\circ\\\bullet&\bullet\end{smallmatrix}\big) + \underset{\mathrm{ASE}}{\nu_r}\Big(\Big(\tfrac{1}{4}\big(\mathrm{P_E}\begin{smallmatrix}\bullet\\\circ\\\circ\\\circ\end{smallmatrix}+\mathrm{P_E}\begin{smallmatrix}\circ\\\bullet\\\circ\\\circ\end{smallmatrix}+\mathrm{P_E}\begin{smallmatrix}\circ\\\circ\\\bullet\\\circ\end{smallmatrix}+\mathrm{P_E}\begin{smallmatrix}\circ\\\circ\\\circ\\\bullet\end{smallmatrix}\big) + \tfrac{1}{6}\big(\mathrm{P_E}\begin{smallmatrix}\bullet\\\bullet\\\circ\\\circ\end{smallmatrix}+\mathrm{P_E}\begin{smallmatrix}\bullet\\\circ\\\bullet\\\circ\end{smallmatrix}+\mathrm{P_E}\begin{smallmatrix}\bullet\\\circ\\\circ\\\bullet\end{smallmatrix}+\mathrm{P_E}\begin{smallmatrix}\circ\\\bullet\\\bullet\\\circ\end{smallmatrix}+\mathrm{P_E}\begin{smallmatrix}\circ\\\bullet\\\circ\\\bullet\end{smallmatrix}+\mathrm{P_E}\begin{smallmatrix}\circ\\\circ\\\bullet\\\bullet\end{smallmatrix}\big)\Big)\mathrm{D_E}\begin{smallmatrix}\bullet\\\bullet\\\bullet\end{smallmatrix}\Big) - \underset{\mathrm{ASE}}{\nu_f}\Big(\big(\mathrm{W_E}\bullet + \mathrm{W_E}\circ\big)\mathrm{T_E}\begin{smallmatrix}\bullet&\circ\\\bullet&\circ\\\bullet&\bullet\end{smallmatrix}\Big)$$

$$\frac{d\mathrm{T_E}\begin{smallmatrix}\bullet&\circ\\\bullet&\bullet\\\bullet&\circ\end{smallmatrix}}{dt} = \underset{\mathrm{ATPoE}}{\nu_f}\big(\mathrm{T_O}\begin{smallmatrix}\bullet&\circ\\\bullet&\bullet\\\bullet&\circ\end{smallmatrix}\big) + \underset{\mathrm{ASE}}{\nu_r}\Big(\Big(\tfrac{1}{4}\big(\mathrm{P_E}\begin{smallmatrix}\bullet\\\circ\\\circ\\\circ\end{smallmatrix}+\mathrm{P_E}\begin{smallmatrix}\circ\\\bullet\\\circ\\\circ\end{smallmatrix}+\mathrm{P_E}\begin{smallmatrix}\circ\\\circ\\\bullet\\\circ\end{smallmatrix}+\mathrm{P_E}\begin{smallmatrix}\circ\\\circ\\\circ\\\bullet\end{smallmatrix}\big) + \tfrac{1}{6}\big(\mathrm{P_E}\begin{smallmatrix}\bullet\\\bullet\\\circ\\\circ\end{smallmatrix}+\mathrm{P_E}\begin{smallmatrix}\bullet\\\circ\\\bullet\\\circ\end{smallmatrix}+\mathrm{P_E}\begin{smallmatrix}\bullet\\\circ\\\circ\\\bullet\end{smallmatrix}+\mathrm{P_E}\begin{smallmatrix}\circ\\\bullet\\\bullet\\\circ\end{smallmatrix}+\mathrm{P_E}\begin{smallmatrix}\circ\\\bullet\\\circ\\\bullet\end{smallmatrix}+\mathrm{P_E}\begin{smallmatrix}\circ\\\circ\\\bullet\\\bullet\end{smallmatrix}\big)\Big)\mathrm{D_E}\begin{smallmatrix}\bullet\\\bullet\\\bullet\end{smallmatrix}\Big) - \underset{\mathrm{ASE}}{\nu_f}\Big(\big(\mathrm{W_E}\bullet + \mathrm{W_E}\circ\big)\mathrm{T_E}\begin{smallmatrix}\bullet&\circ\\\bullet&\bullet\\\bullet&\circ\end{smallmatrix}\Big)$$

$$\frac{d\mathrm{T_E}\begin{smallmatrix}\bullet&\circ\\\bullet&\bullet\\\bullet&\bullet\end{smallmatrix}}{dt} = \underset{\mathrm{ATPoE}}{\nu_f}\big(\mathrm{T_O}\begin{smallmatrix}\bullet&\circ\\\bullet&\bullet\\\bullet&\bullet\end{smallmatrix}\big) + \underset{\mathrm{ASE}}{\nu_r}\Big(\Big(\tfrac{1}{4}\big(\mathrm{P_E}\begin{smallmatrix}\bullet\\\bullet\\\bullet\\\circ\end{smallmatrix}+\mathrm{P_E}\begin{smallmatrix}\bullet\\\bullet\\\circ\\\bullet\end{smallmatrix}+\mathrm{P_E}\begin{smallmatrix}\bullet\\\circ\\\bullet\\\bullet\end{smallmatrix}+\mathrm{P_E}\begin{smallmatrix}\circ\\\bullet\\\bullet\\\bullet\end{smallmatrix}\big) + \tfrac{1}{6}\big(\mathrm{P_E}\begin{smallmatrix}\bullet\\\bullet\\\circ\\\circ\end{smallmatrix}+\mathrm{P_E}\begin{smallmatrix}\bullet\\\circ\\\bullet\\\circ\end{smallmatrix}+\mathrm{P_E}\begin{smallmatrix}\bullet\\\circ\\\circ\\\bullet\end{smallmatrix}+\mathrm{P_E}\begin{smallmatrix}\circ\\\bullet\\\bullet\\\circ\end{smallmatrix}+\mathrm{P_E}\begin{smallmatrix}\circ\\\bullet\\\circ\\\bullet\end{smallmatrix}+\mathrm{P_E}\begin{smallmatrix}\circ\\\circ\\\bullet\\\bullet\end{smallmatrix}\big)\Big)\mathrm{D_E}\begin{smallmatrix}\bullet\\\bullet\\\bullet\end{smallmatrix}\Big) - \underset{\mathrm{ASE}}{\nu_f}\Big(\big(\mathrm{W_E}\bullet + \mathrm{W_E}\circ\big)\mathrm{T_E}\begin{smallmatrix}\bullet&\circ\\\bullet&\bullet\\\bullet&\bullet\end{smallmatrix}\Big)$$

$$\frac{d\mathrm{T_E}\begin{smallmatrix}\bullet&\bullet\\\bullet&\circ\\\bullet&\circ\end{smallmatrix}}{dt} = \underset{\mathrm{ATPoE}}{\nu_f}\big(\mathrm{T_O}\begin{smallmatrix}\bullet&\bullet\\\bullet&\circ\\\bullet&\circ\end{smallmatrix}\big) + \underset{\mathrm{ASE}}{\nu_r}\Big(\Big(\tfrac{1}{4}\big(\mathrm{P_E}\begin{smallmatrix}\bullet\\\circ\\\circ\\\circ\end{smallmatrix}+\mathrm{P_E}\begin{smallmatrix}\circ\\\bullet\\\circ\\\circ\end{smallmatrix}+\mathrm{P_E}\begin{smallmatrix}\circ\\\circ\\\bullet\\\circ\end{smallmatrix}+\mathrm{P_E}\begin{smallmatrix}\circ\\\circ\\\circ\\\bullet\end{smallmatrix}\big) + \tfrac{1}{6}\big(\mathrm{P_E}\begin{smallmatrix}\bullet\\\bullet\\\circ\\\circ\end{smallmatrix}+\mathrm{P_E}\begin{smallmatrix}\bullet\\\circ\\\bullet\\\circ\end{smallmatrix}+\mathrm{P_E}\begin{smallmatrix}\bullet\\\circ\\\circ\\\bullet\end{smallmatrix}+\mathrm{P_E}\begin{smallmatrix}\circ\\\bullet\\\bullet\\\circ\end{smallmatrix}+\mathrm{P_E}\begin{smallmatrix}\circ\\\bullet\\\circ\\\bullet\end{smallmatrix}+\mathrm{P_E}\begin{smallmatrix}\circ\\\circ\\\bullet\\\bullet\end{smallmatrix}\big)\Big)\mathrm{D_E}\begin{smallmatrix}\bullet\\\bullet\\\bullet\end{smallmatrix}\Big) - \underset{\mathrm{ASE}}{\nu_f}\Big(\big(\mathrm{W_E}\bullet + \mathrm{W_E}\circ\big)\mathrm{T_E}\begin{smallmatrix}\bullet&\bullet\\\bullet&\circ\\\bullet&\circ\end{smallmatrix}\Big)$$

$$\frac{d\mathrm{T_E}\begin{smallmatrix}\bullet&\bullet\\\bullet&\circ\\\bullet&\bullet\end{smallmatrix}}{dt} = \underset{\mathrm{ATPoE}}{\nu_f}\big(\mathrm{T_O}\begin{smallmatrix}\bullet&\bullet\\\bullet&\circ\\\bullet&\bullet\end{smallmatrix}\big) + \underset{\mathrm{ASE}}{\nu_r}\Big(\Big(\tfrac{1}{4}\big(\mathrm{P_E}\begin{smallmatrix}\bullet\\\bullet\\\bullet\\\circ\end{smallmatrix}+\mathrm{P_E}\begin{smallmatrix}\bullet\\\bullet\\\circ\\\bullet\end{smallmatrix}+\mathrm{P_E}\begin{smallmatrix}\bullet\\\circ\\\bullet\\\bullet\end{smallmatrix}+\mathrm{P_E}\begin{smallmatrix}\circ\\\bullet\\\bullet\\\bullet\end{smallmatrix}\big) + \tfrac{1}{6}\big(\mathrm{P_E}\begin{smallmatrix}\bullet\\\bullet\\\circ\\\circ\end{smallmatrix}+\mathrm{P_E}\begin{smallmatrix}\bullet\\\circ\\\bullet\\\circ\end{smallmatrix}+\mathrm{P_E}\begin{smallmatrix}\bullet\\\circ\\\circ\\\bullet\end{smallmatrix}+\mathrm{P_E}\begin{smallmatrix}\circ\\\bullet\\\bullet\\\circ\end{smallmatrix}+\mathrm{P_E}\begin{smallmatrix}\circ\\\bullet\\\circ\\\bullet\end{smallmatrix}+\mathrm{P_E}\begin{smallmatrix}\circ\\\circ\\\bullet\\\bullet\end{smallmatrix}\big)\Big)\mathrm{D_E}\begin{smallmatrix}\bullet\\\bullet\\\bullet\end{smallmatrix}\Big) - \underset{\mathrm{ASE}}{\nu_f}\Big(\big(\mathrm{W_E}\bullet + \mathrm{W_E}\circ\big)\mathrm{T_E}\begin{smallmatrix}\bullet&\bullet\\\bullet&\circ\\\bullet&\bullet\end{smallmatrix}\Big)$$

$$\frac{d\mathrm{T_E}\begin{smallmatrix}\bullet&\bullet\\\bullet&\bullet\\\bullet&\circ\end{smallmatrix}}{dt} = \underset{\mathrm{ATPoE}}{\nu_f}\big(\mathrm{T_O}\begin{smallmatrix}\bullet&\bullet\\\bullet&\bullet\\\bullet&\circ\end{smallmatrix}\big) + \underset{\mathrm{ASE}}{\nu_r}\Big(\Big(\tfrac{1}{4}\big(\mathrm{P_E}\begin{smallmatrix}\bullet\\\bullet\\\bullet\\\circ\end{smallmatrix}+\mathrm{P_E}\begin{smallmatrix}\bullet\\\bullet\\\circ\\\bullet\end{smallmatrix}+\mathrm{P_E}\begin{smallmatrix}\bullet\\\circ\\\bullet\\\bullet\end{smallmatrix}+\mathrm{P_E}\begin{smallmatrix}\circ\\\bullet\\\bullet\\\bullet\end{smallmatrix}\big) + \tfrac{1}{6}\big(\mathrm{P_E}\begin{smallmatrix}\bullet\\\bullet\\\circ\\\circ\end{smallmatrix}+\mathrm{P_E}\begin{smallmatrix}\bullet\\\circ\\\bullet\\\circ\end{smallmatrix}+\mathrm{P_E}\begin{smallmatrix}\bullet\\\circ\\\circ\\\bullet\end{smallmatrix}+\mathrm{P_E}\begin{smallmatrix}\circ\\\bullet\\\bullet\\\circ\end{smallmatrix}+\mathrm{P_E}\begin{smallmatrix}\circ\\\bullet\\\circ\\\bullet\end{smallmatrix}+\mathrm{P_E}\begin{smallmatrix}\circ\\\circ\\\bullet\\\bullet\end{smallmatrix}\big)\Big)\mathrm{D_E}\begin{smallmatrix}\bullet\\\bullet\\\bullet\end{smallmatrix}\Big) - \underset{\mathrm{ASE}}{\nu_f}\Big(\big(\mathrm{W_E}\bullet + \mathrm{W_E}\circ\big)\mathrm{T_E}\begin{smallmatrix}\bullet&\bullet\\\bullet&\bullet\\\bullet&\circ\end{smallmatrix}\Big)$$

$$\frac{d\mathrm{T_E}\begin{smallmatrix}\bullet&\bullet\\\bullet&\bullet\\\bullet&\bullet\end{smallmatrix}}{dt} = \underset{\mathrm{ATPoE}}{\nu_f}\big(\mathrm{T_O}\begin{smallmatrix}\bullet&\bullet\\\bullet&\bullet\\\bullet&\bullet\end{smallmatrix}\big) + \underset{\mathrm{ASE}}{\nu_r}\Big(\Big(\mathrm{P_E}\begin{smallmatrix}\bullet\\\bullet\\\bullet\\\bullet\end{smallmatrix} + \tfrac{1}{4}\big(\mathrm{P_E}\begin{smallmatrix}\bullet\\\bullet\\\bullet\\\circ\end{smallmatrix}+\mathrm{P_E}\begin{smallmatrix}\bullet\\\bullet\\\circ\\\bullet\end{smallmatrix}+\mathrm{P_E}\begin{smallmatrix}\bullet\\\circ\\\bullet\\\bullet\end{smallmatrix}+\mathrm{P_E}\begin{smallmatrix}\circ\\\bullet\\\bullet\\\bullet\end{smallmatrix}\big)\Big)\mathrm{D_E}\begin{smallmatrix}\bullet\\\bullet\\\bullet\end{smallmatrix}\Big) - \underset{\mathrm{ASE}}{\nu_f}\Big(\big(\mathrm{W_E}\bullet + \mathrm{W_E}\circ\big)\mathrm{T_E}\begin{smallmatrix}\bullet&\bullet\\\bullet&\bullet\\\bullet&\bullet\end{smallmatrix}\Big)$$

$$\frac{dT_{\mathrm{I}}^{\substack{\circ\circ\\\circ\circ\\\circ\circ}}}{dt} = \nu^{f}_{\mathrm{ATPMI}}(T_{\mathrm{M}}^{\substack{\circ\circ\\\circ\circ\\\circ\circ}}) + \nu^{r}_{\mathrm{AKI}}(D_{\mathrm{I}}^{\substack{\circ\\\circ\\\circ}}D_{\mathrm{I}}^{\substack{\circ\\\circ\\\circ}}) + \nu^{r}_{\mathrm{ATPIO}}(T_{\mathrm{O}}^{\substack{\circ\circ\\\circ\circ\\\circ\circ}}) + \nu^{r}_{\mathrm{CKI}}(D_{\mathrm{I}}^{\substack{\circ\\\circ\\\circ}}C_{\mathrm{I}}^{\substack{\circ\\\circ\\\circ}}) - \nu^{f}_{\mathrm{AKI}}(T_{\mathrm{I}}^{\substack{\circ\circ\\\circ\circ\\\circ\circ}}) - \nu^{f}_{\mathrm{ATPIO}}(T_{\mathrm{I}}^{\substack{\circ\circ\\\circ\circ\\\circ\circ}}) - \nu^{f}_{\mathrm{CKI}}(T_{\mathrm{I}}^{\substack{\circ\circ\\\circ\circ\\\circ\circ}}) - \nu^{r}_{\mathrm{ATPMI}}(T_{\mathrm{I}}^{\substack{\circ\circ\\\circ\circ\\\circ\circ}})$$

$$\frac{dT_{\mathrm{I}}^{\substack{\circ\circ\\\circ\circ\\\circ\bullet}}}{dt} = \nu^{f}_{\mathrm{ATPMI}}(T_{\mathrm{M}}^{\substack{\circ\circ\\\circ\circ\\\circ\bullet}}) + \nu^{r}_{\mathrm{AKI}}(D_{\mathrm{I}}^{\substack{\circ\\\circ\\\bullet}}D_{\mathrm{I}}^{\substack{\circ\\\circ\\\circ}}) + \nu^{r}_{\mathrm{ATPIO}}(T_{\mathrm{O}}^{\substack{\circ\circ\\\circ\circ\\\circ\bullet}}) + \nu^{r}_{\mathrm{CKI}}(D_{\mathrm{I}}^{\substack{\circ\\\circ\\\circ}}C_{\mathrm{I}}^{\substack{\circ\\\circ\\\bullet}}) - \nu^{f}_{\mathrm{AKI}}(T_{\mathrm{I}}^{\substack{\circ\circ\\\circ\circ\\\circ\bullet}}) - \nu^{f}_{\mathrm{ATPIO}}(T_{\mathrm{I}}^{\substack{\circ\circ\\\circ\circ\\\circ\bullet}}) - \nu^{f}_{\mathrm{CKI}}(T_{\mathrm{I}}^{\substack{\circ\circ\\\circ\circ\\\circ\bullet}}) - \nu^{r}_{\mathrm{ATPMI}}(T_{\mathrm{I}}^{\substack{\circ\circ\\\circ\circ\\\circ\bullet}})$$

$$\frac{dT_{\mathrm{I}}^{\substack{\circ\circ\\\circ\bullet\\\circ\circ}}}{dt} = \nu^{f}_{\mathrm{ATPMI}}(T_{\mathrm{M}}^{\substack{\circ\circ\\\circ\bullet\\\circ\circ}}) + \nu^{r}_{\mathrm{AKI}}(D_{\mathrm{I}}^{\substack{\circ\\\bullet\\\circ}}D_{\mathrm{I}}^{\substack{\circ\\\circ\\\circ}}) + \nu^{r}_{\mathrm{ATPIO}}(T_{\mathrm{O}}^{\substack{\circ\circ\\\circ\bullet\\\circ\circ}}) + \nu^{r}_{\mathrm{CKI}}(D_{\mathrm{I}}^{\substack{\circ\\\circ\\\circ}}C_{\mathrm{I}}^{\substack{\circ\\\bullet\\\circ}}) - \nu^{f}_{\mathrm{AKI}}(T_{\mathrm{I}}^{\substack{\circ\circ\\\circ\bullet\\\circ\circ}}) - \nu^{f}_{\mathrm{ATPIO}}(T_{\mathrm{I}}^{\substack{\circ\circ\\\circ\bullet\\\circ\circ}}) - \nu^{f}_{\mathrm{CKI}}(T_{\mathrm{I}}^{\substack{\circ\circ\\\circ\bullet\\\circ\circ}}) - \nu^{r}_{\mathrm{ATPMI}}(T_{\mathrm{I}}^{\substack{\circ\circ\\\circ\bullet\\\circ\circ}})$$

$$\frac{dT_{\mathrm{I}}^{\substack{\circ\circ\\\circ\bullet\\\circ\bullet}}}{dt} = \nu^{f}_{\mathrm{ATPMI}}(T_{\mathrm{M}}^{\substack{\circ\circ\\\circ\bullet\\\circ\bullet}}) + \nu^{r}_{\mathrm{AKI}}(D_{\mathrm{I}}^{\substack{\circ\\\bullet\\\bullet}}D_{\mathrm{I}}^{\substack{\circ\\\circ\\\circ}}) + \nu^{r}_{\mathrm{ATPIO}}(T_{\mathrm{O}}^{\substack{\circ\circ\\\circ\bullet\\\circ\bullet}}) + \nu^{r}_{\mathrm{CKI}}(D_{\mathrm{I}}^{\substack{\circ\\\circ\\\circ}}C_{\mathrm{I}}^{\substack{\circ\\\bullet\\\bullet}}) - \nu^{f}_{\mathrm{AKI}}(T_{\mathrm{I}}^{\substack{\circ\circ\\\circ\bullet\\\circ\bullet}}) - \nu^{f}_{\mathrm{ATPIO}}(T_{\mathrm{I}}^{\substack{\circ\circ\\\circ\bullet\\\circ\bullet}}) - \nu^{f}_{\mathrm{CKI}}(T_{\mathrm{I}}^{\substack{\circ\circ\\\circ\bullet\\\circ\bullet}}) - \nu^{r}_{\mathrm{ATPMI}}(T_{\mathrm{I}}^{\substack{\circ\circ\\\circ\bullet\\\circ\bullet}})$$

$$\frac{dT_{\mathrm{I}}^{\substack{\circ\bullet\\\circ\circ\\\circ\circ}}}{dt} = \nu^{f}_{\mathrm{ATPMI}}(T_{\mathrm{M}}^{\substack{\circ\bullet\\\circ\circ\\\circ\circ}}) + \nu^{r}_{\mathrm{AKI}}(D_{\mathrm{I}}^{\substack{\bullet\\\circ\\\circ}}D_{\mathrm{I}}^{\substack{\circ\\\circ\\\circ}}) + \nu^{r}_{\mathrm{ATPIO}}(T_{\mathrm{O}}^{\substack{\circ\bullet\\\circ\circ\\\circ\circ}}) + \nu^{r}_{\mathrm{CKI}}(D_{\mathrm{I}}^{\substack{\circ\\\circ\\\circ}}C_{\mathrm{I}}^{\substack{\bullet\\\circ\\\circ}}) - \nu^{f}_{\mathrm{AKI}}(T_{\mathrm{I}}^{\substack{\circ\bullet\\\circ\circ\\\circ\circ}}) - \nu^{f}_{\mathrm{ATPIO}}(T_{\mathrm{I}}^{\substack{\circ\bullet\\\circ\circ\\\circ\circ}}) - \nu^{f}_{\mathrm{CKI}}(T_{\mathrm{I}}^{\substack{\circ\bullet\\\circ\circ\\\circ\circ}}) - \nu^{r}_{\mathrm{ATPMI}}(T_{\mathrm{I}}^{\substack{\circ\bullet\\\circ\circ\\\circ\circ}})$$

$$\frac{dT_{\mathrm{I}}^{\substack{\circ\bullet\\\circ\circ\\\circ\bullet}}}{dt} = \nu^{f}_{\mathrm{ATPMI}}(T_{\mathrm{M}}^{\substack{\circ\bullet\\\circ\circ\\\circ\bullet}}) + \nu^{r}_{\mathrm{AKI}}(D_{\mathrm{I}}^{\substack{\bullet\\\circ\\\bullet}}D_{\mathrm{I}}^{\substack{\circ\\\circ\\\circ}}) + \nu^{r}_{\mathrm{ATPIO}}(T_{\mathrm{O}}^{\substack{\circ\bullet\\\circ\circ\\\circ\bullet}}) + \nu^{r}_{\mathrm{CKI}}(D_{\mathrm{I}}^{\substack{\circ\\\circ\\\circ}}C_{\mathrm{I}}^{\substack{\bullet\\\circ\\\bullet}}) - \nu^{f}_{\mathrm{AKI}}(T_{\mathrm{I}}^{\substack{\circ\bullet\\\circ\circ\\\circ\bullet}}) - \nu^{f}_{\mathrm{ATPIO}}(T_{\mathrm{I}}^{\substack{\circ\bullet\\\circ\circ\\\circ\bullet}}) - \nu^{f}_{\mathrm{CKI}}(T_{\mathrm{I}}^{\substack{\circ\bullet\\\circ\circ\\\circ\bullet}}) - \nu^{r}_{\mathrm{ATPMI}}(T_{\mathrm{I}}^{\substack{\circ\bullet\\\circ\circ\\\circ\bullet}})$$

$$\frac{dT_{\mathrm{I}}^{\substack{\circ\bullet\\\circ\bullet\\\circ\circ}}}{dt} = \nu^{f}_{\mathrm{ATPMI}}(T_{\mathrm{M}}^{\substack{\circ\bullet\\\circ\bullet\\\circ\circ}}) + \nu^{r}_{\mathrm{AKI}}(D_{\mathrm{I}}^{\substack{\bullet\\\bullet\\\circ}}D_{\mathrm{I}}^{\substack{\circ\\\circ\\\circ}}) + \nu^{r}_{\mathrm{ATPIO}}(T_{\mathrm{O}}^{\substack{\circ\bullet\\\circ\bullet\\\circ\circ}}) + \nu^{r}_{\mathrm{CKI}}(D_{\mathrm{I}}^{\substack{\circ\\\circ\\\circ}}C_{\mathrm{I}}^{\substack{\bullet\\\bullet\\\circ}}) - \nu^{f}_{\mathrm{AKI}}(T_{\mathrm{I}}^{\substack{\circ\bullet\\\circ\bullet\\\circ\circ}}) - \nu^{f}_{\mathrm{ATPIO}}(T_{\mathrm{I}}^{\substack{\circ\bullet\\\circ\bullet\\\circ\circ}}) - \nu^{f}_{\mathrm{CKI}}(T_{\mathrm{I}}^{\substack{\circ\bullet\\\circ\bullet\\\circ\circ}}) - \nu^{r}_{\mathrm{ATPMI}}(T_{\mathrm{I}}^{\substack{\circ\bullet\\\circ\bullet\\\circ\circ}})$$

$$\frac{dT_{\mathrm{I}}^{\substack{\circ\bullet\\\circ\bullet\\\circ\bullet}}}{dt} = \nu^{f}_{\mathrm{ATPMI}}(T_{\mathrm{M}}^{\substack{\circ\bullet\\\circ\bullet\\\circ\bullet}}) + \nu^{r}_{\mathrm{AKI}}(D_{\mathrm{I}}^{\substack{\bullet\\\bullet\\\bullet}}D_{\mathrm{I}}^{\substack{\circ\\\circ\\\circ}}) + \nu^{r}_{\mathrm{ATPIO}}(T_{\mathrm{O}}^{\substack{\circ\bullet\\\circ\bullet\\\circ\bullet}}) + \nu^{r}_{\mathrm{CKI}}(D_{\mathrm{I}}^{\substack{\circ\\\circ\\\circ}}C_{\mathrm{I}}^{\substack{\bullet\\\bullet\\\bullet}}) - \nu^{f}_{\mathrm{AKI}}(T_{\mathrm{I}}^{\substack{\circ\bullet\\\circ\bullet\\\circ\bullet}}) - \nu^{f}_{\mathrm{ATPIO}}(T_{\mathrm{I}}^{\substack{\circ\bullet\\\circ\bullet\\\circ\bullet}}) - \nu^{f}_{\mathrm{CKI}}(T_{\mathrm{I}}^{\substack{\circ\bullet\\\circ\bullet\\\circ\bullet}}) - \nu^{r}_{\mathrm{ATPMI}}(T_{\mathrm{I}}^{\substack{\circ\bullet\\\circ\bullet\\\circ\bullet}})$$

$$\frac{dT_{\mathrm{I}}^{\substack{\circ\circ\\\circ\circ\\\bullet\circ}}}{dt} = \nu^{f}_{\mathrm{ATPMI}}(T_{\mathrm{M}}^{\substack{\circ\circ\\\circ\circ\\\bullet\circ}}) + \nu^{r}_{\mathrm{AKI}}(D_{\mathrm{I}}^{\substack{\circ\\\circ\\\bullet}}D_{\mathrm{I}}^{\substack{\circ\\\circ\\\circ}}) + \nu^{r}_{\mathrm{ATPIO}}(T_{\mathrm{O}}^{\substack{\circ\circ\\\circ\circ\\\bullet\circ}}) + \nu^{r}_{\mathrm{CKI}}(D_{\mathrm{I}}^{\substack{\circ\\\circ\\\bullet}}C_{\mathrm{I}}^{\substack{\circ\\\circ\\\circ}}) - \nu^{f}_{\mathrm{AKI}}(T_{\mathrm{I}}^{\substack{\circ\circ\\\circ\circ\\\bullet\circ}}) - \nu^{f}_{\mathrm{ATPIO}}(T_{\mathrm{I}}^{\substack{\circ\circ\\\circ\circ\\\bullet\circ}}) - \nu^{f}_{\mathrm{CKI}}(T_{\mathrm{I}}^{\substack{\circ\circ\\\circ\circ\\\bullet\circ}}) - \nu^{r}_{\mathrm{ATPMI}}(T_{\mathrm{I}}^{\substack{\circ\circ\\\circ\circ\\\bullet\circ}})$$

$$\frac{dT_{\mathrm{I}}^{\substack{\circ\circ\\\circ\circ\\\bullet\bullet}}}{dt} = \nu^{f}_{\mathrm{ATPMI}}(T_{\mathrm{M}}^{\substack{\circ\circ\\\circ\circ\\\bullet\bullet}}) + \nu^{r}_{\mathrm{AKI}}(D_{\mathrm{I}}^{\substack{\circ\\\circ\\\bullet}}D_{\mathrm{I}}^{\substack{\circ\\\circ\\\bullet}}) + \nu^{r}_{\mathrm{ATPIO}}(T_{\mathrm{O}}^{\substack{\circ\circ\\\circ\circ\\\bullet\bullet}}) + \nu^{r}_{\mathrm{CKI}}(D_{\mathrm{I}}^{\substack{\circ\\\circ\\\bullet}}C_{\mathrm{I}}^{\substack{\circ\\\circ\\\bullet}}) - \nu^{f}_{\mathrm{AKI}}(T_{\mathrm{I}}^{\substack{\circ\circ\\\circ\circ\\\bullet\bullet}}) - \nu^{f}_{\mathrm{ATPIO}}(T_{\mathrm{I}}^{\substack{\circ\circ\\\circ\circ\\\bullet\bullet}}) - \nu^{f}_{\mathrm{CKI}}(T_{\mathrm{I}}^{\substack{\circ\circ\\\circ\circ\\\bullet\bullet}}) - \nu^{r}_{\mathrm{ATPMI}}(T_{\mathrm{I}}^{\substack{\circ\circ\\\circ\circ\\\bullet\bullet}})$$

$$\frac{dT_{\mathrm{I}}^{\substack{\circ\circ\\\circ\bullet\\\bullet\circ}}}{dt} = \nu^{f}_{\mathrm{ATPMI}}(T_{\mathrm{M}}^{\substack{\circ\circ\\\circ\bullet\\\bullet\circ}}) + \nu^{r}_{\mathrm{AKI}}(D_{\mathrm{I}}^{\substack{\circ\\\bullet\\\circ}}D_{\mathrm{I}}^{\substack{\circ\\\circ\\\bullet}}) + \nu^{r}_{\mathrm{ATPIO}}(T_{\mathrm{O}}^{\substack{\circ\circ\\\circ\bullet\\\bullet\circ}}) + \nu^{r}_{\mathrm{CKI}}(D_{\mathrm{I}}^{\substack{\circ\\\circ\\\bullet}}C_{\mathrm{I}}^{\substack{\circ\\\bullet\\\circ}}) - \nu^{f}_{\mathrm{AKI}}(T_{\mathrm{I}}^{\substack{\circ\circ\\\circ\bullet\\\bullet\circ}}) - \nu^{f}_{\mathrm{ATPIO}}(T_{\mathrm{I}}^{\substack{\circ\circ\\\circ\bullet\\\bullet\circ}}) - \nu^{f}_{\mathrm{CKI}}(T_{\mathrm{I}}^{\substack{\circ\circ\\\circ\bullet\\\bullet\circ}}) - \nu^{r}_{\mathrm{ATPMI}}(T_{\mathrm{I}}^{\substack{\circ\circ\\\circ\bullet\\\bullet\circ}})$$

$$\frac{dT_{\mathrm{I}}^{\substack{\circ\circ\\\circ\bullet\\\bullet\bullet}}}{dt} = \nu^{f}_{\mathrm{ATPMI}}(T_{\mathrm{M}}^{\substack{\circ\circ\\\circ\bullet\\\bullet\bullet}}) + \nu^{r}_{\mathrm{AKI}}(D_{\mathrm{I}}^{\substack{\circ\\\bullet\\\bullet}}D_{\mathrm{I}}^{\substack{\circ\\\circ\\\bullet}}) + \nu^{r}_{\mathrm{ATPIO}}(T_{\mathrm{O}}^{\substack{\circ\circ\\\circ\bullet\\\bullet\bullet}}) + \nu^{r}_{\mathrm{CKI}}(D_{\mathrm{I}}^{\substack{\circ\\\circ\\\bullet}}C_{\mathrm{I}}^{\substack{\circ\\\bullet\\\bullet}}) - \nu^{f}_{\mathrm{AKI}}(T_{\mathrm{I}}^{\substack{\circ\circ\\\circ\bullet\\\bullet\bullet}}) - \nu^{f}_{\mathrm{ATPIO}}(T_{\mathrm{I}}^{\substack{\circ\circ\\\circ\bullet\\\bullet\bullet}}) - \nu^{f}_{\mathrm{CKI}}(T_{\mathrm{I}}^{\substack{\circ\circ\\\circ\bullet\\\bullet\bullet}}) - \nu^{r}_{\mathrm{ATPMI}}(T_{\mathrm{I}}^{\substack{\circ\circ\\\circ\bullet\\\bullet\bullet}})$$

$$\frac{dT_{\mathrm{I}}^{\substack{\circ\bullet\\\circ\circ\\\bullet\circ}}}{dt} = \nu^{f}_{\mathrm{ATPMI}}(T_{\mathrm{M}}^{\substack{\circ\bullet\\\circ\circ\\\bullet\circ}}) + \nu^{r}_{\mathrm{AKI}}(D_{\mathrm{I}}^{\substack{\bullet\\\circ\\\circ}}D_{\mathrm{I}}^{\substack{\circ\\\circ\\\bullet}}) + \nu^{r}_{\mathrm{ATPIO}}(T_{\mathrm{O}}^{\substack{\circ\bullet\\\circ\circ\\\bullet\circ}}) + \nu^{r}_{\mathrm{CKI}}(D_{\mathrm{I}}^{\substack{\circ\\\circ\\\bullet}}C_{\mathrm{I}}^{\substack{\bullet\\\circ\\\circ}}) - \nu^{f}_{\mathrm{AKI}}(T_{\mathrm{I}}^{\substack{\circ\bullet\\\circ\circ\\\bullet\circ}}) - \nu^{f}_{\mathrm{ATPIO}}(T_{\mathrm{I}}^{\substack{\circ\bullet\\\circ\circ\\\bullet\circ}}) - \nu^{f}_{\mathrm{CKI}}(T_{\mathrm{I}}^{\substack{\circ\bullet\\\circ\circ\\\bullet\circ}}) - \nu^{r}_{\mathrm{ATPMI}}(T_{\mathrm{I}}^{\substack{\circ\bullet\\\circ\circ\\\bullet\circ}})$$

$$\frac{dT_{\mathrm{I}}^{\substack{\circ\bullet\\\circ\circ\\\bullet\bullet}}}{dt} = \nu^{f}_{\mathrm{ATPMI}}(T_{\mathrm{M}}^{\substack{\circ\bullet\\\circ\circ\\\bullet\bullet}}) + \nu^{r}_{\mathrm{AKI}}(D_{\mathrm{I}}^{\substack{\bullet\\\circ\\\bullet}}D_{\mathrm{I}}^{\substack{\circ\\\circ\\\bullet}}) + \nu^{r}_{\mathrm{ATPIO}}(T_{\mathrm{O}}^{\substack{\circ\bullet\\\circ\circ\\\bullet\bullet}}) + \nu^{r}_{\mathrm{CKI}}(D_{\mathrm{I}}^{\substack{\circ\\\circ\\\bullet}}C_{\mathrm{I}}^{\substack{\bullet\\\circ\\\bullet}}) - \nu^{f}_{\mathrm{AKI}}(T_{\mathrm{I}}^{\substack{\circ\bullet\\\circ\circ\\\bullet\bullet}}) - \nu^{f}_{\mathrm{ATPIO}}(T_{\mathrm{I}}^{\substack{\circ\bullet\\\circ\circ\\\bullet\bullet}}) - \nu^{f}_{\mathrm{CKI}}(T_{\mathrm{I}}^{\substack{\circ\bullet\\\circ\circ\\\bullet\bullet}}) - \nu^{r}_{\mathrm{ATPMI}}(T_{\mathrm{I}}^{\substack{\circ\bullet\\\circ\circ\\\bullet\bullet}})$$

$$\frac{dT_{\mathrm{I}}^{\substack{\circ\bullet\\\circ\bullet\\\bullet\circ}}}{dt} = \nu^{f}_{\mathrm{ATPMI}}(T_{\mathrm{M}}^{\substack{\circ\bullet\\\circ\bullet\\\bullet\circ}}) + \nu^{r}_{\mathrm{AKI}}(D_{\mathrm{I}}^{\substack{\bullet\\\bullet\\\circ}}D_{\mathrm{I}}^{\substack{\circ\\\circ\\\bullet}}) + \nu^{r}_{\mathrm{ATPIO}}(T_{\mathrm{O}}^{\substack{\circ\bullet\\\circ\bullet\\\bullet\circ}}) + \nu^{r}_{\mathrm{CKI}}(D_{\mathrm{I}}^{\substack{\circ\\\circ\\\bullet}}C_{\mathrm{I}}^{\substack{\bullet\\\bullet\\\circ}}) - \nu^{f}_{\mathrm{AKI}}(T_{\mathrm{I}}^{\substack{\circ\bullet\\\circ\bullet\\\bullet\circ}}) - \nu^{f}_{\mathrm{ATPIO}}(T_{\mathrm{I}}^{\substack{\circ\bullet\\\circ\bullet\\\bullet\circ}}) - \nu^{f}_{\mathrm{CKI}}(T_{\mathrm{I}}^{\substack{\circ\bullet\\\circ\bullet\\\bullet\circ}}) - \nu^{r}_{\mathrm{ATPMI}}(T_{\mathrm{I}}^{\substack{\circ\bullet\\\circ\bullet\\\bullet\circ}})$$

$$\frac{dT_{\mathrm{I}}^{\substack{\circ\bullet\\\circ\bullet\\\bullet\bullet}}}{dt} = \nu^{f}_{\mathrm{ATPMI}}(T_{\mathrm{M}}^{\substack{\circ\bullet\\\circ\bullet\\\bullet\bullet}}) + \nu^{r}_{\mathrm{AKI}}(D_{\mathrm{I}}^{\substack{\bullet\\\bullet\\\bullet}}D_{\mathrm{I}}^{\substack{\circ\\\circ\\\bullet}}) + \nu^{r}_{\mathrm{ATPIO}}(T_{\mathrm{O}}^{\substack{\circ\bullet\\\circ\bullet\\\bullet\bullet}}) + \nu^{r}_{\mathrm{CKI}}(D_{\mathrm{I}}^{\substack{\circ\\\circ\\\bullet}}C_{\mathrm{I}}^{\substack{\bullet\\\bullet\\\bullet}}) - \nu^{f}_{\mathrm{AKI}}(T_{\mathrm{I}}^{\substack{\circ\bullet\\\circ\bullet\\\bullet\bullet}}) - \nu^{f}_{\mathrm{ATPIO}}(T_{\mathrm{I}}^{\substack{\circ\bullet\\\circ\bullet\\\bullet\bullet}}) - \nu^{f}_{\mathrm{CKI}}(T_{\mathrm{I}}^{\substack{\circ\bullet\\\circ\bullet\\\bullet\bullet}}) - \nu^{r}_{\mathrm{ATPMI}}(T_{\mathrm{I}}^{\substack{\circ\bullet\\\circ\bullet\\\bullet\bullet}})$$

$$\frac{dT_{\mathrm{I}}^{\substack{\circ\circ\\\bullet\circ\\\circ\circ}}}{dt} = \nu^{f}_{\mathrm{ATPMI}}(T_{\mathrm{M}}^{\substack{\circ\circ\\\bullet\circ\\\circ\circ}}) + \nu^{r}_{\mathrm{AKI}}(D_{\mathrm{I}}^{\substack{\circ\\\bullet\\\circ}}D_{\mathrm{I}}^{\substack{\circ\\\circ\\\circ}}) + \nu^{r}_{\mathrm{ATPIO}}(T_{\mathrm{O}}^{\substack{\circ\circ\\\bullet\circ\\\circ\circ}}) + \nu^{r}_{\mathrm{CKI}}(D_{\mathrm{I}}^{\substack{\circ\\\bullet\\\circ}}C_{\mathrm{I}}^{\substack{\circ\\\circ\\\circ}}) - \nu^{f}_{\mathrm{AKI}}(T_{\mathrm{I}}^{\substack{\circ\circ\\\bullet\circ\\\circ\circ}}) - \nu^{f}_{\mathrm{ATPIO}}(T_{\mathrm{I}}^{\substack{\circ\circ\\\bullet\circ\\\circ\circ}}) - \nu^{f}_{\mathrm{CKI}}(T_{\mathrm{I}}^{\substack{\circ\circ\\\bullet\circ\\\circ\circ}}) - \nu^{r}_{\mathrm{ATPMI}}(T_{\mathrm{I}}^{\substack{\circ\circ\\\bullet\circ\\\circ\circ}})$$

$$\frac{dT_{\mathrm{I}}^{\substack{\circ\circ\\\bullet\circ\\\circ\bullet}}}{dt} = \nu^{f}_{\mathrm{ATPMI}}(T_{\mathrm{M}}^{\substack{\circ\circ\\\bullet\circ\\\circ\bullet}}) + \nu^{r}_{\mathrm{AKI}}(D_{\mathrm{I}}^{\substack{\circ\\\bullet\\\circ}}D_{\mathrm{I}}^{\substack{\circ\\\circ\\\bullet}}) + \nu^{r}_{\mathrm{ATPIO}}(T_{\mathrm{O}}^{\substack{\circ\circ\\\bullet\circ\\\circ\bullet}}) + \nu^{r}_{\mathrm{CKI}}(D_{\mathrm{I}}^{\substack{\circ\\\bullet\\\circ}}C_{\mathrm{I}}^{\substack{\circ\\\circ\\\bullet}}) - \nu^{f}_{\mathrm{AKI}}(T_{\mathrm{I}}^{\substack{\circ\circ\\\bullet\circ\\\circ\bullet}}) - \nu^{f}_{\mathrm{ATPIO}}(T_{\mathrm{I}}^{\substack{\circ\circ\\\bullet\circ\\\circ\bullet}}) - \nu^{f}_{\mathrm{CKI}}(T_{\mathrm{I}}^{\substack{\circ\circ\\\bullet\circ\\\circ\bullet}}) - \nu^{r}_{\mathrm{ATPMI}}(T_{\mathrm{I}}^{\substack{\circ\circ\\\bullet\circ\\\circ\bullet}})$$

$$\frac{dT_{\mathrm{I}}^{\substack{\circ\circ\\\bullet\bullet\\\circ\circ}}}{dt} = \nu^{f}_{\mathrm{ATPMI}}(T_{\mathrm{M}}^{\substack{\circ\circ\\\bullet\bullet\\\circ\circ}}) + \nu^{r}_{\mathrm{AKI}}(D_{\mathrm{I}}^{\substack{\circ\\\bullet\\\circ}}D_{\mathrm{I}}^{\substack{\circ\\\bullet\\\circ}}) + \nu^{r}_{\mathrm{ATPIO}}(T_{\mathrm{O}}^{\substack{\circ\circ\\\bullet\bullet\\\circ\circ}}) + \nu^{r}_{\mathrm{CKI}}(D_{\mathrm{I}}^{\substack{\circ\\\bullet\\\circ}}C_{\mathrm{I}}^{\substack{\circ\\\bullet\\\circ}}) - \nu^{f}_{\mathrm{AKI}}(T_{\mathrm{I}}^{\substack{\circ\circ\\\bullet\bullet\\\circ\circ}}) - \nu^{f}_{\mathrm{ATPIO}}(T_{\mathrm{I}}^{\substack{\circ\circ\\\bullet\bullet\\\circ\circ}}) - \nu^{f}_{\mathrm{CKI}}(T_{\mathrm{I}}^{\substack{\circ\circ\\\bullet\bullet\\\circ\circ}}) - \nu^{r}_{\mathrm{ATPMI}}(T_{\mathrm{I}}^{\substack{\circ\circ\\\bullet\bullet\\\circ\circ}})$$

$$\frac{dT_{\mathrm{I}}^{\substack{\circ\circ\\\bullet\bullet\\\circ\bullet}}}{dt} = \nu^{f}_{\mathrm{ATPMI}}(T_{\mathrm{M}}^{\substack{\circ\circ\\\bullet\bullet\\\circ\bullet}}) + \nu^{r}_{\mathrm{AKI}}(D_{\mathrm{I}}^{\substack{\circ\\\bullet\\\bullet}}D_{\mathrm{I}}^{\substack{\circ\\\bullet\\\circ}}) + \nu^{r}_{\mathrm{ATPIO}}(T_{\mathrm{O}}^{\substack{\circ\circ\\\bullet\bullet\\\circ\bullet}}) + \nu^{r}_{\mathrm{CKI}}(D_{\mathrm{I}}^{\substack{\circ\\\bullet\\\circ}}C_{\mathrm{I}}^{\substack{\circ\\\bullet\\\bullet}}) - \nu^{f}_{\mathrm{AKI}}(T_{\mathrm{I}}^{\substack{\circ\circ\\\bullet\bullet\\\circ\bullet}}) - \nu^{f}_{\mathrm{ATPIO}}(T_{\mathrm{I}}^{\substack{\circ\circ\\\bullet\bullet\\\circ\bullet}}) - \nu^{f}_{\mathrm{CKI}}(T_{\mathrm{I}}^{\substack{\circ\circ\\\bullet\bullet\\\circ\bullet}}) - \nu^{r}_{\mathrm{ATPMI}}(T_{\mathrm{I}}^{\substack{\circ\circ\\\bullet\bullet\\\circ\bullet}})$$

$$\frac{dT_{\mathrm{I}}^{\substack{\circ\bullet\\\bullet\circ\\\circ\circ}}}{dt} = \nu^{f}_{\mathrm{ATPMI}}(T_{\mathrm{M}}^{\substack{\circ\bullet\\\bullet\circ\\\circ\circ}}) + \nu^{r}_{\mathrm{AKI}}(D_{\mathrm{I}}^{\substack{\bullet\\\circ\\\circ}}D_{\mathrm{I}}^{\substack{\circ\\\bullet\\\circ}}) + \nu^{r}_{\mathrm{ATPIO}}(T_{\mathrm{O}}^{\substack{\circ\bullet\\\bullet\circ\\\circ\circ}}) + \nu^{r}_{\mathrm{CKI}}(D_{\mathrm{I}}^{\substack{\circ\\\bullet\\\circ}}C_{\mathrm{I}}^{\substack{\bullet\\\circ\\\circ}}) - \nu^{f}_{\mathrm{AKI}}(T_{\mathrm{I}}^{\substack{\circ\bullet\\\bullet\circ\\\circ\circ}}) - \nu^{f}_{\mathrm{ATPIO}}(T_{\mathrm{I}}^{\substack{\circ\bullet\\\bullet\circ\\\circ\circ}}) - \nu^{f}_{\mathrm{CKI}}(T_{\mathrm{I}}^{\substack{\circ\bullet\\\bullet\circ\\\circ\circ}}) - \nu^{r}_{\mathrm{ATPMI}}(T_{\mathrm{I}}^{\substack{\circ\bullet\\\bullet\circ\\\circ\circ}})$$

$$\frac{d\mathrm{T_I}^{\substack{\circ\bullet\\\bullet\circ\\\circ\bullet}}}{dt} = \underset{\mathrm{ATPMI}}{\nu_f}\left(\mathrm{T_M}^{\substack{\circ\bullet\\\bullet\circ\\\circ\bullet}}\right) + \underset{\mathrm{AKI}}{\nu_r}\left(\mathrm{D_I}^{\substack{\bullet\\\circ\\\bullet}}\mathrm{D_I}^{\substack{\circ\\\bullet\\\circ}}\right) + \underset{\mathrm{ATPIO}}{\nu_r}\left(\mathrm{T_O}^{\substack{\circ\bullet\\\bullet\circ\\\circ\bullet}}\right) + \underset{\mathrm{CKI}}{\nu_r}\left(\mathrm{D_I}^{\substack{\circ\\\bullet\\\circ}}\mathrm{C_I}^{\substack{\bullet\\\circ\\\bullet}}\right) - \underset{\mathrm{AKI}}{\nu_f}\left(\mathrm{T_I}^{\substack{\circ\bullet\\\bullet\circ\\\circ\bullet}}\right) - \underset{\mathrm{ATPIO}}{\nu_f}\left(\mathrm{T_I}^{\substack{\circ\bullet\\\bullet\circ\\\circ\bullet}}\right) - \underset{\mathrm{CKI}}{\nu_f}\left(\mathrm{T_I}^{\substack{\circ\bullet\\\bullet\circ\\\circ\bullet}}\right) - \underset{\mathrm{ATPMI}}{\nu_r}\left(\mathrm{T_I}^{\substack{\circ\bullet\\\bullet\circ\\\circ\bullet}}\right)$$

$$\frac{d\mathrm{T_I}^{\substack{\circ\bullet\\\bullet\bullet\\\circ\circ}}}{dt} = \underset{\mathrm{ATPMI}}{\nu_f}\left(\mathrm{T_M}^{\substack{\circ\bullet\\\bullet\bullet\\\circ\circ}}\right) + \underset{\mathrm{AKI}}{\nu_r}\left(\mathrm{D_I}^{\substack{\bullet\\\bullet\\\circ}}\mathrm{D_I}^{\substack{\circ\\\bullet\\\circ}}\right) + \underset{\mathrm{ATPIO}}{\nu_r}\left(\mathrm{T_O}^{\substack{\circ\bullet\\\bullet\bullet\\\circ\circ}}\right) + \underset{\mathrm{CKI}}{\nu_r}\left(\mathrm{D_I}^{\substack{\circ\\\bullet\\\circ}}\mathrm{C_I}^{\substack{\bullet\\\bullet\\\circ}}\right) - \underset{\mathrm{AKI}}{\nu_f}\left(\mathrm{T_I}^{\substack{\circ\bullet\\\bullet\bullet\\\circ\circ}}\right) - \underset{\mathrm{ATPIO}}{\nu_f}\left(\mathrm{T_I}^{\substack{\circ\bullet\\\bullet\bullet\\\circ\circ}}\right) - \underset{\mathrm{CKI}}{\nu_f}\left(\mathrm{T_I}^{\substack{\circ\bullet\\\bullet\bullet\\\circ\circ}}\right) - \underset{\mathrm{ATPMI}}{\nu_r}\left(\mathrm{T_I}^{\substack{\circ\bullet\\\bullet\bullet\\\circ\circ}}\right)$$

$$\frac{d\mathrm{T_I}^{\substack{\circ\bullet\\\bullet\bullet\\\circ\bullet}}}{dt} = \underset{\mathrm{ATPMI}}{\nu_f}\left(\mathrm{T_M}^{\substack{\circ\bullet\\\bullet\bullet\\\circ\bullet}}\right) + \underset{\mathrm{AKI}}{\nu_r}\left(\mathrm{D_I}^{\substack{\bullet\\\bullet\\\bullet}}\mathrm{D_I}^{\substack{\circ\\\bullet\\\circ}}\right) + \underset{\mathrm{ATPIO}}{\nu_r}\left(\mathrm{T_O}^{\substack{\circ\bullet\\\bullet\bullet\\\circ\bullet}}\right) + \underset{\mathrm{CKI}}{\nu_r}\left(\mathrm{D_I}^{\substack{\circ\\\bullet\\\circ}}\mathrm{C_I}^{\substack{\bullet\\\bullet\\\bullet}}\right) - \underset{\mathrm{AKI}}{\nu_f}\left(\mathrm{T_I}^{\substack{\circ\bullet\\\bullet\bullet\\\circ\bullet}}\right) - \underset{\mathrm{ATPIO}}{\nu_f}\left(\mathrm{T_I}^{\substack{\circ\bullet\\\bullet\bullet\\\circ\bullet}}\right) - \underset{\mathrm{CKI}}{\nu_f}\left(\mathrm{T_I}^{\substack{\circ\bullet\\\bullet\bullet\\\circ\bullet}}\right) - \underset{\mathrm{ATPMI}}{\nu_r}\left(\mathrm{T_I}^{\substack{\circ\bullet\\\bullet\bullet\\\circ\bullet}}\right)$$

$$\frac{d\mathrm{T_I}^{\substack{\circ\circ\\\bullet\circ\\\bullet\circ}}}{dt} = \underset{\mathrm{ATPMI}}{\nu_f}\left(\mathrm{T_M}^{\substack{\circ\circ\\\bullet\circ\\\bullet\circ}}\right) + \underset{\mathrm{AKI}}{\nu_r}\left(\mathrm{D_I}^{\substack{\circ\\\bullet\\\bullet}}\mathrm{D_I}^{\substack{\circ\\\circ\\\circ}}\right) + \underset{\mathrm{ATPIO}}{\nu_r}\left(\mathrm{T_O}^{\substack{\circ\circ\\\bullet\circ\\\bullet\circ}}\right) + \underset{\mathrm{CKI}}{\nu_r}\left(\mathrm{D_I}^{\substack{\circ\\\bullet\\\bullet}}\mathrm{C_I}^{\substack{\circ\\\circ\\\circ}}\right) - \underset{\mathrm{AKI}}{\nu_f}\left(\mathrm{T_I}^{\substack{\circ\circ\\\bullet\circ\\\bullet\circ}}\right) - \underset{\mathrm{ATPIO}}{\nu_f}\left(\mathrm{T_I}^{\substack{\circ\circ\\\bullet\circ\\\bullet\circ}}\right) - \underset{\mathrm{CKI}}{\nu_f}\left(\mathrm{T_I}^{\substack{\circ\circ\\\bullet\circ\\\bullet\circ}}\right) - \underset{\mathrm{ATPMI}}{\nu_r}\left(\mathrm{T_I}^{\substack{\circ\circ\\\bullet\circ\\\bullet\circ}}\right)$$

$$\frac{d\mathrm{T_I}^{\substack{\circ\circ\\\bullet\circ\\\bullet\bullet}}}{dt} = \underset{\mathrm{ATPMI}}{\nu_f}\left(\mathrm{T_M}^{\substack{\circ\circ\\\bullet\circ\\\bullet\bullet}}\right) + \underset{\mathrm{AKI}}{\nu_r}\left(\mathrm{D_I}^{\substack{\circ\\\bullet\\\bullet}}\mathrm{D_I}^{\substack{\circ\\\circ\\\bullet}}\right) + \underset{\mathrm{ATPIO}}{\nu_r}\left(\mathrm{T_O}^{\substack{\circ\circ\\\bullet\circ\\\bullet\bullet}}\right) + \underset{\mathrm{CKI}}{\nu_r}\left(\mathrm{D_I}^{\substack{\circ\\\bullet\\\bullet}}\mathrm{C_I}^{\substack{\circ\\\circ\\\bullet}}\right) - \underset{\mathrm{AKI}}{\nu_f}\left(\mathrm{T_I}^{\substack{\circ\circ\\\bullet\circ\\\bullet\bullet}}\right) - \underset{\mathrm{ATPIO}}{\nu_f}\left(\mathrm{T_I}^{\substack{\circ\circ\\\bullet\circ\\\bullet\bullet}}\right) - \underset{\mathrm{CKI}}{\nu_f}\left(\mathrm{T_I}^{\substack{\circ\circ\\\bullet\circ\\\bullet\bullet}}\right) - \underset{\mathrm{ATPMI}}{\nu_r}\left(\mathrm{T_I}^{\substack{\circ\circ\\\bullet\circ\\\bullet\bullet}}\right)$$

$$\frac{d\mathrm{T_I}^{\substack{\circ\circ\\\bullet\bullet\\\bullet\circ}}}{dt} = \underset{\mathrm{ATPMI}}{\nu_f}\left(\mathrm{T_M}^{\substack{\circ\circ\\\bullet\bullet\\\bullet\circ}}\right) + \underset{\mathrm{AKI}}{\nu_r}\left(\mathrm{D_I}^{\substack{\circ\\\bullet\\\bullet}}\mathrm{D_I}^{\substack{\circ\\\bullet\\\circ}}\right) + \underset{\mathrm{ATPIO}}{\nu_r}\left(\mathrm{T_O}^{\substack{\circ\circ\\\bullet\bullet\\\bullet\circ}}\right) + \underset{\mathrm{CKI}}{\nu_r}\left(\mathrm{D_I}^{\substack{\circ\\\bullet\\\bullet}}\mathrm{C_I}^{\substack{\circ\\\bullet\\\circ}}\right) - \underset{\mathrm{AKI}}{\nu_f}\left(\mathrm{T_I}^{\substack{\circ\circ\\\bullet\bullet\\\bullet\circ}}\right) - \underset{\mathrm{ATPIO}}{\nu_f}\left(\mathrm{T_I}^{\substack{\circ\circ\\\bullet\bullet\\\bullet\circ}}\right) - \underset{\mathrm{CKI}}{\nu_f}\left(\mathrm{T_I}^{\substack{\circ\circ\\\bullet\bullet\\\bullet\circ}}\right) - \underset{\mathrm{ATPMI}}{\nu_r}\left(\mathrm{T_I}^{\substack{\circ\circ\\\bullet\bullet\\\bullet\circ}}\right)$$

$$\frac{d\mathrm{T_I}^{\substack{\circ\circ\\\bullet\bullet\\\bullet\bullet}}}{dt} = \underset{\mathrm{ATPMI}}{\nu_f}\left(\mathrm{T_M}^{\substack{\circ\circ\\\bullet\bullet\\\bullet\bullet}}\right) + \underset{\mathrm{AKI}}{\nu_r}\left(\mathrm{D_I}^{\substack{\circ\\\bullet\\\bullet}}\mathrm{D_I}^{\substack{\circ\\\bullet\\\bullet}}\right) + \underset{\mathrm{ATPIO}}{\nu_r}\left(\mathrm{T_O}^{\substack{\circ\circ\\\bullet\bullet\\\bullet\bullet}}\right) + \underset{\mathrm{CKI}}{\nu_r}\left(\mathrm{D_I}^{\substack{\circ\\\bullet\\\bullet}}\mathrm{C_I}^{\substack{\circ\\\bullet\\\bullet}}\right) - \underset{\mathrm{AKI}}{\nu_f}\left(\mathrm{T_I}^{\substack{\circ\circ\\\bullet\bullet\\\bullet\bullet}}\right) - \underset{\mathrm{ATPIO}}{\nu_f}\left(\mathrm{T_I}^{\substack{\circ\circ\\\bullet\bullet\\\bullet\bullet}}\right) - \underset{\mathrm{CKI}}{\nu_f}\left(\mathrm{T_I}^{\substack{\circ\circ\\\bullet\bullet\\\bullet\bullet}}\right) - \underset{\mathrm{ATPMI}}{\nu_r}\left(\mathrm{T_I}^{\substack{\circ\circ\\\bullet\bullet\\\bullet\bullet}}\right)$$

$$\frac{d\mathrm{T_I}^{\substack{\circ\bullet\\\bullet\circ\\\bullet\circ}}}{dt} = \underset{\mathrm{ATPMI}}{\nu_f}\left(\mathrm{T_M}^{\substack{\circ\bullet\\\bullet\circ\\\bullet\circ}}\right) + \underset{\mathrm{AKI}}{\nu_r}\left(\mathrm{D_I}^{\substack{\bullet\\\circ\\\circ}}\mathrm{D_I}^{\substack{\circ\\\bullet\\\bullet}}\right) + \underset{\mathrm{ATPIO}}{\nu_r}\left(\mathrm{T_O}^{\substack{\circ\bullet\\\bullet\circ\\\bullet\circ}}\right) + \underset{\mathrm{CKI}}{\nu_r}\left(\mathrm{D_I}^{\substack{\circ\\\bullet\\\bullet}}\mathrm{C_I}^{\substack{\bullet\\\circ\\\circ}}\right) - \underset{\mathrm{AKI}}{\nu_f}\left(\mathrm{T_I}^{\substack{\circ\bullet\\\bullet\circ\\\bullet\circ}}\right) - \underset{\mathrm{ATPIO}}{\nu_f}\left(\mathrm{T_I}^{\substack{\circ\bullet\\\bullet\circ\\\bullet\circ}}\right) - \underset{\mathrm{CKI}}{\nu_f}\left(\mathrm{T_I}^{\substack{\circ\bullet\\\bullet\circ\\\bullet\circ}}\right) - \underset{\mathrm{ATPMI}}{\nu_r}\left(\mathrm{T_I}^{\substack{\circ\bullet\\\bullet\circ\\\bullet\circ}}\right)$$

$$\frac{d\mathrm{T_I}^{\substack{\circ\bullet\\\bullet\circ\\\bullet\bullet}}}{dt} = \underset{\mathrm{ATPMI}}{\nu_f}\left(\mathrm{T_M}^{\substack{\circ\bullet\\\bullet\circ\\\bullet\bullet}}\right) + \underset{\mathrm{AKI}}{\nu_r}\left(\mathrm{D_I}^{\substack{\bullet\\\circ\\\bullet}}\mathrm{D_I}^{\substack{\circ\\\bullet\\\bullet}}\right) + \underset{\mathrm{ATPIO}}{\nu_r}\left(\mathrm{T_O}^{\substack{\circ\bullet\\\bullet\circ\\\bullet\bullet}}\right) + \underset{\mathrm{CKI}}{\nu_r}\left(\mathrm{D_I}^{\substack{\circ\\\bullet\\\bullet}}\mathrm{C_I}^{\substack{\bullet\\\circ\\\bullet}}\right) - \underset{\mathrm{AKI}}{\nu_f}\left(\mathrm{T_I}^{\substack{\circ\bullet\\\bullet\circ\\\bullet\bullet}}\right) - \underset{\mathrm{ATPIO}}{\nu_f}\left(\mathrm{T_I}^{\substack{\circ\bullet\\\bullet\circ\\\bullet\bullet}}\right) - \underset{\mathrm{CKI}}{\nu_f}\left(\mathrm{T_I}^{\substack{\circ\bullet\\\bullet\circ\\\bullet\bullet}}\right) - \underset{\mathrm{ATPMI}}{\nu_r}\left(\mathrm{T_I}^{\substack{\circ\bullet\\\bullet\circ\\\bullet\bullet}}\right)$$

$$\frac{d\mathrm{T_I}^{\substack{\circ\bullet\\\bullet\bullet\\\bullet\circ}}}{dt} = \underset{\mathrm{ATPMI}}{\nu_f}\left(\mathrm{T_M}^{\substack{\circ\bullet\\\bullet\bullet\\\bullet\circ}}\right) + \underset{\mathrm{AKI}}{\nu_r}\left(\mathrm{D_I}^{\substack{\bullet\\\bullet\\\circ}}\mathrm{D_I}^{\substack{\circ\\\bullet\\\bullet}}\right) + \underset{\mathrm{ATPIO}}{\nu_r}\left(\mathrm{T_O}^{\substack{\circ\bullet\\\bullet\bullet\\\bullet\circ}}\right) + \underset{\mathrm{CKI}}{\nu_r}\left(\mathrm{D_I}^{\substack{\circ\\\bullet\\\bullet}}\mathrm{C_I}^{\substack{\bullet\\\bullet\\\circ}}\right) - \underset{\mathrm{AKI}}{\nu_f}\left(\mathrm{T_I}^{\substack{\circ\bullet\\\bullet\bullet\\\bullet\circ}}\right) - \underset{\mathrm{ATPIO}}{\nu_f}\left(\mathrm{T_I}^{\substack{\circ\bullet\\\bullet\bullet\\\bullet\circ}}\right) - \underset{\mathrm{CKI}}{\nu_f}\left(\mathrm{T_I}^{\substack{\circ\bullet\\\bullet\bullet\\\bullet\circ}}\right) - \underset{\mathrm{ATPMI}}{\nu_r}\left(\mathrm{T_I}^{\substack{\circ\bullet\\\bullet\bullet\\\bullet\circ}}\right)$$

$$\frac{d\mathrm{T_I}^{\substack{\circ\bullet\\\bullet\bullet\\\bullet\bullet}}}{dt} = \underset{\mathrm{ATPMI}}{\nu_f}\left(\mathrm{T_M}^{\substack{\circ\bullet\\\bullet\bullet\\\bullet\bullet}}\right) + \underset{\mathrm{AKI}}{\nu_r}\left(\mathrm{D_I}^{\substack{\bullet\\\bullet\\\bullet}}\mathrm{D_I}^{\substack{\circ\\\bullet\\\bullet}}\right) + \underset{\mathrm{ATPIO}}{\nu_r}\left(\mathrm{T_O}^{\substack{\circ\bullet\\\bullet\bullet\\\bullet\bullet}}\right) + \underset{\mathrm{CKI}}{\nu_r}\left(\mathrm{D_I}^{\substack{\circ\\\bullet\\\bullet}}\mathrm{C_I}^{\substack{\bullet\\\bullet\\\bullet}}\right) - \underset{\mathrm{AKI}}{\nu_f}\left(\mathrm{T_I}^{\substack{\circ\bullet\\\bullet\bullet\\\bullet\bullet}}\right) - \underset{\mathrm{ATPIO}}{\nu_f}\left(\mathrm{T_I}^{\substack{\circ\bullet\\\bullet\bullet\\\bullet\bullet}}\right) - \underset{\mathrm{CKI}}{\nu_f}\left(\mathrm{T_I}^{\substack{\circ\bullet\\\bullet\bullet\\\bullet\bullet}}\right) - \underset{\mathrm{ATPMI}}{\nu_r}\left(\mathrm{T_I}^{\substack{\circ\bullet\\\bullet\bullet\\\bullet\bullet}}\right)$$

$$\frac{d\mathrm{T_I}^{\substack{\bullet\circ\\\circ\circ\\\circ\circ}}}{dt} = \underset{\mathrm{ATPMI}}{\nu_f}\left(\mathrm{T_M}^{\substack{\bullet\circ\\\circ\circ\\\circ\circ}}\right) + \underset{\mathrm{AKI}}{\nu_r}\left(\mathrm{D_I}^{\substack{\bullet\\\circ\\\circ}}\mathrm{D_I}^{\substack{\circ\\\circ\\\circ}}\right) + \underset{\mathrm{ATPIO}}{\nu_r}\left(\mathrm{T_O}^{\substack{\bullet\circ\\\circ\circ\\\circ\circ}}\right) + \underset{\mathrm{CKI}}{\nu_r}\left(\mathrm{D_I}^{\substack{\bullet\\\circ\\\circ}}\mathrm{C_I}^{\substack{\circ\\\circ\\\circ}}\right) - \underset{\mathrm{AKI}}{\nu_f}\left(\mathrm{T_I}^{\substack{\bullet\circ\\\circ\circ\\\circ\circ}}\right) - \underset{\mathrm{ATPIO}}{\nu_f}\left(\mathrm{T_I}^{\substack{\bullet\circ\\\circ\circ\\\circ\circ}}\right) - \underset{\mathrm{CKI}}{\nu_f}\left(\mathrm{T_I}^{\substack{\bullet\circ\\\circ\circ\\\circ\circ}}\right) - \underset{\mathrm{ATPMI}}{\nu_r}\left(\mathrm{T_I}^{\substack{\bullet\circ\\\circ\circ\\\circ\circ}}\right)$$

$$\frac{d\mathrm{T_I}^{\substack{\bullet\circ\\\circ\circ\\\circ\bullet}}}{dt} = \underset{\mathrm{ATPMI}}{\nu_f}\left(\mathrm{T_M}^{\substack{\bullet\circ\\\circ\circ\\\circ\bullet}}\right) + \underset{\mathrm{AKI}}{\nu_r}\left(\mathrm{D_I}^{\substack{\bullet\\\circ\\\circ}}\mathrm{D_I}^{\substack{\circ\\\circ\\\bullet}}\right) + \underset{\mathrm{ATPIO}}{\nu_r}\left(\mathrm{T_O}^{\substack{\bullet\circ\\\circ\circ\\\circ\bullet}}\right) + \underset{\mathrm{CKI}}{\nu_r}\left(\mathrm{D_I}^{\substack{\bullet\\\circ\\\circ}}\mathrm{C_I}^{\substack{\circ\\\circ\\\bullet}}\right) - \underset{\mathrm{AKI}}{\nu_f}\left(\mathrm{T_I}^{\substack{\bullet\circ\\\circ\circ\\\circ\bullet}}\right) - \underset{\mathrm{ATPIO}}{\nu_f}\left(\mathrm{T_I}^{\substack{\bullet\circ\\\circ\circ\\\circ\bullet}}\right) - \underset{\mathrm{CKI}}{\nu_f}\left(\mathrm{T_I}^{\substack{\bullet\circ\\\circ\circ\\\circ\bullet}}\right) - \underset{\mathrm{ATPMI}}{\nu_r}\left(\mathrm{T_I}^{\substack{\bullet\circ\\\circ\circ\\\circ\bullet}}\right)$$

$$\frac{d\mathrm{T_I}^{\substack{\bullet\circ\\\circ\bullet\\\circ\circ}}}{dt} = \underset{\mathrm{ATPMI}}{\nu_f}\left(\mathrm{T_M}^{\substack{\bullet\circ\\\circ\bullet\\\circ\circ}}\right) + \underset{\mathrm{AKI}}{\nu_r}\left(\mathrm{D_I}^{\substack{\bullet\\\circ\\\circ}}\mathrm{D_I}^{\substack{\circ\\\bullet\\\circ}}\right) + \underset{\mathrm{ATPIO}}{\nu_r}\left(\mathrm{T_O}^{\substack{\bullet\circ\\\circ\bullet\\\circ\circ}}\right) + \underset{\mathrm{CKI}}{\nu_r}\left(\mathrm{D_I}^{\substack{\bullet\\\circ\\\circ}}\mathrm{C_I}^{\substack{\circ\\\bullet\\\circ}}\right) - \underset{\mathrm{AKI}}{\nu_f}\left(\mathrm{T_I}^{\substack{\bullet\circ\\\circ\bullet\\\circ\circ}}\right) - \underset{\mathrm{ATPIO}}{\nu_f}\left(\mathrm{T_I}^{\substack{\bullet\circ\\\circ\bullet\\\circ\circ}}\right) - \underset{\mathrm{CKI}}{\nu_f}\left(\mathrm{T_I}^{\substack{\bullet\circ\\\circ\bullet\\\circ\circ}}\right) - \underset{\mathrm{ATPMI}}{\nu_r}\left(\mathrm{T_I}^{\substack{\bullet\circ\\\circ\bullet\\\circ\circ}}\right)$$

$$\frac{d\mathrm{T_I}^{\substack{\bullet\circ\\\circ\bullet\\\circ\bullet}}}{dt} = \underset{\mathrm{ATPMI}}{\nu_f}\left(\mathrm{T_M}^{\substack{\bullet\circ\\\circ\bullet\\\circ\bullet}}\right) + \underset{\mathrm{AKI}}{\nu_r}\left(\mathrm{D_I}^{\substack{\bullet\\\circ\\\circ}}\mathrm{D_I}^{\substack{\circ\\\bullet\\\bullet}}\right) + \underset{\mathrm{ATPIO}}{\nu_r}\left(\mathrm{T_O}^{\substack{\bullet\circ\\\circ\bullet\\\circ\bullet}}\right) + \underset{\mathrm{CKI}}{\nu_r}\left(\mathrm{D_I}^{\substack{\bullet\\\circ\\\circ}}\mathrm{C_I}^{\substack{\circ\\\bullet\\\bullet}}\right) - \underset{\mathrm{AKI}}{\nu_f}\left(\mathrm{T_I}^{\substack{\bullet\circ\\\circ\bullet\\\circ\bullet}}\right) - \underset{\mathrm{ATPIO}}{\nu_f}\left(\mathrm{T_I}^{\substack{\bullet\circ\\\circ\bullet\\\circ\bullet}}\right) - \underset{\mathrm{CKI}}{\nu_f}\left(\mathrm{T_I}^{\substack{\bullet\circ\\\circ\bullet\\\circ\bullet}}\right) - \underset{\mathrm{ATPMI}}{\nu_r}\left(\mathrm{T_I}^{\substack{\bullet\circ\\\circ\bullet\\\circ\bullet}}\right)$$

$$\frac{d\mathrm{T_I}^{\substack{\bullet\bullet\\\circ\circ\\\circ\circ}}}{dt} = \underset{\mathrm{ATPMI}}{\nu_f}\left(\mathrm{T_M}^{\substack{\bullet\bullet\\\circ\circ\\\circ\circ}}\right) + \underset{\mathrm{AKI}}{\nu_r}\left(\mathrm{D_I}^{\substack{\bullet\\\circ\\\circ}}\mathrm{D_I}^{\substack{\bullet\\\circ\\\circ}}\right) + \underset{\mathrm{ATPIO}}{\nu_r}\left(\mathrm{T_O}^{\substack{\bullet\bullet\\\circ\circ\\\circ\circ}}\right) + \underset{\mathrm{CKI}}{\nu_r}\left(\mathrm{D_I}^{\substack{\bullet\\\circ\\\circ}}\mathrm{C_I}^{\substack{\bullet\\\circ\\\circ}}\right) - \underset{\mathrm{AKI}}{\nu_f}\left(\mathrm{T_I}^{\substack{\bullet\bullet\\\circ\circ\\\circ\circ}}\right) - \underset{\mathrm{ATPIO}}{\nu_f}\left(\mathrm{T_I}^{\substack{\bullet\bullet\\\circ\circ\\\circ\circ}}\right) - \underset{\mathrm{CKI}}{\nu_f}\left(\mathrm{T_I}^{\substack{\bullet\bullet\\\circ\circ\\\circ\circ}}\right) - \underset{\mathrm{ATPMI}}{\nu_r}\left(\mathrm{T_I}^{\substack{\bullet\bullet\\\circ\circ\\\circ\circ}}\right)$$

$$\frac{d\mathrm{T_I}^{\substack{\bullet\bullet\\\circ\circ\\\circ\bullet}}}{dt} = \underset{\mathrm{ATPMI}}{\nu_f}\left(\mathrm{T_M}^{\substack{\bullet\bullet\\\circ\circ\\\circ\bullet}}\right) + \underset{\mathrm{AKI}}{\nu_r}\left(\mathrm{D_I}^{\substack{\bullet\\\circ\\\bullet}}\mathrm{D_I}^{\substack{\bullet\\\circ\\\circ}}\right) + \underset{\mathrm{ATPIO}}{\nu_r}\left(\mathrm{T_O}^{\substack{\bullet\bullet\\\circ\circ\\\circ\bullet}}\right) + \underset{\mathrm{CKI}}{\nu_r}\left(\mathrm{D_I}^{\substack{\bullet\\\circ\\\circ}}\mathrm{C_I}^{\substack{\bullet\\\circ\\\bullet}}\right) - \underset{\mathrm{AKI}}{\nu_f}\left(\mathrm{T_I}^{\substack{\bullet\bullet\\\circ\circ\\\circ\bullet}}\right) - \underset{\mathrm{ATPIO}}{\nu_f}\left(\mathrm{T_I}^{\substack{\bullet\bullet\\\circ\circ\\\circ\bullet}}\right) - \underset{\mathrm{CKI}}{\nu_f}\left(\mathrm{T_I}^{\substack{\bullet\bullet\\\circ\circ\\\circ\bullet}}\right) - \underset{\mathrm{ATPMI}}{\nu_r}\left(\mathrm{T_I}^{\substack{\bullet\bullet\\\circ\circ\\\circ\bullet}}\right)$$

$$\frac{d\mathrm{T_I}^{\substack{\bullet\bullet\\\circ\bullet\\\circ\circ}}}{dt} = \underset{\mathrm{ATPMI}}{\nu_f}\left(\mathrm{T_M}^{\substack{\bullet\bullet\\\circ\bullet\\\circ\circ}}\right) + \underset{\mathrm{AKI}}{\nu_r}\left(\mathrm{D_I}^{\substack{\bullet\\\bullet\\\circ}}\mathrm{D_I}^{\substack{\bullet\\\circ\\\circ}}\right) + \underset{\mathrm{ATPIO}}{\nu_r}\left(\mathrm{T_O}^{\substack{\bullet\bullet\\\circ\bullet\\\circ\circ}}\right) + \underset{\mathrm{CKI}}{\nu_r}\left(\mathrm{D_I}^{\substack{\bullet\\\circ\\\circ}}\mathrm{C_I}^{\substack{\bullet\\\bullet\\\circ}}\right) - \underset{\mathrm{AKI}}{\nu_f}\left(\mathrm{T_I}^{\substack{\bullet\bullet\\\circ\bullet\\\circ\circ}}\right) - \underset{\mathrm{ATPIO}}{\nu_f}\left(\mathrm{T_I}^{\substack{\bullet\bullet\\\circ\bullet\\\circ\circ}}\right) - \underset{\mathrm{CKI}}{\nu_f}\left(\mathrm{T_I}^{\substack{\bullet\bullet\\\circ\bullet\\\circ\circ}}\right) - \underset{\mathrm{ATPMI}}{\nu_r}\left(\mathrm{T_I}^{\substack{\bullet\bullet\\\circ\bullet\\\circ\circ}}\right)$$

$$\frac{d\mathrm{T_I}^{\substack{\bullet\bullet\\\circ\bullet\\\circ\bullet}}}{dt} = \underset{\mathrm{ATPMI}}{\nu_f}\left(\mathrm{T_M}^{\substack{\bullet\bullet\\\circ\bullet\\\circ\bullet}}\right) + \underset{\mathrm{AKI}}{\nu_r}\left(\mathrm{D_I}^{\substack{\bullet\\\bullet\\\bullet}}\mathrm{D_I}^{\substack{\bullet\\\circ\\\circ}}\right) + \underset{\mathrm{ATPIO}}{\nu_r}\left(\mathrm{T_O}^{\substack{\bullet\bullet\\\circ\bullet\\\circ\bullet}}\right) + \underset{\mathrm{CKI}}{\nu_r}\left(\mathrm{D_I}^{\substack{\bullet\\\circ\\\circ}}\mathrm{C_I}^{\substack{\bullet\\\bullet\\\bullet}}\right) - \underset{\mathrm{AKI}}{\nu_f}\left(\mathrm{T_I}^{\substack{\bullet\bullet\\\circ\bullet\\\circ\bullet}}\right) - \underset{\mathrm{ATPIO}}{\nu_f}\left(\mathrm{T_I}^{\substack{\bullet\bullet\\\circ\bullet\\\circ\bullet}}\right) - \underset{\mathrm{CKI}}{\nu_f}\left(\mathrm{T_I}^{\substack{\bullet\bullet\\\circ\bullet\\\circ\bullet}}\right) - \underset{\mathrm{ATPMI}}{\nu_r}\left(\mathrm{T_I}^{\substack{\bullet\bullet\\\circ\bullet\\\circ\bullet}}\right)$$

$$\frac{d\mathrm{T_I}^{\substack{\bullet\circ\\\circ\circ\\\bullet\circ}}}{dt} = \underset{\mathrm{ATPMI}}{\nu_f}\left(\mathrm{T_M}^{\substack{\bullet\circ\\\circ\circ\\\bullet\circ}}\right) + \underset{\mathrm{AKI}}{\nu_r}\left(\mathrm{D_I}^{\substack{\bullet\\\circ\\\bullet}}\mathrm{D_I}^{\substack{\circ\\\circ\\\circ}}\right) + \underset{\mathrm{ATPIO}}{\nu_r}\left(\mathrm{T_O}^{\substack{\bullet\circ\\\circ\circ\\\bullet\circ}}\right) + \underset{\mathrm{CKI}}{\nu_r}\left(\mathrm{D_I}^{\substack{\bullet\\\circ\\\bullet}}\mathrm{C_I}^{\substack{\circ\\\circ\\\circ}}\right) - \underset{\mathrm{AKI}}{\nu_f}\left(\mathrm{T_I}^{\substack{\bullet\circ\\\circ\circ\\\bullet\circ}}\right) - \underset{\mathrm{ATPIO}}{\nu_f}\left(\mathrm{T_I}^{\substack{\bullet\circ\\\circ\circ\\\bullet\circ}}\right) - \underset{\mathrm{CKI}}{\nu_f}\left(\mathrm{T_I}^{\substack{\bullet\circ\\\circ\circ\\\bullet\circ}}\right) - \underset{\mathrm{ATPMI}}{\nu_r}\left(\mathrm{T_I}^{\substack{\bullet\circ\\\circ\circ\\\bullet\circ}}\right)$$

$$\frac{d\mathrm{T_I}^{\substack{\bullet\circ\\\circ\circ\\\bullet\bullet}}}{dt} = \underset{\mathrm{ATPMI}}{\nu_f}\left(\mathrm{T_M}^{\substack{\bullet\circ\\\circ\circ\\\bullet\bullet}}\right) + \underset{\mathrm{AKI}}{\nu_r}\left(\mathrm{D_I}^{\substack{\bullet\\\circ\\\bullet}}\mathrm{D_I}^{\substack{\circ\\\circ\\\bullet}}\right) + \underset{\mathrm{ATPIO}}{\nu_r}\left(\mathrm{T_O}^{\substack{\bullet\circ\\\circ\circ\\\bullet\bullet}}\right) + \underset{\mathrm{CKI}}{\nu_r}\left(\mathrm{D_I}^{\substack{\bullet\\\circ\\\bullet}}\mathrm{C_I}^{\substack{\circ\\\circ\\\bullet}}\right) - \underset{\mathrm{AKI}}{\nu_f}\left(\mathrm{T_I}^{\substack{\bullet\circ\\\circ\circ\\\bullet\bullet}}\right) - \underset{\mathrm{ATPIO}}{\nu_f}\left(\mathrm{T_I}^{\substack{\bullet\circ\\\circ\circ\\\bullet\bullet}}\right) - \underset{\mathrm{CKI}}{\nu_f}\left(\mathrm{T_I}^{\substack{\bullet\circ\\\circ\circ\\\bullet\bullet}}\right) - \underset{\mathrm{ATPMI}}{\nu_r}\left(\mathrm{T_I}^{\substack{\bullet\circ\\\circ\circ\\\bullet\bullet}}\right)$$

$$\frac{d\mathrm{T_I}\substack{\bullet\circ\\\circ\bullet\\\bullet\circ}}{dt} = \underset{\mathrm{ATP_{MI}}}{\nu_f}\left(\mathrm{T_M}\substack{\bullet\circ\\\circ\bullet\\\bullet\circ}\right) + \underset{\mathrm{AK_I}}{\nu_r}\left(\mathrm{D_I}\substack{\bullet\\\circ\\\bullet}\mathrm{D_I}\substack{\circ\\\bullet\\\circ}\right) + \underset{\mathrm{ATP_{IO}}}{\nu_r}\left(\mathrm{T_O}\substack{\bullet\circ\\\circ\bullet\\\bullet\circ}\right) + \underset{\mathrm{CK_I}}{\nu_r}\left(\mathrm{D_I}\substack{\bullet\\\circ\\\bullet}\mathrm{C_I}\substack{\circ\\\bullet\\\circ}\right) - \underset{\mathrm{AK_I}}{\nu_f}\left(\mathrm{T_I}\substack{\bullet\circ\\\circ\bullet\\\bullet\circ}\right) - \underset{\mathrm{ATP_{IO}}}{\nu_f}\left(\mathrm{T_I}\substack{\bullet\circ\\\circ\bullet\\\bullet\circ}\right) - \underset{\mathrm{CK_I}}{\nu_f}\left(\mathrm{T_I}\substack{\bullet\circ\\\circ\bullet\\\bullet\circ}\right) - \underset{\mathrm{ATP_{MI}}}{\nu_r}\left(\mathrm{T_I}\substack{\bullet\circ\\\circ\bullet\\\bullet\circ}\right)$$

$$\frac{d\mathrm{T_I}\substack{\bullet\circ\\\circ\bullet\\\bullet\bullet}}{dt} = \underset{\mathrm{ATP_{MI}}}{\nu_f}\left(\mathrm{T_M}\substack{\bullet\circ\\\circ\bullet\\\bullet\bullet}\right) + \underset{\mathrm{AK_I}}{\nu_r}\left(\mathrm{D_I}\substack{\bullet\\\circ\\\bullet}\mathrm{D_I}\substack{\circ\\\bullet\\\bullet}\right) + \underset{\mathrm{ATP_{IO}}}{\nu_r}\left(\mathrm{T_O}\substack{\bullet\circ\\\circ\bullet\\\bullet\bullet}\right) + \underset{\mathrm{CK_I}}{\nu_r}\left(\mathrm{D_I}\substack{\bullet\\\circ\\\bullet}\mathrm{C_I}\substack{\circ\\\bullet\\\bullet}\right) - \underset{\mathrm{AK_I}}{\nu_f}\left(\mathrm{T_I}\substack{\bullet\circ\\\circ\bullet\\\bullet\bullet}\right) - \underset{\mathrm{ATP_{IO}}}{\nu_f}\left(\mathrm{T_I}\substack{\bullet\circ\\\circ\bullet\\\bullet\bullet}\right) - \underset{\mathrm{CK_I}}{\nu_f}\left(\mathrm{T_I}\substack{\bullet\circ\\\circ\bullet\\\bullet\bullet}\right) - \underset{\mathrm{ATP_{MI}}}{\nu_r}\left(\mathrm{T_I}\substack{\bullet\circ\\\circ\bullet\\\bullet\bullet}\right)$$

$$\frac{d\mathrm{T_I}\substack{\bullet\bullet\\\circ\circ\\\bullet\circ}}{dt} = \underset{\mathrm{ATP_{MI}}}{\nu_f}\left(\mathrm{T_M}\substack{\bullet\bullet\\\circ\circ\\\bullet\circ}\right) + \underset{\mathrm{AK_I}}{\nu_r}\left(\mathrm{D_I}\substack{\bullet\\\circ\\\bullet}\mathrm{D_I}\substack{\bullet\\\circ\\\circ}\right) + \underset{\mathrm{ATP_{IO}}}{\nu_r}\left(\mathrm{T_O}\substack{\bullet\bullet\\\circ\circ\\\bullet\circ}\right) + \underset{\mathrm{CK_I}}{\nu_r}\left(\mathrm{D_I}\substack{\bullet\\\circ\\\bullet}\mathrm{C_I}\substack{\bullet\\\circ\\\circ}\right) - \underset{\mathrm{AK_I}}{\nu_f}\left(\mathrm{T_I}\substack{\bullet\bullet\\\circ\circ\\\bullet\circ}\right) - \underset{\mathrm{ATP_{IO}}}{\nu_f}\left(\mathrm{T_I}\substack{\bullet\bullet\\\circ\circ\\\bullet\circ}\right) - \underset{\mathrm{CK_I}}{\nu_f}\left(\mathrm{T_I}\substack{\bullet\bullet\\\circ\circ\\\bullet\circ}\right) - \underset{\mathrm{ATP_{MI}}}{\nu_r}\left(\mathrm{T_I}\substack{\bullet\bullet\\\circ\circ\\\bullet\circ}\right)$$

$$\frac{d\mathrm{T_I}\substack{\bullet\bullet\\\circ\circ\\\bullet\bullet}}{dt} = \underset{\mathrm{ATP_{MI}}}{\nu_f}\left(\mathrm{T_M}\substack{\bullet\bullet\\\circ\circ\\\bullet\bullet}\right) + \underset{\mathrm{AK_I}}{\nu_r}\left(\mathrm{D_I}\substack{\bullet\\\circ\\\bullet}\mathrm{D_I}\substack{\bullet\\\circ\\\bullet}\right) + \underset{\mathrm{ATP_{IO}}}{\nu_r}\left(\mathrm{T_O}\substack{\bullet\bullet\\\circ\circ\\\bullet\bullet}\right) + \underset{\mathrm{CK_I}}{\nu_r}\left(\mathrm{D_I}\substack{\bullet\\\circ\\\bullet}\mathrm{C_I}\substack{\bullet\\\circ\\\bullet}\right) - \underset{\mathrm{AK_I}}{\nu_f}\left(\mathrm{T_I}\substack{\bullet\bullet\\\circ\circ\\\bullet\bullet}\right) - \underset{\mathrm{ATP_{IO}}}{\nu_f}\left(\mathrm{T_I}\substack{\bullet\bullet\\\circ\circ\\\bullet\bullet}\right) - \underset{\mathrm{CK_I}}{\nu_f}\left(\mathrm{T_I}\substack{\bullet\bullet\\\circ\circ\\\bullet\bullet}\right) - \underset{\mathrm{ATP_{MI}}}{\nu_r}\left(\mathrm{T_I}\substack{\bullet\bullet\\\circ\circ\\\bullet\bullet}\right)$$

$$\frac{d\mathrm{T_I}\substack{\bullet\bullet\\\circ\bullet\\\bullet\circ}}{dt} = \underset{\mathrm{ATP_{MI}}}{\nu_f}\left(\mathrm{T_M}\substack{\bullet\bullet\\\circ\bullet\\\bullet\circ}\right) + \underset{\mathrm{AK_I}}{\nu_r}\left(\mathrm{D_I}\substack{\bullet\\\bullet\\\circ}\mathrm{D_I}\substack{\bullet\\\circ\\\bullet}\right) + \underset{\mathrm{ATP_{IO}}}{\nu_r}\left(\mathrm{T_O}\substack{\bullet\bullet\\\circ\bullet\\\bullet\circ}\right) + \underset{\mathrm{CK_I}}{\nu_r}\left(\mathrm{D_I}\substack{\bullet\\\circ\\\bullet}\mathrm{C_I}\substack{\bullet\\\bullet\\\circ}\right) - \underset{\mathrm{AK_I}}{\nu_f}\left(\mathrm{T_I}\substack{\bullet\bullet\\\circ\bullet\\\bullet\circ}\right) - \underset{\mathrm{ATP_{IO}}}{\nu_f}\left(\mathrm{T_I}\substack{\bullet\bullet\\\circ\bullet\\\bullet\circ}\right) - \underset{\mathrm{CK_I}}{\nu_f}\left(\mathrm{T_I}\substack{\bullet\bullet\\\circ\bullet\\\bullet\circ}\right) - \underset{\mathrm{ATP_{MI}}}{\nu_r}\left(\mathrm{T_I}\substack{\bullet\bullet\\\circ\bullet\\\bullet\circ}\right)$$

$$\frac{d\mathrm{T_I}\substack{\bullet\bullet\\\circ\bullet\\\bullet\bullet}}{dt} = \underset{\mathrm{ATP_{MI}}}{\nu_f}\left(\mathrm{T_M}\substack{\bullet\bullet\\\circ\bullet\\\bullet\bullet}\right) + \underset{\mathrm{AK_I}}{\nu_r}\left(\mathrm{D_I}\substack{\bullet\\\bullet\\\bullet}\mathrm{D_I}\substack{\bullet\\\circ\\\bullet}\right) + \underset{\mathrm{ATP_{IO}}}{\nu_r}\left(\mathrm{T_O}\substack{\bullet\bullet\\\circ\bullet\\\bullet\bullet}\right) + \underset{\mathrm{CK_I}}{\nu_r}\left(\mathrm{D_I}\substack{\bullet\\\circ\\\bullet}\mathrm{C_I}\substack{\bullet\\\bullet\\\bullet}\right) - \underset{\mathrm{AK_I}}{\nu_f}\left(\mathrm{T_I}\substack{\bullet\bullet\\\circ\bullet\\\bullet\bullet}\right) - \underset{\mathrm{ATP_{IO}}}{\nu_f}\left(\mathrm{T_I}\substack{\bullet\bullet\\\circ\bullet\\\bullet\bullet}\right) - \underset{\mathrm{CK_I}}{\nu_f}\left(\mathrm{T_I}\substack{\bullet\bullet\\\circ\bullet\\\bullet\bullet}\right) - \underset{\mathrm{ATP_{MI}}}{\nu_r}\left(\mathrm{T_I}\substack{\bullet\bullet\\\circ\bullet\\\bullet\bullet}\right)$$

$$\frac{d\mathrm{T_I}\substack{\bullet\circ\\\bullet\circ\\\circ\circ}}{dt} = \underset{\mathrm{ATP_{MI}}}{\nu_f}\left(\mathrm{T_M}\substack{\bullet\circ\\\bullet\circ\\\circ\circ}\right) + \underset{\mathrm{AK_I}}{\nu_r}\left(\mathrm{D_I}\substack{\bullet\\\bullet\\\circ}\mathrm{D_I}\substack{\circ\\\circ\\\circ}\right) + \underset{\mathrm{ATP_{IO}}}{\nu_r}\left(\mathrm{T_O}\substack{\bullet\circ\\\bullet\circ\\\circ\circ}\right) + \underset{\mathrm{CK_I}}{\nu_r}\left(\mathrm{D_I}\substack{\bullet\\\bullet\\\circ}\mathrm{C_I}\substack{\circ\\\circ\\\circ}\right) - \underset{\mathrm{AK_I}}{\nu_f}\left(\mathrm{T_I}\substack{\bullet\circ\\\bullet\circ\\\circ\circ}\right) - \underset{\mathrm{ATP_{IO}}}{\nu_f}\left(\mathrm{T_I}\substack{\bullet\circ\\\bullet\circ\\\circ\circ}\right) - \underset{\mathrm{CK_I}}{\nu_f}\left(\mathrm{T_I}\substack{\bullet\circ\\\bullet\circ\\\circ\circ}\right) - \underset{\mathrm{ATP_{MI}}}{\nu_r}\left(\mathrm{T_I}\substack{\bullet\circ\\\bullet\circ\\\circ\circ}\right)$$

$$\frac{d\mathrm{T_I}\substack{\bullet\circ\\\bullet\circ\\\circ\bullet}}{dt} = \underset{\mathrm{ATP_{MI}}}{\nu_f}\left(\mathrm{T_M}\substack{\bullet\circ\\\bullet\circ\\\circ\bullet}\right) + \underset{\mathrm{AK_I}}{\nu_r}\left(\mathrm{D_I}\substack{\bullet\\\bullet\\\circ}\mathrm{D_I}\substack{\circ\\\circ\\\bullet}\right) + \underset{\mathrm{ATP_{IO}}}{\nu_r}\left(\mathrm{T_O}\substack{\bullet\circ\\\bullet\circ\\\circ\bullet}\right) + \underset{\mathrm{CK_I}}{\nu_r}\left(\mathrm{D_I}\substack{\bullet\\\bullet\\\circ}\mathrm{C_I}\substack{\circ\\\circ\\\bullet}\right) - \underset{\mathrm{AK_I}}{\nu_f}\left(\mathrm{T_I}\substack{\bullet\circ\\\bullet\circ\\\circ\bullet}\right) - \underset{\mathrm{ATP_{IO}}}{\nu_f}\left(\mathrm{T_I}\substack{\bullet\circ\\\bullet\circ\\\circ\bullet}\right) - \underset{\mathrm{CK_I}}{\nu_f}\left(\mathrm{T_I}\substack{\bullet\circ\\\bullet\circ\\\circ\bullet}\right) - \underset{\mathrm{ATP_{MI}}}{\nu_r}\left(\mathrm{T_I}\substack{\bullet\circ\\\bullet\circ\\\circ\bullet}\right)$$

$$\frac{d\mathrm{T_I}\substack{\bullet\circ\\\bullet\bullet\\\circ\circ}}{dt} = \underset{\mathrm{ATP_{MI}}}{\nu_f}\left(\mathrm{T_M}\substack{\bullet\circ\\\bullet\bullet\\\circ\circ}\right) + \underset{\mathrm{AK_I}}{\nu_r}\left(\mathrm{D_I}\substack{\bullet\\\bullet\\\circ}\mathrm{D_I}\substack{\circ\\\bullet\\\circ}\right) + \underset{\mathrm{ATP_{IO}}}{\nu_r}\left(\mathrm{T_O}\substack{\bullet\circ\\\bullet\bullet\\\circ\circ}\right) + \underset{\mathrm{CK_I}}{\nu_r}\left(\mathrm{D_I}\substack{\bullet\\\bullet\\\circ}\mathrm{C_I}\substack{\circ\\\bullet\\\circ}\right) - \underset{\mathrm{AK_I}}{\nu_f}\left(\mathrm{T_I}\substack{\bullet\circ\\\bullet\bullet\\\circ\circ}\right) - \underset{\mathrm{ATP_{IO}}}{\nu_f}\left(\mathrm{T_I}\substack{\bullet\circ\\\bullet\bullet\\\circ\circ}\right) - \underset{\mathrm{CK_I}}{\nu_f}\left(\mathrm{T_I}\substack{\bullet\circ\\\bullet\bullet\\\circ\circ}\right) - \underset{\mathrm{ATP_{MI}}}{\nu_r}\left(\mathrm{T_I}\substack{\bullet\circ\\\bullet\bullet\\\circ\circ}\right)$$

$$\frac{d\mathrm{T_I}\substack{\bullet\circ\\\bullet\bullet\\\circ\bullet}}{dt} = \underset{\mathrm{ATP_{MI}}}{\nu_f}\left(\mathrm{T_M}\substack{\bullet\circ\\\bullet\bullet\\\circ\bullet}\right) + \underset{\mathrm{AK_I}}{\nu_r}\left(\mathrm{D_I}\substack{\bullet\\\bullet\\\circ}\mathrm{D_I}\substack{\circ\\\bullet\\\bullet}\right) + \underset{\mathrm{ATP_{IO}}}{\nu_r}\left(\mathrm{T_O}\substack{\bullet\circ\\\bullet\bullet\\\circ\bullet}\right) + \underset{\mathrm{CK_I}}{\nu_r}\left(\mathrm{D_I}\substack{\bullet\\\bullet\\\circ}\mathrm{C_I}\substack{\circ\\\bullet\\\bullet}\right) - \underset{\mathrm{AK_I}}{\nu_f}\left(\mathrm{T_I}\substack{\bullet\circ\\\bullet\bullet\\\circ\bullet}\right) - \underset{\mathrm{ATP_{IO}}}{\nu_f}\left(\mathrm{T_I}\substack{\bullet\circ\\\bullet\bullet\\\circ\bullet}\right) - \underset{\mathrm{CK_I}}{\nu_f}\left(\mathrm{T_I}\substack{\bullet\circ\\\bullet\bullet\\\circ\bullet}\right) - \underset{\mathrm{ATP_{MI}}}{\nu_r}\left(\mathrm{T_I}\substack{\bullet\circ\\\bullet\bullet\\\circ\bullet}\right)$$

$$\frac{d\mathrm{T_I}\substack{\bullet\bullet\\\bullet\circ\\\circ\circ}}{dt} = \underset{\mathrm{ATP_{MI}}}{\nu_f}\left(\mathrm{T_M}\substack{\bullet\bullet\\\bullet\circ\\\circ\circ}\right) + \underset{\mathrm{AK_I}}{\nu_r}\left(\mathrm{D_I}\substack{\bullet\\\bullet\\\circ}\mathrm{D_I}\substack{\bullet\\\circ\\\circ}\right) + \underset{\mathrm{ATP_{IO}}}{\nu_r}\left(\mathrm{T_O}\substack{\bullet\bullet\\\bullet\circ\\\circ\circ}\right) + \underset{\mathrm{CK_I}}{\nu_r}\left(\mathrm{D_I}\substack{\bullet\\\bullet\\\circ}\mathrm{C_I}\substack{\bullet\\\circ\\\circ}\right) - \underset{\mathrm{AK_I}}{\nu_f}\left(\mathrm{T_I}\substack{\bullet\bullet\\\bullet\circ\\\circ\circ}\right) - \underset{\mathrm{ATP_{IO}}}{\nu_f}\left(\mathrm{T_I}\substack{\bullet\bullet\\\bullet\circ\\\circ\circ}\right) - \underset{\mathrm{CK_I}}{\nu_f}\left(\mathrm{T_I}\substack{\bullet\bullet\\\bullet\circ\\\circ\circ}\right) - \underset{\mathrm{ATP_{MI}}}{\nu_r}\left(\mathrm{T_I}\substack{\bullet\bullet\\\bullet\circ\\\circ\circ}\right)$$

$$\frac{d\mathrm{T_I}\substack{\bullet\bullet\\\bullet\circ\\\circ\bullet}}{dt} = \underset{\mathrm{ATP_{MI}}}{\nu_f}\left(\mathrm{T_M}\substack{\bullet\bullet\\\bullet\circ\\\circ\bullet}\right) + \underset{\mathrm{AK_I}}{\nu_r}\left(\mathrm{D_I}\substack{\bullet\\\bullet\\\circ}\mathrm{D_I}\substack{\bullet\\\circ\\\bullet}\right) + \underset{\mathrm{ATP_{IO}}}{\nu_r}\left(\mathrm{T_O}\substack{\bullet\bullet\\\bullet\circ\\\circ\bullet}\right) + \underset{\mathrm{CK_I}}{\nu_r}\left(\mathrm{D_I}\substack{\bullet\\\bullet\\\circ}\mathrm{C_I}\substack{\bullet\\\circ\\\bullet}\right) - \underset{\mathrm{AK_I}}{\nu_f}\left(\mathrm{T_I}\substack{\bullet\bullet\\\bullet\circ\\\circ\bullet}\right) - \underset{\mathrm{ATP_{IO}}}{\nu_f}\left(\mathrm{T_I}\substack{\bullet\bullet\\\bullet\circ\\\circ\bullet}\right) - \underset{\mathrm{CK_I}}{\nu_f}\left(\mathrm{T_I}\substack{\bullet\bullet\\\bullet\circ\\\circ\bullet}\right) - \underset{\mathrm{ATP_{MI}}}{\nu_r}\left(\mathrm{T_I}\substack{\bullet\bullet\\\bullet\circ\\\circ\bullet}\right)$$

$$\frac{d\mathrm{T_I}\substack{\bullet\bullet\\\bullet\bullet\\\circ\circ}}{dt} = \underset{\mathrm{ATP_{MI}}}{\nu_f}\left(\mathrm{T_M}\substack{\bullet\bullet\\\bullet\bullet\\\circ\circ}\right) + \underset{\mathrm{AK_I}}{\nu_r}\left(\mathrm{D_I}\substack{\bullet\\\bullet\\\circ}\mathrm{D_I}\substack{\bullet\\\bullet\\\circ}\right) + \underset{\mathrm{ATP_{IO}}}{\nu_r}\left(\mathrm{T_O}\substack{\bullet\bullet\\\bullet\bullet\\\circ\circ}\right) + \underset{\mathrm{CK_I}}{\nu_r}\left(\mathrm{D_I}\substack{\bullet\\\bullet\\\circ}\mathrm{C_I}\substack{\bullet\\\bullet\\\circ}\right) - \underset{\mathrm{AK_I}}{\nu_f}\left(\mathrm{T_I}\substack{\bullet\bullet\\\bullet\bullet\\\circ\circ}\right) - \underset{\mathrm{ATP_{IO}}}{\nu_f}\left(\mathrm{T_I}\substack{\bullet\bullet\\\bullet\bullet\\\circ\circ}\right) - \underset{\mathrm{CK_I}}{\nu_f}\left(\mathrm{T_I}\substack{\bullet\bullet\\\bullet\bullet\\\circ\circ}\right) - \underset{\mathrm{ATP_{MI}}}{\nu_r}\left(\mathrm{T_I}\substack{\bullet\bullet\\\bullet\bullet\\\circ\circ}\right)$$

$$\frac{d\mathrm{T_I}\substack{\bullet\bullet\\\bullet\bullet\\\circ\bullet}}{dt} = \underset{\mathrm{ATP_{MI}}}{\nu_f}\left(\mathrm{T_M}\substack{\bullet\bullet\\\bullet\bullet\\\circ\bullet}\right) + \underset{\mathrm{AK_I}}{\nu_r}\left(\mathrm{D_I}\substack{\bullet\\\bullet\\\bullet}\mathrm{D_I}\substack{\bullet\\\bullet\\\circ}\right) + \underset{\mathrm{ATP_{IO}}}{\nu_r}\left(\mathrm{T_O}\substack{\bullet\bullet\\\bullet\bullet\\\circ\bullet}\right) + \underset{\mathrm{CK_I}}{\nu_r}\left(\mathrm{D_I}\substack{\bullet\\\bullet\\\circ}\mathrm{C_I}\substack{\bullet\\\bullet\\\bullet}\right) - \underset{\mathrm{AK_I}}{\nu_f}\left(\mathrm{T_I}\substack{\bullet\bullet\\\bullet\bullet\\\circ\bullet}\right) - \underset{\mathrm{ATP_{IO}}}{\nu_f}\left(\mathrm{T_I}\substack{\bullet\bullet\\\bullet\bullet\\\circ\bullet}\right) - \underset{\mathrm{CK_I}}{\nu_f}\left(\mathrm{T_I}\substack{\bullet\bullet\\\bullet\bullet\\\circ\bullet}\right) - \underset{\mathrm{ATP_{MI}}}{\nu_r}\left(\mathrm{T_I}\substack{\bullet\bullet\\\bullet\bullet\\\circ\bullet}\right)$$

$$\frac{d\mathrm{T_I}\substack{\bullet\circ\\\bullet\circ\\\bullet\circ}}{dt} = \underset{\mathrm{ATP_{MI}}}{\nu_f}\left(\mathrm{T_M}\substack{\bullet\circ\\\bullet\circ\\\bullet\circ}\right) + \underset{\mathrm{AK_I}}{\nu_r}\left(\mathrm{D_I}\substack{\bullet\\\bullet\\\bullet}\mathrm{D_I}\substack{\circ\\\circ\\\circ}\right) + \underset{\mathrm{ATP_{IO}}}{\nu_r}\left(\mathrm{T_O}\substack{\bullet\circ\\\bullet\circ\\\bullet\circ}\right) + \underset{\mathrm{CK_I}}{\nu_r}\left(\mathrm{D_I}\substack{\bullet\\\bullet\\\bullet}\mathrm{C_I}\substack{\circ\\\circ\\\circ}\right) - \underset{\mathrm{AK_I}}{\nu_f}\left(\mathrm{T_I}\substack{\bullet\circ\\\bullet\circ\\\bullet\circ}\right) - \underset{\mathrm{ATP_{IO}}}{\nu_f}\left(\mathrm{T_I}\substack{\bullet\circ\\\bullet\circ\\\bullet\circ}\right) - \underset{\mathrm{CK_I}}{\nu_f}\left(\mathrm{T_I}\substack{\bullet\circ\\\bullet\circ\\\bullet\circ}\right) - \underset{\mathrm{ATP_{MI}}}{\nu_r}\left(\mathrm{T_I}\substack{\bullet\circ\\\bullet\circ\\\bullet\circ}\right)$$

$$\frac{d\mathrm{T_I}\substack{\bullet\circ\\\bullet\circ\\\bullet\bullet}}{dt} = \underset{\mathrm{ATP_{MI}}}{\nu_f}\left(\mathrm{T_M}\substack{\bullet\circ\\\bullet\circ\\\bullet\bullet}\right) + \underset{\mathrm{AK_I}}{\nu_r}\left(\mathrm{D_I}\substack{\bullet\\\bullet\\\bullet}\mathrm{D_I}\substack{\circ\\\circ\\\bullet}\right) + \underset{\mathrm{ATP_{IO}}}{\nu_r}\left(\mathrm{T_O}\substack{\bullet\circ\\\bullet\circ\\\bullet\bullet}\right) + \underset{\mathrm{CK_I}}{\nu_r}\left(\mathrm{D_I}\substack{\bullet\\\bullet\\\bullet}\mathrm{C_I}\substack{\circ\\\circ\\\bullet}\right) - \underset{\mathrm{AK_I}}{\nu_f}\left(\mathrm{T_I}\substack{\bullet\circ\\\bullet\circ\\\bullet\bullet}\right) - \underset{\mathrm{ATP_{IO}}}{\nu_f}\left(\mathrm{T_I}\substack{\bullet\circ\\\bullet\circ\\\bullet\bullet}\right) - \underset{\mathrm{CK_I}}{\nu_f}\left(\mathrm{T_I}\substack{\bullet\circ\\\bullet\circ\\\bullet\bullet}\right) - \underset{\mathrm{ATP_{MI}}}{\nu_r}\left(\mathrm{T_I}\substack{\bullet\circ\\\bullet\circ\\\bullet\bullet}\right)$$

$$\frac{d\mathrm{T_I}\substack{\bullet\circ\\\bullet\bullet\\\bullet\circ}}{dt} = \underset{\mathrm{ATP_{MI}}}{\nu_f}\left(\mathrm{T_M}\substack{\bullet\circ\\\bullet\bullet\\\bullet\circ}\right) + \underset{\mathrm{AK_I}}{\nu_r}\left(\mathrm{D_I}\substack{\bullet\\\bullet\\\bullet}\mathrm{D_I}\substack{\circ\\\bullet\\\circ}\right) + \underset{\mathrm{ATP_{IO}}}{\nu_r}\left(\mathrm{T_O}\substack{\bullet\circ\\\bullet\bullet\\\bullet\circ}\right) + \underset{\mathrm{CK_I}}{\nu_r}\left(\mathrm{D_I}\substack{\bullet\\\bullet\\\bullet}\mathrm{C_I}\substack{\circ\\\bullet\\\circ}\right) - \underset{\mathrm{AK_I}}{\nu_f}\left(\mathrm{T_I}\substack{\bullet\circ\\\bullet\bullet\\\bullet\circ}\right) - \underset{\mathrm{ATP_{IO}}}{\nu_f}\left(\mathrm{T_I}\substack{\bullet\circ\\\bullet\bullet\\\bullet\circ}\right) - \underset{\mathrm{CK_I}}{\nu_f}\left(\mathrm{T_I}\substack{\bullet\circ\\\bullet\bullet\\\bullet\circ}\right) - \underset{\mathrm{ATP_{MI}}}{\nu_r}\left(\mathrm{T_I}\substack{\bullet\circ\\\bullet\bullet\\\bullet\circ}\right)$$

$$\frac{d\mathrm{T_I}\substack{\bullet\circ\\\bullet\bullet\\\bullet\bullet}}{dt} = \underset{\mathrm{ATP_{MI}}}{\nu_f}\left(\mathrm{T_M}\substack{\bullet\circ\\\bullet\bullet\\\bullet\bullet}\right) + \underset{\mathrm{AK_I}}{\nu_r}\left(\mathrm{D_I}\substack{\bullet\\\bullet\\\bullet}\mathrm{D_I}\substack{\circ\\\bullet\\\bullet}\right) + \underset{\mathrm{ATP_{IO}}}{\nu_r}\left(\mathrm{T_O}\substack{\bullet\circ\\\bullet\bullet\\\bullet\bullet}\right) + \underset{\mathrm{CK_I}}{\nu_r}\left(\mathrm{D_I}\substack{\bullet\\\bullet\\\bullet}\mathrm{C_I}\substack{\circ\\\bullet\\\bullet}\right) - \underset{\mathrm{AK_I}}{\nu_f}\left(\mathrm{T_I}\substack{\bullet\circ\\\bullet\bullet\\\bullet\bullet}\right) - \underset{\mathrm{ATP_{IO}}}{\nu_f}\left(\mathrm{T_I}\substack{\bullet\circ\\\bullet\bullet\\\bullet\bullet}\right) - \underset{\mathrm{CK_I}}{\nu_f}\left(\mathrm{T_I}\substack{\bullet\circ\\\bullet\bullet\\\bullet\bullet}\right) - \underset{\mathrm{ATP_{MI}}}{\nu_r}\left(\mathrm{T_I}\substack{\bullet\circ\\\bullet\bullet\\\bullet\bullet}\right)$$

$$\frac{d\mathrm{T_I}\substack{\bullet\bullet\\\bullet\circ\\\bullet\circ}}{dt} = \underset{\mathrm{ATP_{MI}}}{\nu_f}\left(\mathrm{T_M}\substack{\bullet\bullet\\\bullet\circ\\\bullet\circ}\right) + \underset{\mathrm{AK_I}}{\nu_r}\left(\mathrm{D_I}\substack{\bullet\\\bullet\\\bullet}\mathrm{D_I}\substack{\bullet\\\circ\\\circ}\right) + \underset{\mathrm{ATP_{IO}}}{\nu_r}\left(\mathrm{T_O}\substack{\bullet\bullet\\\bullet\circ\\\bullet\circ}\right) + \underset{\mathrm{CK_I}}{\nu_r}\left(\mathrm{D_I}\substack{\bullet\\\bullet\\\bullet}\mathrm{C_I}\substack{\bullet\\\circ\\\circ}\right) - \underset{\mathrm{AK_I}}{\nu_f}\left(\mathrm{T_I}\substack{\bullet\bullet\\\bullet\circ\\\bullet\circ}\right) - \underset{\mathrm{ATP_{IO}}}{\nu_f}\left(\mathrm{T_I}\substack{\bullet\bullet\\\bullet\circ\\\bullet\circ}\right) - \underset{\mathrm{CK_I}}{\nu_f}\left(\mathrm{T_I}\substack{\bullet\bullet\\\bullet\circ\\\bullet\circ}\right) - \underset{\mathrm{ATP_{MI}}}{\nu_r}\left(\mathrm{T_I}\substack{\bullet\bullet\\\bullet\circ\\\bullet\circ}\right)$$

$$\frac{d\mathrm{T_I}\substack{\bullet\bullet\\\bullet\circ\\\bullet\bullet}}{dt} = \underset{\mathrm{ATP_{MI}}}{\nu_f}\left(\mathrm{T_M}\substack{\bullet\bullet\\\bullet\circ\\\bullet\bullet}\right) + \underset{\mathrm{AK_I}}{\nu_r}\left(\mathrm{D_I}\substack{\bullet\\\bullet\\\bullet}\mathrm{D_I}\substack{\bullet\\\circ\\\bullet}\right) + \underset{\mathrm{ATP_{IO}}}{\nu_r}\left(\mathrm{T_O}\substack{\bullet\bullet\\\bullet\circ\\\bullet\bullet}\right) + \underset{\mathrm{CK_I}}{\nu_r}\left(\mathrm{D_I}\substack{\bullet\\\bullet\\\bullet}\mathrm{C_I}\substack{\bullet\\\circ\\\bullet}\right) - \underset{\mathrm{AK_I}}{\nu_f}\left(\mathrm{T_I}\substack{\bullet\bullet\\\bullet\circ\\\bullet\bullet}\right) - \underset{\mathrm{ATP_{IO}}}{\nu_f}\left(\mathrm{T_I}\substack{\bullet\bullet\\\bullet\circ\\\bullet\bullet}\right) - \underset{\mathrm{CK_I}}{\nu_f}\left(\mathrm{T_I}\substack{\bullet\bullet\\\bullet\circ\\\bullet\bullet}\right) - \underset{\mathrm{ATP_{MI}}}{\nu_r}\left(\mathrm{T_I}\substack{\bullet\bullet\\\bullet\circ\\\bullet\bullet}\right)$$

$$\frac{d\mathrm{T_I}\substack{\bullet\bullet\\\bullet\bullet\\\bullet\circ}}{dt} = \underset{\mathrm{ATP_{MI}}}{\nu_f}\left(\mathrm{T_M}\substack{\bullet\bullet\\\bullet\bullet\\\bullet\circ}\right) + \underset{\mathrm{AK_I}}{\nu_r}\left(\mathrm{D_I}\substack{\bullet\\\bullet\\\bullet}\mathrm{D_I}\substack{\bullet\\\bullet\\\circ}\right) + \underset{\mathrm{ATP_{IO}}}{\nu_r}\left(\mathrm{T_O}\substack{\bullet\bullet\\\bullet\bullet\\\bullet\circ}\right) + \underset{\mathrm{CK_I}}{\nu_r}\left(\mathrm{D_I}\substack{\bullet\\\bullet\\\bullet}\mathrm{C_I}\substack{\bullet\\\bullet\\\circ}\right) - \underset{\mathrm{AK_I}}{\nu_f}\left(\mathrm{T_I}\substack{\bullet\bullet\\\bullet\bullet\\\bullet\circ}\right) - \underset{\mathrm{ATP_{IO}}}{\nu_f}\left(\mathrm{T_I}\substack{\bullet\bullet\\\bullet\bullet\\\bullet\circ}\right) - \underset{\mathrm{CK_I}}{\nu_f}\left(\mathrm{T_I}\substack{\bullet\bullet\\\bullet\bullet\\\bullet\circ}\right) - \underset{\mathrm{ATP_{MI}}}{\nu_r}\left(\mathrm{T_I}\substack{\bullet\bullet\\\bullet\bullet\\\bullet\circ}\right)$$

$$\frac{d\mathrm{T}_{\mathrm{I}}^{\substack{\bullet\bullet\\\bullet\bullet\\\bullet\bullet}}}{dt} = \underset{\mathrm{ATPMI}}{\nu_f}\left(\mathrm{T}_{\mathrm{M}}^{\substack{\bullet\bullet\\\bullet\bullet\\\bullet\bullet}}\right) + \underset{\mathrm{AKI}}{\nu_r}\left(\mathrm{D}_{\mathrm{I}}^{\substack{\bullet\\\bullet\\\bullet}}\mathrm{D}_{\mathrm{I}}^{\substack{\bullet\\\bullet\\\bullet}}\right) + \underset{\mathrm{ATPIO}}{\nu_r}\left(\mathrm{T}_{\mathrm{O}}^{\substack{\bullet\bullet\\\bullet\bullet\\\bullet\bullet}}\right) + \underset{\mathrm{CKI}}{\nu_r}\left(\mathrm{D}_{\mathrm{I}}^{\substack{\bullet\\\bullet\\\bullet}}\mathrm{C}_{\mathrm{I}}^{\substack{\bullet\\\bullet\\\bullet}}\right) - \underset{\mathrm{AKI}}{\nu_f}\left(\mathrm{T}_{\mathrm{I}}^{\substack{\bullet\bullet\\\bullet\bullet\\\bullet\bullet}}\right) - \underset{\mathrm{ATPIO}}{\nu_f}\left(\mathrm{T}_{\mathrm{I}}^{\substack{\bullet\bullet\\\bullet\bullet\\\bullet\bullet}}\right) - \underset{\mathrm{CKI}}{\nu_f}\left(\mathrm{T}_{\mathrm{I}}^{\substack{\bullet\bullet\\\bullet\bullet\\\bullet\bullet}}\right) - \underset{\mathrm{ATPMI}}{\nu_r}\left(\mathrm{T}_{\mathrm{I}}^{\substack{\bullet\bullet\\\bullet\bullet\\\bullet\bullet}}\right)$$

$$\frac{d\mathrm{T}_{\mathrm{M}}^{\substack{\circ\circ\\\circ\circ\\\circ\circ}}}{dt} = \underset{\mathrm{ATPSM}}{\nu_f}\left(\mathrm{T}_{\mathrm{S}}^{\substack{\circ\circ\\\circ\circ\\\circ\circ}}\right) + \underset{\mathrm{ATPMI}}{\nu_r}\left(\mathrm{T}_{\mathrm{I}}^{\substack{\circ\circ\\\circ\circ\\\circ\circ}}\right) - \underset{\mathrm{ATPMI}}{\nu_f}\left(\mathrm{T}_{\mathrm{M}}^{\substack{\circ\circ\\\circ\circ\\\circ\circ}}\right)$$

$$\frac{d\mathrm{T}_{\mathrm{M}}^{\substack{\circ\circ\\\circ\circ\\\circ\bullet}}}{dt} = \underset{\mathrm{ATPSM}}{\nu_f}\left(\mathrm{T}_{\mathrm{S}}^{\substack{\circ\circ\\\circ\circ\\\circ\bullet}}\right) + \underset{\mathrm{ATPMI}}{\nu_r}\left(\mathrm{T}_{\mathrm{I}}^{\substack{\circ\circ\\\circ\circ\\\circ\bullet}}\right) - \underset{\mathrm{ATPMI}}{\nu_f}\left(\mathrm{T}_{\mathrm{M}}^{\substack{\circ\circ\\\circ\circ\\\circ\bullet}}\right)$$

$$\frac{d\mathrm{T}_{\mathrm{M}}^{\substack{\circ\circ\\\circ\bullet\\\circ\circ}}}{dt} = \underset{\mathrm{ATPSM}}{\nu_f}\left(\mathrm{T}_{\mathrm{S}}^{\substack{\circ\circ\\\circ\bullet\\\circ\circ}}\right) + \underset{\mathrm{ATPMI}}{\nu_r}\left(\mathrm{T}_{\mathrm{I}}^{\substack{\circ\circ\\\circ\bullet\\\circ\circ}}\right) - \underset{\mathrm{ATPMI}}{\nu_f}\left(\mathrm{T}_{\mathrm{M}}^{\substack{\circ\circ\\\circ\bullet\\\circ\circ}}\right)$$

$$\frac{d\mathrm{T}_{\mathrm{M}}^{\substack{\circ\circ\\\circ\bullet\\\circ\bullet}}}{dt} = \underset{\mathrm{ATPSM}}{\nu_f}\left(\mathrm{T}_{\mathrm{S}}^{\substack{\circ\circ\\\circ\bullet\\\circ\bullet}}\right) + \underset{\mathrm{ATPMI}}{\nu_r}\left(\mathrm{T}_{\mathrm{I}}^{\substack{\circ\circ\\\circ\bullet\\\circ\bullet}}\right) - \underset{\mathrm{ATPMI}}{\nu_f}\left(\mathrm{T}_{\mathrm{M}}^{\substack{\circ\circ\\\circ\bullet\\\circ\bullet}}\right)$$

$$\frac{d\mathrm{T}_{\mathrm{M}}^{\substack{\circ\bullet\\\circ\circ\\\circ\circ}}}{dt} = \underset{\mathrm{ATPSM}}{\nu_f}\left(\mathrm{T}_{\mathrm{S}}^{\substack{\circ\bullet\\\circ\circ\\\circ\circ}}\right) + \underset{\mathrm{ATPMI}}{\nu_r}\left(\mathrm{T}_{\mathrm{I}}^{\substack{\circ\bullet\\\circ\circ\\\circ\circ}}\right) - \underset{\mathrm{ATPMI}}{\nu_f}\left(\mathrm{T}_{\mathrm{M}}^{\substack{\circ\bullet\\\circ\circ\\\circ\circ}}\right)$$

$$\frac{d\mathrm{T}_{\mathrm{M}}^{\substack{\circ\bullet\\\circ\circ\\\circ\bullet}}}{dt} = \underset{\mathrm{ATPSM}}{\nu_f}\left(\mathrm{T}_{\mathrm{S}}^{\substack{\circ\bullet\\\circ\circ\\\circ\bullet}}\right) + \underset{\mathrm{ATPMI}}{\nu_r}\left(\mathrm{T}_{\mathrm{I}}^{\substack{\circ\bullet\\\circ\circ\\\circ\bullet}}\right) - \underset{\mathrm{ATPMI}}{\nu_f}\left(\mathrm{T}_{\mathrm{M}}^{\substack{\circ\bullet\\\circ\circ\\\circ\bullet}}\right)$$

$$\frac{d\mathrm{T}_{\mathrm{M}}^{\substack{\circ\bullet\\\circ\bullet\\\circ\circ}}}{dt} = \underset{\mathrm{ATPSM}}{\nu_f}\left(\mathrm{T}_{\mathrm{S}}^{\substack{\circ\bullet\\\circ\bullet\\\circ\circ}}\right) + \underset{\mathrm{ATPMI}}{\nu_r}\left(\mathrm{T}_{\mathrm{I}}^{\substack{\circ\bullet\\\circ\bullet\\\circ\circ}}\right) - \underset{\mathrm{ATPMI}}{\nu_f}\left(\mathrm{T}_{\mathrm{M}}^{\substack{\circ\bullet\\\circ\bullet\\\circ\circ}}\right)$$

$$\frac{d\mathrm{T}_{\mathrm{M}}^{\substack{\circ\bullet\\\circ\bullet\\\circ\bullet}}}{dt} = \underset{\mathrm{ATPSM}}{\nu_f}\left(\mathrm{T}_{\mathrm{S}}^{\substack{\circ\bullet\\\circ\bullet\\\circ\bullet}}\right) + \underset{\mathrm{ATPMI}}{\nu_r}\left(\mathrm{T}_{\mathrm{I}}^{\substack{\circ\bullet\\\circ\bullet\\\circ\bullet}}\right) - \underset{\mathrm{ATPMI}}{\nu_f}\left(\mathrm{T}_{\mathrm{M}}^{\substack{\circ\bullet\\\circ\bullet\\\circ\bullet}}\right)$$

$$\frac{d\mathrm{T}_{\mathrm{M}}^{\substack{\circ\circ\\\circ\circ\\\bullet\circ}}}{dt} = \underset{\mathrm{ATPSM}}{\nu_f}\left(\mathrm{T}_{\mathrm{S}}^{\substack{\circ\circ\\\circ\circ\\\bullet\circ}}\right) + \underset{\mathrm{ATPMI}}{\nu_r}\left(\mathrm{T}_{\mathrm{I}}^{\substack{\circ\circ\\\circ\circ\\\bullet\circ}}\right) - \underset{\mathrm{ATPMI}}{\nu_f}\left(\mathrm{T}_{\mathrm{M}}^{\substack{\circ\circ\\\circ\circ\\\bullet\circ}}\right)$$

$$\frac{d\mathrm{T}_{\mathrm{M}}^{\substack{\circ\circ\\\circ\circ\\\bullet\bullet}}}{dt} = \underset{\mathrm{ATPSM}}{\nu_f}\left(\mathrm{T}_{\mathrm{S}}^{\substack{\circ\circ\\\circ\circ\\\bullet\bullet}}\right) + \underset{\mathrm{ATPMI}}{\nu_r}\left(\mathrm{T}_{\mathrm{I}}^{\substack{\circ\circ\\\circ\circ\\\bullet\bullet}}\right) - \underset{\mathrm{ATPMI}}{\nu_f}\left(\mathrm{T}_{\mathrm{M}}^{\substack{\circ\circ\\\circ\circ\\\bullet\bullet}}\right)$$

$$\frac{d\mathrm{T}_{\mathrm{M}}^{\substack{\circ\circ\\\circ\bullet\\\bullet\circ}}}{dt} = \underset{\mathrm{ATPSM}}{\nu_f}\left(\mathrm{T}_{\mathrm{S}}^{\substack{\circ\circ\\\circ\bullet\\\bullet\circ}}\right) + \underset{\mathrm{ATPMI}}{\nu_r}\left(\mathrm{T}_{\mathrm{I}}^{\substack{\circ\circ\\\circ\bullet\\\bullet\circ}}\right) - \underset{\mathrm{ATPMI}}{\nu_f}\left(\mathrm{T}_{\mathrm{M}}^{\substack{\circ\circ\\\circ\bullet\\\bullet\circ}}\right)$$

$$\frac{d\mathrm{T}_{\mathrm{M}}^{\substack{\circ\circ\\\circ\bullet\\\bullet\bullet}}}{dt} = \underset{\mathrm{ATPSM}}{\nu_f}\left(\mathrm{T}_{\mathrm{S}}^{\substack{\circ\circ\\\circ\bullet\\\bullet\bullet}}\right) + \underset{\mathrm{ATPMI}}{\nu_r}\left(\mathrm{T}_{\mathrm{I}}^{\substack{\circ\circ\\\circ\bullet\\\bullet\bullet}}\right) - \underset{\mathrm{ATPMI}}{\nu_f}\left(\mathrm{T}_{\mathrm{M}}^{\substack{\circ\circ\\\circ\bullet\\\bullet\bullet}}\right)$$

$$\frac{d\mathrm{T}_{\mathrm{M}}^{\substack{\circ\bullet\\\circ\circ\\\bullet\circ}}}{dt} = \underset{\mathrm{ATPSM}}{\nu_f}\left(\mathrm{T}_{\mathrm{S}}^{\substack{\circ\bullet\\\circ\circ\\\bullet\circ}}\right) + \underset{\mathrm{ATPMI}}{\nu_r}\left(\mathrm{T}_{\mathrm{I}}^{\substack{\circ\bullet\\\circ\circ\\\bullet\circ}}\right) - \underset{\mathrm{ATPMI}}{\nu_f}\left(\mathrm{T}_{\mathrm{M}}^{\substack{\circ\bullet\\\circ\circ\\\bullet\circ}}\right)$$

$$\frac{d\mathrm{T}_{\mathrm{M}}^{\substack{\circ\bullet\\\circ\circ\\\bullet\bullet}}}{dt} = \underset{\mathrm{ATPSM}}{\nu_f}\left(\mathrm{T}_{\mathrm{S}}^{\substack{\circ\bullet\\\circ\circ\\\bullet\bullet}}\right) + \underset{\mathrm{ATPMI}}{\nu_r}\left(\mathrm{T}_{\mathrm{I}}^{\substack{\circ\bullet\\\circ\circ\\\bullet\bullet}}\right) - \underset{\mathrm{ATPMI}}{\nu_f}\left(\mathrm{T}_{\mathrm{M}}^{\substack{\circ\bullet\\\circ\circ\\\bullet\bullet}}\right)$$

$$\frac{d\mathrm{T}_{\mathrm{M}}^{\substack{\circ\bullet\\\circ\bullet\\\bullet\circ}}}{dt} = \underset{\mathrm{ATPSM}}{\nu_f}\left(\mathrm{T}_{\mathrm{S}}^{\substack{\circ\bullet\\\circ\bullet\\\bullet\circ}}\right) + \underset{\mathrm{ATPMI}}{\nu_r}\left(\mathrm{T}_{\mathrm{I}}^{\substack{\circ\bullet\\\circ\bullet\\\bullet\circ}}\right) - \underset{\mathrm{ATPMI}}{\nu_f}\left(\mathrm{T}_{\mathrm{M}}^{\substack{\circ\bullet\\\circ\bullet\\\bullet\circ}}\right)$$

$$\frac{d\mathrm{T}_{\mathrm{M}}^{\substack{\circ\bullet\\\circ\bullet\\\bullet\bullet}}}{dt} = \underset{\mathrm{ATPSM}}{\nu_f}\left(\mathrm{T}_{\mathrm{S}}^{\substack{\circ\bullet\\\circ\bullet\\\bullet\bullet}}\right) + \underset{\mathrm{ATPMI}}{\nu_r}\left(\mathrm{T}_{\mathrm{I}}^{\substack{\circ\bullet\\\circ\bullet\\\bullet\bullet}}\right) - \underset{\mathrm{ATPMI}}{\nu_f}\left(\mathrm{T}_{\mathrm{M}}^{\substack{\circ\bullet\\\circ\bullet\\\bullet\bullet}}\right)$$

$$\frac{d\mathrm{T}_{\mathrm{M}}^{\substack{\circ\circ\\\bullet\circ\\\circ\circ}}}{dt} = \underset{\mathrm{ATPSM}}{\nu_f}\left(\mathrm{T}_{\mathrm{S}}^{\substack{\circ\circ\\\bullet\circ\\\circ\circ}}\right) + \underset{\mathrm{ATPMI}}{\nu_r}\left(\mathrm{T}_{\mathrm{I}}^{\substack{\circ\circ\\\bullet\circ\\\circ\circ}}\right) - \underset{\mathrm{ATPMI}}{\nu_f}\left(\mathrm{T}_{\mathrm{M}}^{\substack{\circ\circ\\\bullet\circ\\\circ\circ}}\right)$$

$$\frac{d\mathrm{T}_{\mathrm{M}}^{\substack{\circ\circ\\\bullet\circ\\\circ\bullet}}}{dt} = \underset{\mathrm{ATPSM}}{\nu_f}\left(\mathrm{T}_{\mathrm{S}}^{\substack{\circ\circ\\\bullet\circ\\\circ\bullet}}\right) + \underset{\mathrm{ATPMI}}{\nu_r}\left(\mathrm{T}_{\mathrm{I}}^{\substack{\circ\circ\\\bullet\circ\\\circ\bullet}}\right) - \underset{\mathrm{ATPMI}}{\nu_f}\left(\mathrm{T}_{\mathrm{M}}^{\substack{\circ\circ\\\bullet\circ\\\circ\bullet}}\right)$$

$$\frac{d\mathrm{T}_{\mathrm{M}}^{\substack{\circ\circ\\\bullet\bullet\\\circ\circ}}}{dt} = \underset{\mathrm{ATPSM}}{\nu_f}\left(\mathrm{T}_{\mathrm{S}}^{\substack{\circ\circ\\\bullet\bullet\\\circ\circ}}\right) + \underset{\mathrm{ATPMI}}{\nu_r}\left(\mathrm{T}_{\mathrm{I}}^{\substack{\circ\circ\\\bullet\bullet\\\circ\circ}}\right) - \underset{\mathrm{ATPMI}}{\nu_f}\left(\mathrm{T}_{\mathrm{M}}^{\substack{\circ\circ\\\bullet\bullet\\\circ\circ}}\right)$$

$$\frac{d\mathrm{T}_{\mathrm{M}}^{\substack{\circ\circ\\\bullet\bullet\\\circ\bullet}}}{dt} = \underset{\mathrm{ATPSM}}{\nu_f}\left(\mathrm{T}_{\mathrm{S}}^{\substack{\circ\circ\\\bullet\bullet\\\circ\bullet}}\right) + \underset{\mathrm{ATPMI}}{\nu_r}\left(\mathrm{T}_{\mathrm{I}}^{\substack{\circ\circ\\\bullet\bullet\\\circ\bullet}}\right) - \underset{\mathrm{ATPMI}}{\nu_f}\left(\mathrm{T}_{\mathrm{M}}^{\substack{\circ\circ\\\bullet\bullet\\\circ\bullet}}\right)$$

$$\frac{d\mathrm{T}_{\mathrm{M}\substack{\circ\bullet\\\bullet\circ\\\circ\circ}}}{dt} = \underset{\mathrm{ATPsm}}{\nu_f}\left(\mathrm{T}_{\mathrm{S}\substack{\circ\bullet\\\bullet\circ\\\circ\circ}}\right) + \underset{\mathrm{ATPmi}}{\nu_r}\left(\mathrm{T}_{\mathrm{I}\substack{\circ\bullet\\\bullet\circ\\\circ\circ}}\right) - \underset{\mathrm{ATPmi}}{\nu_f}\left(\mathrm{T}_{\mathrm{M}\substack{\circ\bullet\\\bullet\circ\\\circ\circ}}\right)$$

$$\frac{d\mathrm{T}_{\mathrm{M}\substack{\circ\bullet\\\bullet\circ\\\circ\bullet}}}{dt} = \underset{\mathrm{ATPsm}}{\nu_f}\left(\mathrm{T}_{\mathrm{S}\substack{\circ\bullet\\\bullet\circ\\\circ\bullet}}\right) + \underset{\mathrm{ATPmi}}{\nu_r}\left(\mathrm{T}_{\mathrm{I}\substack{\circ\bullet\\\bullet\circ\\\circ\bullet}}\right) - \underset{\mathrm{ATPmi}}{\nu_f}\left(\mathrm{T}_{\mathrm{M}\substack{\circ\bullet\\\bullet\circ\\\circ\bullet}}\right)$$

$$\frac{d\mathrm{T}_{\mathrm{M}\substack{\circ\bullet\\\bullet\bullet\\\circ\circ}}}{dt} = \underset{\mathrm{ATPsm}}{\nu_f}\left(\mathrm{T}_{\mathrm{S}\substack{\circ\bullet\\\bullet\bullet\\\circ\circ}}\right) + \underset{\mathrm{ATPmi}}{\nu_r}\left(\mathrm{T}_{\mathrm{I}\substack{\circ\bullet\\\bullet\bullet\\\circ\circ}}\right) - \underset{\mathrm{ATPmi}}{\nu_f}\left(\mathrm{T}_{\mathrm{M}\substack{\circ\bullet\\\bullet\bullet\\\circ\circ}}\right)$$

$$\frac{d\mathrm{T}_{\mathrm{M}\substack{\circ\bullet\\\bullet\bullet\\\circ\bullet}}}{dt} = \underset{\mathrm{ATPsm}}{\nu_f}\left(\mathrm{T}_{\mathrm{S}\substack{\circ\bullet\\\bullet\bullet\\\circ\bullet}}\right) + \underset{\mathrm{ATPmi}}{\nu_r}\left(\mathrm{T}_{\mathrm{I}\substack{\circ\bullet\\\bullet\bullet\\\circ\bullet}}\right) - \underset{\mathrm{ATPmi}}{\nu_f}\left(\mathrm{T}_{\mathrm{M}\substack{\circ\bullet\\\bullet\bullet\\\circ\bullet}}\right)$$

$$\frac{d\mathrm{T}_{\mathrm{M}\substack{\circ\circ\\\bullet\circ\\\bullet\circ}}}{dt} = \underset{\mathrm{ATPsm}}{\nu_f}\left(\mathrm{T}_{\mathrm{S}\substack{\circ\circ\\\bullet\circ\\\bullet\circ}}\right) + \underset{\mathrm{ATPmi}}{\nu_r}\left(\mathrm{T}_{\mathrm{I}\substack{\circ\circ\\\bullet\circ\\\bullet\circ}}\right) - \underset{\mathrm{ATPmi}}{\nu_f}\left(\mathrm{T}_{\mathrm{M}\substack{\circ\circ\\\bullet\circ\\\bullet\circ}}\right)$$

$$\frac{d\mathrm{T}_{\mathrm{M}\substack{\circ\circ\\\bullet\circ\\\bullet\bullet}}}{dt} = \underset{\mathrm{ATPsm}}{\nu_f}\left(\mathrm{T}_{\mathrm{S}\substack{\circ\circ\\\bullet\circ\\\bullet\bullet}}\right) + \underset{\mathrm{ATPmi}}{\nu_r}\left(\mathrm{T}_{\mathrm{I}\substack{\circ\circ\\\bullet\circ\\\bullet\bullet}}\right) - \underset{\mathrm{ATPmi}}{\nu_f}\left(\mathrm{T}_{\mathrm{M}\substack{\circ\circ\\\bullet\circ\\\bullet\bullet}}\right)$$

$$\frac{d\mathrm{T}_{\mathrm{M}\substack{\circ\circ\\\bullet\bullet\\\bullet\circ}}}{dt} = \underset{\mathrm{ATPsm}}{\nu_f}\left(\mathrm{T}_{\mathrm{S}\substack{\circ\circ\\\bullet\bullet\\\bullet\circ}}\right) + \underset{\mathrm{ATPmi}}{\nu_r}\left(\mathrm{T}_{\mathrm{I}\substack{\circ\circ\\\bullet\bullet\\\bullet\circ}}\right) - \underset{\mathrm{ATPmi}}{\nu_f}\left(\mathrm{T}_{\mathrm{M}\substack{\circ\circ\\\bullet\bullet\\\bullet\circ}}\right)$$

$$\frac{d\mathrm{T}_{\mathrm{M}\substack{\circ\circ\\\bullet\bullet\\\bullet\bullet}}}{dt} = \underset{\mathrm{ATPsm}}{\nu_f}\left(\mathrm{T}_{\mathrm{S}\substack{\circ\circ\\\bullet\bullet\\\bullet\bullet}}\right) + \underset{\mathrm{ATPmi}}{\nu_r}\left(\mathrm{T}_{\mathrm{I}\substack{\circ\circ\\\bullet\bullet\\\bullet\bullet}}\right) - \underset{\mathrm{ATPmi}}{\nu_f}\left(\mathrm{T}_{\mathrm{M}\substack{\circ\circ\\\bullet\bullet\\\bullet\bullet}}\right)$$

$$\frac{d\mathrm{T}_{\mathrm{M}\substack{\circ\bullet\\\bullet\circ\\\bullet\circ}}}{dt} = \underset{\mathrm{ATPsm}}{\nu_f}\left(\mathrm{T}_{\mathrm{S}\substack{\circ\bullet\\\bullet\circ\\\bullet\circ}}\right) + \underset{\mathrm{ATPmi}}{\nu_r}\left(\mathrm{T}_{\mathrm{I}\substack{\circ\bullet\\\bullet\circ\\\bullet\circ}}\right) - \underset{\mathrm{ATPmi}}{\nu_f}\left(\mathrm{T}_{\mathrm{M}\substack{\circ\bullet\\\bullet\circ\\\bullet\circ}}\right)$$

$$\frac{d\mathrm{T}_{\mathrm{M}\substack{\circ\bullet\\\bullet\circ\\\bullet\bullet}}}{dt} = \underset{\mathrm{ATPsm}}{\nu_f}\left(\mathrm{T}_{\mathrm{S}\substack{\circ\bullet\\\bullet\circ\\\bullet\bullet}}\right) + \underset{\mathrm{ATPmi}}{\nu_r}\left(\mathrm{T}_{\mathrm{I}\substack{\circ\bullet\\\bullet\circ\\\bullet\bullet}}\right) - \underset{\mathrm{ATPmi}}{\nu_f}\left(\mathrm{T}_{\mathrm{M}\substack{\circ\bullet\\\bullet\circ\\\bullet\bullet}}\right)$$

$$\frac{d\mathrm{T}_{\mathrm{M}\substack{\circ\bullet\\\bullet\bullet\\\bullet\circ}}}{dt} = \underset{\mathrm{ATPsm}}{\nu_f}\left(\mathrm{T}_{\mathrm{S}\substack{\circ\bullet\\\bullet\bullet\\\bullet\circ}}\right) + \underset{\mathrm{ATPmi}}{\nu_r}\left(\mathrm{T}_{\mathrm{I}\substack{\circ\bullet\\\bullet\bullet\\\bullet\circ}}\right) - \underset{\mathrm{ATPmi}}{\nu_f}\left(\mathrm{T}_{\mathrm{M}\substack{\circ\bullet\\\bullet\bullet\\\bullet\circ}}\right)$$

$$\frac{d\mathrm{T}_{\mathrm{M}\substack{\circ\bullet\\\bullet\bullet\\\bullet\bullet}}}{dt} = \underset{\mathrm{ATPsm}}{\nu_f}\left(\mathrm{T}_{\mathrm{S}\substack{\circ\bullet\\\bullet\bullet\\\bullet\bullet}}\right) + \underset{\mathrm{ATPmi}}{\nu_r}\left(\mathrm{T}_{\mathrm{I}\substack{\circ\bullet\\\bullet\bullet\\\bullet\bullet}}\right) - \underset{\mathrm{ATPmi}}{\nu_f}\left(\mathrm{T}_{\mathrm{M}\substack{\circ\bullet\\\bullet\bullet\\\bullet\bullet}}\right)$$

$$\frac{d\mathrm{T}_{\mathrm{M}\substack{\bullet\circ\\\circ\circ\\\circ\circ}}}{dt} = \underset{\mathrm{ATPsm}}{\nu_f}\left(\mathrm{T}_{\mathrm{S}\substack{\bullet\circ\\\circ\circ\\\circ\circ}}\right) + \underset{\mathrm{ATPmi}}{\nu_r}\left(\mathrm{T}_{\mathrm{I}\substack{\bullet\circ\\\circ\circ\\\circ\circ}}\right) - \underset{\mathrm{ATPmi}}{\nu_f}\left(\mathrm{T}_{\mathrm{M}\substack{\bullet\circ\\\circ\circ\\\circ\circ}}\right)$$

$$\frac{d\mathrm{T}_{\mathrm{M}\substack{\bullet\circ\\\circ\circ\\\circ\bullet}}}{dt} = \underset{\mathrm{ATPsm}}{\nu_f}\left(\mathrm{T}_{\mathrm{S}\substack{\bullet\circ\\\circ\circ\\\circ\bullet}}\right) + \underset{\mathrm{ATPmi}}{\nu_r}\left(\mathrm{T}_{\mathrm{I}\substack{\bullet\circ\\\circ\circ\\\circ\bullet}}\right) - \underset{\mathrm{ATPmi}}{\nu_f}\left(\mathrm{T}_{\mathrm{M}\substack{\bullet\circ\\\circ\circ\\\circ\bullet}}\right)$$

$$\frac{d\mathrm{T}_{\mathrm{M}\substack{\bullet\circ\\\circ\bullet\\\circ\circ}}}{dt} = \underset{\mathrm{ATPsm}}{\nu_f}\left(\mathrm{T}_{\mathrm{S}\substack{\bullet\circ\\\circ\bullet\\\circ\circ}}\right) + \underset{\mathrm{ATPmi}}{\nu_r}\left(\mathrm{T}_{\mathrm{I}\substack{\bullet\circ\\\circ\bullet\\\circ\circ}}\right) - \underset{\mathrm{ATPmi}}{\nu_f}\left(\mathrm{T}_{\mathrm{M}\substack{\bullet\circ\\\circ\bullet\\\circ\circ}}\right)$$

$$\frac{d\mathrm{T}_{\mathrm{M}\substack{\bullet\circ\\\circ\bullet\\\circ\bullet}}}{dt} = \underset{\mathrm{ATPsm}}{\nu_f}\left(\mathrm{T}_{\mathrm{S}\substack{\bullet\circ\\\circ\bullet\\\circ\bullet}}\right) + \underset{\mathrm{ATPmi}}{\nu_r}\left(\mathrm{T}_{\mathrm{I}\substack{\bullet\circ\\\circ\bullet\\\circ\bullet}}\right) - \underset{\mathrm{ATPmi}}{\nu_f}\left(\mathrm{T}_{\mathrm{M}\substack{\bullet\circ\\\circ\bullet\\\circ\bullet}}\right)$$

$$\frac{d\mathrm{T}_{\mathrm{M}\substack{\bullet\bullet\\\circ\circ\\\circ\circ}}}{dt} = \underset{\mathrm{ATPsm}}{\nu_f}\left(\mathrm{T}_{\mathrm{S}\substack{\bullet\bullet\\\circ\circ\\\circ\circ}}\right) + \underset{\mathrm{ATPmi}}{\nu_r}\left(\mathrm{T}_{\mathrm{I}\substack{\bullet\bullet\\\circ\circ\\\circ\circ}}\right) - \underset{\mathrm{ATPmi}}{\nu_f}\left(\mathrm{T}_{\mathrm{M}\substack{\bullet\bullet\\\circ\circ\\\circ\circ}}\right)$$

$$\frac{d\mathrm{T}_{\mathrm{M}\substack{\bullet\bullet\\\circ\circ\\\circ\bullet}}}{dt} = \underset{\mathrm{ATPsm}}{\nu_f}\left(\mathrm{T}_{\mathrm{S}\substack{\bullet\bullet\\\circ\circ\\\circ\bullet}}\right) + \underset{\mathrm{ATPmi}}{\nu_r}\left(\mathrm{T}_{\mathrm{I}\substack{\bullet\bullet\\\circ\circ\\\circ\bullet}}\right) - \underset{\mathrm{ATPmi}}{\nu_f}\left(\mathrm{T}_{\mathrm{M}\substack{\bullet\bullet\\\circ\circ\\\circ\bullet}}\right)$$

$$\frac{d\mathrm{T}_{\mathrm{M}\substack{\bullet\bullet\\\circ\bullet\\\circ\circ}}}{dt} = \underset{\mathrm{ATPsm}}{\nu_f}\left(\mathrm{T}_{\mathrm{S}\substack{\bullet\bullet\\\circ\bullet\\\circ\circ}}\right) + \underset{\mathrm{ATPmi}}{\nu_r}\left(\mathrm{T}_{\mathrm{I}\substack{\bullet\bullet\\\circ\bullet\\\circ\circ}}\right) - \underset{\mathrm{ATPmi}}{\nu_f}\left(\mathrm{T}_{\mathrm{M}\substack{\bullet\bullet\\\circ\bullet\\\circ\circ}}\right)$$

$$\frac{d\mathrm{T}_{\mathrm{M}\substack{\bullet\bullet\\\circ\bullet\\\circ\bullet}}}{dt} = \underset{\mathrm{ATPsm}}{\nu_f}\left(\mathrm{T}_{\mathrm{S}\substack{\bullet\bullet\\\circ\bullet\\\circ\bullet}}\right) + \underset{\mathrm{ATPmi}}{\nu_r}\left(\mathrm{T}_{\mathrm{I}\substack{\bullet\bullet\\\circ\bullet\\\circ\bullet}}\right) - \underset{\mathrm{ATPmi}}{\nu_f}\left(\mathrm{T}_{\mathrm{M}\substack{\bullet\bullet\\\circ\bullet\\\circ\bullet}}\right)$$

$$\frac{d\mathrm{T}_{\mathrm{M}\substack{\bullet\circ\\\circ\circ\\\bullet\circ}}}{dt} = \underset{\mathrm{ATPsm}}{\nu_f}\left(\mathrm{T}_{\mathrm{S}\substack{\bullet\circ\\\circ\circ\\\bullet\circ}}\right) + \underset{\mathrm{ATPmi}}{\nu_r}\left(\mathrm{T}_{\mathrm{I}\substack{\bullet\circ\\\circ\circ\\\bullet\circ}}\right) - \underset{\mathrm{ATPmi}}{\nu_f}\left(\mathrm{T}_{\mathrm{M}\substack{\bullet\circ\\\circ\circ\\\bullet\circ}}\right)$$

$$\frac{d\mathrm{T}_{\mathrm{M}\substack{\bullet\circ\\\circ\circ\\\bullet\bullet}}}{dt} = \overset{\nu_f}{\mathrm{ATP}_{\mathrm{SM}}}\left(\mathrm{T}_{\mathrm{S}\substack{\bullet\circ\\\circ\circ\\\bullet\bullet}}\right) + \overset{\nu_r}{\mathrm{ATP}_{\mathrm{MI}}}\left(\mathrm{T}_{\mathrm{I}\substack{\bullet\circ\\\circ\circ\\\bullet\bullet}}\right) - \overset{\nu_f}{\mathrm{ATP}_{\mathrm{MI}}}\left(\mathrm{T}_{\mathrm{M}\substack{\bullet\circ\\\circ\circ\\\bullet\bullet}}\right)$$

$$\frac{d\mathrm{T}_{\mathrm{M}\substack{\bullet\circ\\\circ\bullet\\\bullet\circ}}}{dt} = \overset{\nu_f}{\mathrm{ATP}_{\mathrm{SM}}}\left(\mathrm{T}_{\mathrm{S}\substack{\bullet\circ\\\circ\bullet\\\bullet\circ}}\right) + \overset{\nu_r}{\mathrm{ATP}_{\mathrm{MI}}}\left(\mathrm{T}_{\mathrm{I}\substack{\bullet\circ\\\circ\bullet\\\bullet\circ}}\right) - \overset{\nu_f}{\mathrm{ATP}_{\mathrm{MI}}}\left(\mathrm{T}_{\mathrm{M}\substack{\bullet\circ\\\circ\bullet\\\bullet\circ}}\right)$$

$$\frac{d\mathrm{T}_{\mathrm{M}\substack{\bullet\circ\\\circ\bullet\\\bullet\bullet}}}{dt} = \overset{\nu_f}{\mathrm{ATP}_{\mathrm{SM}}}\left(\mathrm{T}_{\mathrm{S}\substack{\bullet\circ\\\circ\bullet\\\bullet\bullet}}\right) + \overset{\nu_r}{\mathrm{ATP}_{\mathrm{MI}}}\left(\mathrm{T}_{\mathrm{I}\substack{\bullet\circ\\\circ\bullet\\\bullet\bullet}}\right) - \overset{\nu_f}{\mathrm{ATP}_{\mathrm{MI}}}\left(\mathrm{T}_{\mathrm{M}\substack{\bullet\circ\\\circ\bullet\\\bullet\bullet}}\right)$$

$$\frac{d\mathrm{T}_{\mathrm{M}\substack{\bullet\bullet\\\circ\circ\\\bullet\circ}}}{dt} = \overset{\nu_f}{\mathrm{ATP}_{\mathrm{SM}}}\left(\mathrm{T}_{\mathrm{S}\substack{\bullet\bullet\\\circ\circ\\\bullet\circ}}\right) + \overset{\nu_r}{\mathrm{ATP}_{\mathrm{MI}}}\left(\mathrm{T}_{\mathrm{I}\substack{\bullet\bullet\\\circ\circ\\\bullet\circ}}\right) - \overset{\nu_f}{\mathrm{ATP}_{\mathrm{MI}}}\left(\mathrm{T}_{\mathrm{M}\substack{\bullet\bullet\\\circ\circ\\\bullet\circ}}\right)$$

$$\frac{d\mathrm{T}_{\mathrm{M}\substack{\bullet\bullet\\\circ\circ\\\bullet\bullet}}}{dt} = \overset{\nu_f}{\mathrm{ATP}_{\mathrm{SM}}}\left(\mathrm{T}_{\mathrm{S}\substack{\bullet\bullet\\\circ\circ\\\bullet\bullet}}\right) + \overset{\nu_r}{\mathrm{ATP}_{\mathrm{MI}}}\left(\mathrm{T}_{\mathrm{I}\substack{\bullet\bullet\\\circ\circ\\\bullet\bullet}}\right) - \overset{\nu_f}{\mathrm{ATP}_{\mathrm{MI}}}\left(\mathrm{T}_{\mathrm{M}\substack{\bullet\bullet\\\circ\circ\\\bullet\bullet}}\right)$$

$$\frac{d\mathrm{T}_{\mathrm{M}\substack{\bullet\bullet\\\circ\bullet\\\bullet\circ}}}{dt} = \overset{\nu_f}{\mathrm{ATP}_{\mathrm{SM}}}\left(\mathrm{T}_{\mathrm{S}\substack{\bullet\bullet\\\circ\bullet\\\bullet\circ}}\right) + \overset{\nu_r}{\mathrm{ATP}_{\mathrm{MI}}}\left(\mathrm{T}_{\mathrm{I}\substack{\bullet\bullet\\\circ\bullet\\\bullet\circ}}\right) - \overset{\nu_f}{\mathrm{ATP}_{\mathrm{MI}}}\left(\mathrm{T}_{\mathrm{M}\substack{\bullet\bullet\\\circ\bullet\\\bullet\circ}}\right)$$

$$\frac{d\mathrm{T}_{\mathrm{M}\substack{\bullet\bullet\\\circ\bullet\\\bullet\bullet}}}{dt} = \overset{\nu_f}{\mathrm{ATP}_{\mathrm{SM}}}\left(\mathrm{T}_{\mathrm{S}\substack{\bullet\bullet\\\circ\bullet\\\bullet\bullet}}\right) + \overset{\nu_r}{\mathrm{ATP}_{\mathrm{MI}}}\left(\mathrm{T}_{\mathrm{I}\substack{\bullet\bullet\\\circ\bullet\\\bullet\bullet}}\right) - \overset{\nu_f}{\mathrm{ATP}_{\mathrm{MI}}}\left(\mathrm{T}_{\mathrm{M}\substack{\bullet\bullet\\\circ\bullet\\\bullet\bullet}}\right)$$

$$\frac{d\mathrm{T}_{\mathrm{M}\substack{\bullet\circ\\\bullet\circ\\\circ\circ}}}{dt} = \overset{\nu_f}{\mathrm{ATP}_{\mathrm{SM}}}\left(\mathrm{T}_{\mathrm{S}\substack{\bullet\circ\\\bullet\circ\\\circ\circ}}\right) + \overset{\nu_r}{\mathrm{ATP}_{\mathrm{MI}}}\left(\mathrm{T}_{\mathrm{I}\substack{\bullet\circ\\\bullet\circ\\\circ\circ}}\right) - \overset{\nu_f}{\mathrm{ATP}_{\mathrm{MI}}}\left(\mathrm{T}_{\mathrm{M}\substack{\bullet\circ\\\bullet\circ\\\circ\circ}}\right)$$

$$\frac{d\mathrm{T}_{\mathrm{M}\substack{\bullet\circ\\\bullet\circ\\\circ\bullet}}}{dt} = \overset{\nu_f}{\mathrm{ATP}_{\mathrm{SM}}}\left(\mathrm{T}_{\mathrm{S}\substack{\bullet\circ\\\bullet\circ\\\circ\bullet}}\right) + \overset{\nu_r}{\mathrm{ATP}_{\mathrm{MI}}}\left(\mathrm{T}_{\mathrm{I}\substack{\bullet\circ\\\bullet\circ\\\circ\bullet}}\right) - \overset{\nu_f}{\mathrm{ATP}_{\mathrm{MI}}}\left(\mathrm{T}_{\mathrm{M}\substack{\bullet\circ\\\bullet\circ\\\circ\bullet}}\right)$$

$$\frac{d\mathrm{T}_{\mathrm{M}\substack{\bullet\circ\\\bullet\bullet\\\circ\circ}}}{dt} = \overset{\nu_f}{\mathrm{ATP}_{\mathrm{SM}}}\left(\mathrm{T}_{\mathrm{S}\substack{\bullet\circ\\\bullet\bullet\\\circ\circ}}\right) + \overset{\nu_r}{\mathrm{ATP}_{\mathrm{MI}}}\left(\mathrm{T}_{\mathrm{I}\substack{\bullet\circ\\\bullet\bullet\\\circ\circ}}\right) - \overset{\nu_f}{\mathrm{ATP}_{\mathrm{MI}}}\left(\mathrm{T}_{\mathrm{M}\substack{\bullet\circ\\\bullet\bullet\\\circ\circ}}\right)$$

$$\frac{d\mathrm{T}_{\mathrm{M}\substack{\bullet\circ\\\bullet\bullet\\\circ\bullet}}}{dt} = \overset{\nu_f}{\mathrm{ATP}_{\mathrm{SM}}}\left(\mathrm{T}_{\mathrm{S}\substack{\bullet\circ\\\bullet\bullet\\\circ\bullet}}\right) + \overset{\nu_r}{\mathrm{ATP}_{\mathrm{MI}}}\left(\mathrm{T}_{\mathrm{I}\substack{\bullet\circ\\\bullet\bullet\\\circ\bullet}}\right) - \overset{\nu_f}{\mathrm{ATP}_{\mathrm{MI}}}\left(\mathrm{T}_{\mathrm{M}\substack{\bullet\circ\\\bullet\bullet\\\circ\bullet}}\right)$$

$$\frac{d\mathrm{T}_{\mathrm{M}\substack{\bullet\bullet\\\bullet\circ\\\circ\circ}}}{dt} = \overset{\nu_f}{\mathrm{ATP}_{\mathrm{SM}}}\left(\mathrm{T}_{\mathrm{S}\substack{\bullet\bullet\\\bullet\circ\\\circ\circ}}\right) + \overset{\nu_r}{\mathrm{ATP}_{\mathrm{MI}}}\left(\mathrm{T}_{\mathrm{I}\substack{\bullet\bullet\\\bullet\circ\\\circ\circ}}\right) - \overset{\nu_f}{\mathrm{ATP}_{\mathrm{MI}}}\left(\mathrm{T}_{\mathrm{M}\substack{\bullet\bullet\\\bullet\circ\\\circ\circ}}\right)$$

$$\frac{d\mathrm{T}_{\mathrm{M}\substack{\bullet\bullet\\\bullet\circ\\\circ\bullet}}}{dt} = \overset{\nu_f}{\mathrm{ATP}_{\mathrm{SM}}}\left(\mathrm{T}_{\mathrm{S}\substack{\bullet\bullet\\\bullet\circ\\\circ\bullet}}\right) + \overset{\nu_r}{\mathrm{ATP}_{\mathrm{MI}}}\left(\mathrm{T}_{\mathrm{I}\substack{\bullet\bullet\\\bullet\circ\\\circ\bullet}}\right) - \overset{\nu_f}{\mathrm{ATP}_{\mathrm{MI}}}\left(\mathrm{T}_{\mathrm{M}\substack{\bullet\bullet\\\bullet\circ\\\circ\bullet}}\right)$$

$$\frac{d\mathrm{T}_{\mathrm{M}\substack{\bullet\bullet\\\bullet\bullet\\\circ\circ}}}{dt} = \overset{\nu_f}{\mathrm{ATP}_{\mathrm{SM}}}\left(\mathrm{T}_{\mathrm{S}\substack{\bullet\bullet\\\bullet\bullet\\\circ\circ}}\right) + \overset{\nu_r}{\mathrm{ATP}_{\mathrm{MI}}}\left(\mathrm{T}_{\mathrm{I}\substack{\bullet\bullet\\\bullet\bullet\\\circ\circ}}\right) - \overset{\nu_f}{\mathrm{ATP}_{\mathrm{MI}}}\left(\mathrm{T}_{\mathrm{M}\substack{\bullet\bullet\\\bullet\bullet\\\circ\circ}}\right)$$

$$\frac{d\mathrm{T}_{\mathrm{M}\substack{\bullet\bullet\\\bullet\bullet\\\circ\bullet}}}{dt} = \overset{\nu_f}{\mathrm{ATP}_{\mathrm{SM}}}\left(\mathrm{T}_{\mathrm{S}\substack{\bullet\bullet\\\bullet\bullet\\\circ\bullet}}\right) + \overset{\nu_r}{\mathrm{ATP}_{\mathrm{MI}}}\left(\mathrm{T}_{\mathrm{I}\substack{\bullet\bullet\\\bullet\bullet\\\circ\bullet}}\right) - \overset{\nu_f}{\mathrm{ATP}_{\mathrm{MI}}}\left(\mathrm{T}_{\mathrm{M}\substack{\bullet\bullet\\\bullet\bullet\\\circ\bullet}}\right)$$

$$\frac{d\mathrm{T}_{\mathrm{M}\substack{\bullet\circ\\\bullet\circ\\\bullet\circ}}}{dt} = \overset{\nu_f}{\mathrm{ATP}_{\mathrm{SM}}}\left(\mathrm{T}_{\mathrm{S}\substack{\bullet\circ\\\bullet\circ\\\bullet\circ}}\right) + \overset{\nu_r}{\mathrm{ATP}_{\mathrm{MI}}}\left(\mathrm{T}_{\mathrm{I}\substack{\bullet\circ\\\bullet\circ\\\bullet\circ}}\right) - \overset{\nu_f}{\mathrm{ATP}_{\mathrm{MI}}}\left(\mathrm{T}_{\mathrm{M}\substack{\bullet\circ\\\bullet\circ\\\bullet\circ}}\right)$$

$$\frac{d\mathrm{T}_{\mathrm{M}\substack{\bullet\circ\\\bullet\circ\\\bullet\bullet}}}{dt} = \overset{\nu_f}{\mathrm{ATP}_{\mathrm{SM}}}\left(\mathrm{T}_{\mathrm{S}\substack{\bullet\circ\\\bullet\circ\\\bullet\bullet}}\right) + \overset{\nu_r}{\mathrm{ATP}_{\mathrm{MI}}}\left(\mathrm{T}_{\mathrm{I}\substack{\bullet\circ\\\bullet\circ\\\bullet\bullet}}\right) - \overset{\nu_f}{\mathrm{ATP}_{\mathrm{MI}}}\left(\mathrm{T}_{\mathrm{M}\substack{\bullet\circ\\\bullet\circ\\\bullet\bullet}}\right)$$

$$\frac{d\mathrm{T}_{\mathrm{M}\substack{\bullet\circ\\\bullet\bullet\\\bullet\circ}}}{dt} = \overset{\nu_f}{\mathrm{ATP}_{\mathrm{SM}}}\left(\mathrm{T}_{\mathrm{S}\substack{\bullet\circ\\\bullet\bullet\\\bullet\circ}}\right) + \overset{\nu_r}{\mathrm{ATP}_{\mathrm{MI}}}\left(\mathrm{T}_{\mathrm{I}\substack{\bullet\circ\\\bullet\bullet\\\bullet\circ}}\right) - \overset{\nu_f}{\mathrm{ATP}_{\mathrm{MI}}}\left(\mathrm{T}_{\mathrm{M}\substack{\bullet\circ\\\bullet\bullet\\\bullet\circ}}\right)$$

$$\frac{d\mathrm{T}_{\mathrm{M}\substack{\bullet\circ\\\bullet\bullet\\\bullet\bullet}}}{dt} = \overset{\nu_f}{\mathrm{ATP}_{\mathrm{SM}}}\left(\mathrm{T}_{\mathrm{S}\substack{\bullet\circ\\\bullet\bullet\\\bullet\bullet}}\right) + \overset{\nu_r}{\mathrm{ATP}_{\mathrm{MI}}}\left(\mathrm{T}_{\mathrm{I}\substack{\bullet\circ\\\bullet\bullet\\\bullet\bullet}}\right) - \overset{\nu_f}{\mathrm{ATP}_{\mathrm{MI}}}\left(\mathrm{T}_{\mathrm{M}\substack{\bullet\circ\\\bullet\bullet\\\bullet\bullet}}\right)$$

$$\frac{d\mathrm{T}_{\mathrm{M}\substack{\bullet\bullet\\\bullet\circ\\\bullet\circ}}}{dt} = \overset{\nu_f}{\mathrm{ATP}_{\mathrm{SM}}}\left(\mathrm{T}_{\mathrm{S}\substack{\bullet\bullet\\\bullet\circ\\\bullet\circ}}\right) + \overset{\nu_r}{\mathrm{ATP}_{\mathrm{MI}}}\left(\mathrm{T}_{\mathrm{I}\substack{\bullet\bullet\\\bullet\circ\\\bullet\circ}}\right) - \overset{\nu_f}{\mathrm{ATP}_{\mathrm{MI}}}\left(\mathrm{T}_{\mathrm{M}\substack{\bullet\bullet\\\bullet\circ\\\bullet\circ}}\right)$$

$$\frac{d\mathrm{T}_{\mathrm{M}\substack{\bullet\bullet\\\bullet\circ\\\bullet\bullet}}}{dt} = \overset{\nu_f}{\mathrm{ATP}_{\mathrm{SM}}}\left(\mathrm{T}_{\mathrm{S}\substack{\bullet\bullet\\\bullet\circ\\\bullet\bullet}}\right) + \overset{\nu_r}{\mathrm{ATP}_{\mathrm{MI}}}\left(\mathrm{T}_{\mathrm{I}\substack{\bullet\bullet\\\bullet\circ\\\bullet\bullet}}\right) - \overset{\nu_f}{\mathrm{ATP}_{\mathrm{MI}}}\left(\mathrm{T}_{\mathrm{M}\substack{\bullet\bullet\\\bullet\circ\\\bullet\bullet}}\right)$$

$$\frac{d\mathrm{T_M}^{\substack{\bullet\bullet\\\bullet\bullet\\\bullet\circ}}}{dt} = \underset{\textsc{ATPsm}}{\nu_f}\big(\mathrm{T_S}^{\substack{\bullet\bullet\\\bullet\bullet\\\bullet\circ}}\big) + \underset{\textsc{ATPmi}}{\nu_r}\big(\mathrm{T_I}^{\substack{\bullet\bullet\\\bullet\bullet\\\bullet\circ}}\big) - \underset{\textsc{ATPmi}}{\nu_f}\big(\mathrm{T_M}^{\substack{\bullet\bullet\\\bullet\bullet\\\bullet\circ}}\big)$$

$$\frac{d\mathrm{T_M}^{\substack{\bullet\bullet\\\bullet\bullet\\\bullet\bullet}}}{dt} = \underset{\textsc{ATPsm}}{\nu_f}\big(\mathrm{T_S}^{\substack{\bullet\bullet\\\bullet\bullet\\\bullet\bullet}}\big) + \underset{\textsc{ATPmi}}{\nu_r}\big(\mathrm{T_I}^{\substack{\bullet\bullet\\\bullet\bullet\\\bullet\bullet}}\big) - \underset{\textsc{ATPmi}}{\nu_f}\big(\mathrm{T_M}^{\substack{\bullet\bullet\\\bullet\bullet\\\bullet\bullet}}\big)$$

$$\frac{d\mathrm{T_O}^{\substack{\circ\circ\\\circ\circ\\\circ\circ}}}{dt} = \underset{\textsc{ATPio}}{\nu_f}\big(\mathrm{T_I}^{\substack{\circ\circ\\\circ\circ\\\circ\circ}}\big) + \underset{\textsc{AKo}}{\nu_r}\big(\mathrm{D_O}^{\substack{\circ\\\circ\\\circ}}\mathrm{D_O}^{\substack{\circ\\\circ\\\circ}}\big) + \underset{\textsc{CKo}}{\nu_r}\big(\mathrm{D_O}^{\substack{\circ\\\circ\\\circ}}\mathrm{C_O}^{\substack{\circ\\\circ\\\circ}}\big) - \underset{\textsc{AKo}}{\nu_f}\big(\mathrm{T_O}^{\substack{\circ\circ\\\circ\circ\\\circ\circ}}\big) - \underset{\textsc{ATPoe}}{\nu_f}\big(\mathrm{T_O}^{\substack{\circ\circ\\\circ\circ\\\circ\circ}}\big) - \underset{\textsc{CKo}}{\nu_f}\big(\mathrm{T_O}^{\substack{\circ\circ\\\circ\circ\\\circ\circ}}\big) - \underset{\textsc{ATPio}}{\nu_r}\big(\mathrm{T_O}^{\substack{\circ\circ\\\circ\circ\\\circ\circ}}\big)$$

$$\frac{d\mathrm{T_O}^{\substack{\circ\circ\\\circ\circ\\\circ\bullet}}}{dt} = \underset{\textsc{ATPio}}{\nu_f}\big(\mathrm{T_I}^{\substack{\circ\circ\\\circ\circ\\\circ\bullet}}\big) + \underset{\textsc{AKo}}{\nu_r}\big(\mathrm{D_O}^{\substack{\circ\\\circ\\\bullet}}\mathrm{D_O}^{\substack{\circ\\\circ\\\circ}}\big) + \underset{\textsc{CKo}}{\nu_r}\big(\mathrm{D_O}^{\substack{\circ\\\circ\\\circ}}\mathrm{C_O}^{\substack{\circ\\\circ\\\bullet}}\big) - \underset{\textsc{AKo}}{\nu_f}\big(\mathrm{T_O}^{\substack{\circ\circ\\\circ\circ\\\circ\bullet}}\big) - \underset{\textsc{ATPoe}}{\nu_f}\big(\mathrm{T_O}^{\substack{\circ\circ\\\circ\circ\\\circ\bullet}}\big) - \underset{\textsc{CKo}}{\nu_f}\big(\mathrm{T_O}^{\substack{\circ\circ\\\circ\circ\\\circ\bullet}}\big) - \underset{\textsc{ATPio}}{\nu_r}\big(\mathrm{T_O}^{\substack{\circ\circ\\\circ\circ\\\circ\bullet}}\big)$$

$$\frac{d\mathrm{T_O}^{\substack{\circ\circ\\\circ\bullet\\\circ\circ}}}{dt} = \underset{\textsc{ATPio}}{\nu_f}\big(\mathrm{T_I}^{\substack{\circ\circ\\\circ\bullet\\\circ\circ}}\big) + \underset{\textsc{AKo}}{\nu_r}\big(\mathrm{D_O}^{\substack{\circ\\\bullet\\\circ}}\mathrm{D_O}^{\substack{\circ\\\circ\\\circ}}\big) + \underset{\textsc{CKo}}{\nu_r}\big(\mathrm{D_O}^{\substack{\circ\\\circ\\\circ}}\mathrm{C_O}^{\substack{\circ\\\bullet\\\circ}}\big) - \underset{\textsc{AKo}}{\nu_f}\big(\mathrm{T_O}^{\substack{\circ\circ\\\circ\bullet\\\circ\circ}}\big) - \underset{\textsc{ATPoe}}{\nu_f}\big(\mathrm{T_O}^{\substack{\circ\circ\\\circ\bullet\\\circ\circ}}\big) - \underset{\textsc{CKo}}{\nu_f}\big(\mathrm{T_O}^{\substack{\circ\circ\\\circ\bullet\\\circ\circ}}\big) - \underset{\textsc{ATPio}}{\nu_r}\big(\mathrm{T_O}^{\substack{\circ\circ\\\circ\bullet\\\circ\circ}}\big)$$

$$\frac{d\mathrm{T_O}^{\substack{\circ\circ\\\circ\bullet\\\circ\bullet}}}{dt} = \underset{\textsc{ATPio}}{\nu_f}\big(\mathrm{T_I}^{\substack{\circ\circ\\\circ\bullet\\\circ\bullet}}\big) + \underset{\textsc{AKo}}{\nu_r}\big(\mathrm{D_O}^{\substack{\circ\\\bullet\\\bullet}}\mathrm{D_O}^{\substack{\circ\\\circ\\\circ}}\big) + \underset{\textsc{CKo}}{\nu_r}\big(\mathrm{D_O}^{\substack{\circ\\\circ\\\circ}}\mathrm{C_O}^{\substack{\circ\\\bullet\\\bullet}}\big) - \underset{\textsc{AKo}}{\nu_f}\big(\mathrm{T_O}^{\substack{\circ\circ\\\circ\bullet\\\circ\bullet}}\big) - \underset{\textsc{ATPoe}}{\nu_f}\big(\mathrm{T_O}^{\substack{\circ\circ\\\circ\bullet\\\circ\bullet}}\big) - \underset{\textsc{CKo}}{\nu_f}\big(\mathrm{T_O}^{\substack{\circ\circ\\\circ\bullet\\\circ\bullet}}\big) - \underset{\textsc{ATPio}}{\nu_r}\big(\mathrm{T_O}^{\substack{\circ\circ\\\circ\bullet\\\circ\bullet}}\big)$$

$$\frac{d\mathrm{T_O}^{\substack{\circ\bullet\\\circ\circ\\\circ\circ}}}{dt} = \underset{\textsc{ATPio}}{\nu_f}\big(\mathrm{T_I}^{\substack{\circ\bullet\\\circ\circ\\\circ\circ}}\big) + \underset{\textsc{AKo}}{\nu_r}\big(\mathrm{D_O}^{\substack{\bullet\\\circ\\\circ}}\mathrm{D_O}^{\substack{\circ\\\circ\\\circ}}\big) + \underset{\textsc{CKo}}{\nu_r}\big(\mathrm{D_O}^{\substack{\circ\\\circ\\\circ}}\mathrm{C_O}^{\substack{\bullet\\\circ\\\circ}}\big) - \underset{\textsc{AKo}}{\nu_f}\big(\mathrm{T_O}^{\substack{\circ\bullet\\\circ\circ\\\circ\circ}}\big) - \underset{\textsc{ATPoe}}{\nu_f}\big(\mathrm{T_O}^{\substack{\circ\bullet\\\circ\circ\\\circ\circ}}\big) - \underset{\textsc{CKo}}{\nu_f}\big(\mathrm{T_O}^{\substack{\circ\bullet\\\circ\circ\\\circ\circ}}\big) - \underset{\textsc{ATPio}}{\nu_r}\big(\mathrm{T_O}^{\substack{\circ\bullet\\\circ\circ\\\circ\circ}}\big)$$

$$\frac{d\mathrm{T_O}^{\substack{\circ\bullet\\\circ\circ\\\circ\bullet}}}{dt} = \underset{\textsc{ATPio}}{\nu_f}\big(\mathrm{T_I}^{\substack{\circ\bullet\\\circ\circ\\\circ\bullet}}\big) + \underset{\textsc{AKo}}{\nu_r}\big(\mathrm{D_O}^{\substack{\bullet\\\circ\\\bullet}}\mathrm{D_O}^{\substack{\circ\\\circ\\\circ}}\big) + \underset{\textsc{CKo}}{\nu_r}\big(\mathrm{D_O}^{\substack{\circ\\\circ\\\circ}}\mathrm{C_O}^{\substack{\bullet\\\circ\\\bullet}}\big) - \underset{\textsc{AKo}}{\nu_f}\big(\mathrm{T_O}^{\substack{\circ\bullet\\\circ\circ\\\circ\bullet}}\big) - \underset{\textsc{ATPoe}}{\nu_f}\big(\mathrm{T_O}^{\substack{\circ\bullet\\\circ\circ\\\circ\bullet}}\big) - \underset{\textsc{CKo}}{\nu_f}\big(\mathrm{T_O}^{\substack{\circ\bullet\\\circ\circ\\\circ\bullet}}\big) - \underset{\textsc{ATPio}}{\nu_r}\big(\mathrm{T_O}^{\substack{\circ\bullet\\\circ\circ\\\circ\bullet}}\big)$$

$$\frac{d\mathrm{T_O}^{\substack{\circ\bullet\\\circ\bullet\\\circ\circ}}}{dt} = \underset{\textsc{ATPio}}{\nu_f}\big(\mathrm{T_I}^{\substack{\circ\bullet\\\circ\bullet\\\circ\circ}}\big) + \underset{\textsc{AKo}}{\nu_r}\big(\mathrm{D_O}^{\substack{\bullet\\\bullet\\\circ}}\mathrm{D_O}^{\substack{\circ\\\circ\\\circ}}\big) + \underset{\textsc{CKo}}{\nu_r}\big(\mathrm{D_O}^{\substack{\circ\\\circ\\\circ}}\mathrm{C_O}^{\substack{\bullet\\\bullet\\\circ}}\big) - \underset{\textsc{AKo}}{\nu_f}\big(\mathrm{T_O}^{\substack{\circ\bullet\\\circ\bullet\\\circ\circ}}\big) - \underset{\textsc{ATPoe}}{\nu_f}\big(\mathrm{T_O}^{\substack{\circ\bullet\\\circ\bullet\\\circ\circ}}\big) - \underset{\textsc{CKo}}{\nu_f}\big(\mathrm{T_O}^{\substack{\circ\bullet\\\circ\bullet\\\circ\circ}}\big) - \underset{\textsc{ATPio}}{\nu_r}\big(\mathrm{T_O}^{\substack{\circ\bullet\\\circ\bullet\\\circ\circ}}\big)$$

$$\frac{d\mathrm{T_O}^{\substack{\circ\bullet\\\circ\bullet\\\circ\bullet}}}{dt} = \underset{\textsc{ATPio}}{\nu_f}\big(\mathrm{T_I}^{\substack{\circ\bullet\\\circ\bullet\\\circ\bullet}}\big) + \underset{\textsc{AKo}}{\nu_r}\big(\mathrm{D_O}^{\substack{\bullet\\\bullet\\\bullet}}\mathrm{D_O}^{\substack{\circ\\\circ\\\circ}}\big) + \underset{\textsc{CKo}}{\nu_r}\big(\mathrm{D_O}^{\substack{\circ\\\circ\\\circ}}\mathrm{C_O}^{\substack{\bullet\\\bullet\\\bullet}}\big) - \underset{\textsc{AKo}}{\nu_f}\big(\mathrm{T_O}^{\substack{\circ\bullet\\\circ\bullet\\\circ\bullet}}\big) - \underset{\textsc{ATPoe}}{\nu_f}\big(\mathrm{T_O}^{\substack{\circ\bullet\\\circ\bullet\\\circ\bullet}}\big) - \underset{\textsc{CKo}}{\nu_f}\big(\mathrm{T_O}^{\substack{\circ\bullet\\\circ\bullet\\\circ\bullet}}\big) - \underset{\textsc{ATPio}}{\nu_r}\big(\mathrm{T_O}^{\substack{\circ\bullet\\\circ\bullet\\\circ\bullet}}\big)$$

$$\frac{d\mathrm{T_O}^{\substack{\circ\circ\\\circ\circ\\\bullet\circ}}}{dt} = \underset{\textsc{ATPio}}{\nu_f}\big(\mathrm{T_I}^{\substack{\circ\circ\\\circ\circ\\\bullet\circ}}\big) + \underset{\textsc{AKo}}{\nu_r}\big(\mathrm{D_O}^{\substack{\circ\\\circ\\\bullet}}\mathrm{D_O}^{\substack{\circ\\\circ\\\circ}}\big) + \underset{\textsc{CKo}}{\nu_r}\big(\mathrm{D_O}^{\substack{\circ\\\circ\\\bullet}}\mathrm{C_O}^{\substack{\circ\\\circ\\\circ}}\big) - \underset{\textsc{AKo}}{\nu_f}\big(\mathrm{T_O}^{\substack{\circ\circ\\\circ\circ\\\bullet\circ}}\big) - \underset{\textsc{ATPoe}}{\nu_f}\big(\mathrm{T_O}^{\substack{\circ\circ\\\circ\circ\\\bullet\circ}}\big) - \underset{\textsc{CKo}}{\nu_f}\big(\mathrm{T_O}^{\substack{\circ\circ\\\circ\circ\\\bullet\circ}}\big) - \underset{\textsc{ATPio}}{\nu_r}\big(\mathrm{T_O}^{\substack{\circ\circ\\\circ\circ\\\bullet\circ}}\big)$$

$$\frac{d\mathrm{T_O}^{\substack{\circ\circ\\\circ\circ\\\bullet\bullet}}}{dt} = \underset{\textsc{ATPio}}{\nu_f}\big(\mathrm{T_I}^{\substack{\circ\circ\\\circ\circ\\\bullet\bullet}}\big) + \underset{\textsc{AKo}}{\nu_r}\big(\mathrm{D_O}^{\substack{\circ\\\circ\\\bullet}}\mathrm{D_O}^{\substack{\circ\\\circ\\\bullet}}\big) + \underset{\textsc{CKo}}{\nu_r}\big(\mathrm{D_O}^{\substack{\circ\\\circ\\\bullet}}\mathrm{C_O}^{\substack{\circ\\\circ\\\bullet}}\big) - \underset{\textsc{AKo}}{\nu_f}\big(\mathrm{T_O}^{\substack{\circ\circ\\\circ\circ\\\bullet\bullet}}\big) - \underset{\textsc{ATPoe}}{\nu_f}\big(\mathrm{T_O}^{\substack{\circ\circ\\\circ\circ\\\bullet\bullet}}\big) - \underset{\textsc{CKo}}{\nu_f}\big(\mathrm{T_O}^{\substack{\circ\circ\\\circ\circ\\\bullet\bullet}}\big) - \underset{\textsc{ATPio}}{\nu_r}\big(\mathrm{T_O}^{\substack{\circ\circ\\\circ\circ\\\bullet\bullet}}\big)$$

$$\frac{d\mathrm{T_O}^{\substack{\circ\circ\\\circ\bullet\\\bullet\circ}}}{dt} = \underset{\textsc{ATPio}}{\nu_f}\big(\mathrm{T_I}^{\substack{\circ\circ\\\circ\bullet\\\bullet\circ}}\big) + \underset{\textsc{AKo}}{\nu_r}\big(\mathrm{D_O}^{\substack{\circ\\\bullet\\\circ}}\mathrm{D_O}^{\substack{\circ\\\circ\\\bullet}}\big) + \underset{\textsc{CKo}}{\nu_r}\big(\mathrm{D_O}^{\substack{\circ\\\circ\\\bullet}}\mathrm{C_O}^{\substack{\circ\\\bullet\\\circ}}\big) - \underset{\textsc{AKo}}{\nu_f}\big(\mathrm{T_O}^{\substack{\circ\circ\\\circ\bullet\\\bullet\circ}}\big) - \underset{\textsc{ATPoe}}{\nu_f}\big(\mathrm{T_O}^{\substack{\circ\circ\\\circ\bullet\\\bullet\circ}}\big) - \underset{\textsc{CKo}}{\nu_f}\big(\mathrm{T_O}^{\substack{\circ\circ\\\circ\bullet\\\bullet\circ}}\big) - \underset{\textsc{ATPio}}{\nu_r}\big(\mathrm{T_O}^{\substack{\circ\circ\\\circ\bullet\\\bullet\circ}}\big)$$

$$\frac{d\mathrm{T_O}^{\substack{\circ\circ\\\circ\bullet\\\bullet\bullet}}}{dt} = \underset{\textsc{ATPio}}{\nu_f}\big(\mathrm{T_I}^{\substack{\circ\circ\\\circ\bullet\\\bullet\bullet}}\big) + \underset{\textsc{AKo}}{\nu_r}\big(\mathrm{D_O}^{\substack{\circ\\\bullet\\\bullet}}\mathrm{D_O}^{\substack{\circ\\\circ\\\bullet}}\big) + \underset{\textsc{CKo}}{\nu_r}\big(\mathrm{D_O}^{\substack{\circ\\\circ\\\bullet}}\mathrm{C_O}^{\substack{\circ\\\bullet\\\bullet}}\big) - \underset{\textsc{AKo}}{\nu_f}\big(\mathrm{T_O}^{\substack{\circ\circ\\\circ\bullet\\\bullet\bullet}}\big) - \underset{\textsc{ATPoe}}{\nu_f}\big(\mathrm{T_O}^{\substack{\circ\circ\\\circ\bullet\\\bullet\bullet}}\big) - \underset{\textsc{CKo}}{\nu_f}\big(\mathrm{T_O}^{\substack{\circ\circ\\\circ\bullet\\\bullet\bullet}}\big) - \underset{\textsc{ATPio}}{\nu_r}\big(\mathrm{T_O}^{\substack{\circ\circ\\\circ\bullet\\\bullet\bullet}}\big)$$

$$\frac{d\mathrm{T_O}^{\substack{\circ\bullet\\\circ\circ\\\bullet\circ}}}{dt} = \underset{\textsc{ATPio}}{\nu_f}\big(\mathrm{T_I}^{\substack{\circ\bullet\\\circ\circ\\\bullet\circ}}\big) + \underset{\textsc{AKo}}{\nu_r}\big(\mathrm{D_O}^{\substack{\bullet\\\circ\\\circ}}\mathrm{D_O}^{\substack{\circ\\\circ\\\bullet}}\big) + \underset{\textsc{CKo}}{\nu_r}\big(\mathrm{D_O}^{\substack{\circ\\\circ\\\bullet}}\mathrm{C_O}^{\substack{\bullet\\\circ\\\circ}}\big) - \underset{\textsc{AKo}}{\nu_f}\big(\mathrm{T_O}^{\substack{\circ\bullet\\\circ\circ\\\bullet\circ}}\big) - \underset{\textsc{ATPoe}}{\nu_f}\big(\mathrm{T_O}^{\substack{\circ\bullet\\\circ\circ\\\bullet\circ}}\big) - \underset{\textsc{CKo}}{\nu_f}\big(\mathrm{T_O}^{\substack{\circ\bullet\\\circ\circ\\\bullet\circ}}\big) - \underset{\textsc{ATPio}}{\nu_r}\big(\mathrm{T_O}^{\substack{\circ\bullet\\\circ\circ\\\bullet\circ}}\big)$$

$$\frac{d\mathrm{T_O}^{\substack{\circ\bullet\\\circ\circ\\\bullet\bullet}}}{dt} = \underset{\textsc{ATPio}}{\nu_f}\big(\mathrm{T_I}^{\substack{\circ\bullet\\\circ\circ\\\bullet\bullet}}\big) + \underset{\textsc{AKo}}{\nu_r}\big(\mathrm{D_O}^{\substack{\bullet\\\circ\\\bullet}}\mathrm{D_O}^{\substack{\circ\\\circ\\\bullet}}\big) + \underset{\textsc{CKo}}{\nu_r}\big(\mathrm{D_O}^{\substack{\circ\\\circ\\\bullet}}\mathrm{C_O}^{\substack{\bullet\\\circ\\\bullet}}\big) - \underset{\textsc{AKo}}{\nu_f}\big(\mathrm{T_O}^{\substack{\circ\bullet\\\circ\circ\\\bullet\bullet}}\big) - \underset{\textsc{ATPoe}}{\nu_f}\big(\mathrm{T_O}^{\substack{\circ\bullet\\\circ\circ\\\bullet\bullet}}\big) - \underset{\textsc{CKo}}{\nu_f}\big(\mathrm{T_O}^{\substack{\circ\bullet\\\circ\circ\\\bullet\bullet}}\big) - \underset{\textsc{ATPio}}{\nu_r}\big(\mathrm{T_O}^{\substack{\circ\bullet\\\circ\circ\\\bullet\bullet}}\big)$$

$$\frac{d\mathrm{T_O}^{\substack{\circ\bullet\\\circ\bullet\\\bullet\circ}}}{dt} = \underset{\textsc{ATPio}}{\nu_f}\big(\mathrm{T_I}^{\substack{\circ\bullet\\\circ\bullet\\\bullet\circ}}\big) + \underset{\textsc{AKo}}{\nu_r}\big(\mathrm{D_O}^{\substack{\bullet\\\bullet\\\circ}}\mathrm{D_O}^{\substack{\circ\\\circ\\\bullet}}\big) + \underset{\textsc{CKo}}{\nu_r}\big(\mathrm{D_O}^{\substack{\circ\\\circ\\\bullet}}\mathrm{C_O}^{\substack{\bullet\\\bullet\\\circ}}\big) - \underset{\textsc{AKo}}{\nu_f}\big(\mathrm{T_O}^{\substack{\circ\bullet\\\circ\bullet\\\bullet\circ}}\big) - \underset{\textsc{ATPoe}}{\nu_f}\big(\mathrm{T_O}^{\substack{\circ\bullet\\\circ\bullet\\\bullet\circ}}\big) - \underset{\textsc{CKo}}{\nu_f}\big(\mathrm{T_O}^{\substack{\circ\bullet\\\circ\bullet\\\bullet\circ}}\big) - \underset{\textsc{ATPio}}{\nu_r}\big(\mathrm{T_O}^{\substack{\circ\bullet\\\circ\bullet\\\bullet\circ}}\big)$$

$$\frac{d\mathrm{T_O}^{\substack{\circ\bullet\\\circ\bullet\\\bullet\bullet}}}{dt} = \underset{\textsc{ATPio}}{\nu_f}\big(\mathrm{T_I}^{\substack{\circ\bullet\\\circ\bullet\\\bullet\bullet}}\big) + \underset{\textsc{AKo}}{\nu_r}\big(\mathrm{D_O}^{\substack{\bullet\\\bullet\\\bullet}}\mathrm{D_O}^{\substack{\circ\\\circ\\\bullet}}\big) + \underset{\textsc{CKo}}{\nu_r}\big(\mathrm{D_O}^{\substack{\circ\\\circ\\\bullet}}\mathrm{C_O}^{\substack{\bullet\\\bullet\\\bullet}}\big) - \underset{\textsc{AKo}}{\nu_f}\big(\mathrm{T_O}^{\substack{\circ\bullet\\\circ\bullet\\\bullet\bullet}}\big) - \underset{\textsc{ATPoe}}{\nu_f}\big(\mathrm{T_O}^{\substack{\circ\bullet\\\circ\bullet\\\bullet\bullet}}\big) - \underset{\textsc{CKo}}{\nu_f}\big(\mathrm{T_O}^{\substack{\circ\bullet\\\circ\bullet\\\bullet\bullet}}\big) - \underset{\textsc{ATPio}}{\nu_r}\big(\mathrm{T_O}^{\substack{\circ\bullet\\\circ\bullet\\\bullet\bullet}}\big)$$

$$\frac{d\mathrm{T_O}^{\substack{\circ\circ\\\bullet\circ\\\circ\circ}}}{dt} = \underset{\textsc{ATPio}}{\nu_f}\big(\mathrm{T_I}^{\substack{\circ\circ\\\bullet\circ\\\circ\circ}}\big) + \underset{\textsc{AKo}}{\nu_r}\big(\mathrm{D_O}^{\substack{\circ\\\bullet\\\circ}}\mathrm{D_O}^{\substack{\circ\\\circ\\\circ}}\big) + \underset{\textsc{CKo}}{\nu_r}\big(\mathrm{D_O}^{\substack{\circ\\\bullet\\\circ}}\mathrm{C_O}^{\substack{\circ\\\circ\\\circ}}\big) - \underset{\textsc{AKo}}{\nu_f}\big(\mathrm{T_O}^{\substack{\circ\circ\\\bullet\circ\\\circ\circ}}\big) - \underset{\textsc{ATPoe}}{\nu_f}\big(\mathrm{T_O}^{\substack{\circ\circ\\\bullet\circ\\\circ\circ}}\big) - \underset{\textsc{CKo}}{\nu_f}\big(\mathrm{T_O}^{\substack{\circ\circ\\\bullet\circ\\\circ\circ}}\big) - \underset{\textsc{ATPio}}{\nu_r}\big(\mathrm{T_O}^{\substack{\circ\circ\\\bullet\circ\\\circ\circ}}\big)$$

$$\frac{d\mathrm{T_O}^{\substack{\circ\circ\\\bullet\circ\\\circ\bullet}}}{dt} = \underset{\textsc{ATPio}}{\nu_f}\big(\mathrm{T_I}^{\substack{\circ\circ\\\bullet\circ\\\circ\bullet}}\big) + \underset{\textsc{AKo}}{\nu_r}\big(\mathrm{D_O}^{\substack{\circ\\\bullet\\\circ}}\mathrm{D_O}^{\substack{\circ\\\circ\\\bullet}}\big) + \underset{\textsc{CKo}}{\nu_r}\big(\mathrm{D_O}^{\substack{\circ\\\bullet\\\circ}}\mathrm{C_O}^{\substack{\circ\\\circ\\\bullet}}\big) - \underset{\textsc{AKo}}{\nu_f}\big(\mathrm{T_O}^{\substack{\circ\circ\\\bullet\circ\\\circ\bullet}}\big) - \underset{\textsc{ATPoe}}{\nu_f}\big(\mathrm{T_O}^{\substack{\circ\circ\\\bullet\circ\\\circ\bullet}}\big) - \underset{\textsc{CKo}}{\nu_f}\big(\mathrm{T_O}^{\substack{\circ\circ\\\bullet\circ\\\circ\bullet}}\big) - \underset{\textsc{ATPio}}{\nu_r}\big(\mathrm{T_O}^{\substack{\circ\circ\\\bullet\circ\\\circ\bullet}}\big)$$

$$\frac{d\mathrm{T_O}^{\substack{\circ\circ\\\bullet\bullet\\\circ\circ}}}{dt} = \underset{\textsc{ATPio}}{\nu_f}\big(\mathrm{T_I}^{\substack{\circ\circ\\\bullet\bullet\\\circ\circ}}\big) + \underset{\textsc{AKo}}{\nu_r}\big(\mathrm{D_O}^{\substack{\circ\\\bullet\\\circ}}\mathrm{D_O}^{\substack{\circ\\\bullet\\\circ}}\big) + \underset{\textsc{CKo}}{\nu_r}\big(\mathrm{D_O}^{\substack{\circ\\\bullet\\\circ}}\mathrm{C_O}^{\substack{\circ\\\bullet\\\circ}}\big) - \underset{\textsc{AKo}}{\nu_f}\big(\mathrm{T_O}^{\substack{\circ\circ\\\bullet\bullet\\\circ\circ}}\big) - \underset{\textsc{ATPoe}}{\nu_f}\big(\mathrm{T_O}^{\substack{\circ\circ\\\bullet\bullet\\\circ\circ}}\big) - \underset{\textsc{CKo}}{\nu_f}\big(\mathrm{T_O}^{\substack{\circ\circ\\\bullet\bullet\\\circ\circ}}\big) - \underset{\textsc{ATPio}}{\nu_r}\big(\mathrm{T_O}^{\substack{\circ\circ\\\bullet\bullet\\\circ\circ}}\big)$$

$$\frac{d\mathrm{T}_{\mathrm{O}}^{\substack{\circ\circ\\\bullet\bullet\\\circ\bullet}}}{dt} = \underset{\text{ATPIO}}{\nu_f}(\mathrm{T}_{\mathrm{I}}^{\substack{\circ\circ\\\bullet\bullet\\\circ\bullet}}) + \underset{\text{AKO}}{\nu_r}(\mathrm{D}_{\mathrm{O}}^{\substack{\circ\\\bullet\\\bullet}}\mathrm{D}_{\mathrm{O}}^{\substack{\circ\\\bullet\\\circ}}) + \underset{\text{CKO}}{\nu_r}(\mathrm{D}_{\mathrm{O}}^{\substack{\circ\\\bullet\\\circ}}\mathrm{C}_{\mathrm{O}}^{\substack{\circ\\\bullet\\\bullet}}) - \underset{\text{AKO}}{\nu_f}(\mathrm{T}_{\mathrm{O}}^{\substack{\circ\circ\\\bullet\bullet\\\circ\bullet}}) - \underset{\text{ATPOE}}{\nu_f}(\mathrm{T}_{\mathrm{O}}^{\substack{\circ\circ\\\bullet\bullet\\\circ\bullet}}) - \underset{\text{CKO}}{\nu_f}(\mathrm{T}_{\mathrm{O}}^{\substack{\circ\circ\\\bullet\bullet\\\circ\bullet}}) - \underset{\text{ATPIO}}{\nu_r}(\mathrm{T}_{\mathrm{O}}^{\substack{\circ\circ\\\bullet\bullet\\\circ\bullet}})$$

$$\frac{d\mathrm{T}_{\mathrm{O}}^{\substack{\circ\bullet\\\bullet\circ\\\circ\circ}}}{dt} = \underset{\text{ATPIO}}{\nu_f}(\mathrm{T}_{\mathrm{I}}^{\substack{\circ\bullet\\\bullet\circ\\\circ\circ}}) + \underset{\text{AKO}}{\nu_r}(\mathrm{D}_{\mathrm{O}}^{\substack{\bullet\\\circ\\\circ}}\mathrm{D}_{\mathrm{O}}^{\substack{\circ\\\bullet\\\circ}}) + \underset{\text{CKO}}{\nu_r}(\mathrm{D}_{\mathrm{O}}^{\substack{\circ\\\bullet\\\circ}}\mathrm{C}_{\mathrm{O}}^{\substack{\bullet\\\circ\\\circ}}) - \underset{\text{AKO}}{\nu_f}(\mathrm{T}_{\mathrm{O}}^{\substack{\circ\bullet\\\bullet\circ\\\circ\circ}}) - \underset{\text{ATPOE}}{\nu_f}(\mathrm{T}_{\mathrm{O}}^{\substack{\circ\bullet\\\bullet\circ\\\circ\circ}}) - \underset{\text{CKO}}{\nu_f}(\mathrm{T}_{\mathrm{O}}^{\substack{\circ\bullet\\\bullet\circ\\\circ\circ}}) - \underset{\text{ATPIO}}{\nu_r}(\mathrm{T}_{\mathrm{O}}^{\substack{\circ\bullet\\\bullet\circ\\\circ\circ}})$$

$$\frac{d\mathrm{T}_{\mathrm{O}}^{\substack{\circ\bullet\\\bullet\circ\\\circ\bullet}}}{dt} = \underset{\text{ATPIO}}{\nu_f}(\mathrm{T}_{\mathrm{I}}^{\substack{\circ\bullet\\\bullet\circ\\\circ\bullet}}) + \underset{\text{AKO}}{\nu_r}(\mathrm{D}_{\mathrm{O}}^{\substack{\bullet\\\circ\\\bullet}}\mathrm{D}_{\mathrm{O}}^{\substack{\circ\\\bullet\\\circ}}) + \underset{\text{CKO}}{\nu_r}(\mathrm{D}_{\mathrm{O}}^{\substack{\circ\\\bullet\\\circ}}\mathrm{C}_{\mathrm{O}}^{\substack{\bullet\\\circ\\\bullet}}) - \underset{\text{AKO}}{\nu_f}(\mathrm{T}_{\mathrm{O}}^{\substack{\circ\bullet\\\bullet\circ\\\circ\bullet}}) - \underset{\text{ATPOE}}{\nu_f}(\mathrm{T}_{\mathrm{O}}^{\substack{\circ\bullet\\\bullet\circ\\\circ\bullet}}) - \underset{\text{CKO}}{\nu_f}(\mathrm{T}_{\mathrm{O}}^{\substack{\circ\bullet\\\bullet\circ\\\circ\bullet}}) - \underset{\text{ATPIO}}{\nu_r}(\mathrm{T}_{\mathrm{O}}^{\substack{\circ\bullet\\\bullet\circ\\\circ\bullet}})$$

$$\frac{d\mathrm{T}_{\mathrm{O}}^{\substack{\circ\bullet\\\bullet\bullet\\\circ\circ}}}{dt} = \underset{\text{ATPIO}}{\nu_f}(\mathrm{T}_{\mathrm{I}}^{\substack{\circ\bullet\\\bullet\bullet\\\circ\circ}}) + \underset{\text{AKO}}{\nu_r}(\mathrm{D}_{\mathrm{O}}^{\substack{\bullet\\\bullet\\\circ}}\mathrm{D}_{\mathrm{O}}^{\substack{\circ\\\bullet\\\circ}}) + \underset{\text{CKO}}{\nu_r}(\mathrm{D}_{\mathrm{O}}^{\substack{\circ\\\bullet\\\circ}}\mathrm{C}_{\mathrm{O}}^{\substack{\bullet\\\bullet\\\circ}}) - \underset{\text{AKO}}{\nu_f}(\mathrm{T}_{\mathrm{O}}^{\substack{\circ\bullet\\\bullet\bullet\\\circ\circ}}) - \underset{\text{ATPOE}}{\nu_f}(\mathrm{T}_{\mathrm{O}}^{\substack{\circ\bullet\\\bullet\bullet\\\circ\circ}}) - \underset{\text{CKO}}{\nu_f}(\mathrm{T}_{\mathrm{O}}^{\substack{\circ\bullet\\\bullet\bullet\\\circ\circ}}) - \underset{\text{ATPIO}}{\nu_r}(\mathrm{T}_{\mathrm{O}}^{\substack{\circ\bullet\\\bullet\bullet\\\circ\circ}})$$

$$\frac{d\mathrm{T}_{\mathrm{O}}^{\substack{\circ\bullet\\\bullet\bullet\\\circ\bullet}}}{dt} = \underset{\text{ATPIO}}{\nu_f}(\mathrm{T}_{\mathrm{I}}^{\substack{\circ\bullet\\\bullet\bullet\\\circ\bullet}}) + \underset{\text{AKO}}{\nu_r}(\mathrm{D}_{\mathrm{O}}^{\substack{\bullet\\\bullet\\\bullet}}\mathrm{D}_{\mathrm{O}}^{\substack{\circ\\\bullet\\\circ}}) + \underset{\text{CKO}}{\nu_r}(\mathrm{D}_{\mathrm{O}}^{\substack{\circ\\\bullet\\\circ}}\mathrm{C}_{\mathrm{O}}^{\substack{\bullet\\\bullet\\\bullet}}) - \underset{\text{AKO}}{\nu_f}(\mathrm{T}_{\mathrm{O}}^{\substack{\circ\bullet\\\bullet\bullet\\\circ\bullet}}) - \underset{\text{ATPOE}}{\nu_f}(\mathrm{T}_{\mathrm{O}}^{\substack{\circ\bullet\\\bullet\bullet\\\circ\bullet}}) - \underset{\text{CKO}}{\nu_f}(\mathrm{T}_{\mathrm{O}}^{\substack{\circ\bullet\\\bullet\bullet\\\circ\bullet}}) - \underset{\text{ATPIO}}{\nu_r}(\mathrm{T}_{\mathrm{O}}^{\substack{\circ\bullet\\\bullet\bullet\\\circ\bullet}})$$

$$\frac{d\mathrm{T}_{\mathrm{O}}^{\substack{\circ\circ\\\bullet\circ\\\bullet\circ}}}{dt} = \underset{\text{ATPIO}}{\nu_f}(\mathrm{T}_{\mathrm{I}}^{\substack{\circ\circ\\\bullet\circ\\\bullet\circ}}) + \underset{\text{AKO}}{\nu_r}(\mathrm{D}_{\mathrm{O}}^{\substack{\circ\\\bullet\\\bullet}}\mathrm{D}_{\mathrm{O}}^{\substack{\circ\\\circ\\\circ}}) + \underset{\text{CKO}}{\nu_r}(\mathrm{D}_{\mathrm{O}}^{\substack{\circ\\\bullet\\\bullet}}\mathrm{C}_{\mathrm{O}}^{\substack{\circ\\\circ\\\circ}}) - \underset{\text{AKO}}{\nu_f}(\mathrm{T}_{\mathrm{O}}^{\substack{\circ\circ\\\bullet\circ\\\bullet\circ}}) - \underset{\text{ATPOE}}{\nu_f}(\mathrm{T}_{\mathrm{O}}^{\substack{\circ\circ\\\bullet\circ\\\bullet\circ}}) - \underset{\text{CKO}}{\nu_f}(\mathrm{T}_{\mathrm{O}}^{\substack{\circ\circ\\\bullet\circ\\\bullet\circ}}) - \underset{\text{ATPIO}}{\nu_r}(\mathrm{T}_{\mathrm{O}}^{\substack{\circ\circ\\\bullet\circ\\\bullet\circ}})$$

$$\frac{d\mathrm{T}_{\mathrm{O}}^{\substack{\circ\circ\\\bullet\circ\\\bullet\bullet}}}{dt} = \underset{\text{ATPIO}}{\nu_f}(\mathrm{T}_{\mathrm{I}}^{\substack{\circ\circ\\\bullet\circ\\\bullet\bullet}}) + \underset{\text{AKO}}{\nu_r}(\mathrm{D}_{\mathrm{O}}^{\substack{\circ\\\bullet\\\bullet}}\mathrm{D}_{\mathrm{O}}^{\substack{\circ\\\circ\\\bullet}}) + \underset{\text{CKO}}{\nu_r}(\mathrm{D}_{\mathrm{O}}^{\substack{\circ\\\bullet\\\bullet}}\mathrm{C}_{\mathrm{O}}^{\substack{\circ\\\circ\\\bullet}}) - \underset{\text{AKO}}{\nu_f}(\mathrm{T}_{\mathrm{O}}^{\substack{\circ\circ\\\bullet\circ\\\bullet\bullet}}) - \underset{\text{ATPOE}}{\nu_f}(\mathrm{T}_{\mathrm{O}}^{\substack{\circ\circ\\\bullet\circ\\\bullet\bullet}}) - \underset{\text{CKO}}{\nu_f}(\mathrm{T}_{\mathrm{O}}^{\substack{\circ\circ\\\bullet\circ\\\bullet\bullet}}) - \underset{\text{ATPIO}}{\nu_r}(\mathrm{T}_{\mathrm{O}}^{\substack{\circ\circ\\\bullet\circ\\\bullet\bullet}})$$

$$\frac{d\mathrm{T}_{\mathrm{O}}^{\substack{\circ\circ\\\bullet\bullet\\\bullet\circ}}}{dt} = \underset{\text{ATPIO}}{\nu_f}(\mathrm{T}_{\mathrm{I}}^{\substack{\circ\circ\\\bullet\bullet\\\bullet\circ}}) + \underset{\text{AKO}}{\nu_r}(\mathrm{D}_{\mathrm{O}}^{\substack{\circ\\\bullet\\\bullet}}\mathrm{D}_{\mathrm{O}}^{\substack{\circ\\\bullet\\\circ}}) + \underset{\text{CKO}}{\nu_r}(\mathrm{D}_{\mathrm{O}}^{\substack{\circ\\\bullet\\\bullet}}\mathrm{C}_{\mathrm{O}}^{\substack{\circ\\\bullet\\\circ}}) - \underset{\text{AKO}}{\nu_f}(\mathrm{T}_{\mathrm{O}}^{\substack{\circ\circ\\\bullet\bullet\\\bullet\circ}}) - \underset{\text{ATPOE}}{\nu_f}(\mathrm{T}_{\mathrm{O}}^{\substack{\circ\circ\\\bullet\bullet\\\bullet\circ}}) - \underset{\text{CKO}}{\nu_f}(\mathrm{T}_{\mathrm{O}}^{\substack{\circ\circ\\\bullet\bullet\\\bullet\circ}}) - \underset{\text{ATPIO}}{\nu_r}(\mathrm{T}_{\mathrm{O}}^{\substack{\circ\circ\\\bullet\bullet\\\bullet\circ}})$$

$$\frac{d\mathrm{T}_{\mathrm{O}}^{\substack{\circ\circ\\\bullet\bullet\\\bullet\bullet}}}{dt} = \underset{\text{ATPIO}}{\nu_f}(\mathrm{T}_{\mathrm{I}}^{\substack{\circ\circ\\\bullet\bullet\\\bullet\bullet}}) + \underset{\text{AKO}}{\nu_r}(\mathrm{D}_{\mathrm{O}}^{\substack{\circ\\\bullet\\\bullet}}\mathrm{D}_{\mathrm{O}}^{\substack{\circ\\\bullet\\\bullet}}) + \underset{\text{CKO}}{\nu_r}(\mathrm{D}_{\mathrm{O}}^{\substack{\circ\\\bullet\\\bullet}}\mathrm{C}_{\mathrm{O}}^{\substack{\circ\\\bullet\\\bullet}}) - \underset{\text{AKO}}{\nu_f}(\mathrm{T}_{\mathrm{O}}^{\substack{\circ\circ\\\bullet\bullet\\\bullet\bullet}}) - \underset{\text{ATPOE}}{\nu_f}(\mathrm{T}_{\mathrm{O}}^{\substack{\circ\circ\\\bullet\bullet\\\bullet\bullet}}) - \underset{\text{CKO}}{\nu_f}(\mathrm{T}_{\mathrm{O}}^{\substack{\circ\circ\\\bullet\bullet\\\bullet\bullet}}) - \underset{\text{ATPIO}}{\nu_r}(\mathrm{T}_{\mathrm{O}}^{\substack{\circ\circ\\\bullet\bullet\\\bullet\bullet}})$$

$$\frac{d\mathrm{T}_{\mathrm{O}}^{\substack{\circ\bullet\\\bullet\circ\\\bullet\circ}}}{dt} = \underset{\text{ATPIO}}{\nu_f}(\mathrm{T}_{\mathrm{I}}^{\substack{\circ\bullet\\\bullet\circ\\\bullet\circ}}) + \underset{\text{AKO}}{\nu_r}(\mathrm{D}_{\mathrm{O}}^{\substack{\bullet\\\circ\\\circ}}\mathrm{D}_{\mathrm{O}}^{\substack{\circ\\\bullet\\\bullet}}) + \underset{\text{CKO}}{\nu_r}(\mathrm{D}_{\mathrm{O}}^{\substack{\circ\\\bullet\\\bullet}}\mathrm{C}_{\mathrm{O}}^{\substack{\bullet\\\circ\\\circ}}) - \underset{\text{AKO}}{\nu_f}(\mathrm{T}_{\mathrm{O}}^{\substack{\circ\bullet\\\bullet\circ\\\bullet\circ}}) - \underset{\text{ATPOE}}{\nu_f}(\mathrm{T}_{\mathrm{O}}^{\substack{\circ\bullet\\\bullet\circ\\\bullet\circ}}) - \underset{\text{CKO}}{\nu_f}(\mathrm{T}_{\mathrm{O}}^{\substack{\circ\bullet\\\bullet\circ\\\bullet\circ}}) - \underset{\text{ATPIO}}{\nu_r}(\mathrm{T}_{\mathrm{O}}^{\substack{\circ\bullet\\\bullet\circ\\\bullet\circ}})$$

$$\frac{d\mathrm{T}_{\mathrm{O}}^{\substack{\circ\bullet\\\bullet\circ\\\bullet\bullet}}}{dt} = \underset{\text{ATPIO}}{\nu_f}(\mathrm{T}_{\mathrm{I}}^{\substack{\circ\bullet\\\bullet\circ\\\bullet\bullet}}) + \underset{\text{AKO}}{\nu_r}(\mathrm{D}_{\mathrm{O}}^{\substack{\bullet\\\circ\\\bullet}}\mathrm{D}_{\mathrm{O}}^{\substack{\circ\\\bullet\\\bullet}}) + \underset{\text{CKO}}{\nu_r}(\mathrm{D}_{\mathrm{O}}^{\substack{\circ\\\bullet\\\bullet}}\mathrm{C}_{\mathrm{O}}^{\substack{\bullet\\\circ\\\bullet}}) - \underset{\text{AKO}}{\nu_f}(\mathrm{T}_{\mathrm{O}}^{\substack{\circ\bullet\\\bullet\circ\\\bullet\bullet}}) - \underset{\text{ATPOE}}{\nu_f}(\mathrm{T}_{\mathrm{O}}^{\substack{\circ\bullet\\\bullet\circ\\\bullet\bullet}}) - \underset{\text{CKO}}{\nu_f}(\mathrm{T}_{\mathrm{O}}^{\substack{\circ\bullet\\\bullet\circ\\\bullet\bullet}}) - \underset{\text{ATPIO}}{\nu_r}(\mathrm{T}_{\mathrm{O}}^{\substack{\circ\bullet\\\bullet\circ\\\bullet\bullet}})$$

$$\frac{d\mathrm{T}_{\mathrm{O}}^{\substack{\circ\bullet\\\bullet\bullet\\\bullet\circ}}}{dt} = \underset{\text{ATPIO}}{\nu_f}(\mathrm{T}_{\mathrm{I}}^{\substack{\circ\bullet\\\bullet\bullet\\\bullet\circ}}) + \underset{\text{AKO}}{\nu_r}(\mathrm{D}_{\mathrm{O}}^{\substack{\bullet\\\bullet\\\circ}}\mathrm{D}_{\mathrm{O}}^{\substack{\circ\\\bullet\\\bullet}}) + \underset{\text{CKO}}{\nu_r}(\mathrm{D}_{\mathrm{O}}^{\substack{\circ\\\bullet\\\bullet}}\mathrm{C}_{\mathrm{O}}^{\substack{\bullet\\\bullet\\\circ}}) - \underset{\text{AKO}}{\nu_f}(\mathrm{T}_{\mathrm{O}}^{\substack{\circ\bullet\\\bullet\bullet\\\bullet\circ}}) - \underset{\text{ATPOE}}{\nu_f}(\mathrm{T}_{\mathrm{O}}^{\substack{\circ\bullet\\\bullet\bullet\\\bullet\circ}}) - \underset{\text{CKO}}{\nu_f}(\mathrm{T}_{\mathrm{O}}^{\substack{\circ\bullet\\\bullet\bullet\\\bullet\circ}}) - \underset{\text{ATPIO}}{\nu_r}(\mathrm{T}_{\mathrm{O}}^{\substack{\circ\bullet\\\bullet\bullet\\\bullet\circ}})$$

$$\frac{d\mathrm{T}_{\mathrm{O}}^{\substack{\circ\bullet\\\bullet\bullet\\\bullet\bullet}}}{dt} = \underset{\text{ATPIO}}{\nu_f}(\mathrm{T}_{\mathrm{I}}^{\substack{\circ\bullet\\\bullet\bullet\\\bullet\bullet}}) + \underset{\text{AKO}}{\nu_r}(\mathrm{D}_{\mathrm{O}}^{\substack{\bullet\\\bullet\\\bullet}}\mathrm{D}_{\mathrm{O}}^{\substack{\circ\\\bullet\\\bullet}}) + \underset{\text{CKO}}{\nu_r}(\mathrm{D}_{\mathrm{O}}^{\substack{\circ\\\bullet\\\bullet}}\mathrm{C}_{\mathrm{O}}^{\substack{\bullet\\\bullet\\\bullet}}) - \underset{\text{AKO}}{\nu_f}(\mathrm{T}_{\mathrm{O}}^{\substack{\circ\bullet\\\bullet\bullet\\\bullet\bullet}}) - \underset{\text{ATPOE}}{\nu_f}(\mathrm{T}_{\mathrm{O}}^{\substack{\circ\bullet\\\bullet\bullet\\\bullet\bullet}}) - \underset{\text{CKO}}{\nu_f}(\mathrm{T}_{\mathrm{O}}^{\substack{\circ\bullet\\\bullet\bullet\\\bullet\bullet}}) - \underset{\text{ATPIO}}{\nu_r}(\mathrm{T}_{\mathrm{O}}^{\substack{\circ\bullet\\\bullet\bullet\\\bullet\bullet}})$$

$$\frac{d\mathrm{T}_{\mathrm{O}}^{\substack{\bullet\circ\\\circ\circ\\\circ\circ}}}{dt} = \underset{\text{ATPIO}}{\nu_f}(\mathrm{T}_{\mathrm{I}}^{\substack{\bullet\circ\\\circ\circ\\\circ\circ}}) + \underset{\text{AKO}}{\nu_r}(\mathrm{D}_{\mathrm{O}}^{\substack{\bullet\\\circ\\\circ}}\mathrm{D}_{\mathrm{O}}^{\substack{\circ\\\circ\\\circ}}) + \underset{\text{CKO}}{\nu_r}(\mathrm{D}_{\mathrm{O}}^{\substack{\bullet\\\circ\\\circ}}\mathrm{C}_{\mathrm{O}}^{\substack{\circ\\\circ\\\circ}}) - \underset{\text{AKO}}{\nu_f}(\mathrm{T}_{\mathrm{O}}^{\substack{\bullet\circ\\\circ\circ\\\circ\circ}}) - \underset{\text{ATPOE}}{\nu_f}(\mathrm{T}_{\mathrm{O}}^{\substack{\bullet\circ\\\circ\circ\\\circ\circ}}) - \underset{\text{CKO}}{\nu_f}(\mathrm{T}_{\mathrm{O}}^{\substack{\bullet\circ\\\circ\circ\\\circ\circ}}) - \underset{\text{ATPIO}}{\nu_r}(\mathrm{T}_{\mathrm{O}}^{\substack{\bullet\circ\\\circ\circ\\\circ\circ}})$$

$$\frac{d\mathrm{T}_{\mathrm{O}}^{\substack{\bullet\circ\\\circ\circ\\\circ\bullet}}}{dt} = \underset{\text{ATPIO}}{\nu_f}(\mathrm{T}_{\mathrm{I}}^{\substack{\bullet\circ\\\circ\circ\\\circ\bullet}}) + \underset{\text{AKO}}{\nu_r}(\mathrm{D}_{\mathrm{O}}^{\substack{\bullet\\\circ\\\circ}}\mathrm{D}_{\mathrm{O}}^{\substack{\circ\\\circ\\\bullet}}) + \underset{\text{CKO}}{\nu_r}(\mathrm{D}_{\mathrm{O}}^{\substack{\bullet\\\circ\\\circ}}\mathrm{C}_{\mathrm{O}}^{\substack{\circ\\\circ\\\bullet}}) - \underset{\text{AKO}}{\nu_f}(\mathrm{T}_{\mathrm{O}}^{\substack{\bullet\circ\\\circ\circ\\\circ\bullet}}) - \underset{\text{ATPOE}}{\nu_f}(\mathrm{T}_{\mathrm{O}}^{\substack{\bullet\circ\\\circ\circ\\\circ\bullet}}) - \underset{\text{CKO}}{\nu_f}(\mathrm{T}_{\mathrm{O}}^{\substack{\bullet\circ\\\circ\circ\\\circ\bullet}}) - \underset{\text{ATPIO}}{\nu_r}(\mathrm{T}_{\mathrm{O}}^{\substack{\bullet\circ\\\circ\circ\\\circ\bullet}})$$

$$\frac{d\mathrm{T}_{\mathrm{O}}^{\substack{\bullet\circ\\\circ\bullet\\\circ\circ}}}{dt} = \underset{\text{ATPIO}}{\nu_f}(\mathrm{T}_{\mathrm{I}}^{\substack{\bullet\circ\\\circ\bullet\\\circ\circ}}) + \underset{\text{AKO}}{\nu_r}(\mathrm{D}_{\mathrm{O}}^{\substack{\bullet\\\circ\\\circ}}\mathrm{D}_{\mathrm{O}}^{\substack{\circ\\\bullet\\\circ}}) + \underset{\text{CKO}}{\nu_r}(\mathrm{D}_{\mathrm{O}}^{\substack{\bullet\\\circ\\\circ}}\mathrm{C}_{\mathrm{O}}^{\substack{\circ\\\bullet\\\circ}}) - \underset{\text{AKO}}{\nu_f}(\mathrm{T}_{\mathrm{O}}^{\substack{\bullet\circ\\\circ\bullet\\\circ\circ}}) - \underset{\text{ATPOE}}{\nu_f}(\mathrm{T}_{\mathrm{O}}^{\substack{\bullet\circ\\\circ\bullet\\\circ\circ}}) - \underset{\text{CKO}}{\nu_f}(\mathrm{T}_{\mathrm{O}}^{\substack{\bullet\circ\\\circ\bullet\\\circ\circ}}) - \underset{\text{ATPIO}}{\nu_r}(\mathrm{T}_{\mathrm{O}}^{\substack{\bullet\circ\\\circ\bullet\\\circ\circ}})$$

$$\frac{d\mathrm{T}_{\mathrm{O}}^{\substack{\bullet\circ\\\circ\bullet\\\circ\bullet}}}{dt} = \underset{\text{ATPIO}}{\nu_f}(\mathrm{T}_{\mathrm{I}}^{\substack{\bullet\circ\\\circ\bullet\\\circ\bullet}}) + \underset{\text{AKO}}{\nu_r}(\mathrm{D}_{\mathrm{O}}^{\substack{\bullet\\\circ\\\circ}}\mathrm{D}_{\mathrm{O}}^{\substack{\circ\\\bullet\\\bullet}}) + \underset{\text{CKO}}{\nu_r}(\mathrm{D}_{\mathrm{O}}^{\substack{\bullet\\\circ\\\circ}}\mathrm{C}_{\mathrm{O}}^{\substack{\circ\\\bullet\\\bullet}}) - \underset{\text{AKO}}{\nu_f}(\mathrm{T}_{\mathrm{O}}^{\substack{\bullet\circ\\\circ\bullet\\\circ\bullet}}) - \underset{\text{ATPOE}}{\nu_f}(\mathrm{T}_{\mathrm{O}}^{\substack{\bullet\circ\\\circ\bullet\\\circ\bullet}}) - \underset{\text{CKO}}{\nu_f}(\mathrm{T}_{\mathrm{O}}^{\substack{\bullet\circ\\\circ\bullet\\\circ\bullet}}) - \underset{\text{ATPIO}}{\nu_r}(\mathrm{T}_{\mathrm{O}}^{\substack{\bullet\circ\\\circ\bullet\\\circ\bullet}})$$

$$\frac{d\mathrm{T}_{\mathrm{O}}^{\substack{\bullet\bullet\\\circ\circ\\\circ\circ}}}{dt} = \underset{\text{ATPIO}}{\nu_f}(\mathrm{T}_{\mathrm{I}}^{\substack{\bullet\bullet\\\circ\circ\\\circ\circ}}) + \underset{\text{AKO}}{\nu_r}(\mathrm{D}_{\mathrm{O}}^{\substack{\bullet\\\circ\\\circ}}\mathrm{D}_{\mathrm{O}}^{\substack{\bullet\\\circ\\\circ}}) + \underset{\text{CKO}}{\nu_r}(\mathrm{D}_{\mathrm{O}}^{\substack{\bullet\\\circ\\\circ}}\mathrm{C}_{\mathrm{O}}^{\substack{\bullet\\\circ\\\circ}}) - \underset{\text{AKO}}{\nu_f}(\mathrm{T}_{\mathrm{O}}^{\substack{\bullet\bullet\\\circ\circ\\\circ\circ}}) - \underset{\text{ATPOE}}{\nu_f}(\mathrm{T}_{\mathrm{O}}^{\substack{\bullet\bullet\\\circ\circ\\\circ\circ}}) - \underset{\text{CKO}}{\nu_f}(\mathrm{T}_{\mathrm{O}}^{\substack{\bullet\bullet\\\circ\circ\\\circ\circ}}) - \underset{\text{ATPIO}}{\nu_r}(\mathrm{T}_{\mathrm{O}}^{\substack{\bullet\bullet\\\circ\circ\\\circ\circ}})$$

$$\frac{d\mathrm{T}_{\mathrm{O}}^{\substack{\bullet\bullet\\\circ\circ\\\circ\bullet}}}{dt} = \underset{\text{ATPIO}}{\nu_f}(\mathrm{T}_{\mathrm{I}}^{\substack{\bullet\bullet\\\circ\circ\\\circ\bullet}}) + \underset{\text{AKO}}{\nu_r}(\mathrm{D}_{\mathrm{O}}^{\substack{\bullet\\\circ\\\bullet}}\mathrm{D}_{\mathrm{O}}^{\substack{\bullet\\\circ\\\circ}}) + \underset{\text{CKO}}{\nu_r}(\mathrm{D}_{\mathrm{O}}^{\substack{\bullet\\\circ\\\circ}}\mathrm{C}_{\mathrm{O}}^{\substack{\bullet\\\circ\\\bullet}}) - \underset{\text{AKO}}{\nu_f}(\mathrm{T}_{\mathrm{O}}^{\substack{\bullet\bullet\\\circ\circ\\\circ\bullet}}) - \underset{\text{ATPOE}}{\nu_f}(\mathrm{T}_{\mathrm{O}}^{\substack{\bullet\bullet\\\circ\circ\\\circ\bullet}}) - \underset{\text{CKO}}{\nu_f}(\mathrm{T}_{\mathrm{O}}^{\substack{\bullet\bullet\\\circ\circ\\\circ\bullet}}) - \underset{\text{ATPIO}}{\nu_r}(\mathrm{T}_{\mathrm{O}}^{\substack{\bullet\bullet\\\circ\circ\\\circ\bullet}})$$

$$\frac{d\mathrm{T}_{\mathrm{O}}^{\substack{\bullet\bullet\\\circ\bullet\\\circ\circ}}}{dt} = \underset{\text{ATPIO}}{\nu_f}(\mathrm{T}_{\mathrm{I}}^{\substack{\bullet\bullet\\\circ\bullet\\\circ\circ}}) + \underset{\text{AKO}}{\nu_r}(\mathrm{D}_{\mathrm{O}}^{\substack{\bullet\\\bullet\\\circ}}\mathrm{D}_{\mathrm{O}}^{\substack{\bullet\\\circ\\\circ}}) + \underset{\text{CKO}}{\nu_r}(\mathrm{D}_{\mathrm{O}}^{\substack{\bullet\\\circ\\\circ}}\mathrm{C}_{\mathrm{O}}^{\substack{\bullet\\\bullet\\\circ}}) - \underset{\text{AKO}}{\nu_f}(\mathrm{T}_{\mathrm{O}}^{\substack{\bullet\bullet\\\circ\bullet\\\circ\circ}}) - \underset{\text{ATPOE}}{\nu_f}(\mathrm{T}_{\mathrm{O}}^{\substack{\bullet\bullet\\\circ\bullet\\\circ\circ}}) - \underset{\text{CKO}}{\nu_f}(\mathrm{T}_{\mathrm{O}}^{\substack{\bullet\bullet\\\circ\bullet\\\circ\circ}}) - \underset{\text{ATPIO}}{\nu_r}(\mathrm{T}_{\mathrm{O}}^{\substack{\bullet\bullet\\\circ\bullet\\\circ\circ}})$$

$$\frac{d\mathrm{T}_{\mathrm{O}}^{\substack{\bullet\bullet\\\circ\bullet\\\circ\bullet}}}{dt} = \underset{\text{ATPIO}}{\nu_f}(\mathrm{T}_{\mathrm{I}}^{\substack{\bullet\bullet\\\circ\bullet\\\circ\bullet}}) + \underset{\text{AKO}}{\nu_r}(\mathrm{D}_{\mathrm{O}}^{\substack{\bullet\\\bullet\\\bullet}}\mathrm{D}_{\mathrm{O}}^{\substack{\bullet\\\circ\\\circ}}) + \underset{\text{CKO}}{\nu_r}(\mathrm{D}_{\mathrm{O}}^{\substack{\bullet\\\circ\\\circ}}\mathrm{C}_{\mathrm{O}}^{\substack{\bullet\\\bullet\\\bullet}}) - \underset{\text{AKO}}{\nu_f}(\mathrm{T}_{\mathrm{O}}^{\substack{\bullet\bullet\\\circ\bullet\\\circ\bullet}}) - \underset{\text{ATPOE}}{\nu_f}(\mathrm{T}_{\mathrm{O}}^{\substack{\bullet\bullet\\\circ\bullet\\\circ\bullet}}) - \underset{\text{CKO}}{\nu_f}(\mathrm{T}_{\mathrm{O}}^{\substack{\bullet\bullet\\\circ\bullet\\\circ\bullet}}) - \underset{\text{ATPIO}}{\nu_r}(\mathrm{T}_{\mathrm{O}}^{\substack{\bullet\bullet\\\circ\bullet\\\circ\bullet}})$$

$$\frac{d\mathrm{T}_{\mathrm{O}}^{\substack{\bullet\circ\\\circ\circ\\\bullet\circ}}}{dt} = \nu^{f}_{\mathrm{ATPIO}}\left(\mathrm{T}_{\mathrm{I}}^{\substack{\bullet\circ\\\circ\circ\\\bullet\circ}}\right) + \nu^{r}_{\mathrm{AKO}}\left(\mathrm{D}_{\mathrm{O}}^{\substack{\bullet\\\circ\\\bullet}}\mathrm{D}_{\mathrm{O}}^{\substack{\circ\\\circ\\\circ}}\right) + \nu^{r}_{\mathrm{CKO}}\left(\mathrm{D}_{\mathrm{O}}^{\substack{\bullet\\\circ\\\bullet}}\mathrm{C}_{\mathrm{O}}^{\substack{\circ\\\circ\\\circ}}\right) - \nu^{f}_{\mathrm{AKO}}\left(\mathrm{T}_{\mathrm{O}}^{\substack{\bullet\circ\\\circ\circ\\\bullet\circ}}\right) - \nu^{f}_{\mathrm{ATPOE}}\left(\mathrm{T}_{\mathrm{O}}^{\substack{\bullet\circ\\\circ\circ\\\bullet\circ}}\right) - \nu^{f}_{\mathrm{CKO}}\left(\mathrm{T}_{\mathrm{O}}^{\substack{\bullet\circ\\\circ\circ\\\bullet\circ}}\right) - \nu^{r}_{\mathrm{ATPIO}}\left(\mathrm{T}_{\mathrm{O}}^{\substack{\bullet\circ\\\circ\circ\\\bullet\circ}}\right)$$

$$\frac{d\mathrm{T}_{\mathrm{O}}^{\substack{\bullet\circ\\\circ\circ\\\bullet\bullet}}}{dt} = \nu^{f}_{\mathrm{ATPIO}}\left(\mathrm{T}_{\mathrm{I}}^{\substack{\bullet\circ\\\circ\circ\\\bullet\bullet}}\right) + \nu^{r}_{\mathrm{AKO}}\left(\mathrm{D}_{\mathrm{O}}^{\substack{\bullet\\\circ\\\bullet}}\mathrm{D}_{\mathrm{O}}^{\substack{\circ\\\circ\\\bullet}}\right) + \nu^{r}_{\mathrm{CKO}}\left(\mathrm{D}_{\mathrm{O}}^{\substack{\bullet\\\circ\\\bullet}}\mathrm{C}_{\mathrm{O}}^{\substack{\circ\\\circ\\\bullet}}\right) - \nu^{f}_{\mathrm{AKO}}\left(\mathrm{T}_{\mathrm{O}}^{\substack{\bullet\circ\\\circ\circ\\\bullet\bullet}}\right) - \nu^{f}_{\mathrm{ATPOE}}\left(\mathrm{T}_{\mathrm{O}}^{\substack{\bullet\circ\\\circ\circ\\\bullet\bullet}}\right) - \nu^{f}_{\mathrm{CKO}}\left(\mathrm{T}_{\mathrm{O}}^{\substack{\bullet\circ\\\circ\circ\\\bullet\bullet}}\right) - \nu^{r}_{\mathrm{ATPIO}}\left(\mathrm{T}_{\mathrm{O}}^{\substack{\bullet\circ\\\circ\circ\\\bullet\bullet}}\right)$$

$$\frac{d\mathrm{T}_{\mathrm{O}}^{\substack{\bullet\circ\\\circ\bullet\\\bullet\circ}}}{dt} = \nu^{f}_{\mathrm{ATPIO}}\left(\mathrm{T}_{\mathrm{I}}^{\substack{\bullet\circ\\\circ\bullet\\\bullet\circ}}\right) + \nu^{r}_{\mathrm{AKO}}\left(\mathrm{D}_{\mathrm{O}}^{\substack{\bullet\\\circ\\\bullet}}\mathrm{D}_{\mathrm{O}}^{\substack{\circ\\\bullet\\\circ}}\right) + \nu^{r}_{\mathrm{CKO}}\left(\mathrm{D}_{\mathrm{O}}^{\substack{\bullet\\\circ\\\bullet}}\mathrm{C}_{\mathrm{O}}^{\substack{\circ\\\bullet\\\circ}}\right) - \nu^{f}_{\mathrm{AKO}}\left(\mathrm{T}_{\mathrm{O}}^{\substack{\bullet\circ\\\circ\bullet\\\bullet\circ}}\right) - \nu^{f}_{\mathrm{ATPOE}}\left(\mathrm{T}_{\mathrm{O}}^{\substack{\bullet\circ\\\circ\bullet\\\bullet\circ}}\right) - \nu^{f}_{\mathrm{CKO}}\left(\mathrm{T}_{\mathrm{O}}^{\substack{\bullet\circ\\\circ\bullet\\\bullet\circ}}\right) - \nu^{r}_{\mathrm{ATPIO}}\left(\mathrm{T}_{\mathrm{O}}^{\substack{\bullet\circ\\\circ\bullet\\\bullet\circ}}\right)$$

$$\frac{d\mathrm{T}_{\mathrm{O}}^{\substack{\bullet\circ\\\circ\bullet\\\bullet\bullet}}}{dt} = \nu^{f}_{\mathrm{ATPIO}}\left(\mathrm{T}_{\mathrm{I}}^{\substack{\bullet\circ\\\circ\bullet\\\bullet\bullet}}\right) + \nu^{r}_{\mathrm{AKO}}\left(\mathrm{D}_{\mathrm{O}}^{\substack{\bullet\\\circ\\\bullet}}\mathrm{D}_{\mathrm{O}}^{\substack{\circ\\\bullet\\\bullet}}\right) + \nu^{r}_{\mathrm{CKO}}\left(\mathrm{D}_{\mathrm{O}}^{\substack{\bullet\\\circ\\\bullet}}\mathrm{C}_{\mathrm{O}}^{\substack{\circ\\\bullet\\\bullet}}\right) - \nu^{f}_{\mathrm{AKO}}\left(\mathrm{T}_{\mathrm{O}}^{\substack{\bullet\circ\\\circ\bullet\\\bullet\bullet}}\right) - \nu^{f}_{\mathrm{ATPOE}}\left(\mathrm{T}_{\mathrm{O}}^{\substack{\bullet\circ\\\circ\bullet\\\bullet\bullet}}\right) - \nu^{f}_{\mathrm{CKO}}\left(\mathrm{T}_{\mathrm{O}}^{\substack{\bullet\circ\\\circ\bullet\\\bullet\bullet}}\right) - \nu^{r}_{\mathrm{ATPIO}}\left(\mathrm{T}_{\mathrm{O}}^{\substack{\bullet\circ\\\circ\bullet\\\bullet\bullet}}\right)$$

$$\frac{d\mathrm{T}_{\mathrm{O}}^{\substack{\bullet\bullet\\\circ\circ\\\bullet\circ}}}{dt} = \nu^{f}_{\mathrm{ATPIO}}\left(\mathrm{T}_{\mathrm{I}}^{\substack{\bullet\bullet\\\circ\circ\\\bullet\circ}}\right) + \nu^{r}_{\mathrm{AKO}}\left(\mathrm{D}_{\mathrm{O}}^{\substack{\bullet\\\circ\\\bullet}}\mathrm{D}_{\mathrm{O}}^{\substack{\bullet\\\circ\\\circ}}\right) + \nu^{r}_{\mathrm{CKO}}\left(\mathrm{D}_{\mathrm{O}}^{\substack{\bullet\\\circ\\\bullet}}\mathrm{C}_{\mathrm{O}}^{\substack{\bullet\\\circ\\\circ}}\right) - \nu^{f}_{\mathrm{AKO}}\left(\mathrm{T}_{\mathrm{O}}^{\substack{\bullet\bullet\\\circ\circ\\\bullet\circ}}\right) - \nu^{f}_{\mathrm{ATPOE}}\left(\mathrm{T}_{\mathrm{O}}^{\substack{\bullet\bullet\\\circ\circ\\\bullet\circ}}\right) - \nu^{f}_{\mathrm{CKO}}\left(\mathrm{T}_{\mathrm{O}}^{\substack{\bullet\bullet\\\circ\circ\\\bullet\circ}}\right) - \nu^{r}_{\mathrm{ATPIO}}\left(\mathrm{T}_{\mathrm{O}}^{\substack{\bullet\bullet\\\circ\circ\\\bullet\circ}}\right)$$

$$\frac{d\mathrm{T}_{\mathrm{O}}^{\substack{\bullet\bullet\\\circ\circ\\\bullet\bullet}}}{dt} = \nu^{f}_{\mathrm{ATPIO}}\left(\mathrm{T}_{\mathrm{I}}^{\substack{\bullet\bullet\\\circ\circ\\\bullet\bullet}}\right) + \nu^{r}_{\mathrm{AKO}}\left(\mathrm{D}_{\mathrm{O}}^{\substack{\bullet\\\circ\\\bullet}}\mathrm{D}_{\mathrm{O}}^{\substack{\bullet\\\circ\\\bullet}}\right) + \nu^{r}_{\mathrm{CKO}}\left(\mathrm{D}_{\mathrm{O}}^{\substack{\bullet\\\circ\\\bullet}}\mathrm{C}_{\mathrm{O}}^{\substack{\bullet\\\circ\\\bullet}}\right) - \nu^{f}_{\mathrm{AKO}}\left(\mathrm{T}_{\mathrm{O}}^{\substack{\bullet\bullet\\\circ\circ\\\bullet\bullet}}\right) - \nu^{f}_{\mathrm{ATPOE}}\left(\mathrm{T}_{\mathrm{O}}^{\substack{\bullet\bullet\\\circ\circ\\\bullet\bullet}}\right) - \nu^{f}_{\mathrm{CKO}}\left(\mathrm{T}_{\mathrm{O}}^{\substack{\bullet\bullet\\\circ\circ\\\bullet\bullet}}\right) - \nu^{r}_{\mathrm{ATPIO}}\left(\mathrm{T}_{\mathrm{O}}^{\substack{\bullet\bullet\\\circ\circ\\\bullet\bullet}}\right)$$

$$\frac{d\mathrm{T}_{\mathrm{O}}^{\substack{\bullet\bullet\\\circ\bullet\\\bullet\circ}}}{dt} = \nu^{f}_{\mathrm{ATPIO}}\left(\mathrm{T}_{\mathrm{I}}^{\substack{\bullet\bullet\\\circ\bullet\\\bullet\circ}}\right) + \nu^{r}_{\mathrm{AKO}}\left(\mathrm{D}_{\mathrm{O}}^{\substack{\bullet\\\bullet\\\circ}}\mathrm{D}_{\mathrm{O}}^{\substack{\bullet\\\circ\\\bullet}}\right) + \nu^{r}_{\mathrm{CKO}}\left(\mathrm{D}_{\mathrm{O}}^{\substack{\bullet\\\circ\\\bullet}}\mathrm{C}_{\mathrm{O}}^{\substack{\bullet\\\bullet\\\circ}}\right) - \nu^{f}_{\mathrm{AKO}}\left(\mathrm{T}_{\mathrm{O}}^{\substack{\bullet\bullet\\\circ\bullet\\\bullet\circ}}\right) - \nu^{f}_{\mathrm{ATPOE}}\left(\mathrm{T}_{\mathrm{O}}^{\substack{\bullet\bullet\\\circ\bullet\\\bullet\circ}}\right) - \nu^{f}_{\mathrm{CKO}}\left(\mathrm{T}_{\mathrm{O}}^{\substack{\bullet\bullet\\\circ\bullet\\\bullet\circ}}\right) - \nu^{r}_{\mathrm{ATPIO}}\left(\mathrm{T}_{\mathrm{O}}^{\substack{\bullet\bullet\\\circ\bullet\\\bullet\circ}}\right)$$

$$\frac{d\mathrm{T}_{\mathrm{O}}^{\substack{\bullet\bullet\\\circ\bullet\\\bullet\bullet}}}{dt} = \nu^{f}_{\mathrm{ATPIO}}\left(\mathrm{T}_{\mathrm{I}}^{\substack{\bullet\bullet\\\circ\bullet\\\bullet\bullet}}\right) + \nu^{r}_{\mathrm{AKO}}\left(\mathrm{D}_{\mathrm{O}}^{\substack{\bullet\\\bullet\\\bullet}}\mathrm{D}_{\mathrm{O}}^{\substack{\bullet\\\circ\\\bullet}}\right) + \nu^{r}_{\mathrm{CKO}}\left(\mathrm{D}_{\mathrm{O}}^{\substack{\bullet\\\circ\\\bullet}}\mathrm{C}_{\mathrm{O}}^{\substack{\bullet\\\bullet\\\bullet}}\right) - \nu^{f}_{\mathrm{AKO}}\left(\mathrm{T}_{\mathrm{O}}^{\substack{\bullet\bullet\\\circ\bullet\\\bullet\bullet}}\right) - \nu^{f}_{\mathrm{ATPOE}}\left(\mathrm{T}_{\mathrm{O}}^{\substack{\bullet\bullet\\\circ\bullet\\\bullet\bullet}}\right) - \nu^{f}_{\mathrm{CKO}}\left(\mathrm{T}_{\mathrm{O}}^{\substack{\bullet\bullet\\\circ\bullet\\\bullet\bullet}}\right) - \nu^{r}_{\mathrm{ATPIO}}\left(\mathrm{T}_{\mathrm{O}}^{\substack{\bullet\bullet\\\circ\bullet\\\bullet\bullet}}\right)$$

$$\frac{d\mathrm{T}_{\mathrm{O}}^{\substack{\bullet\circ\\\bullet\circ\\\circ\circ}}}{dt} = \nu^{f}_{\mathrm{ATPIO}}\left(\mathrm{T}_{\mathrm{I}}^{\substack{\bullet\circ\\\bullet\circ\\\circ\circ}}\right) + \nu^{r}_{\mathrm{AKO}}\left(\mathrm{D}_{\mathrm{O}}^{\substack{\bullet\\\bullet\\\circ}}\mathrm{D}_{\mathrm{O}}^{\substack{\circ\\\circ\\\circ}}\right) + \nu^{r}_{\mathrm{CKO}}\left(\mathrm{D}_{\mathrm{O}}^{\substack{\bullet\\\bullet\\\circ}}\mathrm{C}_{\mathrm{O}}^{\substack{\circ\\\circ\\\circ}}\right) - \nu^{f}_{\mathrm{AKO}}\left(\mathrm{T}_{\mathrm{O}}^{\substack{\bullet\circ\\\bullet\circ\\\circ\circ}}\right) - \nu^{f}_{\mathrm{ATPOE}}\left(\mathrm{T}_{\mathrm{O}}^{\substack{\bullet\circ\\\bullet\circ\\\circ\circ}}\right) - \nu^{f}_{\mathrm{CKO}}\left(\mathrm{T}_{\mathrm{O}}^{\substack{\bullet\circ\\\bullet\circ\\\circ\circ}}\right) - \nu^{r}_{\mathrm{ATPIO}}\left(\mathrm{T}_{\mathrm{O}}^{\substack{\bullet\circ\\\bullet\circ\\\circ\circ}}\right)$$

$$\frac{d\mathrm{T}_{\mathrm{O}}^{\substack{\bullet\circ\\\bullet\circ\\\circ\bullet}}}{dt} = \nu^{f}_{\mathrm{ATPIO}}\left(\mathrm{T}_{\mathrm{I}}^{\substack{\bullet\circ\\\bullet\circ\\\circ\bullet}}\right) + \nu^{r}_{\mathrm{AKO}}\left(\mathrm{D}_{\mathrm{O}}^{\substack{\bullet\\\bullet\\\circ}}\mathrm{D}_{\mathrm{O}}^{\substack{\circ\\\circ\\\bullet}}\right) + \nu^{r}_{\mathrm{CKO}}\left(\mathrm{D}_{\mathrm{O}}^{\substack{\bullet\\\bullet\\\circ}}\mathrm{C}_{\mathrm{O}}^{\substack{\circ\\\circ\\\bullet}}\right) - \nu^{f}_{\mathrm{AKO}}\left(\mathrm{T}_{\mathrm{O}}^{\substack{\bullet\circ\\\bullet\circ\\\circ\bullet}}\right) - \nu^{f}_{\mathrm{ATPOE}}\left(\mathrm{T}_{\mathrm{O}}^{\substack{\bullet\circ\\\bullet\circ\\\circ\bullet}}\right) - \nu^{f}_{\mathrm{CKO}}\left(\mathrm{T}_{\mathrm{O}}^{\substack{\bullet\circ\\\bullet\circ\\\circ\bullet}}\right) - \nu^{r}_{\mathrm{ATPIO}}\left(\mathrm{T}_{\mathrm{O}}^{\substack{\bullet\circ\\\bullet\circ\\\circ\bullet}}\right)$$

$$\frac{d\mathrm{T}_{\mathrm{O}}^{\substack{\bullet\circ\\\bullet\bullet\\\circ\circ}}}{dt} = \nu^{f}_{\mathrm{ATPIO}}\left(\mathrm{T}_{\mathrm{I}}^{\substack{\bullet\circ\\\bullet\bullet\\\circ\circ}}\right) + \nu^{r}_{\mathrm{AKO}}\left(\mathrm{D}_{\mathrm{O}}^{\substack{\bullet\\\bullet\\\circ}}\mathrm{D}_{\mathrm{O}}^{\substack{\circ\\\bullet\\\circ}}\right) + \nu^{r}_{\mathrm{CKO}}\left(\mathrm{D}_{\mathrm{O}}^{\substack{\bullet\\\bullet\\\circ}}\mathrm{C}_{\mathrm{O}}^{\substack{\circ\\\bullet\\\circ}}\right) - \nu^{f}_{\mathrm{AKO}}\left(\mathrm{T}_{\mathrm{O}}^{\substack{\bullet\circ\\\bullet\bullet\\\circ\circ}}\right) - \nu^{f}_{\mathrm{ATPOE}}\left(\mathrm{T}_{\mathrm{O}}^{\substack{\bullet\circ\\\bullet\bullet\\\circ\circ}}\right) - \nu^{f}_{\mathrm{CKO}}\left(\mathrm{T}_{\mathrm{O}}^{\substack{\bullet\circ\\\bullet\bullet\\\circ\circ}}\right) - \nu^{r}_{\mathrm{ATPIO}}\left(\mathrm{T}_{\mathrm{O}}^{\substack{\bullet\circ\\\bullet\bullet\\\circ\circ}}\right)$$

$$\frac{d\mathrm{T}_{\mathrm{O}}^{\substack{\bullet\circ\\\bullet\bullet\\\circ\bullet}}}{dt} = \nu^{f}_{\mathrm{ATPIO}}\left(\mathrm{T}_{\mathrm{I}}^{\substack{\bullet\circ\\\bullet\bullet\\\circ\bullet}}\right) + \nu^{r}_{\mathrm{AKO}}\left(\mathrm{D}_{\mathrm{O}}^{\substack{\bullet\\\bullet\\\circ}}\mathrm{D}_{\mathrm{O}}^{\substack{\circ\\\bullet\\\bullet}}\right) + \nu^{r}_{\mathrm{CKO}}\left(\mathrm{D}_{\mathrm{O}}^{\substack{\bullet\\\bullet\\\circ}}\mathrm{C}_{\mathrm{O}}^{\substack{\circ\\\bullet\\\bullet}}\right) - \nu^{f}_{\mathrm{AKO}}\left(\mathrm{T}_{\mathrm{O}}^{\substack{\bullet\circ\\\bullet\bullet\\\circ\bullet}}\right) - \nu^{f}_{\mathrm{ATPOE}}\left(\mathrm{T}_{\mathrm{O}}^{\substack{\bullet\circ\\\bullet\bullet\\\circ\bullet}}\right) - \nu^{f}_{\mathrm{CKO}}\left(\mathrm{T}_{\mathrm{O}}^{\substack{\bullet\circ\\\bullet\bullet\\\circ\bullet}}\right) - \nu^{r}_{\mathrm{ATPIO}}\left(\mathrm{T}_{\mathrm{O}}^{\substack{\bullet\circ\\\bullet\bullet\\\circ\bullet}}\right)$$

$$\frac{d\mathrm{T}_{\mathrm{O}}^{\substack{\bullet\bullet\\\bullet\circ\\\circ\circ}}}{dt} = \nu^{f}_{\mathrm{ATPIO}}\left(\mathrm{T}_{\mathrm{I}}^{\substack{\bullet\bullet\\\bullet\circ\\\circ\circ}}\right) + \nu^{r}_{\mathrm{AKO}}\left(\mathrm{D}_{\mathrm{O}}^{\substack{\bullet\\\bullet\\\circ}}\mathrm{D}_{\mathrm{O}}^{\substack{\bullet\\\circ\\\circ}}\right) + \nu^{r}_{\mathrm{CKO}}\left(\mathrm{D}_{\mathrm{O}}^{\substack{\bullet\\\bullet\\\circ}}\mathrm{C}_{\mathrm{O}}^{\substack{\bullet\\\circ\\\circ}}\right) - \nu^{f}_{\mathrm{AKO}}\left(\mathrm{T}_{\mathrm{O}}^{\substack{\bullet\bullet\\\bullet\circ\\\circ\circ}}\right) - \nu^{f}_{\mathrm{ATPOE}}\left(\mathrm{T}_{\mathrm{O}}^{\substack{\bullet\bullet\\\bullet\circ\\\circ\circ}}\right) - \nu^{f}_{\mathrm{CKO}}\left(\mathrm{T}_{\mathrm{O}}^{\substack{\bullet\bullet\\\bullet\circ\\\circ\circ}}\right) - \nu^{r}_{\mathrm{ATPIO}}\left(\mathrm{T}_{\mathrm{O}}^{\substack{\bullet\bullet\\\bullet\circ\\\circ\circ}}\right)$$

$$\frac{d\mathrm{T}_{\mathrm{O}}^{\substack{\bullet\bullet\\\bullet\circ\\\circ\bullet}}}{dt} = \nu^{f}_{\mathrm{ATPIO}}\left(\mathrm{T}_{\mathrm{I}}^{\substack{\bullet\bullet\\\bullet\circ\\\circ\bullet}}\right) + \nu^{r}_{\mathrm{AKO}}\left(\mathrm{D}_{\mathrm{O}}^{\substack{\bullet\\\bullet\\\circ}}\mathrm{D}_{\mathrm{O}}^{\substack{\bullet\\\circ\\\bullet}}\right) + \nu^{r}_{\mathrm{CKO}}\left(\mathrm{D}_{\mathrm{O}}^{\substack{\bullet\\\bullet\\\circ}}\mathrm{C}_{\mathrm{O}}^{\substack{\bullet\\\circ\\\bullet}}\right) - \nu^{f}_{\mathrm{AKO}}\left(\mathrm{T}_{\mathrm{O}}^{\substack{\bullet\bullet\\\bullet\circ\\\circ\bullet}}\right) - \nu^{f}_{\mathrm{ATPOE}}\left(\mathrm{T}_{\mathrm{O}}^{\substack{\bullet\bullet\\\bullet\circ\\\circ\bullet}}\right) - \nu^{f}_{\mathrm{CKO}}\left(\mathrm{T}_{\mathrm{O}}^{\substack{\bullet\bullet\\\bullet\circ\\\circ\bullet}}\right) - \nu^{r}_{\mathrm{ATPIO}}\left(\mathrm{T}_{\mathrm{O}}^{\substack{\bullet\bullet\\\bullet\circ\\\circ\bullet}}\right)$$

$$\frac{d\mathrm{T}_{\mathrm{O}}^{\substack{\bullet\bullet\\\bullet\bullet\\\circ\circ}}}{dt} = \nu^{f}_{\mathrm{ATPIO}}\left(\mathrm{T}_{\mathrm{I}}^{\substack{\bullet\bullet\\\bullet\bullet\\\circ\circ}}\right) + \nu^{r}_{\mathrm{AKO}}\left(\mathrm{D}_{\mathrm{O}}^{\substack{\bullet\\\bullet\\\circ}}\mathrm{D}_{\mathrm{O}}^{\substack{\bullet\\\bullet\\\circ}}\right) + \nu^{r}_{\mathrm{CKO}}\left(\mathrm{D}_{\mathrm{O}}^{\substack{\bullet\\\bullet\\\circ}}\mathrm{C}_{\mathrm{O}}^{\substack{\bullet\\\bullet\\\circ}}\right) - \nu^{f}_{\mathrm{AKO}}\left(\mathrm{T}_{\mathrm{O}}^{\substack{\bullet\bullet\\\bullet\bullet\\\circ\circ}}\right) - \nu^{f}_{\mathrm{ATPOE}}\left(\mathrm{T}_{\mathrm{O}}^{\substack{\bullet\bullet\\\bullet\bullet\\\circ\circ}}\right) - \nu^{f}_{\mathrm{CKO}}\left(\mathrm{T}_{\mathrm{O}}^{\substack{\bullet\bullet\\\bullet\bullet\\\circ\circ}}\right) - \nu^{r}_{\mathrm{ATPIO}}\left(\mathrm{T}_{\mathrm{O}}^{\substack{\bullet\bullet\\\bullet\bullet\\\circ\circ}}\right)$$

$$\frac{d\mathrm{T}_{\mathrm{O}}^{\substack{\bullet\bullet\\\bullet\bullet\\\circ\bullet}}}{dt} = \nu^{f}_{\mathrm{ATPIO}}\left(\mathrm{T}_{\mathrm{I}}^{\substack{\bullet\bullet\\\bullet\bullet\\\circ\bullet}}\right) + \nu^{r}_{\mathrm{AKO}}\left(\mathrm{D}_{\mathrm{O}}^{\substack{\bullet\\\bullet\\\bullet}}\mathrm{D}_{\mathrm{O}}^{\substack{\bullet\\\bullet\\\circ}}\right) + \nu^{r}_{\mathrm{CKO}}\left(\mathrm{D}_{\mathrm{O}}^{\substack{\bullet\\\bullet\\\circ}}\mathrm{C}_{\mathrm{O}}^{\substack{\bullet\\\bullet\\\bullet}}\right) - \nu^{f}_{\mathrm{AKO}}\left(\mathrm{T}_{\mathrm{O}}^{\substack{\bullet\bullet\\\bullet\bullet\\\circ\bullet}}\right) - \nu^{f}_{\mathrm{ATPOE}}\left(\mathrm{T}_{\mathrm{O}}^{\substack{\bullet\bullet\\\bullet\bullet\\\circ\bullet}}\right) - \nu^{f}_{\mathrm{CKO}}\left(\mathrm{T}_{\mathrm{O}}^{\substack{\bullet\bullet\\\bullet\bullet\\\circ\bullet}}\right) - \nu^{r}_{\mathrm{ATPIO}}\left(\mathrm{T}_{\mathrm{O}}^{\substack{\bullet\bullet\\\bullet\bullet\\\circ\bullet}}\right)$$

$$\frac{d\mathrm{T}_{\mathrm{O}}^{\substack{\bullet\circ\\\bullet\circ\\\bullet\circ}}}{dt} = \nu^{f}_{\mathrm{ATPIO}}\left(\mathrm{T}_{\mathrm{I}}^{\substack{\bullet\circ\\\bullet\circ\\\bullet\circ}}\right) + \nu^{r}_{\mathrm{AKO}}\left(\mathrm{D}_{\mathrm{O}}^{\substack{\bullet\\\bullet\\\bullet}}\mathrm{D}_{\mathrm{O}}^{\substack{\circ\\\circ\\\circ}}\right) + \nu^{r}_{\mathrm{CKO}}\left(\mathrm{D}_{\mathrm{O}}^{\substack{\bullet\\\bullet\\\bullet}}\mathrm{C}_{\mathrm{O}}^{\substack{\circ\\\circ\\\circ}}\right) - \nu^{f}_{\mathrm{AKO}}\left(\mathrm{T}_{\mathrm{O}}^{\substack{\bullet\circ\\\bullet\circ\\\bullet\circ}}\right) - \nu^{f}_{\mathrm{ATPOE}}\left(\mathrm{T}_{\mathrm{O}}^{\substack{\bullet\circ\\\bullet\circ\\\bullet\circ}}\right) - \nu^{f}_{\mathrm{CKO}}\left(\mathrm{T}_{\mathrm{O}}^{\substack{\bullet\circ\\\bullet\circ\\\bullet\circ}}\right) - \nu^{r}_{\mathrm{ATPIO}}\left(\mathrm{T}_{\mathrm{O}}^{\substack{\bullet\circ\\\bullet\circ\\\bullet\circ}}\right)$$

$$\frac{d\mathrm{T}_{\mathrm{O}}^{\substack{\bullet\circ\\\bullet\circ\\\bullet\bullet}}}{dt} = \nu^{f}_{\mathrm{ATPIO}}\left(\mathrm{T}_{\mathrm{I}}^{\substack{\bullet\circ\\\bullet\circ\\\bullet\bullet}}\right) + \nu^{r}_{\mathrm{AKO}}\left(\mathrm{D}_{\mathrm{O}}^{\substack{\bullet\\\bullet\\\bullet}}\mathrm{D}_{\mathrm{O}}^{\substack{\circ\\\circ\\\bullet}}\right) + \nu^{r}_{\mathrm{CKO}}\left(\mathrm{D}_{\mathrm{O}}^{\substack{\bullet\\\bullet\\\bullet}}\mathrm{C}_{\mathrm{O}}^{\substack{\circ\\\circ\\\bullet}}\right) - \nu^{f}_{\mathrm{AKO}}\left(\mathrm{T}_{\mathrm{O}}^{\substack{\bullet\circ\\\bullet\circ\\\bullet\bullet}}\right) - \nu^{f}_{\mathrm{ATPOE}}\left(\mathrm{T}_{\mathrm{O}}^{\substack{\bullet\circ\\\bullet\circ\\\bullet\bullet}}\right) - \nu^{f}_{\mathrm{CKO}}\left(\mathrm{T}_{\mathrm{O}}^{\substack{\bullet\circ\\\bullet\circ\\\bullet\bullet}}\right) - \nu^{r}_{\mathrm{ATPIO}}\left(\mathrm{T}_{\mathrm{O}}^{\substack{\bullet\circ\\\bullet\circ\\\bullet\bullet}}\right)$$

$$\frac{d\mathrm{T}_{\mathrm{O}}^{\substack{\bullet\circ\\\bullet\bullet\\\bullet\circ}}}{dt} = \nu^{f}_{\mathrm{ATPIO}}\left(\mathrm{T}_{\mathrm{I}}^{\substack{\bullet\circ\\\bullet\bullet\\\bullet\circ}}\right) + \nu^{r}_{\mathrm{AKO}}\left(\mathrm{D}_{\mathrm{O}}^{\substack{\bullet\\\bullet\\\bullet}}\mathrm{D}_{\mathrm{O}}^{\substack{\circ\\\bullet\\\circ}}\right) + \nu^{r}_{\mathrm{CKO}}\left(\mathrm{D}_{\mathrm{O}}^{\substack{\bullet\\\bullet\\\bullet}}\mathrm{C}_{\mathrm{O}}^{\substack{\circ\\\bullet\\\circ}}\right) - \nu^{f}_{\mathrm{AKO}}\left(\mathrm{T}_{\mathrm{O}}^{\substack{\bullet\circ\\\bullet\bullet\\\bullet\circ}}\right) - \nu^{f}_{\mathrm{ATPOE}}\left(\mathrm{T}_{\mathrm{O}}^{\substack{\bullet\circ\\\bullet\bullet\\\bullet\circ}}\right) - \nu^{f}_{\mathrm{CKO}}\left(\mathrm{T}_{\mathrm{O}}^{\substack{\bullet\circ\\\bullet\bullet\\\bullet\circ}}\right) - \nu^{r}_{\mathrm{ATPIO}}\left(\mathrm{T}_{\mathrm{O}}^{\substack{\bullet\circ\\\bullet\bullet\\\bullet\circ}}\right)$$

$$\frac{d\mathrm{T}_{\mathrm{O}}^{\substack{\bullet\circ\\\bullet\bullet\\\bullet\bullet}}}{dt} = \nu^{f}_{\mathrm{ATPIO}}\left(\mathrm{T}_{\mathrm{I}}^{\substack{\bullet\circ\\\bullet\bullet\\\bullet\bullet}}\right) + \nu^{r}_{\mathrm{AKO}}\left(\mathrm{D}_{\mathrm{O}}^{\substack{\bullet\\\bullet\\\bullet}}\mathrm{D}_{\mathrm{O}}^{\substack{\circ\\\bullet\\\bullet}}\right) + \nu^{r}_{\mathrm{CKO}}\left(\mathrm{D}_{\mathrm{O}}^{\substack{\bullet\\\bullet\\\bullet}}\mathrm{C}_{\mathrm{O}}^{\substack{\circ\\\bullet\\\bullet}}\right) - \nu^{f}_{\mathrm{AKO}}\left(\mathrm{T}_{\mathrm{O}}^{\substack{\bullet\circ\\\bullet\bullet\\\bullet\bullet}}\right) - \nu^{f}_{\mathrm{ATPOE}}\left(\mathrm{T}_{\mathrm{O}}^{\substack{\bullet\circ\\\bullet\bullet\\\bullet\bullet}}\right) - \nu^{f}_{\mathrm{CKO}}\left(\mathrm{T}_{\mathrm{O}}^{\substack{\bullet\circ\\\bullet\bullet\\\bullet\bullet}}\right) - \nu^{r}_{\mathrm{ATPIO}}\left(\mathrm{T}_{\mathrm{O}}^{\substack{\bullet\circ\\\bullet\bullet\\\bullet\bullet}}\right)$$

$$\frac{d\mathrm{T}_{\mathrm{O}}^{\substack{\bullet\bullet\\\bullet\circ\\\bullet\circ}}}{dt} = \nu^{f}_{\mathrm{ATPIO}}\left(\mathrm{T}_{\mathrm{I}}^{\substack{\bullet\bullet\\\bullet\circ\\\bullet\circ}}\right) + \nu^{r}_{\mathrm{AKO}}\left(\mathrm{D}_{\mathrm{O}}^{\substack{\bullet\\\bullet\\\bullet}}\mathrm{D}_{\mathrm{O}}^{\substack{\bullet\\\circ\\\circ}}\right) + \nu^{r}_{\mathrm{CKO}}\left(\mathrm{D}_{\mathrm{O}}^{\substack{\bullet\\\bullet\\\bullet}}\mathrm{C}_{\mathrm{O}}^{\substack{\bullet\\\circ\\\circ}}\right) - \nu^{f}_{\mathrm{AKO}}\left(\mathrm{T}_{\mathrm{O}}^{\substack{\bullet\bullet\\\bullet\circ\\\bullet\circ}}\right) - \nu^{f}_{\mathrm{ATPOE}}\left(\mathrm{T}_{\mathrm{O}}^{\substack{\bullet\bullet\\\bullet\circ\\\bullet\circ}}\right) - \nu^{f}_{\mathrm{CKO}}\left(\mathrm{T}_{\mathrm{O}}^{\substack{\bullet\bullet\\\bullet\circ\\\bullet\circ}}\right) - \nu^{r}_{\mathrm{ATPIO}}\left(\mathrm{T}_{\mathrm{O}}^{\substack{\bullet\bullet\\\bullet\circ\\\bullet\circ}}\right)$$

$$\frac{d\mathrm{T_O}\substack{\bullet\bullet\\\bullet\circ\\\bullet\bullet}}{dt}=\nu^{f}_{\mathrm{ATPio}}\big(\mathrm{T_I}\substack{\bullet\bullet\\\bullet\circ\\\bullet\bullet}\big)+\nu^{r}_{\mathrm{AKo}}\big(\mathrm{D_O}\substack{\bullet\\\bullet\\\bullet}\mathrm{D_O}\substack{\bullet\\\circ\\\bullet}\big)+\nu^{r}_{\mathrm{CKo}}\big(\mathrm{D_O}\substack{\bullet\\\bullet\\\bullet}\mathrm{C_O}\substack{\bullet\\\circ\\\bullet}\big)-\nu^{f}_{\mathrm{AKo}}\big(\mathrm{T_O}\substack{\bullet\bullet\\\bullet\circ\\\bullet\bullet}\big)-\nu^{f}_{\mathrm{ATPoe}}\big(\mathrm{T_O}\substack{\bullet\bullet\\\bullet\circ\\\bullet\bullet}\big)-\nu^{f}_{\mathrm{CKo}}\big(\mathrm{T_O}\substack{\bullet\bullet\\\bullet\circ\\\bullet\bullet}\big)-\nu^{r}_{\mathrm{ATPio}}\big(\mathrm{T_O}\substack{\bullet\bullet\\\bullet\circ\\\bullet\bullet}\big)$$

$$\frac{d\mathrm{T_O}\substack{\bullet\bullet\\\bullet\bullet\\\bullet\circ}}{dt}=\nu^{f}_{\mathrm{ATPio}}\big(\mathrm{T_I}\substack{\bullet\bullet\\\bullet\bullet\\\bullet\circ}\big)+\nu^{r}_{\mathrm{AKo}}\big(\mathrm{D_O}\substack{\bullet\\\bullet\\\bullet}\mathrm{D_O}\substack{\bullet\\\bullet\\\circ}\big)+\nu^{r}_{\mathrm{CKo}}\big(\mathrm{D_O}\substack{\bullet\\\bullet\\\bullet}\mathrm{C_O}\substack{\bullet\\\bullet\\\circ}\big)-\nu^{f}_{\mathrm{AKo}}\big(\mathrm{T_O}\substack{\bullet\bullet\\\bullet\bullet\\\bullet\circ}\big)-\nu^{f}_{\mathrm{ATPoe}}\big(\mathrm{T_O}\substack{\bullet\bullet\\\bullet\bullet\\\bullet\circ}\big)-\nu^{f}_{\mathrm{CKo}}\big(\mathrm{T_O}\substack{\bullet\bullet\\\bullet\bullet\\\bullet\circ}\big)-\nu^{r}_{\mathrm{ATPio}}\big(\mathrm{T_O}\substack{\bullet\bullet\\\bullet\bullet\\\bullet\circ}\big)$$

$$\frac{d\mathrm{T_O}\substack{\bullet\bullet\\\bullet\bullet\\\bullet\bullet}}{dt}=\nu^{f}_{\mathrm{ATPio}}\big(\mathrm{T_I}\substack{\bullet\bullet\\\bullet\bullet\\\bullet\bullet}\big)+\nu^{r}_{\mathrm{AKo}}\big(\mathrm{D_O}\substack{\bullet\\\bullet\\\bullet}\mathrm{D_O}\substack{\bullet\\\bullet\\\bullet}\big)+\nu^{r}_{\mathrm{CKo}}\big(\mathrm{D_O}\substack{\bullet\\\bullet\\\bullet}\mathrm{C_O}\substack{\bullet\\\bullet\\\bullet}\big)-\nu^{f}_{\mathrm{AKo}}\big(\mathrm{T_O}\substack{\bullet\bullet\\\bullet\bullet\\\bullet\bullet}\big)-\nu^{f}_{\mathrm{ATPoe}}\big(\mathrm{T_O}\substack{\bullet\bullet\\\bullet\bullet\\\bullet\bullet}\big)-\nu^{f}_{\mathrm{CKo}}\big(\mathrm{T_O}\substack{\bullet\bullet\\\bullet\bullet\\\bullet\bullet}\big)-\nu^{r}_{\mathrm{ATPio}}\big(\mathrm{T_O}\substack{\bullet\bullet\\\bullet\bullet\\\bullet\bullet}\big)$$

$$\frac{d\mathrm{T_S}\substack{\circ\circ\\\circ\circ\\\circ\circ}}{dt}=\nu^{f}_{\mathrm{ASs}}\Big(\Big(\mathrm{P_S}\substack{\circ\\\circ\\\circ\\\circ}+\tfrac{1}{4}\big(\mathrm{P_S}\substack{\bullet\\\circ\\\circ\\\circ}+\mathrm{P_S}\substack{\circ\\\bullet\\\circ\\\circ}+\mathrm{P_S}\substack{\circ\\\circ\\\bullet\\\circ}+\mathrm{P_S}\substack{\circ\\\circ\\\circ\\\bullet}\big)\Big)\mathrm{D_S}\substack{\circ\\\circ}\Big)-\nu^{f}_{\mathrm{ATPsm}}\big(\mathrm{T_S}\substack{\circ\circ\\\circ\circ\\\circ\circ}\big)-\nu^{r}_{\mathrm{ASs}}\big(\big(\mathrm{W_S}^{\bullet}+\mathrm{W_S}^{\circ}\big)\mathrm{T_S}\substack{\circ\circ\\\circ\circ\\\circ\circ}\big)$$

$$\frac{d\mathrm{T_S}\substack{\circ\circ\\\circ\circ\\\circ\bullet}}{dt}=\nu^{f}_{\mathrm{ASs}}\Big(\Big(\tfrac{1}{4}\big(\mathrm{P_S}\substack{\bullet\\\circ\\\circ\\\circ}+\mathrm{P_S}\substack{\circ\\\bullet\\\circ\\\circ}+\mathrm{P_S}\substack{\circ\\\circ\\\bullet\\\circ}+\mathrm{P_S}\substack{\circ\\\circ\\\circ\\\bullet}\big)+\tfrac{1}{6}\big(\mathrm{P_S}\substack{\bullet\\\bullet\\\circ\\\circ}+\mathrm{P_S}\substack{\bullet\\\circ\\\bullet\\\circ}+\mathrm{P_S}\substack{\bullet\\\circ\\\circ\\\bullet}+\mathrm{P_S}\substack{\circ\\\bullet\\\bullet\\\circ}+\mathrm{P_S}\substack{\circ\\\bullet\\\circ\\\bullet}+\mathrm{P_S}\substack{\circ\\\circ\\\bullet\\\bullet}\big)\Big)\mathrm{D_S}\substack{\circ\\\circ}\Big)-\nu^{f}_{\mathrm{ATPsm}}\big(\mathrm{T_S}\substack{\circ\circ\\\circ\circ\\\circ\bullet}\big)-\nu^{r}_{\mathrm{ASs}}\big(\big(\mathrm{W_S}^{\bullet}+\mathrm{W_S}^{\circ}\big)\mathrm{T_S}\substack{\circ\circ\\\circ\circ\\\circ\bullet}\big)$$

$$\frac{d\mathrm{T_S}\substack{\circ\circ\\\circ\bullet\\\circ\circ}}{dt}=\nu^{f}_{\mathrm{ASs}}\Big(\Big(\tfrac{1}{4}\big(\mathrm{P_S}\substack{\bullet\\\circ\\\circ\\\circ}+\mathrm{P_S}\substack{\circ\\\bullet\\\circ\\\circ}+\mathrm{P_S}\substack{\circ\\\circ\\\bullet\\\circ}+\mathrm{P_S}\substack{\circ\\\circ\\\circ\\\bullet}\big)+\tfrac{1}{6}\big(\mathrm{P_S}\substack{\bullet\\\bullet\\\circ\\\circ}+\mathrm{P_S}\substack{\bullet\\\circ\\\bullet\\\circ}+\mathrm{P_S}\substack{\bullet\\\circ\\\circ\\\bullet}+\mathrm{P_S}\substack{\circ\\\bullet\\\bullet\\\circ}+\mathrm{P_S}\substack{\circ\\\bullet\\\circ\\\bullet}+\mathrm{P_S}\substack{\circ\\\circ\\\bullet\\\bullet}\big)\Big)\mathrm{D_S}\substack{\circ\\\circ}\Big)-\nu^{f}_{\mathrm{ATPsm}}\big(\mathrm{T_S}\substack{\circ\circ\\\circ\bullet\\\circ\circ}\big)-\nu^{r}_{\mathrm{ASs}}\big(\big(\mathrm{W_S}^{\bullet}+\mathrm{W_S}^{\circ}\big)\mathrm{T_S}\substack{\circ\circ\\\circ\bullet\\\circ\circ}\big)$$

$$\frac{d\mathrm{T_S}\substack{\circ\circ\\\circ\bullet\\\circ\bullet}}{dt}=\nu^{f}_{\mathrm{ASs}}\Big(\Big(\tfrac{1}{4}\big(\mathrm{P_S}\substack{\bullet\\\bullet\\\bullet\\\circ}+\mathrm{P_S}\substack{\bullet\\\bullet\\\circ\\\bullet}+\mathrm{P_S}\substack{\bullet\\\circ\\\bullet\\\bullet}+\mathrm{P_S}\substack{\circ\\\bullet\\\bullet\\\bullet}\big)+\tfrac{1}{6}\big(\mathrm{P_S}\substack{\bullet\\\bullet\\\circ\\\circ}+\mathrm{P_S}\substack{\bullet\\\circ\\\bullet\\\circ}+\mathrm{P_S}\substack{\bullet\\\circ\\\circ\\\bullet}+\mathrm{P_S}\substack{\circ\\\bullet\\\bullet\\\circ}+\mathrm{P_S}\substack{\circ\\\bullet\\\circ\\\bullet}+\mathrm{P_S}\substack{\circ\\\circ\\\bullet\\\bullet}\big)\Big)\mathrm{D_S}\substack{\circ\\\circ}\Big)-\nu^{f}_{\mathrm{ATPsm}}\big(\mathrm{T_S}\substack{\circ\circ\\\circ\bullet\\\circ\bullet}\big)-\nu^{r}_{\mathrm{ASs}}\big(\big(\mathrm{W_S}^{\bullet}+\mathrm{W_S}^{\circ}\big)\mathrm{T_S}\substack{\circ\circ\\\circ\bullet\\\circ\bullet}\big)$$

$$\frac{d\mathrm{T_S}\substack{\circ\bullet\\\circ\circ\\\circ\circ}}{dt}=\nu^{f}_{\mathrm{ASs}}\Big(\Big(\tfrac{1}{4}\big(\mathrm{P_S}\substack{\bullet\\\circ\\\circ\\\circ}+\mathrm{P_S}\substack{\circ\\\bullet\\\circ\\\circ}+\mathrm{P_S}\substack{\circ\\\circ\\\bullet\\\circ}+\mathrm{P_S}\substack{\circ\\\circ\\\circ\\\bullet}\big)+\tfrac{1}{6}\big(\mathrm{P_S}\substack{\bullet\\\bullet\\\circ\\\circ}+\mathrm{P_S}\substack{\bullet\\\circ\\\bullet\\\circ}+\mathrm{P_S}\substack{\bullet\\\circ\\\circ\\\bullet}+\mathrm{P_S}\substack{\circ\\\bullet\\\bullet\\\circ}+\mathrm{P_S}\substack{\circ\\\bullet\\\circ\\\bullet}+\mathrm{P_S}\substack{\circ\\\circ\\\bullet\\\bullet}\big)\Big)\mathrm{D_S}\substack{\circ\\\circ}\Big)-\nu^{f}_{\mathrm{ATPsm}}\big(\mathrm{T_S}\substack{\circ\bullet\\\circ\circ\\\circ\circ}\big)-\nu^{r}_{\mathrm{ASs}}\big(\big(\mathrm{W_S}^{\bullet}+\mathrm{W_S}^{\circ}\big)\mathrm{T_S}\substack{\circ\bullet\\\circ\circ\\\circ\circ}\big)$$

$$\frac{d\mathrm{T_S}\substack{\circ\bullet\\\circ\circ\\\circ\bullet}}{dt}=\nu^{f}_{\mathrm{ASs}}\Big(\Big(\tfrac{1}{4}\big(\mathrm{P_S}\substack{\bullet\\\bullet\\\bullet\\\circ}+\mathrm{P_S}\substack{\bullet\\\bullet\\\circ\\\bullet}+\mathrm{P_S}\substack{\bullet\\\circ\\\bullet\\\bullet}+\mathrm{P_S}\substack{\circ\\\bullet\\\bullet\\\bullet}\big)+\tfrac{1}{6}\big(\mathrm{P_S}\substack{\bullet\\\bullet\\\circ\\\circ}+\mathrm{P_S}\substack{\bullet\\\circ\\\bullet\\\circ}+\mathrm{P_S}\substack{\bullet\\\circ\\\circ\\\bullet}+\mathrm{P_S}\substack{\circ\\\bullet\\\bullet\\\circ}+\mathrm{P_S}\substack{\circ\\\bullet\\\circ\\\bullet}+\mathrm{P_S}\substack{\circ\\\circ\\\bullet\\\bullet}\big)\Big)\mathrm{D_S}\substack{\circ\\\circ}\Big)-\nu^{f}_{\mathrm{ATPsm}}\big(\mathrm{T_S}\substack{\circ\bullet\\\circ\circ\\\circ\bullet}\big)-\nu^{r}_{\mathrm{ASs}}\big(\big(\mathrm{W_S}^{\bullet}+\mathrm{W_S}^{\circ}\big)\mathrm{T_S}\substack{\circ\bullet\\\circ\circ\\\circ\bullet}\big)$$

$$\frac{d\mathrm{T_S}\substack{\circ\bullet\\\circ\bullet\\\circ\circ}}{dt}=\nu^{f}_{\mathrm{ASs}}\Big(\Big(\tfrac{1}{4}\big(\mathrm{P_S}\substack{\bullet\\\bullet\\\bullet\\\circ}+\mathrm{P_S}\substack{\bullet\\\bullet\\\circ\\\bullet}+\mathrm{P_S}\substack{\bullet\\\circ\\\bullet\\\bullet}+\mathrm{P_S}\substack{\circ\\\bullet\\\bullet\\\bullet}\big)+\tfrac{1}{6}\big(\mathrm{P_S}\substack{\bullet\\\bullet\\\circ\\\circ}+\mathrm{P_S}\substack{\bullet\\\circ\\\bullet\\\circ}+\mathrm{P_S}\substack{\bullet\\\circ\\\circ\\\bullet}+\mathrm{P_S}\substack{\circ\\\bullet\\\bullet\\\circ}+\mathrm{P_S}\substack{\circ\\\bullet\\\circ\\\bullet}+\mathrm{P_S}\substack{\circ\\\circ\\\bullet\\\bullet}\big)\Big)\mathrm{D_S}\substack{\circ\\\circ}\Big)-\nu^{f}_{\mathrm{ATPsm}}\big(\mathrm{T_S}\substack{\circ\bullet\\\circ\bullet\\\circ\circ}\big)-\nu^{r}_{\mathrm{ASs}}\big(\big(\mathrm{W_S}^{\bullet}+\mathrm{W_S}^{\circ}\big)\mathrm{T_S}\substack{\circ\bullet\\\circ\bullet\\\circ\circ}\big)$$

$$\frac{d\mathrm{T_S}\substack{\circ\bullet\\\circ\bullet\\\circ\bullet}}{dt}=\nu^{f}_{\mathrm{ASs}}\Big(\Big(\mathrm{P_S}\substack{\bullet\\\bullet\\\bullet\\\bullet}+\tfrac{1}{4}\big(\mathrm{P_S}\substack{\bullet\\\bullet\\\bullet\\\circ}+\mathrm{P_S}\substack{\bullet\\\bullet\\\circ\\\bullet}+\mathrm{P_S}\substack{\bullet\\\circ\\\bullet\\\bullet}+\mathrm{P_S}\substack{\circ\\\bullet\\\bullet\\\bullet}\big)\Big)\mathrm{D_S}\substack{\circ\\\circ}\Big)-\nu^{f}_{\mathrm{ATPsm}}\big(\mathrm{T_S}\substack{\circ\bullet\\\circ\bullet\\\circ\bullet}\big)-\nu^{r}_{\mathrm{ASs}}\big(\big(\mathrm{W_S}^{\bullet}+\mathrm{W_S}^{\circ}\big)\mathrm{T_S}\substack{\circ\bullet\\\circ\bullet\\\circ\bullet}\big)$$

$$\frac{d\mathrm{T_S}\substack{\circ\circ\\\circ\circ\\\bullet\circ}}{dt}=\nu^{f}_{\mathrm{ASs}}\Big(\Big(\mathrm{P_S}\substack{\circ\\\circ\\\circ\\\circ}+\tfrac{1}{4}\big(\mathrm{P_S}\substack{\bullet\\\circ\\\circ\\\circ}+\mathrm{P_S}\substack{\circ\\\bullet\\\circ\\\circ}+\mathrm{P_S}\substack{\circ\\\circ\\\bullet\\\circ}+\mathrm{P_S}\substack{\circ\\\circ\\\circ\\\bullet}\big)\Big)\mathrm{D_S}\substack{\circ\\\bullet}\Big)-\nu^{f}_{\mathrm{ATPsm}}\big(\mathrm{T_S}\substack{\circ\circ\\\circ\circ\\\bullet\circ}\big)-\nu^{r}_{\mathrm{ASs}}\big(\big(\mathrm{W_S}^{\bullet}+\mathrm{W_S}^{\circ}\big)\mathrm{T_S}\substack{\circ\circ\\\circ\circ\\\bullet\circ}\big)$$

$$\frac{d\mathrm{T_S}\substack{\circ\circ\\\circ\circ\\\bullet\bullet}}{dt}=\nu^{f}_{\mathrm{ASs}}\Big(\Big(\tfrac{1}{4}\big(\mathrm{P_S}\substack{\bullet\\\circ\\\circ\\\circ}+\mathrm{P_S}\substack{\circ\\\bullet\\\circ\\\circ}+\mathrm{P_S}\substack{\circ\\\circ\\\bullet\\\circ}+\mathrm{P_S}\substack{\circ\\\circ\\\circ\\\bullet}\big)+\tfrac{1}{6}\big(\mathrm{P_S}\substack{\bullet\\\bullet\\\circ\\\circ}+\mathrm{P_S}\substack{\bullet\\\circ\\\bullet\\\circ}+\mathrm{P_S}\substack{\bullet\\\circ\\\circ\\\bullet}+\mathrm{P_S}\substack{\circ\\\bullet\\\bullet\\\circ}+\mathrm{P_S}\substack{\circ\\\bullet\\\circ\\\bullet}+\mathrm{P_S}\substack{\circ\\\circ\\\bullet\\\bullet}\big)\Big)\mathrm{D_S}\substack{\circ\\\bullet}\Big)-\nu^{f}_{\mathrm{ATPsm}}\big(\mathrm{T_S}\substack{\circ\circ\\\circ\circ\\\bullet\bullet}\big)-\nu^{r}_{\mathrm{ASs}}\big(\big(\mathrm{W_S}^{\bullet}+\mathrm{W_S}^{\circ}\big)\mathrm{T_S}\substack{\circ\circ\\\circ\circ\\\bullet\bullet}\big)$$

$$\frac{d\mathrm{T_S}\substack{\circ\circ\\\circ\bullet\\\bullet\circ}}{dt}=\nu^{f}_{\mathrm{ASs}}\Big(\Big(\tfrac{1}{4}\big(\mathrm{P_S}\substack{\bullet\\\circ\\\circ\\\circ}+\mathrm{P_S}\substack{\circ\\\bullet\\\circ\\\circ}+\mathrm{P_S}\substack{\circ\\\circ\\\bullet\\\circ}+\mathrm{P_S}\substack{\circ\\\circ\\\circ\\\bullet}\big)+\tfrac{1}{6}\big(\mathrm{P_S}\substack{\bullet\\\bullet\\\circ\\\circ}+\mathrm{P_S}\substack{\bullet\\\circ\\\bullet\\\circ}+\mathrm{P_S}\substack{\bullet\\\circ\\\circ\\\bullet}+\mathrm{P_S}\substack{\circ\\\bullet\\\bullet\\\circ}+\mathrm{P_S}\substack{\circ\\\bullet\\\circ\\\bullet}+\mathrm{P_S}\substack{\circ\\\circ\\\bullet\\\bullet}\big)\Big)\mathrm{D_S}\substack{\circ\\\bullet}\Big)-\nu^{f}_{\mathrm{ATPsm}}\big(\mathrm{T_S}\substack{\circ\circ\\\circ\bullet\\\bullet\circ}\big)-\nu^{r}_{\mathrm{ASs}}\big(\big(\mathrm{W_S}^{\bullet}+\mathrm{W_S}^{\circ}\big)\mathrm{T_S}\substack{\circ\circ\\\circ\bullet\\\bullet\circ}\big)$$

$$\frac{d\mathrm{T_S}\substack{\circ\circ\\\circ\bullet\\\bullet\bullet}}{dt}=\nu^{f}_{\mathrm{ASs}}\Big(\Big(\tfrac{1}{4}\big(\mathrm{P_S}\substack{\bullet\\\bullet\\\bullet\\\circ}+\mathrm{P_S}\substack{\bullet\\\bullet\\\circ\\\bullet}+\mathrm{P_S}\substack{\bullet\\\circ\\\bullet\\\bullet}+\mathrm{P_S}\substack{\circ\\\bullet\\\bullet\\\bullet}\big)+\tfrac{1}{6}\big(\mathrm{P_S}\substack{\bullet\\\bullet\\\circ\\\circ}+\mathrm{P_S}\substack{\bullet\\\circ\\\bullet\\\circ}+\mathrm{P_S}\substack{\bullet\\\circ\\\circ\\\bullet}+\mathrm{P_S}\substack{\circ\\\bullet\\\bullet\\\circ}+\mathrm{P_S}\substack{\circ\\\bullet\\\circ\\\bullet}+\mathrm{P_S}\substack{\circ\\\circ\\\bullet\\\bullet}\big)\Big)\mathrm{D_S}\substack{\circ\\\bullet}\Big)-\nu^{f}_{\mathrm{ATPsm}}\big(\mathrm{T_S}\substack{\circ\circ\\\circ\bullet\\\bullet\bullet}\big)-\nu^{r}_{\mathrm{ASs}}\big(\big(\mathrm{W_S}^{\bullet}+\mathrm{W_S}^{\circ}\big)\mathrm{T_S}\substack{\circ\circ\\\circ\bullet\\\bullet\bullet}\big)$$

$$\frac{d\mathrm{T_S}\substack{\circ\bullet\\\circ\circ\\\bullet\circ}}{dt}=\nu^{f}_{\mathrm{ASs}}\Big(\Big(\tfrac{1}{4}\big(\mathrm{P_S}\substack{\bullet\\\circ\\\circ\\\circ}+\mathrm{P_S}\substack{\circ\\\bullet\\\circ\\\circ}+\mathrm{P_S}\substack{\circ\\\circ\\\bullet\\\circ}+\mathrm{P_S}\substack{\circ\\\circ\\\circ\\\bullet}\big)+\tfrac{1}{6}\big(\mathrm{P_S}\substack{\bullet\\\bullet\\\circ\\\circ}+\mathrm{P_S}\substack{\bullet\\\circ\\\bullet\\\circ}+\mathrm{P_S}\substack{\bullet\\\circ\\\circ\\\bullet}+\mathrm{P_S}\substack{\circ\\\bullet\\\bullet\\\circ}+\mathrm{P_S}\substack{\circ\\\bullet\\\circ\\\bullet}+\mathrm{P_S}\substack{\circ\\\circ\\\bullet\\\bullet}\big)\Big)\mathrm{D_S}\substack{\circ\\\bullet}\Big)-\nu^{f}_{\mathrm{ATPsm}}\big(\mathrm{T_S}\substack{\circ\bullet\\\circ\circ\\\bullet\circ}\big)-\nu^{r}_{\mathrm{ASs}}\big(\big(\mathrm{W_S}^{\bullet}+\mathrm{W_S}^{\circ}\big)\mathrm{T_S}\substack{\circ\bullet\\\circ\circ\\\bullet\circ}\big)$$

$$\frac{d\mathrm{T_S}\substack{\circ\bullet\\\circ\circ\\\bullet\bullet}}{dt}=\nu^{f}_{\mathrm{ASs}}\Big(\Big(\tfrac{1}{4}\big(\mathrm{P_S}\substack{\bullet\\\bullet\\\bullet\\\circ}+\mathrm{P_S}\substack{\bullet\\\bullet\\\circ\\\bullet}+\mathrm{P_S}\substack{\bullet\\\circ\\\bullet\\\bullet}+\mathrm{P_S}\substack{\circ\\\bullet\\\bullet\\\bullet}\big)+\tfrac{1}{6}\big(\mathrm{P_S}\substack{\bullet\\\bullet\\\circ\\\circ}+\mathrm{P_S}\substack{\bullet\\\circ\\\bullet\\\circ}+\mathrm{P_S}\substack{\bullet\\\circ\\\circ\\\bullet}+\mathrm{P_S}\substack{\circ\\\bullet\\\bullet\\\circ}+\mathrm{P_S}\substack{\circ\\\bullet\\\circ\\\bullet}+\mathrm{P_S}\substack{\circ\\\circ\\\bullet\\\bullet}\big)\Big)\mathrm{D_S}\substack{\circ\\\bullet}\Big)-\nu^{f}_{\mathrm{ATPsm}}\big(\mathrm{T_S}\substack{\circ\bullet\\\circ\circ\\\bullet\bullet}\big)-\nu^{r}_{\mathrm{ASs}}\big(\big(\mathrm{W_S}^{\bullet}+\mathrm{W_S}^{\circ}\big)\mathrm{T_S}\substack{\circ\bullet\\\circ\circ\\\bullet\bullet}\big)$$

$$\frac{d\mathrm{T_S}\substack{\circ\bullet\\\circ\bullet\\\bullet\circ}}{dt}=\nu^{f}_{\mathrm{ASs}}\Big(\Big(\tfrac{1}{4}\big(\mathrm{P_S}\substack{\bullet\\\bullet\\\bullet\\\circ}+\mathrm{P_S}\substack{\bullet\\\bullet\\\circ\\\bullet}+\mathrm{P_S}\substack{\bullet\\\circ\\\bullet\\\bullet}+\mathrm{P_S}\substack{\circ\\\bullet\\\bullet\\\bullet}\big)+\tfrac{1}{6}\big(\mathrm{P_S}\substack{\bullet\\\bullet\\\circ\\\circ}+\mathrm{P_S}\substack{\bullet\\\circ\\\bullet\\\circ}+\mathrm{P_S}\substack{\bullet\\\circ\\\circ\\\bullet}+\mathrm{P_S}\substack{\circ\\\bullet\\\bullet\\\circ}+\mathrm{P_S}\substack{\circ\\\bullet\\\circ\\\bullet}+\mathrm{P_S}\substack{\circ\\\circ\\\bullet\\\bullet}\big)\Big)\mathrm{D_S}\substack{\circ\\\bullet}\Big)-\nu^{f}_{\mathrm{ATPsm}}\big(\mathrm{T_S}\substack{\circ\bullet\\\circ\bullet\\\bullet\circ}\big)-\nu^{r}_{\mathrm{ASs}}\big(\big(\mathrm{W_S}^{\bullet}+\mathrm{W_S}^{\circ}\big)\mathrm{T_S}\substack{\circ\bullet\\\circ\bullet\\\bullet\circ}\big)$$

$$\frac{d\mathrm{T_S}\substack{\circ\bullet\\\circ\bullet\\\bullet\bullet}}{dt}=\nu^{f}_{\mathrm{ASs}}\Big(\Big(\mathrm{P_S}\substack{\bullet\\\bullet\\\bullet\\\bullet}+\tfrac{1}{4}\big(\mathrm{P_S}\substack{\bullet\\\bullet\\\bullet\\\circ}+\mathrm{P_S}\substack{\bullet\\\bullet\\\circ\\\bullet}+\mathrm{P_S}\substack{\bullet\\\circ\\\bullet\\\bullet}+\mathrm{P_S}\substack{\circ\\\bullet\\\bullet\\\bullet}\big)\Big)\mathrm{D_S}\substack{\circ\\\bullet}\Big)-\nu^{f}_{\mathrm{ATPsm}}\big(\mathrm{T_S}\substack{\circ\bullet\\\circ\bullet\\\bullet\bullet}\big)-\nu^{r}_{\mathrm{ASs}}\big(\big(\mathrm{W_S}^{\bullet}+\mathrm{W_S}^{\circ}\big)\mathrm{T_S}\substack{\circ\bullet\\\circ\bullet\\\bullet\bullet}\big)$$

$$\frac{d\mathrm{T_S}\substack{\circ\circ\\\bullet\circ\\\circ\circ}}{dt}=\nu^{f}_{\mathrm{ASs}}\Big(\Big(\mathrm{P_S}\substack{\circ\\\circ\\\circ\\\circ}+\tfrac{1}{4}\big(\mathrm{P_S}\substack{\bullet\\\circ\\\circ\\\circ}+\mathrm{P_S}\substack{\circ\\\bullet\\\circ\\\circ}+\mathrm{P_S}\substack{\circ\\\circ\\\bullet\\\circ}+\mathrm{P_S}\substack{\circ\\\circ\\\circ\\\bullet}\big)\Big)\mathrm{D_S}\substack{\bullet\\\circ}\Big)-\nu^{f}_{\mathrm{ATPsm}}\big(\mathrm{T_S}\substack{\circ\circ\\\bullet\circ\\\circ\circ}\big)-\nu^{r}_{\mathrm{ASs}}\big(\big(\mathrm{W_S}^{\bullet}+\mathrm{W_S}^{\circ}\big)\mathrm{T_S}\substack{\circ\circ\\\bullet\circ\\\circ\circ}\big)$$

$$\frac{d\mathrm{T_S}\substack{\circ\circ\\\bullet\circ\\\circ\bullet}}{dt}=\nu^{f}_{\mathrm{ASs}}\Big(\Big(\tfrac{1}{4}\big(\mathrm{P_S}\substack{\bullet\\\circ\\\circ\\\circ}+\mathrm{P_S}\substack{\circ\\\bullet\\\circ\\\circ}+\mathrm{P_S}\substack{\circ\\\circ\\\bullet\\\circ}+\mathrm{P_S}\substack{\circ\\\circ\\\circ\\\bullet}\big)+\tfrac{1}{6}\big(\mathrm{P_S}\substack{\bullet\\\bullet\\\circ\\\circ}+\mathrm{P_S}\substack{\bullet\\\circ\\\bullet\\\circ}+\mathrm{P_S}\substack{\bullet\\\circ\\\circ\\\bullet}+\mathrm{P_S}\substack{\circ\\\bullet\\\bullet\\\circ}+\mathrm{P_S}\substack{\circ\\\bullet\\\circ\\\bullet}+\mathrm{P_S}\substack{\circ\\\circ\\\bullet\\\bullet}\big)\Big)\mathrm{D_S}\substack{\bullet\\\circ}\Big)-\nu^{f}_{\mathrm{ATPsm}}\big(\mathrm{T_S}\substack{\circ\circ\\\bullet\circ\\\circ\bullet}\big)-\nu^{r}_{\mathrm{ASs}}\big(\big(\mathrm{W_S}^{\bullet}+\mathrm{W_S}^{\circ}\big)\mathrm{T_S}\substack{\circ\circ\\\bullet\circ\\\circ\bullet}\big)$$

$$\frac{d\mathrm{T_S}\substack{\circ\circ\\\bullet\bullet\\\circ\circ}}{dt}=\underset{\mathrm{ASs}}{\nu_f}\Big(\big(\tfrac{1}{4}\big(\mathrm{P_S}\substack{\bullet\\\circ\\\circ\\\circ}+\mathrm{P_S}\substack{\circ\\\bullet\\\circ\\\circ}+\mathrm{P_S}\substack{\circ\\\circ\\\bullet\\\circ}+\mathrm{P_S}\substack{\circ\\\circ\\\circ\\\bullet}\big)+\tfrac{1}{6}\big(\mathrm{P_S}\substack{\bullet\\\bullet\\\circ\\\circ}+\mathrm{P_S}\substack{\bullet\\\circ\\\bullet\\\circ}+\mathrm{P_S}\substack{\bullet\\\circ\\\circ\\\bullet}+\mathrm{P_S}\substack{\circ\\\bullet\\\bullet\\\circ}+\mathrm{P_S}\substack{\circ\\\bullet\\\circ\\\bullet}+\mathrm{P_S}\substack{\circ\\\circ\\\bullet\\\bullet}\big)\big)\mathrm{D_S}\substack{\circ\\\bullet\\\circ}\Big)-\underset{\mathrm{ATPsm}}{\nu_f}\big(\mathrm{T_S}\substack{\circ\circ\\\bullet\bullet\\\circ\circ}\big)-\underset{\mathrm{ASs}}{\nu_r}\big((\mathrm{W_S}\bullet+\mathrm{W_S}\circ)\mathrm{T_S}\substack{\circ\circ\\\bullet\bullet\\\circ\circ}\big)$$

$$\frac{d\mathrm{T_S}\substack{\circ\circ\\\bullet\bullet\\\circ\bullet}}{dt}=\underset{\mathrm{ASs}}{\nu_f}\Big(\big(\tfrac{1}{4}\big(\mathrm{P_S}\substack{\bullet\\\bullet\\\bullet\\\circ}+\mathrm{P_S}\substack{\bullet\\\bullet\\\circ\\\bullet}+\mathrm{P_S}\substack{\bullet\\\circ\\\bullet\\\bullet}+\mathrm{P_S}\substack{\circ\\\bullet\\\bullet\\\bullet}\big)+\tfrac{1}{6}\big(\mathrm{P_S}\substack{\bullet\\\bullet\\\circ\\\circ}+\mathrm{P_S}\substack{\bullet\\\circ\\\bullet\\\circ}+\mathrm{P_S}\substack{\bullet\\\circ\\\circ\\\bullet}+\mathrm{P_S}\substack{\circ\\\bullet\\\bullet\\\circ}+\mathrm{P_S}\substack{\circ\\\bullet\\\circ\\\bullet}+\mathrm{P_S}\substack{\circ\\\circ\\\bullet\\\bullet}\big)\big)\mathrm{D_S}\substack{\circ\\\bullet\\\circ}\Big)-\underset{\mathrm{ATPsm}}{\nu_f}\big(\mathrm{T_S}\substack{\circ\circ\\\bullet\bullet\\\circ\bullet}\big)-\underset{\mathrm{ASs}}{\nu_r}\big((\mathrm{W_S}\bullet+\mathrm{W_S}\circ)\mathrm{T_S}\substack{\circ\circ\\\bullet\bullet\\\circ\bullet}\big)$$

$$\frac{d\mathrm{T_S}\substack{\circ\bullet\\\bullet\circ\\\circ\circ}}{dt}=\underset{\mathrm{ASs}}{\nu_f}\Big(\big(\tfrac{1}{4}\big(\mathrm{P_S}\substack{\bullet\\\circ\\\circ\\\circ}+\mathrm{P_S}\substack{\circ\\\bullet\\\circ\\\circ}+\mathrm{P_S}\substack{\circ\\\circ\\\bullet\\\circ}+\mathrm{P_S}\substack{\circ\\\circ\\\circ\\\bullet}\big)+\tfrac{1}{6}\big(\mathrm{P_S}\substack{\bullet\\\bullet\\\circ\\\circ}+\mathrm{P_S}\substack{\bullet\\\circ\\\bullet\\\circ}+\mathrm{P_S}\substack{\bullet\\\circ\\\circ\\\bullet}+\mathrm{P_S}\substack{\circ\\\bullet\\\bullet\\\circ}+\mathrm{P_S}\substack{\circ\\\bullet\\\circ\\\bullet}+\mathrm{P_S}\substack{\circ\\\circ\\\bullet\\\bullet}\big)\big)\mathrm{D_S}\substack{\circ\\\bullet\\\circ}\Big)-\underset{\mathrm{ATPsm}}{\nu_f}\big(\mathrm{T_S}\substack{\circ\bullet\\\bullet\circ\\\circ\circ}\big)-\underset{\mathrm{ASs}}{\nu_r}\big((\mathrm{W_S}\bullet+\mathrm{W_S}\circ)\mathrm{T_S}\substack{\circ\bullet\\\bullet\circ\\\circ\circ}\big)$$

$$\frac{d\mathrm{T_S}\substack{\circ\bullet\\\bullet\circ\\\circ\bullet}}{dt}=\underset{\mathrm{ASs}}{\nu_f}\Big(\big(\tfrac{1}{4}\big(\mathrm{P_S}\substack{\bullet\\\bullet\\\bullet\\\circ}+\mathrm{P_S}\substack{\bullet\\\bullet\\\circ\\\bullet}+\mathrm{P_S}\substack{\bullet\\\circ\\\bullet\\\bullet}+\mathrm{P_S}\substack{\circ\\\bullet\\\bullet\\\bullet}\big)+\tfrac{1}{6}\big(\mathrm{P_S}\substack{\bullet\\\bullet\\\circ\\\circ}+\mathrm{P_S}\substack{\bullet\\\circ\\\bullet\\\circ}+\mathrm{P_S}\substack{\bullet\\\circ\\\circ\\\bullet}+\mathrm{P_S}\substack{\circ\\\bullet\\\bullet\\\circ}+\mathrm{P_S}\substack{\circ\\\bullet\\\circ\\\bullet}+\mathrm{P_S}\substack{\circ\\\circ\\\bullet\\\bullet}\big)\big)\mathrm{D_S}\substack{\circ\\\bullet\\\circ}\Big)-\underset{\mathrm{ATPsm}}{\nu_f}\big(\mathrm{T_S}\substack{\circ\bullet\\\bullet\circ\\\circ\bullet}\big)-\underset{\mathrm{ASs}}{\nu_r}\big((\mathrm{W_S}\bullet+\mathrm{W_S}\circ)\mathrm{T_S}\substack{\circ\bullet\\\bullet\circ\\\circ\bullet}\big)$$

$$\frac{d\mathrm{T_S}\substack{\circ\bullet\\\bullet\bullet\\\circ\circ}}{dt}=\underset{\mathrm{ASs}}{\nu_f}\Big(\big(\tfrac{1}{4}\big(\mathrm{P_S}\substack{\bullet\\\bullet\\\bullet\\\circ}+\mathrm{P_S}\substack{\bullet\\\bullet\\\circ\\\bullet}+\mathrm{P_S}\substack{\bullet\\\circ\\\bullet\\\bullet}+\mathrm{P_S}\substack{\circ\\\bullet\\\bullet\\\bullet}\big)+\tfrac{1}{6}\big(\mathrm{P_S}\substack{\bullet\\\bullet\\\circ\\\circ}+\mathrm{P_S}\substack{\bullet\\\circ\\\bullet\\\circ}+\mathrm{P_S}\substack{\bullet\\\circ\\\circ\\\bullet}+\mathrm{P_S}\substack{\circ\\\bullet\\\bullet\\\circ}+\mathrm{P_S}\substack{\circ\\\bullet\\\circ\\\bullet}+\mathrm{P_S}\substack{\circ\\\circ\\\bullet\\\bullet}\big)\big)\mathrm{D_S}\substack{\circ\\\bullet\\\circ}\Big)-\underset{\mathrm{ATPsm}}{\nu_f}\big(\mathrm{T_S}\substack{\circ\bullet\\\bullet\bullet\\\circ\circ}\big)-\underset{\mathrm{ASs}}{\nu_r}\big((\mathrm{W_S}\bullet+\mathrm{W_S}\circ)\mathrm{T_S}\substack{\circ\bullet\\\bullet\bullet\\\circ\circ}\big)$$

$$\frac{d\mathrm{T_S}\substack{\circ\bullet\\\bullet\bullet\\\circ\bullet}}{dt}=\underset{\mathrm{ASs}}{\nu_f}\Big(\big(\mathrm{P_S}\substack{\bullet\\\bullet\\\bullet\\\bullet}+\tfrac{1}{4}\big(\mathrm{P_S}\substack{\bullet\\\bullet\\\bullet\\\circ}+\mathrm{P_S}\substack{\bullet\\\bullet\\\circ\\\bullet}+\mathrm{P_S}\substack{\bullet\\\circ\\\bullet\\\bullet}+\mathrm{P_S}\substack{\circ\\\bullet\\\bullet\\\bullet}\big)\big)\mathrm{D_S}\substack{\circ\\\bullet\\\circ}\Big)-\underset{\mathrm{ATPsm}}{\nu_f}\big(\mathrm{T_S}\substack{\circ\bullet\\\bullet\bullet\\\circ\bullet}\big)-\underset{\mathrm{ASs}}{\nu_r}\big((\mathrm{W_S}\bullet+\mathrm{W_S}\circ)\mathrm{T_S}\substack{\circ\bullet\\\bullet\bullet\\\circ\bullet}\big)$$

$$\frac{d\mathrm{T_S}\substack{\circ\circ\\\bullet\circ\\\bullet\circ}}{dt}=\underset{\mathrm{ASs}}{\nu_f}\Big(\big(\mathrm{P_S}\substack{\circ\\\circ\\\circ\\\circ}+\tfrac{1}{4}\big(\mathrm{P_S}\substack{\bullet\\\circ\\\circ\\\circ}+\mathrm{P_S}\substack{\circ\\\bullet\\\circ\\\circ}+\mathrm{P_S}\substack{\circ\\\circ\\\bullet\\\circ}+\mathrm{P_S}\substack{\circ\\\circ\\\circ\\\bullet}\big)\big)\mathrm{D_S}\substack{\circ\\\bullet\\\bullet}\Big)-\underset{\mathrm{ATPsm}}{\nu_f}\big(\mathrm{T_S}\substack{\circ\circ\\\bullet\circ\\\bullet\circ}\big)-\underset{\mathrm{ASs}}{\nu_r}\big((\mathrm{W_S}\bullet+\mathrm{W_S}\circ)\mathrm{T_S}\substack{\circ\circ\\\bullet\circ\\\bullet\circ}\big)$$

$$\frac{d\mathrm{T_S}\substack{\circ\circ\\\bullet\circ\\\bullet\bullet}}{dt}=\underset{\mathrm{ASs}}{\nu_f}\Big(\big(\tfrac{1}{4}\big(\mathrm{P_S}\substack{\bullet\\\circ\\\circ\\\circ}+\mathrm{P_S}\substack{\circ\\\bullet\\\circ\\\circ}+\mathrm{P_S}\substack{\circ\\\circ\\\bullet\\\circ}+\mathrm{P_S}\substack{\circ\\\circ\\\circ\\\bullet}\big)+\tfrac{1}{6}\big(\mathrm{P_S}\substack{\bullet\\\bullet\\\circ\\\circ}+\mathrm{P_S}\substack{\bullet\\\circ\\\bullet\\\circ}+\mathrm{P_S}\substack{\bullet\\\circ\\\circ\\\bullet}+\mathrm{P_S}\substack{\circ\\\bullet\\\bullet\\\circ}+\mathrm{P_S}\substack{\circ\\\bullet\\\circ\\\bullet}+\mathrm{P_S}\substack{\circ\\\circ\\\bullet\\\bullet}\big)\big)\mathrm{D_S}\substack{\circ\\\bullet\\\bullet}\Big)-\underset{\mathrm{ATPsm}}{\nu_f}\big(\mathrm{T_S}\substack{\circ\circ\\\bullet\circ\\\bullet\bullet}\big)-\underset{\mathrm{ASs}}{\nu_r}\big((\mathrm{W_S}\bullet+\mathrm{W_S}\circ)\mathrm{T_S}\substack{\circ\circ\\\bullet\circ\\\bullet\bullet}\big)$$

$$\frac{d\mathrm{T_S}\substack{\circ\circ\\\bullet\bullet\\\bullet\circ}}{dt}=\underset{\mathrm{ASs}}{\nu_f}\Big(\big(\tfrac{1}{4}\big(\mathrm{P_S}\substack{\bullet\\\circ\\\circ\\\circ}+\mathrm{P_S}\substack{\circ\\\bullet\\\circ\\\circ}+\mathrm{P_S}\substack{\circ\\\circ\\\bullet\\\circ}+\mathrm{P_S}\substack{\circ\\\circ\\\circ\\\bullet}\big)+\tfrac{1}{6}\big(\mathrm{P_S}\substack{\bullet\\\bullet\\\circ\\\circ}+\mathrm{P_S}\substack{\bullet\\\circ\\\bullet\\\circ}+\mathrm{P_S}\substack{\bullet\\\circ\\\circ\\\bullet}+\mathrm{P_S}\substack{\circ\\\bullet\\\bullet\\\circ}+\mathrm{P_S}\substack{\circ\\\bullet\\\circ\\\bullet}+\mathrm{P_S}\substack{\circ\\\circ\\\bullet\\\bullet}\big)\big)\mathrm{D_S}\substack{\circ\\\bullet\\\bullet}\Big)-\underset{\mathrm{ATPsm}}{\nu_f}\big(\mathrm{T_S}\substack{\circ\circ\\\bullet\bullet\\\bullet\circ}\big)-\underset{\mathrm{ASs}}{\nu_r}\big((\mathrm{W_S}\bullet+\mathrm{W_S}\circ)\mathrm{T_S}\substack{\circ\circ\\\bullet\bullet\\\bullet\circ}\big)$$

$$\frac{d\mathrm{T_S}\substack{\circ\circ\\\bullet\bullet\\\bullet\bullet}}{dt}=\underset{\mathrm{ASs}}{\nu_f}\Big(\big(\tfrac{1}{4}\big(\mathrm{P_S}\substack{\bullet\\\bullet\\\bullet\\\circ}+\mathrm{P_S}\substack{\bullet\\\bullet\\\circ\\\bullet}+\mathrm{P_S}\substack{\bullet\\\circ\\\bullet\\\bullet}+\mathrm{P_S}\substack{\circ\\\bullet\\\bullet\\\bullet}\big)+\tfrac{1}{6}\big(\mathrm{P_S}\substack{\bullet\\\bullet\\\circ\\\circ}+\mathrm{P_S}\substack{\bullet\\\circ\\\bullet\\\circ}+\mathrm{P_S}\substack{\bullet\\\circ\\\circ\\\bullet}+\mathrm{P_S}\substack{\circ\\\bullet\\\bullet\\\circ}+\mathrm{P_S}\substack{\circ\\\bullet\\\circ\\\bullet}+\mathrm{P_S}\substack{\circ\\\circ\\\bullet\\\bullet}\big)\big)\mathrm{D_S}\substack{\circ\\\bullet\\\bullet}\Big)-\underset{\mathrm{ATPsm}}{\nu_f}\big(\mathrm{T_S}\substack{\circ\circ\\\bullet\bullet\\\bullet\bullet}\big)-\underset{\mathrm{ASs}}{\nu_r}\big((\mathrm{W_S}\bullet+\mathrm{W_S}\circ)\mathrm{T_S}\substack{\circ\circ\\\bullet\bullet\\\bullet\bullet}\big)$$

$$\frac{d\mathrm{T_S}\substack{\circ\bullet\\\bullet\circ\\\bullet\circ}}{dt}=\underset{\mathrm{ASs}}{\nu_f}\Big(\big(\tfrac{1}{4}\big(\mathrm{P_S}\substack{\bullet\\\circ\\\circ\\\circ}+\mathrm{P_S}\substack{\circ\\\bullet\\\circ\\\circ}+\mathrm{P_S}\substack{\circ\\\circ\\\bullet\\\circ}+\mathrm{P_S}\substack{\circ\\\circ\\\circ\\\bullet}\big)+\tfrac{1}{6}\big(\mathrm{P_S}\substack{\bullet\\\bullet\\\circ\\\circ}+\mathrm{P_S}\substack{\bullet\\\circ\\\bullet\\\circ}+\mathrm{P_S}\substack{\bullet\\\circ\\\circ\\\bullet}+\mathrm{P_S}\substack{\circ\\\bullet\\\bullet\\\circ}+\mathrm{P_S}\substack{\circ\\\bullet\\\circ\\\bullet}+\mathrm{P_S}\substack{\circ\\\circ\\\bullet\\\bullet}\big)\big)\mathrm{D_S}\substack{\circ\\\bullet\\\bullet}\Big)-\underset{\mathrm{ATPsm}}{\nu_f}\big(\mathrm{T_S}\substack{\circ\bullet\\\bullet\circ\\\bullet\circ}\big)-\underset{\mathrm{ASs}}{\nu_r}\big((\mathrm{W_S}\bullet+\mathrm{W_S}\circ)\mathrm{T_S}\substack{\circ\bullet\\\bullet\circ\\\bullet\circ}\big)$$

$$\frac{d\mathrm{T_S}\substack{\circ\bullet\\\bullet\circ\\\bullet\bullet}}{dt}=\underset{\mathrm{ASs}}{\nu_f}\Big(\big(\tfrac{1}{4}\big(\mathrm{P_S}\substack{\bullet\\\bullet\\\bullet\\\circ}+\mathrm{P_S}\substack{\bullet\\\bullet\\\circ\\\bullet}+\mathrm{P_S}\substack{\bullet\\\circ\\\bullet\\\bullet}+\mathrm{P_S}\substack{\circ\\\bullet\\\bullet\\\bullet}\big)+\tfrac{1}{6}\big(\mathrm{P_S}\substack{\bullet\\\bullet\\\circ\\\circ}+\mathrm{P_S}\substack{\bullet\\\circ\\\bullet\\\circ}+\mathrm{P_S}\substack{\bullet\\\circ\\\circ\\\bullet}+\mathrm{P_S}\substack{\circ\\\bullet\\\bullet\\\circ}+\mathrm{P_S}\substack{\circ\\\bullet\\\circ\\\bullet}+\mathrm{P_S}\substack{\circ\\\circ\\\bullet\\\bullet}\big)\big)\mathrm{D_S}\substack{\circ\\\bullet\\\bullet}\Big)-\underset{\mathrm{ATPsm}}{\nu_f}\big(\mathrm{T_S}\substack{\circ\bullet\\\bullet\circ\\\bullet\bullet}\big)-\underset{\mathrm{ASs}}{\nu_r}\big((\mathrm{W_S}\bullet+\mathrm{W_S}\circ)\mathrm{T_S}\substack{\circ\bullet\\\bullet\circ\\\bullet\bullet}\big)$$

$$\frac{d\mathrm{T_S}\substack{\circ\bullet\\\bullet\bullet\\\bullet\circ}}{dt}=\underset{\mathrm{ASs}}{\nu_f}\Big(\big(\tfrac{1}{4}\big(\mathrm{P_S}\substack{\bullet\\\bullet\\\bullet\\\circ}+\mathrm{P_S}\substack{\bullet\\\bullet\\\circ\\\bullet}+\mathrm{P_S}\substack{\bullet\\\circ\\\bullet\\\bullet}+\mathrm{P_S}\substack{\circ\\\bullet\\\bullet\\\bullet}\big)+\tfrac{1}{6}\big(\mathrm{P_S}\substack{\bullet\\\bullet\\\circ\\\circ}+\mathrm{P_S}\substack{\bullet\\\circ\\\bullet\\\circ}+\mathrm{P_S}\substack{\bullet\\\circ\\\circ\\\bullet}+\mathrm{P_S}\substack{\circ\\\bullet\\\bullet\\\circ}+\mathrm{P_S}\substack{\circ\\\bullet\\\circ\\\bullet}+\mathrm{P_S}\substack{\circ\\\circ\\\bullet\\\bullet}\big)\big)\mathrm{D_S}\substack{\circ\\\bullet\\\bullet}\Big)-\underset{\mathrm{ATPsm}}{\nu_f}\big(\mathrm{T_S}\substack{\circ\bullet\\\bullet\bullet\\\bullet\circ}\big)-\underset{\mathrm{ASs}}{\nu_r}\big((\mathrm{W_S}\bullet+\mathrm{W_S}\circ)\mathrm{T_S}\substack{\circ\bullet\\\bullet\bullet\\\bullet\circ}\big)$$

$$\frac{d\mathrm{T_S}\substack{\circ\bullet\\\bullet\bullet\\\bullet\bullet}}{dt}=\underset{\mathrm{ASs}}{\nu_f}\Big(\big(\mathrm{P_S}\substack{\bullet\\\bullet\\\bullet\\\bullet}+\tfrac{1}{4}\big(\mathrm{P_S}\substack{\bullet\\\bullet\\\bullet\\\circ}+\mathrm{P_S}\substack{\bullet\\\bullet\\\circ\\\bullet}+\mathrm{P_S}\substack{\bullet\\\circ\\\bullet\\\bullet}+\mathrm{P_S}\substack{\circ\\\bullet\\\bullet\\\bullet}\big)\big)\mathrm{D_S}\substack{\circ\\\bullet\\\bullet}\Big)-\underset{\mathrm{ATPsm}}{\nu_f}\big(\mathrm{T_S}\substack{\circ\bullet\\\bullet\bullet\\\bullet\bullet}\big)-\underset{\mathrm{ASs}}{\nu_r}\big((\mathrm{W_S}\bullet+\mathrm{W_S}\circ)\mathrm{T_S}\substack{\circ\bullet\\\bullet\bullet\\\bullet\bullet}\big)$$

$$\frac{d\mathrm{T_S}\substack{\bullet\circ\\\circ\circ\\\circ\circ}}{dt}=\underset{\mathrm{ASs}}{\nu_f}\Big(\big(\mathrm{P_S}\substack{\circ\\\circ\\\circ\\\circ}+\tfrac{1}{4}\big(\mathrm{P_S}\substack{\bullet\\\circ\\\circ\\\circ}+\mathrm{P_S}\substack{\circ\\\bullet\\\circ\\\circ}+\mathrm{P_S}\substack{\circ\\\circ\\\bullet\\\circ}+\mathrm{P_S}\substack{\circ\\\circ\\\circ\\\bullet}\big)\big)\mathrm{D_S}\substack{\bullet\\\circ\\\circ}\Big)-\underset{\mathrm{ATPsm}}{\nu_f}\big(\mathrm{T_S}\substack{\bullet\circ\\\circ\circ\\\circ\circ}\big)-\underset{\mathrm{ASs}}{\nu_r}\big((\mathrm{W_S}\bullet+\mathrm{W_S}\circ)\mathrm{T_S}\substack{\bullet\circ\\\circ\circ\\\circ\circ}\big)$$

$$\frac{d\mathrm{T_S}\substack{\bullet\circ\\\circ\circ\\\circ\bullet}}{dt}=\underset{\mathrm{ASs}}{\nu_f}\Big(\big(\tfrac{1}{4}\big(\mathrm{P_S}\substack{\bullet\\\circ\\\circ\\\circ}+\mathrm{P_S}\substack{\circ\\\bullet\\\circ\\\circ}+\mathrm{P_S}\substack{\circ\\\circ\\\bullet\\\circ}+\mathrm{P_S}\substack{\circ\\\circ\\\circ\\\bullet}\big)+\tfrac{1}{6}\big(\mathrm{P_S}\substack{\bullet\\\bullet\\\circ\\\circ}+\mathrm{P_S}\substack{\bullet\\\circ\\\bullet\\\circ}+\mathrm{P_S}\substack{\bullet\\\circ\\\circ\\\bullet}+\mathrm{P_S}\substack{\circ\\\bullet\\\bullet\\\circ}+\mathrm{P_S}\substack{\circ\\\bullet\\\circ\\\bullet}+\mathrm{P_S}\substack{\circ\\\circ\\\bullet\\\bullet}\big)\big)\mathrm{D_S}\substack{\bullet\\\circ\\\circ}\Big)-\underset{\mathrm{ATPsm}}{\nu_f}\big(\mathrm{T_S}\substack{\bullet\circ\\\circ\circ\\\circ\bullet}\big)-\underset{\mathrm{ASs}}{\nu_r}\big((\mathrm{W_S}\bullet+\mathrm{W_S}\circ)\mathrm{T_S}\substack{\bullet\circ\\\circ\circ\\\circ\bullet}\big)$$

$$\frac{d\mathrm{T_S}\substack{\bullet\circ\\\circ\bullet\\\circ\circ}}{dt}=\underset{\mathrm{ASs}}{\nu_f}\Big(\big(\tfrac{1}{4}\big(\mathrm{P_S}\substack{\bullet\\\circ\\\circ\\\circ}+\mathrm{P_S}\substack{\circ\\\bullet\\\circ\\\circ}+\mathrm{P_S}\substack{\circ\\\circ\\\bullet\\\circ}+\mathrm{P_S}\substack{\circ\\\circ\\\circ\\\bullet}\big)+\tfrac{1}{6}\big(\mathrm{P_S}\substack{\bullet\\\bullet\\\circ\\\circ}+\mathrm{P_S}\substack{\bullet\\\circ\\\bullet\\\circ}+\mathrm{P_S}\substack{\bullet\\\circ\\\circ\\\bullet}+\mathrm{P_S}\substack{\circ\\\bullet\\\bullet\\\circ}+\mathrm{P_S}\substack{\circ\\\bullet\\\circ\\\bullet}+\mathrm{P_S}\substack{\circ\\\circ\\\bullet\\\bullet}\big)\big)\mathrm{D_S}\substack{\bullet\\\circ\\\circ}\Big)-\underset{\mathrm{ATPsm}}{\nu_f}\big(\mathrm{T_S}\substack{\bullet\circ\\\circ\bullet\\\circ\circ}\big)-\underset{\mathrm{ASs}}{\nu_r}\big((\mathrm{W_S}\bullet+\mathrm{W_S}\circ)\mathrm{T_S}\substack{\bullet\circ\\\circ\bullet\\\circ\circ}\big)$$

$$\frac{d\mathrm{T_S}\substack{\bullet\circ\\\circ\bullet\\\circ\bullet}}{dt}=\underset{\mathrm{ASs}}{\nu_f}\Big(\big(\tfrac{1}{4}\big(\mathrm{P_S}\substack{\bullet\\\bullet\\\bullet\\\circ}+\mathrm{P_S}\substack{\bullet\\\bullet\\\circ\\\bullet}+\mathrm{P_S}\substack{\bullet\\\circ\\\bullet\\\bullet}+\mathrm{P_S}\substack{\circ\\\bullet\\\bullet\\\bullet}\big)+\tfrac{1}{6}\big(\mathrm{P_S}\substack{\bullet\\\bullet\\\circ\\\circ}+\mathrm{P_S}\substack{\bullet\\\circ\\\bullet\\\circ}+\mathrm{P_S}\substack{\bullet\\\circ\\\circ\\\bullet}+\mathrm{P_S}\substack{\circ\\\bullet\\\bullet\\\circ}+\mathrm{P_S}\substack{\circ\\\bullet\\\circ\\\bullet}+\mathrm{P_S}\substack{\circ\\\circ\\\bullet\\\bullet}\big)\big)\mathrm{D_S}\substack{\bullet\\\circ\\\circ}\Big)-\underset{\mathrm{ATPsm}}{\nu_f}\big(\mathrm{T_S}\substack{\bullet\circ\\\circ\bullet\\\circ\bullet}\big)-\underset{\mathrm{ASs}}{\nu_r}\big((\mathrm{W_S}\bullet+\mathrm{W_S}\circ)\mathrm{T_S}\substack{\bullet\circ\\\circ\bullet\\\circ\bullet}\big)$$

$$\frac{d\mathrm{T_S}\substack{\bullet\bullet\\\circ\circ\\\circ\circ}}{dt}=\underset{\mathrm{ASs}}{\nu_f}\Big(\big(\tfrac{1}{4}\big(\mathrm{P_S}\substack{\bullet\\\circ\\\circ\\\circ}+\mathrm{P_S}\substack{\circ\\\bullet\\\circ\\\circ}+\mathrm{P_S}\substack{\circ\\\circ\\\bullet\\\circ}+\mathrm{P_S}\substack{\circ\\\circ\\\circ\\\bullet}\big)+\tfrac{1}{6}\big(\mathrm{P_S}\substack{\bullet\\\bullet\\\circ\\\circ}+\mathrm{P_S}\substack{\bullet\\\circ\\\bullet\\\circ}+\mathrm{P_S}\substack{\bullet\\\circ\\\circ\\\bullet}+\mathrm{P_S}\substack{\circ\\\bullet\\\bullet\\\circ}+\mathrm{P_S}\substack{\circ\\\bullet\\\circ\\\bullet}+\mathrm{P_S}\substack{\circ\\\circ\\\bullet\\\bullet}\big)\big)\mathrm{D_S}\substack{\bullet\\\circ\\\circ}\Big)-\underset{\mathrm{ATPsm}}{\nu_f}\big(\mathrm{T_S}\substack{\bullet\bullet\\\circ\circ\\\circ\circ}\big)-\underset{\mathrm{ASs}}{\nu_r}\big((\mathrm{W_S}\bullet+\mathrm{W_S}\circ)\mathrm{T_S}\substack{\bullet\bullet\\\circ\circ\\\circ\circ}\big)$$

$$\frac{d\mathrm{T_S}\substack{\bullet\bullet\\\circ\circ\\\circ\bullet}}{dt}=\underset{\mathrm{ASs}}{\nu_f}\Big(\big(\tfrac{1}{4}\big(\mathrm{P_S}\substack{\bullet\\\bullet\\\bullet\\\circ}+\mathrm{P_S}\substack{\bullet\\\bullet\\\circ\\\bullet}+\mathrm{P_S}\substack{\bullet\\\circ\\\bullet\\\bullet}+\mathrm{P_S}\substack{\circ\\\bullet\\\bullet\\\bullet}\big)+\tfrac{1}{6}\big(\mathrm{P_S}\substack{\bullet\\\bullet\\\circ\\\circ}+\mathrm{P_S}\substack{\bullet\\\circ\\\bullet\\\circ}+\mathrm{P_S}\substack{\bullet\\\circ\\\circ\\\bullet}+\mathrm{P_S}\substack{\circ\\\bullet\\\bullet\\\circ}+\mathrm{P_S}\substack{\circ\\\bullet\\\circ\\\bullet}+\mathrm{P_S}\substack{\circ\\\circ\\\bullet\\\bullet}\big)\big)\mathrm{D_S}\substack{\bullet\\\circ\\\circ}\Big)-\underset{\mathrm{ATPsm}}{\nu_f}\big(\mathrm{T_S}\substack{\bullet\bullet\\\circ\circ\\\circ\bullet}\big)-\underset{\mathrm{ASs}}{\nu_r}\big((\mathrm{W_S}\bullet+\mathrm{W_S}\circ)\mathrm{T_S}\substack{\bullet\bullet\\\circ\circ\\\circ\bullet}\big)$$

$$\frac{d\mathrm{T_S}\substack{\bullet\bullet\\\circ\bullet\\\circ\circ}}{dt}=\underset{\mathrm{ASs}}{\nu_f}\Big(\big(\tfrac{1}{4}\big(\mathrm{P_S}\substack{\bullet\\\bullet\\\bullet\\\circ}+\mathrm{P_S}\substack{\bullet\\\bullet\\\circ\\\bullet}+\mathrm{P_S}\substack{\bullet\\\circ\\\bullet\\\bullet}+\mathrm{P_S}\substack{\circ\\\bullet\\\bullet\\\bullet}\big)+\tfrac{1}{6}\big(\mathrm{P_S}\substack{\bullet\\\bullet\\\circ\\\circ}+\mathrm{P_S}\substack{\bullet\\\circ\\\bullet\\\circ}+\mathrm{P_S}\substack{\bullet\\\circ\\\circ\\\bullet}+\mathrm{P_S}\substack{\circ\\\bullet\\\bullet\\\circ}+\mathrm{P_S}\substack{\circ\\\bullet\\\circ\\\bullet}+\mathrm{P_S}\substack{\circ\\\circ\\\bullet\\\bullet}\big)\big)\mathrm{D_S}\substack{\bullet\\\circ\\\circ}\Big)-\underset{\mathrm{ATPsm}}{\nu_f}\big(\mathrm{T_S}\substack{\bullet\bullet\\\circ\bullet\\\circ\circ}\big)-\underset{\mathrm{ASs}}{\nu_r}\big((\mathrm{W_S}\bullet+\mathrm{W_S}\circ)\mathrm{T_S}\substack{\bullet\bullet\\\circ\bullet\\\circ\circ}\big)$$

$$\frac{d\mathrm{T}_{\mathrm{S}}^{\substack{\bullet\bullet\\\circ\bullet\\\circ\bullet}}}{dt}=\nu^{f}_{\mathrm{ASs}}\Big(\big(\mathrm{P}_{\mathrm{S}}^{\substack{\bullet\\\bullet\\\bullet\\\bullet}}+\tfrac{1}{4}\big(\mathrm{P}_{\mathrm{S}}^{\substack{\bullet\\\bullet\\\bullet\\\circ}}+\mathrm{P}_{\mathrm{S}}^{\substack{\bullet\\\bullet\\\circ\\\bullet}}+\mathrm{P}_{\mathrm{S}}^{\substack{\bullet\\\circ\\\bullet\\\bullet}}+\mathrm{P}_{\mathrm{S}}^{\substack{\circ\\\bullet\\\bullet\\\bullet}}\big)\big)\mathrm{D}_{\mathrm{S}}^{\substack{\bullet\\\circ\\\circ}}\Big)-\nu^{f}_{\mathrm{ATPsm}}\big(\mathrm{T}_{\mathrm{S}}^{\substack{\bullet\bullet\\\circ\bullet\\\circ\bullet}}\big)-\nu^{r}_{\mathrm{ASs}}\big((\mathrm{W}_{\mathrm{S}}^{\bullet}+\mathrm{W}_{\mathrm{S}}^{\circ})\mathrm{T}_{\mathrm{S}}^{\substack{\bullet\bullet\\\circ\bullet\\\circ\bullet}}\big)$$

$$\frac{d\mathrm{T}_{\mathrm{S}}^{\substack{\bullet\circ\\\circ\circ\\\bullet\circ}}}{dt}=\nu^{f}_{\mathrm{ASs}}\Big(\big(\mathrm{P}_{\mathrm{S}}^{\substack{\circ\\\circ\\\circ\\\circ}}+\tfrac{1}{4}\big(\mathrm{P}_{\mathrm{S}}^{\substack{\bullet\\\circ\\\circ\\\circ}}+\mathrm{P}_{\mathrm{S}}^{\substack{\circ\\\bullet\\\circ\\\circ}}+\mathrm{P}_{\mathrm{S}}^{\substack{\circ\\\circ\\\bullet\\\circ}}+\mathrm{P}_{\mathrm{S}}^{\substack{\circ\\\circ\\\circ\\\bullet}}\big)\big)\mathrm{D}_{\mathrm{S}}^{\substack{\bullet\\\circ\\\bullet}}\Big)-\nu^{f}_{\mathrm{ATPsm}}\big(\mathrm{T}_{\mathrm{S}}^{\substack{\bullet\circ\\\circ\circ\\\bullet\circ}}\big)-\nu^{r}_{\mathrm{ASs}}\big((\mathrm{W}_{\mathrm{S}}^{\bullet}+\mathrm{W}_{\mathrm{S}}^{\circ})\mathrm{T}_{\mathrm{S}}^{\substack{\bullet\circ\\\circ\circ\\\bullet\circ}}\big)$$

$$\frac{d\mathrm{T}_{\mathrm{S}}^{\substack{\bullet\circ\\\circ\circ\\\bullet\bullet}}}{dt}=\nu^{f}_{\mathrm{ASs}}\Big(\big(\tfrac{1}{4}\big(\mathrm{P}_{\mathrm{S}}^{\substack{\bullet\\\circ\\\circ\\\circ}}+\mathrm{P}_{\mathrm{S}}^{\substack{\circ\\\bullet\\\circ\\\circ}}+\mathrm{P}_{\mathrm{S}}^{\substack{\circ\\\circ\\\bullet\\\circ}}+\mathrm{P}_{\mathrm{S}}^{\substack{\circ\\\circ\\\circ\\\bullet}}\big)+\tfrac{1}{6}\big(\mathrm{P}_{\mathrm{S}}^{\substack{\bullet\\\bullet\\\circ\\\circ}}+\mathrm{P}_{\mathrm{S}}^{\substack{\bullet\\\circ\\\bullet\\\circ}}+\mathrm{P}_{\mathrm{S}}^{\substack{\bullet\\\circ\\\circ\\\bullet}}+\mathrm{P}_{\mathrm{S}}^{\substack{\circ\\\bullet\\\bullet\\\circ}}+\mathrm{P}_{\mathrm{S}}^{\substack{\circ\\\bullet\\\circ\\\bullet}}+\mathrm{P}_{\mathrm{S}}^{\substack{\circ\\\circ\\\bullet\\\bullet}}\big)\big)\mathrm{D}_{\mathrm{S}}^{\substack{\bullet\\\circ\\\bullet}}\Big)-\nu^{f}_{\mathrm{ATPsm}}\big(\mathrm{T}_{\mathrm{S}}^{\substack{\bullet\circ\\\circ\circ\\\bullet\bullet}}\big)-\nu^{r}_{\mathrm{ASs}}\big((\mathrm{W}_{\mathrm{S}}^{\bullet}+\mathrm{W}_{\mathrm{S}}^{\circ})\mathrm{T}_{\mathrm{S}}^{\substack{\bullet\circ\\\circ\circ\\\bullet\bullet}}\big)$$

$$\frac{d\mathrm{T}_{\mathrm{S}}^{\substack{\bullet\circ\\\circ\bullet\\\bullet\circ}}}{dt}=\nu^{f}_{\mathrm{ASs}}\Big(\big(\tfrac{1}{4}\big(\mathrm{P}_{\mathrm{S}}^{\substack{\bullet\\\circ\\\circ\\\circ}}+\mathrm{P}_{\mathrm{S}}^{\substack{\circ\\\bullet\\\circ\\\circ}}+\mathrm{P}_{\mathrm{S}}^{\substack{\circ\\\circ\\\bullet\\\circ}}+\mathrm{P}_{\mathrm{S}}^{\substack{\circ\\\circ\\\circ\\\bullet}}\big)+\tfrac{1}{6}\big(\mathrm{P}_{\mathrm{S}}^{\substack{\bullet\\\bullet\\\circ\\\circ}}+\mathrm{P}_{\mathrm{S}}^{\substack{\bullet\\\circ\\\bullet\\\circ}}+\mathrm{P}_{\mathrm{S}}^{\substack{\bullet\\\circ\\\circ\\\bullet}}+\mathrm{P}_{\mathrm{S}}^{\substack{\circ\\\bullet\\\bullet\\\circ}}+\mathrm{P}_{\mathrm{S}}^{\substack{\circ\\\bullet\\\circ\\\bullet}}+\mathrm{P}_{\mathrm{S}}^{\substack{\circ\\\circ\\\bullet\\\bullet}}\big)\big)\mathrm{D}_{\mathrm{S}}^{\substack{\bullet\\\circ\\\bullet}}\Big)-\nu^{f}_{\mathrm{ATPsm}}\big(\mathrm{T}_{\mathrm{S}}^{\substack{\bullet\circ\\\circ\bullet\\\bullet\circ}}\big)-\nu^{r}_{\mathrm{ASs}}\big((\mathrm{W}_{\mathrm{S}}^{\bullet}+\mathrm{W}_{\mathrm{S}}^{\circ})\mathrm{T}_{\mathrm{S}}^{\substack{\bullet\circ\\\circ\bullet\\\bullet\circ}}\big)$$

$$\frac{d\mathrm{T}_{\mathrm{S}}^{\substack{\bullet\circ\\\circ\bullet\\\bullet\bullet}}}{dt}=\nu^{f}_{\mathrm{ASs}}\Big(\big(\tfrac{1}{4}\big(\mathrm{P}_{\mathrm{S}}^{\substack{\bullet\\\bullet\\\bullet\\\circ}}+\mathrm{P}_{\mathrm{S}}^{\substack{\bullet\\\bullet\\\circ\\\bullet}}+\mathrm{P}_{\mathrm{S}}^{\substack{\bullet\\\circ\\\bullet\\\bullet}}+\mathrm{P}_{\mathrm{S}}^{\substack{\circ\\\bullet\\\bullet\\\bullet}}\big)+\tfrac{1}{6}\big(\mathrm{P}_{\mathrm{S}}^{\substack{\bullet\\\bullet\\\circ\\\circ}}+\mathrm{P}_{\mathrm{S}}^{\substack{\bullet\\\circ\\\bullet\\\circ}}+\mathrm{P}_{\mathrm{S}}^{\substack{\bullet\\\circ\\\circ\\\bullet}}+\mathrm{P}_{\mathrm{S}}^{\substack{\circ\\\bullet\\\bullet\\\circ}}+\mathrm{P}_{\mathrm{S}}^{\substack{\circ\\\bullet\\\circ\\\bullet}}+\mathrm{P}_{\mathrm{S}}^{\substack{\circ\\\circ\\\bullet\\\bullet}}\big)\big)\mathrm{D}_{\mathrm{S}}^{\substack{\bullet\\\circ\\\bullet}}\Big)-\nu^{f}_{\mathrm{ATPsm}}\big(\mathrm{T}_{\mathrm{S}}^{\substack{\bullet\circ\\\circ\bullet\\\bullet\bullet}}\big)-\nu^{r}_{\mathrm{ASs}}\big((\mathrm{W}_{\mathrm{S}}^{\bullet}+\mathrm{W}_{\mathrm{S}}^{\circ})\mathrm{T}_{\mathrm{S}}^{\substack{\bullet\circ\\\circ\bullet\\\bullet\bullet}}\big)$$

$$\frac{d\mathrm{T}_{\mathrm{S}}^{\substack{\bullet\bullet\\\circ\circ\\\bullet\circ}}}{dt}=\nu^{f}_{\mathrm{ASs}}\Big(\big(\tfrac{1}{4}\big(\mathrm{P}_{\mathrm{S}}^{\substack{\bullet\\\circ\\\circ\\\circ}}+\mathrm{P}_{\mathrm{S}}^{\substack{\circ\\\bullet\\\circ\\\circ}}+\mathrm{P}_{\mathrm{S}}^{\substack{\circ\\\circ\\\bullet\\\circ}}+\mathrm{P}_{\mathrm{S}}^{\substack{\circ\\\circ\\\circ\\\bullet}}\big)+\tfrac{1}{6}\big(\mathrm{P}_{\mathrm{S}}^{\substack{\bullet\\\bullet\\\circ\\\circ}}+\mathrm{P}_{\mathrm{S}}^{\substack{\bullet\\\circ\\\bullet\\\circ}}+\mathrm{P}_{\mathrm{S}}^{\substack{\bullet\\\circ\\\circ\\\bullet}}+\mathrm{P}_{\mathrm{S}}^{\substack{\circ\\\bullet\\\bullet\\\circ}}+\mathrm{P}_{\mathrm{S}}^{\substack{\circ\\\bullet\\\circ\\\bullet}}+\mathrm{P}_{\mathrm{S}}^{\substack{\circ\\\circ\\\bullet\\\bullet}}\big)\big)\mathrm{D}_{\mathrm{S}}^{\substack{\bullet\\\circ\\\bullet}}\Big)-\nu^{f}_{\mathrm{ATPsm}}\big(\mathrm{T}_{\mathrm{S}}^{\substack{\bullet\bullet\\\circ\circ\\\bullet\circ}}\big)-\nu^{r}_{\mathrm{ASs}}\big((\mathrm{W}_{\mathrm{S}}^{\bullet}+\mathrm{W}_{\mathrm{S}}^{\circ})\mathrm{T}_{\mathrm{S}}^{\substack{\bullet\bullet\\\circ\circ\\\bullet\circ}}\big)$$

$$\frac{d\mathrm{T}_{\mathrm{S}}^{\substack{\bullet\bullet\\\circ\circ\\\bullet\bullet}}}{dt}=\nu^{f}_{\mathrm{ASs}}\Big(\big(\tfrac{1}{4}\big(\mathrm{P}_{\mathrm{S}}^{\substack{\bullet\\\bullet\\\bullet\\\circ}}+\mathrm{P}_{\mathrm{S}}^{\substack{\bullet\\\bullet\\\circ\\\bullet}}+\mathrm{P}_{\mathrm{S}}^{\substack{\bullet\\\circ\\\bullet\\\bullet}}+\mathrm{P}_{\mathrm{S}}^{\substack{\circ\\\bullet\\\bullet\\\bullet}}\big)+\tfrac{1}{6}\big(\mathrm{P}_{\mathrm{S}}^{\substack{\bullet\\\bullet\\\circ\\\circ}}+\mathrm{P}_{\mathrm{S}}^{\substack{\bullet\\\circ\\\bullet\\\circ}}+\mathrm{P}_{\mathrm{S}}^{\substack{\bullet\\\circ\\\circ\\\bullet}}+\mathrm{P}_{\mathrm{S}}^{\substack{\circ\\\bullet\\\bullet\\\circ}}+\mathrm{P}_{\mathrm{S}}^{\substack{\circ\\\bullet\\\circ\\\bullet}}+\mathrm{P}_{\mathrm{S}}^{\substack{\circ\\\circ\\\bullet\\\bullet}}\big)\big)\mathrm{D}_{\mathrm{S}}^{\substack{\bullet\\\circ\\\bullet}}\Big)-\nu^{f}_{\mathrm{ATPsm}}\big(\mathrm{T}_{\mathrm{S}}^{\substack{\bullet\bullet\\\circ\circ\\\bullet\bullet}}\big)-\nu^{r}_{\mathrm{ASs}}\big((\mathrm{W}_{\mathrm{S}}^{\bullet}+\mathrm{W}_{\mathrm{S}}^{\circ})\mathrm{T}_{\mathrm{S}}^{\substack{\bullet\bullet\\\circ\circ\\\bullet\bullet}}\big)$$

$$\frac{d\mathrm{T}_{\mathrm{S}}^{\substack{\bullet\bullet\\\circ\bullet\\\bullet\circ}}}{dt}=\nu^{f}_{\mathrm{ASs}}\Big(\big(\tfrac{1}{4}\big(\mathrm{P}_{\mathrm{S}}^{\substack{\bullet\\\bullet\\\bullet\\\circ}}+\mathrm{P}_{\mathrm{S}}^{\substack{\bullet\\\bullet\\\circ\\\bullet}}+\mathrm{P}_{\mathrm{S}}^{\substack{\bullet\\\circ\\\bullet\\\bullet}}+\mathrm{P}_{\mathrm{S}}^{\substack{\circ\\\bullet\\\bullet\\\bullet}}\big)+\tfrac{1}{6}\big(\mathrm{P}_{\mathrm{S}}^{\substack{\bullet\\\bullet\\\circ\\\circ}}+\mathrm{P}_{\mathrm{S}}^{\substack{\bullet\\\circ\\\bullet\\\circ}}+\mathrm{P}_{\mathrm{S}}^{\substack{\bullet\\\circ\\\circ\\\bullet}}+\mathrm{P}_{\mathrm{S}}^{\substack{\circ\\\bullet\\\bullet\\\circ}}+\mathrm{P}_{\mathrm{S}}^{\substack{\circ\\\bullet\\\circ\\\bullet}}+\mathrm{P}_{\mathrm{S}}^{\substack{\circ\\\circ\\\bullet\\\bullet}}\big)\big)\mathrm{D}_{\mathrm{S}}^{\substack{\bullet\\\circ\\\bullet}}\Big)-\nu^{f}_{\mathrm{ATPsm}}\big(\mathrm{T}_{\mathrm{S}}^{\substack{\bullet\bullet\\\circ\bullet\\\bullet\circ}}\big)-\nu^{r}_{\mathrm{ASs}}\big((\mathrm{W}_{\mathrm{S}}^{\bullet}+\mathrm{W}_{\mathrm{S}}^{\circ})\mathrm{T}_{\mathrm{S}}^{\substack{\bullet\bullet\\\circ\bullet\\\bullet\circ}}\big)$$

$$\frac{d\mathrm{T}_{\mathrm{S}}^{\substack{\bullet\bullet\\\circ\bullet\\\bullet\bullet}}}{dt}=\nu^{f}_{\mathrm{ASs}}\Big(\big(\mathrm{P}_{\mathrm{S}}^{\substack{\bullet\\\bullet\\\bullet\\\bullet}}+\tfrac{1}{4}\big(\mathrm{P}_{\mathrm{S}}^{\substack{\bullet\\\bullet\\\bullet\\\circ}}+\mathrm{P}_{\mathrm{S}}^{\substack{\bullet\\\bullet\\\circ\\\bullet}}+\mathrm{P}_{\mathrm{S}}^{\substack{\bullet\\\circ\\\bullet\\\bullet}}+\mathrm{P}_{\mathrm{S}}^{\substack{\circ\\\bullet\\\bullet\\\bullet}}\big)\big)\mathrm{D}_{\mathrm{S}}^{\substack{\bullet\\\circ\\\bullet}}\Big)-\nu^{f}_{\mathrm{ATPsm}}\big(\mathrm{T}_{\mathrm{S}}^{\substack{\bullet\bullet\\\circ\bullet\\\bullet\bullet}}\big)-\nu^{r}_{\mathrm{ASs}}\big((\mathrm{W}_{\mathrm{S}}^{\bullet}+\mathrm{W}_{\mathrm{S}}^{\circ})\mathrm{T}_{\mathrm{S}}^{\substack{\bullet\bullet\\\circ\bullet\\\bullet\bullet}}\big)$$

$$\frac{d\mathrm{T}_{\mathrm{S}}^{\substack{\bullet\circ\\\bullet\circ\\\circ\circ}}}{dt}=\nu^{f}_{\mathrm{ASs}}\Big(\big(\mathrm{P}_{\mathrm{S}}^{\substack{\circ\\\circ\\\circ\\\circ}}+\tfrac{1}{4}\big(\mathrm{P}_{\mathrm{S}}^{\substack{\bullet\\\circ\\\circ\\\circ}}+\mathrm{P}_{\mathrm{S}}^{\substack{\circ\\\bullet\\\circ\\\circ}}+\mathrm{P}_{\mathrm{S}}^{\substack{\circ\\\circ\\\bullet\\\circ}}+\mathrm{P}_{\mathrm{S}}^{\substack{\circ\\\circ\\\circ\\\bullet}}\big)\big)\mathrm{D}_{\mathrm{S}}^{\substack{\bullet\\\bullet\\\circ}}\Big)-\nu^{f}_{\mathrm{ATPsm}}\big(\mathrm{T}_{\mathrm{S}}^{\substack{\bullet\circ\\\bullet\circ\\\circ\circ}}\big)-\nu^{r}_{\mathrm{ASs}}\big((\mathrm{W}_{\mathrm{S}}^{\bullet}+\mathrm{W}_{\mathrm{S}}^{\circ})\mathrm{T}_{\mathrm{S}}^{\substack{\bullet\circ\\\bullet\circ\\\circ\circ}}\big)$$

$$\frac{d\mathrm{T}_{\mathrm{S}}^{\substack{\bullet\circ\\\bullet\circ\\\circ\bullet}}}{dt}=\nu^{f}_{\mathrm{ASs}}\Big(\big(\tfrac{1}{4}\big(\mathrm{P}_{\mathrm{S}}^{\substack{\bullet\\\circ\\\circ\\\circ}}+\mathrm{P}_{\mathrm{S}}^{\substack{\circ\\\bullet\\\circ\\\circ}}+\mathrm{P}_{\mathrm{S}}^{\substack{\circ\\\circ\\\bullet\\\circ}}+\mathrm{P}_{\mathrm{S}}^{\substack{\circ\\\circ\\\circ\\\bullet}}\big)+\tfrac{1}{6}\big(\mathrm{P}_{\mathrm{S}}^{\substack{\bullet\\\bullet\\\circ\\\circ}}+\mathrm{P}_{\mathrm{S}}^{\substack{\bullet\\\circ\\\bullet\\\circ}}+\mathrm{P}_{\mathrm{S}}^{\substack{\bullet\\\circ\\\circ\\\bullet}}+\mathrm{P}_{\mathrm{S}}^{\substack{\circ\\\bullet\\\bullet\\\circ}}+\mathrm{P}_{\mathrm{S}}^{\substack{\circ\\\bullet\\\circ\\\bullet}}+\mathrm{P}_{\mathrm{S}}^{\substack{\circ\\\circ\\\bullet\\\bullet}}\big)\big)\mathrm{D}_{\mathrm{S}}^{\substack{\bullet\\\bullet\\\circ}}\Big)-\nu^{f}_{\mathrm{ATPsm}}\big(\mathrm{T}_{\mathrm{S}}^{\substack{\bullet\circ\\\bullet\circ\\\circ\bullet}}\big)-\nu^{r}_{\mathrm{ASs}}\big((\mathrm{W}_{\mathrm{S}}^{\bullet}+\mathrm{W}_{\mathrm{S}}^{\circ})\mathrm{T}_{\mathrm{S}}^{\substack{\bullet\circ\\\bullet\circ\\\circ\bullet}}\big)$$

$$\frac{d\mathrm{T}_{\mathrm{S}}^{\substack{\bullet\circ\\\bullet\bullet\\\circ\circ}}}{dt}=\nu^{f}_{\mathrm{ASs}}\Big(\big(\tfrac{1}{4}\big(\mathrm{P}_{\mathrm{S}}^{\substack{\bullet\\\circ\\\circ\\\circ}}+\mathrm{P}_{\mathrm{S}}^{\substack{\circ\\\bullet\\\circ\\\circ}}+\mathrm{P}_{\mathrm{S}}^{\substack{\circ\\\circ\\\bullet\\\circ}}+\mathrm{P}_{\mathrm{S}}^{\substack{\circ\\\circ\\\circ\\\bullet}}\big)+\tfrac{1}{6}\big(\mathrm{P}_{\mathrm{S}}^{\substack{\bullet\\\bullet\\\circ\\\circ}}+\mathrm{P}_{\mathrm{S}}^{\substack{\bullet\\\circ\\\bullet\\\circ}}+\mathrm{P}_{\mathrm{S}}^{\substack{\bullet\\\circ\\\circ\\\bullet}}+\mathrm{P}_{\mathrm{S}}^{\substack{\circ\\\bullet\\\bullet\\\circ}}+\mathrm{P}_{\mathrm{S}}^{\substack{\circ\\\bullet\\\circ\\\bullet}}+\mathrm{P}_{\mathrm{S}}^{\substack{\circ\\\circ\\\bullet\\\bullet}}\big)\big)\mathrm{D}_{\mathrm{S}}^{\substack{\bullet\\\bullet\\\circ}}\Big)-\nu^{f}_{\mathrm{ATPsm}}\big(\mathrm{T}_{\mathrm{S}}^{\substack{\bullet\circ\\\bullet\bullet\\\circ\circ}}\big)-\nu^{r}_{\mathrm{ASs}}\big((\mathrm{W}_{\mathrm{S}}^{\bullet}+\mathrm{W}_{\mathrm{S}}^{\circ})\mathrm{T}_{\mathrm{S}}^{\substack{\bullet\circ\\\bullet\bullet\\\circ\circ}}\big)$$

$$\frac{d\mathrm{T}_{\mathrm{S}}^{\substack{\bullet\circ\\\bullet\bullet\\\circ\bullet}}}{dt}=\nu^{f}_{\mathrm{ASs}}\Big(\big(\tfrac{1}{4}\big(\mathrm{P}_{\mathrm{S}}^{\substack{\bullet\\\bullet\\\bullet\\\circ}}+\mathrm{P}_{\mathrm{S}}^{\substack{\bullet\\\bullet\\\circ\\\bullet}}+\mathrm{P}_{\mathrm{S}}^{\substack{\bullet\\\circ\\\bullet\\\bullet}}+\mathrm{P}_{\mathrm{S}}^{\substack{\circ\\\bullet\\\bullet\\\bullet}}\big)+\tfrac{1}{6}\big(\mathrm{P}_{\mathrm{S}}^{\substack{\bullet\\\bullet\\\circ\\\circ}}+\mathrm{P}_{\mathrm{S}}^{\substack{\bullet\\\circ\\\bullet\\\circ}}+\mathrm{P}_{\mathrm{S}}^{\substack{\bullet\\\circ\\\circ\\\bullet}}+\mathrm{P}_{\mathrm{S}}^{\substack{\circ\\\bullet\\\bullet\\\circ}}+\mathrm{P}_{\mathrm{S}}^{\substack{\circ\\\bullet\\\circ\\\bullet}}+\mathrm{P}_{\mathrm{S}}^{\substack{\circ\\\circ\\\bullet\\\bullet}}\big)\big)\mathrm{D}_{\mathrm{S}}^{\substack{\bullet\\\bullet\\\circ}}\Big)-\nu^{f}_{\mathrm{ATPsm}}\big(\mathrm{T}_{\mathrm{S}}^{\substack{\bullet\circ\\\bullet\bullet\\\circ\bullet}}\big)-\nu^{r}_{\mathrm{ASs}}\big((\mathrm{W}_{\mathrm{S}}^{\bullet}+\mathrm{W}_{\mathrm{S}}^{\circ})\mathrm{T}_{\mathrm{S}}^{\substack{\bullet\circ\\\bullet\bullet\\\circ\bullet}}\big)$$

$$\frac{d\mathrm{T}_{\mathrm{S}}^{\substack{\bullet\bullet\\\bullet\circ\\\circ\circ}}}{dt}=\nu^{f}_{\mathrm{ASs}}\Big(\big(\tfrac{1}{4}\big(\mathrm{P}_{\mathrm{S}}^{\substack{\bullet\\\circ\\\circ\\\circ}}+\mathrm{P}_{\mathrm{S}}^{\substack{\circ\\\bullet\\\circ\\\circ}}+\mathrm{P}_{\mathrm{S}}^{\substack{\circ\\\circ\\\bullet\\\circ}}+\mathrm{P}_{\mathrm{S}}^{\substack{\circ\\\circ\\\circ\\\bullet}}\big)+\tfrac{1}{6}\big(\mathrm{P}_{\mathrm{S}}^{\substack{\bullet\\\bullet\\\circ\\\circ}}+\mathrm{P}_{\mathrm{S}}^{\substack{\bullet\\\circ\\\bullet\\\circ}}+\mathrm{P}_{\mathrm{S}}^{\substack{\bullet\\\circ\\\circ\\\bullet}}+\mathrm{P}_{\mathrm{S}}^{\substack{\circ\\\bullet\\\bullet\\\circ}}+\mathrm{P}_{\mathrm{S}}^{\substack{\circ\\\bullet\\\circ\\\bullet}}+\mathrm{P}_{\mathrm{S}}^{\substack{\circ\\\circ\\\bullet\\\bullet}}\big)\big)\mathrm{D}_{\mathrm{S}}^{\substack{\bullet\\\bullet\\\circ}}\Big)-\nu^{f}_{\mathrm{ATPsm}}\big(\mathrm{T}_{\mathrm{S}}^{\substack{\bullet\bullet\\\bullet\circ\\\circ\circ}}\big)-\nu^{r}_{\mathrm{ASs}}\big((\mathrm{W}_{\mathrm{S}}^{\bullet}+\mathrm{W}_{\mathrm{S}}^{\circ})\mathrm{T}_{\mathrm{S}}^{\substack{\bullet\bullet\\\bullet\circ\\\circ\circ}}\big)$$

$$\frac{d\mathrm{T}_{\mathrm{S}}^{\substack{\bullet\bullet\\\bullet\circ\\\circ\bullet}}}{dt}=\nu^{f}_{\mathrm{ASs}}\Big(\big(\tfrac{1}{4}\big(\mathrm{P}_{\mathrm{S}}^{\substack{\bullet\\\bullet\\\bullet\\\circ}}+\mathrm{P}_{\mathrm{S}}^{\substack{\bullet\\\bullet\\\circ\\\bullet}}+\mathrm{P}_{\mathrm{S}}^{\substack{\bullet\\\circ\\\bullet\\\bullet}}+\mathrm{P}_{\mathrm{S}}^{\substack{\circ\\\bullet\\\bullet\\\bullet}}\big)+\tfrac{1}{6}\big(\mathrm{P}_{\mathrm{S}}^{\substack{\bullet\\\bullet\\\circ\\\circ}}+\mathrm{P}_{\mathrm{S}}^{\substack{\bullet\\\circ\\\bullet\\\circ}}+\mathrm{P}_{\mathrm{S}}^{\substack{\bullet\\\circ\\\circ\\\bullet}}+\mathrm{P}_{\mathrm{S}}^{\substack{\circ\\\bullet\\\bullet\\\circ}}+\mathrm{P}_{\mathrm{S}}^{\substack{\circ\\\bullet\\\circ\\\bullet}}+\mathrm{P}_{\mathrm{S}}^{\substack{\circ\\\circ\\\bullet\\\bullet}}\big)\big)\mathrm{D}_{\mathrm{S}}^{\substack{\bullet\\\bullet\\\circ}}\Big)-\nu^{f}_{\mathrm{ATPsm}}\big(\mathrm{T}_{\mathrm{S}}^{\substack{\bullet\bullet\\\bullet\circ\\\circ\bullet}}\big)-\nu^{r}_{\mathrm{ASs}}\big((\mathrm{W}_{\mathrm{S}}^{\bullet}+\mathrm{W}_{\mathrm{S}}^{\circ})\mathrm{T}_{\mathrm{S}}^{\substack{\bullet\bullet\\\bullet\circ\\\circ\bullet}}\big)$$

$$\frac{d\mathrm{T}_{\mathrm{S}}^{\substack{\bullet\bullet\\\bullet\bullet\\\circ\circ}}}{dt}=\nu^{f}_{\mathrm{ASs}}\Big(\big(\tfrac{1}{4}\big(\mathrm{P}_{\mathrm{S}}^{\substack{\bullet\\\bullet\\\bullet\\\circ}}+\mathrm{P}_{\mathrm{S}}^{\substack{\bullet\\\bullet\\\circ\\\bullet}}+\mathrm{P}_{\mathrm{S}}^{\substack{\bullet\\\circ\\\bullet\\\bullet}}+\mathrm{P}_{\mathrm{S}}^{\substack{\circ\\\bullet\\\bullet\\\bullet}}\big)+\tfrac{1}{6}\big(\mathrm{P}_{\mathrm{S}}^{\substack{\bullet\\\bullet\\\circ\\\circ}}+\mathrm{P}_{\mathrm{S}}^{\substack{\bullet\\\circ\\\bullet\\\circ}}+\mathrm{P}_{\mathrm{S}}^{\substack{\bullet\\\circ\\\circ\\\bullet}}+\mathrm{P}_{\mathrm{S}}^{\substack{\circ\\\bullet\\\bullet\\\circ}}+\mathrm{P}_{\mathrm{S}}^{\substack{\circ\\\bullet\\\circ\\\bullet}}+\mathrm{P}_{\mathrm{S}}^{\substack{\circ\\\circ\\\bullet\\\bullet}}\big)\big)\mathrm{D}_{\mathrm{S}}^{\substack{\bullet\\\bullet\\\circ}}\Big)-\nu^{f}_{\mathrm{ATPsm}}\big(\mathrm{T}_{\mathrm{S}}^{\substack{\bullet\bullet\\\bullet\bullet\\\circ\circ}}\big)-\nu^{r}_{\mathrm{ASs}}\big((\mathrm{W}_{\mathrm{S}}^{\bullet}+\mathrm{W}_{\mathrm{S}}^{\circ})\mathrm{T}_{\mathrm{S}}^{\substack{\bullet\bullet\\\bullet\bullet\\\circ\circ}}\big)$$

$$\frac{d\mathrm{T}_{\mathrm{S}}^{\substack{\bullet\bullet\\\bullet\bullet\\\circ\bullet}}}{dt}=\nu^{f}_{\mathrm{ASs}}\Big(\big(\mathrm{P}_{\mathrm{S}}^{\substack{\bullet\\\bullet\\\bullet\\\bullet}}+\tfrac{1}{4}\big(\mathrm{P}_{\mathrm{S}}^{\substack{\bullet\\\bullet\\\bullet\\\circ}}+\mathrm{P}_{\mathrm{S}}^{\substack{\bullet\\\bullet\\\circ\\\bullet}}+\mathrm{P}_{\mathrm{S}}^{\substack{\bullet\\\circ\\\bullet\\\bullet}}+\mathrm{P}_{\mathrm{S}}^{\substack{\circ\\\bullet\\\bullet\\\bullet}}\big)\big)\mathrm{D}_{\mathrm{S}}^{\substack{\bullet\\\bullet\\\circ}}\Big)-\nu^{f}_{\mathrm{ATPsm}}\big(\mathrm{T}_{\mathrm{S}}^{\substack{\bullet\bullet\\\bullet\bullet\\\circ\bullet}}\big)-\nu^{r}_{\mathrm{ASs}}\big((\mathrm{W}_{\mathrm{S}}^{\bullet}+\mathrm{W}_{\mathrm{S}}^{\circ})\mathrm{T}_{\mathrm{S}}^{\substack{\bullet\bullet\\\bullet\bullet\\\circ\bullet}}\big)$$

$$\frac{d\mathrm{T}_{\mathrm{S}}^{\substack{\bullet\circ\\\bullet\circ\\\bullet\circ}}}{dt}=\nu^{f}_{\mathrm{ASs}}\Big(\big(\mathrm{P}_{\mathrm{S}}^{\substack{\circ\\\circ\\\circ\\\circ}}+\tfrac{1}{4}\big(\mathrm{P}_{\mathrm{S}}^{\substack{\bullet\\\circ\\\circ\\\circ}}+\mathrm{P}_{\mathrm{S}}^{\substack{\circ\\\bullet\\\circ\\\circ}}+\mathrm{P}_{\mathrm{S}}^{\substack{\circ\\\circ\\\bullet\\\circ}}+\mathrm{P}_{\mathrm{S}}^{\substack{\circ\\\circ\\\circ\\\bullet}}\big)\big)\mathrm{D}_{\mathrm{S}}^{\substack{\bullet\\\bullet\\\bullet}}\Big)-\nu^{f}_{\mathrm{ATPsm}}\big(\mathrm{T}_{\mathrm{S}}^{\substack{\bullet\circ\\\bullet\circ\\\bullet\circ}}\big)-\nu^{r}_{\mathrm{ASs}}\big((\mathrm{W}_{\mathrm{S}}^{\bullet}+\mathrm{W}_{\mathrm{S}}^{\circ})\mathrm{T}_{\mathrm{S}}^{\substack{\bullet\circ\\\bullet\circ\\\bullet\circ}}\big)$$

$$\frac{d\mathrm{T}_{\mathrm{S}}^{\substack{\bullet\circ\\\bullet\circ\\\bullet\bullet}}}{dt}=\nu^{f}_{\mathrm{ASs}}\Big(\big(\tfrac{1}{4}\big(\mathrm{P}_{\mathrm{S}}^{\substack{\bullet\\\circ\\\circ\\\circ}}+\mathrm{P}_{\mathrm{S}}^{\substack{\circ\\\bullet\\\circ\\\circ}}+\mathrm{P}_{\mathrm{S}}^{\substack{\circ\\\circ\\\bullet\\\circ}}+\mathrm{P}_{\mathrm{S}}^{\substack{\circ\\\circ\\\circ\\\bullet}}\big)+\tfrac{1}{6}\big(\mathrm{P}_{\mathrm{S}}^{\substack{\bullet\\\bullet\\\circ\\\circ}}+\mathrm{P}_{\mathrm{S}}^{\substack{\bullet\\\circ\\\bullet\\\circ}}+\mathrm{P}_{\mathrm{S}}^{\substack{\bullet\\\circ\\\circ\\\bullet}}+\mathrm{P}_{\mathrm{S}}^{\substack{\circ\\\bullet\\\bullet\\\circ}}+\mathrm{P}_{\mathrm{S}}^{\substack{\circ\\\bullet\\\circ\\\bullet}}+\mathrm{P}_{\mathrm{S}}^{\substack{\circ\\\circ\\\bullet\\\bullet}}\big)\big)\mathrm{D}_{\mathrm{S}}^{\substack{\bullet\\\bullet\\\bullet}}\Big)-\nu^{f}_{\mathrm{ATPsm}}\big(\mathrm{T}_{\mathrm{S}}^{\substack{\bullet\circ\\\bullet\circ\\\bullet\bullet}}\big)-\nu^{r}_{\mathrm{ASs}}\big((\mathrm{W}_{\mathrm{S}}^{\bullet}+\mathrm{W}_{\mathrm{S}}^{\circ})\mathrm{T}_{\mathrm{S}}^{\substack{\bullet\circ\\\bullet\circ\\\bullet\bullet}}\big)$$

$$\frac{d\mathrm{T}_{\mathrm{S}}^{\substack{\bullet\circ\\\bullet\bullet\\\bullet\circ}}}{dt}=\nu^{f}_{\mathrm{ASs}}\Big(\big(\tfrac{1}{4}\big(\mathrm{P}_{\mathrm{S}}^{\substack{\bullet\\\circ\\\circ\\\circ}}+\mathrm{P}_{\mathrm{S}}^{\substack{\circ\\\bullet\\\circ\\\circ}}+\mathrm{P}_{\mathrm{S}}^{\substack{\circ\\\circ\\\bullet\\\circ}}+\mathrm{P}_{\mathrm{S}}^{\substack{\circ\\\circ\\\circ\\\bullet}}\big)+\tfrac{1}{6}\big(\mathrm{P}_{\mathrm{S}}^{\substack{\bullet\\\bullet\\\circ\\\circ}}+\mathrm{P}_{\mathrm{S}}^{\substack{\bullet\\\circ\\\bullet\\\circ}}+\mathrm{P}_{\mathrm{S}}^{\substack{\bullet\\\circ\\\circ\\\bullet}}+\mathrm{P}_{\mathrm{S}}^{\substack{\circ\\\bullet\\\bullet\\\circ}}+\mathrm{P}_{\mathrm{S}}^{\substack{\circ\\\bullet\\\circ\\\bullet}}+\mathrm{P}_{\mathrm{S}}^{\substack{\circ\\\circ\\\bullet\\\bullet}}\big)\big)\mathrm{D}_{\mathrm{S}}^{\substack{\bullet\\\bullet\\\bullet}}\Big)-\nu^{f}_{\mathrm{ATPsm}}\big(\mathrm{T}_{\mathrm{S}}^{\substack{\bullet\circ\\\bullet\bullet\\\bullet\circ}}\big)-\nu^{r}_{\mathrm{ASs}}\big((\mathrm{W}_{\mathrm{S}}^{\bullet}+\mathrm{W}_{\mathrm{S}}^{\circ})\mathrm{T}_{\mathrm{S}}^{\substack{\bullet\circ\\\bullet\bullet\\\bullet\circ}}\big)$$

$$\frac{d\mathrm{T}_{\mathrm{S}}^{\substack{\bullet\circ\\\bullet\bullet\\\bullet\bullet}}}{dt}=\nu^{f}_{\mathrm{ASs}}\Big(\big(\tfrac{1}{4}\big(\mathrm{P}_{\mathrm{S}}^{\substack{\bullet\\\bullet\\\bullet\\\circ}}+\mathrm{P}_{\mathrm{S}}^{\substack{\bullet\\\bullet\\\circ\\\bullet}}+\mathrm{P}_{\mathrm{S}}^{\substack{\bullet\\\circ\\\bullet\\\bullet}}+\mathrm{P}_{\mathrm{S}}^{\substack{\circ\\\bullet\\\bullet\\\bullet}}\big)+\tfrac{1}{6}\big(\mathrm{P}_{\mathrm{S}}^{\substack{\bullet\\\bullet\\\circ\\\circ}}+\mathrm{P}_{\mathrm{S}}^{\substack{\bullet\\\circ\\\bullet\\\circ}}+\mathrm{P}_{\mathrm{S}}^{\substack{\bullet\\\circ\\\circ\\\bullet}}+\mathrm{P}_{\mathrm{S}}^{\substack{\circ\\\bullet\\\bullet\\\circ}}+\mathrm{P}_{\mathrm{S}}^{\substack{\circ\\\bullet\\\circ\\\bullet}}+\mathrm{P}_{\mathrm{S}}^{\substack{\circ\\\circ\\\bullet\\\bullet}}\big)\big)\mathrm{D}_{\mathrm{S}}^{\substack{\bullet\\\bullet\\\bullet}}\Big)-\nu^{f}_{\mathrm{ATPsm}}\big(\mathrm{T}_{\mathrm{S}}^{\substack{\bullet\circ\\\bullet\bullet\\\bullet\bullet}}\big)-\nu^{r}_{\mathrm{ASs}}\big((\mathrm{W}_{\mathrm{S}}^{\bullet}+\mathrm{W}_{\mathrm{S}}^{\circ})\mathrm{T}_{\mathrm{S}}^{\substack{\bullet\circ\\\bullet\bullet\\\bullet\bullet}}\big)$$

$$\frac{d\mathrm{T}_{\mathrm{S}}^{\substack{\bullet\bullet\\\bullet\circ\\\bullet\circ}}}{dt} = \underset{\mathrm{ASs}}{\nu_f}\Big(\Big(\tfrac{1}{4}\big(\mathrm{P}_{\mathrm{S}}^{\substack{\bullet\\\circ\\\circ\\\circ}} + \mathrm{P}_{\mathrm{S}}^{\substack{\circ\\\bullet\\\circ\\\circ}} + \mathrm{P}_{\mathrm{S}}^{\substack{\circ\\\circ\\\bullet\\\circ}} + \mathrm{P}_{\mathrm{S}}^{\substack{\circ\\\circ\\\circ\\\bullet}}\big) + \tfrac{1}{6}\big(\mathrm{P}_{\mathrm{S}}^{\substack{\bullet\\\bullet\\\circ\\\circ}} + \mathrm{P}_{\mathrm{S}}^{\substack{\bullet\\\circ\\\bullet\\\circ}} + \mathrm{P}_{\mathrm{S}}^{\substack{\bullet\\\circ\\\circ\\\bullet}} + \mathrm{P}_{\mathrm{S}}^{\substack{\circ\\\bullet\\\bullet\\\circ}} + \mathrm{P}_{\mathrm{S}}^{\substack{\circ\\\bullet\\\circ\\\bullet}} + \mathrm{P}_{\mathrm{S}}^{\substack{\circ\\\circ\\\bullet\\\bullet}}\big)\Big)\mathrm{D}_{\mathrm{S}}^{\substack{\bullet\\\bullet\\\bullet}}\Big) - \underset{\mathrm{ATPsm}}{\nu_f}\big(\mathrm{T}_{\mathrm{S}}^{\substack{\bullet\bullet\\\bullet\circ\\\bullet\circ}}\big) - \underset{\mathrm{ASs}}{\nu_r}\big((\mathrm{W}_{\mathrm{S}}^{\bullet} + \mathrm{W}_{\mathrm{S}}^{\circ})\mathrm{T}_{\mathrm{S}}^{\substack{\bullet\bullet\\\bullet\circ\\\bullet\circ}}\big)$$

$$\frac{d\mathrm{T}_{\mathrm{S}}^{\substack{\bullet\bullet\\\bullet\circ\\\bullet\bullet}}}{dt} = \underset{\mathrm{ASs}}{\nu_f}\Big(\Big(\tfrac{1}{4}\big(\mathrm{P}_{\mathrm{S}}^{\substack{\bullet\\\bullet\\\bullet\\\circ}} + \mathrm{P}_{\mathrm{S}}^{\substack{\bullet\\\bullet\\\circ\\\bullet}} + \mathrm{P}_{\mathrm{S}}^{\substack{\bullet\\\circ\\\bullet\\\bullet}} + \mathrm{P}_{\mathrm{S}}^{\substack{\circ\\\bullet\\\bullet\\\bullet}}\big) + \tfrac{1}{6}\big(\mathrm{P}_{\mathrm{S}}^{\substack{\bullet\\\bullet\\\circ\\\circ}} + \mathrm{P}_{\mathrm{S}}^{\substack{\bullet\\\circ\\\bullet\\\circ}} + \mathrm{P}_{\mathrm{S}}^{\substack{\bullet\\\circ\\\circ\\\bullet}} + \mathrm{P}_{\mathrm{S}}^{\substack{\circ\\\bullet\\\bullet\\\circ}} + \mathrm{P}_{\mathrm{S}}^{\substack{\circ\\\bullet\\\circ\\\bullet}} + \mathrm{P}_{\mathrm{S}}^{\substack{\circ\\\circ\\\bullet\\\bullet}}\big)\Big)\mathrm{D}_{\mathrm{S}}^{\substack{\bullet\\\bullet\\\bullet}}\Big) - \underset{\mathrm{ATPsm}}{\nu_f}\big(\mathrm{T}_{\mathrm{S}}^{\substack{\bullet\bullet\\\bullet\circ\\\bullet\bullet}}\big) - \underset{\mathrm{ASs}}{\nu_r}\big((\mathrm{W}_{\mathrm{S}}^{\bullet} + \mathrm{W}_{\mathrm{S}}^{\circ})\mathrm{T}_{\mathrm{S}}^{\substack{\bullet\bullet\\\bullet\circ\\\bullet\bullet}}\big)$$

$$\frac{d\mathrm{T}_{\mathrm{S}}^{\substack{\bullet\bullet\\\bullet\bullet\\\bullet\circ}}}{dt} = \underset{\mathrm{ASs}}{\nu_f}\Big(\Big(\tfrac{1}{4}\big(\mathrm{P}_{\mathrm{S}}^{\substack{\bullet\\\bullet\\\bullet\\\circ}} + \mathrm{P}_{\mathrm{S}}^{\substack{\bullet\\\bullet\\\circ\\\bullet}} + \mathrm{P}_{\mathrm{S}}^{\substack{\bullet\\\circ\\\bullet\\\bullet}} + \mathrm{P}_{\mathrm{S}}^{\substack{\circ\\\bullet\\\bullet\\\bullet}}\big) + \tfrac{1}{6}\big(\mathrm{P}_{\mathrm{S}}^{\substack{\bullet\\\bullet\\\circ\\\circ}} + \mathrm{P}_{\mathrm{S}}^{\substack{\bullet\\\circ\\\bullet\\\circ}} + \mathrm{P}_{\mathrm{S}}^{\substack{\bullet\\\circ\\\circ\\\bullet}} + \mathrm{P}_{\mathrm{S}}^{\substack{\circ\\\bullet\\\bullet\\\circ}} + \mathrm{P}_{\mathrm{S}}^{\substack{\circ\\\bullet\\\circ\\\bullet}} + \mathrm{P}_{\mathrm{S}}^{\substack{\circ\\\circ\\\bullet\\\bullet}}\big)\Big)\mathrm{D}_{\mathrm{S}}^{\substack{\bullet\\\bullet\\\bullet}}\Big) - \underset{\mathrm{ATPsm}}{\nu_f}\big(\mathrm{T}_{\mathrm{S}}^{\substack{\bullet\bullet\\\bullet\bullet\\\bullet\circ}}\big) - \underset{\mathrm{ASs}}{\nu_r}\big((\mathrm{W}_{\mathrm{S}}^{\bullet} + \mathrm{W}_{\mathrm{S}}^{\circ})\mathrm{T}_{\mathrm{S}}^{\substack{\bullet\bullet\\\bullet\bullet\\\bullet\circ}}\big)$$

$$\frac{d\mathrm{T}_{\mathrm{S}}^{\substack{\bullet\bullet\\\bullet\bullet\\\bullet\bullet}}}{dt} = \underset{\mathrm{ASs}}{\nu_f}\Big(\big(\mathrm{P}_{\mathrm{S}}^{\substack{\bullet\\\bullet\\\bullet\\\bullet}} + \tfrac{1}{4}\big(\mathrm{P}_{\mathrm{S}}^{\substack{\bullet\\\bullet\\\bullet\\\circ}} + \mathrm{P}_{\mathrm{S}}^{\substack{\bullet\\\bullet\\\circ\\\bullet}} + \mathrm{P}_{\mathrm{S}}^{\substack{\bullet\\\circ\\\bullet\\\bullet}} + \mathrm{P}_{\mathrm{S}}^{\substack{\circ\\\bullet\\\bullet\\\bullet}}\big)\big)\mathrm{D}_{\mathrm{S}}^{\substack{\bullet\\\bullet\\\bullet}}\Big) - \underset{\mathrm{ATPsm}}{\nu_f}\big(\mathrm{T}_{\mathrm{S}}^{\substack{\bullet\bullet\\\bullet\bullet\\\bullet\bullet}}\big) - \underset{\mathrm{ASs}}{\nu_r}\big((\mathrm{W}_{\mathrm{S}}^{\bullet} + \mathrm{W}_{\mathrm{S}}^{\circ})\mathrm{T}_{\mathrm{S}}^{\substack{\bullet\bullet\\\bullet\bullet\\\bullet\bullet}}\big)$$

$$\frac{d\mathrm{C}_{\mathrm{I}}^{\substack{\circ\\\circ\\\circ}}}{dt} = \underset{\mathrm{CKi}}{\nu_f}\big(\mathrm{T}_{\mathrm{I}}^{\substack{\bullet\circ\\\bullet\circ\\\bullet\circ}} + \mathrm{T}_{\mathrm{I}}^{\substack{\bullet\circ\\\bullet\circ\\\circ\circ}} + \mathrm{T}_{\mathrm{I}}^{\substack{\bullet\circ\\\circ\circ\\\bullet\circ}} + \mathrm{T}_{\mathrm{I}}^{\substack{\bullet\circ\\\circ\circ\\\circ\circ}} + \mathrm{T}_{\mathrm{I}}^{\substack{\circ\circ\\\bullet\circ\\\bullet\circ}} + \mathrm{T}_{\mathrm{I}}^{\substack{\circ\circ\\\bullet\circ\\\circ\circ}} + \mathrm{T}_{\mathrm{I}}^{\substack{\circ\circ\\\circ\circ\\\bullet\circ}} + \mathrm{T}_{\mathrm{I}}^{\substack{\circ\circ\\\circ\circ\\\circ\circ}}\big) + \underset{\mathrm{CIo}}{\nu_r}\big(\mathrm{C}_{\mathrm{O}}^{\substack{\circ\\\circ\\\circ}}\big) - \underset{\mathrm{CIo}}{\nu_f}\big(\mathrm{C}_{\mathrm{I}}^{\substack{\circ\\\circ\\\circ}}\big) - \underset{\mathrm{CKi}}{\nu_r}\Big(\big(\mathrm{D}_{\mathrm{I}}^{\substack{\bullet\\\bullet\\\bullet}} + \mathrm{D}_{\mathrm{I}}^{\substack{\bullet\\\bullet\\\circ}} + \mathrm{D}_{\mathrm{I}}^{\substack{\bullet\\\circ\\\bullet}} + \mathrm{D}_{\mathrm{I}}^{\substack{\bullet\\\circ\\\circ}} + \mathrm{D}_{\mathrm{I}}^{\substack{\circ\\\bullet\\\bullet}} + \mathrm{D}_{\mathrm{I}}^{\substack{\circ\\\bullet\\\circ}} + \mathrm{D}_{\mathrm{I}}^{\substack{\circ\\\circ\\\bullet}} + \mathrm{D}_{\mathrm{I}}^{\substack{\circ\\\circ\\\circ}}\big)\mathrm{C}_{\mathrm{I}}^{\substack{\circ\\\circ\\\circ}}\Big)$$

$$\frac{d\mathrm{C}_{\mathrm{I}}^{\substack{\circ\\\circ\\\bullet}}}{dt} = \underset{\mathrm{CKi}}{\nu_f}\big(\mathrm{T}_{\mathrm{I}}^{\substack{\bullet\circ\\\bullet\circ\\\bullet\bullet}} + \mathrm{T}_{\mathrm{I}}^{\substack{\bullet\circ\\\bullet\circ\\\circ\bullet}} + \mathrm{T}_{\mathrm{I}}^{\substack{\bullet\circ\\\circ\circ\\\bullet\bullet}} + \mathrm{T}_{\mathrm{I}}^{\substack{\bullet\circ\\\circ\circ\\\circ\bullet}} + \mathrm{T}_{\mathrm{I}}^{\substack{\circ\circ\\\bullet\circ\\\bullet\bullet}} + \mathrm{T}_{\mathrm{I}}^{\substack{\circ\circ\\\bullet\circ\\\circ\bullet}} + \mathrm{T}_{\mathrm{I}}^{\substack{\circ\circ\\\circ\circ\\\bullet\bullet}} + \mathrm{T}_{\mathrm{I}}^{\substack{\circ\circ\\\circ\circ\\\circ\bullet}}\big) + \underset{\mathrm{CIo}}{\nu_r}\big(\mathrm{C}_{\mathrm{O}}^{\substack{\circ\\\circ\\\bullet}}\big) - \underset{\mathrm{CIo}}{\nu_f}\big(\mathrm{C}_{\mathrm{I}}^{\substack{\circ\\\circ\\\bullet}}\big) - \underset{\mathrm{CKi}}{\nu_r}\Big(\big(\mathrm{D}_{\mathrm{I}}^{\substack{\bullet\\\bullet\\\bullet}} + \mathrm{D}_{\mathrm{I}}^{\substack{\bullet\\\bullet\\\circ}} + \mathrm{D}_{\mathrm{I}}^{\substack{\bullet\\\circ\\\bullet}} + \mathrm{D}_{\mathrm{I}}^{\substack{\bullet\\\circ\\\circ}} + \mathrm{D}_{\mathrm{I}}^{\substack{\circ\\\bullet\\\bullet}} + \mathrm{D}_{\mathrm{I}}^{\substack{\circ\\\bullet\\\circ}} + \mathrm{D}_{\mathrm{I}}^{\substack{\circ\\\circ\\\bullet}} + \mathrm{D}_{\mathrm{I}}^{\substack{\circ\\\circ\\\circ}}\big)\mathrm{C}_{\mathrm{I}}^{\substack{\circ\\\circ\\\bullet}}\Big)$$

$$\frac{d\mathrm{C}_{\mathrm{I}}^{\substack{\circ\\\bullet\\\circ}}}{dt} = \underset{\mathrm{CKi}}{\nu_f}\big(\mathrm{T}_{\mathrm{I}}^{\substack{\bullet\circ\\\bullet\bullet\\\bullet\circ}} + \mathrm{T}_{\mathrm{I}}^{\substack{\bullet\circ\\\bullet\bullet\\\circ\circ}} + \mathrm{T}_{\mathrm{I}}^{\substack{\bullet\circ\\\circ\bullet\\\bullet\circ}} + \mathrm{T}_{\mathrm{I}}^{\substack{\bullet\circ\\\circ\bullet\\\circ\circ}} + \mathrm{T}_{\mathrm{I}}^{\substack{\circ\circ\\\bullet\bullet\\\bullet\circ}} + \mathrm{T}_{\mathrm{I}}^{\substack{\circ\circ\\\bullet\bullet\\\circ\circ}} + \mathrm{T}_{\mathrm{I}}^{\substack{\circ\circ\\\circ\bullet\\\bullet\circ}} + \mathrm{T}_{\mathrm{I}}^{\substack{\circ\circ\\\circ\bullet\\\circ\circ}}\big) + \underset{\mathrm{CIo}}{\nu_r}\big(\mathrm{C}_{\mathrm{O}}^{\substack{\circ\\\bullet\\\circ}}\big) - \underset{\mathrm{CIo}}{\nu_f}\big(\mathrm{C}_{\mathrm{I}}^{\substack{\circ\\\bullet\\\circ}}\big) - \underset{\mathrm{CKi}}{\nu_r}\Big(\big(\mathrm{D}_{\mathrm{I}}^{\substack{\bullet\\\bullet\\\bullet}} + \mathrm{D}_{\mathrm{I}}^{\substack{\bullet\\\bullet\\\circ}} + \mathrm{D}_{\mathrm{I}}^{\substack{\bullet\\\circ\\\bullet}} + \mathrm{D}_{\mathrm{I}}^{\substack{\bullet\\\circ\\\circ}} + \mathrm{D}_{\mathrm{I}}^{\substack{\circ\\\bullet\\\bullet}} + \mathrm{D}_{\mathrm{I}}^{\substack{\circ\\\bullet\\\circ}} + \mathrm{D}_{\mathrm{I}}^{\substack{\circ\\\circ\\\bullet}} + \mathrm{D}_{\mathrm{I}}^{\substack{\circ\\\circ\\\circ}}\big)\mathrm{C}_{\mathrm{I}}^{\substack{\circ\\\bullet\\\circ}}\Big)$$

$$\frac{d\mathrm{C}_{\mathrm{I}}^{\substack{\circ\\\bullet\\\bullet}}}{dt} = \underset{\mathrm{CKi}}{\nu_f}\big(\mathrm{T}_{\mathrm{I}}^{\substack{\bullet\circ\\\bullet\bullet\\\bullet\bullet}} + \mathrm{T}_{\mathrm{I}}^{\substack{\bullet\circ\\\bullet\bullet\\\circ\bullet}} + \mathrm{T}_{\mathrm{I}}^{\substack{\bullet\circ\\\circ\bullet\\\bullet\bullet}} + \mathrm{T}_{\mathrm{I}}^{\substack{\bullet\circ\\\circ\bullet\\\circ\bullet}} + \mathrm{T}_{\mathrm{I}}^{\substack{\circ\circ\\\bullet\bullet\\\bullet\bullet}} + \mathrm{T}_{\mathrm{I}}^{\substack{\circ\circ\\\bullet\bullet\\\circ\bullet}} + \mathrm{T}_{\mathrm{I}}^{\substack{\circ\circ\\\circ\bullet\\\bullet\bullet}} + \mathrm{T}_{\mathrm{I}}^{\substack{\circ\circ\\\circ\bullet\\\circ\bullet}}\big) + \underset{\mathrm{CIo}}{\nu_r}\big(\mathrm{C}_{\mathrm{O}}^{\substack{\circ\\\bullet\\\bullet}}\big) - \underset{\mathrm{CIo}}{\nu_f}\big(\mathrm{C}_{\mathrm{I}}^{\substack{\circ\\\bullet\\\bullet}}\big) - \underset{\mathrm{CKi}}{\nu_r}\Big(\big(\mathrm{D}_{\mathrm{I}}^{\substack{\bullet\\\bullet\\\bullet}} + \mathrm{D}_{\mathrm{I}}^{\substack{\bullet\\\bullet\\\circ}} + \mathrm{D}_{\mathrm{I}}^{\substack{\bullet\\\circ\\\bullet}} + \mathrm{D}_{\mathrm{I}}^{\substack{\bullet\\\circ\\\circ}} + \mathrm{D}_{\mathrm{I}}^{\substack{\circ\\\bullet\\\bullet}} + \mathrm{D}_{\mathrm{I}}^{\substack{\circ\\\bullet\\\circ}} + \mathrm{D}_{\mathrm{I}}^{\substack{\circ\\\circ\\\bullet}} + \mathrm{D}_{\mathrm{I}}^{\substack{\circ\\\circ\\\circ}}\big)\mathrm{C}_{\mathrm{I}}^{\substack{\circ\\\bullet\\\bullet}}\Big)$$

$$\frac{d\mathrm{C}_{\mathrm{I}}^{\substack{\bullet\\\circ\\\circ}}}{dt} = \underset{\mathrm{CKi}}{\nu_f}\big(\mathrm{T}_{\mathrm{I}}^{\substack{\bullet\bullet\\\bullet\circ\\\bullet\circ}} + \mathrm{T}_{\mathrm{I}}^{\substack{\bullet\bullet\\\bullet\circ\\\circ\circ}} + \mathrm{T}_{\mathrm{I}}^{\substack{\bullet\bullet\\\circ\circ\\\bullet\circ}} + \mathrm{T}_{\mathrm{I}}^{\substack{\bullet\bullet\\\circ\circ\\\circ\circ}} + \mathrm{T}_{\mathrm{I}}^{\substack{\circ\bullet\\\bullet\circ\\\bullet\circ}} + \mathrm{T}_{\mathrm{I}}^{\substack{\circ\bullet\\\bullet\circ\\\circ\circ}} + \mathrm{T}_{\mathrm{I}}^{\substack{\circ\bullet\\\circ\circ\\\bullet\circ}} + \mathrm{T}_{\mathrm{I}}^{\substack{\circ\bullet\\\circ\circ\\\circ\circ}}\big) + \underset{\mathrm{CIo}}{\nu_r}\big(\mathrm{C}_{\mathrm{O}}^{\substack{\bullet\\\circ\\\circ}}\big) - \underset{\mathrm{CIo}}{\nu_f}\big(\mathrm{C}_{\mathrm{I}}^{\substack{\bullet\\\circ\\\circ}}\big) - \underset{\mathrm{CKi}}{\nu_r}\Big(\big(\mathrm{D}_{\mathrm{I}}^{\substack{\bullet\\\bullet\\\bullet}} + \mathrm{D}_{\mathrm{I}}^{\substack{\bullet\\\bullet\\\circ}} + \mathrm{D}_{\mathrm{I}}^{\substack{\bullet\\\circ\\\bullet}} + \mathrm{D}_{\mathrm{I}}^{\substack{\bullet\\\circ\\\circ}} + \mathrm{D}_{\mathrm{I}}^{\substack{\circ\\\bullet\\\bullet}} + \mathrm{D}_{\mathrm{I}}^{\substack{\circ\\\bullet\\\circ}} + \mathrm{D}_{\mathrm{I}}^{\substack{\circ\\\circ\\\bullet}} + \mathrm{D}_{\mathrm{I}}^{\substack{\circ\\\circ\\\circ}}\big)\mathrm{C}_{\mathrm{I}}^{\substack{\bullet\\\circ\\\circ}}\Big)$$

$$\frac{d\mathrm{C}_{\mathrm{I}}^{\substack{\bullet\\\circ\\\bullet}}}{dt} = \underset{\mathrm{CKi}}{\nu_f}\big(\mathrm{T}_{\mathrm{I}}^{\substack{\bullet\bullet\\\bullet\circ\\\bullet\bullet}} + \mathrm{T}_{\mathrm{I}}^{\substack{\bullet\bullet\\\bullet\circ\\\circ\bullet}} + \mathrm{T}_{\mathrm{I}}^{\substack{\bullet\bullet\\\circ\circ\\\bullet\bullet}} + \mathrm{T}_{\mathrm{I}}^{\substack{\bullet\bullet\\\circ\circ\\\circ\bullet}} + \mathrm{T}_{\mathrm{I}}^{\substack{\circ\bullet\\\bullet\circ\\\bullet\bullet}} + \mathrm{T}_{\mathrm{I}}^{\substack{\circ\bullet\\\bullet\circ\\\circ\bullet}} + \mathrm{T}_{\mathrm{I}}^{\substack{\circ\bullet\\\circ\circ\\\bullet\bullet}} + \mathrm{T}_{\mathrm{I}}^{\substack{\circ\bullet\\\circ\circ\\\circ\bullet}}\big) + \underset{\mathrm{CIo}}{\nu_r}\big(\mathrm{C}_{\mathrm{O}}^{\substack{\bullet\\\circ\\\bullet}}\big) - \underset{\mathrm{CIo}}{\nu_f}\big(\mathrm{C}_{\mathrm{I}}^{\substack{\bullet\\\circ\\\bullet}}\big) - \underset{\mathrm{CKi}}{\nu_r}\Big(\big(\mathrm{D}_{\mathrm{I}}^{\substack{\bullet\\\bullet\\\bullet}} + \mathrm{D}_{\mathrm{I}}^{\substack{\bullet\\\bullet\\\circ}} + \mathrm{D}_{\mathrm{I}}^{\substack{\bullet\\\circ\\\bullet}} + \mathrm{D}_{\mathrm{I}}^{\substack{\bullet\\\circ\\\circ}} + \mathrm{D}_{\mathrm{I}}^{\substack{\circ\\\bullet\\\bullet}} + \mathrm{D}_{\mathrm{I}}^{\substack{\circ\\\bullet\\\circ}} + \mathrm{D}_{\mathrm{I}}^{\substack{\circ\\\circ\\\bullet}} + \mathrm{D}_{\mathrm{I}}^{\substack{\circ\\\circ\\\circ}}\big)\mathrm{C}_{\mathrm{I}}^{\substack{\bullet\\\circ\\\bullet}}\Big)$$

$$\frac{d\mathrm{C}_{\mathrm{I}}^{\substack{\bullet\\\bullet\\\circ}}}{dt} = \underset{\mathrm{CKi}}{\nu_f}\big(\mathrm{T}_{\mathrm{I}}^{\substack{\bullet\bullet\\\bullet\bullet\\\bullet\circ}} + \mathrm{T}_{\mathrm{I}}^{\substack{\bullet\bullet\\\bullet\bullet\\\circ\circ}} + \mathrm{T}_{\mathrm{I}}^{\substack{\bullet\bullet\\\circ\bullet\\\bullet\circ}} + \mathrm{T}_{\mathrm{I}}^{\substack{\bullet\bullet\\\circ\bullet\\\circ\circ}} + \mathrm{T}_{\mathrm{I}}^{\substack{\circ\bullet\\\bullet\bullet\\\bullet\circ}} + \mathrm{T}_{\mathrm{I}}^{\substack{\circ\bullet\\\bullet\bullet\\\circ\circ}} + \mathrm{T}_{\mathrm{I}}^{\substack{\circ\bullet\\\circ\bullet\\\bullet\circ}} + \mathrm{T}_{\mathrm{I}}^{\substack{\circ\bullet\\\circ\bullet\\\circ\circ}}\big) + \underset{\mathrm{CIo}}{\nu_r}\big(\mathrm{C}_{\mathrm{O}}^{\substack{\bullet\\\bullet\\\circ}}\big) - \underset{\mathrm{CIo}}{\nu_f}\big(\mathrm{C}_{\mathrm{I}}^{\substack{\bullet\\\bullet\\\circ}}\big) - \underset{\mathrm{CKi}}{\nu_r}\Big(\big(\mathrm{D}_{\mathrm{I}}^{\substack{\bullet\\\bullet\\\bullet}} + \mathrm{D}_{\mathrm{I}}^{\substack{\bullet\\\bullet\\\circ}} + \mathrm{D}_{\mathrm{I}}^{\substack{\bullet\\\circ\\\bullet}} + \mathrm{D}_{\mathrm{I}}^{\substack{\bullet\\\circ\\\circ}} + \mathrm{D}_{\mathrm{I}}^{\substack{\circ\\\bullet\\\bullet}} + \mathrm{D}_{\mathrm{I}}^{\substack{\circ\\\bullet\\\circ}} + \mathrm{D}_{\mathrm{I}}^{\substack{\circ\\\circ\\\bullet}} + \mathrm{D}_{\mathrm{I}}^{\substack{\circ\\\circ\\\circ}}\big)\mathrm{C}_{\mathrm{I}}^{\substack{\bullet\\\bullet\\\circ}}\Big)$$

$$\frac{d\mathrm{C}_{\mathrm{I}}^{\substack{\bullet\\\bullet\\\bullet}}}{dt} = \underset{\mathrm{CKi}}{\nu_f}\big(\mathrm{T}_{\mathrm{I}}^{\substack{\bullet\bullet\\\bullet\bullet\\\bullet\bullet}} + \mathrm{T}_{\mathrm{I}}^{\substack{\bullet\bullet\\\bullet\bullet\\\circ\bullet}} + \mathrm{T}_{\mathrm{I}}^{\substack{\bullet\bullet\\\circ\bullet\\\bullet\bullet}} + \mathrm{T}_{\mathrm{I}}^{\substack{\bullet\bullet\\\circ\bullet\\\circ\bullet}} + \mathrm{T}_{\mathrm{I}}^{\substack{\circ\bullet\\\bullet\bullet\\\bullet\bullet}} + \mathrm{T}_{\mathrm{I}}^{\substack{\circ\bullet\\\bullet\bullet\\\circ\bullet}} + \mathrm{T}_{\mathrm{I}}^{\substack{\circ\bullet\\\circ\bullet\\\bullet\bullet}} + \mathrm{T}_{\mathrm{I}}^{\substack{\circ\bullet\\\circ\bullet\\\circ\bullet}}\big) + \underset{\mathrm{CIo}}{\nu_r}\big(\mathrm{C}_{\mathrm{O}}^{\substack{\bullet\\\bullet\\\bullet}}\big) - \underset{\mathrm{CIo}}{\nu_f}\big(\mathrm{C}_{\mathrm{I}}^{\substack{\bullet\\\bullet\\\bullet}}\big) - \underset{\mathrm{CKi}}{\nu_r}\Big(\big(\mathrm{D}_{\mathrm{I}}^{\substack{\bullet\\\bullet\\\bullet}} + \mathrm{D}_{\mathrm{I}}^{\substack{\bullet\\\bullet\\\circ}} + \mathrm{D}_{\mathrm{I}}^{\substack{\bullet\\\circ\\\bullet}} + \mathrm{D}_{\mathrm{I}}^{\substack{\bullet\\\circ\\\circ}} + \mathrm{D}_{\mathrm{I}}^{\substack{\circ\\\bullet\\\bullet}} + \mathrm{D}_{\mathrm{I}}^{\substack{\circ\\\bullet\\\circ}} + \mathrm{D}_{\mathrm{I}}^{\substack{\circ\\\circ\\\bullet}} + \mathrm{D}_{\mathrm{I}}^{\substack{\circ\\\circ\\\circ}}\big)\mathrm{C}_{\mathrm{I}}^{\substack{\bullet\\\bullet\\\bullet}}\Big)$$

$$\frac{d\mathrm{C}_{\mathrm{O}}^{\substack{\circ\\\circ\\\circ}}}{dt} = \underset{\mathrm{CKo}}{\nu_f}\big(\mathrm{T}_{\mathrm{O}}^{\substack{\bullet\circ\\\bullet\circ\\\bullet\circ}} + \mathrm{T}_{\mathrm{O}}^{\substack{\bullet\circ\\\bullet\circ\\\circ\circ}} + \mathrm{T}_{\mathrm{O}}^{\substack{\bullet\circ\\\circ\circ\\\bullet\circ}} + \mathrm{T}_{\mathrm{O}}^{\substack{\bullet\circ\\\circ\circ\\\circ\circ}} + \mathrm{T}_{\mathrm{O}}^{\substack{\circ\circ\\\bullet\circ\\\bullet\circ}} + \mathrm{T}_{\mathrm{O}}^{\substack{\circ\circ\\\bullet\circ\\\circ\circ}} + \mathrm{T}_{\mathrm{O}}^{\substack{\circ\circ\\\circ\circ\\\bullet\circ}} + \mathrm{T}_{\mathrm{O}}^{\substack{\circ\circ\\\circ\circ\\\circ\circ}}\big) + \underset{\mathrm{CIo}}{\nu_f}\big(\mathrm{C}_{\mathrm{I}}^{\substack{\circ\\\circ\\\circ}}\big) - \underset{\mathrm{CKo}}{\nu_r}\Big(\big(\mathrm{D}_{\mathrm{O}}^{\substack{\bullet\\\bullet\\\bullet}} + \mathrm{D}_{\mathrm{O}}^{\substack{\bullet\\\bullet\\\circ}} + \mathrm{D}_{\mathrm{O}}^{\substack{\bullet\\\circ\\\bullet}} + \mathrm{D}_{\mathrm{O}}^{\substack{\bullet\\\circ\\\circ}} + \mathrm{D}_{\mathrm{O}}^{\substack{\circ\\\bullet\\\bullet}} + \mathrm{D}_{\mathrm{O}}^{\substack{\circ\\\bullet\\\circ}} + \mathrm{D}_{\mathrm{O}}^{\substack{\circ\\\circ\\\bullet}} + \mathrm{D}_{\mathrm{O}}^{\substack{\circ\\\circ\\\circ}}\big)\mathrm{C}_{\mathrm{O}}^{\substack{\circ\\\circ\\\circ}}\Big) - \underset{\mathrm{CIo}}{\nu_r}\big(\mathrm{C}_{\mathrm{O}}^{\substack{\circ\\\circ\\\circ}}\big)$$

$$\frac{d\mathrm{C}_{\mathrm{O}}^{\substack{\circ\\\circ\\\bullet}}}{dt} = \underset{\mathrm{CKo}}{\nu_f}\big(\mathrm{T}_{\mathrm{O}}^{\substack{\bullet\circ\\\bullet\circ\\\bullet\bullet}} + \mathrm{T}_{\mathrm{O}}^{\substack{\bullet\circ\\\bullet\circ\\\circ\bullet}} + \mathrm{T}_{\mathrm{O}}^{\substack{\bullet\circ\\\circ\circ\\\bullet\bullet}} + \mathrm{T}_{\mathrm{O}}^{\substack{\bullet\circ\\\circ\circ\\\circ\bullet}} + \mathrm{T}_{\mathrm{O}}^{\substack{\circ\circ\\\bullet\circ\\\bullet\bullet}} + \mathrm{T}_{\mathrm{O}}^{\substack{\circ\circ\\\bullet\circ\\\circ\bullet}} + \mathrm{T}_{\mathrm{O}}^{\substack{\circ\circ\\\circ\circ\\\bullet\bullet}} + \mathrm{T}_{\mathrm{O}}^{\substack{\circ\circ\\\circ\circ\\\circ\bullet}}\big) + \underset{\mathrm{CIo}}{\nu_f}\big(\mathrm{C}_{\mathrm{I}}^{\substack{\circ\\\circ\\\bullet}}\big) - \underset{\mathrm{CKo}}{\nu_r}\Big(\big(\mathrm{D}_{\mathrm{O}}^{\substack{\bullet\\\bullet\\\bullet}} + \mathrm{D}_{\mathrm{O}}^{\substack{\bullet\\\bullet\\\circ}} + \mathrm{D}_{\mathrm{O}}^{\substack{\bullet\\\circ\\\bullet}} + \mathrm{D}_{\mathrm{O}}^{\substack{\bullet\\\circ\\\circ}} + \mathrm{D}_{\mathrm{O}}^{\substack{\circ\\\bullet\\\bullet}} + \mathrm{D}_{\mathrm{O}}^{\substack{\circ\\\bullet\\\circ}} + \mathrm{D}_{\mathrm{O}}^{\substack{\circ\\\circ\\\bullet}} + \mathrm{D}_{\mathrm{O}}^{\substack{\circ\\\circ\\\circ}}\big)\mathrm{C}_{\mathrm{O}}^{\substack{\circ\\\circ\\\bullet}}\Big) - \underset{\mathrm{CIo}}{\nu_r}\big(\mathrm{C}_{\mathrm{O}}^{\substack{\circ\\\circ\\\bullet}}\big)$$

$$\frac{d\mathrm{C}_{\mathrm{O}}^{\substack{\circ\\\bullet\\\circ}}}{dt} = \underset{\mathrm{CKo}}{\nu_f}\big(\mathrm{T}_{\mathrm{O}}^{\substack{\bullet\circ\\\bullet\bullet\\\bullet\circ}} + \mathrm{T}_{\mathrm{O}}^{\substack{\bullet\circ\\\bullet\bullet\\\circ\circ}} + \mathrm{T}_{\mathrm{O}}^{\substack{\bullet\circ\\\circ\bullet\\\bullet\circ}} + \mathrm{T}_{\mathrm{O}}^{\substack{\bullet\circ\\\circ\bullet\\\circ\circ}} + \mathrm{T}_{\mathrm{O}}^{\substack{\circ\circ\\\bullet\bullet\\\bullet\circ}} + \mathrm{T}_{\mathrm{O}}^{\substack{\circ\circ\\\bullet\bullet\\\circ\circ}} + \mathrm{T}_{\mathrm{O}}^{\substack{\circ\circ\\\circ\bullet\\\bullet\circ}} + \mathrm{T}_{\mathrm{O}}^{\substack{\circ\circ\\\circ\bullet\\\circ\circ}}\big) + \underset{\mathrm{CIo}}{\nu_f}\big(\mathrm{C}_{\mathrm{I}}^{\substack{\circ\\\bullet\\\circ}}\big) - \underset{\mathrm{CKo}}{\nu_r}\Big(\big(\mathrm{D}_{\mathrm{O}}^{\substack{\bullet\\\bullet\\\bullet}} + \mathrm{D}_{\mathrm{O}}^{\substack{\bullet\\\bullet\\\circ}} + \mathrm{D}_{\mathrm{O}}^{\substack{\bullet\\\circ\\\bullet}} + \mathrm{D}_{\mathrm{O}}^{\substack{\bullet\\\circ\\\circ}} + \mathrm{D}_{\mathrm{O}}^{\substack{\circ\\\bullet\\\bullet}} + \mathrm{D}_{\mathrm{O}}^{\substack{\circ\\\bullet\\\circ}} + \mathrm{D}_{\mathrm{O}}^{\substack{\circ\\\circ\\\bullet}} + \mathrm{D}_{\mathrm{O}}^{\substack{\circ\\\circ\\\circ}}\big)\mathrm{C}_{\mathrm{O}}^{\substack{\circ\\\bullet\\\circ}}\Big) - \underset{\mathrm{CIo}}{\nu_r}\big(\mathrm{C}_{\mathrm{O}}^{\substack{\circ\\\bullet\\\circ}}\big)$$

$$\frac{d\mathrm{C}_{\mathrm{O}}^{\substack{\circ\\\bullet\\\bullet}}}{dt} = \underset{\mathrm{CKo}}{\nu_f}\big(\mathrm{T}_{\mathrm{O}}^{\substack{\bullet\circ\\\bullet\bullet\\\bullet\bullet}} + \mathrm{T}_{\mathrm{O}}^{\substack{\bullet\circ\\\bullet\bullet\\\circ\bullet}} + \mathrm{T}_{\mathrm{O}}^{\substack{\bullet\circ\\\circ\bullet\\\bullet\bullet}} + \mathrm{T}_{\mathrm{O}}^{\substack{\bullet\circ\\\circ\bullet\\\circ\bullet}} + \mathrm{T}_{\mathrm{O}}^{\substack{\circ\circ\\\bullet\bullet\\\bullet\bullet}} + \mathrm{T}_{\mathrm{O}}^{\substack{\circ\circ\\\bullet\bullet\\\circ\bullet}} + \mathrm{T}_{\mathrm{O}}^{\substack{\circ\circ\\\circ\bullet\\\bullet\bullet}} + \mathrm{T}_{\mathrm{O}}^{\substack{\circ\circ\\\circ\bullet\\\circ\bullet}}\big) + \underset{\mathrm{CIo}}{\nu_f}\big(\mathrm{C}_{\mathrm{I}}^{\substack{\circ\\\bullet\\\bullet}}\big) - \underset{\mathrm{CKo}}{\nu_r}\Big(\big(\mathrm{D}_{\mathrm{O}}^{\substack{\bullet\\\bullet\\\bullet}} + \mathrm{D}_{\mathrm{O}}^{\substack{\bullet\\\bullet\\\circ}} + \mathrm{D}_{\mathrm{O}}^{\substack{\bullet\\\circ\\\bullet}} + \mathrm{D}_{\mathrm{O}}^{\substack{\bullet\\\circ\\\circ}} + \mathrm{D}_{\mathrm{O}}^{\substack{\circ\\\bullet\\\bullet}} + \mathrm{D}_{\mathrm{O}}^{\substack{\circ\\\bullet\\\circ}} +$$

$$\mathrm{D_O}^{\circ\circ\bullet} + \mathrm{D_O}^{\circ\circ\circ})\mathrm{C_O}^{\circ\bullet\bullet}) - \nu^{r}_{\mathrm{CIO}}(\mathrm{C_O}^{\circ\bullet\bullet})$$

$$\frac{d\mathrm{C_O}^{\bullet\circ\circ}}{dt} = \nu^{f}_{\mathrm{CKO}}(\mathrm{T_O}^{\bullet\bullet,\bullet\circ,\bullet\circ} + \mathrm{T_O}^{\bullet\bullet,\bullet\circ,\circ\circ} + \mathrm{T_O}^{\bullet\bullet,\circ\circ,\bullet\circ} + \mathrm{T_O}^{\bullet\bullet,\circ\circ,\circ\circ} + \mathrm{T_O}^{\circ\bullet,\bullet\circ,\bullet\circ} + \mathrm{T_O}^{\circ\bullet,\bullet\circ,\circ\circ} + \mathrm{T_O}^{\circ\bullet,\circ\circ,\bullet\circ} + \mathrm{T_O}^{\circ\bullet,\circ\circ,\circ\circ}) + \nu^{f}_{\mathrm{CIO}}(\mathrm{C_I}^{\bullet\circ\circ}) - \nu^{r}_{\mathrm{CKO}}((\mathrm{D_O}^{\bullet\bullet\bullet} + \mathrm{D_O}^{\bullet\bullet\circ} + \mathrm{D_O}^{\bullet\circ\bullet} + \mathrm{D_O}^{\bullet\circ\circ} + \mathrm{D_O}^{\circ\bullet\bullet} + \mathrm{D_O}^{\circ\bullet\circ} + \mathrm{D_O}^{\circ\circ\bullet} + \mathrm{D_O}^{\circ\circ\circ})\mathrm{C_O}^{\bullet\circ\circ}) - \nu^{r}_{\mathrm{CIO}}(\mathrm{C_O}^{\bullet\circ\circ})$$

$$\frac{d\mathrm{C_O}^{\bullet\circ\bullet}}{dt} = \nu^{f}_{\mathrm{CKO}}(\mathrm{T_O}^{\bullet\bullet,\bullet\circ,\bullet\bullet} + \mathrm{T_O}^{\bullet\bullet,\bullet\circ,\circ\bullet} + \mathrm{T_O}^{\bullet\bullet,\circ\circ,\bullet\bullet} + \mathrm{T_O}^{\bullet\bullet,\circ\circ,\circ\bullet} + \mathrm{T_O}^{\circ\bullet,\bullet\circ,\bullet\bullet} + \mathrm{T_O}^{\circ\bullet,\bullet\circ,\circ\bullet} + \mathrm{T_O}^{\circ\bullet,\circ\circ,\bullet\bullet} + \mathrm{T_O}^{\circ\bullet,\circ\circ,\circ\bullet}) + \nu^{f}_{\mathrm{CIO}}(\mathrm{C_I}^{\bullet\circ\bullet}) - \nu^{r}_{\mathrm{CKO}}((\mathrm{D_O}^{\bullet\bullet\bullet} + \mathrm{D_O}^{\bullet\bullet\circ} + \mathrm{D_O}^{\bullet\circ\bullet} + \mathrm{D_O}^{\bullet\circ\circ} + \mathrm{D_O}^{\circ\bullet\bullet} + \mathrm{D_O}^{\circ\bullet\circ} + \mathrm{D_O}^{\circ\circ\bullet} + \mathrm{D_O}^{\circ\circ\circ})\mathrm{C_O}^{\bullet\circ\bullet}) - \nu^{r}_{\mathrm{CIO}}(\mathrm{C_O}^{\bullet\circ\bullet})$$

$$\frac{d\mathrm{C_O}^{\bullet\bullet\circ}}{dt} = \nu^{f}_{\mathrm{CKO}}(\mathrm{T_O}^{\bullet\bullet,\bullet\bullet,\bullet\circ} + \mathrm{T_O}^{\bullet\bullet,\bullet\bullet,\circ\circ} + \mathrm{T_O}^{\bullet\bullet,\circ\bullet,\bullet\circ} + \mathrm{T_O}^{\bullet\bullet,\circ\bullet,\circ\circ} + \mathrm{T_O}^{\circ\bullet,\bullet\bullet,\bullet\circ} + \mathrm{T_O}^{\circ\bullet,\bullet\bullet,\circ\circ} + \mathrm{T_O}^{\circ\bullet,\circ\bullet,\bullet\circ} + \mathrm{T_O}^{\circ\bullet,\circ\bullet,\circ\circ}) + \nu^{f}_{\mathrm{CIO}}(\mathrm{C_I}^{\bullet\bullet\circ}) - \nu^{r}_{\mathrm{CKO}}((\mathrm{D_O}^{\bullet\bullet\bullet} + \mathrm{D_O}^{\bullet\bullet\circ} + \mathrm{D_O}^{\bullet\circ\bullet} + \mathrm{D_O}^{\bullet\circ\circ} + \mathrm{D_O}^{\circ\bullet\bullet} + \mathrm{D_O}^{\circ\bullet\circ} + \mathrm{D_O}^{\circ\circ\bullet} + \mathrm{D_O}^{\circ\circ\circ})\mathrm{C_O}^{\bullet\bullet\circ}) - \nu^{r}_{\mathrm{CIO}}(\mathrm{C_O}^{\bullet\bullet\circ})$$

$$\frac{d\mathrm{C_O}^{\bullet\bullet\bullet}}{dt} = \nu^{f}_{\mathrm{CKO}}(\mathrm{T_O}^{\bullet\bullet,\bullet\bullet,\bullet\bullet} + \mathrm{T_O}^{\bullet\bullet,\bullet\bullet,\circ\bullet} + \mathrm{T_O}^{\bullet\bullet,\circ\bullet,\bullet\bullet} + \mathrm{T_O}^{\bullet\bullet,\circ\bullet,\circ\bullet} + \mathrm{T_O}^{\circ\bullet,\bullet\bullet,\bullet\bullet} + \mathrm{T_O}^{\circ\bullet,\bullet\bullet,\circ\bullet} + \mathrm{T_O}^{\circ\bullet,\circ\bullet,\bullet\bullet} + \mathrm{T_O}^{\circ\bullet,\circ\bullet,\circ\bullet}) + \nu^{f}_{\mathrm{CIO}}(\mathrm{C_I}^{\bullet\bullet\bullet}) - \nu^{r}_{\mathrm{CKO}}((\mathrm{D_O}^{\bullet\bullet\bullet} + \mathrm{D_O}^{\bullet\bullet\circ} + \mathrm{D_O}^{\bullet\circ\bullet} + \mathrm{D_O}^{\bullet\circ\circ} + \mathrm{D_O}^{\circ\bullet\bullet} + \mathrm{D_O}^{\circ\bullet\circ} + \mathrm{D_O}^{\circ\circ\bullet} + \mathrm{D_O}^{\circ\circ\circ})\mathrm{C_O}^{\bullet\bullet\bullet}) - \nu^{r}_{\mathrm{CIO}}(\mathrm{C_O}^{\bullet\bullet\bullet})$$

$$\frac{d\mathrm{P_E}^{\circ\circ\circ\circ}}{dt} = \nu^{f}_{\mathrm{ASE}}((\mathrm{T_E}^{\bullet\circ,\bullet\circ,\bullet\circ} + \mathrm{T_E}^{\bullet\circ,\bullet\circ,\circ\circ} + \mathrm{T_E}^{\bullet\circ,\circ\circ,\bullet\circ} + \mathrm{T_E}^{\bullet\circ,\circ\circ,\circ\circ} + \mathrm{T_E}^{\circ\circ,\bullet\circ,\bullet\circ} + \mathrm{T_E}^{\circ\circ,\bullet\circ,\circ\circ} + \mathrm{T_E}^{\circ\circ,\circ\circ,\bullet\circ} + \mathrm{T_E}^{\circ\circ,\circ\circ,\circ\circ})\mathrm{W_E}^{\circ}) + \nu^{r}_{\mathrm{PEO}}(\mathrm{P_O}^{\circ\circ\circ\circ}) - \nu^{f}_{\mathrm{PEO}}(\mathrm{P_E}^{\circ\circ\circ\circ}) - \nu^{r}_{\mathrm{ASE}}((\mathrm{D_E}^{\bullet\bullet\bullet} + \mathrm{D_E}^{\bullet\bullet\circ} + \mathrm{D_E}^{\bullet\circ\bullet} + \mathrm{D_E}^{\bullet\circ\circ} + \mathrm{D_E}^{\circ\bullet\bullet} + \mathrm{D_E}^{\circ\bullet\circ} + \mathrm{D_E}^{\circ\circ\bullet} + \mathrm{D_E}^{\circ\circ\circ})\mathrm{P_E}^{\circ\circ\circ\circ})$$

$$\frac{d\mathrm{P_E}^{\circ\circ\circ\bullet}}{dt} = \nu^{f}_{\mathrm{ASE}}(\tfrac{1}{4}((\mathrm{T_E}^{\bullet\bullet,\bullet\circ,\bullet\circ} + \mathrm{T_E}^{\bullet\circ,\bullet\bullet,\bullet\circ} + \mathrm{T_E}^{\bullet\circ,\bullet\circ,\bullet\bullet} + \mathrm{T_E}^{\bullet\bullet,\bullet\circ,\circ\circ} + \mathrm{T_E}^{\bullet\circ,\bullet\bullet,\circ\circ} + \mathrm{T_E}^{\bullet\circ,\bullet\circ,\circ\bullet} + \mathrm{T_E}^{\bullet\bullet,\circ\circ,\bullet\circ} + \mathrm{T_E}^{\bullet\circ,\circ\bullet,\bullet\circ} + \mathrm{T_E}^{\bullet\circ,\circ\circ,\bullet\bullet} + \mathrm{T_E}^{\bullet\bullet,\circ\circ,\circ\circ} + \mathrm{T_E}^{\bullet\circ,\circ\bullet,\circ\circ} + \mathrm{T_E}^{\bullet\circ,\circ\circ,\circ\bullet} + \mathrm{T_E}^{\circ\bullet,\bullet\circ,\bullet\circ} + \mathrm{T_E}^{\circ\circ,\bullet\bullet,\bullet\circ} + \mathrm{T_E}^{\circ\circ,\bullet\circ,\bullet\bullet} + \mathrm{T_E}^{\circ\bullet,\bullet\circ,\circ\circ} + \mathrm{T_E}^{\circ\circ,\bullet\bullet,\circ\circ} + \mathrm{T_E}^{\circ\circ,\bullet\circ,\circ\bullet} + \mathrm{T_E}^{\circ\bullet,\circ\circ,\bullet\circ} + \mathrm{T_E}^{\circ\circ,\circ\bullet,\bullet\circ} + \mathrm{T_E}^{\circ\circ,\circ\circ,\bullet\bullet} + \mathrm{T_E}^{\circ\bullet,\circ\circ,\circ\circ} + \mathrm{T_E}^{\circ\circ,\circ\bullet,\circ\circ} + \mathrm{T_E}^{\circ\circ,\circ\circ,\circ\bullet})\mathrm{W_E}^{\circ} + (\mathrm{T_E}^{\bullet\circ,\bullet\circ,\bullet\circ} + \mathrm{T_E}^{\bullet\circ,\bullet\circ,\circ\circ} + \mathrm{T_E}^{\bullet\circ,\circ\circ,\bullet\circ} + \mathrm{T_E}^{\bullet\circ,\circ\circ,\circ\circ} + \mathrm{T_E}^{\circ\circ,\bullet\circ,\bullet\circ} + \mathrm{T_E}^{\circ\circ,\bullet\circ,\circ\circ} + \mathrm{T_E}^{\circ\circ,\circ\circ,\bullet\circ} + \mathrm{T_E}^{\circ\circ,\circ\circ,\circ\circ})\mathrm{W_E}^{\bullet})) + \nu^{r}_{\mathrm{PEO}}(\mathrm{P_O}^{\circ\circ\circ\bullet}) - \nu^{f}_{\mathrm{PEO}}(\mathrm{P_E}^{\circ\circ\circ\bullet}) - \nu^{r}_{\mathrm{ASE}}((\mathrm{D_E}^{\bullet\bullet\bullet} + \mathrm{D_E}^{\bullet\bullet\circ} + \mathrm{D_E}^{\bullet\circ\bullet} + \mathrm{D_E}^{\bullet\circ\circ} + \mathrm{D_E}^{\circ\bullet\bullet} + \mathrm{D_E}^{\circ\bullet\circ} + \mathrm{D_E}^{\circ\circ\bullet} + \mathrm{D_E}^{\circ\circ\circ})\mathrm{P_E}^{\circ\circ\circ\bullet})$$

$$\frac{d\mathrm{P_E}^{\circ\circ\bullet\circ}}{dt} = \nu^{f}_{\mathrm{ASE}}(\tfrac{1}{4}((\mathrm{T_E}^{\bullet\bullet,\bullet\circ,\bullet\circ} + \mathrm{T_E}^{\bullet\circ,\bullet\bullet,\bullet\circ} + \mathrm{T_E}^{\bullet\circ,\bullet\circ,\bullet\bullet} + \mathrm{T_E}^{\bullet\bullet,\bullet\circ,\circ\circ} + \mathrm{T_E}^{\bullet\circ,\bullet\bullet,\circ\circ} + \mathrm{T_E}^{\bullet\circ,\bullet\circ,\circ\bullet} + \mathrm{T_E}^{\bullet\bullet,\circ\circ,\bullet\circ} + \mathrm{T_E}^{\bullet\circ,\circ\bullet,\bullet\circ} + \mathrm{T_E}^{\bullet\circ,\circ\circ,\bullet\bullet} + \mathrm{T_E}^{\bullet\bullet,\circ\circ,\circ\circ} + \mathrm{T_E}^{\bullet\circ,\circ\bullet,\circ\circ} + \mathrm{T_E}^{\bullet\circ,\circ\circ,\circ\bullet} + \mathrm{T_E}^{\circ\bullet,\bullet\circ,\bullet\circ} + \mathrm{T_E}^{\circ\circ,\bullet\bullet,\bullet\circ} + \mathrm{T_E}^{\circ\circ,\bullet\circ,\bullet\bullet} + \mathrm{T_E}^{\circ\bullet,\bullet\circ,\circ\circ} + \mathrm{T_E}^{\circ\circ,\bullet\bullet,\circ\circ} + \mathrm{T_E}^{\circ\circ,\bullet\circ,\circ\bullet} + \mathrm{T_E}^{\circ\bullet,\circ\circ,\bullet\circ} + \mathrm{T_E}^{\circ\circ,\circ\bullet,\bullet\circ} + \mathrm{T_E}^{\circ\circ,\circ\circ,\bullet\bullet} + \mathrm{T_E}^{\circ\bullet,\circ\circ,\circ\circ} + \mathrm{T_E}^{\circ\circ,\circ\bullet,\circ\circ} + \mathrm{T_E}^{\circ\circ,\circ\circ,\circ\bullet})\mathrm{W_E}^{\circ} + (\mathrm{T_E}^{\bullet\circ,\bullet\circ,\bullet\circ} + \mathrm{T_E}^{\bullet\circ,\bullet\circ,\circ\circ} + \mathrm{T_E}^{\bullet\circ,\circ\circ,\bullet\circ} + \mathrm{T_E}^{\bullet\circ,\circ\circ,\circ\circ} + \mathrm{T_E}^{\circ\circ,\bullet\circ,\bullet\circ} + \mathrm{T_E}^{\circ\circ,\bullet\circ,\circ\circ} + \mathrm{T_E}^{\circ\circ,\circ\circ,\bullet\circ} + \mathrm{T_E}^{\circ\circ,\circ\circ,\circ\circ})\mathrm{W_E}^{\bullet})) + \nu^{r}_{\mathrm{PEO}}(\mathrm{P_O}^{\circ\circ\bullet\circ}) - \nu^{f}_{\mathrm{PEO}}(\mathrm{P_E}^{\circ\circ\bullet\circ}) - \nu^{r}_{\mathrm{ASE}}((\mathrm{D_E}^{\bullet\bullet\bullet} + \mathrm{D_E}^{\bullet\bullet\circ} + \mathrm{D_E}^{\bullet\circ\bullet} + \mathrm{D_E}^{\bullet\circ\circ} + \mathrm{D_E}^{\circ\bullet\bullet} + \mathrm{D_E}^{\circ\bullet\circ} + \mathrm{D_E}^{\circ\circ\bullet} + \mathrm{D_E}^{\circ\circ\circ})\mathrm{P_E}^{\circ\circ\bullet\circ})$$

$$\frac{d\mathrm{P_E}^{\circ\circ\bullet\bullet}}{dt} = \nu^{f}_{\mathrm{ASE}}(\tfrac{1}{6}((\mathrm{T_E}^{\bullet\bullet,\bullet\bullet,\bullet\circ} + \mathrm{T_E}^{\bullet\bullet,\bullet\circ,\bullet\bullet} + \mathrm{T_E}^{\bullet\circ,\bullet\bullet,\bullet\bullet} + \mathrm{T_E}^{\bullet\bullet,\bullet\bullet,\circ\circ} + \mathrm{T_E}^{\bullet\bullet,\bullet\circ,\circ\bullet} + \mathrm{T_E}^{\bullet\circ,\bullet\bullet,\circ\bullet} + \mathrm{T_E}^{\bullet\bullet,\circ\bullet,\bullet\circ} + \mathrm{T_E}^{\bullet\bullet,\circ\circ,\bullet\bullet} + \mathrm{T_E}^{\bullet\circ,\circ\bullet,\bullet\bullet} + \mathrm{T_E}^{\bullet\bullet,\circ\bullet,\circ\circ} + \mathrm{T_E}^{\bullet\bullet,\circ\circ,\circ\bullet} + \mathrm{T_E}^{\bullet\circ,\circ\bullet,\circ\bullet} + \mathrm{T_E}^{\circ\bullet,\bullet\bullet,\bullet\circ} + \mathrm{T_E}^{\circ\bullet,\bullet\circ,\bullet\bullet} + \mathrm{T_E}^{\circ\circ,\bullet\bullet,\bullet\bullet} + \mathrm{T_E}^{\circ\bullet,\bullet\bullet,\circ\circ} + \mathrm{T_E}^{\circ\bullet,\bullet\circ,\circ\bullet} + \mathrm{T_E}^{\circ\circ,\bullet\bullet,\circ\bullet} + \mathrm{T_E}^{\circ\bullet,\circ\bullet,\bullet\circ} + \mathrm{T_E}^{\circ\bullet,\circ\circ,\bullet\bullet} + \mathrm{T_E}^{\circ\circ,\circ\bullet,\bullet\bullet} + \mathrm{T_E}^{\circ\bullet,\circ\bullet,\circ\circ} + \mathrm{T_E}^{\circ\bullet,\circ\circ,\circ\bullet} + \mathrm{T_E}^{\circ\circ,\circ\bullet,\circ\bullet})\mathrm{W_E}^{\circ} + (\mathrm{T_E}^{\bullet\bullet,\bullet\circ,\bullet\circ} + \mathrm{T_E}^{\bullet\circ,\bullet\bullet,\bullet\circ} + \mathrm{T_E}^{\bullet\circ,\bullet\circ,\bullet\bullet} + \mathrm{T_E}^{\bullet\bullet,\bullet\circ,\circ\circ} + \mathrm{T_E}^{\bullet\circ,\bullet\bullet,\circ\circ} + \mathrm{T_E}^{\bullet\circ,\bullet\circ,\circ\bullet} + \mathrm{T_E}^{\bullet\bullet,\circ\circ,\bullet\circ} + \mathrm{T_E}^{\bullet\circ,\circ\bullet,\bullet\circ} + \mathrm{T_E}^{\bullet\circ,\circ\circ,\bullet\bullet} + \mathrm{T_E}^{\bullet\bullet,\circ\circ,\circ\circ} + \mathrm{T_E}^{\bullet\circ,\circ\bullet,\circ\circ} + \mathrm{T_E}^{\bullet\circ,\circ\circ,\circ\bullet} + \mathrm{T_E}^{\circ\bullet,\bullet\circ,\bullet\circ} + \mathrm{T_E}^{\circ\circ,\bullet\bullet,\bullet\circ} + \mathrm{T_E}^{\circ\circ,\bullet\circ,\bullet\bullet} + \mathrm{T_E}^{\circ\bullet,\bullet\circ,\circ\circ} + \mathrm{T_E}^{\circ\circ,\bullet\bullet,\circ\circ} + \mathrm{T_E}^{\circ\circ,\bullet\circ,\circ\bullet} + \mathrm{T_E}^{\circ\bullet,\circ\circ,\bullet\circ} + \mathrm{T_E}^{\circ\circ,\circ\bullet,\bullet\circ} + \mathrm{T_E}^{\circ\circ,\circ\circ,\bullet\bullet} + \mathrm{T_E}^{\circ\bullet,\circ\circ,\circ\circ} + \mathrm{T_E}^{\circ\circ,\circ\bullet,\circ\circ} + \mathrm{T_E}^{\circ\circ,\circ\circ,\circ\bullet})\mathrm{W_E}^{\bullet})) + \nu^{r}_{\mathrm{PEO}}(\mathrm{P_O}^{\circ\circ\bullet\bullet}) - \nu^{f}_{\mathrm{PEO}}(\mathrm{P_E}^{\circ\circ\bullet\bullet}) - \nu^{r}_{\mathrm{ASE}}((\mathrm{D_E}^{\bullet\bullet\bullet} + \mathrm{D_E}^{\bullet\bullet\circ} + \mathrm{D_E}^{\bullet\circ\bullet} + \mathrm{D_E}^{\bullet\circ\circ} + \mathrm{D_E}^{\circ\bullet\bullet} + \mathrm{D_E}^{\circ\bullet\circ} + \mathrm{D_E}^{\circ\circ\bullet} + \mathrm{D_E}^{\circ\circ\circ})\mathrm{P_E}^{\circ\circ\bullet\bullet})$$

$$\frac{d\mathrm{P_E}^{\circ\bullet\circ\circ}}{dt} = \nu^{f}_{\mathrm{ASE}}(\tfrac{1}{4}((\mathrm{T_E}^{\bullet\bullet,\bullet\circ,\bullet\circ} + \mathrm{T_E}^{\bullet\circ,\bullet\bullet,\bullet\circ} + \mathrm{T_E}^{\bullet\circ,\bullet\circ,\bullet\bullet} + \mathrm{T_E}^{\bullet\bullet,\bullet\circ,\circ\circ} + \mathrm{T_E}^{\bullet\circ,\bullet\bullet,\circ\circ} + \mathrm{T_E}^{\bullet\circ,\bullet\circ,\circ\bullet} + \mathrm{T_E}^{\bullet\bullet,\circ\circ,\bullet\circ} + \mathrm{T_E}^{\bullet\circ,\circ\bullet,\bullet\circ} + \mathrm{T_E}^{\bullet\circ,\circ\circ,\bullet\bullet} + \mathrm{T_E}^{\bullet\bullet,\circ\circ,\circ\circ} + \mathrm{T_E}^{\bullet\circ,\circ\bullet,\circ\circ} + \mathrm{T_E}^{\bullet\circ,\circ\circ,\circ\bullet} + \mathrm{T_E}^{\circ\bullet,\bullet\circ,\bullet\circ} + \mathrm{T_E}^{\circ\circ,\bullet\bullet,\bullet\circ} + \mathrm{T_E}^{\circ\circ,\bullet\circ,\bullet\bullet} + \mathrm{T_E}^{\circ\bullet,\bullet\circ,\circ\circ} + \mathrm{T_E}^{\circ\circ,\bullet\bullet,\circ\circ} + \mathrm{T_E}^{\circ\circ,\bullet\circ,\circ\bullet} + \mathrm{T_E}^{\circ\bullet,\circ\circ,\bullet\circ} + \mathrm{T_E}^{\circ\circ,\circ\bullet,\bullet\circ} + \mathrm{T_E}^{\circ\circ,\circ\circ,\bullet\bullet} + \mathrm{T_E}^{\circ\bullet,\circ\circ,\circ\circ} + \mathrm{T_E}^{\circ\circ,\circ\bullet,\circ\circ} + \mathrm{T_E}^{\circ\circ,\circ\circ,\circ\bullet})\mathrm{W_E}^{\circ} + (\mathrm{T_E}^{\bullet\circ,\bullet\circ,\bullet\circ} + \mathrm{T_E}^{\bullet\circ,\bullet\circ,\circ\circ} + \mathrm{T_E}^{\bullet\circ,\circ\circ,\bullet\circ} + \mathrm{T_E}^{\bullet\circ,\circ\circ,\circ\circ} + \mathrm{T_E}^{\circ\circ,\bullet\circ,\bullet\circ} + \mathrm{T_E}^{\circ\circ,\bullet\circ,\circ\circ} + \mathrm{T_E}^{\circ\circ,\circ\circ,\bullet\circ} + \mathrm{T_E}^{\circ\circ,\circ\circ,\circ\circ})\mathrm{W_E}^{\bullet})) + \nu^{r}_{\mathrm{PEO}}(\mathrm{P_O}^{\circ\bullet\circ\circ}) - \nu^{f}_{\mathrm{PEO}}(\mathrm{P_E}^{\circ\bullet\circ\circ}) - \nu^{r}_{\mathrm{ASE}}((\mathrm{D_E}^{\bullet\bullet\bullet} + \mathrm{D_E}^{\bullet\bullet\circ} + \mathrm{D_E}^{\bullet\circ\bullet} + \mathrm{D_E}^{\bullet\circ\circ} + \mathrm{D_E}^{\circ\bullet\bullet} + \mathrm{D_E}^{\circ\bullet\circ} + \mathrm{D_E}^{\circ\circ\bullet} + \mathrm{D_E}^{\circ\circ\circ})\mathrm{P_E}^{\circ\bullet\circ\circ})$$

$$\frac{d\mathrm{P_E}^{\circ\bullet\circ\bullet}}{dt} = \nu^{f}_{\mathrm{ASE}}(\tfrac{1}{6}((\mathrm{T_E}^{\bullet\bullet,\bullet\bullet,\bullet\circ} + \mathrm{T_E}^{\bullet\bullet,\bullet\circ,\bullet\bullet} + \mathrm{T_E}^{\bullet\circ,\bullet\bullet,\bullet\bullet} + \mathrm{T_E}^{\bullet\bullet,\bullet\bullet,\circ\circ} + \mathrm{T_E}^{\bullet\bullet,\bullet\circ,\circ\bullet} + \mathrm{T_E}^{\bullet\circ,\bullet\bullet,\circ\bullet} + \mathrm{T_E}^{\bullet\bullet,\circ\bullet,\bullet\circ} + \mathrm{T_E}^{\bullet\bullet,\circ\circ,\bullet\bullet} + \mathrm{T_E}^{\bullet\circ,\circ\bullet,\bullet\bullet} + \mathrm{T_E}^{\bullet\bullet,\circ\bullet,\circ\circ} + \mathrm{T_E}^{\bullet\bullet,\circ\circ,\circ\bullet} + \mathrm{T_E}^{\bullet\circ,\circ\bullet,\circ\bullet} + \mathrm{T_E}^{\circ\bullet,\bullet\bullet,\bullet\circ} + \mathrm{T_E}^{\circ\bullet,\bullet\circ,\bullet\bullet} + \mathrm{T_E}^{\circ\circ,\bullet\bullet,\bullet\bullet} + \mathrm{T_E}^{\circ\bullet,\bullet\bullet,\circ\circ} + \mathrm{T_E}^{\circ\bullet,\bullet\circ,\circ\bullet} + \mathrm{T_E}^{\circ\circ,\bullet\bullet,\circ\bullet} + \mathrm{T_E}^{\circ\bullet,\circ\bullet,\bullet\circ} + \mathrm{T_E}^{\circ\bullet,\circ\circ,\bullet\bullet} + \mathrm{T_E}^{\circ\circ,\circ\bullet,\bullet\bullet} + \mathrm{T_E}^{\circ\bullet,\circ\bullet,\circ\circ} + \mathrm{T_E}^{\circ\bullet,\circ\circ,\circ\bullet} + \mathrm{T_E}^{\circ\circ,\circ\bullet,\circ\bullet})\mathrm{W_E}^{\circ} + (\mathrm{T_E}^{\bullet\bullet,\bullet\circ,\bullet\circ} + \mathrm{T_E}^{\bullet\circ,\bullet\bullet,\bullet\circ} + \mathrm{T_E}^{\bullet\circ,\bullet\circ,\bullet\bullet} + \mathrm{T_E}^{\bullet\bullet,\bullet\circ,\circ\circ} + \mathrm{T_E}^{\bullet\circ,\bullet\bullet,\circ\circ} + \mathrm{T_E}^{\bullet\circ,\bullet\circ,\circ\bullet} + \mathrm{T_E}^{\bullet\bullet,\circ\circ,\bullet\circ} + \mathrm{T_E}^{\bullet\circ,\circ\bullet,\bullet\circ} + \mathrm{T_E}^{\bullet\circ,\circ\circ,\bullet\bullet} + \mathrm{T_E}^{\bullet\bullet,\circ\circ,\circ\circ} + \mathrm{T_E}^{\bullet\circ,\circ\bullet,\circ\circ} + \mathrm{T_E}^{\bullet\circ,\circ\circ,\circ\bullet} + \mathrm{T_E}^{\circ\bullet,\bullet\circ,\bullet\circ} + \mathrm{T_E}^{\circ\circ,\bullet\bullet,\bullet\circ} + \mathrm{T_E}^{\circ\circ,\bullet\circ,\bullet\bullet} + \mathrm{T_E}^{\circ\bullet,\bullet\circ,\circ\circ} + \mathrm{T_E}^{\circ\circ,\bullet\bullet,\circ\circ} + \mathrm{T_E}^{\circ\circ,\bullet\circ,\circ\bullet} + \mathrm{T_E}^{\circ\bullet,\circ\circ,\bullet\circ} + \mathrm{T_E}^{\circ\circ,\circ\bullet,\bullet\circ} + \mathrm{T_E}^{\circ\circ,\circ\circ,\bullet\bullet} + \mathrm{T_E}^{\circ\bullet,\circ\circ,\circ\circ} + \mathrm{T_E}^{\circ\circ,\circ\bullet,\circ\circ} + \mathrm{T_E}^{\circ\circ,\circ\circ,\circ\bullet})\mathrm{W_E}^{\bullet})) + \nu^{r}_{\mathrm{PEO}}(\mathrm{P_O}^{\circ\bullet\circ\bullet}) - \nu^{f}_{\mathrm{PEO}}(\mathrm{P_E}^{\circ\bullet\circ\bullet}) - \nu^{r}_{\mathrm{ASE}}((\mathrm{D_E}^{\bullet\bullet\bullet} + \mathrm{D_E}^{\bullet\bullet\circ} + \mathrm{D_E}^{\bullet\circ\bullet} + \mathrm{D_E}^{\bullet\circ\circ} + \mathrm{D_E}^{\circ\bullet\bullet} + \mathrm{D_E}^{\circ\bullet\circ} + \mathrm{D_E}^{\circ\circ\bullet} + \mathrm{D_E}^{\circ\circ\circ})\mathrm{P_E}^{\circ\bullet\circ\bullet})$$

$$\frac{d\mathrm{P_E}^{[illegible]}}{dt} = \nu^{f}_{\mathrm{ASE}}\Big(\tfrac{1}{6}\Big(\big(\mathrm{T_E}^{[illegible]} + \mathrm{T_E}^{[illegible]} + \mathrm{T_E}^{[illegible]} + \mathrm{T_E}^{[illegible]} + \mathrm{T_E}^{[illegible]} + \mathrm{T_E}^{[illegible]} + \mathrm{T_E}^{[illegible]} + \mathrm{T_E}^{[illegible]} + \mathrm{T_E}^{[illegible]} + \mathrm{T_E}^{[illegible]} + \mathrm{T_E}^{[illegible]} + \mathrm{T_E}^{[illegible]} + \mathrm{T_E}^{[illegible]} + \mathrm{T_E}^{[illegible]} + \mathrm{T_E}^{[illegible]} + \cdots\big)\mathrm{W_E}^{\circ} + \big(\cdots\big)\mathrm{W_E}^{\bullet}\Big)\Big) + \nu^{r}_{\mathrm{PEO}}\big(\mathrm{P_O}^{[illegible]}\big) - \nu^{f}_{\mathrm{PEO}}\big(\mathrm{P_E}^{[illegible]}\big) - \nu^{r}_{\mathrm{ASE}}\Big(\big(\mathrm{D_E}^{[illegible]} + \mathrm{D_E}^{[illegible]} + \mathrm{D_E}^{[illegible]} + \mathrm{D_E}^{[illegible]} + \mathrm{D_E}^{[illegible]} + \mathrm{D_E}^{[illegible]} + \mathrm{D_E}^{[illegible]} + \mathrm{D_E}^{[illegible]}\big)\mathrm{P_E}^{[illegible]}\Big)$$

$$\frac{d\mathrm{P_E}^{[illegible]}}{dt} = \nu^{f}_{\mathrm{ASE}}\Big(\tfrac{1}{4}\Big(\big(\mathrm{T_E}^{[illegible]} + \mathrm{T_E}^{[illegible]} + \mathrm{T_E}^{[illegible]} + \mathrm{T_E}^{[illegible]} + \mathrm{T_E}^{[illegible]} + \mathrm{T_E}^{[illegible]} + \mathrm{T_E}^{[illegible]} + \mathrm{T_E}^{[illegible]}\big)\mathrm{W_E}^{\circ} + \big(\mathrm{T_E}^{[illegible]} + \cdots + \mathrm{T_E}^{[illegible]}\big)\mathrm{W_E}^{\bullet}\Big)\Big) + \nu^{r}_{\mathrm{PEO}}\big(\mathrm{P_O}^{[illegible]}\big) - \nu^{f}_{\mathrm{PEO}}\big(\mathrm{P_E}^{[illegible]}\big) - \nu^{r}_{\mathrm{ASE}}\Big(\big(\mathrm{D_E}^{[illegible]} + \mathrm{D_E}^{[illegible]} + \mathrm{D_E}^{[illegible]} + \mathrm{D_E}^{[illegible]} + \mathrm{D_E}^{[illegible]} + \mathrm{D_E}^{[illegible]} + \mathrm{D_E}^{[illegible]} + \mathrm{D_E}^{[illegible]}\big)\mathrm{P_E}^{[illegible]}\Big)$$

$$\frac{d\mathrm{P_E}^{[illegible]}}{dt} = \nu^{f}_{\mathrm{ASE}}\Big(\tfrac{1}{4}\Big(\big(\mathrm{T_E}^{[illegible]} + \cdots + \mathrm{T_E}^{[illegible]}\big)\mathrm{W_E}^{\circ} + \big(\mathrm{T_E}^{[illegible]} + \mathrm{T_E}^{[illegible]} + \mathrm{T_E}^{[illegible]} + \mathrm{T_E}^{[illegible]} + \mathrm{T_E}^{[illegible]} + \mathrm{T_E}^{[illegible]} + \mathrm{T_E}^{[illegible]} + \mathrm{T_E}^{[illegible]}\big)\mathrm{W_E}^{\bullet}\Big)\Big) + \nu^{r}_{\mathrm{PEO}}\big(\mathrm{P_O}^{[illegible]}\big) - \nu^{f}_{\mathrm{PEO}}\big(\mathrm{P_E}^{[illegible]}\big) - \nu^{r}_{\mathrm{ASE}}\Big(\big(\mathrm{D_E}^{[illegible]} + \mathrm{D_E}^{[illegible]} + \mathrm{D_E}^{[illegible]} + \mathrm{D_E}^{[illegible]} + \mathrm{D_E}^{[illegible]} + \mathrm{D_E}^{[illegible]} + \mathrm{D_E}^{[illegible]} + \mathrm{D_E}^{[illegible]}\big)\mathrm{P_E}^{[illegible]}\Big)$$

$$\frac{d\mathrm{P_E}^{[illegible]}}{dt} = \nu^{f}_{\mathrm{ASE}}\Big(\tfrac{1}{6}\Big(\big(\mathrm{T_E}^{[illegible]} + \cdots + \mathrm{T_E}^{[illegible]}\big)\mathrm{W_E}^{\circ} + \big(\mathrm{T_E}^{[illegible]} + \cdots + \mathrm{T_E}^{[illegible]}\big)\mathrm{W_E}^{\bullet}\Big)\Big) + \nu^{r}_{\mathrm{PEO}}\big(\mathrm{P_O}^{[illegible]}\big) - \nu^{f}_{\mathrm{PEO}}\big(\mathrm{P_E}^{[illegible]}\big) - \nu^{r}_{\mathrm{ASE}}\Big(\big(\mathrm{D_E}^{[illegible]} + \mathrm{D_E}^{[illegible]} + \mathrm{D_E}^{[illegible]} + \mathrm{D_E}^{[illegible]} + \mathrm{D_E}^{[illegible]} + \mathrm{D_E}^{[illegible]} + \mathrm{D_E}^{[illegible]} + \mathrm{D_E}^{[illegible]}\big)\mathrm{P_E}^{[illegible]}\Big)$$

$$\frac{d\mathrm{P_E}^{[illegible]}}{dt} = \nu^{f}_{\mathrm{ASE}}\Big(\tfrac{1}{6}\Big(\big(\mathrm{T_E}^{[illegible]} + \cdots + \mathrm{T_E}^{[illegible]}\big)\mathrm{W_E}^{\circ} + \big(\mathrm{T_E}^{[illegible]} + \cdots + \mathrm{T_E}^{[illegible]}\big)\mathrm{W_E}^{\bullet}\Big)\Big) + \nu^{r}_{\mathrm{PEO}}\big(\mathrm{P_O}^{[illegible]}\big) - \nu^{f}_{\mathrm{PEO}}\big(\mathrm{P_E}^{[illegible]}\big) - \nu^{r}_{\mathrm{ASE}}\Big(\big(\mathrm{D_E}^{[illegible]} + \mathrm{D_E}^{[illegible]} + \mathrm{D_E}^{[illegible]} + \mathrm{D_E}^{[illegible]} + \mathrm{D_E}^{[illegible]} + \mathrm{D_E}^{[illegible]} + \mathrm{D_E}^{[illegible]} + \mathrm{D_E}^{[illegible]}\big)\mathrm{P_E}^{[illegible]}\Big)$$

$$\frac{d\mathrm{P_E}^{[illegible]}}{dt} = \nu^{f}_{\mathrm{ASE}}\Big(\tfrac{1}{4}\Big(\big(\mathrm{T_E}^{[illegible]} + \mathrm{T_E}^{[illegible]} + \mathrm{T_E}^{[illegible]} + \mathrm{T_E}^{[illegible]} + \mathrm{T_E}^{[illegible]} + \mathrm{T_E}^{[illegible]} + \mathrm{T_E}^{[illegible]} + \mathrm{T_E}^{[illegible]}\big)\mathrm{W_E}^{\circ} + \big(\mathrm{T_E}^{[illegible]} + \cdots + \mathrm{T_E}^{[illegible]}\big)\mathrm{W_E}^{\bullet}\Big)\Big) + \nu^{r}_{\mathrm{PEO}}\big(\mathrm{P_O}^{[illegible]}\big) - \nu^{f}_{\mathrm{PEO}}\big(\mathrm{P_E}^{[illegible]}\big) - \nu^{r}_{\mathrm{ASE}}\Big(\big(\mathrm{D_E}^{[illegible]} + \mathrm{D_E}^{[illegible]} + \mathrm{D_E}^{[illegible]} + \mathrm{D_E}^{[illegible]} + \mathrm{D_E}^{[illegible]} + \mathrm{D_E}^{[illegible]} + \mathrm{D_E}^{[illegible]} + \mathrm{D_E}^{[illegible]}\big)\mathrm{P_E}^{[illegible]}\Big)$$

$$\frac{d\mathrm{P_E}^{[illegible]}}{dt} = \nu^{f}_{\mathrm{ASE}}\Big(\tfrac{1}{6}\Big(\big(\mathrm{T_E}^{[illegible]} + \cdots + \mathrm{T_E}^{[illegible]}\big)\mathrm{W_E}^{\circ} + \big(\mathrm{T_E}^{[illegible]} + \cdots + \mathrm{T_E}^{[illegible]}\big)\mathrm{W_E}^{\bullet}\Big)\Big) + \nu^{r}_{\mathrm{PEO}}\big(\mathrm{P_O}^{[illegible]}\big) - \nu^{f}_{\mathrm{PEO}}\big(\mathrm{P_E}^{[illegible]}\big) - \nu^{r}_{\mathrm{ASE}}\Big(\big(\mathrm{D_E}^{[illegible]} + \mathrm{D_E}^{[illegible]} + \mathrm{D_E}^{[illegible]} + \mathrm{D_E}^{[illegible]} + \mathrm{D_E}^{[illegible]} + \mathrm{D_E}^{[illegible]} + \mathrm{D_E}^{[illegible]} + \mathrm{D_E}^{[illegible]}\big)\mathrm{P_E}^{[illegible]}\Big)$$

$$\frac{d\mathrm{P_E}^{[illegible]}}{dt} = \nu^{f}_{\mathrm{ASE}}\Big(\tfrac{1}{4}\Big(\big(\mathrm{T_E}^{[illegible]} + \mathrm{T_E}^{[illegible]} + \mathrm{T_E}^{[illegible]} + \mathrm{T_E}^{[illegible]} + \mathrm{T_E}^{[illegible]} + \mathrm{T_E}^{[illegible]} + \mathrm{T_E}^{[illegible]} + \mathrm{T_E}^{[illegible]}\big)\mathrm{W_E}^{\circ} + \big(\mathrm{T_E}^{[illegible]} + \cdots + \mathrm{T_E}^{[illegible]}\big)\mathrm{W_E}^{\bullet}\Big)\Big) + \nu^{r}_{\mathrm{PEO}}\big(\mathrm{P_O}^{[illegible]}\big) - \nu^{f}_{\mathrm{PEO}}\big(\mathrm{P_E}^{[illegible]}\big) - \nu^{r}_{\mathrm{ASE}}\Big(\big(\mathrm{D_E}^{[illegible]} + \mathrm{D_E}^{[illegible]} + \mathrm{D_E}^{[illegible]} + \mathrm{D_E}^{[illegible]} + \mathrm{D_E}^{[illegible]} + \mathrm{D_E}^{[illegible]} + \mathrm{D_E}^{[illegible]} + \mathrm{D_E}^{[illegible]}\big)\mathrm{P_E}^{[illegible]}\Big)$$

$$\frac{d\mathrm{P_E}^{[illegible]}}{dt} = \nu^{f}_{\mathrm{ASE}}\Big(\tfrac{1}{4}\Big(\big(\mathrm{T_E}^{[illegible]} + \mathrm{T_E}^{[illegible]} + \mathrm{T_E}^{[illegible]} + \mathrm{T_E}^{[illegible]} + \mathrm{T_E}^{[illegible]} + \mathrm{T_E}^{[illegible]} + \mathrm{T_E}^{[illegible]} + \mathrm{T_E}^{[illegible]}\big)\mathrm{W_E}^{\circ} + \big(\mathrm{T_E}^{[illegible]} + \mathrm{T_E}^{[illegible]} + \mathrm{T_E}^{[illegible]} + \mathrm{T_E}^{[illegible]} + \mathrm{T_E}^{[illegible]} + \mathrm{T_E}^{[illegible]} +$$

$$\mathrm{T}_{\mathrm{E}\substack{\bullet\bullet\\\circ\bullet\\\bullet\circ}} + \mathrm{T}_{\mathrm{E}\substack{\bullet\bullet\\\circ\circ\\\bullet\bullet}} + \mathrm{T}_{\mathrm{E}\substack{\bullet\circ\\\bullet\bullet\\\bullet\bullet}} + \mathrm{T}_{\mathrm{E}\substack{\bullet\bullet\\\circ\bullet\\\circ\circ}} + \mathrm{T}_{\mathrm{E}\substack{\bullet\bullet\\\circ\circ\\\circ\bullet}} + \mathrm{T}_{\mathrm{E}\substack{\bullet\circ\\\circ\bullet\\\circ\bullet}} + \mathrm{T}_{\mathrm{E}\substack{\circ\bullet\\\bullet\bullet\\\bullet\circ}} + \mathrm{T}_{\mathrm{E}\substack{\circ\bullet\\\bullet\circ\\\bullet\bullet}} + \mathrm{T}_{\mathrm{E}\substack{\circ\circ\\\bullet\bullet\\\bullet\bullet}} + \mathrm{T}_{\mathrm{E}\substack{\circ\bullet\\\bullet\bullet\\\circ\circ}} + \mathrm{T}_{\mathrm{E}\substack{\circ\bullet\\\bullet\circ\\\circ\bullet}} + \mathrm{T}_{\mathrm{E}\substack{\circ\circ\\\bullet\bullet\\\circ\bullet}} + \mathrm{T}_{\mathrm{E}\substack{\circ\bullet\\\circ\bullet\\\bullet\circ}} + \mathrm{T}_{\mathrm{E}\substack{\circ\bullet\\\circ\circ\\\bullet\bullet}} + \mathrm{T}_{\mathrm{E}\substack{\circ\circ\\\circ\bullet\\\bullet\bullet}} + \mathrm{T}_{\mathrm{E}\substack{\circ\bullet\\\circ\bullet\\\circ\circ}} + \mathrm{T}_{\mathrm{E}\substack{\circ\bullet\\\circ\circ\\\circ\bullet}} +$$
$$\mathrm{T}_{\mathrm{E}\substack{\circ\circ\\\circ\bullet\\\circ\bullet}})\mathrm{W}_{\mathrm{E}\bullet})) + \underset{\mathrm{PEO}}{\nu_r}(\mathrm{P}_{\mathrm{O}\substack{\bullet\\\bullet\\\bullet\\\circ}}) - \underset{\mathrm{PEO}}{\nu_f}(\mathrm{P}_{\mathrm{E}\substack{\bullet\\\bullet\\\bullet\\\circ}}) - \underset{\mathrm{ASE}}{\nu_r}((\mathrm{D}_{\mathrm{E}\substack{\bullet\\\bullet\\\bullet}} + \mathrm{D}_{\mathrm{E}\substack{\bullet\\\bullet\\\circ}} + \mathrm{D}_{\mathrm{E}\substack{\bullet\\\circ\\\bullet}} + \mathrm{D}_{\mathrm{E}\substack{\bullet\\\circ\\\circ}} + \mathrm{D}_{\mathrm{E}\substack{\circ\\\bullet\\\bullet}} + \mathrm{D}_{\mathrm{E}\substack{\circ\\\bullet\\\circ}} + \mathrm{D}_{\mathrm{E}\substack{\circ\\\circ\\\bullet}} + \mathrm{D}_{\mathrm{E}\substack{\circ\\\circ\\\circ}})\mathrm{P}_{\mathrm{E}\substack{\bullet\\\bullet\\\bullet\\\circ}})$$

$$\frac{d\mathrm{P}_{\mathrm{E}\substack{\bullet\\\bullet\\\bullet\\\bullet}}}{dt} = \underset{\mathrm{ASE}}{\nu_f}((\mathrm{T}_{\mathrm{E}\substack{\bullet\bullet\\\bullet\bullet\\\bullet\bullet}} + \mathrm{T}_{\mathrm{E}\substack{\bullet\bullet\\\bullet\bullet\\\circ\bullet}} + \mathrm{T}_{\mathrm{E}\substack{\bullet\bullet\\\circ\bullet\\\bullet\bullet}} + \mathrm{T}_{\mathrm{E}\substack{\bullet\bullet\\\circ\bullet\\\circ\bullet}} + \mathrm{T}_{\mathrm{E}\substack{\circ\bullet\\\bullet\bullet\\\bullet\bullet}} + \mathrm{T}_{\mathrm{E}\substack{\circ\bullet\\\bullet\bullet\\\circ\bullet}} + \mathrm{T}_{\mathrm{E}\substack{\circ\bullet\\\circ\bullet\\\bullet\bullet}} + \mathrm{T}_{\mathrm{E}\substack{\circ\bullet\\\circ\bullet\\\circ\bullet}})\mathrm{W}_{\mathrm{E}\bullet}) + \underset{\mathrm{PEO}}{\nu_r}(\mathrm{P}_{\mathrm{O}\substack{\bullet\\\bullet\\\bullet\\\bullet}}) - \underset{\mathrm{PEO}}{\nu_f}(\mathrm{P}_{\mathrm{E}\substack{\bullet\\\bullet\\\bullet\\\bullet}}) - \underset{\mathrm{ASE}}{\nu_r}((\mathrm{D}_{\mathrm{E}\substack{\bullet\\\bullet\\\bullet}} + \mathrm{D}_{\mathrm{E}\substack{\bullet\\\bullet\\\circ}} + \mathrm{D}_{\mathrm{E}\substack{\bullet\\\circ\\\bullet}} + \mathrm{D}_{\mathrm{E}\substack{\bullet\\\circ\\\circ}} +$$
$$\mathrm{D}_{\mathrm{E}\substack{\circ\\\bullet\\\bullet}} + \mathrm{D}_{\mathrm{E}\substack{\circ\\\bullet\\\circ}} + \mathrm{D}_{\mathrm{E}\substack{\circ\\\circ\\\bullet}} + \mathrm{D}_{\mathrm{E}\substack{\circ\\\circ\\\circ}})\mathrm{P}_{\mathrm{E}\substack{\bullet\\\bullet\\\bullet\\\bullet}})$$

$$\frac{d\mathrm{P}_{\mathrm{M}\substack{\circ\\\circ\\\circ\\\circ}}}{dt} = \underset{\mathrm{POM}}{\nu_f}(\mathrm{P}_{\mathrm{O}\substack{\circ\\\circ\\\circ\\\circ}}) + \underset{\mathrm{PMS}}{\nu_r}(\mathrm{P}_{\mathrm{S}\substack{\circ\\\circ\\\circ\\\circ}}) - \underset{\mathrm{PMS}}{\nu_f}(\mathrm{P}_{\mathrm{M}\substack{\circ\\\circ\\\circ\\\circ}})$$

$$\frac{d\mathrm{P}_{\mathrm{M}\substack{\circ\\\circ\\\circ\\\bullet}}}{dt} = \underset{\mathrm{POM}}{\nu_f}(\mathrm{P}_{\mathrm{O}\substack{\circ\\\circ\\\circ\\\bullet}}) + \underset{\mathrm{PMS}}{\nu_r}(\mathrm{P}_{\mathrm{S}\substack{\circ\\\circ\\\circ\\\bullet}}) - \underset{\mathrm{PMS}}{\nu_f}(\mathrm{P}_{\mathrm{M}\substack{\circ\\\circ\\\circ\\\bullet}})$$

$$\frac{d\mathrm{P}_{\mathrm{M}\substack{\circ\\\circ\\\bullet\\\circ}}}{dt} = \underset{\mathrm{POM}}{\nu_f}(\mathrm{P}_{\mathrm{O}\substack{\circ\\\circ\\\bullet\\\circ}}) + \underset{\mathrm{PMS}}{\nu_r}(\mathrm{P}_{\mathrm{S}\substack{\circ\\\circ\\\bullet\\\circ}}) - \underset{\mathrm{PMS}}{\nu_f}(\mathrm{P}_{\mathrm{M}\substack{\circ\\\circ\\\bullet\\\circ}})$$

$$\frac{d\mathrm{P}_{\mathrm{M}\substack{\circ\\\circ\\\bullet\\\bullet}}}{dt} = \underset{\mathrm{POM}}{\nu_f}(\mathrm{P}_{\mathrm{O}\substack{\circ\\\circ\\\bullet\\\bullet}}) + \underset{\mathrm{PMS}}{\nu_r}(\mathrm{P}_{\mathrm{S}\substack{\circ\\\circ\\\bullet\\\bullet}}) - \underset{\mathrm{PMS}}{\nu_f}(\mathrm{P}_{\mathrm{M}\substack{\circ\\\circ\\\bullet\\\bullet}})$$

$$\frac{d\mathrm{P}_{\mathrm{M}\substack{\circ\\\bullet\\\circ\\\circ}}}{dt} = \underset{\mathrm{POM}}{\nu_f}(\mathrm{P}_{\mathrm{O}\substack{\circ\\\bullet\\\circ\\\circ}}) + \underset{\mathrm{PMS}}{\nu_r}(\mathrm{P}_{\mathrm{S}\substack{\circ\\\bullet\\\circ\\\circ}}) - \underset{\mathrm{PMS}}{\nu_f}(\mathrm{P}_{\mathrm{M}\substack{\circ\\\bullet\\\circ\\\circ}})$$

$$\frac{d\mathrm{P}_{\mathrm{M}\substack{\circ\\\bullet\\\circ\\\bullet}}}{dt} = \underset{\mathrm{POM}}{\nu_f}(\mathrm{P}_{\mathrm{O}\substack{\circ\\\bullet\\\circ\\\bullet}}) + \underset{\mathrm{PMS}}{\nu_r}(\mathrm{P}_{\mathrm{S}\substack{\circ\\\bullet\\\circ\\\bullet}}) - \underset{\mathrm{PMS}}{\nu_f}(\mathrm{P}_{\mathrm{M}\substack{\circ\\\bullet\\\circ\\\bullet}})$$

$$\frac{d\mathrm{P}_{\mathrm{M}\substack{\circ\\\bullet\\\bullet\\\circ}}}{dt} = \underset{\mathrm{POM}}{\nu_f}(\mathrm{P}_{\mathrm{O}\substack{\circ\\\bullet\\\bullet\\\circ}}) + \underset{\mathrm{PMS}}{\nu_r}(\mathrm{P}_{\mathrm{S}\substack{\circ\\\bullet\\\bullet\\\circ}}) - \underset{\mathrm{PMS}}{\nu_f}(\mathrm{P}_{\mathrm{M}\substack{\circ\\\bullet\\\bullet\\\circ}})$$

$$\frac{d\mathrm{P}_{\mathrm{M}\substack{\circ\\\bullet\\\bullet\\\bullet}}}{dt} = \underset{\mathrm{POM}}{\nu_f}(\mathrm{P}_{\mathrm{O}\substack{\circ\\\bullet\\\bullet\\\bullet}}) + \underset{\mathrm{PMS}}{\nu_r}(\mathrm{P}_{\mathrm{S}\substack{\circ\\\bullet\\\bullet\\\bullet}}) - \underset{\mathrm{PMS}}{\nu_f}(\mathrm{P}_{\mathrm{M}\substack{\circ\\\bullet\\\bullet\\\bullet}})$$

$$\frac{d\mathrm{P}_{\mathrm{M}\substack{\bullet\\\circ\\\circ\\\circ}}}{dt} = \underset{\mathrm{POM}}{\nu_f}(\mathrm{P}_{\mathrm{O}\substack{\bullet\\\circ\\\circ\\\circ}}) + \underset{\mathrm{PMS}}{\nu_r}(\mathrm{P}_{\mathrm{S}\substack{\bullet\\\circ\\\circ\\\circ}}) - \underset{\mathrm{PMS}}{\nu_f}(\mathrm{P}_{\mathrm{M}\substack{\bullet\\\circ\\\circ\\\circ}})$$

$$\frac{d\mathrm{P}_{\mathrm{M}\substack{\bullet\\\circ\\\circ\\\bullet}}}{dt} = \underset{\mathrm{POM}}{\nu_f}(\mathrm{P}_{\mathrm{O}\substack{\bullet\\\circ\\\circ\\\bullet}}) + \underset{\mathrm{PMS}}{\nu_r}(\mathrm{P}_{\mathrm{S}\substack{\bullet\\\circ\\\circ\\\bullet}}) - \underset{\mathrm{PMS}}{\nu_f}(\mathrm{P}_{\mathrm{M}\substack{\bullet\\\circ\\\circ\\\bullet}})$$

$$\frac{d\mathrm{P}_{\mathrm{M}\substack{\bullet\\\circ\\\bullet\\\circ}}}{dt} = \underset{\mathrm{POM}}{\nu_f}(\mathrm{P}_{\mathrm{O}\substack{\bullet\\\circ\\\bullet\\\circ}}) + \underset{\mathrm{PMS}}{\nu_r}(\mathrm{P}_{\mathrm{S}\substack{\bullet\\\circ\\\bullet\\\circ}}) - \underset{\mathrm{PMS}}{\nu_f}(\mathrm{P}_{\mathrm{M}\substack{\bullet\\\circ\\\bullet\\\circ}})$$

$$\frac{d\mathrm{P}_{\mathrm{M}\substack{\bullet\\\circ\\\bullet\\\bullet}}}{dt} = \underset{\mathrm{POM}}{\nu_f}(\mathrm{P}_{\mathrm{O}\substack{\bullet\\\circ\\\bullet\\\bullet}}) + \underset{\mathrm{PMS}}{\nu_r}(\mathrm{P}_{\mathrm{S}\substack{\bullet\\\circ\\\bullet\\\bullet}}) - \underset{\mathrm{PMS}}{\nu_f}(\mathrm{P}_{\mathrm{M}\substack{\bullet\\\circ\\\bullet\\\bullet}})$$

$$\frac{d\mathrm{P}_{\mathrm{M}\substack{\bullet\\\bullet\\\circ\\\circ}}}{dt} = \underset{\mathrm{POM}}{\nu_f}(\mathrm{P}_{\mathrm{O}\substack{\bullet\\\bullet\\\circ\\\circ}}) + \underset{\mathrm{PMS}}{\nu_r}(\mathrm{P}_{\mathrm{S}\substack{\bullet\\\bullet\\\circ\\\circ}}) - \underset{\mathrm{PMS}}{\nu_f}(\mathrm{P}_{\mathrm{M}\substack{\bullet\\\bullet\\\circ\\\circ}})$$

$$\frac{d\mathrm{P}_{\mathrm{M}\substack{\bullet\\\bullet\\\circ\\\bullet}}}{dt} = \underset{\mathrm{POM}}{\nu_f}(\mathrm{P}_{\mathrm{O}\substack{\bullet\\\bullet\\\circ\\\bullet}}) + \underset{\mathrm{PMS}}{\nu_r}(\mathrm{P}_{\mathrm{S}\substack{\bullet\\\bullet\\\circ\\\bullet}}) - \underset{\mathrm{PMS}}{\nu_f}(\mathrm{P}_{\mathrm{M}\substack{\bullet\\\bullet\\\circ\\\bullet}})$$

$$\frac{d\mathrm{P}_{\mathrm{M}\substack{\bullet\\\bullet\\\bullet\\\circ}}}{dt} = \underset{\mathrm{POM}}{\nu_f}(\mathrm{P}_{\mathrm{O}\substack{\bullet\\\bullet\\\bullet\\\circ}}) + \underset{\mathrm{PMS}}{\nu_r}(\mathrm{P}_{\mathrm{S}\substack{\bullet\\\bullet\\\bullet\\\circ}}) - \underset{\mathrm{PMS}}{\nu_f}(\mathrm{P}_{\mathrm{M}\substack{\bullet\\\bullet\\\bullet\\\circ}})$$

$$\frac{d\mathrm{P}_{\mathrm{M}\substack{\bullet\\\bullet\\\bullet\\\bullet}}}{dt} = \underset{\mathrm{POM}}{\nu_f}(\mathrm{P}_{\mathrm{O}\substack{\bullet\\\bullet\\\bullet\\\bullet}}) + \underset{\mathrm{PMS}}{\nu_r}(\mathrm{P}_{\mathrm{S}\substack{\bullet\\\bullet\\\bullet\\\bullet}}) - \underset{\mathrm{PMS}}{\nu_f}(\mathrm{P}_{\mathrm{M}\substack{\bullet\\\bullet\\\bullet\\\bullet}})$$

$$\frac{d\mathrm{P_O}^{\substack{\circ\\\circ\\\circ\\\circ}}}{dt} = \overset{\nu_f}{\scriptstyle\mathrm{PEO}}\big(\mathrm{P_E}^{\substack{\circ\\\circ\\\circ\\\circ}}\big) - \overset{\nu_f}{\scriptstyle\mathrm{POM}}\big(\mathrm{P_O}^{\substack{\circ\\\circ\\\circ\\\circ}}\big) - \overset{\nu_r}{\scriptstyle\mathrm{PEO}}\big(\mathrm{P_O}^{\substack{\circ\\\circ\\\circ\\\circ}}\big)$$

$$\frac{d\mathrm{P_O}^{\substack{\circ\\\circ\\\circ\\\bullet}}}{dt} = \overset{\nu_f}{\scriptstyle\mathrm{PEO}}\big(\mathrm{P_E}^{\substack{\circ\\\circ\\\circ\\\bullet}}\big) - \overset{\nu_f}{\scriptstyle\mathrm{POM}}\big(\mathrm{P_O}^{\substack{\circ\\\circ\\\circ\\\bullet}}\big) - \overset{\nu_r}{\scriptstyle\mathrm{PEO}}\big(\mathrm{P_O}^{\substack{\circ\\\circ\\\circ\\\bullet}}\big)$$

$$\frac{d\mathrm{P_O}^{\substack{\circ\\\circ\\\bullet\\\circ}}}{dt} = \overset{\nu_f}{\scriptstyle\mathrm{PEO}}\big(\mathrm{P_E}^{\substack{\circ\\\circ\\\bullet\\\circ}}\big) - \overset{\nu_f}{\scriptstyle\mathrm{POM}}\big(\mathrm{P_O}^{\substack{\circ\\\circ\\\bullet\\\circ}}\big) - \overset{\nu_r}{\scriptstyle\mathrm{PEO}}\big(\mathrm{P_O}^{\substack{\circ\\\circ\\\bullet\\\circ}}\big)$$

$$\frac{d\mathrm{P_O}^{\substack{\circ\\\circ\\\bullet\\\bullet}}}{dt} = \overset{\nu_f}{\scriptstyle\mathrm{PEO}}\big(\mathrm{P_E}^{\substack{\circ\\\circ\\\bullet\\\bullet}}\big) - \overset{\nu_f}{\scriptstyle\mathrm{POM}}\big(\mathrm{P_O}^{\substack{\circ\\\circ\\\bullet\\\bullet}}\big) - \overset{\nu_r}{\scriptstyle\mathrm{PEO}}\big(\mathrm{P_O}^{\substack{\circ\\\circ\\\bullet\\\bullet}}\big)$$

$$\frac{d\mathrm{P_O}^{\substack{\circ\\\bullet\\\circ\\\circ}}}{dt} = \overset{\nu_f}{\scriptstyle\mathrm{PEO}}\big(\mathrm{P_E}^{\substack{\circ\\\bullet\\\circ\\\circ}}\big) - \overset{\nu_f}{\scriptstyle\mathrm{POM}}\big(\mathrm{P_O}^{\substack{\circ\\\bullet\\\circ\\\circ}}\big) - \overset{\nu_r}{\scriptstyle\mathrm{PEO}}\big(\mathrm{P_O}^{\substack{\circ\\\bullet\\\circ\\\circ}}\big)$$

$$\frac{d\mathrm{P_O}^{\substack{\circ\\\bullet\\\circ\\\bullet}}}{dt} = \overset{\nu_f}{\scriptstyle\mathrm{PEO}}\big(\mathrm{P_E}^{\substack{\circ\\\bullet\\\circ\\\bullet}}\big) - \overset{\nu_f}{\scriptstyle\mathrm{POM}}\big(\mathrm{P_O}^{\substack{\circ\\\bullet\\\circ\\\bullet}}\big) - \overset{\nu_r}{\scriptstyle\mathrm{PEO}}\big(\mathrm{P_O}^{\substack{\circ\\\bullet\\\circ\\\bullet}}\big)$$

$$\frac{d\mathrm{P_O}^{\substack{\circ\\\bullet\\\bullet\\\circ}}}{dt} = \overset{\nu_f}{\scriptstyle\mathrm{PEO}}\big(\mathrm{P_E}^{\substack{\circ\\\bullet\\\bullet\\\circ}}\big) - \overset{\nu_f}{\scriptstyle\mathrm{POM}}\big(\mathrm{P_O}^{\substack{\circ\\\bullet\\\bullet\\\circ}}\big) - \overset{\nu_r}{\scriptstyle\mathrm{PEO}}\big(\mathrm{P_O}^{\substack{\circ\\\bullet\\\bullet\\\circ}}\big)$$

$$\frac{d\mathrm{P_O}^{\substack{\circ\\\bullet\\\bullet\\\bullet}}}{dt} = \overset{\nu_f}{\scriptstyle\mathrm{PEO}}\big(\mathrm{P_E}^{\substack{\circ\\\bullet\\\bullet\\\bullet}}\big) - \overset{\nu_f}{\scriptstyle\mathrm{POM}}\big(\mathrm{P_O}^{\substack{\circ\\\bullet\\\bullet\\\bullet}}\big) - \overset{\nu_r}{\scriptstyle\mathrm{PEO}}\big(\mathrm{P_O}^{\substack{\circ\\\bullet\\\bullet\\\bullet}}\big)$$

$$\frac{d\mathrm{P_O}^{\substack{\bullet\\\circ\\\circ\\\circ}}}{dt} = \overset{\nu_f}{\scriptstyle\mathrm{PEO}}\big(\mathrm{P_E}^{\substack{\bullet\\\circ\\\circ\\\circ}}\big) - \overset{\nu_f}{\scriptstyle\mathrm{POM}}\big(\mathrm{P_O}^{\substack{\bullet\\\circ\\\circ\\\circ}}\big) - \overset{\nu_r}{\scriptstyle\mathrm{PEO}}\big(\mathrm{P_O}^{\substack{\bullet\\\circ\\\circ\\\circ}}\big)$$

$$\frac{d\mathrm{P_O}^{\substack{\bullet\\\circ\\\circ\\\bullet}}}{dt} = \overset{\nu_f}{\scriptstyle\mathrm{PEO}}\big(\mathrm{P_E}^{\substack{\bullet\\\circ\\\circ\\\bullet}}\big) - \overset{\nu_f}{\scriptstyle\mathrm{POM}}\big(\mathrm{P_O}^{\substack{\bullet\\\circ\\\circ\\\bullet}}\big) - \overset{\nu_r}{\scriptstyle\mathrm{PEO}}\big(\mathrm{P_O}^{\substack{\bullet\\\circ\\\circ\\\bullet}}\big)$$

$$\frac{d\mathrm{P_O}^{\substack{\bullet\\\circ\\\bullet\\\circ}}}{dt} = \overset{\nu_f}{\scriptstyle\mathrm{PEO}}\big(\mathrm{P_E}^{\substack{\bullet\\\circ\\\bullet\\\circ}}\big) - \overset{\nu_f}{\scriptstyle\mathrm{POM}}\big(\mathrm{P_O}^{\substack{\bullet\\\circ\\\bullet\\\circ}}\big) - \overset{\nu_r}{\scriptstyle\mathrm{PEO}}\big(\mathrm{P_O}^{\substack{\bullet\\\circ\\\bullet\\\circ}}\big)$$

$$\frac{d\mathrm{P_O}^{\substack{\bullet\\\circ\\\bullet\\\bullet}}}{dt} = \overset{\nu_f}{\scriptstyle\mathrm{PEO}}\big(\mathrm{P_E}^{\substack{\bullet\\\circ\\\bullet\\\bullet}}\big) - \overset{\nu_f}{\scriptstyle\mathrm{POM}}\big(\mathrm{P_O}^{\substack{\bullet\\\circ\\\bullet\\\bullet}}\big) - \overset{\nu_r}{\scriptstyle\mathrm{PEO}}\big(\mathrm{P_O}^{\substack{\bullet\\\circ\\\bullet\\\bullet}}\big)$$

$$\frac{d\mathrm{P_O}^{\substack{\bullet\\\bullet\\\circ\\\circ}}}{dt} = \overset{\nu_f}{\scriptstyle\mathrm{PEO}}\big(\mathrm{P_E}^{\substack{\bullet\\\bullet\\\circ\\\circ}}\big) - \overset{\nu_f}{\scriptstyle\mathrm{POM}}\big(\mathrm{P_O}^{\substack{\bullet\\\bullet\\\circ\\\circ}}\big) - \overset{\nu_r}{\scriptstyle\mathrm{PEO}}\big(\mathrm{P_O}^{\substack{\bullet\\\bullet\\\circ\\\circ}}\big)$$

$$\frac{d\mathrm{P_O}^{\substack{\bullet\\\bullet\\\circ\\\bullet}}}{dt} = \overset{\nu_f}{\scriptstyle\mathrm{PEO}}\big(\mathrm{P_E}^{\substack{\bullet\\\bullet\\\circ\\\bullet}}\big) - \overset{\nu_f}{\scriptstyle\mathrm{POM}}\big(\mathrm{P_O}^{\substack{\bullet\\\bullet\\\circ\\\bullet}}\big) - \overset{\nu_r}{\scriptstyle\mathrm{PEO}}\big(\mathrm{P_O}^{\substack{\bullet\\\bullet\\\circ\\\bullet}}\big)$$

$$\frac{d\mathrm{P_O}^{\substack{\bullet\\\bullet\\\bullet\\\circ}}}{dt} = \overset{\nu_f}{\scriptstyle\mathrm{PEO}}\big(\mathrm{P_E}^{\substack{\bullet\\\bullet\\\bullet\\\circ}}\big) - \overset{\nu_f}{\scriptstyle\mathrm{POM}}\big(\mathrm{P_O}^{\substack{\bullet\\\bullet\\\bullet\\\circ}}\big) - \overset{\nu_r}{\scriptstyle\mathrm{PEO}}\big(\mathrm{P_O}^{\substack{\bullet\\\bullet\\\bullet\\\circ}}\big)$$

$$\frac{d\mathrm{P_O}^{\substack{\bullet\\\bullet\\\bullet\\\bullet}}}{dt} = \overset{\nu_f}{\scriptstyle\mathrm{PEO}}\big(\mathrm{P_E}^{\substack{\bullet\\\bullet\\\bullet\\\bullet}}\big) - \overset{\nu_f}{\scriptstyle\mathrm{POM}}\big(\mathrm{P_O}^{\substack{\bullet\\\bullet\\\bullet\\\bullet}}\big) - \overset{\nu_r}{\scriptstyle\mathrm{PEO}}\big(\mathrm{P_O}^{\substack{\bullet\\\bullet\\\bullet\\\bullet}}\big)$$

$$\frac{d\mathrm{P_S}^{\substack{\circ\\\circ\\\circ\\\circ}}}{dt} = \overset{\nu_f}{\scriptstyle\mathrm{PMS}}\big(\mathrm{P_M}^{\substack{\circ\\\circ\\\circ\\\circ}}\big) + \overset{\nu_r}{\scriptstyle\mathrm{ASs}}\Big(\big(\mathrm{T_S}^{\substack{\bullet\circ\\\bullet\circ\\\bullet\circ}} + \mathrm{T_S}^{\substack{\bullet\circ\\\bullet\circ\\\circ\circ}} + \mathrm{T_S}^{\substack{\bullet\circ\\\circ\circ\\\bullet\circ}} + \mathrm{T_S}^{\substack{\bullet\circ\\\circ\circ\\\circ\circ}} + \mathrm{T_S}^{\substack{\circ\circ\\\bullet\circ\\\bullet\circ}} + \mathrm{T_S}^{\substack{\circ\circ\\\bullet\circ\\\circ\circ}} + \mathrm{T_S}^{\substack{\circ\circ\\\circ\circ\\\bullet\circ}} + \mathrm{T_S}^{\substack{\circ\circ\\\circ\circ\\\circ\circ}}\big)\mathrm{W_S}^{\circ}\Big) - \overset{\nu_f}{\scriptstyle\mathrm{ASs}}\Big(\big(\mathrm{D_S}^{\substack{\bullet\\\bullet\\\bullet}} + \mathrm{D_S}^{\substack{\bullet\\\bullet\\\circ}} + \mathrm{D_S}^{\substack{\bullet\\\circ\\\bullet}} + \mathrm{D_S}^{\substack{\bullet\\\circ\\\circ}} + \mathrm{D_S}^{\substack{\circ\\\bullet\\\bullet}} + \mathrm{D_S}^{\substack{\circ\\\bullet\\\circ}} +$$
$$\mathrm{D_S}^{\substack{\circ\\\circ\\\bullet}} + \mathrm{D_S}^{\substack{\circ\\\circ\\\circ}}\big)\mathrm{P_S}^{\substack{\circ\\\circ\\\circ\\\circ}}\Big) - \overset{\nu_r}{\scriptstyle\mathrm{PMS}}\big(\mathrm{P_S}^{\substack{\circ\\\circ\\\circ\\\circ}}\big)$$

$$\frac{d\mathrm{P_S}^{\substack{\circ\\\circ\\\circ\\\bullet}}}{dt} = \overset{\nu_f}{\scriptstyle\mathrm{PMS}}\big(\mathrm{P_M}^{\substack{\circ\\\circ\\\circ\\\bullet}}\big) + \overset{\nu_r}{\scriptstyle\mathrm{ASs}}\Big(\frac{1}{4}\Big(\big(\mathrm{T_S}^{\substack{\bullet\bullet\\\bullet\circ\\\bullet\circ}} + \mathrm{T_S}^{\substack{\bullet\circ\\\bullet\bullet\\\bullet\circ}} + \mathrm{T_S}^{\substack{\bullet\circ\\\bullet\circ\\\bullet\bullet}} + \mathrm{T_S}^{\substack{\bullet\bullet\\\bullet\circ\\\circ\circ}} + \mathrm{T_S}^{\substack{\bullet\circ\\\bullet\bullet\\\circ\circ}} + \mathrm{T_S}^{\substack{\bullet\circ\\\bullet\circ\\\circ\bullet}} + \mathrm{T_S}^{\substack{\bullet\bullet\\\circ\circ\\\bullet\circ}} + \mathrm{T_S}^{\substack{\bullet\circ\\\circ\bullet\\\bullet\circ}} + \mathrm{T_S}^{\substack{\bullet\circ\\\circ\circ\\\bullet\bullet}} + \mathrm{T_S}^{\substack{\bullet\bullet\\\circ\circ\\\circ\circ}} + \mathrm{T_S}^{\substack{\bullet\circ\\\circ\bullet\\\circ\circ}} + \mathrm{T_S}^{\substack{\bullet\circ\\\circ\circ\\\circ\bullet}} + \mathrm{T_S}^{\substack{\circ\bullet\\\bullet\circ\\\bullet\circ}} + \mathrm{T_S}^{\substack{\circ\circ\\\bullet\bullet\\\bullet\circ}} +$$

Notation: dot patterns are written row by row, top to bottom, separated by "/" (∘ = open, • = filled).

$\mathrm{T_S}\{\circ\circ/\bullet\circ/\bullet\bullet\} + \mathrm{T_S}\{\circ\bullet/\bullet\circ/\circ\circ\} + \mathrm{T_S}\{\circ\circ/\bullet\bullet/\circ\circ\} + \mathrm{T_S}\{\circ\circ/\bullet\circ/\circ\bullet\} + \mathrm{T_S}\{\circ\bullet/\circ\circ/\bullet\circ\} + \mathrm{T_S}\{\circ\circ/\circ\bullet/\bullet\circ\} + \mathrm{T_S}\{\circ\circ/\circ\circ/\bullet\bullet\} + \mathrm{T_S}\{\circ\bullet/\circ\circ/\circ\circ\} + \mathrm{T_S}\{\circ\circ/\circ\bullet/\circ\circ\} + \mathrm{T_S}\{\circ\circ/\circ\circ/\circ\bullet\})\mathrm{W_S}\circ + (\mathrm{T_S}[illegible] + \mathrm{T_S}[illegible] + \mathrm{T_S}[illegible] + \mathrm{T_S}[illegible] + \mathrm{T_S}[illegible] + \mathrm{T_S}[illegible] + \mathrm{T_S}[illegible] +$
$\mathrm{T_S}\{\circ\circ/\circ\circ/\circ\circ\})\mathrm{W_S}\bullet)) - \nu^{f}_{\mathrm{ASs}}((\mathrm{D_S}\{\bullet/\bullet/\bullet\} + \mathrm{D_S}\{\bullet/\bullet/\circ\} + \mathrm{D_S}\{\bullet/\circ/\bullet\} + \mathrm{D_S}\{\bullet/\circ/\circ\} + \mathrm{D_S}\{\circ/\bullet/\bullet\} + \mathrm{D_S}\{\circ/\bullet/\circ\} + \mathrm{D_S}\{\circ/\circ/\bullet\} + \mathrm{D_S}\{\circ/\circ/\circ\})\mathrm{P_S}\{\circ/\circ/\circ/\bullet\}) - \nu^{r}_{\mathrm{PMS}}(\mathrm{P_S}[illegible])$

$$\frac{d\mathrm{P_S}\{\circ/\circ/\bullet/\circ\}}{dt} = \nu^{f}_{\mathrm{PMS}}(\mathrm{P_M}\{\circ/\circ/\bullet/\circ\}) + \nu^{r}_{\mathrm{ASs}}(\tfrac{1}{4}((\mathrm{T_S}\{\bullet\bullet/\bullet\circ/\bullet\circ\} + \mathrm{T_S}\{\bullet\circ/\bullet\bullet/\bullet\circ\} + \mathrm{T_S}\{\bullet\circ/\bullet\circ/\bullet\bullet\} + \mathrm{T_S}\{\bullet\bullet/\bullet\circ/\circ\circ\} + \mathrm{T_S}\{\bullet\circ/\bullet\bullet/\circ\circ\} + \mathrm{T_S}\{\bullet\circ/\bullet\circ/\circ\bullet\} + \mathrm{T_S}[illegible] + \ldots [illegible])\mathrm{W_S}\circ + (\ldots [illegible])\mathrm{W_S}\bullet)) - \nu^{f}_{\mathrm{ASs}}((\mathrm{D_S}[illegible] + \ldots)\mathrm{P_S}[illegible]) - \nu^{r}_{\mathrm{PMS}}(\mathrm{P_S}[illegible])$$

$$\frac{d\mathrm{P_S}[illegible]}{dt} = \nu^{f}_{\mathrm{PMS}}(\mathrm{P_M}[illegible]) + \nu^{r}_{\mathrm{ASs}}(\tfrac{1}{6}((\mathrm{T_S}[illegible] + \ldots [illegible])\mathrm{W_S}\circ + (\ldots [illegible])\mathrm{W_S}\bullet)) - \nu^{f}_{\mathrm{ASs}}((\mathrm{D_S}[illegible] + \ldots)\mathrm{P_S}[illegible]) - \nu^{r}_{\mathrm{PMS}}(\mathrm{P_S}[illegible])$$

$$\frac{d\mathrm{P_S}[illegible]}{dt} = \nu^{f}_{\mathrm{PMS}}(\mathrm{P_M}[illegible]) + \nu^{r}_{\mathrm{ASs}}(\tfrac{1}{4}((\mathrm{T_S}[illegible] + \ldots [illegible])\mathrm{W_S}\circ + (\ldots [illegible])\mathrm{W_S}\bullet)) - \nu^{f}_{\mathrm{ASs}}((\mathrm{D_S}[illegible] + \ldots)\mathrm{P_S}[illegible]) - \nu^{r}_{\mathrm{PMS}}(\mathrm{P_S}[illegible])$$

$$\frac{d\mathrm{P_S}[illegible]}{dt} = \nu^{f}_{\mathrm{PMS}}(\mathrm{P_M}[illegible]) + \nu^{r}_{\mathrm{ASs}}(\tfrac{1}{6}((\mathrm{T_S}[illegible] + \ldots [illegible])\mathrm{W_S}\circ + (\ldots [illegible])\mathrm{W_S}\bullet)) - \nu^{f}_{\mathrm{ASs}}((\mathrm{D_S}[illegible] + \ldots)\mathrm{P_S}[illegible]) - \nu^{r}_{\mathrm{PMS}}(\mathrm{P_S}[illegible])$$

$$\frac{d\mathrm{P_S}[illegible]}{dt} = \nu^{f}_{\mathrm{PMS}}(\mathrm{P_M}[illegible]) + \nu^{r}_{\mathrm{ASs}}(\tfrac{1}{6}((\mathrm{T_S}[illegible] + \ldots [illegible])\mathrm{W_S}\circ + (\ldots [illegible])\mathrm{W_S}\bullet)) - \nu^{f}_{\mathrm{ASs}}((\mathrm{D_S}[illegible] + \ldots)\mathrm{P_S}[illegible]) - \nu^{r}_{\mathrm{PMS}}(\mathrm{P_S}[illegible])$$

$$\frac{d\mathrm{P_S}[illegible]}{dt} = \nu^{f}_{\mathrm{PMS}}(\mathrm{P_M}[illegible]) + \nu^{r}_{\mathrm{ASs}}(\tfrac{1}{4}((\mathrm{T_S}[illegible] + \ldots [illegible])\mathrm{W_S}\circ + (\ldots [illegible])\mathrm{W_S}\bullet)) - \nu^{f}_{\mathrm{ASs}}((\mathrm{D_S}[illegible] + \ldots)\mathrm{P_S}[illegible]) - \nu^{r}_{\mathrm{PMS}}(\mathrm{P_S}[illegible])$$

$$\frac{d\mathrm{P_S}[illegible]}{dt} = \nu^{f}_{\mathrm{PMS}}(\mathrm{P_M}[illegible]) + \nu^{r}_{\mathrm{ASs}}(\tfrac{1}{4}((\mathrm{T_S}[illegible] + \ldots [illegible])\mathrm{W_S}\circ + (\ldots [illegible])\mathrm{W_S}\bullet)) - \nu^{f}_{\mathrm{ASs}}((\mathrm{D_S}[illegible] + \ldots)\mathrm{P_S}[illegible]) - \nu^{r}_{\mathrm{PMS}}(\mathrm{P_S}[illegible])$$

$$\frac{d\mathrm{P_S}[illegible]}{dt} = \nu^{f}_{\mathrm{PMS}}(\mathrm{P_M}[illegible]) + \nu^{r}_{\mathrm{ASs}}(\tfrac{1}{6}((\mathrm{T_S}[illegible] + \ldots [illegible])\mathrm{W_S}\circ + (\ldots [illegible])\mathrm{W_S}\bullet)) - \nu^{f}_{\mathrm{ASs}}((\mathrm{D_S}[illegible] + \ldots)\mathrm{P_S}[illegible]) - \nu^{r}_{\mathrm{PMS}}(\mathrm{P_S}[illegible])$$

$$\frac{dP_{S}^{[illegible]}}{dt} = \nu^{f}_{PMS}(P_{M}^{[illegible]}) + \nu^{r}_{ASS}\Big(\tfrac{1}{6}\big((T_{S}^{[illegible]} + \text{[illegible]})W_{S}^{\circ} + (T_{S}^{[illegible]} + \text{[illegible]})W_{S}^{\bullet}\big)\Big) - \nu^{f}_{ASS}\big((D_{S}^{[illegible]} + \text{[illegible]})P_{S}^{[illegible]}\big) - \nu^{r}_{PMS}(P_{S}^{[illegible]})$$

$$\frac{dP_{S}^{[illegible]}}{dt} = \nu^{f}_{PMS}(P_{M}^{[illegible]}) + \nu^{r}_{ASS}\Big(\tfrac{1}{4}\big((T_{S}^{[illegible]} + \text{[illegible]})W_{S}^{\circ} + (T_{S}^{[illegible]} + \text{[illegible]})W_{S}^{\bullet}\big)\Big) - \nu^{f}_{ASS}\big((D_{S}^{[illegible]} + \text{[illegible]})P_{S}^{[illegible]}\big) - \nu^{r}_{PMS}(P_{S}^{[illegible]})$$

$$\frac{dP_{S}^{[illegible]}}{dt} = \nu^{f}_{PMS}(P_{M}^{[illegible]}) + \nu^{r}_{ASS}\Big(\tfrac{1}{6}\big((T_{S}^{[illegible]} + \text{[illegible]})W_{S}^{\circ} + (T_{S}^{[illegible]} + \text{[illegible]})W_{S}^{\bullet}\big)\Big) - \nu^{f}_{ASS}\big((D_{S}^{[illegible]} + \text{[illegible]})P_{S}^{[illegible]}\big) - \nu^{r}_{PMS}(P_{S}^{[illegible]})$$

$$\frac{dP_{S}^{[illegible]}}{dt} = \nu^{f}_{PMS}(P_{M}^{[illegible]}) + \nu^{r}_{ASS}\Big(\tfrac{1}{4}\big((T_{S}^{[illegible]} + \text{[illegible]})W_{S}^{\circ} + (T_{S}^{[illegible]} + \text{[illegible]})W_{S}^{\bullet}\big)\Big) - \nu^{f}_{ASS}\big((D_{S}^{[illegible]} + \text{[illegible]})P_{S}^{[illegible]}\big) - \nu^{r}_{PMS}(P_{S}^{[illegible]})$$

$$\frac{dP_{S}^{[illegible]}}{dt} = \nu^{f}_{PMS}(P_{M}^{[illegible]}) + \nu^{r}_{ASS}\Big(\tfrac{1}{4}\big((T_{S}^{[illegible]} + \text{[illegible]})W_{S}^{\circ} + (T_{S}^{[illegible]} + \text{[illegible]})W_{S}^{\bullet}\big)\Big) - \nu^{f}_{ASS}\big((D_{S}^{[illegible]} + \text{[illegible]})P_{S}^{[illegible]}\big) - \nu^{r}_{PMS}(P_{S}^{[illegible]})$$

$$\frac{dP_{S}^{[illegible]}}{dt} = \nu^{f}_{PMS}(P_{M}^{[illegible]}) + \nu^{r}_{ASS}\big((T_{S}^{[illegible]} + \text{[illegible]})W_{S}^{\bullet}\big) - \nu^{f}_{ASS}\big((D_{S}^{[illegible]} + \text{[illegible]})P_{S}^{[illegible]}\big) - \nu^{r}_{PMS}(P_{S}^{[illegible]})$$

$$\frac{dW_{E}^{\circ}}{dt} = \nu^{r}_{ASE}\Big((D_{E}^{[illegible]} + \text{[illegible]})\big(P_{E}^{[illegible]} + \tfrac{1}{2}(P_{E}^{[illegible]} + \text{[illegible]}) + \tfrac{1}{4}(P_{E}^{[illegible]} + \text{[illegible]}) + \tfrac{3}{4}(P_{E}^{[illegible]} + \text{[illegible]})\big)\Big) + \nu^{r}_{WEO}(W_{O}^{\circ}) - \nu^{f}_{ASE}\big((T_{E}^{[illegible]} + \text{[illegible]})W_{E}^{\circ}\big) - \nu^{f}_{WEO}(W_{E}^{\circ})$$

$$\frac{dW_{E}^{\bullet}}{dt} = \nu^{r}_{ASE}\Big((D_{E}^{[illegible]} + \text{[illegible]})\big(P_{E}^{[illegible]} + \tfrac{1}{2}(P_{E}^{[illegible]} + \text{[illegible]}) + \tfrac{1}{4}(P_{E}^{[illegible]} + \text{[illegible]}) + \tfrac{3}{4}(P_{E}^{[illegible]} + \text{[illegible]})\big)\Big) + \nu^{r}_{WEO}(W_{O}^{\bullet}) - \nu^{f}_{ASE}\big((T_{E}^{[illegible]} + \text{[illegible]})W_{E}^{\bullet}\big) - \nu^{f}_{WEO}(W_{E}^{\bullet})$$

$$\frac{dW_{S}^{\circ}}{dt} = \nu^{f}_{ASS}\Big((D_{S}^{[illegible]} + \text{[illegible]})\big(P_{S}^{[illegible]} + \tfrac{1}{2}(P_{S}^{[illegible]} + \text{[illegible]}) + \tfrac{1}{4}(P_{S}^{[illegible]} + \text{[illegible]}) + \tfrac{3}{4}(P_{S}^{[illegible]} + \text{[illegible]})\big)\Big) + \nu^{f}_{WOS}(W_{O}^{\circ}) - \nu^{r}_{ASS}\big((T_{S}^{[illegible]} + \text{[illegible]} +$$

$$
\mathrm{T_S}\substack{\bullet\bullet\\\bullet\circ\\\circ\circ}+\mathrm{T_S}\substack{\bullet\circ\\\bullet\bullet\\\circ\bullet}+\mathrm{T_S}\substack{\bullet\circ\\\bullet\bullet\\\circ\circ}+\mathrm{T_S}\substack{\bullet\circ\\\bullet\circ\\\circ\bullet}+\mathrm{T_S}\substack{\bullet\circ\\\bullet\circ\\\circ\circ}+\mathrm{T_S}\substack{\bullet\bullet\\\circ\bullet\\\bullet\bullet}+\mathrm{T_S}\substack{\bullet\bullet\\\circ\bullet\\\bullet\circ}+\mathrm{T_S}\substack{\bullet\bullet\\\circ\circ\\\bullet\bullet}+\mathrm{T_S}\substack{\bullet\bullet\\\circ\circ\\\bullet\circ}+\mathrm{T_S}\substack{\bullet\circ\\\circ\bullet\\\bullet\bullet}+\mathrm{T_S}\substack{\bullet\circ\\\circ\bullet\\\bullet\circ}+\mathrm{T_S}\substack{\bullet\circ\\\circ\circ\\\bullet\bullet}+\mathrm{T_S}\substack{\bullet\circ\\\circ\circ\\\bullet\circ}+\mathrm{T_S}\substack{\bullet\bullet\\\circ\bullet\\\circ\bullet}+\mathrm{T_S}\substack{\bullet\bullet\\\circ\bullet\\\circ\circ}+\mathrm{T_S}\substack{\bullet\bullet\\\circ\circ\\\circ\bullet}+\mathrm{T_S}\substack{\bullet\bullet\\\circ\circ\\\circ\circ}+\mathrm{T_S}\substack{\bullet\circ\\\circ\bullet\\\circ\bullet}+\mathrm{T_S}\substack{\bullet\circ\\\circ\bullet\\\circ\circ}+
\mathrm{T_S}\substack{\bullet\circ\\\circ\circ\\\circ\bullet}+\mathrm{T_S}\substack{\bullet\circ\\\circ\circ\\\circ\circ}+\mathrm{T_S}\substack{\circ\bullet\\\bullet\bullet\\\bullet\bullet}+\mathrm{T_S}\substack{\circ\bullet\\\bullet\bullet\\\bullet\circ}+\mathrm{T_S}\substack{\circ\bullet\\\bullet\circ\\\bullet\bullet}+\mathrm{T_S}\substack{\circ\bullet\\\bullet\circ\\\bullet\circ}+\mathrm{T_S}\substack{\circ\circ\\\bullet\bullet\\\bullet\bullet}+\mathrm{T_S}\substack{\circ\circ\\\bullet\bullet\\\bullet\circ}+\mathrm{T_S}\substack{\circ\circ\\\bullet\circ\\\bullet\bullet}+\mathrm{T_S}\substack{\circ\circ\\\bullet\circ\\\bullet\circ}+\mathrm{T_S}\substack{\circ\bullet\\\bullet\bullet\\\circ\bullet}+\mathrm{T_S}\substack{\circ\bullet\\\bullet\bullet\\\circ\circ}+\mathrm{T_S}\substack{\circ\bullet\\\bullet\circ\\\circ\bullet}+\mathrm{T_S}\substack{\circ\bullet\\\bullet\circ\\\circ\circ}+\mathrm{T_S}\substack{\circ\circ\\\bullet\bullet\\\circ\bullet}+\mathrm{T_S}\substack{\circ\circ\\\bullet\bullet\\\circ\circ}+\mathrm{T_S}\substack{\circ\circ\\\bullet\circ\\\circ\bullet}+\mathrm{T_S}\substack{\circ\circ\\\bullet\circ\\\circ\circ}+\mathrm{T_S}\substack{\circ\bullet\\\circ\bullet\\\bullet\bullet}+
\mathrm{T_S}\substack{\circ\bullet\\\circ\bullet\\\bullet\circ}+\mathrm{T_S}\substack{\circ\bullet\\\circ\circ\\\bullet\bullet}+\mathrm{T_S}\substack{\circ\bullet\\\circ\circ\\\bullet\circ}+\mathrm{T_S}\substack{\circ\circ\\\circ\bullet\\\bullet\bullet}+\mathrm{T_S}\substack{\circ\circ\\\circ\bullet\\\bullet\circ}+\mathrm{T_S}\substack{\circ\circ\\\circ\circ\\\bullet\bullet}+\mathrm{T_S}\substack{\circ\circ\\\circ\circ\\\bullet\circ}+\mathrm{T_S}\substack{\circ\bullet\\\circ\bullet\\\circ\bullet}+\mathrm{T_S}\substack{\circ\bullet\\\circ\bullet\\\circ\circ}+\mathrm{T_S}\substack{\circ\bullet\\\circ\circ\\\circ\bullet}+\mathrm{T_S}\substack{\circ\bullet\\\circ\circ\\\circ\circ}+\mathrm{T_S}\substack{\circ\circ\\\circ\bullet\\\circ\bullet}+\mathrm{T_S}\substack{\circ\circ\\\circ\bullet\\\circ\circ}+\mathrm{T_S}\substack{\circ\circ\\\circ\circ\\\circ\bullet}+\mathrm{T_S}\substack{\circ\circ\\\circ\circ\\\circ\circ}\Big)\mathrm{W_S}{\circ}\Big)-\underset{\mathrm{Wos}}{\nu_r}\big(\mathrm{W_S}{\circ}\big)
$$

$$
\frac{d\mathrm{W_S}{\bullet}}{dt}=\underset{\mathrm{ASs}}{\nu_f}\Big(\Big(\mathrm{D_S}\substack{\bullet\\\bullet\\\bullet}+\mathrm{D_S}\substack{\bullet\\\bullet\\\circ}+\mathrm{D_S}\substack{\bullet\\\circ\\\bullet}+\mathrm{D_S}\substack{\bullet\\\circ\\\circ}+\mathrm{D_S}\substack{\circ\\\bullet\\\bullet}+\mathrm{D_S}\substack{\circ\\\bullet\\\circ}+\mathrm{D_S}\substack{\circ\\\circ\\\bullet}+\mathrm{D_S}\substack{\circ\\\circ\\\circ}\Big)\Big(\mathrm{P_S}\substack{\bullet\\\bullet\\\bullet\\\bullet}+\frac{1}{2}\big(\mathrm{P_S}\substack{\bullet\\\bullet\\\circ\\\circ}+\mathrm{P_S}\substack{\bullet\\\circ\\\bullet\\\circ}+\mathrm{P_S}\substack{\bullet\\\circ\\\circ\\\bullet}+\mathrm{P_S}\substack{\circ\\\bullet\\\bullet\\\circ}+\mathrm{P_S}\substack{\circ\\\bullet\\\circ\\\bullet}+\mathrm{P_S}\substack{\circ\\\circ\\\bullet\\\bullet}\big)+\frac{1}{4}\big(\mathrm{P_S}\substack{\bullet\\\circ\\\circ\\\circ}+\mathrm{P_S}\substack{\circ\\\bullet\\\circ\\\circ}+\mathrm{P_S}\substack{\circ\\\circ\\\bullet\\\circ}+
\mathrm{P_S}\substack{\circ\\\circ\\\circ\\\bullet}\big)+\frac{3}{4}\big(\mathrm{P_S}\substack{\bullet\\\bullet\\\bullet\\\circ}+\mathrm{P_S}\substack{\bullet\\\bullet\\\circ\\\bullet}+\mathrm{P_S}\substack{\bullet\\\circ\\\bullet\\\bullet}+\mathrm{P_S}\substack{\circ\\\bullet\\\bullet\\\bullet}\big)\Big)\Big)+\underset{\mathrm{Wos}}{\nu_f}\big(\mathrm{W_O}{\bullet}\big)-\underset{\mathrm{ASs}}{\nu_r}\Big(\Big(\mathrm{T_S}\substack{\bullet\bullet\\\bullet\bullet\\\bullet\bullet}+\mathrm{T_S}\substack{\bullet\bullet\\\bullet\bullet\\\bullet\circ}+\mathrm{T_S}\substack{\bullet\bullet\\\bullet\circ\\\bullet\bullet}+\mathrm{T_S}\substack{\bullet\bullet\\\bullet\circ\\\bullet\circ}+\mathrm{T_S}\substack{\bullet\circ\\\bullet\bullet\\\bullet\bullet}+\mathrm{T_S}\substack{\bullet\circ\\\bullet\bullet\\\bullet\circ}+\mathrm{T_S}\substack{\bullet\circ\\\bullet\circ\\\bullet\bullet}+\mathrm{T_S}\substack{\bullet\circ\\\bullet\circ\\\bullet\circ}+\mathrm{T_S}\substack{\bullet\bullet\\\bullet\bullet\\\circ\bullet}+\mathrm{T_S}\substack{\bullet\bullet\\\bullet\bullet\\\circ\circ}+\mathrm{T_S}\substack{\bullet\bullet\\\bullet\circ\\\circ\bullet}+
\mathrm{T_S}\substack{\bullet\bullet\\\bullet\circ\\\circ\circ}+\mathrm{T_S}\substack{\bullet\circ\\\bullet\bullet\\\circ\bullet}+\mathrm{T_S}\substack{\bullet\circ\\\bullet\bullet\\\circ\circ}+\mathrm{T_S}\substack{\bullet\circ\\\bullet\circ\\\circ\bullet}+\mathrm{T_S}\substack{\bullet\circ\\\bullet\circ\\\circ\circ}+\mathrm{T_S}\substack{\bullet\bullet\\\circ\bullet\\\bullet\bullet}+\mathrm{T_S}\substack{\bullet\bullet\\\circ\bullet\\\bullet\circ}+\mathrm{T_S}\substack{\bullet\bullet\\\circ\circ\\\bullet\bullet}+\mathrm{T_S}\substack{\bullet\bullet\\\circ\circ\\\bullet\circ}+\mathrm{T_S}\substack{\bullet\circ\\\circ\bullet\\\bullet\bullet}+\mathrm{T_S}\substack{\bullet\circ\\\circ\bullet\\\bullet\circ}+\mathrm{T_S}\substack{\bullet\circ\\\circ\circ\\\bullet\bullet}+\mathrm{T_S}\substack{\bullet\circ\\\circ\circ\\\bullet\circ}+\mathrm{T_S}\substack{\bullet\bullet\\\circ\bullet\\\circ\bullet}+\mathrm{T_S}\substack{\bullet\bullet\\\circ\bullet\\\circ\circ}+\mathrm{T_S}\substack{\bullet\bullet\\\circ\circ\\\circ\bullet}+\mathrm{T_S}\substack{\bullet\bullet\\\circ\circ\\\circ\circ}+\mathrm{T_S}\substack{\bullet\circ\\\circ\bullet\\\circ\bullet}+\mathrm{T_S}\substack{\bullet\circ\\\circ\bullet\\\circ\circ}+
\mathrm{T_S}\substack{\bullet\circ\\\circ\circ\\\circ\bullet}+\mathrm{T_S}\substack{\bullet\circ\\\circ\circ\\\circ\circ}+\mathrm{T_S}\substack{\circ\bullet\\\bullet\bullet\\\bullet\bullet}+\mathrm{T_S}\substack{\circ\bullet\\\bullet\bullet\\\bullet\circ}+\mathrm{T_S}\substack{\circ\bullet\\\bullet\circ\\\bullet\bullet}+\mathrm{T_S}\substack{\circ\bullet\\\bullet\circ\\\bullet\circ}+\mathrm{T_S}\substack{\circ\circ\\\bullet\bullet\\\bullet\bullet}+\mathrm{T_S}\substack{\circ\circ\\\bullet\bullet\\\bullet\circ}+\mathrm{T_S}\substack{\circ\circ\\\bullet\circ\\\bullet\bullet}+\mathrm{T_S}\substack{\circ\circ\\\bullet\circ\\\bullet\circ}+\mathrm{T_S}\substack{\circ\bullet\\\bullet\bullet\\\circ\bullet}+\mathrm{T_S}\substack{\circ\bullet\\\bullet\bullet\\\circ\circ}+\mathrm{T_S}\substack{\circ\bullet\\\bullet\circ\\\circ\bullet}+\mathrm{T_S}\substack{\circ\bullet\\\bullet\circ\\\circ\circ}+\mathrm{T_S}\substack{\circ\circ\\\bullet\bullet\\\circ\bullet}+\mathrm{T_S}\substack{\circ\circ\\\bullet\bullet\\\circ\circ}+\mathrm{T_S}\substack{\circ\circ\\\bullet\circ\\\circ\bullet}+\mathrm{T_S}\substack{\circ\circ\\\bullet\circ\\\circ\circ}+\mathrm{T_S}\substack{\circ\bullet\\\circ\bullet\\\bullet\bullet}+
\mathrm{T_S}\substack{\circ\bullet\\\circ\bullet\\\bullet\circ}+\mathrm{T_S}\substack{\circ\bullet\\\circ\circ\\\bullet\bullet}+\mathrm{T_S}\substack{\circ\bullet\\\circ\circ\\\bullet\circ}+\mathrm{T_S}\substack{\circ\circ\\\circ\bullet\\\bullet\bullet}+\mathrm{T_S}\substack{\circ\circ\\\circ\bullet\\\bullet\circ}+\mathrm{T_S}\substack{\circ\circ\\\circ\circ\\\bullet\bullet}+\mathrm{T_S}\substack{\circ\circ\\\circ\circ\\\bullet\circ}+\mathrm{T_S}\substack{\circ\bullet\\\circ\bullet\\\circ\bullet}+\mathrm{T_S}\substack{\circ\bullet\\\circ\bullet\\\circ\circ}+\mathrm{T_S}\substack{\circ\bullet\\\circ\circ\\\circ\bullet}+\mathrm{T_S}\substack{\circ\bullet\\\circ\circ\\\circ\circ}+\mathrm{T_S}\substack{\circ\circ\\\circ\bullet\\\circ\bullet}+\mathrm{T_S}\substack{\circ\circ\\\circ\bullet\\\circ\circ}+\mathrm{T_S}\substack{\circ\circ\\\circ\circ\\\circ\bullet}+\mathrm{T_S}\substack{\circ\circ\\\circ\circ\\\circ\circ}\Big)\mathrm{W_S}{\bullet}\Big)-\underset{\mathrm{Wos}}{\nu_r}\big(\mathrm{W_S}{\bullet}\big)
$$

2.3 Pool definitions

$$\mathrm{D_E}^0=\mathrm{D_E}\substack{\circ\\\circ\\\circ}$$

$$\mathrm{D_E}^1=\mathrm{D_E}\substack{\bullet\\\circ\\\circ}+\mathrm{D_E}\substack{\circ\\\bullet\\\circ}+\mathrm{D_E}\substack{\circ\\\circ\\\bullet}$$

$$\mathrm{D_E}^2=\mathrm{D_E}\substack{\bullet\\\bullet\\\circ}+\mathrm{D_E}\substack{\bullet\\\circ\\\bullet}+\mathrm{D_E}\substack{\circ\\\bullet\\\bullet}$$

$$\mathrm{D_E}^3=\mathrm{D_E}\substack{\bullet\\\bullet\\\bullet}$$

$$\mathrm{D_I}^0=\mathrm{D_I}\substack{\circ\\\circ\\\circ}$$

$$\mathrm{D_I}^1=\mathrm{D_I}\substack{\bullet\\\circ\\\circ}+\mathrm{D_I}\substack{\circ\\\bullet\\\circ}+\mathrm{D_I}\substack{\circ\\\circ\\\bullet}$$

$$\mathrm{D_I}^2=\mathrm{D_I}\substack{\bullet\\\bullet\\\circ}+\mathrm{D_I}\substack{\bullet\\\circ\\\bullet}+\mathrm{D_I}\substack{\circ\\\bullet\\\bullet}$$

$$\mathrm{D_I}^3=\mathrm{D_I}\substack{\bullet\\\bullet\\\bullet}$$

$$\mathrm{D_M}^0=\mathrm{D_M}\substack{\circ\\\circ\\\circ}$$

$$\mathrm{D_M}^1=\mathrm{D_M}\substack{\bullet\\\circ\\\circ}+\mathrm{D_M}\substack{\circ\\\bullet\\\circ}+\mathrm{D_M}\substack{\circ\\\circ\\\bullet}$$

$$\mathrm{D_M}^2=\mathrm{D_M}\substack{\bullet\\\bullet\\\circ}+\mathrm{D_M}\substack{\bullet\\\circ\\\bullet}+\mathrm{D_M}\substack{\circ\\\bullet\\\bullet}$$

$$\mathrm{D_M}^3=\mathrm{D_M}\substack{\bullet\\\bullet\\\bullet}$$

$$\mathrm{D_O}^0=\mathrm{D_O}\substack{\circ\\\circ\\\circ}$$

$$\mathrm{D_O}^1=\mathrm{D_O}\substack{\bullet\\\circ\\\circ}+\mathrm{D_O}\substack{\circ\\\bullet\\\circ}+\mathrm{D_O}\substack{\circ\\\circ\\\bullet}$$

$$\mathrm{D_O}^2=\mathrm{D_O}\substack{\bullet\\\bullet\\\circ}+\mathrm{D_O}\substack{\bullet\\\circ\\\bullet}+\mathrm{D_O}\substack{\circ\\\bullet\\\bullet}$$

$$\mathrm{D_O}^3=\mathrm{D_O}\substack{\bullet\\\bullet\\\bullet}$$

$$\mathrm{D_S}^0=\mathrm{D_S}\substack{\circ\\\circ\\\circ}$$

$$\mathrm{D_S}^1=\mathrm{D_S}\substack{\bullet\\\circ\\\circ}+\mathrm{D_S}\substack{\circ\\\bullet\\\circ}+\mathrm{D_S}\substack{\circ\\\circ\\\bullet}$$

$$\mathrm{D_S}^2=\mathrm{D_S}\substack{\bullet\\\bullet\\\circ}+\mathrm{D_S}\substack{\bullet\\\circ\\\bullet}+\mathrm{D_S}\substack{\circ\\\bullet\\\bullet}$$

$$\mathrm{D_S}^3=\mathrm{D_S}\substack{\bullet\\\bullet\\\bullet}$$

$$\mathrm{T_E}^{00}=\mathrm{T_E}\substack{\circ\circ\\\circ\circ\\\circ\circ}$$

$$\mathrm{T_E}^{01}=\mathrm{T_E}\substack{\circ\bullet\\\circ\circ\\\circ\circ}+\mathrm{T_E}\substack{\circ\circ\\\circ\bullet\\\circ\circ}+\mathrm{T_E}\substack{\circ\circ\\\circ\circ\\\circ\bullet}$$

$$\mathrm{T_E}^{02}=\mathrm{T_E}\substack{\circ\bullet\\\circ\bullet\\\circ\circ}+\mathrm{T_E}\substack{\circ\bullet\\\circ\circ\\\circ\bullet}+\mathrm{T_E}\substack{\circ\circ\\\circ\bullet\\\circ\bullet}$$

$$\mathrm{T_E}^{03}=\mathrm{T_E}\substack{\circ\bullet\\\circ\bullet\\\circ\bullet}$$

$$\mathrm{T_E}^{10}=\mathrm{T_E}\substack{\bullet\circ\\\circ\circ\\\circ\circ}+\mathrm{T_E}\substack{\circ\circ\\\bullet\circ\\\circ\circ}+\mathrm{T_E}\substack{\circ\circ\\\circ\circ\\\bullet\circ}$$

$$\mathrm{T_E}^{11}=\mathrm{T_E}\substack{\bullet\bullet\\\circ\circ\\\circ\circ}+\mathrm{T_E}\substack{\bullet\circ\\\circ\bullet\\\circ\circ}+\mathrm{T_E}\substack{\bullet\circ\\\circ\circ\\\circ\bullet}+\mathrm{T_E}\substack{\circ\bullet\\\bullet\circ\\\circ\circ}+\mathrm{T_E}\substack{\circ\circ\\\bullet\bullet\\\circ\circ}+\mathrm{T_E}\substack{\circ\circ\\\bullet\circ\\\circ\bullet}+\mathrm{T_E}\substack{\circ\bullet\\\circ\circ\\\bullet\circ}+\mathrm{T_E}\substack{\circ\circ\\\circ\bullet\\\bullet\circ}+\mathrm{T_E}\substack{\circ\circ\\\circ\circ\\\bullet\bullet}$$

$$\mathrm{T_E}^{12}=\mathrm{T_E}\substack{\bullet\bullet\\\circ\bullet\\\circ\circ}+\mathrm{T_E}\substack{\bullet\bullet\\\circ\circ\\\circ\bullet}+\mathrm{T_E}\substack{\bullet\circ\\\circ\bullet\\\circ\bullet}+\mathrm{T_E}\substack{\circ\bullet\\\bullet\bullet\\\circ\circ}+\mathrm{T_E}\substack{\circ\bullet\\\bullet\circ\\\circ\bullet}+\mathrm{T_E}\substack{\circ\circ\\\bullet\bullet\\\circ\bullet}+\mathrm{T_E}\substack{\circ\bullet\\\circ\bullet\\\bullet\circ}+\mathrm{T_E}\substack{\circ\bullet\\\circ\circ\\\bullet\bullet}+\mathrm{T_E}\substack{\circ\circ\\\circ\bullet\\\bullet\bullet}$$

$T_E{}^{13}$ = T_E[●●/○●/○●] + T_E[○●/●●/○●] + T_E[○●/○●/●●]

$T_E{}^{20}$ = T_E[●○/●○/○○] + T_E[●○/○○/●○] + T_E[○○/●○/●○]

$T_E{}^{21}$ = T_E[●●/●○/○○] + T_E[●○/●●/○○] + T_E[●○/●○/○●] + T_E[●●/○○/●○] + T_E[●○/○●/●○] + T_E[●○/○○/●●] + T_E[○●/●○/●○] + T_E[○○/●●/●○] + T_E[○○/●○/●●]

$T_E{}^{22}$ = T_E[●●/●●/○○] + T_E[●●/●○/○●] + T_E[●○/●●/○●] + T_E[●●/○●/●○] + T_E[●●/○○/●●] + T_E[●○/○●/●●] + T_E[○●/●●/●○] + T_E[○●/●○/●●] + T_E[○○/●●/●●]

$T_E{}^{23}$ = T_E[●●/●●/○●] + T_E[●●/○●/●●] + T_E[○●/●●/●●]

$T_E{}^{30}$ = T_E[●○/●○/●○]

$T_E{}^{31}$ = T_E[●●/●○/●○] + T_E[●○/●●/●○] + T_E[●○/●○/●●]

$T_E{}^{32}$ = T_E[●●/●●/●○] + T_E[●●/●○/●●] + T_E[●○/●●/●●]

$T_E{}^{33}$ = T_E[●●/●●/●●]

$T_I{}^{00}$ = T_I[○○/○○/○○]

$T_I{}^{01}$ = T_I[○●/○○/○○] + T_I[○○/○●/○○] + T_I[○○/○○/○●]

$T_I{}^{02}$ = T_I[○●/○●/○○] + T_I[○●/○○/○●] + T_I[○○/○●/○●]

$T_I{}^{03}$ = T_I[○●/○●/○●]

$T_I{}^{10}$ = T_I[●○/○○/○○] + T_I[○○/●○/○○] + T_I[○○/○○/●○]

$T_I{}^{11}$ = T_I[●●/○○/○○] + T_I[●○/○●/○○] + T_I[●○/○○/○●] + T_I[○●/●○/○○] + T_I[○○/●●/○○] + T_I[○○/●○/○●] + T_I[○●/○○/●○] + T_I[○○/○●/●○] + T_I[○○/○○/●●]

$T_I{}^{12}$ = T_I[●●/○●/○○] + T_I[●●/○○/○●] + T_I[●○/○●/○●] + T_I[○●/●●/○○] + T_I[○●/●○/○●] + T_I[○○/●●/○●] + T_I[○●/○●/●○] + T_I[○●/○○/●●] + T_I[○○/○●/●●]

$T_I{}^{13}$ = T_I[●●/○●/○●] + T_I[○●/●●/○●] + T_I[○●/○●/●●]

$T_I{}^{20}$ = T_I[●○/●○/○○] + T_I[●○/○○/●○] + T_I[○○/●○/●○]

$T_I{}^{21}$ = T_I[●●/●○/○○] + T_I[●○/●●/○○] + T_I[●○/●○/○●] + T_I[●●/○○/●○] + T_I[●○/○●/●○] + T_I[●○/○○/●●] + T_I[○●/●○/●○] + T_I[○○/●●/●○] + T_I[○○/●○/●●]

$T_I{}^{22}$ = T_I[●●/●●/○○] + T_I[●●/●○/○●] + T_I[●○/●●/○●] + T_I[●●/○●/●○] + T_I[●●/○○/●●] + T_I[●○/○●/●●] + T_I[○●/●●/●○] + T_I[○●/●○/●●] + T_I[○○/●●/●●]

$T_I{}^{23}$ = T_I[●●/●●/○●] + T_I[●●/○●/●●] + T_I[○●/●●/●●]

$T_I{}^{30}$ = T_I[●○/●○/●○]

$T_I{}^{31}$ = T_I[●●/●○/●○] + T_I[●○/●●/●○] + T_I[●○/●○/●●]

$T_I{}^{32}$ = T_I[●●/●●/●○] + T_I[●●/●○/●●] + T_I[●○/●●/●●]

$T_I{}^{33}$ = T_I[●●/●●/●●]

$T_M{}^{00}$ = T_M[○○/○○/○○]

$T_M{}^{01}$ = T_M[○●/○○/○○] + T_M[○○/○●/○○] + T_M[○○/○○/○●]

$T_M{}^{02}$ = T_M[○●/○●/○○] + T_M[○●/○○/○●] + T_M[○○/○●/○●]

$T_M{}^{03}$ = T_M[○●/○●/○●]

$T_M{}^{10}$ = T_M[●○/○○/○○] + T_M[○○/●○/○○] + T_M[○○/○○/●○]

$T_M{}^{11}$ = T_M[●●/○○/○○] + T_M[●○/○●/○○] + T_M[●○/○○/○●] + T_M[○●/●○/○○] + T_M[○○/●●/○○] + T_M[○○/●○/○●] + T_M[○●/○○/●○] + T_M[○○/○●/●○] + T_M[○○/○○/●●]

$T_M{}^{12}$ = T_M[●●/○●/○○] + T_M[●●/○○/○●] + T_M[●○/○●/○●] + T_M[○●/●●/○○] + T_M[○●/●○/○●] + T_M[○○/●●/○●] + T_M[○●/○●/●○] + T_M[○●/○○/●●] + T_M[○○/○●/●●]

$T_M{}^{13}$ = T_M[●●/○●/○●] + T_M[○●/●●/○●] + T_M[○●/○●/●●]

$T_M{}^{20}$ = T_M[●○/●○/○○] + T_M[●○/○○/●○] + T_M[○○/●○/●○]

$T_M{}^{21}$ = T_M[●●/●○/○○] + T_M[●○/●●/○○] + T_M[●○/●○/○●] + T_M[●●/○○/●○] + T_M[●○/○●/●○] + T_M[●○/○○/●●] + T_M[○●/●○/●○] + T_M[○○/●●/●○] + T_M[○○/●○/●●]

$T_M{}^{22}$ = T_M[●●/●●/○○] + T_M[●●/●○/○●] + T_M[●○/●●/○●] + T_M[●●/○●/●○] + T_M[●●/○○/●●] + T_M[●○/○●/●●] + T_M[○●/●●/●○] + T_M[○●/●○/●●] + T_M[○○/●●/●●]

$T_M{}^{23}$ = T_M[●●/●●/○●] + T_M[●●/○●/●●] + T_M[○●/●●/●●]

$$\mathrm{T_M}^{30} = \mathrm{T_M}\substack{\bullet\circ\\\bullet\circ\\\bullet\circ}$$

$$\mathrm{T_M}^{31} = \mathrm{T_M}\substack{\bullet\bullet\\\bullet\circ\\\bullet\circ} + \mathrm{T_M}\substack{\bullet\circ\\\bullet\bullet\\\bullet\circ} + \mathrm{T_M}\substack{\bullet\circ\\\bullet\circ\\\bullet\bullet}$$

$$\mathrm{T_M}^{32} = \mathrm{T_M}\substack{\bullet\bullet\\\bullet\bullet\\\bullet\circ} + \mathrm{T_M}\substack{\bullet\bullet\\\bullet\circ\\\bullet\bullet} + \mathrm{T_M}\substack{\bullet\circ\\\bullet\bullet\\\bullet\bullet}$$

$$\mathrm{T_M}^{33} = \mathrm{T_M}\substack{\bullet\bullet\\\bullet\bullet\\\bullet\bullet}$$

$$\mathrm{T_O}^{00} = \mathrm{T_O}\substack{\circ\circ\\\circ\circ\\\circ\circ}$$

$$\mathrm{T_O}^{01} = \mathrm{T_O}\substack{\circ\bullet\\\circ\circ\\\circ\circ} + \mathrm{T_O}\substack{\circ\circ\\\circ\bullet\\\circ\circ} + \mathrm{T_O}\substack{\circ\circ\\\circ\circ\\\circ\bullet}$$

$$\mathrm{T_O}^{02} = \mathrm{T_O}\substack{\circ\bullet\\\circ\bullet\\\circ\circ} + \mathrm{T_O}\substack{\circ\bullet\\\circ\circ\\\circ\bullet} + \mathrm{T_O}\substack{\circ\circ\\\circ\bullet\\\circ\bullet}$$

$$\mathrm{T_O}^{03} = \mathrm{T_O}\substack{\circ\bullet\\\circ\bullet\\\circ\bullet}$$

$$\mathrm{T_O}^{10} = \mathrm{T_O}\substack{\bullet\circ\\\circ\circ\\\circ\circ} + \mathrm{T_O}\substack{\circ\circ\\\bullet\circ\\\circ\circ} + \mathrm{T_O}\substack{\circ\circ\\\circ\circ\\\bullet\circ}$$

$$\mathrm{T_O}^{11} = \mathrm{T_O}\substack{\bullet\bullet\\\circ\circ\\\circ\circ} + \mathrm{T_O}\substack{\bullet\circ\\\circ\bullet\\\circ\circ} + \mathrm{T_O}\substack{\bullet\circ\\\circ\circ\\\circ\bullet} + \mathrm{T_O}\substack{\circ\bullet\\\bullet\circ\\\circ\circ} + \mathrm{T_O}\substack{\circ\circ\\\bullet\bullet\\\circ\circ} + \mathrm{T_O}\substack{\circ\circ\\\bullet\circ\\\circ\bullet} + \mathrm{T_O}\substack{\circ\bullet\\\circ\circ\\\bullet\circ} + \mathrm{T_O}\substack{\circ\circ\\\circ\bullet\\\bullet\circ} + \mathrm{T_O}\substack{\circ\circ\\\circ\circ\\\bullet\bullet}$$

$$\mathrm{T_O}^{12} = \mathrm{T_O}\substack{\bullet\bullet\\\circ\bullet\\\circ\circ} + \mathrm{T_O}\substack{\bullet\bullet\\\circ\circ\\\circ\bullet} + \mathrm{T_O}\substack{\bullet\circ\\\circ\bullet\\\circ\bullet} + \mathrm{T_O}\substack{\circ\bullet\\\bullet\bullet\\\circ\circ} + \mathrm{T_O}\substack{\circ\bullet\\\bullet\circ\\\circ\bullet} + \mathrm{T_O}\substack{\circ\circ\\\bullet\bullet\\\circ\bullet} + \mathrm{T_O}\substack{\circ\bullet\\\circ\bullet\\\bullet\circ} + \mathrm{T_O}\substack{\circ\bullet\\\circ\circ\\\bullet\bullet} + \mathrm{T_O}\substack{\circ\circ\\\circ\bullet\\\bullet\bullet}$$

$$\mathrm{T_O}^{13} = \mathrm{T_O}\substack{\bullet\bullet\\\circ\bullet\\\circ\bullet} + \mathrm{T_O}\substack{\circ\bullet\\\bullet\bullet\\\circ\bullet} + \mathrm{T_O}\substack{\circ\bullet\\\circ\bullet\\\bullet\bullet}$$

$$\mathrm{T_O}^{20} = \mathrm{T_O}\substack{\bullet\circ\\\bullet\circ\\\circ\circ} + \mathrm{T_O}\substack{\bullet\circ\\\circ\circ\\\bullet\circ} + \mathrm{T_O}\substack{\circ\circ\\\bullet\circ\\\bullet\circ}$$

$$\mathrm{T_O}^{21} = \mathrm{T_O}\substack{\bullet\bullet\\\bullet\circ\\\circ\circ} + \mathrm{T_O}\substack{\bullet\circ\\\bullet\bullet\\\circ\circ} + \mathrm{T_O}\substack{\bullet\circ\\\bullet\circ\\\circ\bullet} + \mathrm{T_O}\substack{\bullet\bullet\\\circ\circ\\\bullet\circ} + \mathrm{T_O}\substack{\bullet\circ\\\circ\bullet\\\bullet\circ} + \mathrm{T_O}\substack{\bullet\circ\\\circ\circ\\\bullet\bullet} + \mathrm{T_O}\substack{\circ\bullet\\\bullet\circ\\\bullet\circ} + \mathrm{T_O}\substack{\circ\circ\\\bullet\bullet\\\bullet\circ} + \mathrm{T_O}\substack{\circ\circ\\\bullet\circ\\\bullet\bullet}$$

$$\mathrm{T_O}^{22} = \mathrm{T_O}\substack{\bullet\bullet\\\bullet\bullet\\\circ\circ} + \mathrm{T_O}\substack{\bullet\bullet\\\bullet\circ\\\circ\bullet} + \mathrm{T_O}\substack{\bullet\circ\\\bullet\bullet\\\circ\bullet} + \mathrm{T_O}\substack{\bullet\bullet\\\circ\bullet\\\bullet\circ} + \mathrm{T_O}\substack{\bullet\bullet\\\circ\circ\\\bullet\bullet} + \mathrm{T_O}\substack{\bullet\circ\\\circ\bullet\\\bullet\bullet} + \mathrm{T_O}\substack{\circ\bullet\\\bullet\bullet\\\bullet\circ} + \mathrm{T_O}\substack{\circ\bullet\\\bullet\circ\\\bullet\bullet} + \mathrm{T_O}\substack{\circ\circ\\\bullet\bullet\\\bullet\bullet}$$

$$\mathrm{T_O}^{23} = \mathrm{T_O}\substack{\bullet\bullet\\\bullet\bullet\\\circ\bullet} + \mathrm{T_O}\substack{\bullet\bullet\\\circ\bullet\\\bullet\bullet} + \mathrm{T_O}\substack{\circ\bullet\\\bullet\bullet\\\bullet\bullet}$$

$$\mathrm{T_O}^{30} = \mathrm{T_O}\substack{\bullet\circ\\\bullet\circ\\\bullet\circ}$$

$$\mathrm{T_O}^{31} = \mathrm{T_O}\substack{\bullet\bullet\\\bullet\circ\\\bullet\circ} + \mathrm{T_O}\substack{\bullet\circ\\\bullet\bullet\\\bullet\circ} + \mathrm{T_O}\substack{\bullet\circ\\\bullet\circ\\\bullet\bullet}$$

$$\mathrm{T_O}^{32} = \mathrm{T_O}\substack{\bullet\bullet\\\bullet\bullet\\\bullet\circ} + \mathrm{T_O}\substack{\bullet\bullet\\\bullet\circ\\\bullet\bullet} + \mathrm{T_O}\substack{\bullet\circ\\\bullet\bullet\\\bullet\bullet}$$

$$\mathrm{T_O}^{33} = \mathrm{T_O}\substack{\bullet\bullet\\\bullet\bullet\\\bullet\bullet}$$

$$\mathrm{T_S}^{00} = \mathrm{T_S}\substack{\circ\circ\\\circ\circ\\\circ\circ}$$

$$\mathrm{T_S}^{01} = \mathrm{T_S}\substack{\circ\bullet\\\circ\circ\\\circ\circ} + \mathrm{T_S}\substack{\circ\circ\\\circ\bullet\\\circ\circ} + \mathrm{T_S}\substack{\circ\circ\\\circ\circ\\\circ\bullet}$$

$$\mathrm{T_S}^{02} = \mathrm{T_S}\substack{\circ\bullet\\\circ\bullet\\\circ\circ} + \mathrm{T_S}\substack{\circ\bullet\\\circ\circ\\\circ\bullet} + \mathrm{T_S}\substack{\circ\circ\\\circ\bullet\\\circ\bullet}$$

$$\mathrm{T_S}^{03} = \mathrm{T_S}\substack{\circ\bullet\\\circ\bullet\\\circ\bullet}$$

$$\mathrm{T_S}^{10} = \mathrm{T_S}\substack{\bullet\circ\\\circ\circ\\\circ\circ} + \mathrm{T_S}\substack{\circ\circ\\\bullet\circ\\\circ\circ} + \mathrm{T_S}\substack{\circ\circ\\\circ\circ\\\bullet\circ}$$

$$\mathrm{T_S}^{11} = \mathrm{T_S}\substack{\bullet\bullet\\\circ\circ\\\circ\circ} + \mathrm{T_S}\substack{\bullet\circ\\\circ\bullet\\\circ\circ} + \mathrm{T_S}\substack{\bullet\circ\\\circ\circ\\\circ\bullet} + \mathrm{T_S}\substack{\circ\bullet\\\bullet\circ\\\circ\circ} + \mathrm{T_S}\substack{\circ\circ\\\bullet\bullet\\\circ\circ} + \mathrm{T_S}\substack{\circ\circ\\\bullet\circ\\\circ\bullet} + \mathrm{T_S}\substack{\circ\bullet\\\circ\circ\\\bullet\circ} + \mathrm{T_S}\substack{\circ\circ\\\circ\bullet\\\bullet\circ} + \mathrm{T_S}\substack{\circ\circ\\\circ\circ\\\bullet\bullet}$$

$$\mathrm{T_S}^{12} = \mathrm{T_S}\substack{\bullet\bullet\\\circ\bullet\\\circ\circ} + \mathrm{T_S}\substack{\bullet\bullet\\\circ\circ\\\circ\bullet} + \mathrm{T_S}\substack{\bullet\circ\\\circ\bullet\\\circ\bullet} + \mathrm{T_S}\substack{\circ\bullet\\\bullet\bullet\\\circ\circ} + \mathrm{T_S}\substack{\circ\bullet\\\bullet\circ\\\circ\bullet} + \mathrm{T_S}\substack{\circ\circ\\\bullet\bullet\\\circ\bullet} + \mathrm{T_S}\substack{\circ\bullet\\\circ\bullet\\\bullet\circ} + \mathrm{T_S}\substack{\circ\bullet\\\circ\circ\\\bullet\bullet} + \mathrm{T_S}\substack{\circ\circ\\\circ\bullet\\\bullet\bullet}$$

$$\mathrm{T_S}^{13} = \mathrm{T_S}\substack{\bullet\bullet\\\circ\bullet\\\circ\bullet} + \mathrm{T_S}\substack{\circ\bullet\\\bullet\bullet\\\circ\bullet} + \mathrm{T_S}\substack{\circ\bullet\\\circ\bullet\\\bullet\bullet}$$

$$\mathrm{T_S}^{20} = \mathrm{T_S}\substack{\bullet\circ\\\bullet\circ\\\circ\circ} + \mathrm{T_S}\substack{\bullet\circ\\\circ\circ\\\bullet\circ} + \mathrm{T_S}\substack{\circ\circ\\\bullet\circ\\\bullet\circ}$$

$$\mathrm{T_S}^{21} = \mathrm{T_S}\substack{\bullet\bullet\\\bullet\circ\\\circ\circ} + \mathrm{T_S}\substack{\bullet\circ\\\bullet\bullet\\\circ\circ} + \mathrm{T_S}\substack{\bullet\circ\\\bullet\circ\\\circ\bullet} + \mathrm{T_S}\substack{\bullet\bullet\\\circ\circ\\\bullet\circ} + \mathrm{T_S}\substack{\bullet\circ\\\circ\bullet\\\bullet\circ} + \mathrm{T_S}\substack{\bullet\circ\\\circ\circ\\\bullet\bullet} + \mathrm{T_S}\substack{\circ\bullet\\\bullet\circ\\\bullet\circ} + \mathrm{T_S}\substack{\circ\circ\\\bullet\bullet\\\bullet\circ} + \mathrm{T_S}\substack{\circ\circ\\\bullet\circ\\\bullet\bullet}$$

$$\mathrm{T_S}^{22} = \mathrm{T_S}\substack{\bullet\bullet\\\bullet\bullet\\\circ\circ} + \mathrm{T_S}\substack{\bullet\bullet\\\bullet\circ\\\circ\bullet} + \mathrm{T_S}\substack{\bullet\circ\\\bullet\bullet\\\circ\bullet} + \mathrm{T_S}\substack{\bullet\bullet\\\circ\bullet\\\bullet\circ} + \mathrm{T_S}\substack{\bullet\bullet\\\circ\circ\\\bullet\bullet} + \mathrm{T_S}\substack{\bullet\circ\\\circ\bullet\\\bullet\bullet} + \mathrm{T_S}\substack{\circ\bullet\\\bullet\bullet\\\bullet\circ} + \mathrm{T_S}\substack{\circ\bullet\\\bullet\circ\\\bullet\bullet} + \mathrm{T_S}\substack{\circ\circ\\\bullet\bullet\\\bullet\bullet}$$

$$\mathrm{T_S}^{23} = \mathrm{T_S}\substack{\bullet\bullet\\\bullet\bullet\\\circ\bullet} + \mathrm{T_S}\substack{\bullet\bullet\\\circ\bullet\\\bullet\bullet} + \mathrm{T_S}\substack{\circ\bullet\\\bullet\bullet\\\bullet\bullet}$$

$$\mathrm{T_S}^{30} = \mathrm{T_S}\substack{\bullet\circ\\\bullet\circ\\\bullet\circ}$$

$$\mathrm{T_S}^{31} = \mathrm{T_S}\substack{\bullet\bullet\\\bullet\circ\\\bullet\circ} + \mathrm{T_S}\substack{\bullet\circ\\\bullet\bullet\\\bullet\circ} + \mathrm{T_S}\substack{\bullet\circ\\\bullet\circ\\\bullet\bullet}$$

$$\mathrm{T_S}^{32} = \mathrm{T_S}\substack{\bullet\bullet\\\bullet\bullet\\\bullet\circ} + \mathrm{T_S}\substack{\bullet\bullet\\\bullet\circ\\\bullet\bullet} + \mathrm{T_S}\substack{\bullet\circ\\\bullet\bullet\\\bullet\bullet}$$

$$\mathrm{T_S}^{33} = \mathrm{T_S}\substack{\bullet\bullet\\\bullet\bullet\\\bullet\bullet}$$

$$\mathrm{C_I}^{0} = \mathrm{C_I}\substack{\circ\\\circ\\\circ}$$

$$\mathrm{C_I}^1 = \mathrm{C_I}\substack{\bullet\\ \circ\\ \circ} + \mathrm{C_I}\substack{\circ\\ \bullet\\ \circ} + \mathrm{C_I}\substack{\circ\\ \circ\\ \bullet}$$

$$\mathrm{C_I}^2 = \mathrm{C_I}\substack{\bullet\\ \bullet\\ \circ} + \mathrm{C_I}\substack{\bullet\\ \circ\\ \bullet} + \mathrm{C_I}\substack{\circ\\ \bullet\\ \bullet}$$

$$\mathrm{C_I}^3 = \mathrm{C_I}\substack{\bullet\\ \bullet\\ \bullet}$$

$$\mathrm{C_O}^0 = \mathrm{C_O}\substack{\circ\\ \circ\\ \circ}$$

$$\mathrm{C_O}^1 = \mathrm{C_O}\substack{\bullet\\ \circ\\ \circ} + \mathrm{C_O}\substack{\circ\\ \bullet\\ \circ} + \mathrm{C_O}\substack{\circ\\ \circ\\ \bullet}$$

$$\mathrm{C_O}^2 = \mathrm{C_O}\substack{\bullet\\ \bullet\\ \circ} + \mathrm{C_O}\substack{\bullet\\ \circ\\ \bullet} + \mathrm{C_O}\substack{\circ\\ \bullet\\ \bullet}$$

$$\mathrm{C_O}^3 = \mathrm{C_O}\substack{\bullet\\ \bullet\\ \bullet}$$

$$\mathrm{P_E}^0 = \mathrm{P_E}\substack{\circ\\ \circ\\ \circ\\ \circ}$$

$$\mathrm{P_E}^1 = \mathrm{P_E}\substack{\bullet\\ \circ\\ \circ\\ \circ} + \mathrm{P_E}\substack{\circ\\ \bullet\\ \circ\\ \circ} + \mathrm{P_E}\substack{\circ\\ \circ\\ \bullet\\ \circ} + \mathrm{P_E}\substack{\circ\\ \circ\\ \circ\\ \bullet}$$

$$\mathrm{P_E}^2 = \mathrm{P_E}\substack{\bullet\\ \bullet\\ \circ\\ \circ} + \mathrm{P_E}\substack{\bullet\\ \circ\\ \bullet\\ \circ} + \mathrm{P_E}\substack{\bullet\\ \circ\\ \circ\\ \bullet} + \mathrm{P_E}\substack{\circ\\ \bullet\\ \bullet\\ \circ} + \mathrm{P_E}\substack{\circ\\ \bullet\\ \circ\\ \bullet} + \mathrm{P_E}\substack{\circ\\ \circ\\ \bullet\\ \bullet}$$

$$\mathrm{P_E}^3 = \mathrm{P_E}\substack{\bullet\\ \bullet\\ \bullet\\ \circ} + \mathrm{P_E}\substack{\bullet\\ \bullet\\ \circ\\ \bullet} + \mathrm{P_E}\substack{\bullet\\ \circ\\ \bullet\\ \bullet} + \mathrm{P_E}\substack{\circ\\ \bullet\\ \bullet\\ \bullet}$$

$$\mathrm{P_E}^4 = \mathrm{P_E}\substack{\bullet\\ \bullet\\ \bullet\\ \bullet}$$

$$\mathrm{P_M}^0 = \mathrm{P_M}\substack{\circ\\ \circ\\ \circ\\ \circ}$$

$$\mathrm{P_M}^1 = \mathrm{P_M}\substack{\bullet\\ \circ\\ \circ\\ \circ} + \mathrm{P_M}\substack{\circ\\ \bullet\\ \circ\\ \circ} + \mathrm{P_M}\substack{\circ\\ \circ\\ \bullet\\ \circ} + \mathrm{P_M}\substack{\circ\\ \circ\\ \circ\\ \bullet}$$

$$\mathrm{P_M}^2 = \mathrm{P_M}\substack{\bullet\\ \bullet\\ \circ\\ \circ} + \mathrm{P_M}\substack{\bullet\\ \circ\\ \bullet\\ \circ} + \mathrm{P_M}\substack{\bullet\\ \circ\\ \circ\\ \bullet} + \mathrm{P_M}\substack{\circ\\ \bullet\\ \bullet\\ \circ} + \mathrm{P_M}\substack{\circ\\ \bullet\\ \circ\\ \bullet} + \mathrm{P_M}\substack{\circ\\ \circ\\ \bullet\\ \bullet}$$

$$\mathrm{P_M}^3 = \mathrm{P_M}\substack{\bullet\\ \bullet\\ \bullet\\ \circ} + \mathrm{P_M}\substack{\bullet\\ \bullet\\ \circ\\ \bullet} + \mathrm{P_M}\substack{\bullet\\ \circ\\ \bullet\\ \bullet} + \mathrm{P_M}\substack{\circ\\ \bullet\\ \bullet\\ \bullet}$$

$$\mathrm{P_M}^4 = \mathrm{P_M}\substack{\bullet\\ \bullet\\ \bullet\\ \bullet}$$

$$\mathrm{P_O}^0 = \mathrm{P_O}\substack{\circ\\ \circ\\ \circ\\ \circ}$$

$$\mathrm{P_O}^1 = \mathrm{P_O}\substack{\bullet\\ \circ\\ \circ\\ \circ} + \mathrm{P_O}\substack{\circ\\ \bullet\\ \circ\\ \circ} + \mathrm{P_O}\substack{\circ\\ \circ\\ \bullet\\ \circ} + \mathrm{P_O}\substack{\circ\\ \circ\\ \circ\\ \bullet}$$

$$\mathrm{P_O}^2 = \mathrm{P_O}\substack{\bullet\\ \bullet\\ \circ\\ \circ} + \mathrm{P_O}\substack{\bullet\\ \circ\\ \bullet\\ \circ} + \mathrm{P_O}\substack{\bullet\\ \circ\\ \circ\\ \bullet} + \mathrm{P_O}\substack{\circ\\ \bullet\\ \bullet\\ \circ} + \mathrm{P_O}\substack{\circ\\ \bullet\\ \circ\\ \bullet} + \mathrm{P_O}\substack{\circ\\ \circ\\ \bullet\\ \bullet}$$

$$\mathrm{P_O}^3 = \mathrm{P_O}\substack{\bullet\\ \bullet\\ \bullet\\ \circ} + \mathrm{P_O}\substack{\bullet\\ \bullet\\ \circ\\ \bullet} + \mathrm{P_O}\substack{\bullet\\ \circ\\ \bullet\\ \bullet} + \mathrm{P_O}\substack{\circ\\ \bullet\\ \bullet\\ \bullet}$$

$$\mathrm{P_O}^4 = \mathrm{P_O}\substack{\bullet\\ \bullet\\ \bullet\\ \bullet}$$

$$\mathrm{P_S}^0 = \mathrm{P_S}\substack{\circ\\ \circ\\ \circ\\ \circ}$$

$$\mathrm{P_S}^1 = \mathrm{P_S}\substack{\bullet\\ \circ\\ \circ\\ \circ} + \mathrm{P_S}\substack{\circ\\ \bullet\\ \circ\\ \circ} + \mathrm{P_S}\substack{\circ\\ \circ\\ \bullet\\ \circ} + \mathrm{P_S}\substack{\circ\\ \circ\\ \circ\\ \bullet}$$

$$\mathrm{P_S}^2 = \mathrm{P_S}\substack{\bullet\\ \bullet\\ \circ\\ \circ} + \mathrm{P_S}\substack{\bullet\\ \circ\\ \bullet\\ \circ} + \mathrm{P_S}\substack{\bullet\\ \circ\\ \circ\\ \bullet} + \mathrm{P_S}\substack{\circ\\ \bullet\\ \bullet\\ \circ} + \mathrm{P_S}\substack{\circ\\ \bullet\\ \circ\\ \bullet} + \mathrm{P_S}\substack{\circ\\ \circ\\ \bullet\\ \bullet}$$

$$\mathrm{P_S}^3 = \mathrm{P_S}\substack{\bullet\\ \bullet\\ \bullet\\ \circ} + \mathrm{P_S}\substack{\bullet\\ \bullet\\ \circ\\ \bullet} + \mathrm{P_S}\substack{\bullet\\ \circ\\ \bullet\\ \bullet} + \mathrm{P_S}\substack{\circ\\ \bullet\\ \bullet\\ \bullet}$$

$$\mathrm{P_S}^4 = \mathrm{P_S}\substack{\bullet\\ \bullet\\ \bullet\\ \bullet}$$

$$\mathrm{W_E}^0 = \mathrm{W_E}\circ$$

$$\mathrm{W_E}^1 = \mathrm{W_E}\bullet$$

$$\mathrm{W_O}^0 = \mathrm{W_O}\circ$$

$$\mathrm{W_O}^1 = \mathrm{W_O}\bullet$$

$$\mathrm{W_S}^0 = \mathrm{W_S}\circ$$

$$\mathrm{W_S}^1 = \mathrm{W_S}\bullet$$

2.4 Kinetic equations for mass isotopomers

$$\frac{d\mathrm{D_E}^0}{dt} = \underset{\text{ASE}}{\nu_f}\big((\mathrm{T_E}^{00}+\mathrm{T_E}^{01}+\mathrm{T_E}^{02}+\mathrm{T_E}^{03})(\mathrm{W_E}^0+\mathrm{W_E}^1)\big) + \underset{\text{ADPEO}}{\nu_r}(\mathrm{D_O}^0) - \underset{\text{ADPEO}}{\nu_f}(\mathrm{D_E}^0) - \underset{\text{ASE}}{\nu_r}\big((\mathrm{P_E}^0+\mathrm{P_E}^1+\mathrm{P_E}^2+\mathrm{P_E}^3+\mathrm{P_E}^4)\mathrm{D_E}^0\big)$$

$$\frac{d\mathrm{D_E}^1}{dt} = \underset{\text{ASE}}{\nu_f}\big((\mathrm{T_E}^{10}+\mathrm{T_E}^{11}+\mathrm{T_E}^{12}+\mathrm{T_E}^{13})(\mathrm{W_E}^0+\mathrm{W_E}^1)\big) + \underset{\text{ADPEO}}{\nu_r}(\mathrm{D_O}^1) - \underset{\text{ADPEO}}{\nu_f}(\mathrm{D_E}^1) - \underset{\text{ASE}}{\nu_r}\big((\mathrm{P_E}^0+\mathrm{P_E}^1+\mathrm{P_E}^2+\mathrm{P_E}^3+\mathrm{P_E}^4)\mathrm{D_E}^1\big)$$

$$\frac{d\mathrm{D_E}^2}{dt} = \underset{\text{ASE}}{\nu_f}\big((\mathrm{T_E}^{20}+\mathrm{T_E}^{21}+\mathrm{T_E}^{22}+\mathrm{T_E}^{23})(\mathrm{W_E}^0+\mathrm{W_E}^1)\big) + \underset{\text{ADPEO}}{\nu_r}(\mathrm{D_O}^2) - \underset{\text{ADPEO}}{\nu_f}(\mathrm{D_E}^2) - \underset{\text{ASE}}{\nu_r}\big((\mathrm{P_E}^0+\mathrm{P_E}^1+\mathrm{P_E}^2+\mathrm{P_E}^3+\mathrm{P_E}^4)\mathrm{D_E}^2\big)$$

$$\frac{d\mathrm{D_E}^3}{dt} = \underset{\text{ASE}}{\nu_f}\big((\mathrm{T_E}^{30}+\mathrm{T_E}^{31}+\mathrm{T_E}^{32}+\mathrm{T_E}^{33})(\mathrm{W_E}^0+\mathrm{W_E}^1)\big) + \underset{\text{ADPEO}}{\nu_r}(\mathrm{D_O}^3) - \underset{\text{ADPEO}}{\nu_f}(\mathrm{D_E}^3) - \underset{\text{ASE}}{\nu_r}\big((\mathrm{P_E}^0+\mathrm{P_E}^1+\mathrm{P_E}^2+\mathrm{P_E}^3+\mathrm{P_E}^4)\mathrm{D_E}^3\big)$$

$$\frac{d\mathrm{D_I}^0}{dt} = \underset{\text{ADPOI}}{\nu_f}(\mathrm{D_O}^0) + \underset{\text{AKI}}{\nu_f}(2\mathrm{T_I}^{00}+\mathrm{T_I}^{01}+\mathrm{T_I}^{02}+\mathrm{T_I}^{03}+\mathrm{T_I}^{10}+\mathrm{T_I}^{20}+\mathrm{T_I}^{30}) + \underset{\text{CKI}}{\nu_f}(\mathrm{T_I}^{00}+\mathrm{T_I}^{01}+\mathrm{T_I}^{02}+\mathrm{T_I}^{03}) + \underset{\text{ADPIM}}{\nu_r}(\mathrm{D_M}^0) - \underset{\text{ADPIM}}{\nu_f}(\mathrm{D_I}^0) - \underset{\text{ADPOI}}{\nu_r}(\mathrm{D_I}^0) - \underset{\text{AKI}}{\nu_r}\big(2(\mathrm{D_I}^0+\mathrm{D_I}^1+\mathrm{D_I}^2+\mathrm{D_I}^3)\mathrm{D_I}^0\big) - \underset{\text{CKI}}{\nu_r}\big((\mathrm{C_I}^0+\mathrm{C_I}^1+\mathrm{C_I}^2+\mathrm{C_I}^3)\mathrm{D_I}^0\big)$$

$$\frac{d\mathrm{D_I}^1}{dt} = \underset{\text{ADPOI}}{\nu_f}(\mathrm{D_O}^1) + \underset{\text{AKI}}{\nu_f}(2\mathrm{T_I}^{11}+\mathrm{T_I}^{01}+\mathrm{T_I}^{10}+\mathrm{T_I}^{12}+\mathrm{T_I}^{13}+\mathrm{T_I}^{21}+\mathrm{T_I}^{31}) + \underset{\text{CKI}}{\nu_f}(\mathrm{T_I}^{10}+\mathrm{T_I}^{11}+\mathrm{T_I}^{12}+\mathrm{T_I}^{13}) + \underset{\text{ADPIM}}{\nu_r}(\mathrm{D_M}^1) - \underset{\text{ADPIM}}{\nu_f}(\mathrm{D_I}^1) - \underset{\text{ADPOI}}{\nu_r}(\mathrm{D_I}^1) - \underset{\text{AKI}}{\nu_r}\big(2(\mathrm{D_I}^0+\mathrm{D_I}^1+\mathrm{D_I}^2+\mathrm{D_I}^3)\mathrm{D_I}^1\big) - \underset{\text{CKI}}{\nu_r}\big((\mathrm{C_I}^0+\mathrm{C_I}^1+\mathrm{C_I}^2+\mathrm{C_I}^3)\mathrm{D_I}^1\big)$$

$$\frac{d\mathrm{D_I}^2}{dt} = \underset{\text{ADPOI}}{\nu_f}(\mathrm{D_O}^2) + \underset{\text{AKI}}{\nu_f}(2\mathrm{T_I}^{22}+\mathrm{T_I}^{02}+\mathrm{T_I}^{12}+\mathrm{T_I}^{20}+\mathrm{T_I}^{21}+\mathrm{T_I}^{23}+\mathrm{T_I}^{32}) + \underset{\text{CKI}}{\nu_f}(\mathrm{T_I}^{20}+\mathrm{T_I}^{21}+\mathrm{T_I}^{22}+\mathrm{T_I}^{23}) + \underset{\text{ADPIM}}{\nu_r}(\mathrm{D_M}^2) - \underset{\text{ADPIM}}{\nu_f}(\mathrm{D_I}^2) - \underset{\text{ADPOI}}{\nu_r}(\mathrm{D_I}^2) - \underset{\text{AKI}}{\nu_r}\big(2(\mathrm{D_I}^0+\mathrm{D_I}^1+\mathrm{D_I}^2+\mathrm{D_I}^3)\mathrm{D_I}^2\big) - \underset{\text{CKI}}{\nu_r}\big((\mathrm{C_I}^0+\mathrm{C_I}^1+\mathrm{C_I}^2+\mathrm{C_I}^3)\mathrm{D_I}^2\big)$$

$$\frac{d\mathrm{D_I}^3}{dt} = \underset{\text{ADPOI}}{\nu_f}(\mathrm{D_O}^3) + \underset{\text{AKI}}{\nu_f}(2\mathrm{T_I}^{33}+\mathrm{T_I}^{03}+\mathrm{T_I}^{13}+\mathrm{T_I}^{23}+\mathrm{T_I}^{30}+\mathrm{T_I}^{31}+\mathrm{T_I}^{32}) + \underset{\text{CKI}}{\nu_f}(\mathrm{T_I}^{30}+\mathrm{T_I}^{31}+\mathrm{T_I}^{32}+\mathrm{T_I}^{33}) + \underset{\text{ADPIM}}{\nu_r}(\mathrm{D_M}^3) - \underset{\text{ADPIM}}{\nu_f}(\mathrm{D_I}^3) - \underset{\text{ADPOI}}{\nu_r}(\mathrm{D_I}^3) - \underset{\text{AKI}}{\nu_r}\big(2(\mathrm{D_I}^0+\mathrm{D_I}^1+\mathrm{D_I}^2+\mathrm{D_I}^3)\mathrm{D_I}^3\big) - \underset{\text{CKI}}{\nu_r}\big((\mathrm{C_I}^0+\mathrm{C_I}^1+\mathrm{C_I}^2+\mathrm{C_I}^3)\mathrm{D_I}^3\big)$$

$$\frac{d\mathrm{D_M}^0}{dt} = \underset{\text{ADPIM}}{\nu_f}(\mathrm{D_I}^0) + \underset{\text{ADPMS}}{\nu_r}(\mathrm{D_S}^0) - \underset{\text{ADPMS}}{\nu_f}(\mathrm{D_M}^0) - \underset{\text{ADPIM}}{\nu_r}(\mathrm{D_M}^0)$$

$$\frac{d\mathrm{D_M}^1}{dt} = \underset{\text{ADPIM}}{\nu_f}(\mathrm{D_I}^1) + \underset{\text{ADPMS}}{\nu_r}(\mathrm{D_S}^1) - \underset{\text{ADPMS}}{\nu_f}(\mathrm{D_M}^1) - \underset{\text{ADPIM}}{\nu_r}(\mathrm{D_M}^1)$$

$$\frac{d\mathrm{D_M}^2}{dt} = \underset{\text{ADPIM}}{\nu_f}(\mathrm{D_I}^2) + \underset{\text{ADPMS}}{\nu_r}(\mathrm{D_S}^2) - \underset{\text{ADPMS}}{\nu_f}(\mathrm{D_M}^2) - \underset{\text{ADPIM}}{\nu_r}(\mathrm{D_M}^2)$$

$$\frac{d\mathrm{D_M}^3}{dt} = \underset{\text{ADPIM}}{\nu_f}(\mathrm{D_I}^3) + \underset{\text{ADPMS}}{\nu_r}(\mathrm{D_S}^3) - \underset{\text{ADPMS}}{\nu_f}(\mathrm{D_M}^3) - \underset{\text{ADPIM}}{\nu_r}(\mathrm{D_M}^3)$$

$$\frac{d\mathrm{D_O}^0}{dt} = \underset{\text{ADPEO}}{\nu_f}(\mathrm{D_E}^0) + \underset{\text{AKO}}{\nu_f}(2\mathrm{T_O}^{00}+\mathrm{T_O}^{01}+\mathrm{T_O}^{02}+\mathrm{T_O}^{03}+\mathrm{T_O}^{10}+\mathrm{T_O}^{20}+\mathrm{T_O}^{30}) + \underset{\text{CKO}}{\nu_f}(\mathrm{T_O}^{00}+\mathrm{T_O}^{01}+\mathrm{T_O}^{02}+\mathrm{T_O}^{03}) + \underset{\text{ADPOI}}{\nu_r}(\mathrm{D_I}^0) - \underset{\text{ADPOI}}{\nu_f}(\mathrm{D_O}^0) - \underset{\text{ADPEO}}{\nu_r}(\mathrm{D_O}^0) - \underset{\text{AKO}}{\nu_r}\big(2(\mathrm{D_O}^0+\mathrm{D_O}^1+\mathrm{D_O}^2+\mathrm{D_O}^3)\mathrm{D_O}^0\big) - \underset{\text{CKO}}{\nu_r}\big((\mathrm{C_O}^0+\mathrm{C_O}^1+\mathrm{C_O}^2+\mathrm{C_O}^3)\mathrm{D_O}^0\big)$$

$$\frac{d\mathrm{D_O}^1}{dt} = \underset{\text{ADPEO}}{\nu_f}(\mathrm{D_E}^1) + \underset{\text{AKO}}{\nu_f}(2\mathrm{T_O}^{11}+\mathrm{T_O}^{01}+\mathrm{T_O}^{10}+\mathrm{T_O}^{12}+\mathrm{T_O}^{13}+\mathrm{T_O}^{21}+\mathrm{T_O}^{31}) + \underset{\text{CKO}}{\nu_f}(\mathrm{T_O}^{10}+\mathrm{T_O}^{11}+\mathrm{T_O}^{12}+\mathrm{T_O}^{13}) + \underset{\text{ADPOI}}{\nu_r}(\mathrm{D_I}^1) - \underset{\text{ADPOI}}{\nu_f}(\mathrm{D_O}^1) - \underset{\text{ADPEO}}{\nu_r}(\mathrm{D_O}^1) - \underset{\text{AKO}}{\nu_r}\big(2(\mathrm{D_O}^0+\mathrm{D_O}^1+\mathrm{D_O}^2+\mathrm{D_O}^3)\mathrm{D_O}^1\big) - \underset{\text{CKO}}{\nu_r}\big((\mathrm{C_O}^0+\mathrm{C_O}^1+\mathrm{C_O}^2+\mathrm{C_O}^3)\mathrm{D_O}^1\big)$$

$$\frac{d\mathrm{D_O}^2}{dt} = \underset{\text{ADPEO}}{\nu_f}(\mathrm{D_E}^2) + \underset{\text{AKO}}{\nu_f}(2\mathrm{T_O}^{22}+\mathrm{T_O}^{02}+\mathrm{T_O}^{12}+\mathrm{T_O}^{20}+\mathrm{T_O}^{21}+\mathrm{T_O}^{23}+\mathrm{T_O}^{32}) + \underset{\text{CKO}}{\nu_f}(\mathrm{T_O}^{20}+\mathrm{T_O}^{21}+\mathrm{T_O}^{22}+\mathrm{T_O}^{23}) + \underset{\text{ADPOI}}{\nu_r}(\mathrm{D_I}^2) - \underset{\text{ADPOI}}{\nu_f}(\mathrm{D_O}^2) - \underset{\text{ADPEO}}{\nu_r}(\mathrm{D_O}^2) - \underset{\text{AKO}}{\nu_r}\big(2(\mathrm{D_O}^0+\mathrm{D_O}^1+\mathrm{D_O}^2+\mathrm{D_O}^3)\mathrm{D_O}^2\big) - \underset{\text{CKO}}{\nu_r}\big((\mathrm{C_O}^0+\mathrm{C_O}^1+\mathrm{C_O}^2+\mathrm{C_O}^3)\mathrm{D_O}^2\big)$$

$$\frac{d\mathrm{D_O}^3}{dt} = \underset{\text{ADPEO}}{\nu_f}(\mathrm{D_E}^3) + \underset{\text{AKO}}{\nu_f}(2\mathrm{T_O}^{33}+\mathrm{T_O}^{03}+\mathrm{T_O}^{13}+\mathrm{T_O}^{23}+\mathrm{T_O}^{30}+\mathrm{T_O}^{31}+\mathrm{T_O}^{32}) + \underset{\text{CKO}}{\nu_f}(\mathrm{T_O}^{30}+\mathrm{T_O}^{31}+\mathrm{T_O}^{32}+\mathrm{T_O}^{33}) + \underset{\text{ADPOI}}{\nu_r}(\mathrm{D_I}^3) - \underset{\text{ADPOI}}{\nu_f}(\mathrm{D_O}^3) - \underset{\text{ADPEO}}{\nu_r}(\mathrm{D_O}^3) - \underset{\text{AKO}}{\nu_r}\big(2(\mathrm{D_O}^0+\mathrm{D_O}^1+\mathrm{D_O}^2+\mathrm{D_O}^3)\mathrm{D_O}^3\big) - \underset{\text{CKO}}{\nu_r}\big((\mathrm{C_O}^0+\mathrm{C_O}^1+\mathrm{C_O}^2+\mathrm{C_O}^3)\mathrm{D_O}^3\big)$$

$$\frac{d\mathrm{D_S}^0}{dt} = \underset{\text{ADPMS}}{\nu_f}(\mathrm{D_M}^0) + \underset{\text{ASS}}{\nu_r}\big((\mathrm{T_S}^{00}+\mathrm{T_S}^{01}+\mathrm{T_S}^{02}+\mathrm{T_S}^{03})(\mathrm{W_S}^0+\mathrm{W_S}^1)\big) - \underset{\text{ASS}}{\nu_f}\big((\mathrm{P_S}^0+\mathrm{P_S}^1+\mathrm{P_S}^2+\mathrm{P_S}^3+\mathrm{P_S}^4)\mathrm{D_S}^0\big) - \underset{\text{ADPMS}}{\nu_r}(\mathrm{D_S}^0)$$

$$\frac{d\mathrm{D_S}^1}{dt} = \underset{\text{ADPMS}}{\nu_f}(\mathrm{D_M}^1) + \underset{\text{ASS}}{\nu_r}\big((\mathrm{T_S}^{10}+\mathrm{T_S}^{11}+\mathrm{T_S}^{12}+\mathrm{T_S}^{13})(\mathrm{W_S}^0+\mathrm{W_S}^1)\big) - \underset{\text{ASS}}{\nu_f}\big((\mathrm{P_S}^0+\mathrm{P_S}^1+\mathrm{P_S}^2+\mathrm{P_S}^3+\mathrm{P_S}^4)\mathrm{D_S}^1\big) - \underset{\text{ADPMS}}{\nu_r}(\mathrm{D_S}^1)$$

$$\frac{d\mathrm{D_S}^2}{dt} = \underset{\text{ADPMS}}{\nu_f}(\mathrm{D_M}^2) + \underset{\text{ASS}}{\nu_r}\big((\mathrm{T_S}^{20}+\mathrm{T_S}^{21}+\mathrm{T_S}^{22}+\mathrm{T_S}^{23})(\mathrm{W_S}^0+\mathrm{W_S}^1)\big) - \underset{\text{ASS}}{\nu_f}\big((\mathrm{P_S}^0+\mathrm{P_S}^1+\mathrm{P_S}^2+\mathrm{P_S}^3+\mathrm{P_S}^4)\mathrm{D_S}^2\big) - \underset{\text{ADPMS}}{\nu_r}(\mathrm{D_S}^2)$$

$$\frac{d\mathrm{D_S}^3}{dt} = \underset{\text{ADPMS}}{\nu_f}(\mathrm{D_M}^3) + \underset{\text{ASS}}{\nu_r}\big((\mathrm{T_S}^{30}+\mathrm{T_S}^{31}+\mathrm{T_S}^{32}+\mathrm{T_S}^{33})(\mathrm{W_S}^0+\mathrm{W_S}^1)\big) - \underset{\text{ASS}}{\nu_f}\big((\mathrm{P_S}^0+\mathrm{P_S}^1+\mathrm{P_S}^2+\mathrm{P_S}^3+\mathrm{P_S}^4)\mathrm{D_S}^3\big) - \underset{\text{ADPMS}}{\nu_r}(\mathrm{D_S}^3)$$

$$\frac{d\mathrm{T_E}^{00}}{dt} = \nu^{f}_{\mathrm{ATPoE}}\big(\mathrm{T_O}^{00}\big) + \nu^{r}_{\mathrm{ASE}}\big(\big(\mathrm{P_E}^0 + \tfrac{1}{4}\mathrm{P_E}^1\big)\mathrm{D_E}^0\big) - \nu^{f}_{\mathrm{ASE}}\big(\big(\mathrm{W_E}^0 + \mathrm{W_E}^1\big)\mathrm{T_E}^{00}\big)$$

$$\frac{d\mathrm{T_E}^{01}}{dt} = \nu^{f}_{\mathrm{ATPoE}}\big(\mathrm{T_O}^{01}\big) + \nu^{r}_{\mathrm{ASE}}\big(\big(\tfrac{1}{2}\mathrm{P_E}^2 + \tfrac{3}{4}\mathrm{P_E}^1\big)\mathrm{D_E}^0\big) - \nu^{f}_{\mathrm{ASE}}\big(\big(\mathrm{W_E}^0 + \mathrm{W_E}^1\big)\mathrm{T_E}^{01}\big)$$

$$\frac{d\mathrm{T_E}^{02}}{dt} = \nu^{f}_{\mathrm{ATPoE}}\big(\mathrm{T_O}^{02}\big) + \nu^{r}_{\mathrm{ASE}}\big(\big(\tfrac{1}{2}\mathrm{P_E}^2 + \tfrac{3}{4}\mathrm{P_E}^3\big)\mathrm{D_E}^0\big) - \nu^{f}_{\mathrm{ASE}}\big(\big(\mathrm{W_E}^0 + \mathrm{W_E}^1\big)\mathrm{T_E}^{02}\big)$$

$$\frac{d\mathrm{T_E}^{03}}{dt} = \nu^{f}_{\mathrm{ATPoE}}\big(\mathrm{T_O}^{03}\big) + \nu^{r}_{\mathrm{ASE}}\big(\big(\mathrm{P_E}^4 + \tfrac{1}{4}\mathrm{P_E}^3\big)\mathrm{D_E}^0\big) - \nu^{f}_{\mathrm{ASE}}\big(\big(\mathrm{W_E}^0 + \mathrm{W_E}^1\big)\mathrm{T_E}^{03}\big)$$

$$\frac{d\mathrm{T_E}^{10}}{dt} = \nu^{f}_{\mathrm{ATPoE}}\big(\mathrm{T_O}^{10}\big) + \nu^{r}_{\mathrm{ASE}}\big(\big(\mathrm{P_E}^0 + \tfrac{1}{4}\mathrm{P_E}^1\big)\mathrm{D_E}^1\big) - \nu^{f}_{\mathrm{ASE}}\big(\big(\mathrm{W_E}^0 + \mathrm{W_E}^1\big)\mathrm{T_E}^{10}\big)$$

$$\frac{d\mathrm{T_E}^{11}}{dt} = \nu^{f}_{\mathrm{ATPoE}}\big(\mathrm{T_O}^{11}\big) + \nu^{r}_{\mathrm{ASE}}\big(\big(\tfrac{1}{2}\mathrm{P_E}^2 + \tfrac{3}{4}\mathrm{P_E}^1\big)\mathrm{D_E}^1\big) - \nu^{f}_{\mathrm{ASE}}\big(\big(\mathrm{W_E}^0 + \mathrm{W_E}^1\big)\mathrm{T_E}^{11}\big)$$

$$\frac{d\mathrm{T_E}^{12}}{dt} = \nu^{f}_{\mathrm{ATPoE}}\big(\mathrm{T_O}^{12}\big) + \nu^{r}_{\mathrm{ASE}}\big(\big(\tfrac{1}{2}\mathrm{P_E}^2 + \tfrac{3}{4}\mathrm{P_E}^3\big)\mathrm{D_E}^1\big) - \nu^{f}_{\mathrm{ASE}}\big(\big(\mathrm{W_E}^0 + \mathrm{W_E}^1\big)\mathrm{T_E}^{12}\big)$$

$$\frac{d\mathrm{T_E}^{13}}{dt} = \nu^{f}_{\mathrm{ATPoE}}\big(\mathrm{T_O}^{13}\big) + \nu^{r}_{\mathrm{ASE}}\big(\big(\mathrm{P_E}^4 + \tfrac{1}{4}\mathrm{P_E}^3\big)\mathrm{D_E}^1\big) - \nu^{f}_{\mathrm{ASE}}\big(\big(\mathrm{W_E}^0 + \mathrm{W_E}^1\big)\mathrm{T_E}^{13}\big)$$

$$\frac{d\mathrm{T_E}^{20}}{dt} = \nu^{f}_{\mathrm{ATPoE}}\big(\mathrm{T_O}^{20}\big) + \nu^{r}_{\mathrm{ASE}}\big(\big(\mathrm{P_E}^0 + \tfrac{1}{4}\mathrm{P_E}^1\big)\mathrm{D_E}^2\big) - \nu^{f}_{\mathrm{ASE}}\big(\big(\mathrm{W_E}^0 + \mathrm{W_E}^1\big)\mathrm{T_E}^{20}\big)$$

$$\frac{d\mathrm{T_E}^{21}}{dt} = \nu^{f}_{\mathrm{ATPoE}}\big(\mathrm{T_O}^{21}\big) + \nu^{r}_{\mathrm{ASE}}\big(\big(\tfrac{1}{2}\mathrm{P_E}^2 + \tfrac{3}{4}\mathrm{P_E}^1\big)\mathrm{D_E}^2\big) - \nu^{f}_{\mathrm{ASE}}\big(\big(\mathrm{W_E}^0 + \mathrm{W_E}^1\big)\mathrm{T_E}^{21}\big)$$

$$\frac{d\mathrm{T_E}^{22}}{dt} = \nu^{f}_{\mathrm{ATPoE}}\big(\mathrm{T_O}^{22}\big) + \nu^{r}_{\mathrm{ASE}}\big(\big(\tfrac{1}{2}\mathrm{P_E}^2 + \tfrac{3}{4}\mathrm{P_E}^3\big)\mathrm{D_E}^2\big) - \nu^{f}_{\mathrm{ASE}}\big(\big(\mathrm{W_E}^0 + \mathrm{W_E}^1\big)\mathrm{T_E}^{22}\big)$$

$$\frac{d\mathrm{T_E}^{23}}{dt} = \nu^{f}_{\mathrm{ATPoE}}\big(\mathrm{T_O}^{23}\big) + \nu^{r}_{\mathrm{ASE}}\big(\big(\mathrm{P_E}^4 + \tfrac{1}{4}\mathrm{P_E}^3\big)\mathrm{D_E}^2\big) - \nu^{f}_{\mathrm{ASE}}\big(\big(\mathrm{W_E}^0 + \mathrm{W_E}^1\big)\mathrm{T_E}^{23}\big)$$

$$\frac{d\mathrm{T_E}^{30}}{dt} = \nu^{f}_{\mathrm{ATPoE}}\big(\mathrm{T_O}^{30}\big) + \nu^{r}_{\mathrm{ASE}}\big(\big(\mathrm{P_E}^0 + \tfrac{1}{4}\mathrm{P_E}^1\big)\mathrm{D_E}^3\big) - \nu^{f}_{\mathrm{ASE}}\big(\big(\mathrm{W_E}^0 + \mathrm{W_E}^1\big)\mathrm{T_E}^{30}\big)$$

$$\frac{d\mathrm{T_E}^{31}}{dt} = \nu^{f}_{\mathrm{ATPoE}}\big(\mathrm{T_O}^{31}\big) + \nu^{r}_{\mathrm{ASE}}\big(\big(\tfrac{1}{2}\mathrm{P_E}^2 + \tfrac{3}{4}\mathrm{P_E}^1\big)\mathrm{D_E}^3\big) - \nu^{f}_{\mathrm{ASE}}\big(\big(\mathrm{W_E}^0 + \mathrm{W_E}^1\big)\mathrm{T_E}^{31}\big)$$

$$\frac{d\mathrm{T_E}^{32}}{dt} = \nu^{f}_{\mathrm{ATPoE}}\big(\mathrm{T_O}^{32}\big) + \nu^{r}_{\mathrm{ASE}}\big(\big(\tfrac{1}{2}\mathrm{P_E}^2 + \tfrac{3}{4}\mathrm{P_E}^3\big)\mathrm{D_E}^3\big) - \nu^{f}_{\mathrm{ASE}}\big(\big(\mathrm{W_E}^0 + \mathrm{W_E}^1\big)\mathrm{T_E}^{32}\big)$$

$$\frac{d\mathrm{T_E}^{33}}{dt} = \nu^{f}_{\mathrm{ATPoE}}\big(\mathrm{T_O}^{33}\big) + \nu^{r}_{\mathrm{ASE}}\big(\big(\mathrm{P_E}^4 + \tfrac{1}{4}\mathrm{P_E}^3\big)\mathrm{D_E}^3\big) - \nu^{f}_{\mathrm{ASE}}\big(\big(\mathrm{W_E}^0 + \mathrm{W_E}^1\big)\mathrm{T_E}^{33}\big)$$

$$\frac{d\mathrm{T_I}^{00}}{dt} = \nu^{f}_{\mathrm{ATPMI}}\big(\mathrm{T_M}^{00}\big) + \nu^{r}_{\mathrm{AKI}}\big(\mathrm{D_I}^0\mathrm{D_I}^0\big) + \nu^{r}_{\mathrm{ATPIO}}\big(\mathrm{T_O}^{00}\big) + \nu^{r}_{\mathrm{CKI}}\big(\mathrm{D_I}^0\mathrm{C_I}^0\big) - \nu^{f}_{\mathrm{AKI}}\big(\mathrm{T_I}^{00}\big) - \nu^{f}_{\mathrm{ATPIO}}\big(\mathrm{T_I}^{00}\big) - \nu^{f}_{\mathrm{CKI}}\big(\mathrm{T_I}^{00}\big) - \nu^{r}_{\mathrm{ATPMI}}\big(\mathrm{T_I}^{00}\big)$$

$$\frac{d\mathrm{T_I}^{01}}{dt} = \nu^{f}_{\mathrm{ATPMI}}\big(\mathrm{T_M}^{01}\big) + \nu^{r}_{\mathrm{AKI}}\big(\mathrm{D_I}^0\mathrm{D_I}^1\big) + \nu^{r}_{\mathrm{ATPIO}}\big(\mathrm{T_O}^{01}\big) + \nu^{r}_{\mathrm{CKI}}\big(\mathrm{D_I}^0\mathrm{C_I}^1\big) - \nu^{f}_{\mathrm{AKI}}\big(\mathrm{T_I}^{01}\big) - \nu^{f}_{\mathrm{ATPIO}}\big(\mathrm{T_I}^{01}\big) - \nu^{f}_{\mathrm{CKI}}\big(\mathrm{T_I}^{01}\big) - \nu^{r}_{\mathrm{ATPMI}}\big(\mathrm{T_I}^{01}\big)$$

$$\frac{d\mathrm{T_I}^{02}}{dt} = \nu^{f}_{\mathrm{ATPMI}}\big(\mathrm{T_M}^{02}\big) + \nu^{r}_{\mathrm{AKI}}\big(\mathrm{D_I}^0\mathrm{D_I}^2\big) + \nu^{r}_{\mathrm{ATPIO}}\big(\mathrm{T_O}^{02}\big) + \nu^{r}_{\mathrm{CKI}}\big(\mathrm{D_I}^0\mathrm{C_I}^2\big) - \nu^{f}_{\mathrm{AKI}}\big(\mathrm{T_I}^{02}\big) - \nu^{f}_{\mathrm{ATPIO}}\big(\mathrm{T_I}^{02}\big) - \nu^{f}_{\mathrm{CKI}}\big(\mathrm{T_I}^{02}\big) - \nu^{r}_{\mathrm{ATPMI}}\big(\mathrm{T_I}^{02}\big)$$

$$\frac{d\mathrm{T_I}^{03}}{dt} = \nu^{f}_{\mathrm{ATPMI}}\big(\mathrm{T_M}^{03}\big) + \nu^{r}_{\mathrm{AKI}}\big(\mathrm{D_I}^0\mathrm{D_I}^3\big) + \nu^{r}_{\mathrm{ATPIO}}\big(\mathrm{T_O}^{03}\big) + \nu^{r}_{\mathrm{CKI}}\big(\mathrm{D_I}^0\mathrm{C_I}^3\big) - \nu^{f}_{\mathrm{AKI}}\big(\mathrm{T_I}^{03}\big) - \nu^{f}_{\mathrm{ATPIO}}\big(\mathrm{T_I}^{03}\big) - \nu^{f}_{\mathrm{CKI}}\big(\mathrm{T_I}^{03}\big) - \nu^{r}_{\mathrm{ATPMI}}\big(\mathrm{T_I}^{03}\big)$$

$$\frac{d\mathrm{T_I}^{10}}{dt} = \nu^{f}_{\mathrm{ATPMI}}\big(\mathrm{T_M}^{10}\big) + \nu^{r}_{\mathrm{AKI}}\big(\mathrm{D_I}^0\mathrm{D_I}^1\big) + \nu^{r}_{\mathrm{ATPIO}}\big(\mathrm{T_O}^{10}\big) + \nu^{r}_{\mathrm{CKI}}\big(\mathrm{D_I}^1\mathrm{C_I}^0\big) - \nu^{f}_{\mathrm{AKI}}\big(\mathrm{T_I}^{10}\big) - \nu^{f}_{\mathrm{ATPIO}}\big(\mathrm{T_I}^{10}\big) - \nu^{f}_{\mathrm{CKI}}\big(\mathrm{T_I}^{10}\big) - \nu^{r}_{\mathrm{ATPMI}}\big(\mathrm{T_I}^{10}\big)$$

$$\frac{d\mathrm{T_I}^{11}}{dt} = \nu^{f}_{\mathrm{ATPMI}}\big(\mathrm{T_M}^{11}\big) + \nu^{r}_{\mathrm{AKI}}\big(\mathrm{D_I}^1\mathrm{D_I}^1\big) + \nu^{r}_{\mathrm{ATPIO}}\big(\mathrm{T_O}^{11}\big) + \nu^{r}_{\mathrm{CKI}}\big(\mathrm{D_I}^1\mathrm{C_I}^1\big) - \nu^{f}_{\mathrm{AKI}}\big(\mathrm{T_I}^{11}\big) - \nu^{f}_{\mathrm{ATPIO}}\big(\mathrm{T_I}^{11}\big) - \nu^{f}_{\mathrm{CKI}}\big(\mathrm{T_I}^{11}\big) - \nu^{r}_{\mathrm{ATPMI}}\big(\mathrm{T_I}^{11}\big)$$

$$\frac{d\mathrm{T_I}^{12}}{dt} = \nu^{f}_{\mathrm{ATPMI}}\big(\mathrm{T_M}^{12}\big) + \nu^{r}_{\mathrm{AKI}}\big(\mathrm{D_I}^1\mathrm{D_I}^2\big) + \nu^{r}_{\mathrm{ATPIO}}\big(\mathrm{T_O}^{12}\big) + \nu^{r}_{\mathrm{CKI}}\big(\mathrm{D_I}^1\mathrm{C_I}^2\big) - \nu^{f}_{\mathrm{AKI}}\big(\mathrm{T_I}^{12}\big) - \nu^{f}_{\mathrm{ATPIO}}\big(\mathrm{T_I}^{12}\big) - \nu^{f}_{\mathrm{CKI}}\big(\mathrm{T_I}^{12}\big) - \nu^{r}_{\mathrm{ATPMI}}\big(\mathrm{T_I}^{12}\big)$$

$$\frac{d\mathrm{T_I}^{13}}{dt} = \nu^{f}_{\mathrm{ATPMI}}\big(\mathrm{T_M}^{13}\big) + \nu^{r}_{\mathrm{AKI}}\big(\mathrm{D_I}^1\mathrm{D_I}^3\big) + \nu^{r}_{\mathrm{ATPIO}}\big(\mathrm{T_O}^{13}\big) + \nu^{r}_{\mathrm{CKI}}\big(\mathrm{D_I}^1\mathrm{C_I}^3\big) - \nu^{f}_{\mathrm{AKI}}\big(\mathrm{T_I}^{13}\big) - \nu^{f}_{\mathrm{ATPIO}}\big(\mathrm{T_I}^{13}\big) - \nu^{f}_{\mathrm{CKI}}\big(\mathrm{T_I}^{13}\big) - \nu^{r}_{\mathrm{ATPMI}}\big(\mathrm{T_I}^{13}\big)$$

$$\frac{d\mathrm{T_I}^{20}}{dt} = \nu^{f}_{\mathrm{ATPMI}}\big(\mathrm{T_M}^{20}\big) + \nu^{r}_{\mathrm{AKI}}\big(\mathrm{D_I}^0\mathrm{D_I}^2\big) + \nu^{r}_{\mathrm{ATPIO}}\big(\mathrm{T_O}^{20}\big) + \nu^{r}_{\mathrm{CKI}}\big(\mathrm{D_I}^2\mathrm{C_I}^0\big) - \nu^{f}_{\mathrm{AKI}}\big(\mathrm{T_I}^{20}\big) - \nu^{f}_{\mathrm{ATPIO}}\big(\mathrm{T_I}^{20}\big) - \nu^{f}_{\mathrm{CKI}}\big(\mathrm{T_I}^{20}\big) - \nu^{r}_{\mathrm{ATPMI}}\big(\mathrm{T_I}^{20}\big)$$

$$\frac{d\mathrm{T_I}^{21}}{dt} = \underset{\mathrm{ATP_{MI}}}{\nu_f}(\mathrm{T_M}^{21}) + \underset{\mathrm{AK_I}}{\nu_r}(\mathrm{D_I}^{1}\mathrm{D_I}^{2}) + \underset{\mathrm{ATP_{IO}}}{\nu_r}(\mathrm{T_O}^{21}) + \underset{\mathrm{CK_I}}{\nu_r}(\mathrm{D_I}^{2}\mathrm{C_I}^{1}) - \underset{\mathrm{AK_I}}{\nu_f}(\mathrm{T_I}^{21}) - \underset{\mathrm{ATP_{IO}}}{\nu_f}(\mathrm{T_I}^{21}) - \underset{\mathrm{CK_I}}{\nu_f}(\mathrm{T_I}^{21}) - \underset{\mathrm{ATP_{MI}}}{\nu_r}(\mathrm{T_I}^{21})$$

$$\frac{d\mathrm{T_I}^{22}}{dt} = \underset{\mathrm{ATP_{MI}}}{\nu_f}(\mathrm{T_M}^{22}) + \underset{\mathrm{AK_I}}{\nu_r}(\mathrm{D_I}^{2}\mathrm{D_I}^{2}) + \underset{\mathrm{ATP_{IO}}}{\nu_r}(\mathrm{T_O}^{22}) + \underset{\mathrm{CK_I}}{\nu_r}(\mathrm{D_I}^{2}\mathrm{C_I}^{2}) - \underset{\mathrm{AK_I}}{\nu_f}(\mathrm{T_I}^{22}) - \underset{\mathrm{ATP_{IO}}}{\nu_f}(\mathrm{T_I}^{22}) - \underset{\mathrm{CK_I}}{\nu_f}(\mathrm{T_I}^{22}) - \underset{\mathrm{ATP_{MI}}}{\nu_r}(\mathrm{T_I}^{22})$$

$$\frac{d\mathrm{T_I}^{23}}{dt} = \underset{\mathrm{ATP_{MI}}}{\nu_f}(\mathrm{T_M}^{23}) + \underset{\mathrm{AK_I}}{\nu_r}(\mathrm{D_I}^{2}\mathrm{D_I}^{3}) + \underset{\mathrm{ATP_{IO}}}{\nu_r}(\mathrm{T_O}^{23}) + \underset{\mathrm{CK_I}}{\nu_r}(\mathrm{D_I}^{2}\mathrm{C_I}^{3}) - \underset{\mathrm{AK_I}}{\nu_f}(\mathrm{T_I}^{23}) - \underset{\mathrm{ATP_{IO}}}{\nu_f}(\mathrm{T_I}^{23}) - \underset{\mathrm{CK_I}}{\nu_f}(\mathrm{T_I}^{23}) - \underset{\mathrm{ATP_{MI}}}{\nu_r}(\mathrm{T_I}^{23})$$

$$\frac{d\mathrm{T_I}^{30}}{dt} = \underset{\mathrm{ATP_{MI}}}{\nu_f}(\mathrm{T_M}^{30}) + \underset{\mathrm{AK_I}}{\nu_r}(\mathrm{D_I}^{0}\mathrm{D_I}^{3}) + \underset{\mathrm{ATP_{IO}}}{\nu_r}(\mathrm{T_O}^{30}) + \underset{\mathrm{CK_I}}{\nu_r}(\mathrm{D_I}^{3}\mathrm{C_I}^{0}) - \underset{\mathrm{AK_I}}{\nu_f}(\mathrm{T_I}^{30}) - \underset{\mathrm{ATP_{IO}}}{\nu_f}(\mathrm{T_I}^{30}) - \underset{\mathrm{CK_I}}{\nu_f}(\mathrm{T_I}^{30}) - \underset{\mathrm{ATP_{MI}}}{\nu_r}(\mathrm{T_I}^{30})$$

$$\frac{d\mathrm{T_I}^{31}}{dt} = \underset{\mathrm{ATP_{MI}}}{\nu_f}(\mathrm{T_M}^{31}) + \underset{\mathrm{AK_I}}{\nu_r}(\mathrm{D_I}^{1}\mathrm{D_I}^{3}) + \underset{\mathrm{ATP_{IO}}}{\nu_r}(\mathrm{T_O}^{31}) + \underset{\mathrm{CK_I}}{\nu_r}(\mathrm{D_I}^{3}\mathrm{C_I}^{1}) - \underset{\mathrm{AK_I}}{\nu_f}(\mathrm{T_I}^{31}) - \underset{\mathrm{ATP_{IO}}}{\nu_f}(\mathrm{T_I}^{31}) - \underset{\mathrm{CK_I}}{\nu_f}(\mathrm{T_I}^{31}) - \underset{\mathrm{ATP_{MI}}}{\nu_r}(\mathrm{T_I}^{31})$$

$$\frac{d\mathrm{T_I}^{32}}{dt} = \underset{\mathrm{ATP_{MI}}}{\nu_f}(\mathrm{T_M}^{32}) + \underset{\mathrm{AK_I}}{\nu_r}(\mathrm{D_I}^{2}\mathrm{D_I}^{3}) + \underset{\mathrm{ATP_{IO}}}{\nu_r}(\mathrm{T_O}^{32}) + \underset{\mathrm{CK_I}}{\nu_r}(\mathrm{D_I}^{3}\mathrm{C_I}^{2}) - \underset{\mathrm{AK_I}}{\nu_f}(\mathrm{T_I}^{32}) - \underset{\mathrm{ATP_{IO}}}{\nu_f}(\mathrm{T_I}^{32}) - \underset{\mathrm{CK_I}}{\nu_f}(\mathrm{T_I}^{32}) - \underset{\mathrm{ATP_{MI}}}{\nu_r}(\mathrm{T_I}^{32})$$

$$\frac{d\mathrm{T_I}^{33}}{dt} = \underset{\mathrm{ATP_{MI}}}{\nu_f}(\mathrm{T_M}^{33}) + \underset{\mathrm{AK_I}}{\nu_r}(\mathrm{D_I}^{3}\mathrm{D_I}^{3}) + \underset{\mathrm{ATP_{IO}}}{\nu_r}(\mathrm{T_O}^{33}) + \underset{\mathrm{CK_I}}{\nu_r}(\mathrm{D_I}^{3}\mathrm{C_I}^{3}) - \underset{\mathrm{AK_I}}{\nu_f}(\mathrm{T_I}^{33}) - \underset{\mathrm{ATP_{IO}}}{\nu_f}(\mathrm{T_I}^{33}) - \underset{\mathrm{CK_I}}{\nu_f}(\mathrm{T_I}^{33}) - \underset{\mathrm{ATP_{MI}}}{\nu_r}(\mathrm{T_I}^{33})$$

$$\frac{d\mathrm{T_M}^{00}}{dt} = \underset{\mathrm{ATP_{SM}}}{\nu_f}(\mathrm{T_S}^{00}) + \underset{\mathrm{ATP_{MI}}}{\nu_r}(\mathrm{T_I}^{00}) - \underset{\mathrm{ATP_{MI}}}{\nu_f}(\mathrm{T_M}^{00})$$

$$\frac{d\mathrm{T_M}^{01}}{dt} = \underset{\mathrm{ATP_{SM}}}{\nu_f}(\mathrm{T_S}^{01}) + \underset{\mathrm{ATP_{MI}}}{\nu_r}(\mathrm{T_I}^{01}) - \underset{\mathrm{ATP_{MI}}}{\nu_f}(\mathrm{T_M}^{01})$$

$$\frac{d\mathrm{T_M}^{02}}{dt} = \underset{\mathrm{ATP_{SM}}}{\nu_f}(\mathrm{T_S}^{02}) + \underset{\mathrm{ATP_{MI}}}{\nu_r}(\mathrm{T_I}^{02}) - \underset{\mathrm{ATP_{MI}}}{\nu_f}(\mathrm{T_M}^{02})$$

$$\frac{d\mathrm{T_M}^{03}}{dt} = \underset{\mathrm{ATP_{SM}}}{\nu_f}(\mathrm{T_S}^{03}) + \underset{\mathrm{ATP_{MI}}}{\nu_r}(\mathrm{T_I}^{03}) - \underset{\mathrm{ATP_{MI}}}{\nu_f}(\mathrm{T_M}^{03})$$

$$\frac{d\mathrm{T_M}^{10}}{dt} = \underset{\mathrm{ATP_{SM}}}{\nu_f}(\mathrm{T_S}^{10}) + \underset{\mathrm{ATP_{MI}}}{\nu_r}(\mathrm{T_I}^{10}) - \underset{\mathrm{ATP_{MI}}}{\nu_f}(\mathrm{T_M}^{10})$$

$$\frac{d\mathrm{T_M}^{11}}{dt} = \underset{\mathrm{ATP_{SM}}}{\nu_f}(\mathrm{T_S}^{11}) + \underset{\mathrm{ATP_{MI}}}{\nu_r}(\mathrm{T_I}^{11}) - \underset{\mathrm{ATP_{MI}}}{\nu_f}(\mathrm{T_M}^{11})$$

$$\frac{d\mathrm{T_M}^{12}}{dt} = \underset{\mathrm{ATP_{SM}}}{\nu_f}(\mathrm{T_S}^{12}) + \underset{\mathrm{ATP_{MI}}}{\nu_r}(\mathrm{T_I}^{12}) - \underset{\mathrm{ATP_{MI}}}{\nu_f}(\mathrm{T_M}^{12})$$

$$\frac{d\mathrm{T_M}^{13}}{dt} = \underset{\mathrm{ATP_{SM}}}{\nu_f}(\mathrm{T_S}^{13}) + \underset{\mathrm{ATP_{MI}}}{\nu_r}(\mathrm{T_I}^{13}) - \underset{\mathrm{ATP_{MI}}}{\nu_f}(\mathrm{T_M}^{13})$$

$$\frac{d\mathrm{T_M}^{20}}{dt} = \underset{\mathrm{ATP_{SM}}}{\nu_f}(\mathrm{T_S}^{20}) + \underset{\mathrm{ATP_{MI}}}{\nu_r}(\mathrm{T_I}^{20}) - \underset{\mathrm{ATP_{MI}}}{\nu_f}(\mathrm{T_M}^{20})$$

$$\frac{d\mathrm{T_M}^{21}}{dt} = \underset{\mathrm{ATP_{SM}}}{\nu_f}(\mathrm{T_S}^{21}) + \underset{\mathrm{ATP_{MI}}}{\nu_r}(\mathrm{T_I}^{21}) - \underset{\mathrm{ATP_{MI}}}{\nu_f}(\mathrm{T_M}^{21})$$

$$\frac{d\mathrm{T_M}^{22}}{dt} = \underset{\mathrm{ATP_{SM}}}{\nu_f}(\mathrm{T_S}^{22}) + \underset{\mathrm{ATP_{MI}}}{\nu_r}(\mathrm{T_I}^{22}) - \underset{\mathrm{ATP_{MI}}}{\nu_f}(\mathrm{T_M}^{22})$$

$$\frac{d\mathrm{T_M}^{23}}{dt} = \underset{\mathrm{ATP_{SM}}}{\nu_f}(\mathrm{T_S}^{23}) + \underset{\mathrm{ATP_{MI}}}{\nu_r}(\mathrm{T_I}^{23}) - \underset{\mathrm{ATP_{MI}}}{\nu_f}(\mathrm{T_M}^{23})$$

$$\frac{d\mathrm{T_M}^{30}}{dt} = \underset{\mathrm{ATP_{SM}}}{\nu_f}(\mathrm{T_S}^{30}) + \underset{\mathrm{ATP_{MI}}}{\nu_r}(\mathrm{T_I}^{30}) - \underset{\mathrm{ATP_{MI}}}{\nu_f}(\mathrm{T_M}^{30})$$

$$\frac{d\mathrm{T_M}^{31}}{dt} = \underset{\mathrm{ATP_{SM}}}{\nu_f}(\mathrm{T_S}^{31}) + \underset{\mathrm{ATP_{MI}}}{\nu_r}(\mathrm{T_I}^{31}) - \underset{\mathrm{ATP_{MI}}}{\nu_f}(\mathrm{T_M}^{31})$$

$$\frac{d\mathrm{T_M}^{32}}{dt} = \underset{\mathrm{ATP_{SM}}}{\nu_f}(\mathrm{T_S}^{32}) + \underset{\mathrm{ATP_{MI}}}{\nu_r}(\mathrm{T_I}^{32}) - \underset{\mathrm{ATP_{MI}}}{\nu_f}(\mathrm{T_M}^{32})$$

$$\frac{d\mathrm{T_M}^{33}}{dt} = \underset{\mathrm{ATP_{SM}}}{\nu_f}(\mathrm{T_S}^{33}) + \underset{\mathrm{ATP_{MI}}}{\nu_r}(\mathrm{T_I}^{33}) - \underset{\mathrm{ATP_{MI}}}{\nu_f}(\mathrm{T_M}^{33})$$

$$\frac{d\mathrm{T_O}^{00}}{dt} = \underset{\mathrm{ATP_{IO}}}{\nu_f}(\mathrm{T_I}^{00}) + \underset{\mathrm{AK_O}}{\nu_r}(\mathrm{D_O}^{0}\mathrm{D_O}^{0}) + \underset{\mathrm{CK_O}}{\nu_r}(\mathrm{D_O}^{0}\mathrm{C_O}^{0}) - \underset{\mathrm{AK_O}}{\nu_f}(\mathrm{T_O}^{00}) - \underset{\mathrm{ATP_{OE}}}{\nu_f}(\mathrm{T_O}^{00}) - \underset{\mathrm{CK_O}}{\nu_f}(\mathrm{T_O}^{00}) - \underset{\mathrm{ATP_{IO}}}{\nu_r}(\mathrm{T_O}^{00})$$

$$\frac{d\mathrm{T_O}^{01}}{dt} = \underset{\mathrm{ATP_{IO}}}{\nu_f}(\mathrm{T_I}^{01}) + \underset{\mathrm{AK_O}}{\nu_r}(\mathrm{D_O}^{0}\mathrm{D_O}^{1}) + \underset{\mathrm{CK_O}}{\nu_r}(\mathrm{D_O}^{0}\mathrm{C_O}^{1}) - \underset{\mathrm{AK_O}}{\nu_f}(\mathrm{T_O}^{01}) - \underset{\mathrm{ATP_{OE}}}{\nu_f}(\mathrm{T_O}^{01}) - \underset{\mathrm{CK_O}}{\nu_f}(\mathrm{T_O}^{01}) - \underset{\mathrm{ATP_{IO}}}{\nu_r}(\mathrm{T_O}^{01})$$

$$\frac{dT_O{}^{02}}{dt} = \nu^{f}_{\mathrm{ATPIO}}\big(T_I{}^{02}\big) + \nu^{r}_{\mathrm{AKO}}\big(D_O{}^{0}D_O{}^{2}\big) + \nu^{r}_{\mathrm{CKO}}\big(D_O{}^{0}C_O{}^{2}\big) - \nu^{f}_{\mathrm{AKO}}\big(T_O{}^{02}\big) - \nu^{f}_{\mathrm{ATPOE}}\big(T_O{}^{02}\big) - \nu^{f}_{\mathrm{CKO}}\big(T_O{}^{02}\big) - \nu^{r}_{\mathrm{ATPIO}}\big(T_O{}^{02}\big)$$

$$\frac{dT_O{}^{03}}{dt} = \nu^{f}_{\mathrm{ATPIO}}\big(T_I{}^{03}\big) + \nu^{r}_{\mathrm{AKO}}\big(D_O{}^{0}D_O{}^{3}\big) + \nu^{r}_{\mathrm{CKO}}\big(D_O{}^{0}C_O{}^{3}\big) - \nu^{f}_{\mathrm{AKO}}\big(T_O{}^{03}\big) - \nu^{f}_{\mathrm{ATPOE}}\big(T_O{}^{03}\big) - \nu^{f}_{\mathrm{CKO}}\big(T_O{}^{03}\big) - \nu^{r}_{\mathrm{ATPIO}}\big(T_O{}^{03}\big)$$

$$\frac{dT_O{}^{10}}{dt} = \nu^{f}_{\mathrm{ATPIO}}\big(T_I{}^{10}\big) + \nu^{r}_{\mathrm{AKO}}\big(D_O{}^{0}D_O{}^{1}\big) + \nu^{r}_{\mathrm{CKO}}\big(D_O{}^{1}C_O{}^{0}\big) - \nu^{f}_{\mathrm{AKO}}\big(T_O{}^{10}\big) - \nu^{f}_{\mathrm{ATPOE}}\big(T_O{}^{10}\big) - \nu^{f}_{\mathrm{CKO}}\big(T_O{}^{10}\big) - \nu^{r}_{\mathrm{ATPIO}}\big(T_O{}^{10}\big)$$

$$\frac{dT_O{}^{11}}{dt} = \nu^{f}_{\mathrm{ATPIO}}\big(T_I{}^{11}\big) + \nu^{r}_{\mathrm{AKO}}\big(D_O{}^{1}D_O{}^{1}\big) + \nu^{r}_{\mathrm{CKO}}\big(D_O{}^{1}C_O{}^{1}\big) - \nu^{f}_{\mathrm{AKO}}\big(T_O{}^{11}\big) - \nu^{f}_{\mathrm{ATPOE}}\big(T_O{}^{11}\big) - \nu^{f}_{\mathrm{CKO}}\big(T_O{}^{11}\big) - \nu^{r}_{\mathrm{ATPIO}}\big(T_O{}^{11}\big)$$

$$\frac{dT_O{}^{12}}{dt} = \nu^{f}_{\mathrm{ATPIO}}\big(T_I{}^{12}\big) + \nu^{r}_{\mathrm{AKO}}\big(D_O{}^{1}D_O{}^{2}\big) + \nu^{r}_{\mathrm{CKO}}\big(D_O{}^{1}C_O{}^{2}\big) - \nu^{f}_{\mathrm{AKO}}\big(T_O{}^{12}\big) - \nu^{f}_{\mathrm{ATPOE}}\big(T_O{}^{12}\big) - \nu^{f}_{\mathrm{CKO}}\big(T_O{}^{12}\big) - \nu^{r}_{\mathrm{ATPIO}}\big(T_O{}^{12}\big)$$

$$\frac{dT_O{}^{13}}{dt} = \nu^{f}_{\mathrm{ATPIO}}\big(T_I{}^{13}\big) + \nu^{r}_{\mathrm{AKO}}\big(D_O{}^{1}D_O{}^{3}\big) + \nu^{r}_{\mathrm{CKO}}\big(D_O{}^{1}C_O{}^{3}\big) - \nu^{f}_{\mathrm{AKO}}\big(T_O{}^{13}\big) - \nu^{f}_{\mathrm{ATPOE}}\big(T_O{}^{13}\big) - \nu^{f}_{\mathrm{CKO}}\big(T_O{}^{13}\big) - \nu^{r}_{\mathrm{ATPIO}}\big(T_O{}^{13}\big)$$

$$\frac{dT_O{}^{20}}{dt} = \nu^{f}_{\mathrm{ATPIO}}\big(T_I{}^{20}\big) + \nu^{r}_{\mathrm{AKO}}\big(D_O{}^{0}D_O{}^{2}\big) + \nu^{r}_{\mathrm{CKO}}\big(D_O{}^{2}C_O{}^{0}\big) - \nu^{f}_{\mathrm{AKO}}\big(T_O{}^{20}\big) - \nu^{f}_{\mathrm{ATPOE}}\big(T_O{}^{20}\big) - \nu^{f}_{\mathrm{CKO}}\big(T_O{}^{20}\big) - \nu^{r}_{\mathrm{ATPIO}}\big(T_O{}^{20}\big)$$

$$\frac{dT_O{}^{21}}{dt} = \nu^{f}_{\mathrm{ATPIO}}\big(T_I{}^{21}\big) + \nu^{r}_{\mathrm{AKO}}\big(D_O{}^{1}D_O{}^{2}\big) + \nu^{r}_{\mathrm{CKO}}\big(D_O{}^{2}C_O{}^{1}\big) - \nu^{f}_{\mathrm{AKO}}\big(T_O{}^{21}\big) - \nu^{f}_{\mathrm{ATPOE}}\big(T_O{}^{21}\big) - \nu^{f}_{\mathrm{CKO}}\big(T_O{}^{21}\big) - \nu^{r}_{\mathrm{ATPIO}}\big(T_O{}^{21}\big)$$

$$\frac{dT_O{}^{22}}{dt} = \nu^{f}_{\mathrm{ATPIO}}\big(T_I{}^{22}\big) + \nu^{r}_{\mathrm{AKO}}\big(D_O{}^{2}D_O{}^{2}\big) + \nu^{r}_{\mathrm{CKO}}\big(D_O{}^{2}C_O{}^{2}\big) - \nu^{f}_{\mathrm{AKO}}\big(T_O{}^{22}\big) - \nu^{f}_{\mathrm{ATPOE}}\big(T_O{}^{22}\big) - \nu^{f}_{\mathrm{CKO}}\big(T_O{}^{22}\big) - \nu^{r}_{\mathrm{ATPIO}}\big(T_O{}^{22}\big)$$

$$\frac{dT_O{}^{23}}{dt} = \nu^{f}_{\mathrm{ATPIO}}\big(T_I{}^{23}\big) + \nu^{r}_{\mathrm{AKO}}\big(D_O{}^{2}D_O{}^{3}\big) + \nu^{r}_{\mathrm{CKO}}\big(D_O{}^{2}C_O{}^{3}\big) - \nu^{f}_{\mathrm{AKO}}\big(T_O{}^{23}\big) - \nu^{f}_{\mathrm{ATPOE}}\big(T_O{}^{23}\big) - \nu^{f}_{\mathrm{CKO}}\big(T_O{}^{23}\big) - \nu^{r}_{\mathrm{ATPIO}}\big(T_O{}^{23}\big)$$

$$\frac{dT_O{}^{30}}{dt} = \nu^{f}_{\mathrm{ATPIO}}\big(T_I{}^{30}\big) + \nu^{r}_{\mathrm{AKO}}\big(D_O{}^{0}D_O{}^{3}\big) + \nu^{r}_{\mathrm{CKO}}\big(D_O{}^{3}C_O{}^{0}\big) - \nu^{f}_{\mathrm{AKO}}\big(T_O{}^{30}\big) - \nu^{f}_{\mathrm{ATPOE}}\big(T_O{}^{30}\big) - \nu^{f}_{\mathrm{CKO}}\big(T_O{}^{30}\big) - \nu^{r}_{\mathrm{ATPIO}}\big(T_O{}^{30}\big)$$

$$\frac{dT_O{}^{31}}{dt} = \nu^{f}_{\mathrm{ATPIO}}\big(T_I{}^{31}\big) + \nu^{r}_{\mathrm{AKO}}\big(D_O{}^{1}D_O{}^{3}\big) + \nu^{r}_{\mathrm{CKO}}\big(D_O{}^{3}C_O{}^{1}\big) - \nu^{f}_{\mathrm{AKO}}\big(T_O{}^{31}\big) - \nu^{f}_{\mathrm{ATPOE}}\big(T_O{}^{31}\big) - \nu^{f}_{\mathrm{CKO}}\big(T_O{}^{31}\big) - \nu^{r}_{\mathrm{ATPIO}}\big(T_O{}^{31}\big)$$

$$\frac{dT_O{}^{32}}{dt} = \nu^{f}_{\mathrm{ATPIO}}\big(T_I{}^{32}\big) + \nu^{r}_{\mathrm{AKO}}\big(D_O{}^{2}D_O{}^{3}\big) + \nu^{r}_{\mathrm{CKO}}\big(D_O{}^{3}C_O{}^{2}\big) - \nu^{f}_{\mathrm{AKO}}\big(T_O{}^{32}\big) - \nu^{f}_{\mathrm{ATPOE}}\big(T_O{}^{32}\big) - \nu^{f}_{\mathrm{CKO}}\big(T_O{}^{32}\big) - \nu^{r}_{\mathrm{ATPIO}}\big(T_O{}^{32}\big)$$

$$\frac{dT_O{}^{33}}{dt} = \nu^{f}_{\mathrm{ATPIO}}\big(T_I{}^{33}\big) + \nu^{r}_{\mathrm{AKO}}\big(D_O{}^{3}D_O{}^{3}\big) + \nu^{r}_{\mathrm{CKO}}\big(D_O{}^{3}C_O{}^{3}\big) - \nu^{f}_{\mathrm{AKO}}\big(T_O{}^{33}\big) - \nu^{f}_{\mathrm{ATPOE}}\big(T_O{}^{33}\big) - \nu^{f}_{\mathrm{CKO}}\big(T_O{}^{33}\big) - \nu^{r}_{\mathrm{ATPIO}}\big(T_O{}^{33}\big)$$

$$\frac{dT_S{}^{00}}{dt} = \nu^{f}_{\mathrm{ASS}}\Big(\big(P_S{}^{0} + \tfrac{1}{4}P_S{}^{1}\big)D_S{}^{0}\Big) - \nu^{f}_{\mathrm{ATPSM}}\big(T_S{}^{00}\big) - \nu^{r}_{\mathrm{ASS}}\Big(\big(W_S{}^{0} + W_S{}^{1}\big)T_S{}^{00}\Big)$$

$$\frac{dT_S{}^{01}}{dt} = \nu^{f}_{\mathrm{ASS}}\Big(\big(\tfrac{1}{2}P_S{}^{2} + \tfrac{3}{4}P_S{}^{1}\big)D_S{}^{0}\Big) - \nu^{f}_{\mathrm{ATPSM}}\big(T_S{}^{01}\big) - \nu^{r}_{\mathrm{ASS}}\Big(\big(W_S{}^{0} + W_S{}^{1}\big)T_S{}^{01}\Big)$$

$$\frac{dT_S{}^{02}}{dt} = \nu^{f}_{\mathrm{ASS}}\Big(\big(\tfrac{1}{2}P_S{}^{2} + \tfrac{3}{4}P_S{}^{3}\big)D_S{}^{0}\Big) - \nu^{f}_{\mathrm{ATPSM}}\big(T_S{}^{02}\big) - \nu^{r}_{\mathrm{ASS}}\Big(\big(W_S{}^{0} + W_S{}^{1}\big)T_S{}^{02}\Big)$$

$$\frac{dT_S{}^{03}}{dt} = \nu^{f}_{\mathrm{ASS}}\Big(\big(P_S{}^{4} + \tfrac{1}{4}P_S{}^{3}\big)D_S{}^{0}\Big) - \nu^{f}_{\mathrm{ATPSM}}\big(T_S{}^{03}\big) - \nu^{r}_{\mathrm{ASS}}\Big(\big(W_S{}^{0} + W_S{}^{1}\big)T_S{}^{03}\Big)$$

$$\frac{dT_S{}^{10}}{dt} = \nu^{f}_{\mathrm{ASS}}\Big(\big(P_S{}^{0} + \tfrac{1}{4}P_S{}^{1}\big)D_S{}^{1}\Big) - \nu^{f}_{\mathrm{ATPSM}}\big(T_S{}^{10}\big) - \nu^{r}_{\mathrm{ASS}}\Big(\big(W_S{}^{0} + W_S{}^{1}\big)T_S{}^{10}\Big)$$

$$\frac{dT_S{}^{11}}{dt} = \nu^{f}_{\mathrm{ASS}}\Big(\big(\tfrac{1}{2}P_S{}^{2} + \tfrac{3}{4}P_S{}^{1}\big)D_S{}^{1}\Big) - \nu^{f}_{\mathrm{ATPSM}}\big(T_S{}^{11}\big) - \nu^{r}_{\mathrm{ASS}}\Big(\big(W_S{}^{0} + W_S{}^{1}\big)T_S{}^{11}\Big)$$

$$\frac{dT_S{}^{12}}{dt} = \nu^{f}_{\mathrm{ASS}}\Big(\big(\tfrac{1}{2}P_S{}^{2} + \tfrac{3}{4}P_S{}^{3}\big)D_S{}^{1}\Big) - \nu^{f}_{\mathrm{ATPSM}}\big(T_S{}^{12}\big) - \nu^{r}_{\mathrm{ASS}}\Big(\big(W_S{}^{0} + W_S{}^{1}\big)T_S{}^{12}\Big)$$

$$\frac{dT_S{}^{13}}{dt} = \nu^{f}_{\mathrm{ASS}}\Big(\big(P_S{}^{4} + \tfrac{1}{4}P_S{}^{3}\big)D_S{}^{1}\Big) - \nu^{f}_{\mathrm{ATPSM}}\big(T_S{}^{13}\big) - \nu^{r}_{\mathrm{ASS}}\Big(\big(W_S{}^{0} + W_S{}^{1}\big)T_S{}^{13}\Big)$$

$$\frac{dT_S{}^{20}}{dt} = \nu^{f}_{\mathrm{ASS}}\Big(\big(P_S{}^{0} + \tfrac{1}{4}P_S{}^{1}\big)D_S{}^{2}\Big) - \nu^{f}_{\mathrm{ATPSM}}\big(T_S{}^{20}\big) - \nu^{r}_{\mathrm{ASS}}\Big(\big(W_S{}^{0} + W_S{}^{1}\big)T_S{}^{20}\Big)$$

$$\frac{dT_S{}^{21}}{dt} = \nu^{f}_{\mathrm{ASS}}\Big(\big(\tfrac{1}{2}P_S{}^{2} + \tfrac{3}{4}P_S{}^{1}\big)D_S{}^{2}\Big) - \nu^{f}_{\mathrm{ATPSM}}\big(T_S{}^{21}\big) - \nu^{r}_{\mathrm{ASS}}\Big(\big(W_S{}^{0} + W_S{}^{1}\big)T_S{}^{21}\Big)$$

$$\frac{dT_S{}^{22}}{dt} = \nu^{f}_{\mathrm{ASS}}\Big(\big(\tfrac{1}{2}P_S{}^{2} + \tfrac{3}{4}P_S{}^{3}\big)D_S{}^{2}\Big) - \nu^{f}_{\mathrm{ATPSM}}\big(T_S{}^{22}\big) - \nu^{r}_{\mathrm{ASS}}\Big(\big(W_S{}^{0} + W_S{}^{1}\big)T_S{}^{22}\Big)$$

$$\frac{d\mathrm{T_S}^{23}}{dt} = \nu^{f}_{\mathrm{ASs}}\big((\mathrm{P_S}^4 + \tfrac{1}{4}\mathrm{P_S}^3)\mathrm{D_S}^2\big) - \nu^{f}_{\mathrm{ATPsm}}\big(\mathrm{T_S}^{23}\big) - \nu^{r}_{\mathrm{ASs}}\big((\mathrm{W_S}^0 + \mathrm{W_S}^1)\mathrm{T_S}^{23}\big)$$

$$\frac{d\mathrm{T_S}^{30}}{dt} = \nu^{f}_{\mathrm{ASs}}\big((\mathrm{P_S}^0 + \tfrac{1}{4}\mathrm{P_S}^1)\mathrm{D_S}^3\big) - \nu^{f}_{\mathrm{ATPsm}}\big(\mathrm{T_S}^{30}\big) - \nu^{r}_{\mathrm{ASs}}\big((\mathrm{W_S}^0 + \mathrm{W_S}^1)\mathrm{T_S}^{30}\big)$$

$$\frac{d\mathrm{T_S}^{31}}{dt} = \nu^{f}_{\mathrm{ASs}}\big((\tfrac{1}{2}\mathrm{P_S}^2 + \tfrac{3}{4}\mathrm{P_S}^1)\mathrm{D_S}^3\big) - \nu^{f}_{\mathrm{ATPsm}}\big(\mathrm{T_S}^{31}\big) - \nu^{r}_{\mathrm{ASs}}\big((\mathrm{W_S}^0 + \mathrm{W_S}^1)\mathrm{T_S}^{31}\big)$$

$$\frac{d\mathrm{T_S}^{32}}{dt} = \nu^{f}_{\mathrm{ASs}}\big((\tfrac{1}{2}\mathrm{P_S}^2 + \tfrac{3}{4}\mathrm{P_S}^3)\mathrm{D_S}^3\big) - \nu^{f}_{\mathrm{ATPsm}}\big(\mathrm{T_S}^{32}\big) - \nu^{r}_{\mathrm{ASs}}\big((\mathrm{W_S}^0 + \mathrm{W_S}^1)\mathrm{T_S}^{32}\big)$$

$$\frac{d\mathrm{T_S}^{33}}{dt} = \nu^{f}_{\mathrm{ASs}}\big((\mathrm{P_S}^4 + \tfrac{1}{4}\mathrm{P_S}^3)\mathrm{D_S}^3\big) - \nu^{f}_{\mathrm{ATPsm}}\big(\mathrm{T_S}^{33}\big) - \nu^{r}_{\mathrm{ASs}}\big((\mathrm{W_S}^0 + \mathrm{W_S}^1)\mathrm{T_S}^{33}\big)$$

$$\frac{d\mathrm{C_I}^0}{dt} = \nu^{f}_{\mathrm{CKi}}\big(\mathrm{T_I}^{00} + \mathrm{T_I}^{10} + \mathrm{T_I}^{20} + \mathrm{T_I}^{30}\big) + \nu^{r}_{\mathrm{CIo}}\big(\mathrm{C_O}^0\big) - \nu^{f}_{\mathrm{CIo}}\big(\mathrm{C_I}^0\big) - \nu^{r}_{\mathrm{CKi}}\big((\mathrm{D_I}^0 + \mathrm{D_I}^1 + \mathrm{D_I}^2 + \mathrm{D_I}^3)\mathrm{C_I}^0\big)$$

$$\frac{d\mathrm{C_I}^1}{dt} = \nu^{f}_{\mathrm{CKi}}\big(\mathrm{T_I}^{01} + \mathrm{T_I}^{11} + \mathrm{T_I}^{21} + \mathrm{T_I}^{31}\big) + \nu^{r}_{\mathrm{CIo}}\big(\mathrm{C_O}^1\big) - \nu^{f}_{\mathrm{CIo}}\big(\mathrm{C_I}^1\big) - \nu^{r}_{\mathrm{CKi}}\big((\mathrm{D_I}^0 + \mathrm{D_I}^1 + \mathrm{D_I}^2 + \mathrm{D_I}^3)\mathrm{C_I}^1\big)$$

$$\frac{d\mathrm{C_I}^2}{dt} = \nu^{f}_{\mathrm{CKi}}\big(\mathrm{T_I}^{02} + \mathrm{T_I}^{12} + \mathrm{T_I}^{22} + \mathrm{T_I}^{32}\big) + \nu^{r}_{\mathrm{CIo}}\big(\mathrm{C_O}^2\big) - \nu^{f}_{\mathrm{CIo}}\big(\mathrm{C_I}^2\big) - \nu^{r}_{\mathrm{CKi}}\big((\mathrm{D_I}^0 + \mathrm{D_I}^1 + \mathrm{D_I}^2 + \mathrm{D_I}^3)\mathrm{C_I}^2\big)$$

$$\frac{d\mathrm{C_I}^3}{dt} = \nu^{f}_{\mathrm{CKi}}\big(\mathrm{T_I}^{03} + \mathrm{T_I}^{13} + \mathrm{T_I}^{23} + \mathrm{T_I}^{33}\big) + \nu^{r}_{\mathrm{CIo}}\big(\mathrm{C_O}^3\big) - \nu^{f}_{\mathrm{CIo}}\big(\mathrm{C_I}^3\big) - \nu^{r}_{\mathrm{CKi}}\big((\mathrm{D_I}^0 + \mathrm{D_I}^1 + \mathrm{D_I}^2 + \mathrm{D_I}^3)\mathrm{C_I}^3\big)$$

$$\frac{d\mathrm{C_O}^0}{dt} = \nu^{f}_{\mathrm{CKo}}\big(\mathrm{T_O}^{00} + \mathrm{T_O}^{10} + \mathrm{T_O}^{20} + \mathrm{T_O}^{30}\big) + \nu^{f}_{\mathrm{CIo}}\big(\mathrm{C_I}^0\big) - \nu^{r}_{\mathrm{CKo}}\big((\mathrm{D_O}^0 + \mathrm{D_O}^1 + \mathrm{D_O}^2 + \mathrm{D_O}^3)\mathrm{C_O}^0\big) - \nu^{r}_{\mathrm{CIo}}\big(\mathrm{C_O}^0\big)$$

$$\frac{d\mathrm{C_O}^1}{dt} = \nu^{f}_{\mathrm{CKo}}\big(\mathrm{T_O}^{01} + \mathrm{T_O}^{11} + \mathrm{T_O}^{21} + \mathrm{T_O}^{31}\big) + \nu^{f}_{\mathrm{CIo}}\big(\mathrm{C_I}^1\big) - \nu^{r}_{\mathrm{CKo}}\big((\mathrm{D_O}^0 + \mathrm{D_O}^1 + \mathrm{D_O}^2 + \mathrm{D_O}^3)\mathrm{C_O}^1\big) - \nu^{r}_{\mathrm{CIo}}\big(\mathrm{C_O}^1\big)$$

$$\frac{d\mathrm{C_O}^2}{dt} = \nu^{f}_{\mathrm{CKo}}\big(\mathrm{T_O}^{02} + \mathrm{T_O}^{12} + \mathrm{T_O}^{22} + \mathrm{T_O}^{32}\big) + \nu^{f}_{\mathrm{CIo}}\big(\mathrm{C_I}^2\big) - \nu^{r}_{\mathrm{CKo}}\big((\mathrm{D_O}^0 + \mathrm{D_O}^1 + \mathrm{D_O}^2 + \mathrm{D_O}^3)\mathrm{C_O}^2\big) - \nu^{r}_{\mathrm{CIo}}\big(\mathrm{C_O}^2\big)$$

$$\frac{d\mathrm{C_O}^3}{dt} = \nu^{f}_{\mathrm{CKo}}\big(\mathrm{T_O}^{03} + \mathrm{T_O}^{13} + \mathrm{T_O}^{23} + \mathrm{T_O}^{33}\big) + \nu^{f}_{\mathrm{CIo}}\big(\mathrm{C_I}^3\big) - \nu^{r}_{\mathrm{CKo}}\big((\mathrm{D_O}^0 + \mathrm{D_O}^1 + \mathrm{D_O}^2 + \mathrm{D_O}^3)\mathrm{C_O}^3\big) - \nu^{r}_{\mathrm{CIo}}\big(\mathrm{C_O}^3\big)$$

$$\frac{d\mathrm{P_E}^0}{dt} = \nu^{f}_{\mathrm{ASe}}\big((\mathrm{T_E}^{00} + \mathrm{T_E}^{10} + \mathrm{T_E}^{20} + \mathrm{T_E}^{30})\mathrm{W_E}^0\big) + \nu^{r}_{\mathrm{PEo}}\big(\mathrm{P_O}^0\big) - \nu^{f}_{\mathrm{PEo}}\big(\mathrm{P_E}^0\big) - \nu^{r}_{\mathrm{ASe}}\big((\mathrm{D_E}^0 + \mathrm{D_E}^1 + \mathrm{D_E}^2 + \mathrm{D_E}^3)\mathrm{P_E}^0\big)$$

$$\frac{d\mathrm{P_E}^1}{dt} = \nu^{f}_{\mathrm{ASe}}\big((\mathrm{T_E}^{00} + \mathrm{T_E}^{10} + \mathrm{T_E}^{20} + \mathrm{T_E}^{30})\mathrm{W_E}^1 + (\mathrm{T_E}^{01} + \mathrm{T_E}^{11} + \mathrm{T_E}^{21} + \mathrm{T_E}^{31})\mathrm{W_E}^0\big) + \nu^{r}_{\mathrm{PEo}}\big(\mathrm{P_O}^1\big) - \nu^{f}_{\mathrm{PEo}}\big(\mathrm{P_E}^1\big) - \nu^{r}_{\mathrm{ASe}}\big((\mathrm{D_E}^0 + \mathrm{D_E}^1 + \mathrm{D_E}^2 + \mathrm{D_E}^3)\mathrm{P_E}^1\big)$$

$$\frac{d\mathrm{P_E}^2}{dt} = \nu^{f}_{\mathrm{ASe}}\big((\mathrm{T_E}^{01} + \mathrm{T_E}^{11} + \mathrm{T_E}^{21} + \mathrm{T_E}^{31})\mathrm{W_E}^1 + (\mathrm{T_E}^{02} + \mathrm{T_E}^{12} + \mathrm{T_E}^{22} + \mathrm{T_E}^{32})\mathrm{W_E}^0\big) + \nu^{r}_{\mathrm{PEo}}\big(\mathrm{P_O}^2\big) - \nu^{f}_{\mathrm{PEo}}\big(\mathrm{P_E}^2\big) - \nu^{r}_{\mathrm{ASe}}\big((\mathrm{D_E}^0 + \mathrm{D_E}^1 + \mathrm{D_E}^2 + \mathrm{D_E}^3)\mathrm{P_E}^2\big)$$

$$\frac{d\mathrm{P_E}^3}{dt} = \nu^{f}_{\mathrm{ASe}}\big((\mathrm{T_E}^{02} + \mathrm{T_E}^{12} + \mathrm{T_E}^{22} + \mathrm{T_E}^{32})\mathrm{W_E}^1 + (\mathrm{T_E}^{03} + \mathrm{T_E}^{13} + \mathrm{T_E}^{23} + \mathrm{T_E}^{33})\mathrm{W_E}^0\big) + \nu^{r}_{\mathrm{PEo}}\big(\mathrm{P_O}^3\big) - \nu^{f}_{\mathrm{PEo}}\big(\mathrm{P_E}^3\big) - \nu^{r}_{\mathrm{ASe}}\big((\mathrm{D_E}^0 + \mathrm{D_E}^1 + \mathrm{D_E}^2 + \mathrm{D_E}^3)\mathrm{P_E}^3\big)$$

$$\frac{d\mathrm{P_E}^4}{dt} = \nu^{f}_{\mathrm{ASe}}\big((\mathrm{T_E}^{03} + \mathrm{T_E}^{13} + \mathrm{T_E}^{23} + \mathrm{T_E}^{33})\mathrm{W_E}^1\big) + \nu^{r}_{\mathrm{PEo}}\big(\mathrm{P_O}^4\big) - \nu^{f}_{\mathrm{PEo}}\big(\mathrm{P_E}^4\big) - \nu^{r}_{\mathrm{ASe}}\big((\mathrm{D_E}^0 + \mathrm{D_E}^1 + \mathrm{D_E}^2 + \mathrm{D_E}^3)\mathrm{P_E}^4\big)$$

$$\frac{d\mathrm{P_M}^0}{dt} = \nu^{f}_{\mathrm{POm}}\big(\mathrm{P_O}^0\big) + \nu^{r}_{\mathrm{PMs}}\big(\mathrm{P_S}^0\big) - \nu^{f}_{\mathrm{PMs}}\big(\mathrm{P_M}^0\big)$$

$$\frac{d\mathrm{P_M}^1}{dt} = \nu^{f}_{\mathrm{POm}}\big(\mathrm{P_O}^1\big) + \nu^{r}_{\mathrm{PMs}}\big(\mathrm{P_S}^1\big) - \nu^{f}_{\mathrm{PMs}}\big(\mathrm{P_M}^1\big)$$

$$\frac{d\mathrm{P_M}^2}{dt} = \nu^{f}_{\mathrm{POm}}\big(\mathrm{P_O}^2\big) + \nu^{r}_{\mathrm{PMs}}\big(\mathrm{P_S}^2\big) - \nu^{f}_{\mathrm{PMs}}\big(\mathrm{P_M}^2\big)$$

$$\frac{d\mathrm{P_M}^3}{dt} = \nu^{f}_{\mathrm{POm}}\big(\mathrm{P_O}^3\big) + \nu^{r}_{\mathrm{PMs}}\big(\mathrm{P_S}^3\big) - \nu^{f}_{\mathrm{PMs}}\big(\mathrm{P_M}^3\big)$$

$$\frac{d\mathrm{P_M}^4}{dt} = \nu^{f}_{\mathrm{POm}}\big(\mathrm{P_O}^4\big) + \nu^{r}_{\mathrm{PMs}}\big(\mathrm{P_S}^4\big) - \nu^{f}_{\mathrm{PMs}}\big(\mathrm{P_M}^4\big)$$

$$\frac{d\mathrm{P_O}^0}{dt} = \nu^{f}_{\mathrm{PEo}}\big(\mathrm{P_E}^0\big) - \nu^{f}_{\mathrm{POm}}\big(\mathrm{P_O}^0\big) - \nu^{r}_{\mathrm{PEo}}\big(\mathrm{P_O}^0\big)$$

$$\frac{d\mathrm{P_O}^1}{dt} = \overset{\nu_f}{\mathrm{PEO}}\big(\mathrm{P_E}^1\big) - \overset{\nu_f}{\mathrm{POM}}\big(\mathrm{P_O}^1\big) - \overset{\nu_r}{\mathrm{PEO}}\big(\mathrm{P_O}^1\big)$$

$$\frac{d\mathrm{P_O}^2}{dt} = \overset{\nu_f}{\mathrm{PEO}}\big(\mathrm{P_E}^2\big) - \overset{\nu_f}{\mathrm{POM}}\big(\mathrm{P_O}^2\big) - \overset{\nu_r}{\mathrm{PEO}}\big(\mathrm{P_O}^2\big)$$

$$\frac{d\mathrm{P_O}^3}{dt} = \overset{\nu_f}{\mathrm{PEO}}\big(\mathrm{P_E}^3\big) - \overset{\nu_f}{\mathrm{POM}}\big(\mathrm{P_O}^3\big) - \overset{\nu_r}{\mathrm{PEO}}\big(\mathrm{P_O}^3\big)$$

$$\frac{d\mathrm{P_O}^4}{dt} = \overset{\nu_f}{\mathrm{PEO}}\big(\mathrm{P_E}^4\big) - \overset{\nu_f}{\mathrm{POM}}\big(\mathrm{P_O}^4\big) - \overset{\nu_r}{\mathrm{PEO}}\big(\mathrm{P_O}^4\big)$$

$$\frac{d\mathrm{P_S}^0}{dt} = \overset{\nu_f}{\mathrm{PMS}}\big(\mathrm{P_M}^0\big) + \overset{\nu_r}{\mathrm{ASs}}\Big(\big(\mathrm{T_S}^{00} + \mathrm{T_S}^{10} + \mathrm{T_S}^{20} + \mathrm{T_S}^{30}\big)\mathrm{W_S}^0\Big) - \overset{\nu_f}{\mathrm{ASs}}\Big(\big(\mathrm{D_S}^0 + \mathrm{D_S}^1 + \mathrm{D_S}^2 + \mathrm{D_S}^3\big)\mathrm{P_S}^0\Big) - \overset{\nu_r}{\mathrm{PMS}}\big(\mathrm{P_S}^0\big)$$

$$\frac{d\mathrm{P_S}^1}{dt} = \overset{\nu_f}{\mathrm{PMS}}\big(\mathrm{P_M}^1\big) + \overset{\nu_r}{\mathrm{ASs}}\Big(\big(\mathrm{T_S}^{00}+\mathrm{T_S}^{10}+\mathrm{T_S}^{20}+\mathrm{T_S}^{30}\big)\mathrm{W_S}^1+\big(\mathrm{T_S}^{01}+\mathrm{T_S}^{11}+\mathrm{T_S}^{21}+\mathrm{T_S}^{31}\big)\mathrm{W_S}^0\Big) - \overset{\nu_f}{\mathrm{ASs}}\Big(\big(\mathrm{D_S}^0+\mathrm{D_S}^1+\mathrm{D_S}^2+\mathrm{D_S}^3\big)\mathrm{P_S}^1\Big) - \overset{\nu_r}{\mathrm{PMS}}\big(\mathrm{P_S}^1\big)$$

$$\frac{d\mathrm{P_S}^2}{dt} = \overset{\nu_f}{\mathrm{PMS}}\big(\mathrm{P_M}^2\big) + \overset{\nu_r}{\mathrm{ASs}}\Big(\big(\mathrm{T_S}^{01}+\mathrm{T_S}^{11}+\mathrm{T_S}^{21}+\mathrm{T_S}^{31}\big)\mathrm{W_S}^1+\big(\mathrm{T_S}^{02}+\mathrm{T_S}^{12}+\mathrm{T_S}^{22}+\mathrm{T_S}^{32}\big)\mathrm{W_S}^0\Big) - \overset{\nu_f}{\mathrm{ASs}}\Big(\big(\mathrm{D_S}^0+\mathrm{D_S}^1+\mathrm{D_S}^2+\mathrm{D_S}^3\big)\mathrm{P_S}^2\Big) - \overset{\nu_r}{\mathrm{PMS}}\big(\mathrm{P_S}^2\big)$$

$$\frac{d\mathrm{P_S}^3}{dt} = \overset{\nu_f}{\mathrm{PMS}}\big(\mathrm{P_M}^3\big) + \overset{\nu_r}{\mathrm{ASs}}\Big(\big(\mathrm{T_S}^{02}+\mathrm{T_S}^{12}+\mathrm{T_S}^{22}+\mathrm{T_S}^{32}\big)\mathrm{W_S}^1+\big(\mathrm{T_S}^{03}+\mathrm{T_S}^{13}+\mathrm{T_S}^{23}+\mathrm{T_S}^{33}\big)\mathrm{W_S}^0\Big) - \overset{\nu_f}{\mathrm{ASs}}\Big(\big(\mathrm{D_S}^0+\mathrm{D_S}^1+\mathrm{D_S}^2+\mathrm{D_S}^3\big)\mathrm{P_S}^3\Big) - \overset{\nu_r}{\mathrm{PMS}}\big(\mathrm{P_S}^3\big)$$

$$\frac{d\mathrm{P_S}^4}{dt} = \overset{\nu_f}{\mathrm{PMS}}\big(\mathrm{P_M}^4\big) + \overset{\nu_r}{\mathrm{ASs}}\Big(\big(\mathrm{T_S}^{03} + \mathrm{T_S}^{13} + \mathrm{T_S}^{23} + \mathrm{T_S}^{33}\big)\mathrm{W_S}^1\Big) - \overset{\nu_f}{\mathrm{ASs}}\Big(\big(\mathrm{D_S}^0 + \mathrm{D_S}^1 + \mathrm{D_S}^2 + \mathrm{D_S}^3\big)\mathrm{P_S}^4\Big) - \overset{\nu_r}{\mathrm{PMS}}\big(\mathrm{P_S}^4\big)$$

$$\frac{d\mathrm{W_E}^0}{dt} = \overset{\nu_r}{\mathrm{ASe}}\Big(\big(\mathrm{D_E}^0 + \mathrm{D_E}^1 + \mathrm{D_E}^2 + \mathrm{D_E}^3\big)\big(\mathrm{P_E}^0 + \tfrac{1}{2}\mathrm{P_E}^2 + \tfrac{1}{4}\mathrm{P_E}^3 + \tfrac{3}{4}\mathrm{P_E}^1\big)\Big) + \overset{\nu_r}{\mathrm{WEO}}\big(\mathrm{W_O}^0\big) - \overset{\nu_f}{\mathrm{ASe}}\Big(\big(\mathrm{T_E}^{00} + \mathrm{T_E}^{01} + \mathrm{T_E}^{02} + \mathrm{T_E}^{03} + \mathrm{T_E}^{10} + \mathrm{T_E}^{11} + \mathrm{T_E}^{12} + \mathrm{T_E}^{13} + \mathrm{T_E}^{20} + \mathrm{T_E}^{21} + \mathrm{T_E}^{22} + \mathrm{T_E}^{23} + \mathrm{T_E}^{30} + \mathrm{T_E}^{31} + \mathrm{T_E}^{32} + \mathrm{T_E}^{33}\big)\mathrm{W_E}^0\Big) - \overset{\nu_f}{\mathrm{WEO}}\big(\mathrm{W_E}^0\big)$$

$$\frac{d\mathrm{W_E}^1}{dt} = \overset{\nu_r}{\mathrm{ASe}}\Big(\big(\mathrm{D_E}^0 + \mathrm{D_E}^1 + \mathrm{D_E}^2 + \mathrm{D_E}^3\big)\big(\mathrm{P_E}^4 + \tfrac{1}{2}\mathrm{P_E}^2 + \tfrac{1}{4}\mathrm{P_E}^1 + \tfrac{3}{4}\mathrm{P_E}^3\big)\Big) + \overset{\nu_r}{\mathrm{WEO}}\big(\mathrm{W_O}^1\big) - \overset{\nu_f}{\mathrm{ASe}}\Big(\big(\mathrm{T_E}^{00} + \mathrm{T_E}^{01} + \mathrm{T_E}^{02} + \mathrm{T_E}^{03} + \mathrm{T_E}^{10} + \mathrm{T_E}^{11} + \mathrm{T_E}^{12} + \mathrm{T_E}^{13} + \mathrm{T_E}^{20} + \mathrm{T_E}^{21} + \mathrm{T_E}^{22} + \mathrm{T_E}^{23} + \mathrm{T_E}^{30} + \mathrm{T_E}^{31} + \mathrm{T_E}^{32} + \mathrm{T_E}^{33}\big)\mathrm{W_E}^1\Big) - \overset{\nu_f}{\mathrm{WEO}}\big(\mathrm{W_E}^1\big)$$

$$\frac{d\mathrm{W_S}^0}{dt} = \overset{\nu_f}{\mathrm{ASs}}\Big(\big(\mathrm{D_S}^0 + \mathrm{D_S}^1 + \mathrm{D_S}^2 + \mathrm{D_S}^3\big)\big(\mathrm{P_S}^0 + \tfrac{1}{2}\mathrm{P_S}^2 + \tfrac{1}{4}\mathrm{P_S}^3 + \tfrac{3}{4}\mathrm{P_S}^1\big)\Big) + \overset{\nu_f}{\mathrm{WOS}}\big(\mathrm{W_O}^0\big) - \overset{\nu_r}{\mathrm{ASs}}\Big(\big(\mathrm{T_S}^{00} + \mathrm{T_S}^{01} + \mathrm{T_S}^{02} + \mathrm{T_S}^{03} + \mathrm{T_S}^{10} + \mathrm{T_S}^{11} + \mathrm{T_S}^{12} + \mathrm{T_S}^{13} + \mathrm{T_S}^{20} + \mathrm{T_S}^{21} + \mathrm{T_S}^{22} + \mathrm{T_S}^{23} + \mathrm{T_S}^{30} + \mathrm{T_S}^{31} + \mathrm{T_S}^{32} + \mathrm{T_S}^{33}\big)\mathrm{W_S}^0\Big) - \overset{\nu_r}{\mathrm{WOS}}\big(\mathrm{W_S}^0\big)$$

$$\frac{d\mathrm{W_S}^1}{dt} = \overset{\nu_f}{\mathrm{ASs}}\Big(\big(\mathrm{D_S}^0 + \mathrm{D_S}^1 + \mathrm{D_S}^2 + \mathrm{D_S}^3\big)\big(\mathrm{P_S}^4 + \tfrac{1}{2}\mathrm{P_S}^2 + \tfrac{1}{4}\mathrm{P_S}^1 + \tfrac{3}{4}\mathrm{P_S}^3\big)\Big) + \overset{\nu_f}{\mathrm{WOS}}\big(\mathrm{W_O}^1\big) - \overset{\nu_r}{\mathrm{ASs}}\Big(\big(\mathrm{T_S}^{00} + \mathrm{T_S}^{01} + \mathrm{T_S}^{02} + \mathrm{T_S}^{03} + \mathrm{T_S}^{10} + \mathrm{T_S}^{11} + \mathrm{T_S}^{12} + \mathrm{T_S}^{13} + \mathrm{T_S}^{20} + \mathrm{T_S}^{21} + \mathrm{T_S}^{22} + \mathrm{T_S}^{23} + \mathrm{T_S}^{30} + \mathrm{T_S}^{31} + \mathrm{T_S}^{32} + \mathrm{T_S}^{33}\big)\mathrm{W_S}^1\Big) - \overset{\nu_r}{\mathrm{WOS}}\big(\mathrm{W_S}^1\big)$$
